# A physicochemical orthophosphate cycle via a kinetically stable thermodynamically activated intermediate enables mild prebiotic phosphorylations

**DOI:** 10.1038/s41467-021-25555-x

**Published:** 2021-09-17

**Authors:** Oliver R. Maguire, Iris B. A. Smokers, Wilhelm T. S. Huck

**Affiliations:** Institute for Molecules and Materials, Radboud University Nijmegen, 6525 AJ Nijmegen, The Netherlands

**Keywords:** Origin of life, Inorganic chemistry

## Abstract

The incorporation of orthophosphate from scarce geochemical sources into the organic compounds essential for life under mild conditions is a fundamental challenge for prebiotic chemistry. Here we report a prebiotic system capable of overcoming this challenge by taking inspiration from extant life’s recycling of orthophosphate via its conversion into kinetically stable thermodynamically activated (KSTA) nucleotide triphosphates (e.g. ATP). We separate the activation of orthophosphate from its transfer to organic compounds by, crucially, first accumulating a KSTA phosphoramidate. We use cyanate to activate orthophosphate in aqueous solution under mild conditions and then react it with imidazole to accumulate the KSTA imidazole phosphate. In a paste, imidazole phosphate phosphorylates all the essential building blocks of life. Integration of this chemistry into a wet/dry cycle enables the continuous recycling of orthophosphate and the accretion of phosphorylated compounds. This system functions even at low reagent concentrations due to solutes concentrating during evaporation. Our system demonstrates a general strategy for how to maximise the usage of scarce resources based upon cycles which accumulate and then release activated intermediates.

## Abstract (web summary)

Transfer of scarce phosphate to organic molecules is a significant challenge for prebiotic chemistry. Here authors show a prebiotic physicochemical cycle to activate orthophosphate and via a kinetically stable, thermodynamically activated molecule phosphorylate all of life’s basic building blocks.

## Introduction

Phosphorylated molecules play a series of diverse and essential roles in extant biology, including driving endergonic reactions via the release of free energy upon phosphate transfer, preserving genetic information due to the hydrolytic stability of phosphate diesters in the backbone of DNA and retaining cellular contents by the presence of a charged phosphate head group on both the phospholipids in the cell membrane and on compounds in the cytoplasm^1,2^. Given the centrality of phosphate chemistry to life, there has been widespread interest in establishing plausible pathways for the formation of prebiotically important phosphorylated organic molecules^3–5^.

On the early Earth, geochemical sources of orthophosphate were scarce and were most likely found in minerals such as apatite, vivianite, berlinite and struvite, or from the hydrolysis of phosphorus-containing minerals such as schreibersite^3^. In order for orthophosphate to be available for transfer to organic compounds, it will require its release from a mineral, which will most probably arise from dissolution of the mineral into solution. It is notable that in aqueous solution, the most thermodynamically stable form of phosphorus is orthophosphate^6^. Transfer of orthophosphate from these geochemical sources to prebiotically important organic compounds is therefore a key requirement for the origins of life. However, previous examples of prebiotic phosphorylation reactions, which use orthophosphate directly, require aggressive heating conditions in conjunction with an activating reagent to overcome the high kinetic barrier for the activation of orthophosphate^7–14^ and/or the use of non-aqueous eutectic solvents such as urea and formamide^15,16^. In particular, aggressive heating conditions (typically at 100 °C) can have deleterious effects on other molecules through either hydrolysis or other unwanted side reactions and are likely incompatible with any form of protocellular chemistry. Alternative approaches to prebiotic phosphorylation have exploited activated sources of phosphate. Phosphoramidates, including diamidophosphate (DAP), have been highlighted as powerful prebiotic phosphorylation reagents^17–23^. However, a prebiotically relevant method to directly recycle orthophosphate back into DAP has not been demonstrated. Finally, given the challenges of using orthophosphate, alternative sources of phosphorus, e.g., phosphite^6,24^ and cyclopolyphosphates^25^, have also been explored.

Considering the challenges posed by activating orthophosphate and transferring it in a single reaction step, we were inspired by extant life’s biochemistry, which is reliant upon the ability of cells to continuously recycle orthophosphate back into an activated form. This cycling of orthophosphate is mediated by nucleotide triphosphates (NTPs), e.g. ATP, which preserve a kinetically stable thermodynamically activated (KSTA) form of phosphate^26–29^.

Here we activate orthophosphate with cyanate in aqueous solution to form carbamoyl phosphate under mild prebiotically plausible conditions. We demonstrate that this activated phosphate can be captured with imidazole to form imidazole phosphate, a KSTA molecule. Imidazole phosphate can accumulate in solution, as it is resistant to both hydrolysis and reactions with most other nucleophiles. We show that in a paste, imidazole phosphate can transfer the phosphate group to a wide range of prebiotically important organic compounds. We then adopt a systems chemistry approach^30,31^ whereby we demonstrate a prebiotically plausible physicochemical orthophosphate cycle by integrating this chemistry into a wet/dry cycle. The cycle is ‘driven’ by repeatedly ‘refuelling’ with fresh cyanate solution and each pass through the cycle results in a stepwise increase in phosphorylated compounds. Finally, we show how the process of solutes being concentrated as a solution evaporates in the wet/dry cycle allows us to exploit significantly lower concentrations (10–20 mM) of cyanate, imidazole, orthophosphate and a nucleophile, and still obtain phosphorylated compounds. Our results illustrate a general strategy for how to construct a system, which maximizes the usage of scarce resources by capturing, storing and accumulating activated versions of these compounds in a KSTA molecule whose chemical energy can be later channelled towards useful functions.

## Results

### The formation and accumulation of imidazole phosphate in mild conditions

**Fig. 1:**
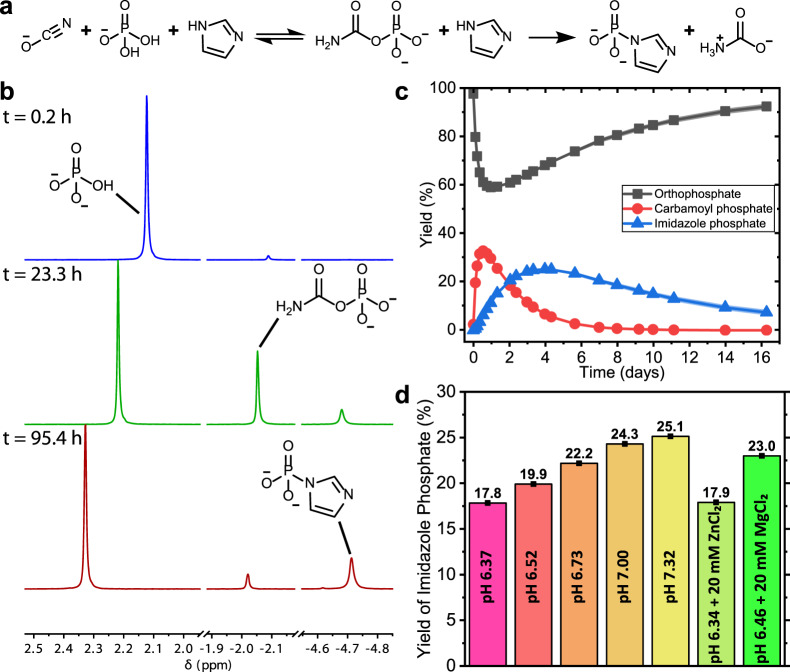
The formation of imidazole phosphate in mild prebiotic conditions. **a** The reaction steps for the formation of imidazole phosphate from orthophosphate, cyanate and imidazole. Cyanate isomerizes to isocyanate prior to reacting with orthophosphate to form carbamoyl phosphate. **b** Representative ^31^P-NMR spectra over time for the formation of imidazole phosphate from 450 mM potassium cyanate + 150 mM sodium phosphate in 1.0 M imidazole buffer at 22 °C and pH 7.32. A minor increase in pH over the course of the reaction causes the change in chemical shift of peaks. **c** Profile of the changing yield of phosphorus-containing species over time for the reaction shown in **b**. Shaded area around traces is the SD from triplicate experiments. **d** The effect of pH and metal ions on the yield of imidazole phosphate. All reactions in **d** used identical concentrations as detailed in **b**.

We reasoned that we could activate orthophosphate by converting it into carbamoyl phosphate through a reaction with isocyanate^32,33^ and then capture the activated phosphate with imidazole to form imidazole phosphate (Fig. 1a). In a solution containing 450 mM potassium cyanate, 150 mM sodium phosphate monobasic and 1.0 M imidazole at pH 7.32, we observed by ^31^P nuclear magnetic resonance (NMR) spectroscopy the accumulation of imidazole phosphate in a yield of 25% after 4 days at 22 °C (Fig. 1b, c). Initially, carbamoyl phosphate was formed and this was converted into imidazole phosphate via substitution reaction with imidazole (Fig. 1b, c). Separate experiments confirmed that imidazole phosphate was formed directly from carbamoyl phosphate (Supplementary Information (SI) Section S2.3). Excess cyanate concentration relative to orthophosphate was added, in order to push the equilibrium toward the formation of carbamoyl phosphate^32,33^. After 3 days, the concentration of imidazole phosphate decreased slowly over time due to hydrolysis (Fig. 1c). Imidazole phosphate was formed in yields of between 18% and 25% across pH 6.37–7.32 and the addition of metal ions Mg^2+^ and Zn^2+^ (typically used as Lewis acid catalysts in prebiotic chemistry) did not significantly affect the yield of imidazole phosphate formed (Fig. 1d and SI Sections S2.2 and S2.3). Under identical conditions, other substituted imidazoles and pyrazole also formed their respective imidazole phosphates, albeit in a lower yield than imidazole (5–20%), thereby demonstrating the generality of this approach for capturing activated phosphate (SI Section S2.4).

Imidazole phosphate is a good candidate for a prebiotically plausible KSTA molecule. Imidazole phosphate has reasonable kinetic stability towards hydrolysis with a half-life of *t*_1/2_ = 23.1 h at pH 7.0 and 39 °C^34,35^. Whether imidazole phosphate can be regarded as thermodynamically activated depends upon whether phosphate transfer from imidazole phosphate to a nucleophile is thermodynamically favoured. An initial assessment of this can be done by comparing the Gibbs free energies of hydrolysis (Δ*G*_hyd_) of imidazole phosphate with that of the phosphorylated nucleophile. The hydrolysis of imidazole phosphate has a Δ*G*_hyd_[pH 7.4, 25 °C] = −32.0 kJ mol^−1^^ 36^. Thus, if Δ*G*_hyd_ > −32.0 kJ mol^−1^ for the phosphorylated nucleophile, then phosphate transfer is favourable and the imidazole phosphate can be described as thermodynamically activated. For example, glycerol-1-phosphate has Δ*G*_hyd_ = −9.2 kJ mol^−1^ and, therefore, imidazole phosphate is thermodynamically activated relative to glycerol^37^. Various prebiotic syntheses of imidazole are known^38,39^.

### Phosphate transfer to the essential building blocks of life in a paste

Next, we set out to confirm whether imidazole phosphate would be kinetically stable towards a wide range of prebiotically relevant organic compounds in aqueous solution. Amino acids, nucleosides, nucleotides and sugars were added to solutions containing synthetically prepared calcium/sodium imidazole phosphate^40^ (SI Section S3) in citric acid buffered solutions at pH 3.0–6.9 and 22 °C (see SI Section S4.1 and Supplementary Table 1). In all bar two cases with hydroxylamines (see SI Section S4), no transfer of phosphate to these nucleophiles was observed in solution and only a slow hydrolysis of imidazole phosphate took place. Overall, this confirmed that in solution, imidazole phosphate is kinetically stable towards almost all organic nucleophiles as well as to hydrolysis. Thus, activated phosphate could be accumulated in solution in the form of imidazole phosphate.

**Fig. 2:**
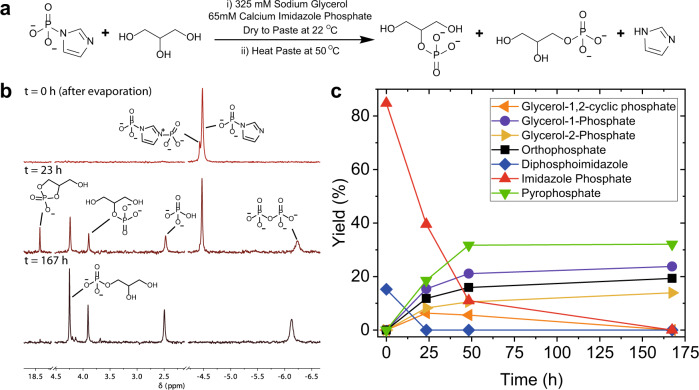
The phosphorylation of glycerol by imidazole phosphate in paste conditions. **a** The phosphorylation reaction of glycerol by calcium imidazole phosphate in paste conditions after drying to a paste using the second method. **b** Representative ^31^P-NMR spectra over time for the reaction of 0.13 mmol calcium imidazole phosphate and 0.65 mmol glycerol in paste conditions at 50 °C. The paste was prepared by leaving 2.0 mL of a 65 mM calcium imidazole phosphate and 325 mM glycerol solution to dry for 48 h at 22 °C. The ^31^P-NMR spectrum at *t* = 0 h was taken from the paste after the evaporation of water. During the drying stage diphosphoimidazole formed from the reaction of imidazole phosphate with itself. **c** Yields of phosphate-containing compounds over time for the reaction in **a** and **b**.

Prebiotic catalysts for phosphate transfer reactions in solution that are analogous to the role of kinases in extant life are currently unknown, but we reasoned that by switching to paste conditions, where the activity of water is decreased, we would be able to transfer the phosphate from imidazole phosphate to oxygen-based nucleophiles apart from water^3,20^. We conducted a series of paste reactions with a diverse set of prebiotically important organic molecules: amphiphile precursors^41^, amino acids/peptides^42–44^, metabolites^45,46^, nucleosides^47,48^, nucleotides and sugars^49^. We made the pastes either by adding a small amount of water to the solid compounds or by dissolving the solid compounds in water and then waiting for the solution to evaporate into a paste via a wet-to-dry transition. In the first set of paste reactions, we mixed solid calcium imidazole phosphate with solid/liquid nucleophile and added water (0.1−0.2 μL mg^−1^) to form a paste and the pastes were heated to 50 °C or 80 °C for between 24 and 168 h (1–7 days) upon which phosphate transfer was observed in yields of up to 52% (SI Sections S5 and S6.8, and Supplementary Table 2). Yields at 50 °C were typically better than at 80 °C. In the second method, we demonstrated that the phosphate transfer reactions could occur after a wet-to-dry transition (Table 1, Fig. 2, and SI Section S6). We dissolved calcium imidazole phosphate and the nucleophile in water, and left the solution to dry at 22 °C for 12–48 h. Notably, in several cases, the addition of the nucleophile to the solution aided the dissolution of calcium imidazole phosphate, presumably by coordinating to the Ca^2+^ ion. The presence of organic compounds in solution has, along with cyanide ions, previously been shown to help solubilize calcium phosphate minerals such as apatite^9,50^. The resultant paste was then heated to 50 °C, to promote phosphate transfer. The reactions were followed by the periodic removal of a sample from the paste and analysed using ^31^P-NMR spectroscopy. The transfer of the phosphate group from imidazole phosphate in the paste was relatively facile with most reactions going to completion after 24–72 h. In the paste, two other reactions were observed: the hydrolysis of imidazole phosphate and the formation of pyrophosphate^51^ via the reaction of imidazole phosphate with either itself or with orthophosphate. To counter the formation of pyrophosphate, we used a five-fold excess of the nucleophile. Spiking experiments with genuine samples of phosphorylated compounds confirmed the identity of phosphorylated compounds in the ^31^P-NMR spectra (SI Section S6).

**Table 1:** The amphiphile precursors, amino acids + peptides, metabolites, nucleosides, nucleotides and sugars phosphorylated by imidazole phosphate under paste conditions (see SI Section S6).

	Nucleophile	Nucleophile (mmol)	Calcium imidazole phosphate (mmol)	*T* (°C)	Duration (h)	Yield (%)
Amphiphile precursor	Glycerol	0.65	0.13	50	167	24% Glycerol-1-phosphate 14% Glycerol-2-phosphate (6% Glycerol-1,2-cyclic phosphate)^a^
Amino acids + peptides	Serine	0.65	0.13	50	46	17% *O*-phosphoserine
	SerOMe	0.65	0.13	50	169	9% *O*-phosphoserine 2% *O*-phosphoserine methyl ester
	ThrOMe	0.65	0.13	50	48	7% *O*-phosphothreonine
	Ser-His	0.33	0.13	50	46	6% *O*-phosphoSer-His
Metabolites	Glycerate (Ca^2+^ salt)	0.65	0.13	50	167	26% 2-Phosphoglycerate 8% 3-Phosphoglycerate
	Glycerate (Na^+^ salt)	0.65	0.13	50	23	9% 2-Phosphoglycerate 16% 3-Phosphoglycerate
	Lactate (Na^+^ salt)	0.65	0.13	50	24	10% 2-Phospholactate
	Glycolate (Na^+^ salt)	0.65	0.13	50	24	31% 2-Phosphoglycolate
	Lactamide	0.65	0.13	50	167	11% Phospholactamide 7% Pyrophospholactamide
Nucleosides	Adenosine	0.65	0.13	50	22	2% A-5′-MP
	Cytidine	0.65	0.13	50	93	7% C-2′-MP 4% C-3′-MP 3% C-5′-MP
	Guanosine	0.65	0.13	50	93	1% G-5′-MP
	Uridine	0.65	0.13	50	22	11% U-5′-MP 7% U-3′-MP
Nucleotides	AMP	0.65	0.13	50	4	20% ADP
	ADP	0.65	0.13	50	0^b^	18% ATP
	CMP	0.65	0.13	50	5	4% CDP
	CDP	0.65	0.13	50	4	12% CTP
	GMP	0.65	0.13	50	4	15% GDP
	GDP	0.65	0.13	50	0^b^	18% GTP
	UMP	0.65	0.13	50	4	4% UDP
	UDP	0.65	0.13	50	0^b^	25% UTP
Sugars	d-Ribose	0.65	0.13	50	23	69%^c^
	d-Glucose	0.65	0.13	50	24	70%^c^
	d-Glucose	0.65	0.13	50	48	33%^c^

^a^The formation of Glycerol-1,2-cyclic phosphate was observed after 24 h. However, this signal disappeared after heating for 167 h (see Supplementary Fig. 65).

^b^*t* = 0 h is after drying to paste.

^c^For ribose and glucose, multiple peaks in the ^31^P-NMR spectra for phosphorylated sugars were observed (see Supplementary Fig. 135–143). The yield quoted is the summation of integrals from all of these peaks.

A wide array of prebiotically important organic compounds were phosphorylated using imidazole phosphate in a paste reaction (Table 1). The amphiphile precursor glycerol phosphate, a key component for the formation of phospholipids with the two hydrophobic tails essential for bilayer formation^41^, was formed in a yield of 38%, with glycerol-1-phosphate and glycerol-2-phosphate formed in a 24% and 14% yield, respectively (Fig. 2). The hydroxyl side groups of amino acids and amino acid residues in peptides, such as serine, were phosphorylated in 6–17% yield. Organic compounds that in extant life function as metabolites and in prebiotic chemistry can act as phosphorylated building blocks were formed in a 10–34% yield. A precursor for phosphoenolpyruvate (biology’s highest-energy phosphate), 2-phosphoglycerate, was formed in a 9% yield. Nucleosides were converted into nucleotide monophosphates in up to an 18% yield. Sugars were phosphorylated in yields of 33–70% including ribose, an important precursor for ribonucleotides, and glucose. The phosphorylation of the sugars was not regioselective and the yields quoted in Table 1 reflect the combined yield of all phosphorylated sugar products. Spiking experiments confirmed the formation of ribose-1-phosphate, ribose-5-phosphate, glucose-1-phosphate and glucose-6-phosphate, but other phosphorylated sugars were also observed. In addition to phosphate monoesters, imidazole phosphate could also synthesize oligophosphates via the formation of a phosphate anhydride bond. Nucleotide-5’-monophosphates (NMPs) were converted into nucleotide diphosphates (NDPs) in a 4–20% yield and NDPs could be converted to NTPs in yields of between 3% and 25%, with the two NTPs predominantly used in cellular biochemistry, ATP and GTP, both being formed in an 18% yield. Control experiments performed in the absence of imidazole phosphate confirmed that the NDPs were formed exclusively from the reaction of the NMP with imidazole phosphate as opposed to phosphate transfer between two NMPs and control reactions with NDPs confirmed that either all or the majority of NTPs were formed from the reaction with imidazole phosphate (SI Section S6.6). The formation of NDPs and NTPs is particularly encouraging, as it provides a direct link between this prebiotic chemistry and the chemistry that life eventually settled on for the cycling of orthophosphate.

### A physicochemical orthophosphate cycle for prebiotic phosphorylations

**Fig. 3:**
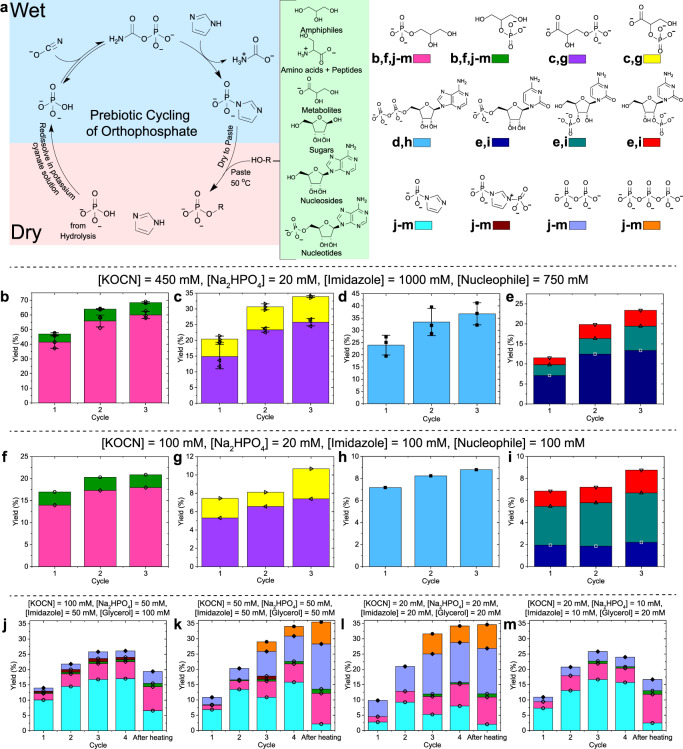
The physicochemical orthophosphate cycle, which upon repeated cycling enables a stepwise increase in the incorporation of orthophosphate into prebiotically important organic compounds. **a** Overview of the orthophosphate cycling experiment. The second and third cycles were performed by redissolving the paste in a solution with the respective potassium cyanate concentration for each set of experiments. The plots **b**–**m** show the yield of incorporation of orthophosphate into phosphorylated products over the course of three cycles and this is referred to as ‘Yield’ on the *y*-axis. **b**–**e** Experiments initiated from a solution of 450 mM potassium cyanate, 1.0 M imidazole and 20 mM sodium phosphate with 750 mM of the nucleophile (glycerol, glycerate, AMP and the nucleoside cytidine). Error bars are based on the SD determined from three repeats. **f**–**i** Experiments initiated from a solution of 100 mM potassium cyanate, 100 M imidazole and 20 mM sodium phosphate with 100 mM of the nucleophile (glycerol, glycerate, AMP and the nucleoside cytidine). **j**–**m** Wet/dry cycling experiments performed at lower initial concentrations in the presence of glycerol, which demonstrate the formation of imidazole phosphate as the solution concentrates while drying and the transfer of phosphate to the nucleophile in the paste. To emphasize the process of a solution concentrating as it dries, the yield of imidazole phosphate and all products that it forms are shown.

Prebiotic sources of free orthophosphate are likely to be limiting^3^ and thus demonstrating a prebiotically plausible route to recycle orthophosphate is crucial. We reasoned that we could construct such an orthophosphate cycle by integrating our above results together into a wet/dry cycle (Fig. 3a). In solution, orthophosphate can be activated via a reaction with cyanate and converted into imidazole phosphate. Upon drying to a paste and heating, the phosphate can be transferred from imidazole phosphate to prebiotically important organic compounds. The cycle can then be completed by redissolving the paste in a cyanate solution, thereby ‘refuelling’ the system to reactivate the remaining orthophosphate and regenerate imidazole phosphate. Repeated cycling would lead to the accumulation of phosphorylated compounds.

To demonstrate the orthophosphate cycling, we first prepared a 450 mM potassium cyanate, 1.0 M imidazole and 20 mM sodium phosphate solution with 750 mM of a nucleophile (glycerol, glycerate, serine, AMP or the nucleoside cytidine) (SI Section S7.1–S7.4 and S7.9). The pH of the solution was adjusted to pH 7.3 and left to react at 22 °C for 24 h, in order to accumulate imidazole phosphate. The solution was then dried to a paste at 22 °C over a period of 24 h. The resultant paste contained both imidazole phosphate and phosphorylated nucleophiles, thereby demonstrating that phosphate transfer from imidazole phosphate to the nucleophile occurred during the drying process. The paste was then heated to 50 °C for 24 h, to promote further phosphate transfer to the nucleophile. Pyrophosphate and triphosphate were formed as side products and hydrolysis of imidazole phosphate back to orthophosphate occurred via a reaction with residual water^51^. The cycle was completed by redissolving the paste in 450 mM potassium cyanate solution, in order to reactivate orthophosphate and accumulate imidazole phosphate once again. Three repetitions of the cycle were performed and the percentage of orthophosphate incorporated into the organic compounds at the end of each cycle (referred to as ‘Yield’ on the *y*-axis) are shown in Fig. 3b–e. A stepwise increase in phosphorylated compounds was observed predominantly between the first and second cycle for all the nucleophiles. Further cycling experiments with other nucleophiles including serine, d-ribose, d-glucose and the nucleoside adenosine showed similar trends (SI Section S7.5–S7.8). When the physicochemical cycle was performed with higher concentrations of orthophosphate of 50 mM and 150 mM, similar trends were also observed (see SI Section S7.2–S7.9). Previous reports have shown phosphate anhydride bond and phosphate monoester formation in cyanate and orthophosphate solutions, albeit usually in very poor yields^52–55^. To ascertain whether this was occurring in our experiments, we performed control reactions in the absence of imidazole (SI Section S7.10). These showed no formation of phosphorylated compounds (or pyrophosphate or triphosphate), thereby confirming that the reagent for phosphate transfer in the pastes was imidazole phosphate and not carbamoyl phosphate. We assume that differences in the conditions between our control experiments and previous studies account for the absence of phosphorylation under these circumstances.

The concentrations of potassium cyanate, imidazole and the nucleophile used in the above experiments were high. This was done in order to clearly observe experimentally the formation and accumulation of imidazole phosphate and identify how the reactions in the cycle proceeded. Nevertheless, such high concentrations are less prebiotically plausible. However, the concentration of any solution is relative to the volume of its solvent and we therefore reasoned that we could use the wet/dry cycle to provide a prebiotically plausible means to access higher concentrations. As solvent water evaporates, the concentration of solutes in solution increases. We therefore chose to perform the physicochemical orthophosphate cycling with concentrations of potassium cyanate, imidazole and a nucleophile at 100 mM level with a sodium phosphate concentration of 20 mM (Fig. 3f–i) and 50 mM (SI Section S7.11). An ∼100 mM concentration range is typical of that used in prebiotic chemistry^44,46,56^. Here, the stepwise increase in the incorporation of orthophosphate into phosphorylated nucleophiles still functions. For the different nucleophiles, the percentage of orthophosphate incorporated into the phosphorylated nucleophile after three cycles is on average only 3.2-fold lower than those for the higher concentrations above, despite the concentrations of cyanate, imidazole and nucleophile being lowered by between 4.5- and 10-fold. The cycle was also performed with serine as the nucleophile (SI Section S7.11.8 and S7.11.9).

Finally, we decided to probe whether this physicochemical orthophosphate cycle would still function well at much lower initial concentrations of potassium cyanate, orthophosphate, imidazole and the nucleophile glycerol. We prepared solutions with concentrations reduced by up to 100-fold compared to those above, with imidazole and sodium phosphate concentrations of 10–50 mM, and potassium cyanate and glycerol concentrations of 20–100 mM (Fig. 3j–m and SI Section S7.12). These solutions were left for ∼24 h before being dried to a paste. To demonstrate that imidazole phosphate can accumulate over the course of several cycles even at these much lower concentrations, we applied heating to the pastes (50 °C for 31 h) only after four cycles. Fig. 3j–m demonstrates that even at these low initial concentrations, the effect of a solution concentrating as it evaporates enabled the formation of imidazole phosphate and the transfer of the phosphate in the paste to the glycerol. For example, Fig. 3m, where reagents were in the 10–20 mM range, shows that 16% of the orthophosphate was accumulated into imidazole phosphate after four cycles, thereby demonstrating its ability to function as a KSTA molecule. In Fig. 3m, 5% of the orthophosphate was incorporated into phosphorylated glycerol over the four cycles, which was elevated to 11% after heating. In Fig. 3j–m, the yield of incorporation of the orthophosphate into phosphorylated glycerol at these lower concentrations was reduced compared to those experiments at the higher concentrations above, but we do not believe this to be a significant issue, as these wet/dry cycles could have occurred multiples times on the early Earth and the resulting phosphate monoester products generally have good hydrolytic stability, which will enable their accretion. Fig. 3j–m also include the side products diphosphoimidazole, pyrophosphate and triphosphate (which are all formed via imidazole phosphate) in order to demonstrate that even at these lower concentrations, overall between 26% and 35% of the orthophosphate was converted into imidazole phosphate over the course of four cycles. For Fig. 3j, m, after the heating was applied, the overall percentage incorporation of orthophosphate dropped due to hydrolysis of imidazole phosphate. The formation of these aforementioned side products was more significant when all reagents were present in an equivalent concentration (Fig. 3k, l) than when the concentrations of cyanate and glycerol were twice that of orthophosphate and imidazole (Fig. 3j, m). We therefore demonstrate here that even at lower initial concentrations of all reagents, this physicochemical orthophosphate cycle still functions well.

## Discussion

Here we have demonstrated a prebiotically plausible system by which orthophosphate can be activated, accumulated in a KSTA compound in solution, transferred to prebiotically important organic compounds in a paste and repeatedly recycled all within a wet/dry cycle (Fig. 3a). Orthophosphate was activated under mild conditions using cyanate to form carbamoyl phosphate and this activated phosphate was captured and stored via the formation of the phosphoramidate imidazole phosphate. The imidazole phosphate is kinetically stable towards hydrolysis and thus an activated form of phosphate can accumulate in solution as a KSTA molecule. Phosphate transfer from imidazole phosphate to all classes of the essential organic molecules necessary for the origins of life was demonstrated in paste conditions after a wet-to-dry transition. Integration of this chemistry into a wet/dry cycle by redissolving the paste in a cyanate solution recycled the remaining orthophosphate back into an activated form via the regeneration of imidazole phosphate. Repeated cycling led to an accretion of phosphorylated compounds and therefore can maximize the use of scarce resources such as orthophosphate. This physicochemical orthophosphate cycle could provide a continuous supply of phosphorylated organic compounds for prebiotic chemistry upon the early Earth.

The simplicity of this system makes it ideal for prebiotic chemistry. Only four simple, prebiotically plausible reagents (orthophosphate, cyanate, imidazole and a nucleophile) are required along with a wet/dry cycle, in order to perform all steps of the physicochemical orthophosphate cycle. Importantly, this approach uses orthophosphate—the most likely source of phosphorus on the early Earth and the most thermodynamically favoured oxidation state of phosphorus in aqueous solution^6,57,58^. Moreover, the activation of orthophosphate and its storage in imidazole phosphate occurs in aqueous solution under mild conditions at 22 °C and pH 7.0–8.0. In principle, this system is capable of capturing solar energy and storing it within imidazole phosphate as cyanate can be formed via a photocatalysed reaction between hydrogen cyanide and water in the presence of an [Fe(CN)_6_]^3−^ or [Cu_2_(CN)_6_]^2^^−^ catalyst^49,59^. Also, spark discharge experiments (simulating lightning) with N_2_, CO_2_ and H_2_ have demonstrated the formation of cyanate in up to 12 mM concentrations, which is in close alignment with the cyanate concentrations of 20 mM used in Fig. 3l, m^60^. The utilization of a wet/dry cycle is a well-established means by which to drive a range of prebiotically plausible reaction processes and allows this physicochemical orthophosphate cycle to function at much lower reagent concentrations by taking advantage of how solutes concentrate as solvent evaporates^47,48,61,62^.

To conclude, we have demonstrated a key strategy for how to construct prebiotically plausible chemical systems that capture chemical energy used for the activation of phosphate, store it within a KSTA molecule, which can accumulate, and then later channel that energy into the phosphorylation of the building blocks of life during a wet/dry cycle. More broadly, our approach to constructing the system harnesses the interplay between the kinetic and thermodynamic aspects of chemical reactions and the environmental processes which alter these factors. Considering the prominent role of other KSTA molecules in extant life, such as acetyl CoA, SAM and NAD(P)H, we believe that our strategy can be used to assemble other KSTA-based prebiotic systems with functions beyond phosphorylation. The KSTA molecules in extant life play an essential role in keeping a cell in an out-of-equilibrium state - a characteristic feature of living systems. Our demonstration that this KSTA strategy would be viable on the early Earth therefore shows how a prebiotic system could access and then persist in an out-of-equilibrium state, akin to life.

## Methods

### Formation of imidazole phosphate from cyanate, orthophosphate and imidazole in aqueous solution

A 450 mM potassium cyanate, 150 mM sodium phosphate and 1.0 M imidazole solution was prepared by dissolving 36.5 mg (0.45 mmol, 3 eq.) potassium cyanate and 26.7 mg (0.15 mmol, 1 eq.) sodium phosphate monobasic anhydrous in 1.0 mL of 1.0 M imidazole buffer (1.00 mmol, 6.67 eq.) containing 50 mM HMPA (hexamethylphosphoramide) internal standard at pH 6.17 in 9:1 H_2_O:D_2_O. The pH of the solution at the start was pH 6.37. The reaction was followed by ^31^P-NMR and ^1^H-NMR spectroscopy, measuring spectra at a series of time points over the course of 16 days. The pH of the sample was measured at time points over the course of the reaction. An identical procedure was followed for the reactions with starting pHs of 6.52, 6.73, 7.00 and 7.32. See SI Section S2.

### Phosphorylation of prebiotically important organic compounds after a wet-to-dry transition

Here, 24.2 mg (0.13 mmol, 1 eq.) of calcium imidazole phosphate (see SI Section S3 for the synthesis of calcium imidazole phosphate) and 98.5 mg (0.65 mmol, 5 eq.) of d-ribose were dissolved in 2.0 mL of MilliQ water to give a 65 mM calcium imidazole phosphate and a 325 mM d-ribose solution. The solution was added to a petri dish and left with the lid off to dry at 22 °C for 24 h. The resulting paste was scraped from the petri dish and placed into an Eppendorf. The Eppendorf was spun on a centrifuge for 5 min at 5590 × *g* in order to move all paste to the bottom of the Eppendorf and compact it together in order to remove air pockets introduced during the transfer of the paste to the Eppendorf. An ~10 mg sample of the paste was taken to record the extent of phosphorylation after the wet-to-dry transition to a paste. The Eppendorf was placed in a thermoshaker at 50 °C and 600 r.p.m. for up to 168 h. The reaction was followed by periodically removing samples (~10 mg), dissolving them in 0.5 mL of 0.5 M citric acid buffer at pH 6.85 and 9:1 H_2_O:D_2_O, and analysing them with ^31^P-NMR and ^1^H-NMR, and ^1^H ^31^P HMBC (Heteronuclear Multiple Bond Correlation) NMR spectroscopy. The citric acid buffer chelated calcium ions enabling full solubilization of all calcium phosphate salts. Reactions with other nucleophiles were conducted using an identical procedure unless otherwise specified in the SI Section S7.

### Physicochemical orthophosphate cycle with three transverses of the cycle

A 450 mM potassium cyanate and a 150 mM sodium phosphate dibasic solution was prepared by dissolving 730.1 mg (9.0 mmol, 3 eq.) of potassium cyanate and 534.0 mg (3.0 mmol, 1 eq.) of sodium phosphate dibasic dihydrate salt in 20.0 mL of 1.0 M imidazole solution (20 mmol, 6.67 eq.) in 9:1 H_2_O:D_2_O. Then, 276.3 mg (3.0 mmol) of glycerol was dissolved in 4.0 mL of this 450 mM potassium cyanate, 150 mM sodium phosphate dibasic and 1.0 M imidazole solution. The pH of the solution was measured and was typically in the region of pH 8.5–10.0. The pH of the solution was adjusted to pH 7.3 using 5 M hydrochloric acid and 5 M sodium hydroxide solutions. The solution was left to react for 24 h, in order to accumulate imidazole phosphate, and 0.5 mL of the solution was placed in an NMR tube and the reaction was followed by ^31^P-NMR and ^1^H-NMR spectroscopy.

After 24 h, the solution in the NMR tube was recombined with the rest of the solution and the solution was placed into a petri dish and left with the lid off to dry at 22 °C for 24 h. The resulting paste was scraped from the petri dish and placed into an Eppendorf (an NMR sample of the paste was also taken). The Eppendorf was spun on a centrifuge for 3 min at 5590 × *g* in order to move all the paste to the bottom of the Eppendorf and compact it together in order to remove air pockets introduced during the transfer of the paste to the Eppendorf. The Eppendorf was placed on a thermoshaker at 50 °C and 600 r.p.m. for 24 h. The reaction was followed by periodically removing samples (~10 mg), dissolving them in 0.5 mL of 0.5 M citric acid buffer at pH 6.85 and 9 : 1 H_2_O : D_2_O and analysing them with ^31^P-NMR and ^1^H-NMR, and ^1^H ^31^P HMBC NMR spectroscopy. After 24 h, the reaction was removed from the thermoshaker and allowed to cool to room temperature.

To complete the first cycle and start the second cycle, the paste was redissolved in 4.0 mL of 450 mM potassium cyanate solution (prepared from 730.1 mg (9.0 mmol) of potassium cyanate in 20.0 mL of 9:1 H_2_O:D_2_O). The pH of the solution was adjusted to pH 7.3. For the second cycle, the same steps of leaving the solution to react for 24 h, drying to a paste for 24 h and heating to 50 °C for 24 h, as detailed above, were followed. After the second cycle was completed, a third cycle was performed using an identical procedure. Reactions with other nucleophiles were conducted using an identical procedure unless otherwise specified in the Supporting Information. For physicochemical orthophosphate cycles performed with different concentrations of reagents (in Fig. 3), similar procedures were used (see SI Sections S7.11 and S7.12 for details).

Further details of all experimental methods are included in the Supporting Information.

## Supplementary information

Supplementary Information

## Data Availability

The authors declare that all data supporting the findings of this study are available within the paper and Supplementary Information.
