# Supplementary material for: A physicochemical orthophosphate cycle via a kinetically stable thermodynamically activated intermediate enables mild prebiotic phosphorylations
Source: Nat Commun. 2021 Sep 17;12:5517. doi: 10.1038/s41467-021-25555-x (PMC8448844; doi:10.1038/s41467-021-25555-x)
Supplement: Supplementary file 1 — Supplementary Information [file 41467_2021_25555_MOESM1_ESM.pdf]

Supplementary Information

A physicochemical orthophosphate cycle via a kinetically stable thermodynamically activated intermediate enables mild prebiotic phosphorylations

Oliver R. Maguire, Iris B. A. Smokers, Wilhelm T. S. Huck*

Institute for Molecules and Materials, Radboud University Nijmegen, Heyendaalseweg 135, 6525 AJ Nijmegen, The Netherlands.

*Correspondence to: w.huck@science.ru.nl.

Table of Contents

S1 General Procedures 7

S1.1 Materials 7

S1.2 Instrumentation and Software 8

S1.3 Yield determination using ^{31}P NMR spectroscopy 8

S1.3.1 A note on yield determination using ^{31}P NMR spectra 8

S1.3.2 Measurement of T1 relaxation times for quantitative ^{31}P NMR experiments 9

S1.3.3 Solubilising calcium phosphate salts with a chelating buffer 10

S2 Formation of imidazole phosphate in aqueous solution (Main Text Figure 1) 12

S2.1 Preparation of stock solutions 12

S2.2 Formation of imidazole phosphate from cyanate and phosphate in imidazole buffer 12

S2.2.1 Supplementary experimental method with internal standard and Results 12

S2.2.2 Supplementary experimental method for formation of imidazole phosphate in the presence of Mg^{2+} and Zn^{2+} ions and Results 24

S2.2.3 Control experiment in the absence of potassium cyanate 26

S2.3 Formation of imidazole phosphate from carbamoyl phosphate in imidazole buffer 27

S2.3.1 Supplementary experimental method 27

S2.3.2 Formation of imidazole phosphate from carbamoyl phosphate in imidazole buffer with no addition of cyanate 31

S2.3.3 Experimental Method for formation of imidazole phosphate in the presence of Mg^{2+} and Zn^{2+} ions and Results 33

S2.4 Formation of activated phosphoramidate from carbamoyl phosphate with imidazole derivatives and pyrazole 34

S2.4.1 Supplementary experimental method 34

S2.4.2 Orthophosphate activation with 1-methylimidazole 35

S2.4.3 Orthophosphate activation with 2-aminoimidazole 37

S2.4.4 Orthophosphate activation with pyrazole 39

S3 Synthetic formation of calcium imidazole phosphate 41

S3.1 Synthesis of calcium diphosphoimidazole 41

S3.2 Synthesis of calcium imidazole phosphate 43

S4 Reactions of imidazole phosphate in aqueous solution 46

S4.1 Summary of reactions for which no phosphorylation of nucleophile was observed in solution 46

S4.2 Reaction with N-methyl hydroxylamine 47

S4.3 Reaction with N-iso-propyl hydroxylamine 48

S5 Phosphorylation of prebiotically important organic compounds using imidazole phosphate in a paste 50

S5.1 Supplementary experimental method for phosphorylating in a paste 50
S5.2 Phosphorylation of Amphiphiles 50
S5.2.1 Phosphorylation of glycerol 50
S5.2.2 Phosphorylation of Pentane-1,2-diol 52
S5.3 Phosphorylation of Amino Acids 53
S5.3.1 Phosphorylation of serine methyl ester 53
S5.4 Phosphorylation of Metabolites 55
S5.4.1 Phosphorylation of sodium glycolate 55
S5.4.2 Phosphorylation of sodium lactate 56
S5.4.3 Phosphorylation of calcium glycerate 58
S5.5 Phosphorylation of Nucleotides 59
S5.5.1 Phosphorylation of adenosine 5′-monophosphate 59
S5.5.2 Phosphorylation of cytidine 5′-monophosphate 61
S5.5.3 Phosphorylation of guanosine-5′-monophosphate 62
S5.5.4 Phosphorylation of adenosine-5′-diphosphate 64
S5.6 Phosphorylation of Sugars 65
S5.6.1 Phosphorylation of ribose 65
S5.6.2 Phosphorylation of glucose 67

S6 Wet-to-dry transition prior to phosphorylation of prebiotically important organic compounds (Main Text Table 1) 69

S6.1 Supplementary experimental method for wet/dry cycle followed by phosphorylating in a paste 69
S6.2 Phosphorylation of Amphiphiles 69
S6.2.1 Phosphorylation of glycerol 69
S6.3 Phosphorylation of Amino acids + Peptides 73
S6.3.1 Phosphorylation of serine methyl ester 73
S6.3.2 Phosphorylation of serine 76
S6.3.3 Phosphorylation of threonine methyl ester 79
S6.3.4 Phosphorylation of Ser-His 82
S6.4 Phosphorylation of Metabolites 84
S6.4.1 Phosphorylation of sodium glycolate 84
S6.4.2 Phosphorylation of sodium lactate 87
S6.4.3 Phosphorylation of calcium glycerate 90
S6.4.4 Phosphorylation of sodium glycerate 93
S6.4.5 Phosphorylation of lactamide 96
S6.5 Phosphorylation of Nucleosides 99
S6.5.1 Phosphorylation of adenosine 99
S6.5.2 Phosphorylation of cytidine 101
S6.5.3 Phosphorylation of guanosine 103

S6.5.4 Phosphorylation of uridine 105

S6.6 Phosphorylation of Nucleotides 107

S6.6.1 Phosphorylation of adenosine-5′-monophosphate 107

S6.6.1.1 Control reaction for the phosphorylation of adenosine-5′-monophosphate 109

S6.6.2 Phosphorylation of adenosine-5′-diphosphate 110

S6.6.2.1 Control reaction for the phosphorylation of adenosine-5′-diphosphate 112

S6.6.3 Phosphorylation of cytidine-5′-monophosphate 114

S6.6.3.1 Control reaction for the phosphorylation of cytidine-5′-monophosphate 116

S6.6.4 Phosphorylation of cytidine-5′-diphosphate 117

S6.6.4.1 Control reaction for the phosphorylation of cytidine-5′-diphosphate 119

S6.6.5 Phosphorylation of guanosine-5′-monophosphate 120

S6.6.5.1 Control reaction for the phosphorylation of guanosine-5′-monophosphate 122

S6.6.6 Phosphorylation of guanosine-5′-diphosphate 123

S6.6.6.1 Control reaction for the phosphorylation of guanosine-5′-diphosphate 125

S6.6.7 Phosphorylation of uridine-5′-monophosphate 126

S6.6.7.1 Control reaction for the phosphorylation of uridine-5′-monophosphate 128

S6.6.8 Phosphorylation of uridine-5′-diphosphate 129

S6.6.8.1 Control reaction for the phosphorylation of uridine-5′-diphosphate 131

S6.7 Phosphorylation of Sugars 132

S6.7.1 Phosphorylation of ribose 132

S6.7.2 Phosphorylation of glucose 135

S6.7.3 Phosphorylation of glucose 138

S6.8 Results of all paste reactions and a comparison between the two methods used to perform the paste reaction. 140

S7 Demonstration of physicochemical orthophosphate cycle which recycles orthophosphate in a wet/dry cycle (Main Text Figure 3) 143

S7.1 Supplementary experimental Method for orthophosphate recycling with a wet/dry cycle 143

S7.2 Phosphorylation of glycerol with orthophosphate recycling 144

S7.2.1 Phosphorylation of glycerol with 150 mM orthophosphate 144

S7.2.2 Phosphorylation of glycerol with 150 mM orthophosphate repeat 1 148

S7.2.3 Phosphorylation of glycerol with 150 mM orthophosphate repeat 2 150

S7.2.4 Phosphorylation of glycerol with 50 mM orthophosphate 153

S7.2.5 Phosphorylation of glycerol with 50 mM orthophosphate repeat 1 157

S7.2.6 Phosphorylation of glycerol with 50 mM orthophosphate repeat 2 159

S7.2.7 Phosphorylation of glycerol with 20 mM orthophosphate 162

S7.2.8 Phosphorylation of glycerol with 20 mM orthophosphate repeat 1 166

S7.2.9 Phosphorylation of glycerol with 20 mM orthophosphate repeat 2 168

S7.3 Phosphorylation of glycerate with orthophosphate recycling 171

S7.3.1 Phosphorylation of glycerate with 150 mM orthophosphate 171

S7.3.2 Phosphorylation of glycerate with 150 mM orthophosphate repeat 1 175

S7.3.3 Phosphorylation of glycerate with 150 mM orthophosphate repeat 2 177

S7.3.4 Phosphorylation of glycerate with 50 mM orthophosphate 180

S7.3.5 Phosphorylation of glycerate with 50 mM orthophosphate repeat 1 184

S7.3.6 Phosphorylation of glycerate with 50 mM orthophosphate repeat 2 186

S7.3.7 Phosphorylation of glycerate with 20 mM orthophosphate 189

S7.3.8 Phosphorylation of glycerate with 20 mM orthophosphate repeat 1 193

S7.3.9 Phosphorylation of glycerate with 20 mM orthophosphate repeat 2 195

S7.4 Phosphorylation of adenosine-5’-monophosphate with orthophosphate recycling 198

S7.4.1 Phosphorylation of adenosine-5’-monophosphate with 150 mM orthophosphate 198

S7.4.2 Phosphorylation of adenosine-5’-monophosphate with 150 mM orthophosphate repeat 1 202

S7.4.3 Phosphorylation of adenosine-5’-monophosphate with 150 mM orthophosphate repeat 2 204

S7.4.4 Phosphorylation of adenosine-5’-monophosphate with 50 mM orthophosphate 207

S7.4.5 Phosphorylation of adenosine-5’-monophosphate with 50 mM orthophosphate repeat 1 211

S7.4.6 Phosphorylation of adenosine-5’-monophosphate with 50 mM orthophosphate repeat 2 213

S7.4.7 Phosphorylation of adenosine-5’-monophosphate with 20 mM orthophosphate 216

S7.4.8 Phosphorylation of adenosine-5’-monophosphate with 20 mM orthophosphate repeat 1 220

S7.4.9 Phosphorylation of adenosine-5’-monophosphate with 20 mM orthophosphate repeat 2 222

S7.5 Phosphorylation of serine with orthophosphate recycling 225

S7.5.1 Phosphorylation of serine with 50 mM orthophosphate 225

S7.5.2 Phosphorylation of serine with 20 mM orthophosphate 229

S7.6 Phosphorylation of D-ribose with orthophosphate recycling 232

S7.6.1 Phosphorylation of D-ribose with 50 mM orthophosphate 232

S7.6.2 Phosphorylation of D-ribose with 20 mM orthophosphate 235

S7.7 Phosphorylation of D-glucose with orthophosphate recycling 238

S7.7.1 Phosphorylation of D-glucose with 50 mM orthophosphate 238

S7.7.2 Phosphorylation of D-glucose with 20 mM orthophosphate 241

S7.8 Phosphorylation of adenosine with orthophosphate recycling 244

S7.8.1 Phosphorylation of adenosine with 50 mM orthophosphate 244

S7.8.2 Phosphorylation of adenosine with 20 mM orthophosphate 247

S7.9 Phosphorylation of cytidine with orthophosphate recycling 250

S7.9.1 Phosphorylation of cytidine with 50 mM orthophosphate 250

S7.9.2 Phosphorylation of cytidine with 20 mM orthophosphate 253

7.10 Control reactions 256

S7.10.1 Supplementary experimental method for control reactions in the absence of imidazole 256

S7.10.2 Control reaction in the absence of imidazole with glycerol 257

S7.10.3 Control reaction in the absence of imidazole with ribose 258

S7.10.4 Control reaction in the absence of imidazole with calcium glycerate 259

S7.10.5 Control reaction in the absence of imidazole with serine methyl ester 260

S7.10.6 Control reaction in the absence of imidazole with adenosine-5′-monophosphate 261

S7.11 Orthophosphate recycling with a wet/dry cycle with 100 mM reagent concentrations 262

S7.11.1 Supplementary experimental method for orthophosphate recycling with a wet/dry cycle with 100 mM reagent concentrations 262

S7.11.2 Phosphorylation of glycerol with 20 mM orthophosphate recycling and 100 mM reagent concentrations 263

S7.11.3 Phosphorylation of glycerol with 50 mM orthophosphate and 100 mM reagent concentrations 265

S7.11.4 Phosphorylation of glycerate with 20 mM orthophosphate and 100 mM reagent concentrations 267

S7.11.5 Phosphorylation of glycerate with 50 mM orthophosphate and 100 mM reagent concentrations 269

S7.11.6 Phosphorylation of adenosine-5′-monophosphate with 20 mM orthophosphate and 100 mM reagent concentrations 271

S7.11.7 Phosphorylation of adenosine-5′-monophosphate with 50 mM orthophosphate and 100 mM reagent concentrations 273

S7.11.8 Phosphorylation of serine with 20 mM orthophosphate and 100 mM reagent concentrations 275

S7.11.9 Phosphorylation of serine with 50 mM orthophosphate and 100 mM reagent concentrations 277

S7.11.10 Phosphorylation of cytidine with 20 mM orthophosphate and 100 mM reagent concentrations 279

S7.11.11 Phosphorylation of cytidine with 50 mM orthophosphate and 100 mM reagent concentrations 281

S7.12 Phosphorylation of glycerol with orthophosphate recycling at reagent concentrations of 10 – 100 mM 283

S7.12.1 Supplementary Experimental method for orthophosphate recycling with a wet/dry cycle at reagent concentrations of 10 – 100 mM 283

S7.12.2 Phosphorylation of glycerol with orthophosphate recycling at [KOCN] = 100 mM, [Na_2HPO_4] = 50 mM, [imidazole] = 50 mM and [glycerol] = 100 mM 283

S7.12.3 Phosphorylation of glycerol with orthophosphate recycling at [KOCN] = 50 mM, [Na_2HPO_4] = 50 mM, [imidazole] = 50 mM and [glycerol] = 50 mM 286

S7.12.4 Phosphorylation of glycerol with orthophosphate recycling at [KOCN] = 20 mM, [Na_2HPO_4] = 20 mM, [imidazole] = 20 mM and [glycerol] = 20 mM 288

S7.12.4 Phosphorylation of glycerol with orthophosphate recycling at [KOCN] = 20 mM, [Na_2HPO_4] = 10 mM, [imidazole] = 10 mM and [glycerol] = 20 mM 290

S8 Synthesis of compounds for spiking 292

S8.1 Synthesis of phosphoserine methyl ester 292

S9 Supplementary References 295

S1 General Procedures

S1.1 Materials

All chemicals and reagents were used as received from commercial suppliers. We used MilliQ (MQ) water (i.e., ultrapure deionized water) from Millipore Corporation.

The following compounds were purchased from Sigma Aldrich: deuterium oxide, imidazole, lithium carbamoyl phosphate dibasic hydrate, zinc chloride, magnesium chloride, hexamethylphosphoramide, phosphor(V)oxychloride, L-serine, L-histidine monochloride monohydrate, serine methyl ester hydrochloric acid salt, D-ribose, D-ribose 5-phosphate disodium salt hydrate, glycerol, 1,2-pentanediol, 1,2-hexanediol, glycolic acid, sodium lactate, (S)-lactamide, calcium DL-glycerate dihydrate, 2-phosphoglycolic acid lithium salt, 2,3-diphospho-D-glyceric acid pentasodium salt, D(+)2-phosphoglyceric acid sodium salt, L-2-phosphoglyceric acid disodium salt, rac-glycerol 1-phosphate sodium salt hydrate, glycerol-2-phosphate sodium salt, D(-)3-phosphoglyceric acid disodium salt, adenosine, adenosine 5'-monophosphate disodium salt, adenosine 5'-diphosphate sodium salt hydrate, adenosine 5'-triphosphate disodium salt hydrate, cytidine, cytidine 5'-triphosphate disodium salt, guanosine, guanosine monophosphate disodium salt hydrate, guanosine 5'-diphosphate disodium salt, uridine 5'-diphosphate disodium salt hydrate, Bio-Rad Chelex 100 resin sodium form 50-100 mesh (dry), calcium chloride.

The following compounds were purchased from Fisher Scientific: sodium phosphate monobasic anhydrous, D-glucose, serine methyl ester hydrochloric acid salt, volumetric 1.0 M hydrochloric acid, volumetric 2.0 M hydrochloric acid, volumetric 1.0 M sodium hydroxide, volumetric 2.0 M sodium hydroxide, Thermo Scientific Orion Standard All-in-One pH buffer Kit.

The following compounds were purchased from TCI Europe: N-methyl hydroxylamine, adenosine 5'-diphosphate disodium salt, cytidine 5'-monophosphate disodium salt, cytidine 5'-diphosphate trisodium salt, guanosine 5'-monophosphate disodium salt, uridine 5'-monophosphate disodium salt, DL-glyceric acid, calcium glycerate.

The following compounds were purchased from Carbosynth: cytidine-2'-monophosphate sodium salt, cytidine 3',5'-cyclic monophosphate monosodium salt, cytidine 3'(2')-monophosphate, cytidine 2',3'-cyclic monophosphate monosodium salt, uridine-3',5'-cyclic monophosphate sodium salt, uridine 3'-monophosphate disodium salt.

The following compounds were purchased from Merck: potassium cyanate.

The following compounds were purchased from Acros Organics: N-isopropyl hydroxylamine.

The following compounds were purchased from Bachem: Ac-Ser-OMe, Ac-Thr-OMe, Ser-His.

The following compounds were purchased from Fluorochem: citric acid.

The following compounds were purchased from Janssen: methyl-(S) lactate.

S1.2 Instrumentation and Software

Nuclear Magnetic Resonance (NMR) spectra for ^{1}H, ^{31}P and ^{13}C nuclei were measured on a *Bruker-AVANCE III 500* spectrometer at 500 MHz or on a *Bruker-AVANCE III 400* spectrometer at 400 MHz. The chemical shifts for ^{1}H and given in parts per million (ppm) and calibrated using a residual solvent peak of 4.79 for D_2O in ^{1}H NMR. ^{31}P Multiplets are reported as s (singlet), d (doublet), dd (doublet of doublet), t (triplet), q (quartet) and m (multiplet). Coupling constants, *J*, are reported in Hertz (Hz). The number of protons (n) for a given resonance is indicated as nH and is based on the spectral integration values.

Quantitative ^{31}P NMR spectroscopy was performed for all ^{31}P NMR spectra. A pulse sequence was set up with 8 transients (nt = 8), a P1 = 13 ms which corresponds to approximately a 90° pulse angle and a d_1 relaxation delay of 30 s, in order to ensure full relaxation of nuclei between each transient. A solvent suppression sequence was used for the measurement of ^{1}H NMR spectra when a 9 : 1 H_2O : D_2O solvent was used.

The pH of solutions was determined using a Mettler Toledo Five Easy FE20 pH meter and a Hamilton SpinTrode pH probe. The pH meter calibrated with Thermo Scientific Orion Standard buffers at pH 4.01, pH 7.00, pH 10.00.

All data was processed in MestReNova 14, and OriginPro 2018b.

S1.3 Yield determination using ^{31}P NMR spectroscopy

S1.3.1 A note on yield determination using ^{31}P NMR spectra

The yields reported in the paper and in the SI were determined by integrating all the peaks in the ^{31}P NMR spectra, summating all of these integrals and calculating the yield based upon this total summated integral. In Figure 1 and in (most cases for) Figure 3, the only phosphate containing species present at the start of the reaction was orthophosphate. For Figure 2 and Table 1, in most cases, the only phosphate containing species present at the start of the reaction was imidazole phosphate. Therefore, by summating the integrals of all phosphate containing species we obtained the total starting concentration of orthophosphate or imidazole phosphate, respectively. Yields were determined relative to the summated integral for all phosphorylated species.

The exception for Figure 3 was for those experiments in which AMP was phosphorylated to form ADP. In these cases, only those phosphate signals in the ^{31}P NMR spectra which arose from orthophosphate were

included to calculate the summated integral (e.g. the signal from beta-phosphate in ADP was included but not the alpha-phosphate). The exception for Figure 2 and Table 1, was for those reactions with NMPs and NDPs. In these cases, only those phosphate signals in the ^{31}P NMR spectra which arose from imidazole phosphate were included in the summated integral.

S1.3.2 Measurement of T1 relaxation times for quantitative ^{31}P NMR experiments

We measured the 90° pulse angle and measured the T1 relaxation times for the ^{31}P nuclei in a series of important phosphate containing compounds used in our study (Supplementary Table 0.1). The longest T1 relaxation time measured was 3.2 s for orthophosphate. To be quantitative an NMR experiment with a 90° pulse requires a delay of 5*T1. This delay ensures full relaxation of the nuclei back into orientation of the applied magnetic field. For our ^{31}P-NMR experiments with orthophosphate T1 = 3.2 s, this means that a minimum delay of 16.0 s is required in order for quantitative ^{31}P-NMR experiments. We used a d1 delay of 30.0 s and along with a 1.5 – 2.0 s acquisition time, this means that a total of 31.5 – 32.0 s was given for the nuclei to relax. Thus is approximately double the minimum delay required and therefore our ^{31}P-NMR experiments are quantitative.

Supplementary Table 0.1: Measured 90° pulse lengths (P90) and T1 relaxation times for important phosphate containing compounds used in our study. The P90 and T1 were determined in a 100 mM solution of the compound in 0.5 M citric acid buffer pH 6.85 at 22.0 °C. This citric acid buffer was typically used in our study to analyse the pastes.

Compound	P90 (μs)	T1 (s)
Orthophosphate	13.300	3.20
Carbamoyl phosphate	13.375	2.15
Imidazole phosphate (Calcium salt)	13.350	1.40
Glycerol-1-phosphate	13.688	2.23
Glycerol-2-phosphate	13.750	2.20
O-phosphorylated serine	13.475	1.77
3-phosphoglycerate	13.500	1.97
AMP	13.350	0.97
ADP α	13.312	0.42
ADP β	13.312	0.70
ATP α	13.250	0.30
ATP β	13.250	0.33
ATP γ	13.250	0.49
Ribose-5-phosphate	13.500	1.53
Glucose-6-phosphate	13.500	1.20

S1.3.3 Solubilising calcium phosphate salts with a chelating buffer

In the paste reactions shown in Figure 2 and Table 1 calcium phosphate, calcium imidazole phosphate and calcium phosphate monoesters are present in the reaction. It is well known that calcium phosphate and related species readily precipitate in water and if such precipitates were present during the analysis by NMR spectroscopy this would lead to inaccurate results.

However, in our experiments we avoided this problem of precipitation of calcium phosphate salts by using a citric acid buffer. We performed the analysis of pastes in a 0.5 M citric acid buffer at pH 6.85. The citric acid (technically citrate) chelates the Ca^{2+} ions and thus ensured that the pastes were always fully solubilised. Thus, we are confident that all the phosphorus signals were present in NMR spectra and therefore the yields we have reported are accurate.

To determine the solubility limit of calcium phosphate in 0.5 M citric acid buffer we prepared equimolar solutions of calcium chloride and sodium phosphate of between 10 mM – 200 mM. Stock solutions of 2 M calcium chloride (222 mg, 2 mmol in 1 mL of water) (warning – add water dropwise to solid calcium chloride in an ice-bath as the dissolution is highly exothermic) and 2 M sodium phosphate monobasic (240 mg, 2 mmol in 1 mL of water) were prepared. Appropriate volumes of these stock solutions were added to 1 mL of 0.5 M citric acid buffer in order to prepare solution of 10 – 83 mM of both calcium chloride and sodium phosphate. Solutions of 100 mM and 200 mM were prepared by dissolving calcium chloride (for 100 mM 11.1 mg, for 200 mM 22.2 mg) and sodium phosphate monobasic (for 100 mM 12.0 mg, for 200 mM 24.0 mg) in 0.5 M citric acid buffer pH 6.9. Solutions were left for 24 h to allow time for precipitate to form prior to being checked. Supplementary Figure 0.1 shows that calcium phosphate precipitated at concentrations above 83 mM and that therefore the solubility limit of calcium phosphate in these circumstances was between 74 – 83 mM.

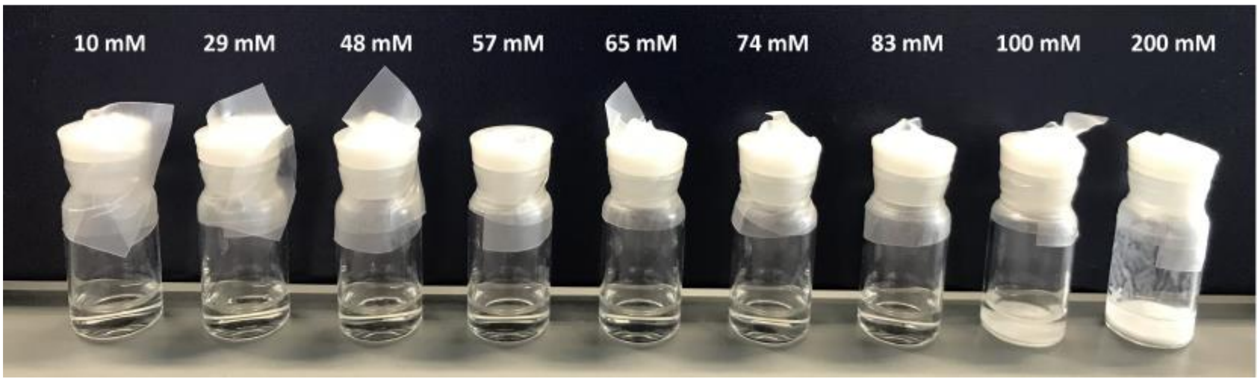

Supplementary Figure 0.1: Vials containing solutions with equimolar concentrations of calcium chloride and sodium phosphate between 10 mM – 200 mM in 0.5 M citric acid buffer pH ~ 6.5. Calcium phosphate can be seen as a white precipitate in solutions of 83 mM and above. Note that only a minor amount of precipitate was observed at 83 mM and therefore it is not visible in the photo. Solutions were left for 24 h prior to photo being taken to give time for precipitate to form.

Note that while calcium phosphate ($Ca_3(PO_4)_2$) has calcium ions : phosphate ions in a 3 : 2 ratio we prepared solutions in a 1 : 1 ratio. We did this because whenever calcium ions were added to our reactions it was in the form of calcium imidazole phosphate where the ratio of calcium ions : phosphate ions is 1 : 1. Therefore to obtain results that are most relevant to our work we also prepared solutions with equimolar (1 : 1) concentrations of calcium chloride and sodium phosphate monobasic in 0.5 M citric acid buffer.

For the experiments in Figure 2 and Table 1 where we followed the reaction between calcium imidazole phosphate and various nucleophiles in a paste over time, we analysed the progress of the reaction by removing approximately 10 mg samples from the pastes which were then dissolved in 0.5 mL of 0.5 M citric acid buffer (pH 6.85). Based on the dry mass of reagents added to the paste this would convert to a maximum concentration of ~30 mM of calcium ions and phosphate ions. In reality, the concentrations are likely to be lower as the pastes were still wet. Given that the solubility limit of the calcium phosphate is between 74 – 83 mM, the maximum concentration of 30 mM in our experiments is well below the solubility limit and therefore all calcium phosphate was solubilised and therefore the NMR analyses were quantitative.

S2 Formation of imidazole phosphate in aqueous solution (Main Text Figure 1)

S2.1 Preparation of stock solutions

A 1.0 M imidazole buffer was prepared by dissolving 680 mg (10.0 mmol) imidazole in 10 mL of 9 : 1 H_2O : D_2O and volumetric 1.0 M hydrochloric acid was used to adjust the pH of the buffer to 6.42.

A 1.0 M imidazole buffer with 50 mM HMPA was prepared by dissolving 340 mg (5 mmol) imidazole in 4.75 mL of 9 : 1 H_2O : D_2O, adding 250 µl 1 M HMPA in D_2O and volumetric 1.0 M hydrochloric acid was used to adjust the pH of the buffer to 6.37. 1.0 M imidazole solutions at pH 6.52, 6.73, 7.00 and 7.32 were made using the same procedure.

A 2.0 M potassium cyanate solution was prepared by dissolving 81 mg (1.0 mmol) potassium cyanate in 0.5 mL of 1.0 M imidazole buffer at pH 6.4.

S2.2 Formation of imidazole phosphate from cyanate and phosphate in imidazole buffer

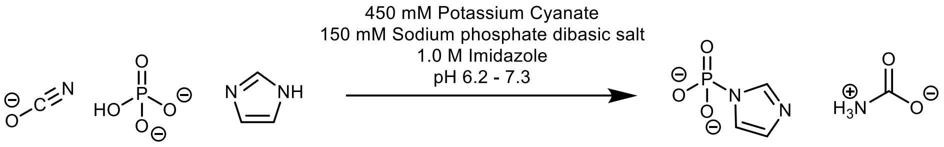

S2.2.1 Supplementary experimental method with internal standard and Results

A 450 mM potassium cyanate and 150 mM sodium phosphate solution was prepared by dissolving 36.5 mg (0.45 mmol, 3 eq) potassium cyanate and 26.7 mg (0.15 mmol, 1 eq) sodium phosphate monobasic anhydrous in 1.0 mL 1.0 M imidazole buffer at pH 6.17 (1.00 mmol, 6.67 eq) containing 50 mM HMPA internal standard. The pH of the solution at the start was pH 6.37. The reaction was followed by ^{31}P-NMR and ^{1}H-NMR spectroscopy, measuring spectra at a series of time points over the course of 16 days. The pH of the sample was measured at time points over the course of the reaction. An identical procedure was followed for the reactions with starting pHs of 6.52, 6.73, 7.00 and 7.32. The experiments were repeated in triplicate.

Supplementary Figures 1 – 5 show the formation of imidazole phosphate at a series of different starting pHs. Supplementary Tables 0.2 – 0.6 show the average yields + standard deviation of orthophosphate, carbamoyl phosphate, imidazole phosphate and pyrophosphate from the triplicate experiments. The changes in yield over time for orthophosphate, carbamoyl phosphate and imidazole phosphate are shown in Supplementary Figure 6.

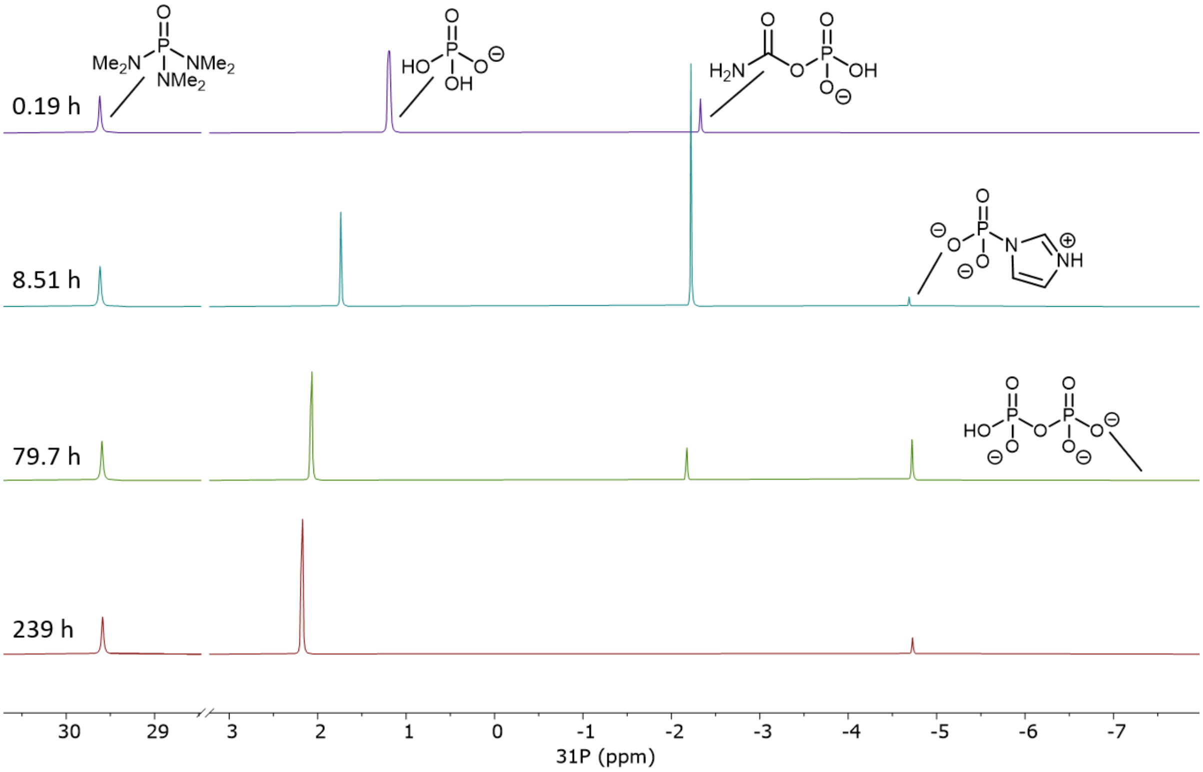

Supplementary Figure 1: Representative ^{31}P-NMR spectra over time for 450 mM potassium cyanate with 150 mM sodium phosphate and 50 mM HMPA internal standard in 1.0 M imidazole buffer. The solution was pH 6.37 at the start and this rose to pH 7.42 at the end of the reaction. The depicted speciation states are for pH 6.37. Changes in chemical shift of peaks are due to an increase in pH over time.

Supplementary Table 0.2: Average changes in percentage yield over time for each phosphorus species in the ^{31}P-NMR spectra (representative Supplementary Figure 6.1) for 450 mM potassium cyanate with 150 mM sodium phosphate and 50 mM HMPA internal standard in 1.0 M imidazole buffer pH 6.37-7.42 performed in triplicate. Standard deviation provided for each value was based on three repeats.

Time (h)	Phosphate (%)	Carbamoyl phosphate (%)	Imidazole phosphate (%)	Pyrophosphate (%)
0.19	86.83 ± 1.13	12.81 ± 1.14	0.00 ± 0.00	0.00 ± 0.00
2.78	44.39 ± 0.53	54.47 ± 0.44	1.00 ± 0.11	0.00 ± 0.00
5.14	37.01 ± 0.12	61.05 ± 0.10	1.85 ± 0.03	0.00 ± 0.00
8.51	34.83 ± 0.41	61.61 ± 0.43	3.49 ± 0.13	0.00 ± 0.00
13.23	36.22 ± 0.32	58.17 ± 0.46	5.52 ± 0.09	0.00 ± 0.00
18.12	38.86 ± 0.37	53.60 ± 0.42	7.54 ± 0.10	0.00 ± 0.00
23.26	41.80 ± 0.32	48.90 ± 0.46	9.30 ± 0.19	0.00 ± 0.00
31.98	46.79 ± 0.19	40.97 ± 0.22	12.24 ± 0.10	0.00 ± 0.00
47.27	53.88 ± 0.17	30.65 ± 0.07	15.44 ± 0.18	0.03 ± 0.03
56.88	58.30 ± 0.60	24.86 ± 0.21	16.78 ± 0.46	0.07 ± 0.04
71.26	63.78 ± 0.45	18.43 ± 0.11	17.70 ± 0.35	0.09 ± 0.03
79.72	66.82 ± 0.98	15.25 ± 0.35	17.84 ± 0.61	0.08 ± 0.02
95.31	71.34 ± 0.99	10.97 ± 0.27	17.60 ± 0.75	0.10 ± 0.04
103.79	73.86 ± 1.18	8.87 ± 0.33	17.16 ± 0.87	0.10 ± 0.03
135.32	81.05 ± 1.48	4.29 ± 0.26	14.51 ± 1.23	0.15 ± 0.05
167.52	86.38 ± 1.30	1.95 ± 0.29	11.45 ± 1.02	0.22 ± 0.01
191.39	89.12 ± 1.43	1.09 ± 0.15	9.53 ± 1.34	0.27 ± 0.03
220.51	91.51 ± 1.54	0.60 ± 0.16	7.64 ± 1.40	0.26 ± 0.03
239.29	93.06 ± 1.32	0.31 ± 0.05	6.38 ± 1.25	0.25 ± 0.02
267.02	94.53 ± 1.26	0.17 ± 0.07	5.04 ± 1.20	0.26 ± 0.03
335.33	96.93 ± 0.97	0.00 ± 0.00	2.76 ± 0.95	0.31 ± 0.03
390.45	97.81 ± 0.86	0.00 ± 0.00	1.83 ± 0.84	0.36 ± 0.03

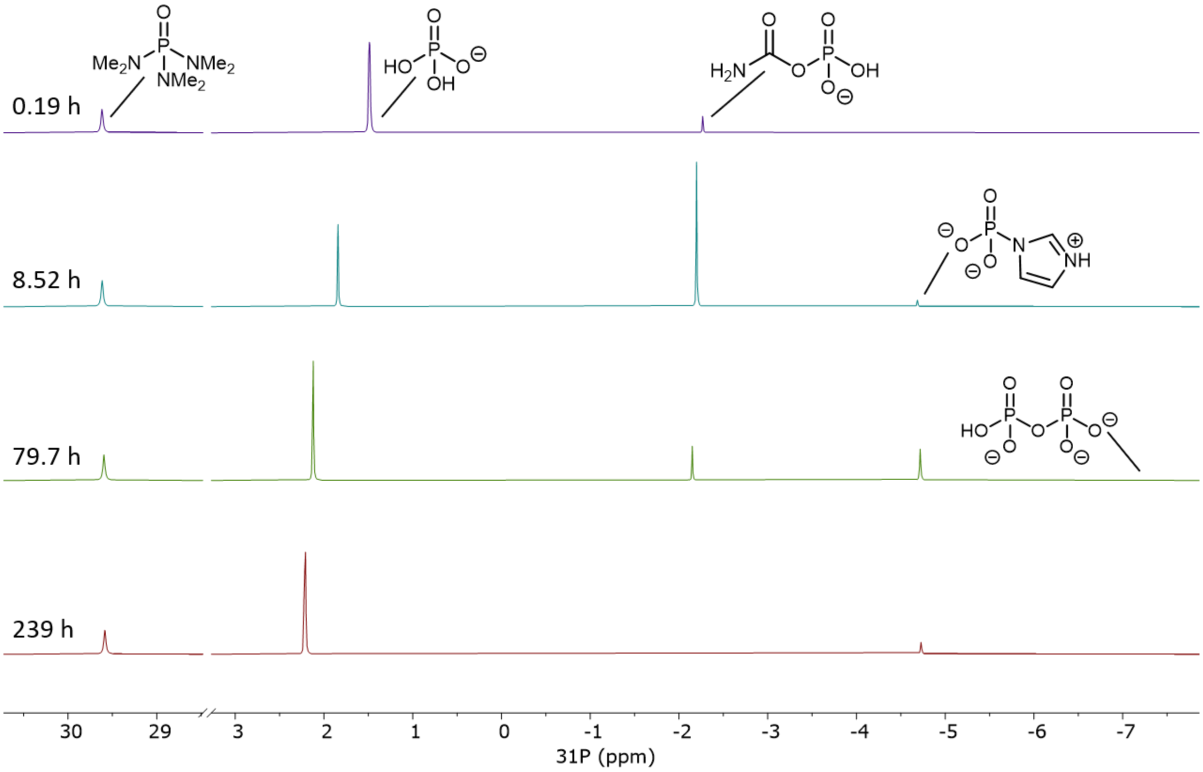

Supplementary Figure 2: Representative ^{31}P-NMR spectra over time for 450 mM potassium cyanate with 150 mM sodium phosphate and 50 mM HMPA internal standard in 1.0 M imidazole buffer. The solution was pH 6.52 at the start and this rose to pH 7.51 at the end of the reaction. The depicted speciation states are for pH 6.52. Changes in chemical shift of peaks are due to an increase in pH over time.

Supplementary Table 0.3: Average changes in percentage yield over time for each phosphorus species in the ^{31}P-NMR spectra (representative Supplementary Figure 6.2) for 450 mM potassium cyanate with 150 mM sodium phosphate and 50 mM HMPA internal standard in 1.0 M imidazole buffer pH 6.52-7.51 performed in triplicate. Standard deviation provided for each value was based on three repeats.

Time (h)	Phosphate (%)	Carbamoyl phosphate (%)	Imidazole phosphate (%)	Pyrophosphate (%)
0.19	91.06 ± 0.35	8.69 ± 0.34	0.00 ± 0.00	0.00 ± 0.00
2.79	52.60 ± 0.24	46.30 ± 0.25	0.94 ± 0.05	0.00 ± 0.00
5.16	43.28 ± 0.28	54.62 ± 0.24	2.04 ± 0.07	0.00 ± 0.00
8.52	39.32 ± 0.23	56.93 ± 0.26	3.67 ± 0.07	0.00 ± 0.00
13.27	39.28 ± 0.10	54.69 ± 0.22	5.97 ± 0.15	0.00 ± 0.00
18.14	40.99 ± 0.15	50.70 ± 0.13	8.32 ± 0.08	0.00 ± 0.00
23.27	43.16 ± 0.13	46.33 ± 0.19	10.48 ± 0.05	0.03 ± 0.03
31.98	47.18 ± 0.25	39.21 ± 0.13	13.55 ± 0.17	0.06 ± 0.04
47.28	53.56 ± 0.08	29.06 ± 0.03	17.33 ± 0.10	0.04 ± 0.01
56.89	57.42 ± 0.36	23.66 ± 0.10	18.87 ± 0.36	0.04 ± 0.02
71.27	62.46 ± 0.62	17.59 ± 0.12	19.88 ± 0.51	0.07 ± 0.01
79.73	65.56 ± 0.54	14.45 ± 0.07	19.90 ± 0.51	0.09 ± 0.04
95.32	70.06 ± 0.85	10.33 ± 0.16	19.50 ± 0.75	0.12 ± 0.05
103.80	72.21 ± 0.97	8.46 ± 0.20	19.19 ± 0.81	0.14 ± 0.03
135.33	79.45 ± 1.53	4.10 ± 0.41	16.29 ± 1.11	0.17 ± 0.02
167.52	85.03 ± 1.33	1.83 ± 0.21	12.95 ± 1.15	0.20 ± 0.03
191.41	87.93 ± 1.43	1.02 ± 0.18	10.83 ± 1.26	0.21 ± 0.03
220.52	90.69 ± 1.51	0.49 ± 0.15	8.56 ± 1.37	0.27 ± 0.01
239.30	91.95 ± 1.39	0.39 ± 0.08	7.39 ± 1.39	0.27 ± 0.05
267.03	93.75 ± 1.42	0.15 ± 0.04	5.83 ± 1.40	0.27 ± 0.03
335.34	96.44 ± 1.22	0.00 ± 0.00	3.24 ± 1.19	0.32 ± 0.03
390.46	97.46 ± 1.09	0.00 ± 0.00	2.19 ± 1.06	0.35 ± 0.03

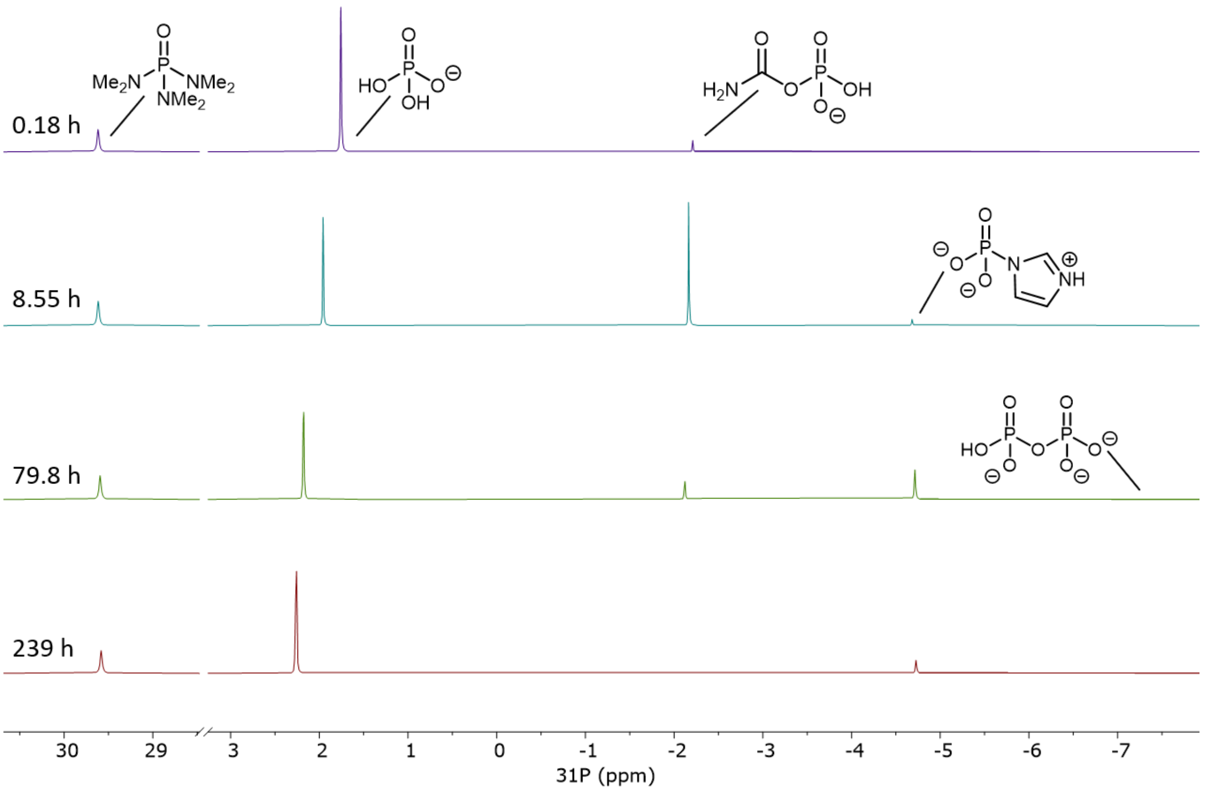

Supplementary Figure 3: Representative ^{31}P-NMR spectra over time for 450 mM potassium cyanate with 150 mM sodium phosphate and 50 mM HMPA internal standard in 1.0 M imidazole buffer. The solution was pH 6.73 at the start and this rose to pH 7.66 at the end of the reaction. The depicted speciation states are for pH 6.73. Changes in chemical shift of peaks are due to an increase in pH over time.

Supplementary Table 0.4: Average changes in percentage yield over time for each phosphorus species in the ^{31}P-NMR spectra (representative Supplementary Figure 6.3) for 450 mM potassium cyanate with 150 mM sodium phosphate and 50 mM HMPA internal standard in 1.0 M imidazole buffer pH 6.73-7.66 performed in triplicate. Standard deviation provided for each value was based on three repeats.

Time (h)	Phosphate (%)	Carbamoyl phosphate (%)	Imidazole phosphate (%)	Pyrophosphate (%)
0.18	94.02 ± 0.04	5.89 ± 0.03	0.00 ± 0.00	0.00 ± 0.00
2.82	61.81 ± 0.23	37.18 ± 0.27	0.88 ± 0.12	0.00 ± 0.00
5.19	51.69 ± 0.07	46.15 ± 0.13	2.09 ± 0.06	0.00 ± 0.00
8.55	46.36 ± 0.12	49.72 ± 0.26	3.87 ± 0.17	0.00 ± 0.00
13.30	44.70 ± 0.18	48.95 ± 0.19	6.35 ± 0.07	0.00 ± 0.00
18.17	45.03 ± 0.17	45.96 ± 0.26	8.98 ± 0.10	0.04 ± 0.02
23.29	46.43 ± 0.12	42.27 ± 0.12	11.28 ± 0.11	0.02 ± 0.01
32.01	49.51 ± 0.09	35.61 ± 0.13	14.86 ± 0.04	0.03 ± 0.01
47.30	54.37 ± 0.06	26.63 ± 0.03	18.94 ± 0.12	0.06 ± 0.03
56.92	57.59 ± 0.23	21.69 ± 0.10	20.67 ± 0.20	0.05 ± 0.01
71.29	62.11 ± 0.58	15.91 ± 0.15	21.88 ± 0.42	0.10 ± 0.03
79.78	64.56 ± 0.90	13.15 ± 0.33	22.18 ± 0.59	0.11 ± 0.01
95.34	68.66 ± 1.05	9.26 ± 0.33	21.97 ± 0.74	0.11 ± 0.03
103.83	70.77 ± 1.25	7.54 ± 0.32	21.57 ± 0.93	0.12 ± 0.01
135.36	77.74 ± 1.42	3.62 ± 0.28	18.48 ± 1.15	0.17 ± 0.04
167.55	83.25 ± 1.51	1.58 ± 0.16	14.96 ± 1.39	0.20 ± 0.04
191.43	86.06 ± 1.57	0.90 ± 0.13	12.83 ± 1.47	0.21 ± 0.03
220.54	89.19 ± 1.46	0.48 ± 0.06	10.08 ± 1.43	0.25 ± 0.02
239.33	90.59 ± 1.72	0.30 ± 0.05	8.87 ± 1.59	0.24 ± 0.09
267.06	92.31 ± 1.64	0.21 ± 0.04	7.25 ± 1.61	0.24 ± 0.03
335.37	95.46 ± 1.39	0.00 ± 0.00	4.23 ± 1.39	0.31 ± 0.01
390.48	96.93 ± 1.28	0.00 ± 0.00	2.75 ± 1.25	0.32 ± 0.04

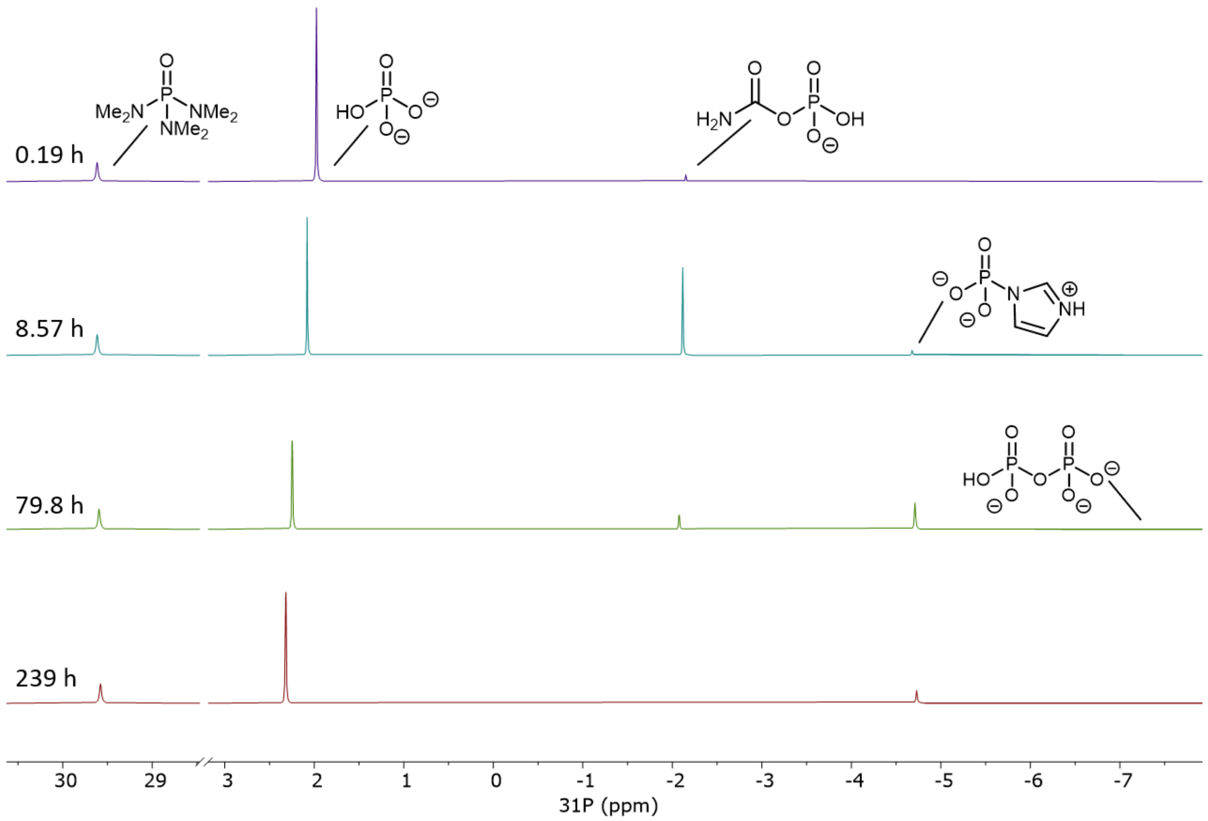

Supplementary Figure 4: Representative ^{31}P-NMR spectra over time for 450 mM potassium cyanate with 150 mM sodium phosphate and 50 mM HMPA internal standard in 1.0 M imidazole buffer. The solution was pH 7.00 at the start and this rose to pH 7.84 at the end of the reaction. The depicted speciation states are for pH 7.00. Changes in chemical shift of peaks are due to an increase in pH over time.

Supplementary Table 0.5: Average changes in percentage yield over time for each phosphorus species in the ^{31}P-NMR spectra (representative Supplementary Figure 6.4) for 450 mM potassium cyanate with 150 mM sodium phosphate and 50 mM HMPA internal standard in 1.0 M imidazole buffer pH 7.00-7.84 performed in triplicate. Standard deviation provided for each value was based on three repeats.

Time (h)	Phosphate (%)	Carbamoyl phosphate (%)	Imidazole phosphate (%)	Pyrophosphate (%)
0.19	96.22 ± 0.05	3.70 ± 0.04	0.00 ± 0.00	0.00 ± 0.00
2.85	72.28 ± 0.27	26.82 ± 0.20	0.83 ± 0.10	0.00 ± 0.00
5.21	62.41 ± 0.03	35.59 ± 0.07	1.95 ± 0.02	0.00 ± 0.00
8.57	55.71 ± 0.08	40.35 ± 0.10	3.89 ± 0.02	0.00 ± 0.00
13.32	52.23 ± 0.13	41.16 ± 0.22	6.61 ± 0.12	0.00 ± 0.00
18.15	52.15 ± 0.82	38.79 ± 0.65	9.06 ± 0.17	0.00 ± 0.00
23.32	51.76 ± 0.13	36.33 ± 0.15	11.90 ± 0.09	0.02 ± 0.01
32.03	53.36 ± 0.09	30.82 ± 0.06	15.79 ± 0.10	0.03 ± 0.02
49.27	56.95 ± 0.12	22.22 ± 0.05	20.78 ± 0.11	0.06 ± 0.02
56.94	59.08 ± 0.36	18.68 ± 0.09	22.19 ± 0.30	0.04 ± 0.04
71.32	62.26 ± 0.33	13.75 ± 0.12	23.93 ± 0.21	0.06 ± 0.01
79.81	64.22 ± 0.35	11.41 ± 0.13	24.30 ± 0.32	0.07 ± 0.04
95.36	67.48 ± 0.68	8.11 ± 0.19	24.29 ± 0.46	0.11 ± 0.03
103.86	69.54 ± 0.81	6.56 ± 0.22	23.79 ± 0.60	0.11 ± 0.02
135.39	75.40 ± 0.80	3.10 ± 0.08	21.33 ± 0.74	0.17 ± 0.02
167.57	80.71 ± 1.08	1.34 ± 0.12	17.77 ± 0.96	0.18 ± 0.02
191.46	83.52 ± 1.13	0.77 ± 0.03	15.50 ± 1.11	0.21 ± 0.04
220.51	86.57 ± 1.29	0.39 ± 0.04	12.83 ± 1.29	0.21 ± 0.04
239.35	88.20 ± 1.27	0.25 ± 0.03	11.34 ± 1.29	0.20 ± 0.02
267.08	90.41 ± 1.22	0.07 ± 0.08	9.30 ± 1.15	0.22 ± 0.03
335.40	93.69 ± 1.33	0.00 ± 0.00	6.03 ± 1.31	0.28 ± 0.03
390.50	95.61 ± 1.19	0.00 ± 0.00	4.11 ± 1.21	0.28 ± 0.02

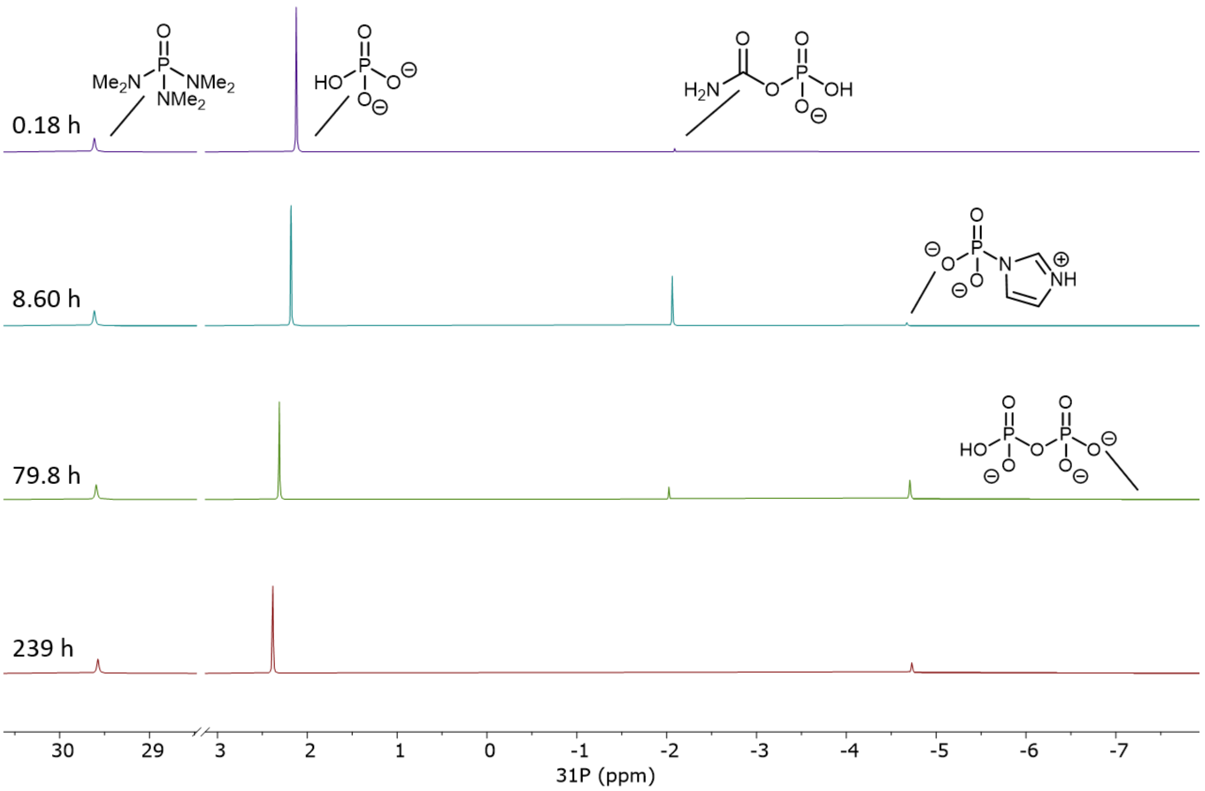

Supplementary Figure 5: Representative ^{31}P-NMR spectra over time for 450 mM potassium cyanate with 150 mM sodium phosphate and 50 mM HMPA internal standard in 1.0 M imidazole buffer. The solution was pH 7.32 at the start and this rose to pH 8.11 at the end of the reaction. The depicted speciation states are for pH 7.32. Changes in chemical shift of peaks are due to an increase in pH over time.

Supplementary Table 0.6: Average changes in percentage yield over time for each phosphorus species in the ^{31}P-NMR spectra (representative Supplementary Figure 6.5) for 450 mM potassium cyanate with 150 mM sodium phosphate and 50 mM HMPA internal standard in 1.0 M imidazole buffer pH 7.32-8.11 performed in triplicate. Standard deviation provided for each value was based on three repeats.

Time (h)	Phosphate (%)	Carbamoyl phosphate (%)	Imidazole phosphate (%)	Pyrophosphate (%)
0.18	97.58 ± 0.20	2.42 ± 0.20	0.00 ± 0.00	0.00 ± 0.00
2.87	79.79 ± 0.07	19.57 ± 0.01	0.65 ± 0.07	0.00 ± 0.00
5.25	71.82 ± 0.22	26.51 ± 0.18	1.67 ± 0.04	0.00 ± 0.00
8.60	65.08 ± 0.18	31.50 ± 0.11	3.42 ± 0.09	0.00 ± 0.00
13.35	61.05 ± 0.11	32.76 ± 0.12	6.19 ± 0.09	0.00 ± 0.00
18.25	59.59 ± 0.10	31.66 ± 0.04	8.75 ± 0.06	0.00 ± 0.00
23.34	58.98 ± 0.06	29.69 ± 0.08	11.29 ± 0.15	0.04 ± 0.01
32.06	59.21 ± 0.23	25.53 ± 0.05	15.20 ± 0.19	0.06 ± 0.01
49.31	60.91 ± 0.11	18.46 ± 0.01	20.60 ± 0.11	0.04 ± 0.00
56.99	62.09 ± 0.11	15.55 ± 0.02	22.28 ± 0.09	0.08 ± 0.05
71.36	64.16 ± 0.12	11.64 ± 0.03	24.15 ± 0.08	0.05 ± 0.01
79.84	65.55 ± 0.03	9.56 ± 0.08	24.82 ± 0.13	0.07 ± 0.02
95.39	68.09 ± 0.40	6.68 ± 0.06	25.13 ± 0.34	0.11 ± 0.03
103.91	69.35 ± 0.25	5.53 ± 0.09	25.03 ± 0.20	0.09 ± 0.01
135.42	73.84 ± 0.70	2.67 ± 0.11	23.36 ± 0.70	0.12 ± 0.03
167.61	78.20 ± 0.84	1.19 ± 0.10	20.51 ± 0.75	0.10 ± 0.00
191.49	80.51 ± 0.97	0.71 ± 0.07	18.60 ± 0.87	0.18 ± 0.04
220.61	83.17 ± 1.00	0.37 ± 0.04	16.29 ± 1.05	0.17 ± 0.01
239.38	84.61 ± 0.88	0.26 ± 0.06	14.92 ± 0.89	0.21 ± 0.03
267.12	86.67 ± 1.05	0.12 ± 0.02	13.01 ± 1.05	0.20 ± 0.02
335.44	90.37 ± 1.12	0.00 ± 0.00	9.38 ± 1.09	0.25 ± 0.03
390.53	92.37 ± 1.14	0.00 ± 0.00	7.37 ± 1.16	0.26 ± 0.04

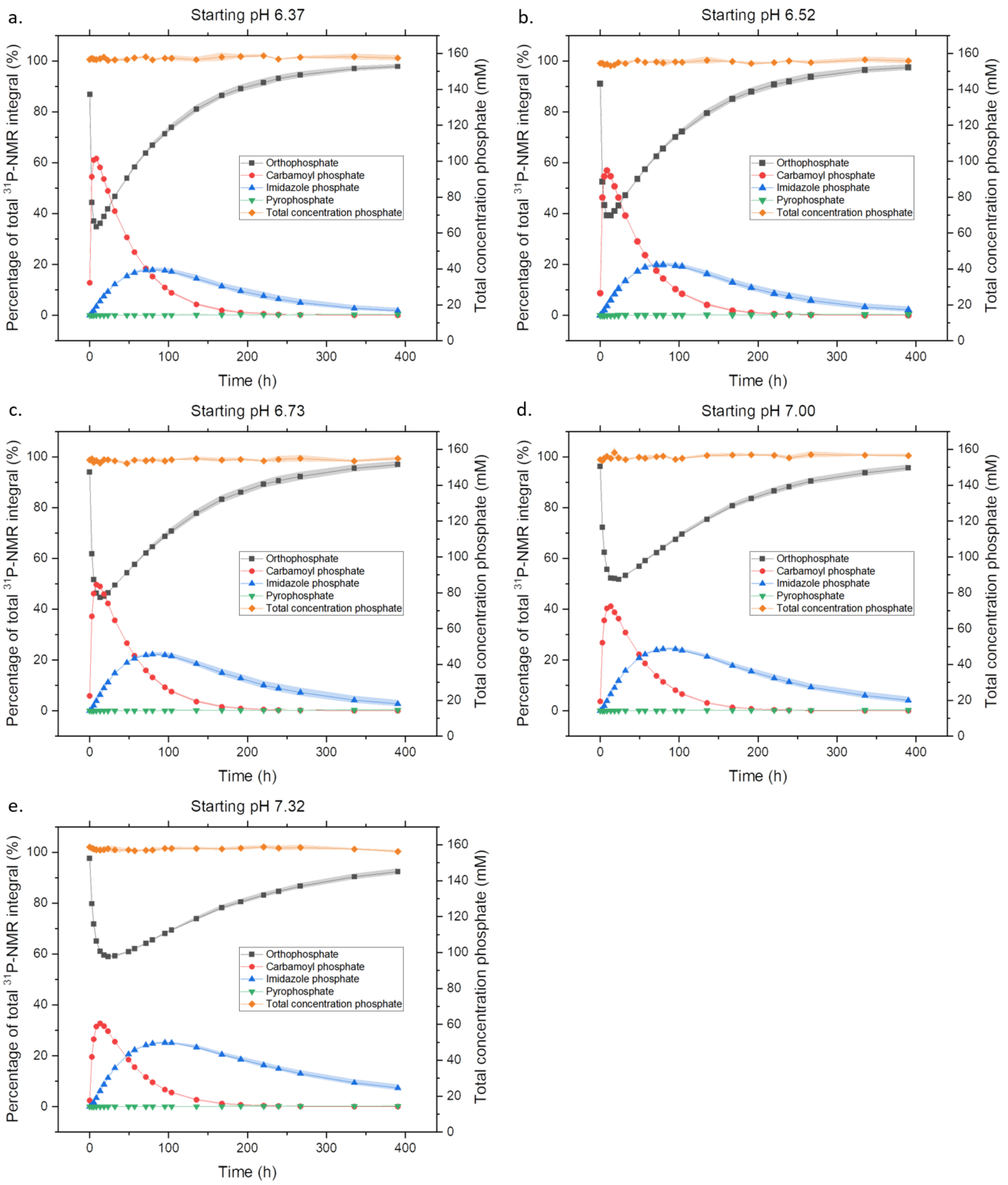

Supplementary Figure 6: The changes in yield over time for the phosphorous species for the reactions with 450 mM potassium cyanate with 150 mM sodium phosphate in 1.0 M imidazole buffer with 50 mM HMPA internal standard at starting pHs: a) pH 6.37, b) pH 6.52, c) pH 6.73, d) pH 7.00 and e) pH 7.32. Standard deviations based upon three repeats are indicated as shaded areas around the trace. Data for plots taken from Supplementary Tables 0.2 – 0.6. See Supplementary Figure 1-5 for representative ^{31}P NMR spectra. Total concentration of phosphate (right hand axis) represents the total of the concentrations of all phosphate species in solution determined with reference to internal standard HMPA.

S2.2.2 Supplementary experimental method for formation of imidazole phosphate in the presence of Mg^{2+} and Zn^{2+} ions and Results

An identical procedure was used as detailed in Section S2.2.1 but with the addition that at the start of the reaction either 1.9 mg of magnesium chloride (0.02 mmol) or 2.7 mg of zinc chloride (0.02 mmol) to 1.0 mL of the reaction solution to give a 20 mM concentration of the metal chloride.

Supplementary Figures 7 to 8 depict representative ^{31}P NMR spectra for these reactions. The changes in yield over time for orthophosphate, carbamoyl phosphate and imidazole phosphate are shown in Supplementary Figure 9.

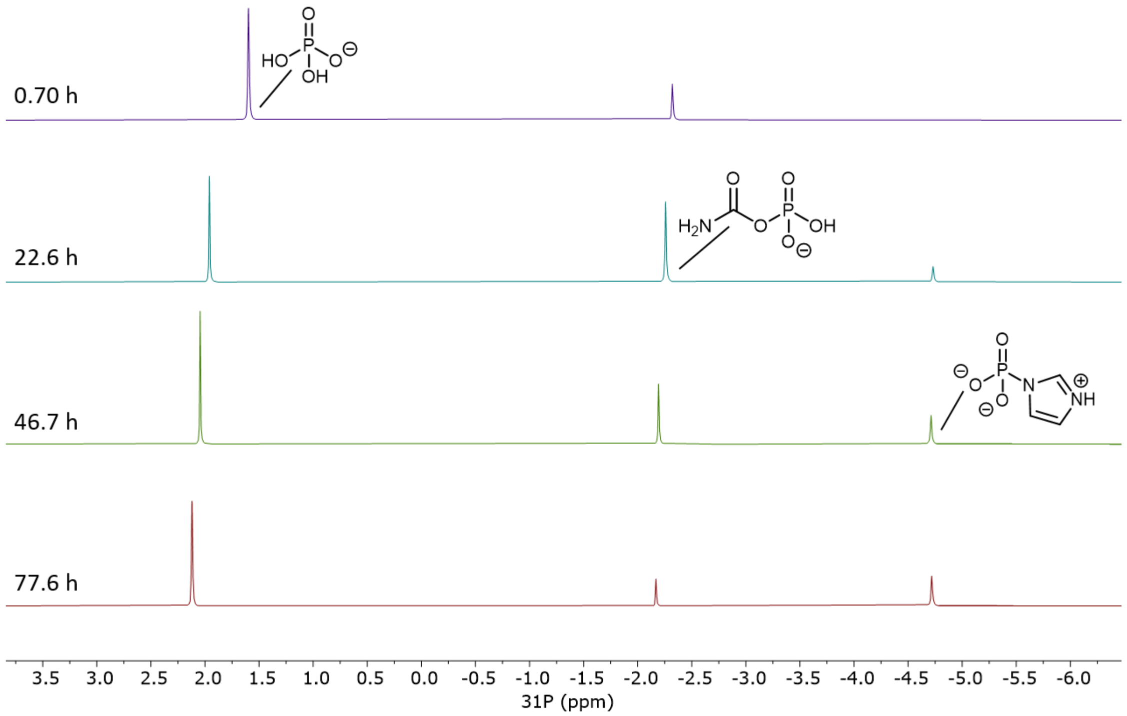

Supplementary Figure 7: Representative ^{31}P-NMR spectra over time for 150 mM sodium phosphate with 450 mM potassium cyanate and 20 mM $MgCl_2$ in 1.0 M imidazole buffer. The solution was pH 6.46 at the start and this rose to pH 7.32 at the end of the reaction. The depicted speciation states are for pH 6.46.

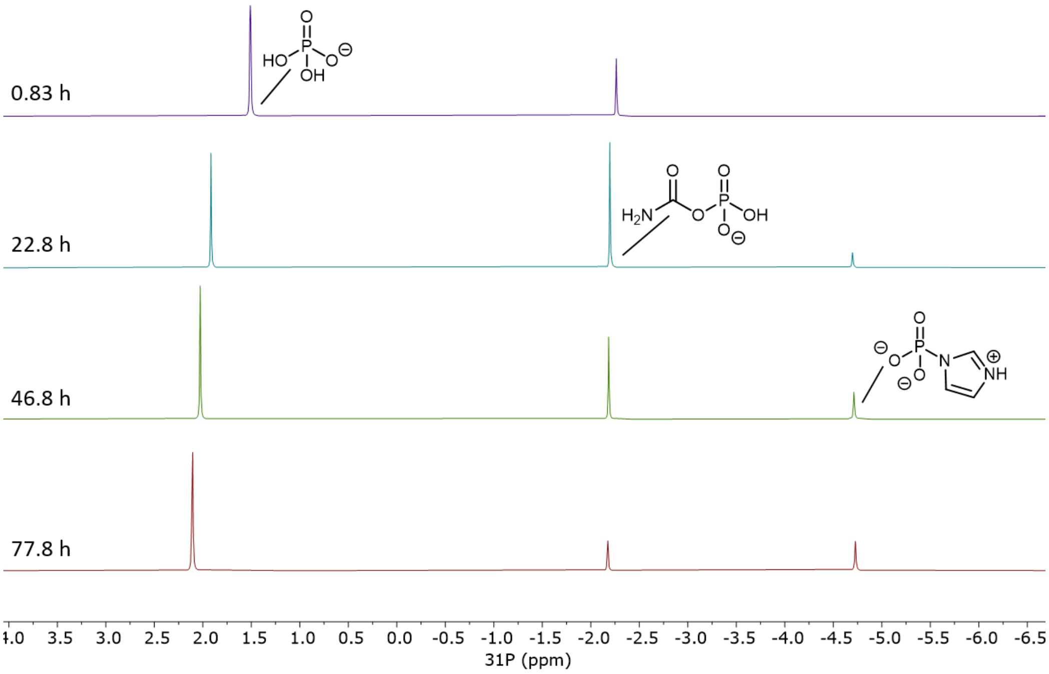

Supplementary Figure 8: Representative ^{31}P-NMR spectra over time for 150 mM sodium phosphate with 450 mM potassium cyanate and 20 mM $ZnCl_2$ in 1.0 M imidazole buffer. The solution was pH 6.34 at the start and this rose to pH 7.27 at the end of the reaction. The depicted speciation states are for pH 6.46.

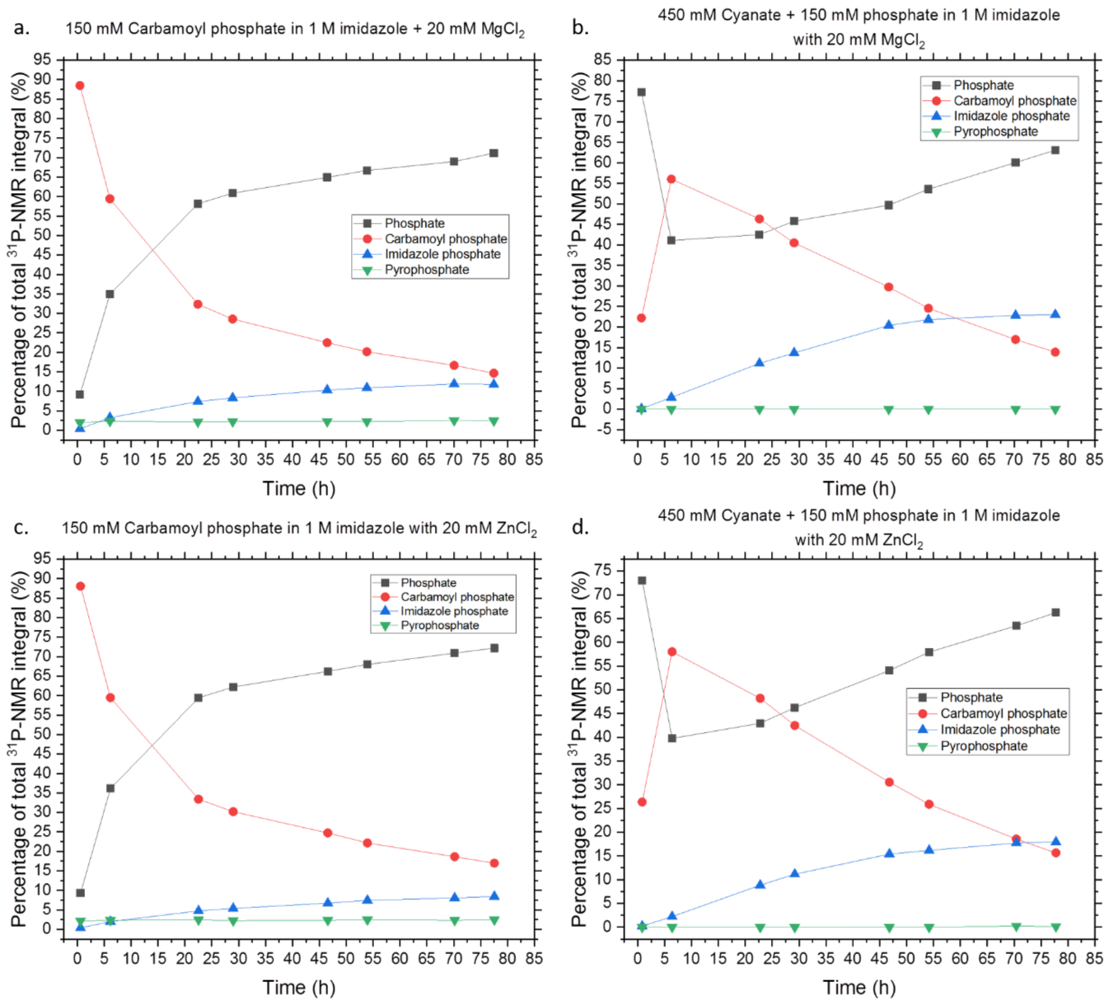

Supplementary Figure 9: The changes in yield of phosphorous species over time for the reactions shown in Supplementary Figure 7 – 8 and Supplementary Figure 17 – 18. a) 150 mM lithium carbamoyl phosphate with 20 mM $MgCl_2$ in 1.0 M imidazole pH 6.5; b) 450 mM potassium cyanate with 150 mM sodium phosphate and 20 mM $MgCl_2$ in 1.0 M imidazole pH 6.25; c) 150 mM lithium carbamoyl phosphate with 20 mM $ZnCl_2$ in 1.0 M imidazole pH 6.5; d) 450 mM potassium cyanate with 150 mM sodium phosphate and 20 mM $ZnCl_2$ in 1.0 M imidazole pH 6.25.

S2.2.3 Control experiment in the absence of potassium cyanate

A control sample was performed to confirm that no imidazole phosphate was formed in the absence of potassium cyanate. The experiment was performed with 150 mM phosphate in 1.0 M imidazole buffer was prepared by dissolving solid sodium phosphate monobasic anhydrous (12.5 mg, 0.104 mmol, 1 eq) in 693 µL 1.0 M imidazole buffer pH 6.42, obtaining a sample with starting pH 6.11. The reaction was followed by ^{31}P-NMR spectroscopy, measuring spectra at a series of time points over the course of the first 23.4 hours of the reaction.

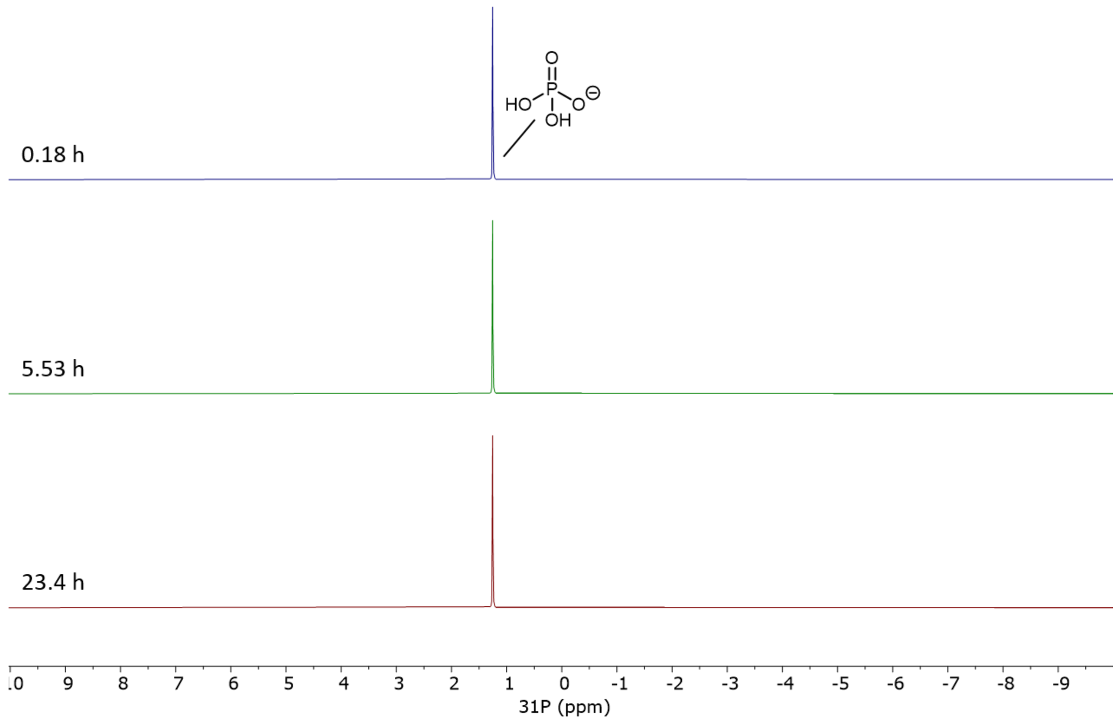

Supplementary Figure 10: Representative ^{31}P-NMR spectra over time for the control reaction of 150 mM sodium phosphate in 1.0 M imidazole buffer pH 6.1. Speciation states are represented for pH 6.1. No other phosphorus containing species than orthophosphate are observed.

S2.3 Formation of imidazole phosphate from carbamoyl phosphate in imidazole buffer

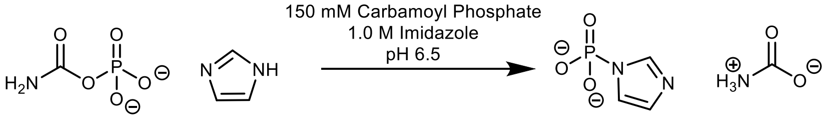

S2.3.1 Supplementary experimental method

A 150 mM lithium carbamoyl phosphate in 1.0 M imidazole buffer was prepared by dissolving lithium carbamoyl phosphate dibasic hydrate (16 mg, 0.1 mmol, 1 eq) in 700 µL 1.0 M imidazole buffer at pH 6.42 (0.7 mmol, 6.67 eq). The pH of the solution at the start was pH 6.51. The reaction was followed by ^{31}P NMR and ^{1}H NMR spectroscopy, measuring spectra at a series of time points over the course of 384 h.

Additional potassium cyanate was added to the solution at 56.4 h, 128.2 h and 316.2 h by adding 53.7 µL of 2.0 M cyanate (0.1 mmol, 1 eq).

An ^{1}H ^{31}P HMBC spectrum was measured 319 hours after the start of the reaction. ^{1}H ^{13}C HSQC spectra were measured 146 and 198 hours after the start of the reaction. An ^{1}H ^{13}C HMBC spectrum was

measured 198 hours after the start of the reaction. The pH of the sample was measured at different time points over the course of the reaction.

Supplementary Figure 11 depicts representative ^{31}P NMR spectra for the reaction. The changes in yield over time for orthophosphate, carbamoyl phosphate and imidazole phosphate are shown in Supplementary Figure 13.

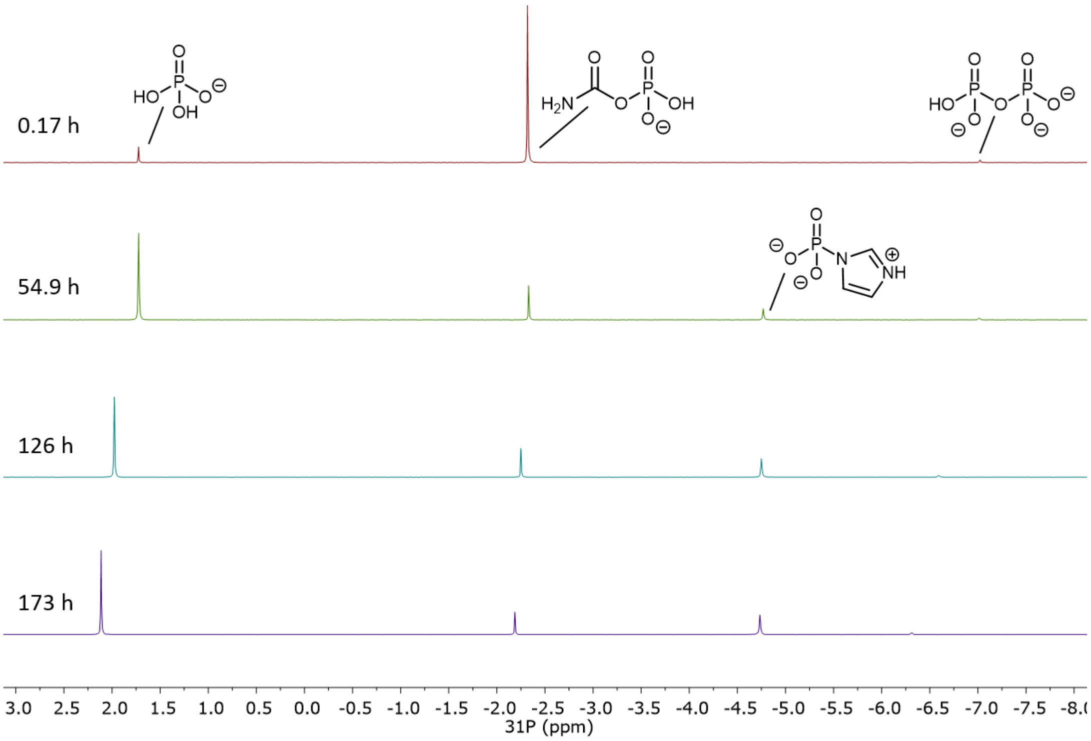

Supplementary Figure 11: Representative ^{31}P-NMR spectra over time for 150 mM lithium carbamoyl phosphate in 1.0 M imidazole buffer. The solution was pH 6.51 at the start and this rose to pH 7.29 at the end of the reaction. One equivalent potassium cyanate was added at 56.4, 128.2 and 316.2 h. The depicted speciation states are for pH 6.51.

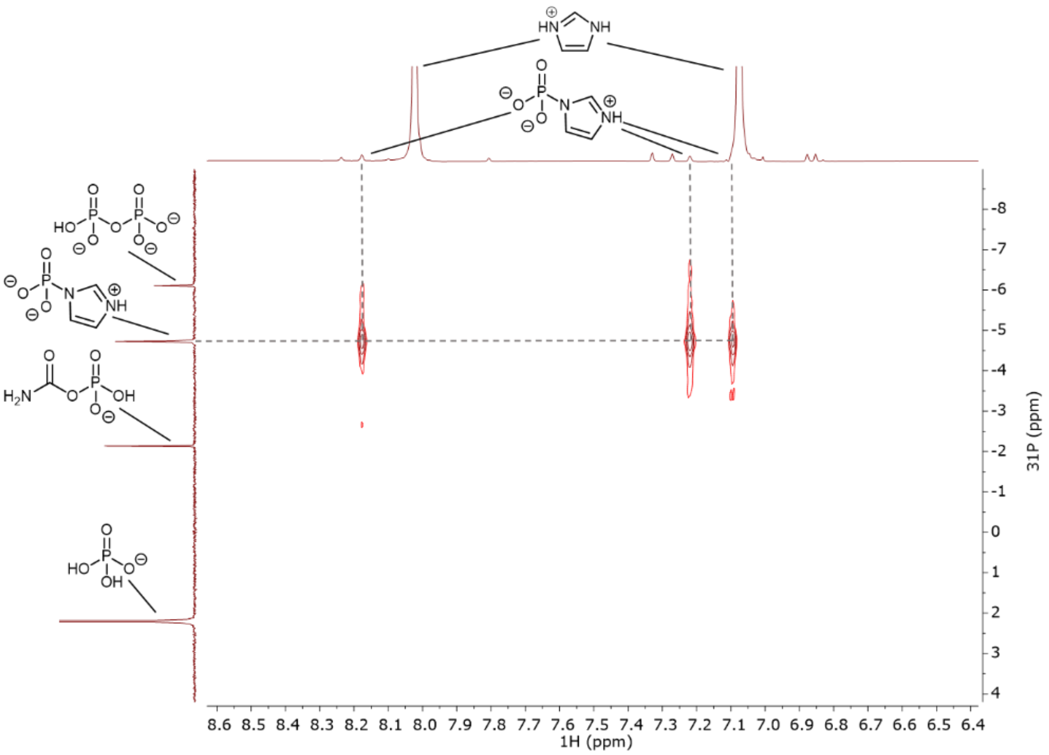

Supplementary Figure 12: ^{1}H ^{31}P-HMBC NMR-spectrum for 150 mM lithium carbamoyl phosphate in 1.0 M imidazole buffer pH 7.29 at 319 h after the start of the reaction. The depicted speciation states are for pH 6.51.

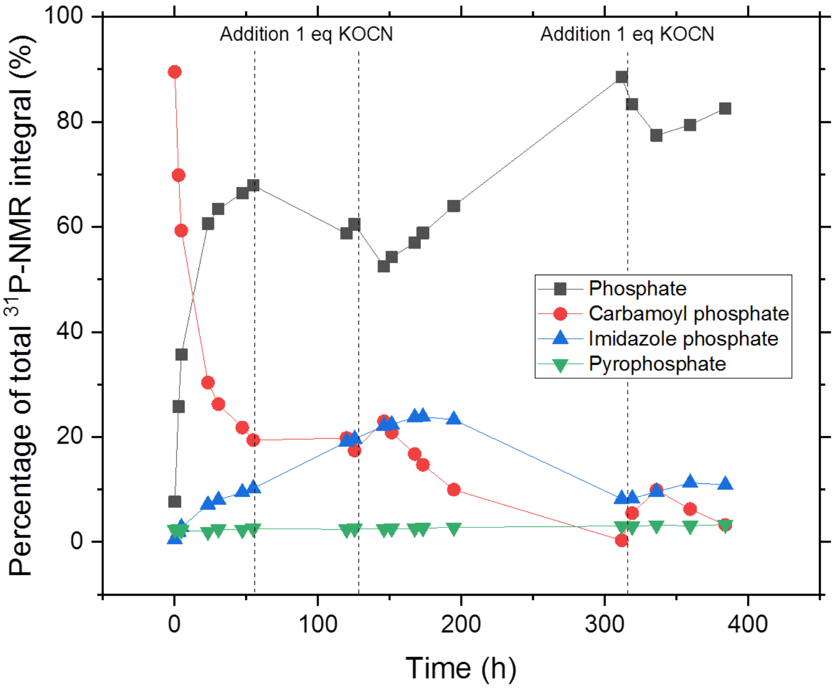

Supplementary Figure 13: The changes in yield of phosphorous species over time for the reaction of 150 mM lithium carbamoyl phosphate in 1.0 M imidazole buffer. The solution was pH 6.51 at the start and this rose to pH 7.29 at the end of the reaction. Dotted vertical lines represent when one equivalent potassium cyanate was added at 56.4, 128.2 and 316.2 h. See Supplementary Figure 11 for representative ^{31}P NMR spectra.

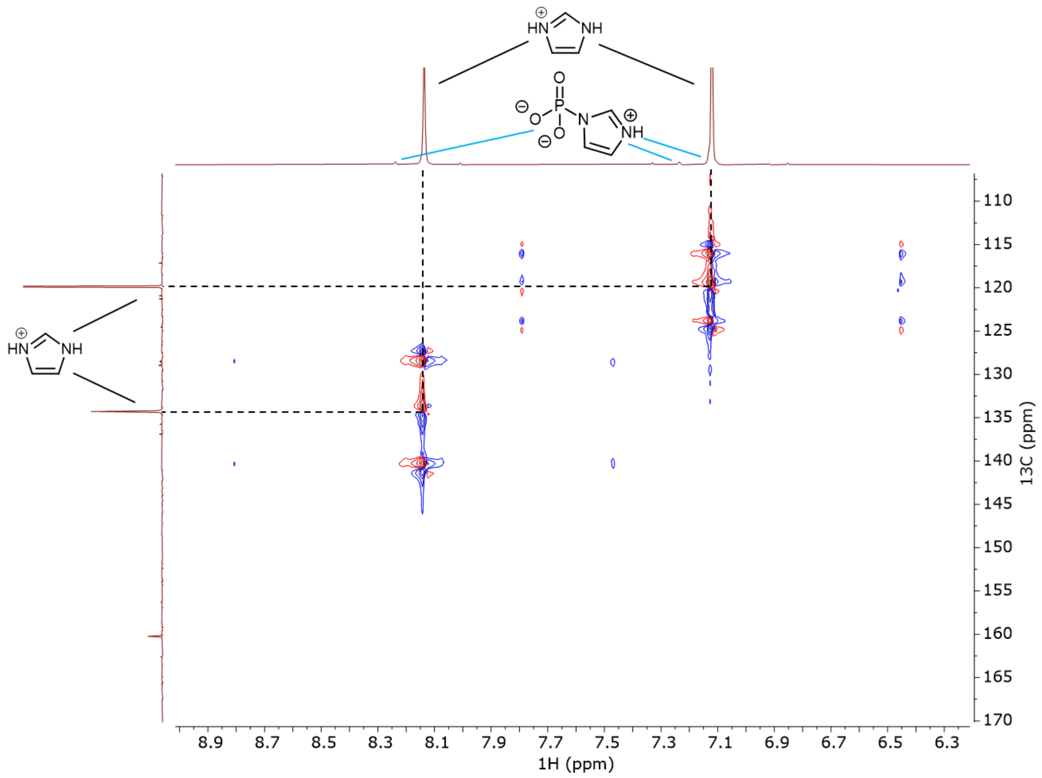

Supplementary Figure 14: ^{1}H ^{13}C-HSQC NMR-spectrum for 150 mM lithium carbamoyl phosphate in 1.0 M imidazole buffer pH 7.04 at 146 h after the start of the reaction. The depicted speciation states are for pH 6.51.

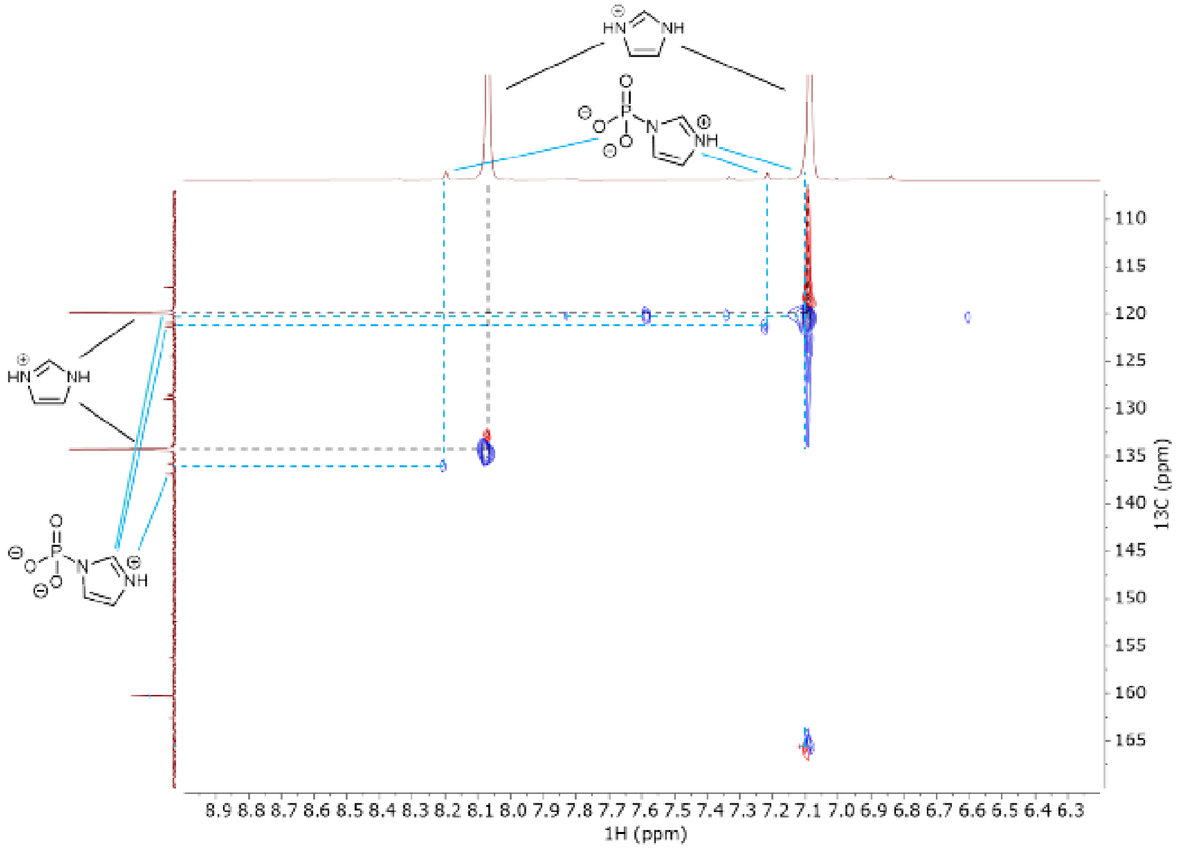

Supplementary Figure 15: ^{1}H ^{13}C-HSQC NMR-spectrum for 150 mM lithium carbamoyl phosphate in 1.0 M imidazole buffer pH 7.29 at 198 h after the start of the reaction. The depicted speciation states are for pH 6.51.

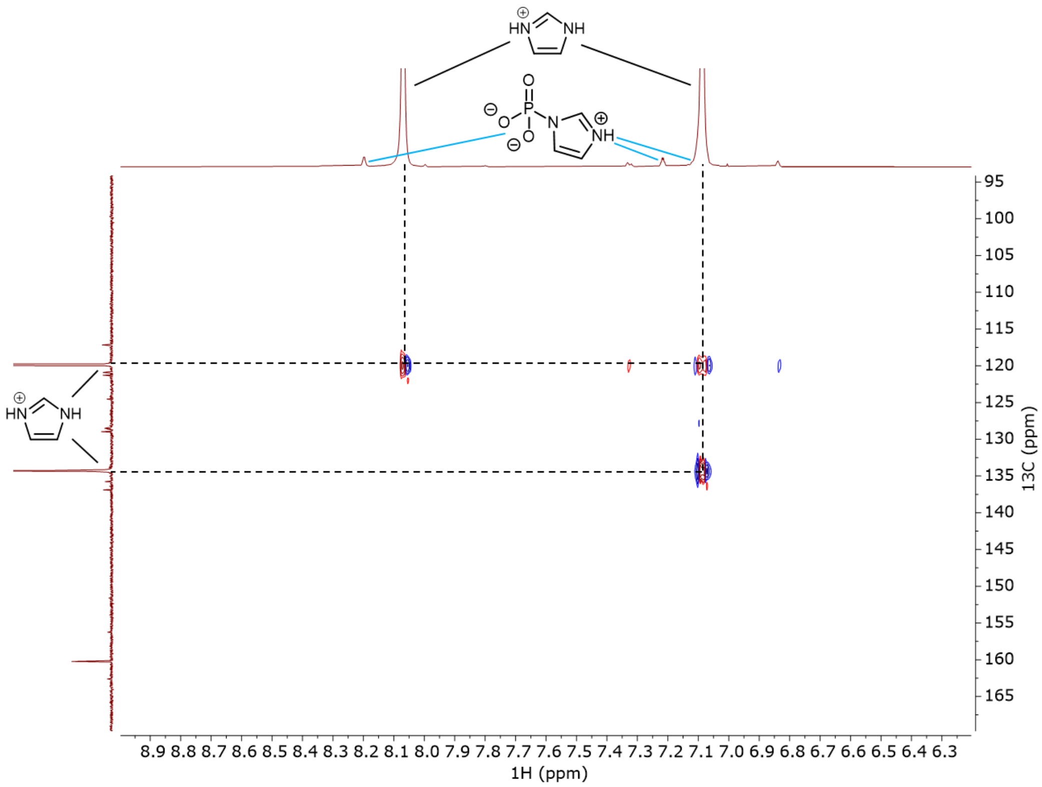

Supplementary Figure 16a: ^{1}H ^{13}C-HMBC NMR-spectrum for 150 mM lithium carbamoyl phosphate in 1.0 M imidazole buffer pH 7.29 at 198 h after the start of the reaction. The depicted speciation states are for pH 6.51.

S2.3.2 Formation of imidazole phosphate from carbamoyl phosphate in imidazole buffer with no addition of cyanate

An experiment was performed to demonstrate the formation of imidazole phosphate from carbamoyl phosphate with no addition of potassium cyanate. A solution of 50 mM lithium carbamoyl phosphate and 0.5 M imidazole was prepared in 9 : 1 H_2O : D_2O. The solution was adjusted to pH 6.53 at the start. The reaction was followed over time using ^{31}P NMR spectroscopy.

Supplementary Figure 16b depicts representative ^{31}P NMR spectra for the reaction. Formation of imidazole phosphate was observed as well as the breakdown of carbamoyl phosphate into orthophosphate and cyanate. The changes in yield over time for orthophosphate, carbamoyl phosphate and imidazole phosphate are shown in Supplementary Figure 16c.

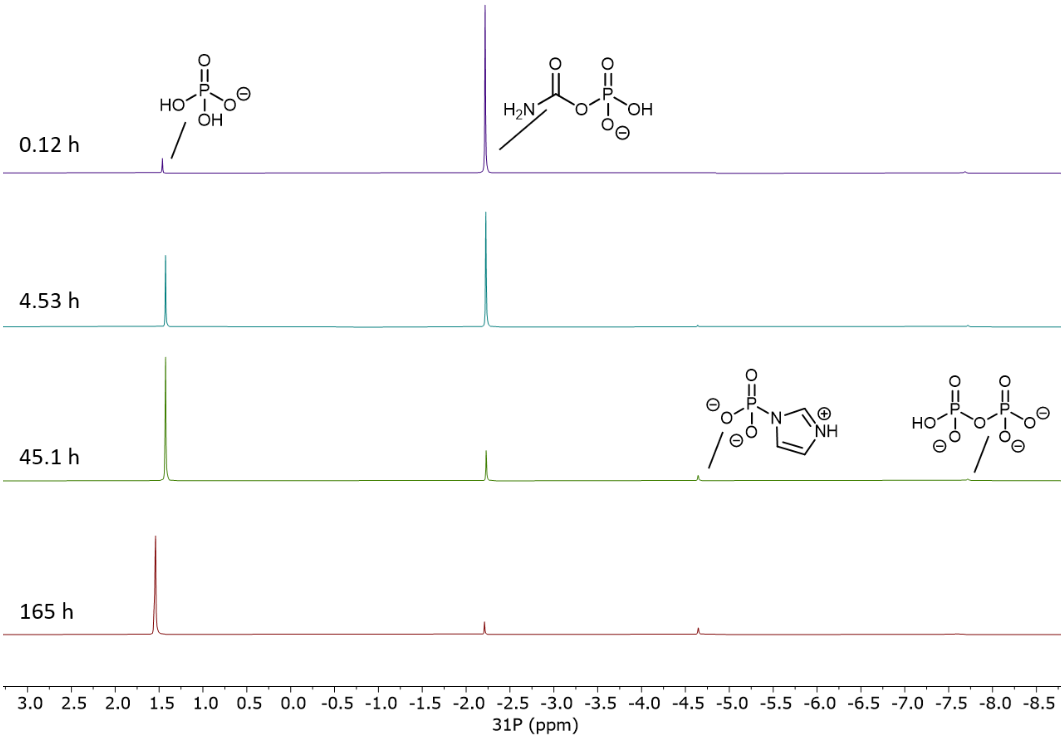

Supplementary Figure 16b: Representative ^{31}P-NMR spectra over time for 50 mM lithium carbamoyl phosphate in 0.5 M imidazole buffer. The solution was pH 6.53 at the start and this rose to pH 6.67 at the end of the reaction. Speciation states are represented for pH 6.53.

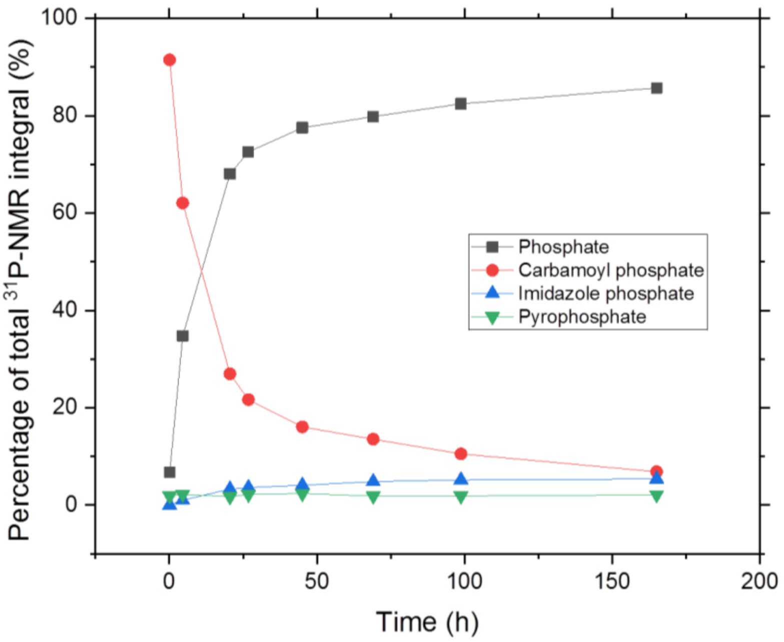

Supplementary Figure 16c: The changes in yield of phosphorous species over time for the reaction of 50 mM lithium carbamoyl phosphate in 0.5 M imidazole buffer. The solution was pH 6.53 at the start and this rose to pH 6.67 at the end of the reaction. See Supplementary Figure 16b for representative ^{31}P NMR spectra.

S2.3.3 Experimental Method for formation of imidazole phosphate in the presence of Mg^{2+} and Zn^{2+} ions and Results

An identical procedure was used as detailed in Section S3.1.1 but with the addition that at the start of the reaction either 1.9 mg of magnesium chloride (0.02 mmol) or 2.7 mg of zinc chloride (0.02 mmol) to 1.0 mL of the reaction solution to give a 20 mM concentration of the metal chloride. No potassium cyanate was added over the course of the reaction.

in Supplementary Figures 17 to 18 depict representative ^{31}P NMR spectra for these reactions. The changes in yield over time for orthophosphate, carbamoyl phosphate and imidazole phosphate are shown in Supplementary Figure 9.

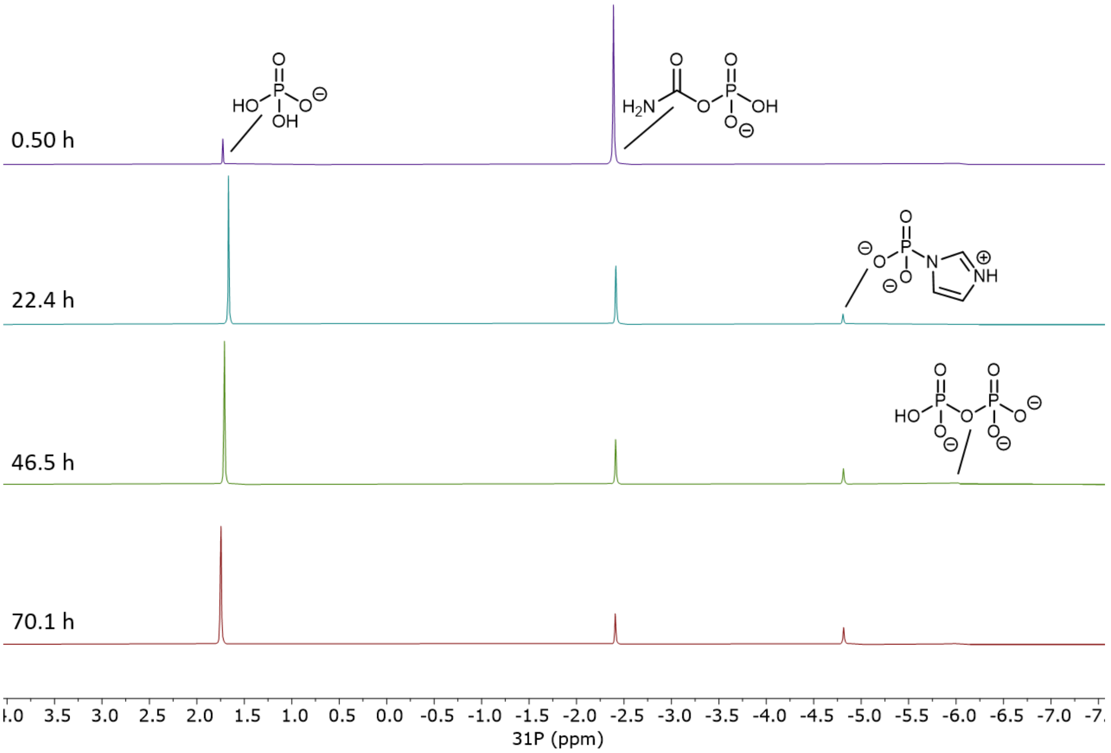

Supplementary Figure 17: Representative ^{31}P-NMR spectra over time for 150 mM lithium carbamoyl phosphate with 20 mM $MgCl_2$ in 1.0 M imidazole buffer. The solution was pH 6.49 at the start and this rose to pH 6.67 at the end of the reaction. The depicted speciation states are for pH 6.49.

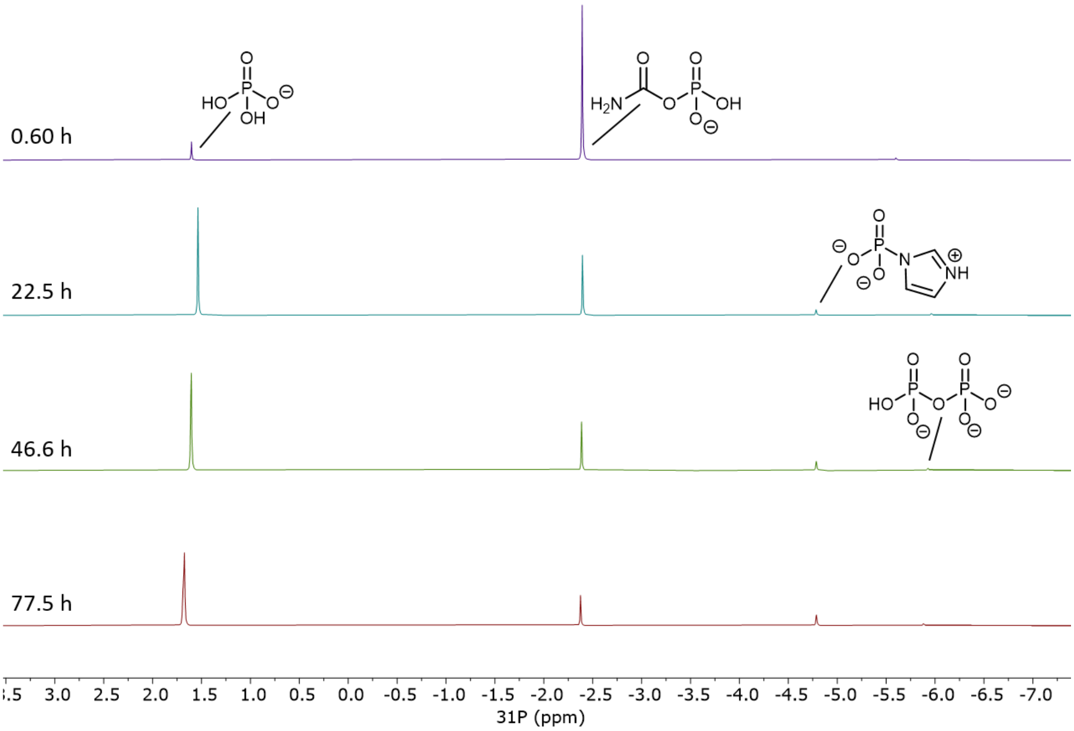

Supplementary Figure 18: Representative ^{31}P-NMR spectra over time for 150 mM lithium carbamoyl phosphate with 20 mM $ZnCl_2$ in 1.0 M imidazole buffer. The solution was pH 6.27 at the start and this rose to pH 6.47 at the end of the reaction. The depicted speciation states are for pH 6.27.

S2.4 Formation of activated phosphoramidate from carbamoyl phosphate with imidazole derivatives and pyrazole

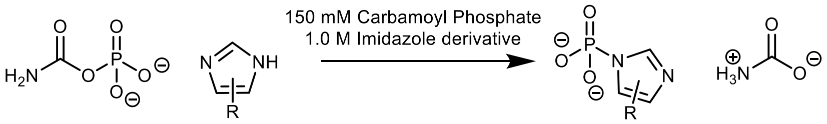

S2.4.1 Supplementary experimental method

A 150 mM lithium carbamoyl phosphate and 300 mM potassium cyanate in 1.0 M imidazole buffer was prepared by dissolving lithium carbamoyl phosphate dibasic hydrate (23 mg, 0.15 mmol, 1 eq) and potassium cyanate (23.4 mg, 0.3 mmol, 2 eq) of in 1.0 mL of 1.0 M imidazole derivative buffer 9 : 1 H_2O : D_2O (1.0 mmol, 6.67 eq). The reaction was followed by ^{31}P NMR and ^{1}H NMR spectroscopy, measuring spectra at a series of time points over the course of 18 - 65 h. An ^{1}H ^{31}P HMBC spectrum was also taken. The pH of the sample was measured at different time points over the course of the reaction.

S2.4.2 Orthophosphate activation with 1-methylimidazole

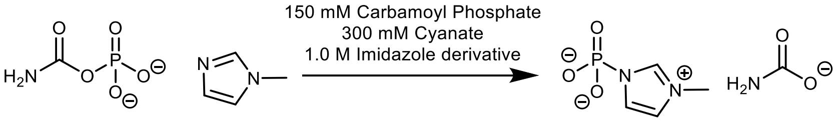

The reaction was performed as described in Section S2.4.1 with 82 mg of 1-methylimidazole dissolved in 1.0 mL of 9 : 1 H_2O : D_2O to give a 1.0 M solution. pH at the start of the reaction was 7.32.

Supplementary Figure 19 depicts representative ^{31}P NMR spectra for the reaction. The changes in yield over time for orthophosphate, carbamoyl phosphate and phosphorylated 1-methylimidazole are shown in Supplementary Figure 21. The highest observed yield of phosphorylated 1-methylimidazole was 19.8 % at 53.6 h In Supplementary Figure 21.

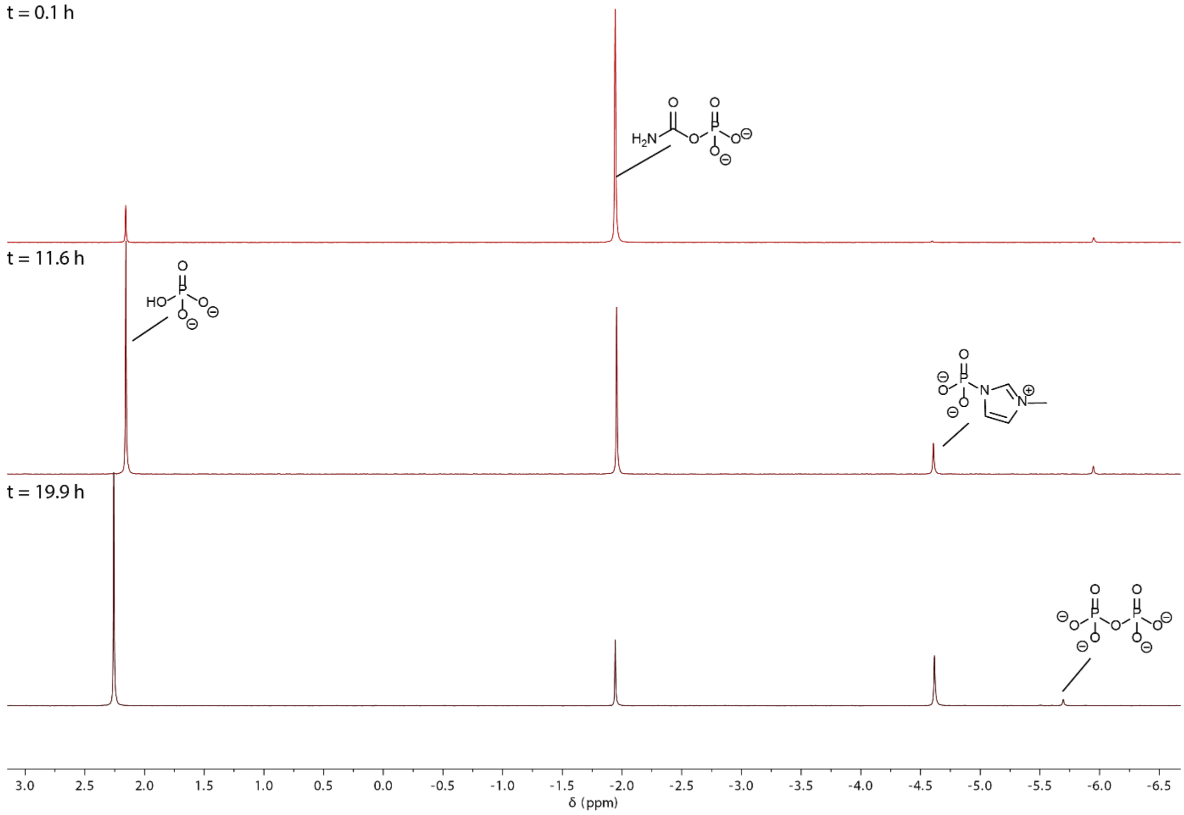

Supplementary Figure 19: Representative ^{31}P-NMR spectra over time for the formation of phosphorylated 1-methylimidazole in a 150 mM lithium carbamoyl phosphate, 300 mM potassium cyanate and 1.0 M 1-methylimidazole solution.

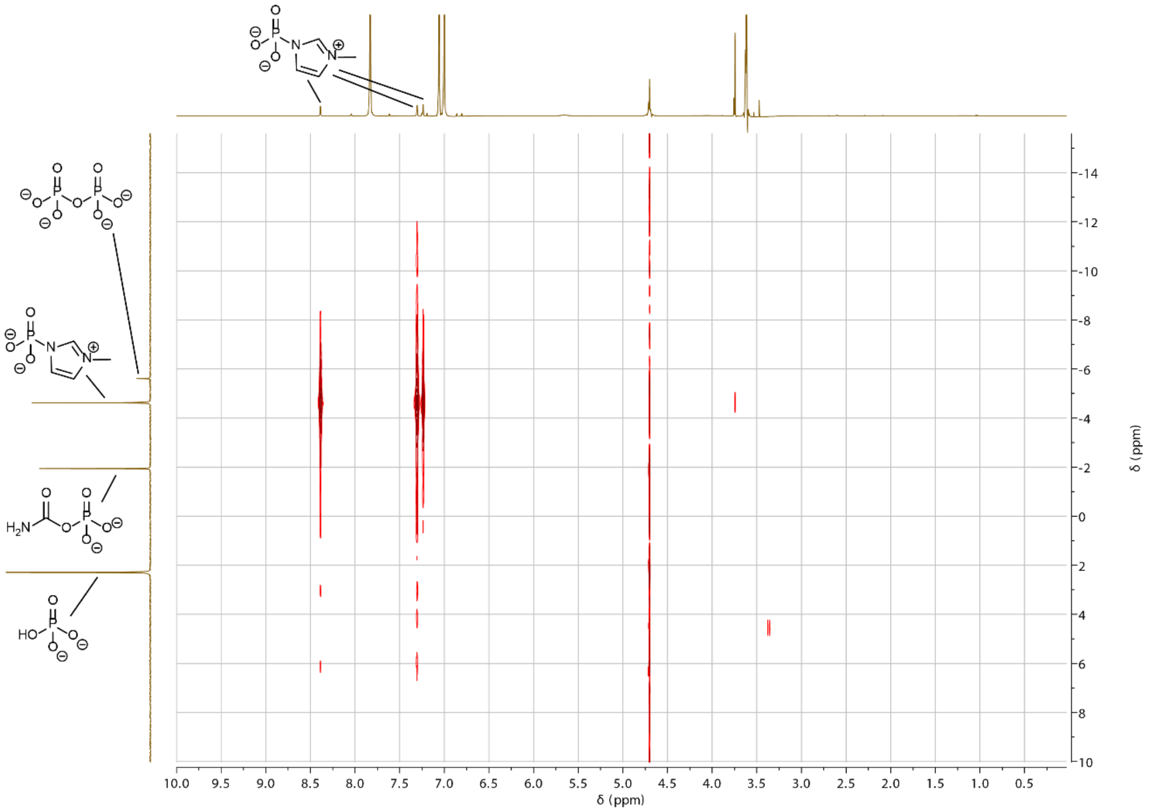

Supplementary Figure 20: ^{1}H ^{31}P-HMBC NMR-spectrum for phosphorylated 1-methylimidazole in a 150 mM lithium carbamoyl phosphate, 300 mM potassium cyanate and 1.0 M 1-methylimidazole solution. x axis = ^{1}H NMR and y axis = ^{31}P NMR.

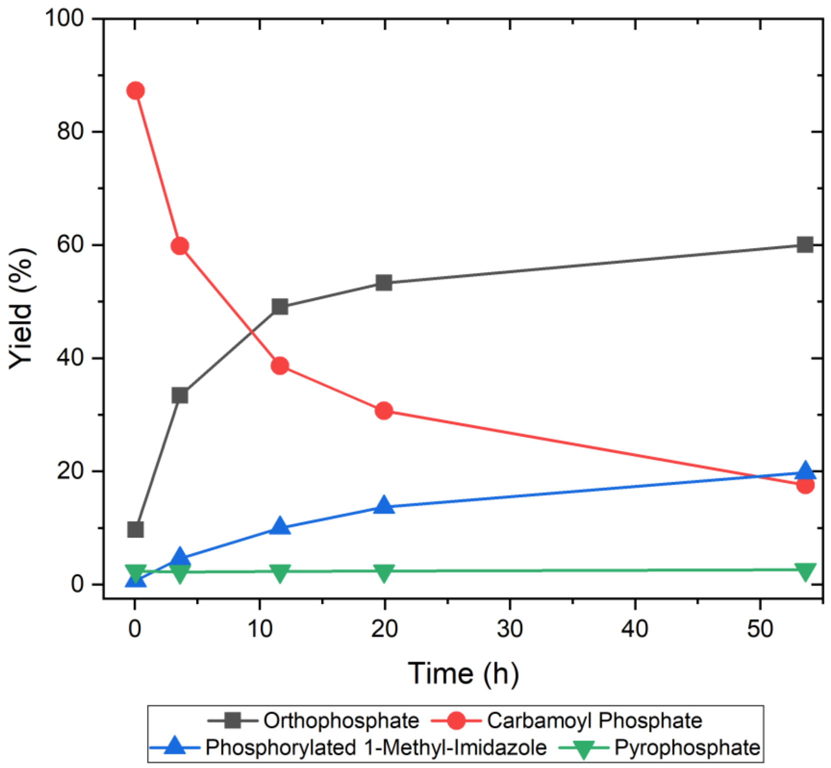

Supplementary Figure 21: Changes in yield over time for the formation phosphorylated 1-methylimidazole in a 150 mM lithium carbamoyl phosphate, 300 mM potassium cyanate and 1.0 M 1-methylimidazole solution.

S2.4.3 Orthophosphate activation with 2-aminoimidazole

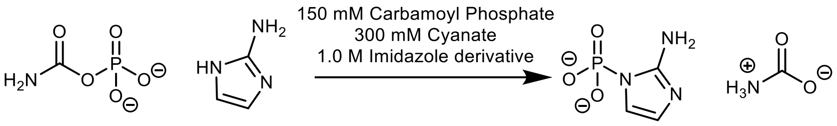

The reaction was performed as described in Section S2.4.1 with 83.1 mg of 2-aminoimidazole dissolved in 1.0 mL of 9 : 1 H_2O : D_2O to give a 1.0 M solution. pH at the start of the reaction was 8.98.

Supplementary Figure 22 depicts representative ^{31}P NMR spectra for the reaction. The changes in yield over time for orthophosphate, carbamoyl phosphate and phosphorylated 2-aminoimidazole are shown in Supplementary Figure 24. The highest observed yield of phosphorylated 2-aminoimidazole was 4.6 % at 13.2 h in Supplementary Figure 24.

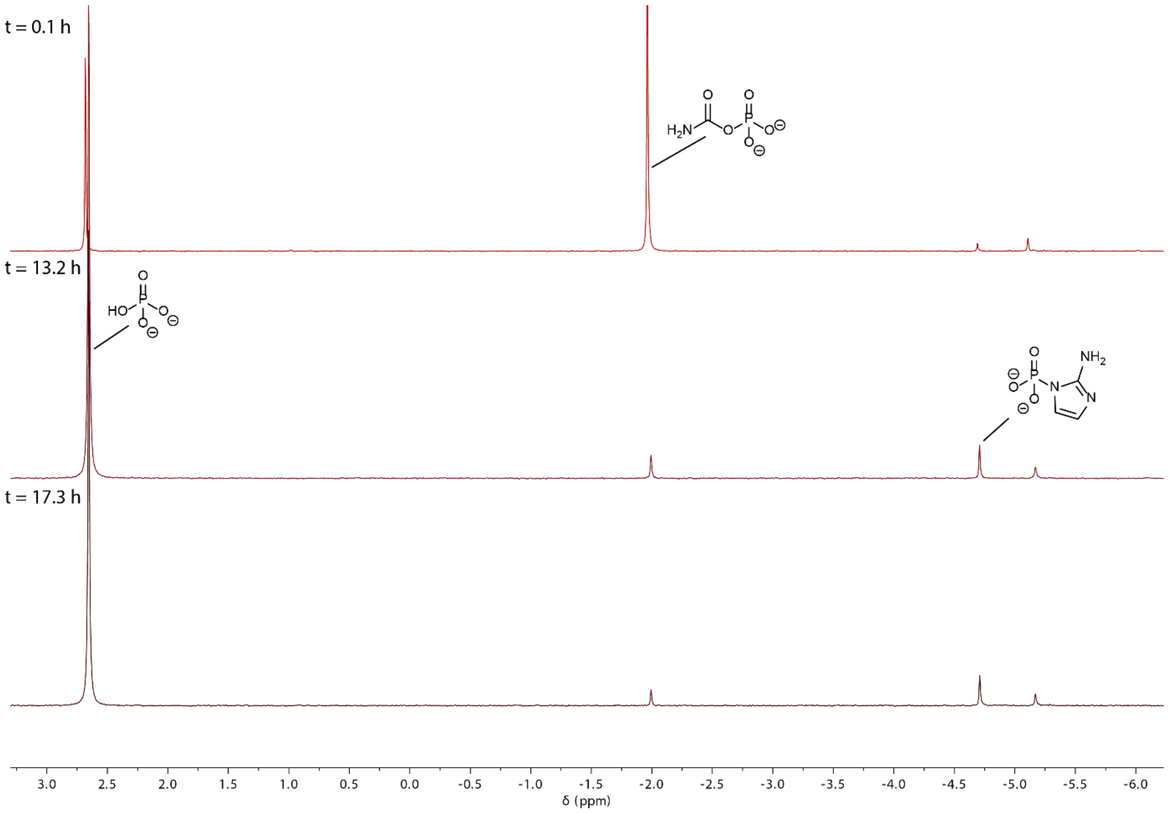

Supplementary Figure 22: Representative ^{31}P-NMR spectra over time for the formation of phosphorylated 2-aminoimidazole in a 150 mM lithium carbamoyl phosphate, 300 mM potassium cyanate and 1.0 M 2-aminoimidazole solution.

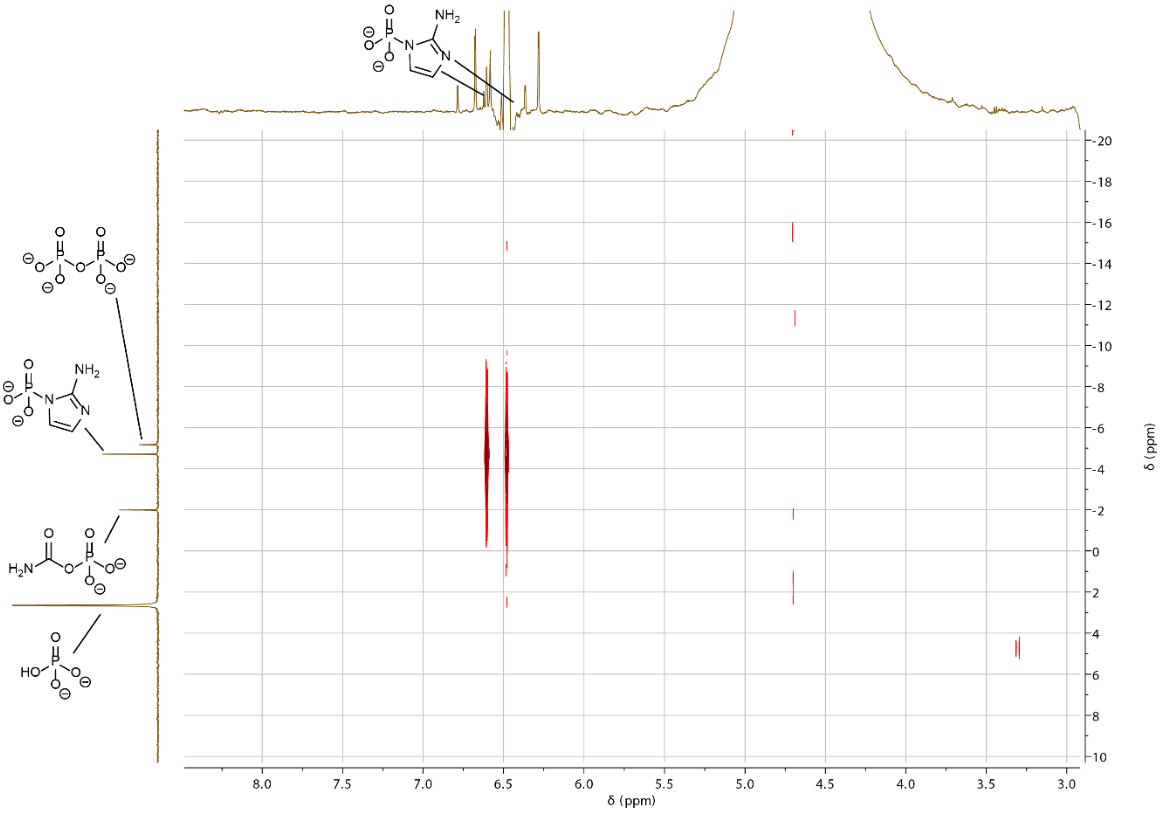

Supplementary Figure 23: ^{1}H ^{31}P-HMBC NMR-spectrum for phosphorylated 2-aminoimidazole in a 150 mM lithium carbamoyl phosphate, 300 mM potassium cyanate and 1.0 M 2-aminoimidazole solution. x axis = ^{1}H NMR and y axis = ^{31}P NMR.

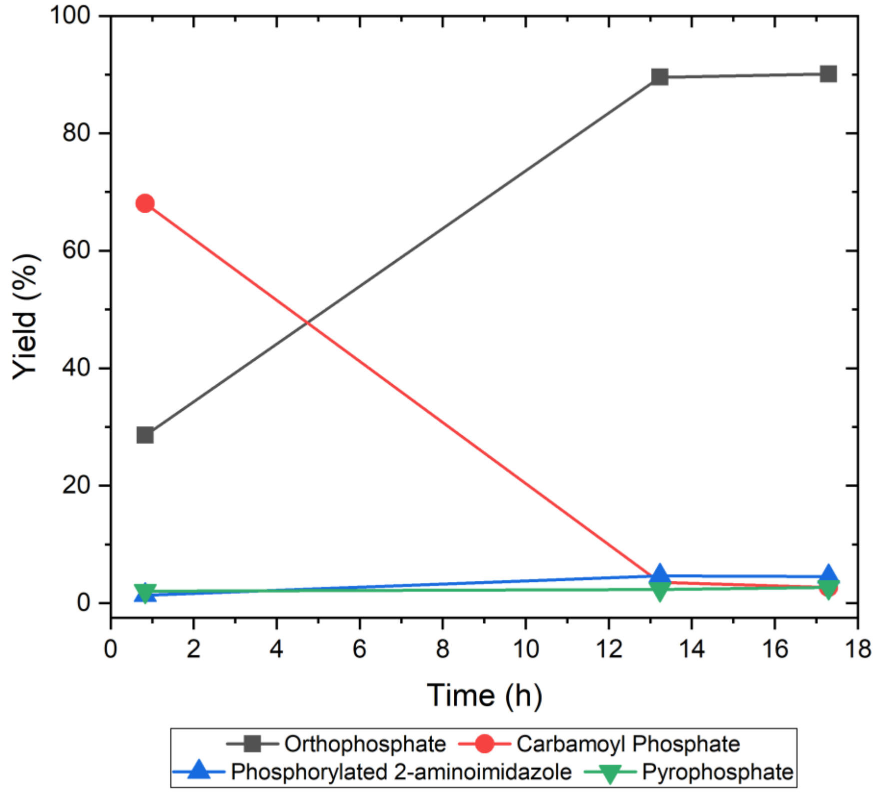

Supplementary Figure 24: Changes in yield over time for the formation phosphorylated 2-aminoimidazole in a 150 mM lithium carbamoyl phosphate, 300 mM potassium cyanate and 1.0 M 2-aminoimidazole solution.

S2.4.4 Orthophosphate activation with pyrazole

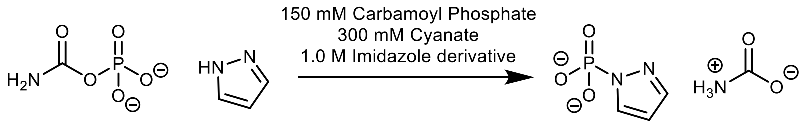

The reaction was performed as described in Section S2.4.1 with 68 mg of pyrazole dissolved in 1.0 mL of 9 : 1 H_2O : D_2O to give a 1.0 M solution. pH at the start of the reaction was 7.40.

Supplementary Figure 25 depicts representative ^{31}P NMR spectra for the reaction. The changes in yield over time for orthophosphate, carbamoyl phosphate and phosphorylated pyrazole are shown in Supplementary Figure 27. The highest observed yield of phosphorylated pyrazole was 7.1 % at 64.0 h in Supplementary Figure 27.

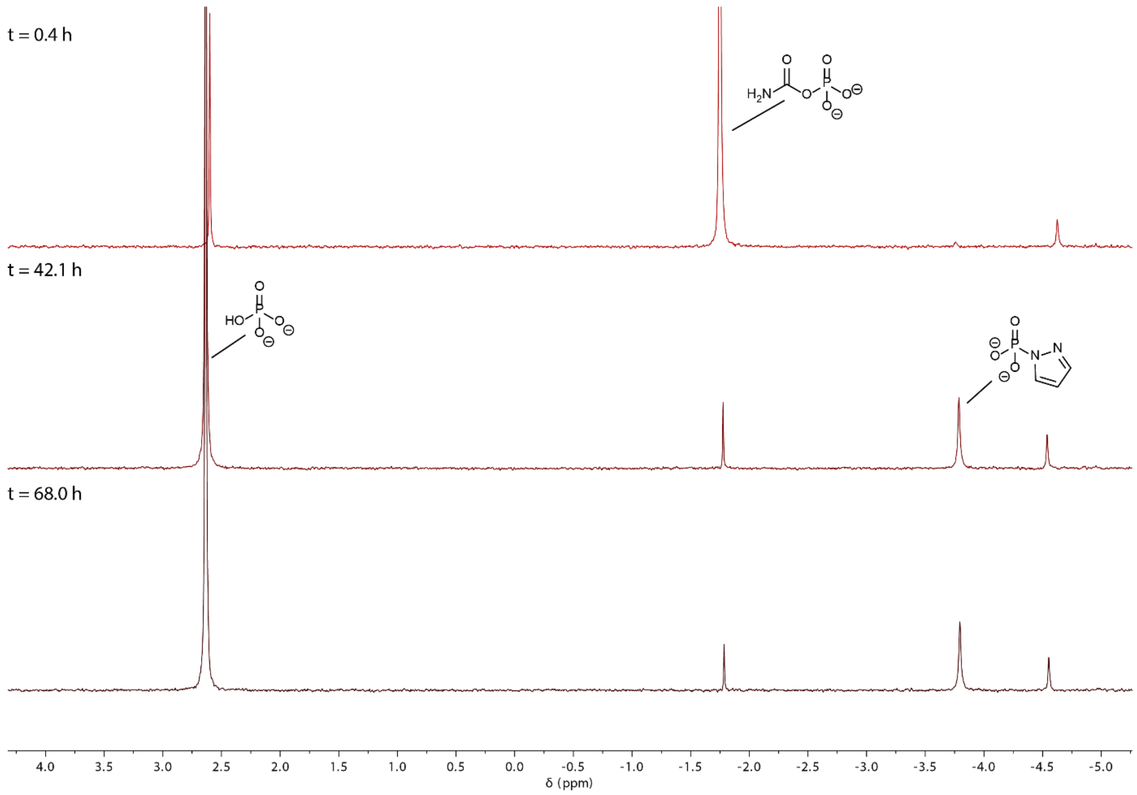

Supplementary Figure 25: Representative ^{31}P-NMR spectra over time for the formation of phosphorylated pyrazole in a 150 mM lithium carbamoyl phosphate, 300 mM potassium cyanate and 1.0 M pyrazole solution.

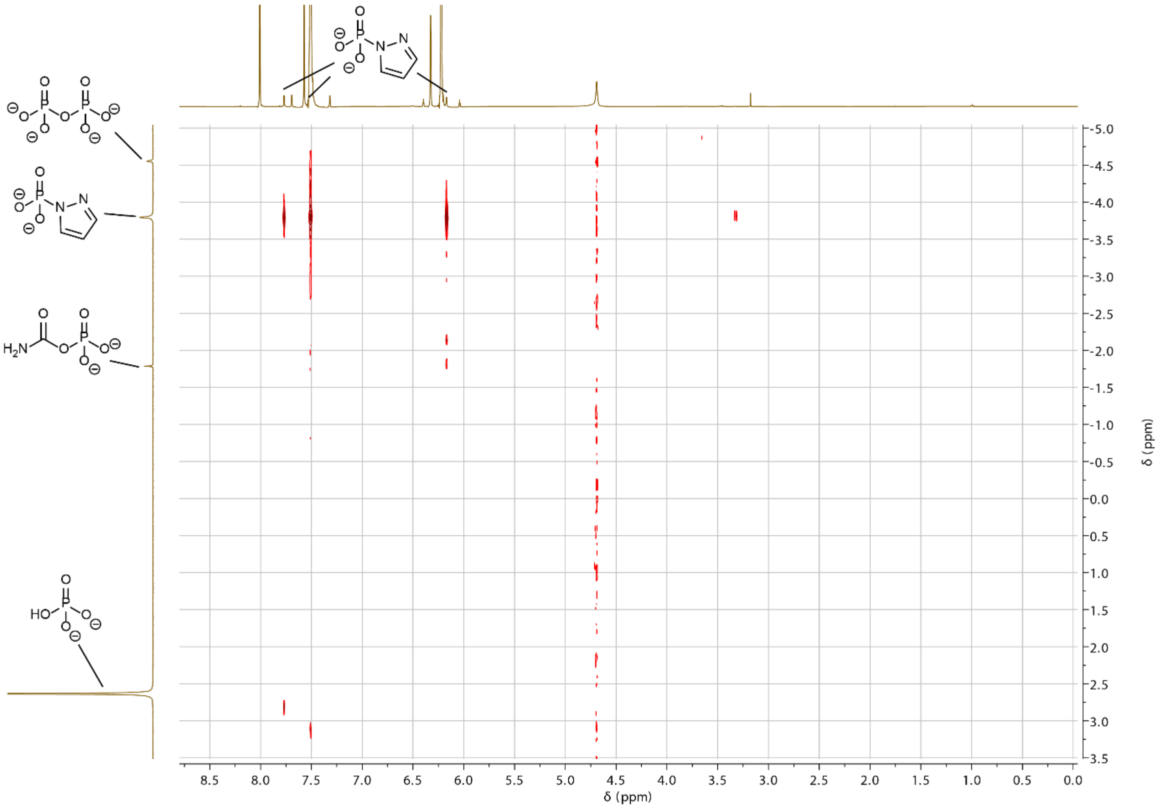

Supplementary Figure 26: ^{1}H ^{31}P-HMBC NMR-spectrum for phosphorylated pyrazole in a 150 mM lithium carbamoyl phosphate, 300 mM potassium cyanate and 1.0 M pyrazole solution. x axis = ^{1}H NMR and y axis = ^{31}P NMR.

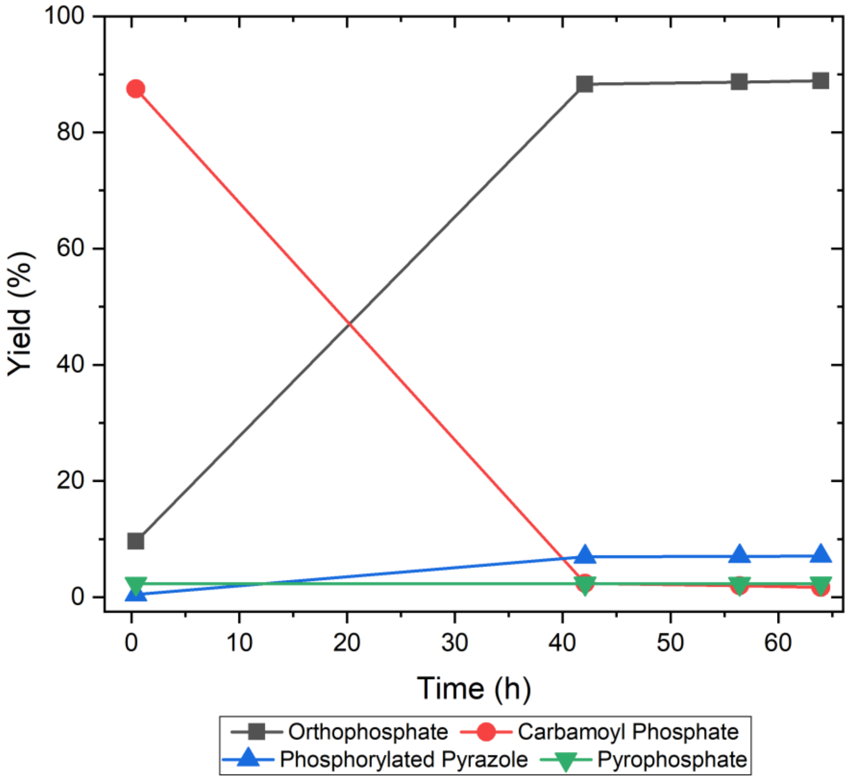

Supplementary Figure 27: Changes in yield over time for the formation phosphorylated pyrazole in a 150 mM lithium carbamoyl phosphate, 300 mM potassium cyanate and 1.0 M pyrazole solution.

S3 Synthetic formation of calcium imidazole phosphate

Calcium imidazole phosphate was prepared synthetically first by formation of diphosphoimidazole followed by hydrolysis.

S3.1 Synthesis of calcium diphosphoimidazole

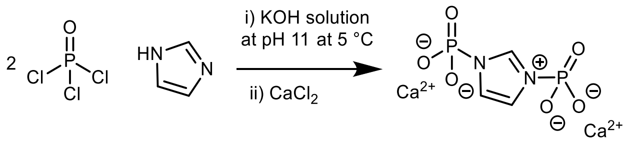

A procedure adapted from Rosenberg was used to prepare calcium diphosphoimidazole.[1]

A 500 mL three-necked round bottom flask was charged with 5.05 g (0.073 mol) of imidazole and a stirrer bar. 100 mL of MilliQ water was added to dissolve the imidazole to give a 730 mM solution. The pH of the solution was adjusted to pH 11 using a 5 M KOH solution. The imidazole solution was then cooled to 5 °C in a refrigerator. After cooling the imidazole solution was placed in an ice-bath on a stirring plate and a dropping funnel was attached and then filled with 28 ml (0.3 mol) of phosphor(V)oxychloride. The phosphor(V)oxychloride was added dropwise to the stirred imidazole solution over a period of 5.25 h while maintaining the temperature between 5 – 15 °C. The strongly exothermic reaction between phosphor(V)oxychloride and water required periods to cool the solution. After each addition of phosphor(V)oxychloride the pH of the solution was checked using pH paper and maintained at pH 11 with a 5 M KOH solution (the 5 M KOH solution was kept on an ice-bath to minimise temperature rises). After the final addition of phosphor(V)oxychloride the solution was allowed to warm to room temperature over a period of 50 min whilst the pH was maintained at pH 11 with a 5 M KOH solution.

To isolate the diphosphoimidazole formed required a series of steps. Once at room temperature the pH of the solution was adjusted to pH 9.5 with a 5 M HCl solution. 25.0 g (0.123 mol) of $MgCl_2{\cdot}6H_2O$ was dissolved in 65 mL of MilliQ to give a 1.9 M solution. The $MgCl_2$ solution was added to the reaction solution and stirred for 25 min upon which a white precipitate formed. The pH dropped to pH 7 upon the addition of $MgCl_2$ solution and was raised to pH 8 using a 5 M KOH solution. The resulting precipitate was then removed via vacuum filtration with a Buchner funnel. The filtrate was adjusted to pH 8.

15.1 g (0.136 mol) of $CaCl_2$ was dissolved in 50 mL of MilliQ to give a 2.72 M solution. The $CaCl_2$ solution was added to the filtrate from the previous step upon which a milky white precipitate formed and pH was maintained at pH 8. The solution was filtered twice under vacuum to remove all the precipitate and the filtrate was again adjusted to pH 8.

The filtrate volume was now approximately 600 mL and 300 mL of ethanol (1/2 volume of filtrate) was added to the filtrate and then it was placed in the refrigerator for 2 h. After 2 h, a fine white precipitate had formed which was filtered off under vacuum. The precipitate was washed twice with 25 mL of a 25 : 75 MilliQ : ethanol solution, washed twice with 25 mL of a 50 : 50 MilliQ : ethanol solution and washed twice with 25 mL of ethanol. The precipitate was then collected and dried on a high vacuum line overnight. The resulting solid calcium diphosphoimidazole had a mass of 31.3 g (quantitative yield). ^{31}P NMR (162.0 MHz, 9 : 1 H_2O : D_2O): *diphosphoimidazole* δ (ppm) = - 4.41 (s, 2P). ^{1}H NMR (400.2 MHz, 9 : 1 H_2O : D_2O): *diphosphoimidazole* δ (ppm) = 8.3 (s, 1H), 7.36 (d, *J* = 1.6 Hz, 2H). ^{13}C NMR (125.8 MHz, 9 : 1 H_2O : D_2O): *diphosphoimidazole* δ (ppm) = 137 (s, 1C), 135 (s, 1C), 122 (s, 1C).

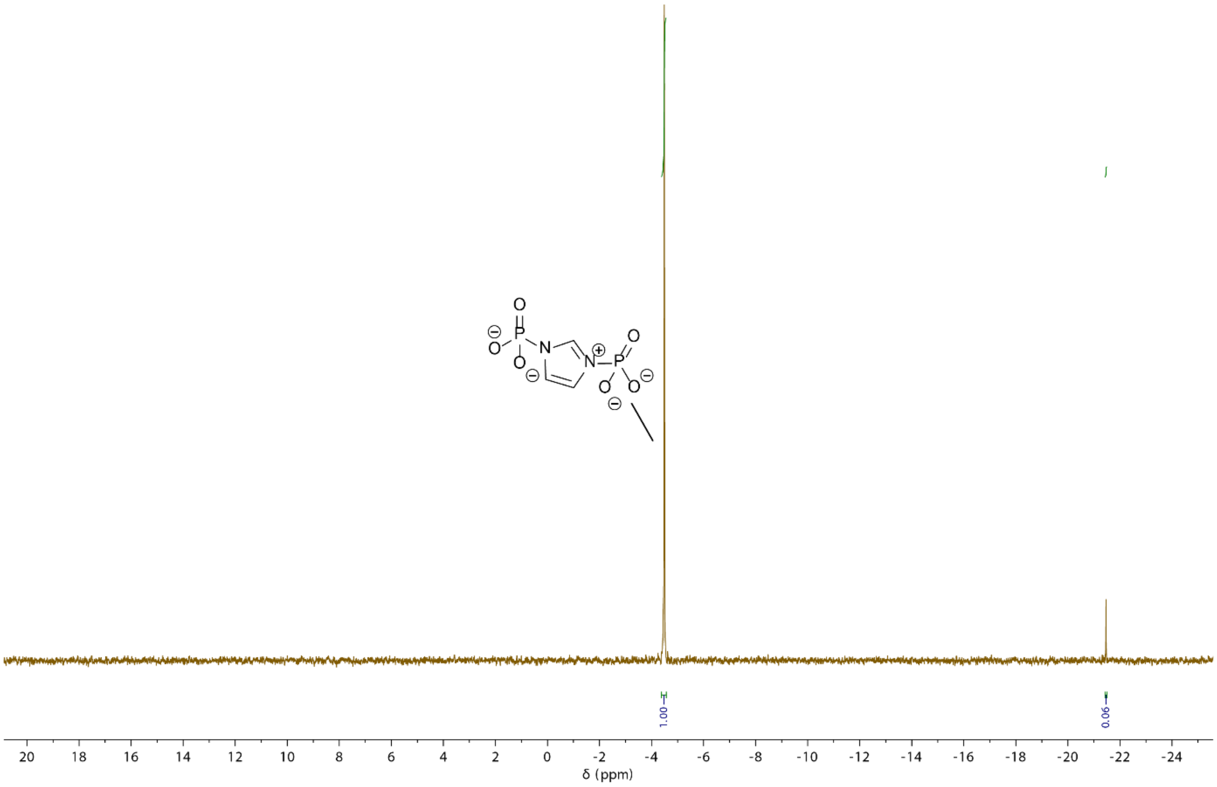

Supplementary Figure 28: ^{31}P NMR spectrum of calcium diphosphoimidazole in D_2O.

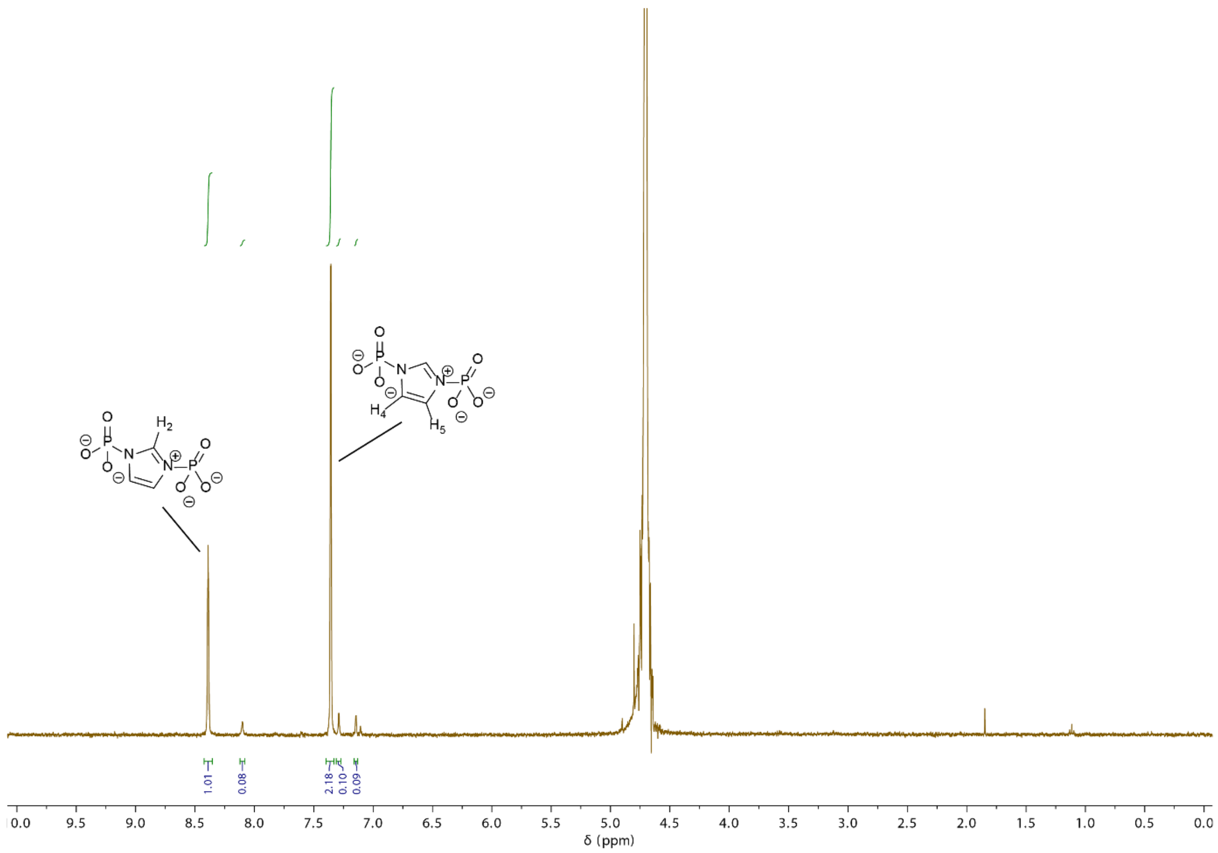

Supplementary Figure 29: ^{1}H NMR spectrum of calcium diphosphoimidazole in D_2O.

S3.2 Synthesis of calcium imidazole phosphate

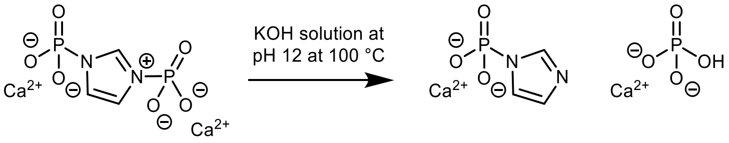

A procedure adapted from Cooperman was used to prepare calcium imidazole phosphate.[2]

A 500 mL three-necked round bottom flask was filled with 402 mL of a KOH solution at pH 12.5 and heated to reflux in an oil bath. Once refluxing, 6.11 g (0.020 mol) of calcium diphosphoimidazole was added to the solution. The pH was maintained between pH 12.0-12.5 using 5 M KOH. The solution was refluxed for 30 min, removed from heating and then cooled to 15 °C on an ice-bath. The white precipitate was removed by filtering under vacuum and the filtrate pH was adjusted to pH 8.5. 2.44 g (0.022 mol) of $CaCl_2$ was dissolved in 10 mL of MilliQ to give a 2.2 M solution. This $CaCl_2$ solution was added to the filtrate upon which a white precipitate formed. The pH was maintained at pH 8.5. The majority of white precipitate was removed under vacuum and a gravity filtration was used to remove the remaining precipitate. The filtrate volume was 413 mL and 1240 mL of ethanol (3-fold volume of filtrate) was added to the filtrate. The solution was put in the refrigerator for 4 h. The resulting fine

white precipitate was filtered off under vacuum and washed with 10 mL of a 75 : 25 ethanol: MilliQ, washed with 10 mL of ethanol and washed with 5 mL of diethyl ether. The white solid was collected and dried on the vacuum line overnight. The resulting solid calcium imidazole phosphate had a mass of 1.09 g (0.006 mol, Yield 29 %). ^{31}P NMR (202.5 MHz, 9 : 1 H_2O : D_2O): *imidazole phosphate* δ (ppm) = - 4.51 (s, 1P). ^{1}H NMR (500.1 MHz, 9 : 1 H_2O : D_2O): *imidazole phosphate* δ (ppm) = 8.05 (s, 1H), 7.27 (d, *J* = 1.0 Hz, 1H), 7.13 (d, *J* = 1.0 Hz, 1H). ^{13}C NMR (125.8 MHz, 9 : 1 H_2O : D_2O): *imidazole phosphate* δ (ppm) = 136 (s, 1C), 122 (s, 1C), 121 (s, 1C).

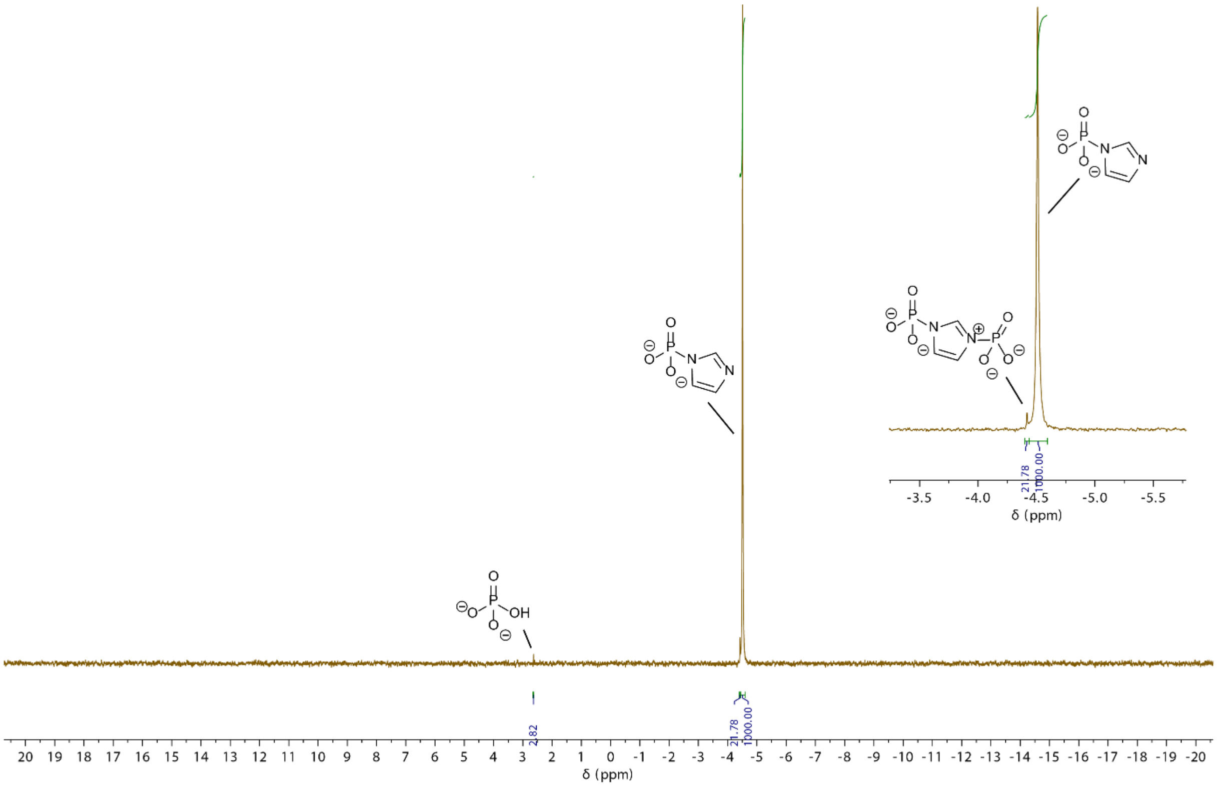

Supplementary Figure 30: ^{31}P NMR spectrum of calcium imidazole phosphate in 0.5 M citric acid buffer pH 6.85 9 : 1 H_2O : D_2O. Insert zooms in on the imidazole phosphate peak.

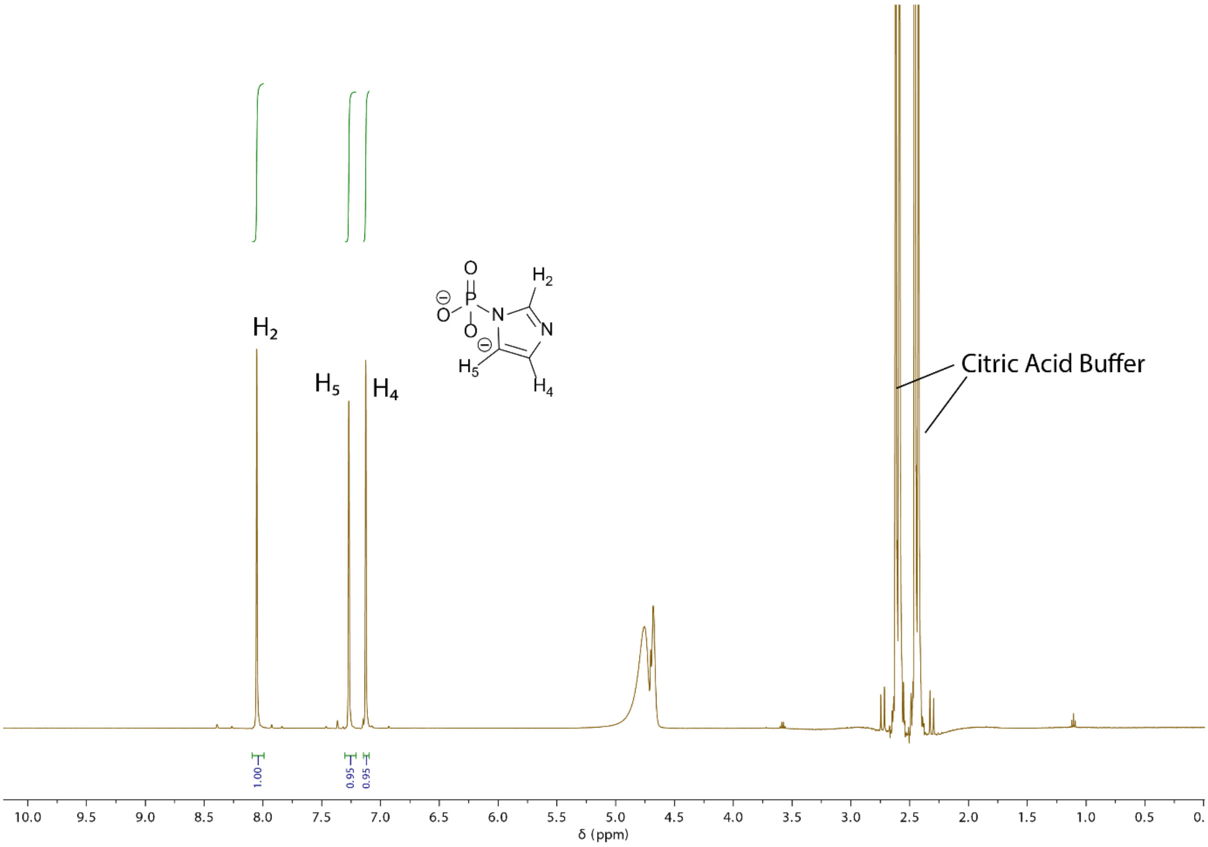

Supplementary Figure 31:^{1}H NMR spectrum of calcium imidazole phosphate in 0.5 M citric acid buffer pH 6.85 9 : 1 H_2O : D_2O.

S4 Reactions of imidazole phosphate in aqueous solution

S4.1 Summary of reactions for which no phosphorylation of nucleophile was observed in solution

Supplementary Table 1: Summary of reactions tried to see if phosphate transfer occurred in solution between imidazole phosphate and various nucleophiles.

Compound	[Nucleophile] (mM)	[sodium imidazole phosphate] (mM)	Outcome
Adenosine	10	10	No phosphorylation of nucleophile observed
Guanosine	20	10	No phosphorylation of nucleophile observed
Adenosine-5-monophoshate	10	10	No phosphorylation of nucleophile observed
Guanosine-5-monophoshate	10	10	No phosphorylation of nucleophile observed
Glucose	100	100	No phosphorylation of nucleophile observed
Ribose	100	100	No phosphorylation of nucleophile observed
Ethanol	100	100	No phosphorylation of nucleophile observed
Ethanolamine	100	100	No phosphorylation of nucleophile observed
Serine methyl ester	100	100	No phosphorylation of nucleophile observed
AcSerOMe	50	100	No phosphorylation of nucleophile observed
AcThrOMe	50	100	No phosphorylation of nucleophile observed

S4.2 Reaction with N-methyl hydroxylamine

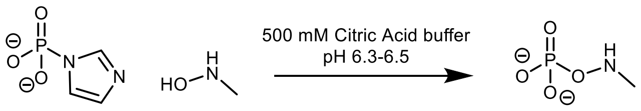

21.0 mg (0.25 mmol) of N-methylhydroxylamine·HCl was dissolved in 2.5 mL of 0.5 M citric acid buffer in 9 : 1 H_2O : D_2O at pH 6.50 to give a 100 mM N-methylhydroxylamine solution. 600 µL of this solution was used to dissolve 11.52 mg (0.06 mmol) of sodium imidazole phosphate to give a 100 mM sodium imidazole phosphate solution. The pH of the solution was again corrected to pH 6.50 with volumetric 2 M NaOH and volumetric 2 M HCl. The reaction solution was placed in a sealed NMR tube, kept at 22 °C and followed over time using ^{31}P and ^{1}H NMR spectroscopy. Periodic pH measurements were also taken. An HMPA internal standard was also added in a 10 mM concentration.

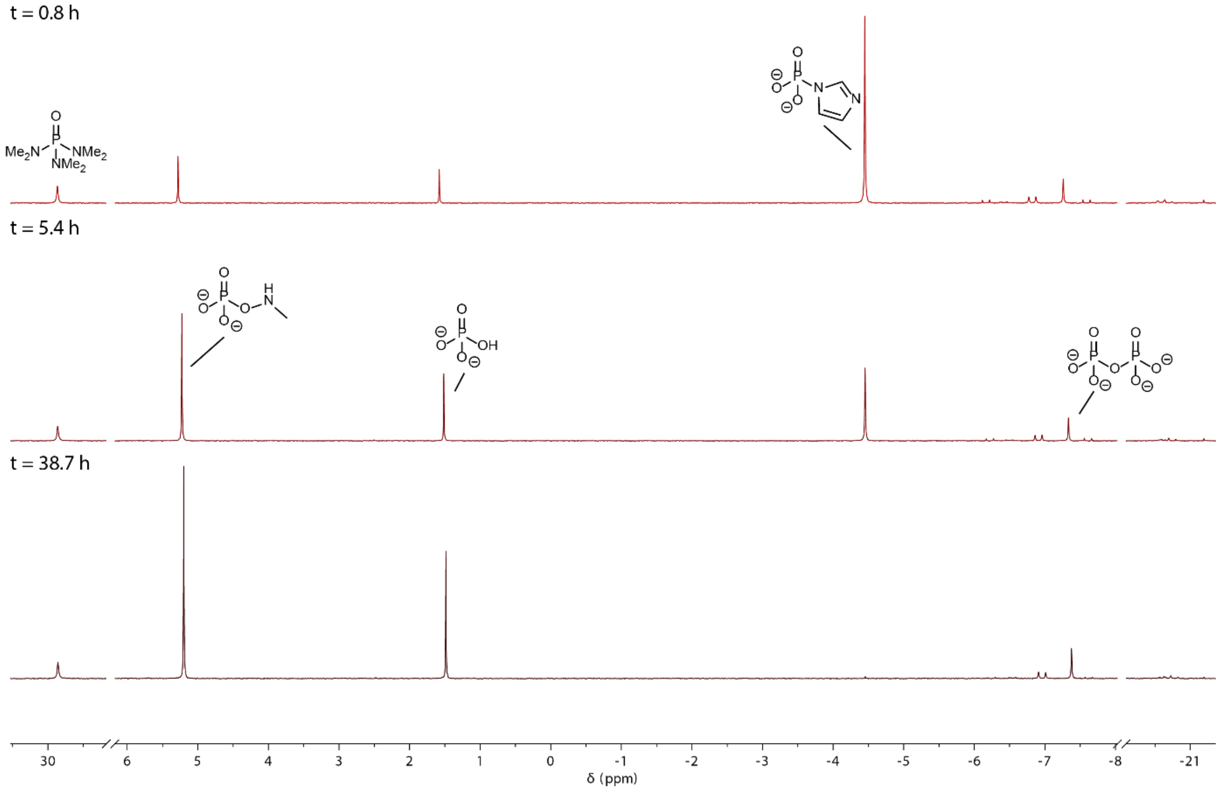

Supplementary Figure 32: Representative ^{31}P NMR spectra over time for 100 mM sodium imidazole phosphate + 100 mM N-methylhydroxylamine in 0.5 M citric acid buffer 9 : 1 H_2O : D_2O at pH 6.48 and 22 °C. An internal standard HMPA (δ = 29.85 ppm) was included.

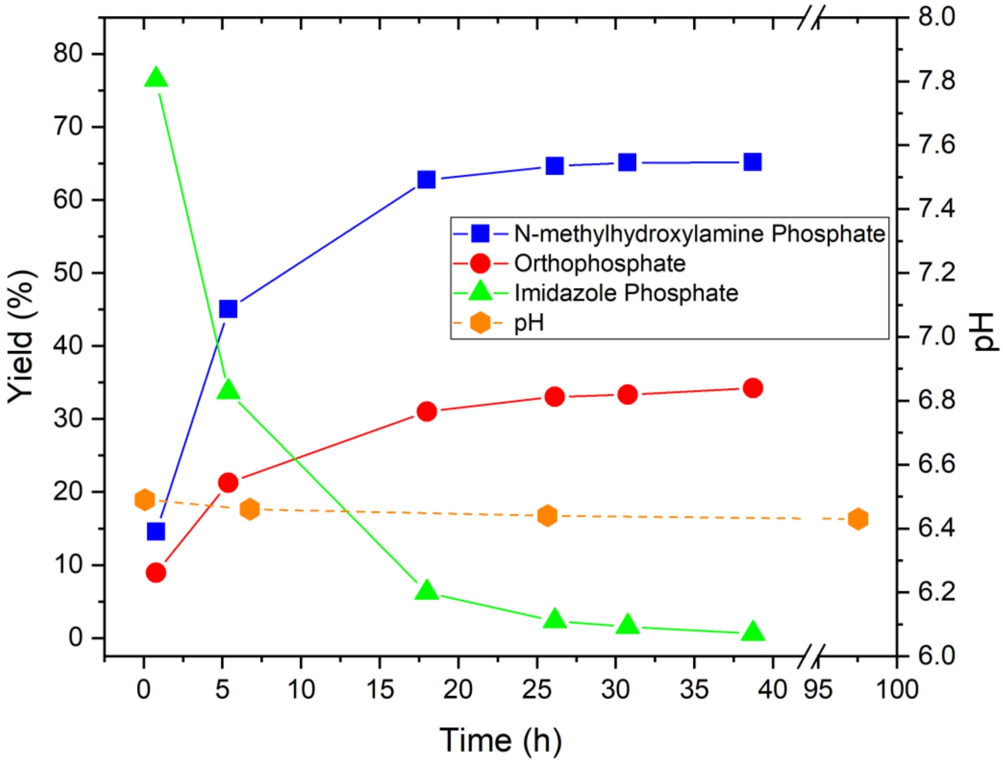

Supplementary Figure 33: Changes in yield over time for the reaction of 100 mM sodium imidazole phosphate + 100 mM N-methylhydroxylamine in 0.5 M citric acid buffer 9 : 1 H_2O : D_2O at pH 6.48 and 22 °C.

S4.3 Reaction with N-iso-propyl hydroxylamine

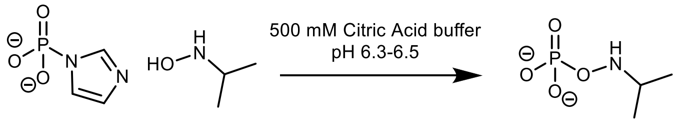

28.0 mg (0.25 mmol) of N-isopropylhydroxylamine·HCl was dissolved in 2.5 mL of 0.5 M citric acid buffer in 9 : 1 H_2O : D_2O at pH 6.50 to give a 100 mM N-isopropylhydroxylamine solution. 600 µL of this solution was used to dissolve 11.52 mg (0.06 mmol) of sodium imidazole phosphate to give a 100 mM sodium imidazole phosphate solution. The pH of the solution was adjusted to pH 6.50 with volumetric 2 M NaOH and volumetric 2 M HCl. The reaction solution was placed in a sealed NMR tube, kept at 22 °C and followed over time using ^{31}P and ^{1}H NMR spectroscopy. Periodic pH measurements were also taken. An HMPA internal standard was also added in a 10 mM concentration.

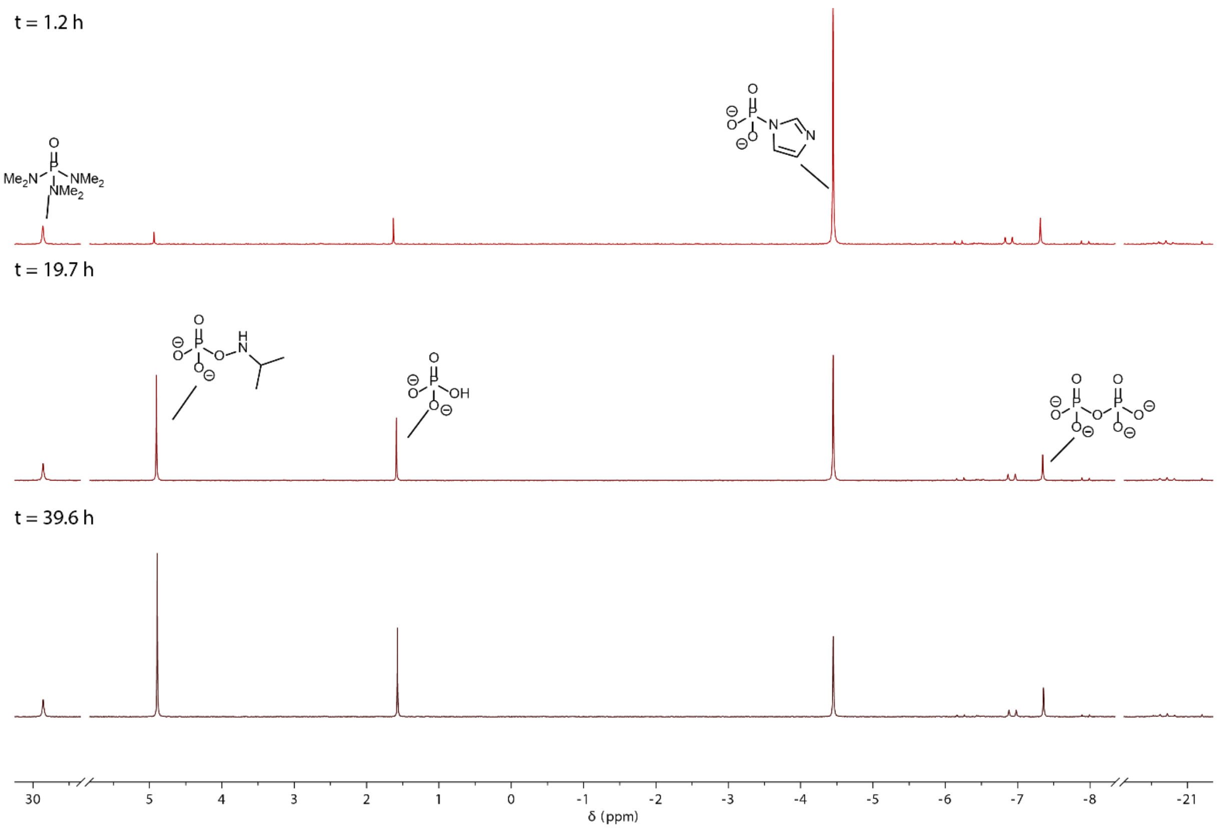

Supplementary Figure 34: Representative ^{31}P NMR spectra over time for 100 mM sodium imidazole phosphate + 100 mM N-Isopropylhydroxylamine in 0.5 M citric acid buffer 9 : 1 H_2O : D_2O at pH 6.48 and 22 °C. An internal standard HMPA (δ = 29.85 ppm) was included.

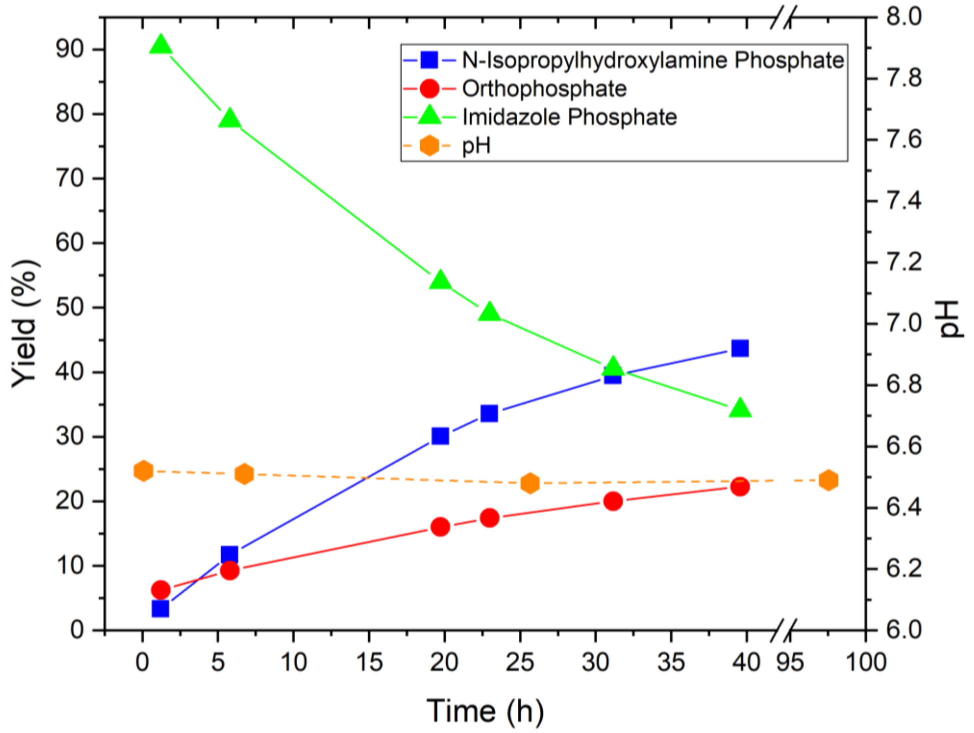

Supplementary Figure 35: Changes in yield over time for the reaction of 100 mM sodium imidazole phosphate + 100 mM N-Isopropylhydroxylamine in 0.5 M citric acid buffer 9 : 1 H_2O : D_2O at pH 6.48, 22 °C.

S5 Phosphorylation of prebiotically important organic compounds using imidazole phosphate in a paste

S5.1 Supplementary experimental method for phosphorylating in a paste

48.5 mg (0.26 mmol) of calcium imidazole phosphate and 39.1 mg (0.26 mmol) of D-ribose were added together in a 2.5 mL Eppendorf. The solids were made into a fine powder by crushing and stirring with a spatula. The solid powders were further mixed on a vortexer for 15 s. 9 µL of D_2O (1 µL/mg) was added to the powder to form a paste. The Eppendorf was then spun on a centrifuge for 5 min at 5590 g in order to move all solid to the bottom of the Eppendorf and compact it together. The Eppendorf was then placed on a thermoshaker at either 50 °C or 80 °C and 600 rpm for up 165 h.

The reaction was followed with ^{31}P-NMR and ^{1}H-NMR and ^{1}H ^{31}P HMBC NMR spectroscopy. A sample of approximately 10 mg was removed periodically and dissolved in 0.5 mL of 0.5 M citric acid buffer at pH 6.85 9 : 1 H_2O : D_2O. NMR spectra were then obtained using this solution.

Reactions with other nucleophiles were conducted using an identical procedure unless otherwise specified.

S5.2 Phosphorylation of Amphiphiles

S5.2.1 Phosphorylation of glycerol

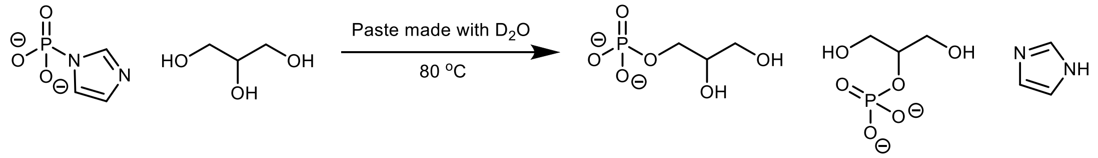

This reaction was performed according to the procedure in Section S5.1 but with 24. 2 mg (0.13 mmol) of calcium imidazole phosphate and with the ribose replaced by 60.0 mg (0.65 mmol) glycerol.

Formation of glycerol-1-phosphate and glycerol-2-phosphate occurred in the paste from the reaction between imidazole phosphate and glycerol with the highest observed yield of 29.1 % glycerol-1-phosphate and 23.0 % glycerol-2-phosphate after heating the paste at 80 °C for 167.3 h (Supplementary Figure 41 and 42).

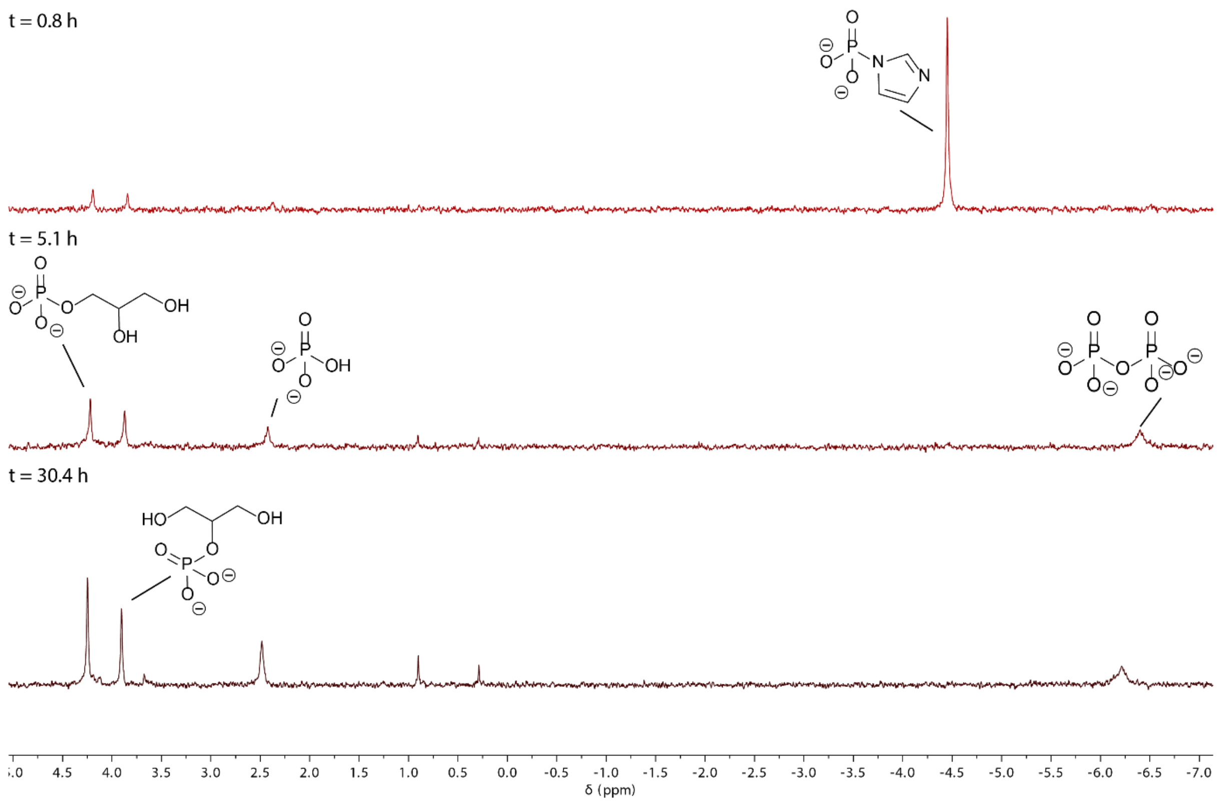

Supplementary Figure 41: Representative ^{31}P NMR spectra over time for the reaction of 0.13 mmol calcium imidazole phosphate + 0.13 mmol glycerol in paste conditions at 80 °C.

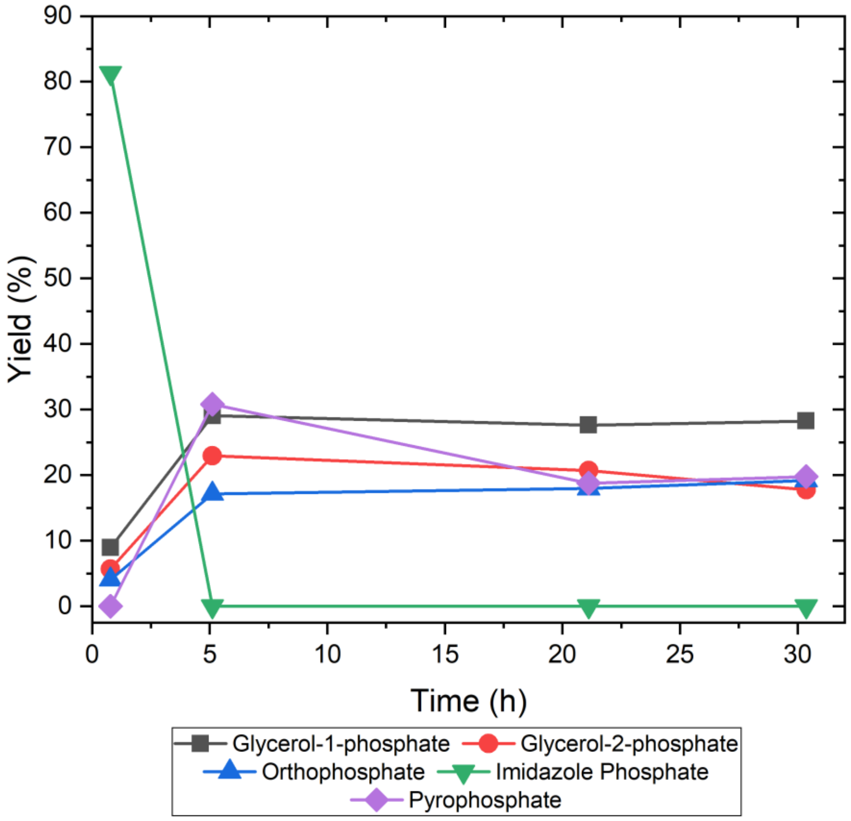

Supplementary Figure 42: Changes in yield over time for the reaction of 0.26 mmol calcium imidazole phosphate + 0.26 mmol glycerol in paste conditions at 80 °C.

S5.2.2 Phosphorylation of Pentane-1,2-diol

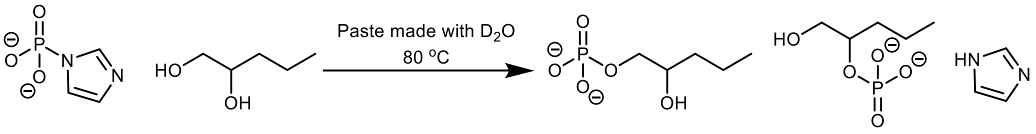

This reaction was performed according to the procedure in Section S5.1 but with 24. 2 mg (0.13 mmol) of calcium imidazole phosphate and with the ribose replaced by 67.7 mg (0.65 mmol) Pentane-1,2-diol.

Formation of Pentane-1,2-diol-1-phosphate and Pentane-1,2-diol-2-phosphate occurred in the paste from the reaction between imidazole phosphate and Pentane-1,2-diol with a yield of 34.4 % Pentane-1,2-diol-1-phosphate and 13.5 % Pentane-1,2-diol-2-phosphate after heating the paste at 80 °C for 54.2 h (Supplementary Figure 43 and 44).

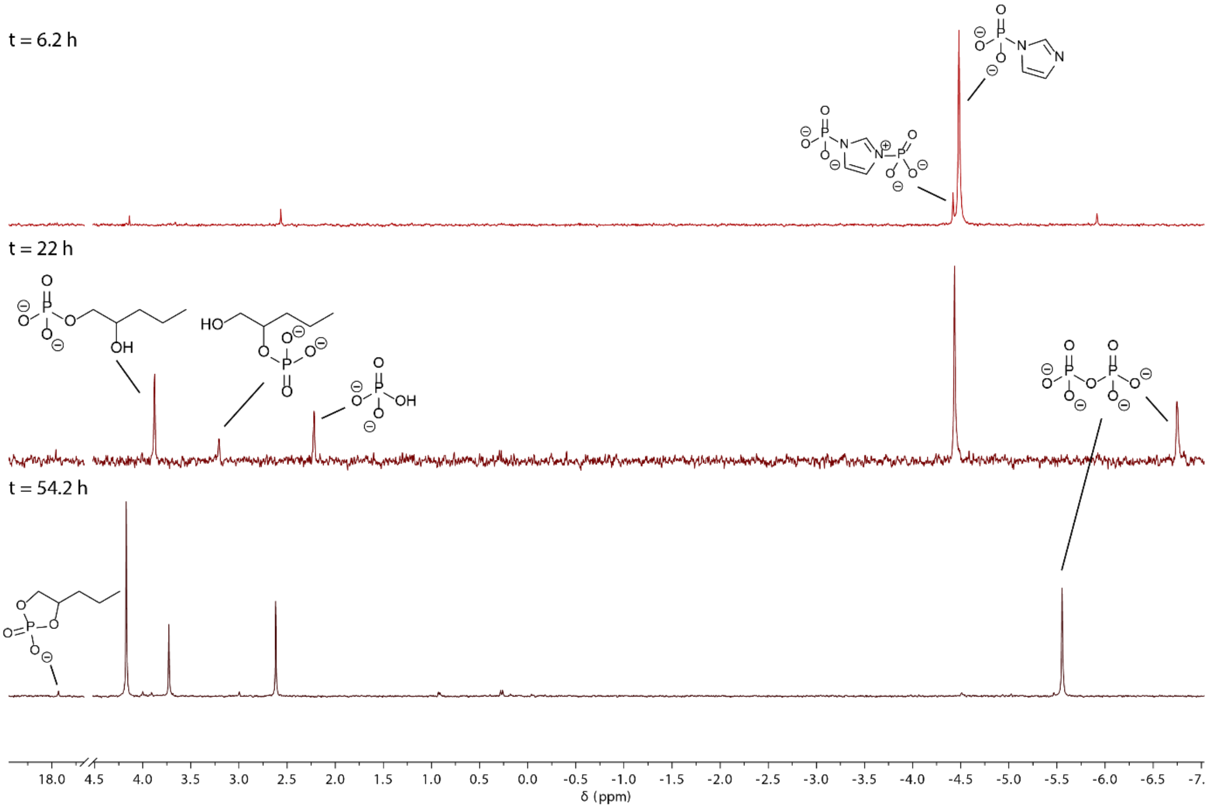

Supplementary Figure 43: Representative ^{31}P NMR spectra over time for the reaction of 0.13 mmol calcium imidazole phosphate + 0.65 mmol Pentane-1,2-diol in paste conditions at 80 °C. All NMR spectra were measured in a 0.5 M citric acid buffer at pH 6.85 9 : 1 H_2O : D_2O. The chemical shift of the pyrophosphate peak is variable at pH 6.85 due to the pyrophosphate pK_a = 6.6.

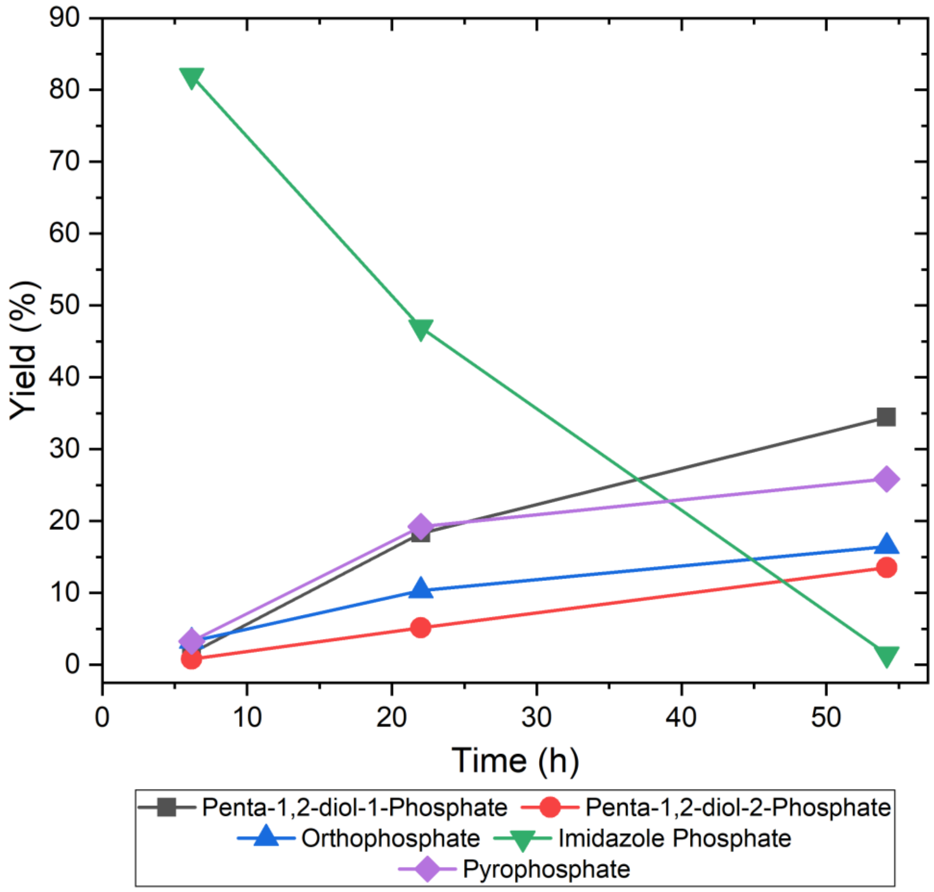

Supplementary Figure 44: Changes in yield over time for the reaction of 0.13 mmol calcium imidazole phosphate + 0.65 mmol Pentane-1,2-diol in paste conditions at 80 °C.

S5.3 Phosphorylation of Amino Acids

S5.3.1 Phosphorylation of serine methyl ester

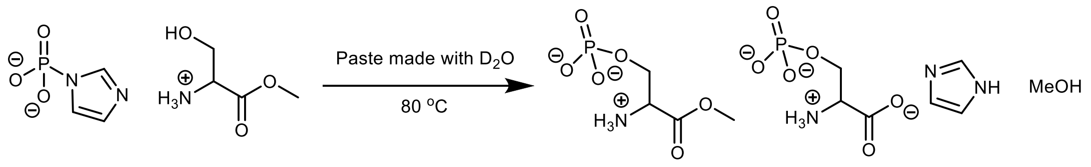

This reaction was performed according to the procedure in Section S5.1 but with the ribose replaced by 40.5 mg (0.26 mmol) serine methyl ester·HCl.

Formation of O-phosphorylated serine methyl ester and O-phosphorylated serine occurred in the paste from the reaction between imidazole phosphate and serine methyl ester / hydrolysed serine methyl ester with the highest observed yield of 1.0 % O-phosphorylated serine methyl ester and 4.0 % O-phosphorylated serine after heating paste at 80 °C for 20.5 h (Supplementary Figure 45 and 46).

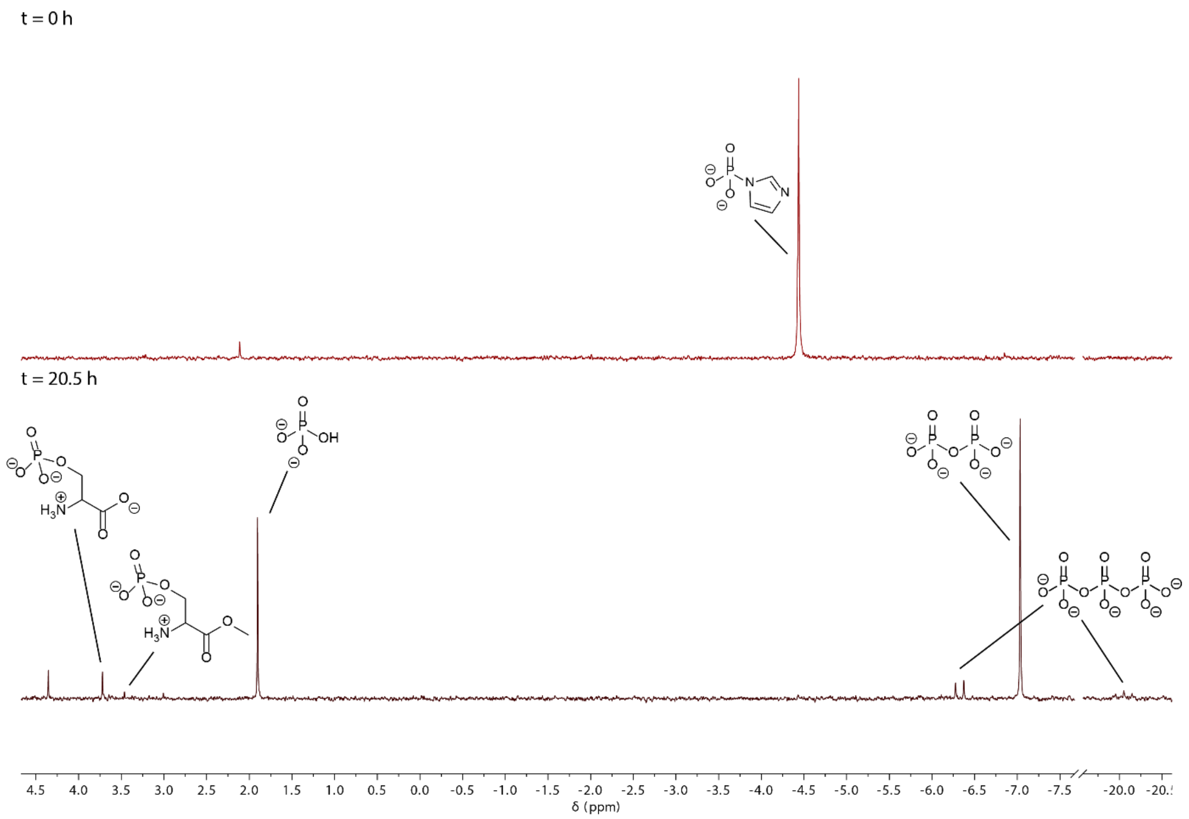

Supplementary Figure 45: Representative ^{31}P NMR spectra over time for the reaction of 0.26 mmol calcium imidazole phosphate + 0.26 mmol serine methyl ester in paste conditions at 80 °C.

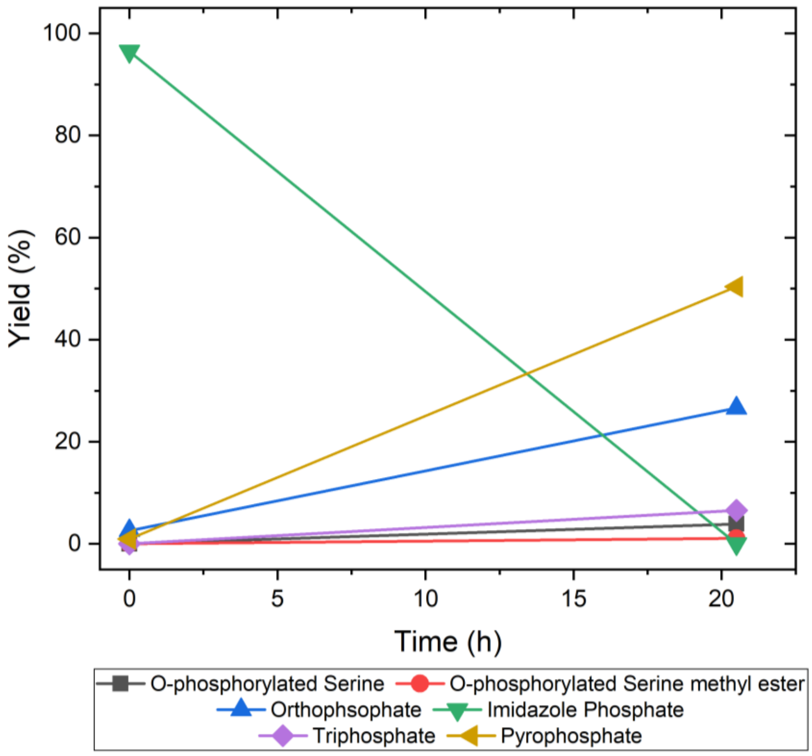

Supplementary Figure 46: Changes in yield over time for the reaction of 0.26 mmol calcium imidazole phosphate + 0.26 mmol serine methyl ester in paste conditions at 80 °C.

S5.4 Phosphorylation of Metabolites

S5.4.1 Phosphorylation of sodium glycolate

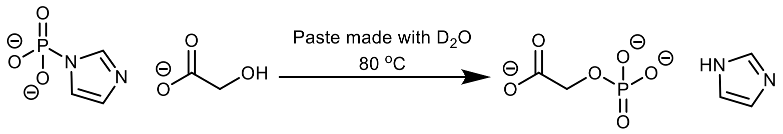

This reaction was performed according to the procedure in Section S5.1 but with 24. 2 mg (0.13 mmol) of calcium imidazole phosphate and with the ribose replaced by 49.4 mg (0.65 mmol) glycolic acid and the addition of sodium hydroxide to neutralise the solution.

Formation of glycolate-2-phosphate occurred in the paste from the reaction between imidazole phosphate and glycolate with the highest observed yield of 15.0 % glycolate-2-phosphate after heating the paste at 80 °C for 54.2 h (Supplementary Figure 47 and 48).

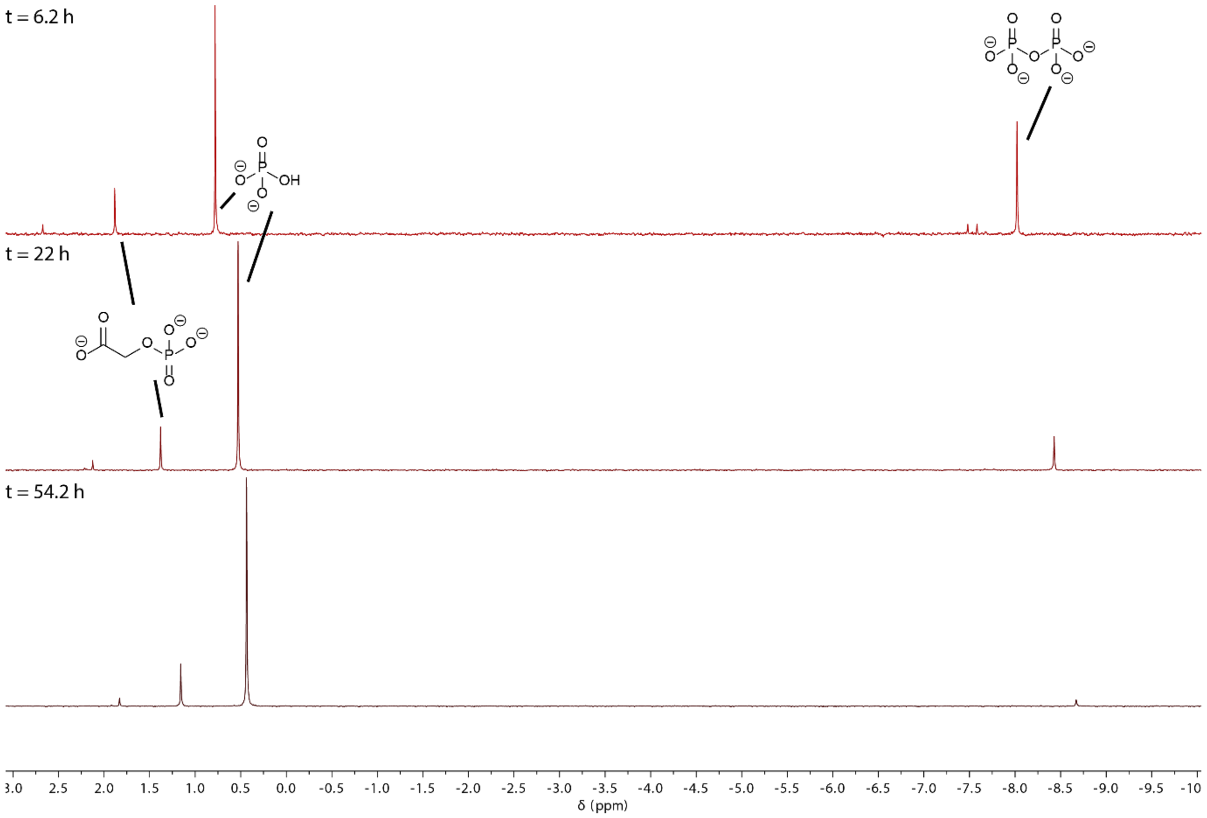

Supplementary Figure 47: Representative ^{31}P NMR spectra over time for the reaction of 0.13 mmol calcium imidazole phosphate + 0.65 mmol sodium glycolate in paste conditions at 80 °C. Note that all imidazole phosphate was consumed before the first spectrum was taken. All NMR spectra were measured in a 0.5 M citric acid buffer at pH 6.85 9 : 1 H_2O : D_2O. The chemical shift of the pyrophosphate peak is variable at pH 6.85 due to the pyrophosphate pK_a = 6.6.

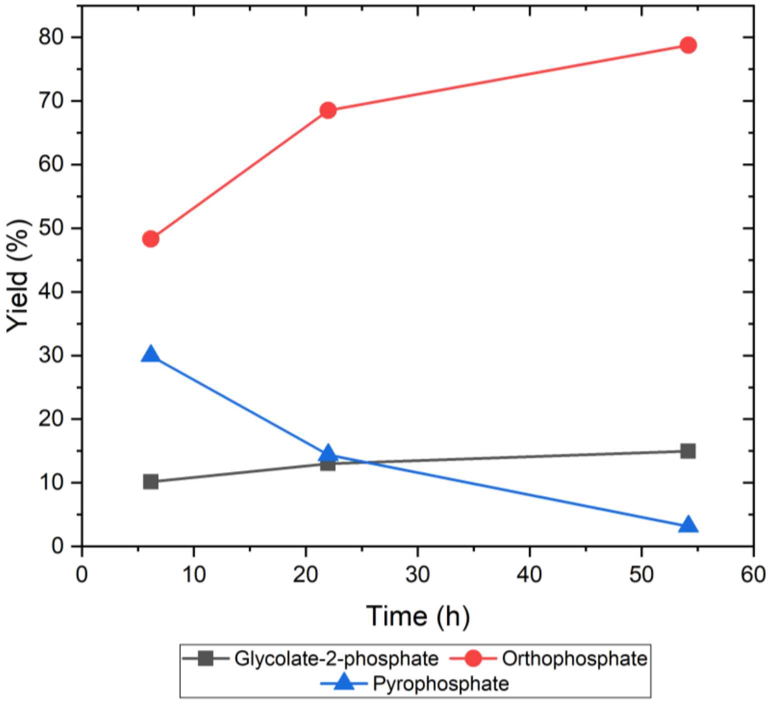

Supplementary Figure 48: Changes in yield over time for the reaction of 0.13 mmol calcium imidazole phosphate + 0.65 mmol sodium glycolate in paste conditions at 80 °C.

S5.4.2 Phosphorylation of sodium lactate

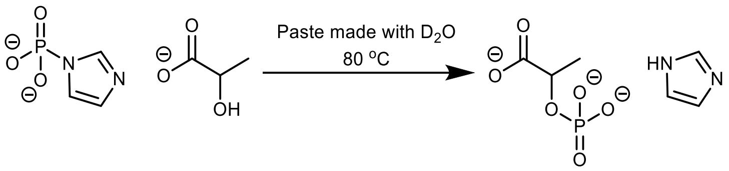

This reaction was performed according to the procedure in Section S5.1 but with 24. 2 mg (0.13 mmol) of calcium imidazole phosphate and with the ribose replaced by 72.8 mg (0.65 mmol) sodium lactate.

Formation of lactate-2-phosphate occurred in the paste from the reaction between imidazole phosphate and lactate with the highest observed yield of 11.0 % lactate-2-phosphate after heating the paste at 80 °C for 6.2 h (Supplementary Figure 49 and 50).

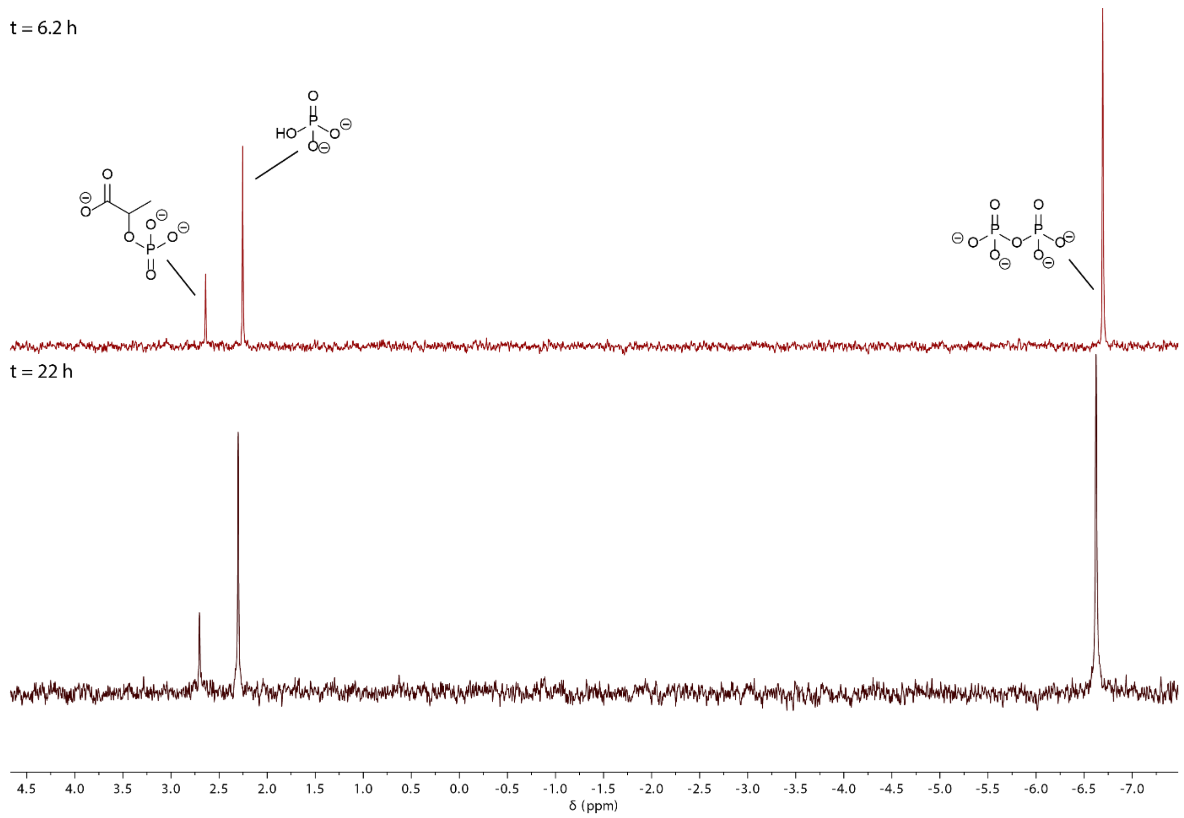

Supplementary Figure 49: Representative ^{31}P NMR spectra over time for the reaction of 0.13 mmol calcium imidazole phosphate + 0.65 mmol sodium lactate in paste conditions at 80 °C. Note that all imidazole phosphate was consumed before the first spectrum was taken.

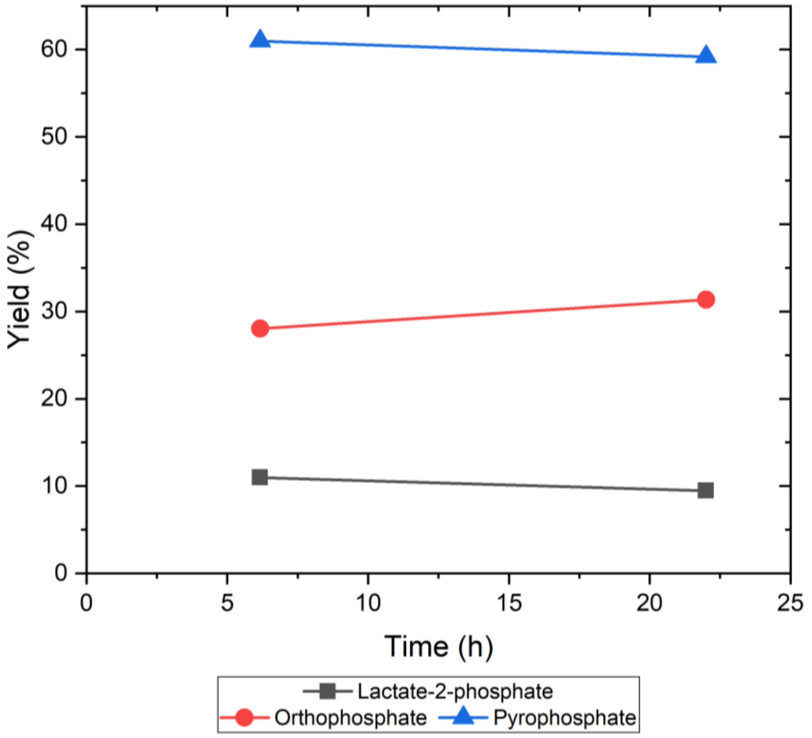

Supplementary Figure 50: Changes in yield over time for the reaction of 0.13 mmol calcium imidazole phosphate + 0.65 mmol sodium lactate in paste conditions at 80 °C.

S5.4.3 Phosphorylation of calcium glycerate

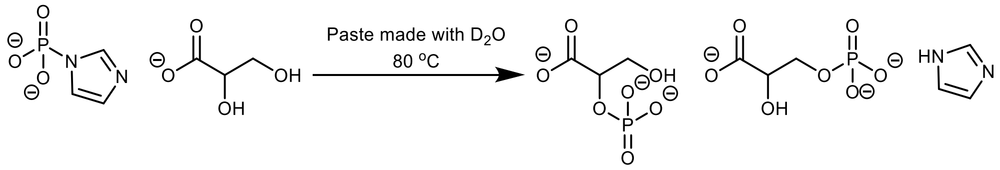

This reaction was performed according to the procedure in Section S5.1 but with 24. 2 mg (0.13 mmol) of calcium imidazole phosphate and with the ribose replaced by 93.2 mg (0.325 mmol) calcium glycerate. There are two glycerate molecules per Ca^{2+} ion and therefore the number of moles of glycerate in the paste is 0.65 mmol.

Formation of glycerate-2-phosphate and glycerate-3-phosphate occurred in the paste from the reaction between imidazole phosphate and glycerate with the highest observed yield of 7.7 % glycerate-2-phosphate and 16.4 % glycerate-3-phosphate after heating the paste at 80 °C for 54.2 h (Supplementary Figure 51 and 52).

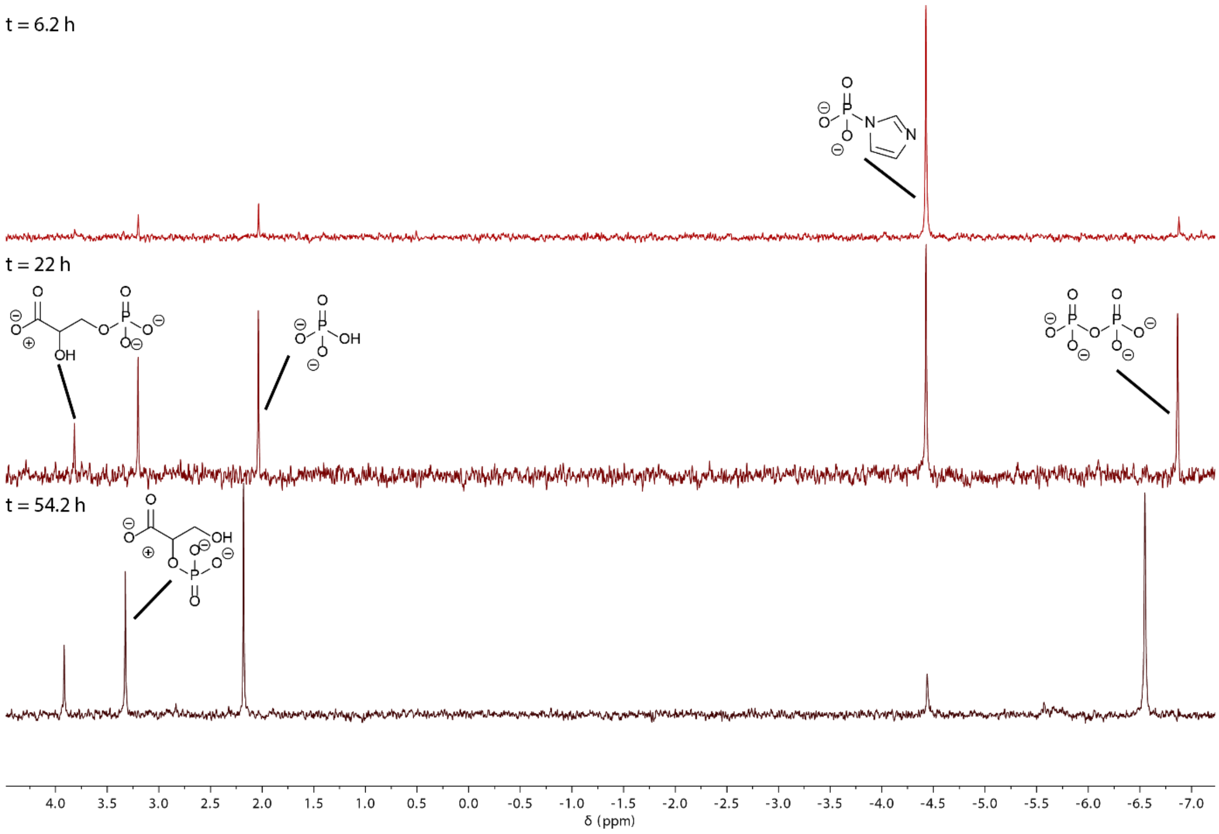

Supplementary Figure 51: Representative ^{31}P NMR spectra over time for the reaction of 0.13 mmol calcium imidazole phosphate + 0.325 mmol calcium glycerate in paste conditions at 80 °C. All NMR spectra were measured in a 0.5 M citric acid buffer at pH 6.85 9 : 1 H_2O : D_2O. The chemical shift of the pyrophosphate peak is variable at pH 6.85 due to the pyrophosphate pK_a = 6.6.

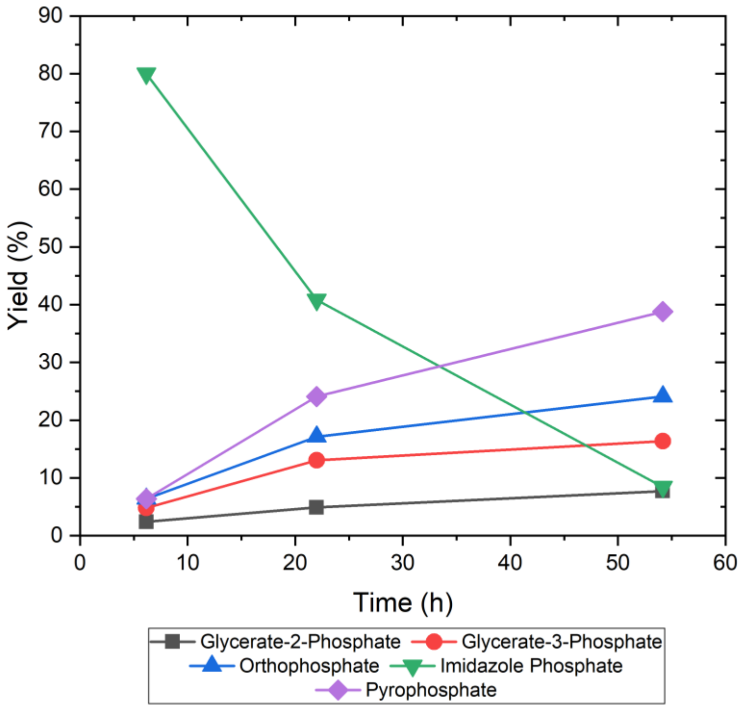

Supplementary Figure 52: Changes in yield over time for the reaction of 0.13 mmol calcium imidazole phosphate + 0.325 mmol calcium glycerate in paste conditions at 80 °C.

S5.5 Phosphorylation of Nucleotides

S5.5.1 Phosphorylation of adenosine 5'-monophosphate

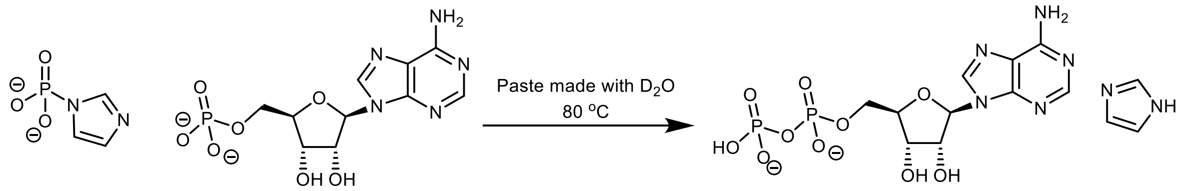

This reaction was performed according to the procedure in Section S5.1 but with 24. 2 mg (0.13 mmol) of calcium imidazole phosphate and with the ribose replaced by 51.1 mg (0.13 mmol) adenosine-5'-monophosphate disodium salt hydrate.

Formation of adenosine-5'-diphosphate occurred in the paste from the reaction between imidazole phosphate and adenosine 5'-monophosphate with the highest observed yield of 9.4 % adenosine-5'-diphosphate after heating the paste at 80 °C for 54.2 h (Supplementary Figure 53 and 54).

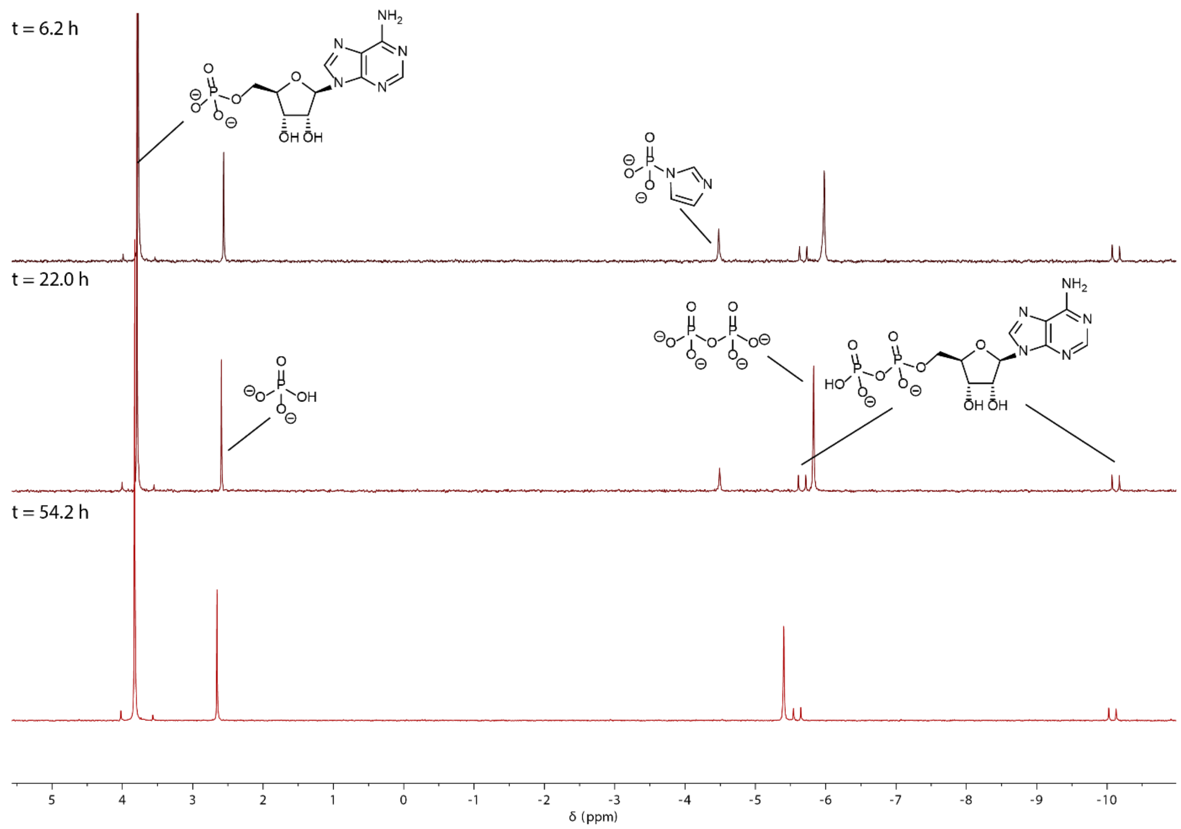

Supplementary Figure 53: Representative ^{31}P NMR spectra over time for the reaction of 0.13 mmol calcium imidazole phosphate + 0.13 mmol adenosine 5′-monophosphate in paste conditions at 80 °C. All NMR spectra were measured in a 0.5 M citric acid buffer at pH 6.85 9 : 1 H_2O : D_2O. The chemical shift of the pyrophosphate peak is variable at pH 6.85 due to the pyrophosphate pK_a = 6.6.

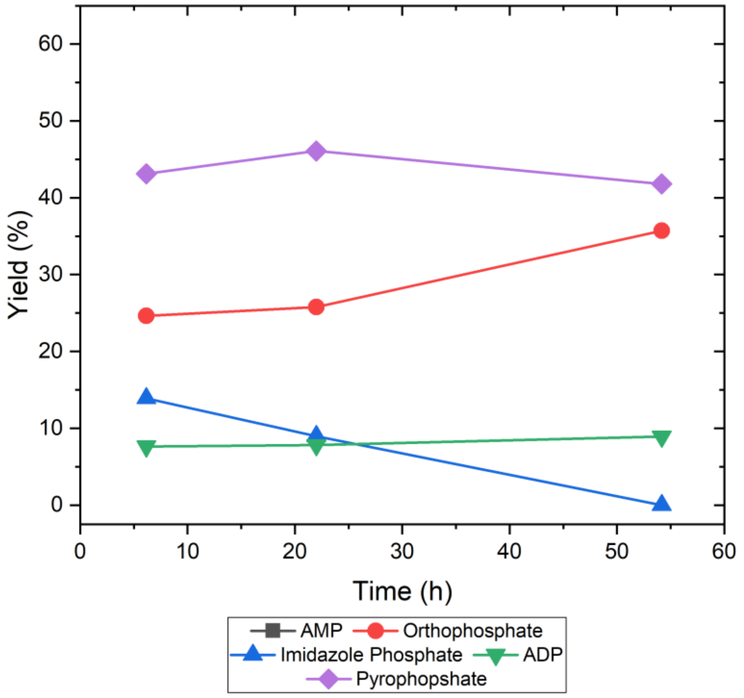

Supplementary Figure 54: Changes in yield over time for the reaction of 0.13 mmol calcium imidazole phosphate + 0.13 mmol adenosine 5′-monophosphate in paste conditions at 80 °C.

S5.5.2 Phosphorylation of cytidine 5'-monophosphate

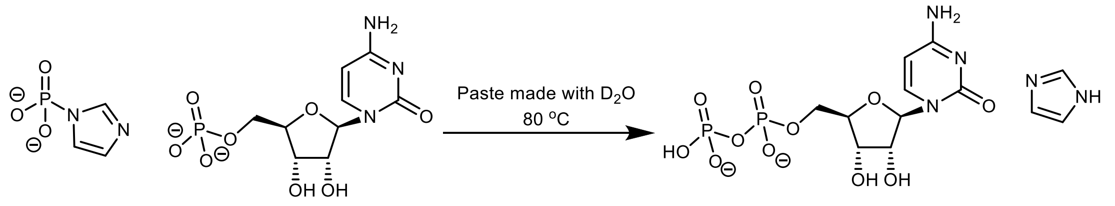

This reaction was performed according to the procedure in Section S5.1 but with the ribose replaced by 42.6 mg (0.13 mmol) cytidine-5'-monophosphate disodium salt.

Formation of cytidine-5'-diphosphate and cytidine-5'-triphosphate occurred in the paste from the reaction between imidazole phosphate and cytidine-5'-monophosphate with the highest observed yield of 11.5 % cytidine-5'-diphosphate and 3.4 % cytidine-5'-triphosphate after heating the paste at 80 °C for 163.5 h (Supplementary Figure 55 and 56).

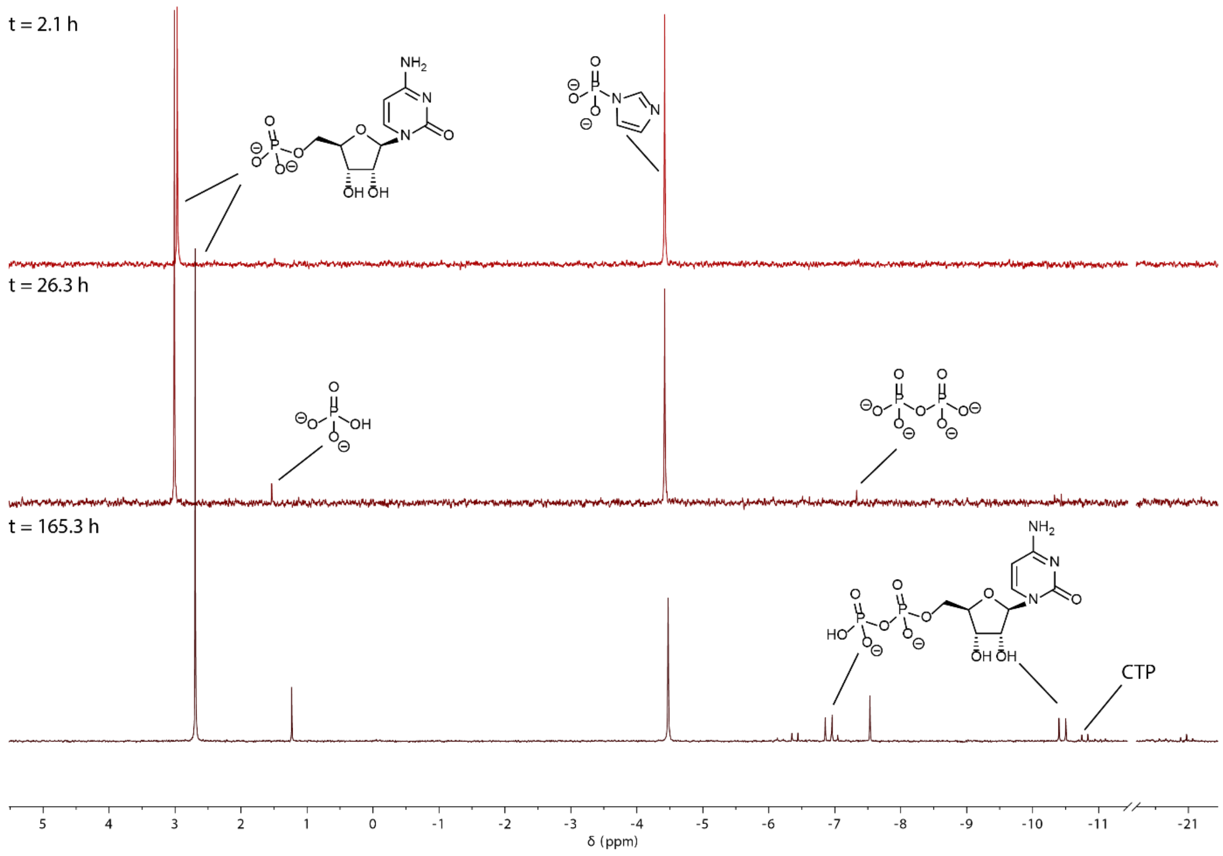

Supplementary Figure 55: Representative ^{31}P NMR spectra over time for the reaction of 0.13 mmol calcium imidazole phosphate + 0.13 mmol cytidine 5'-monophosphate in paste conditions at 80 °C. All NMR spectra were measured in a 0.5 M citric acid buffer at pH 6.85 9 : 1 H_2O : D_2O. The chemical shift of the pyrophosphate peak is variable at pH 6.85 due to the pyrophosphate pK_a = 6.6.

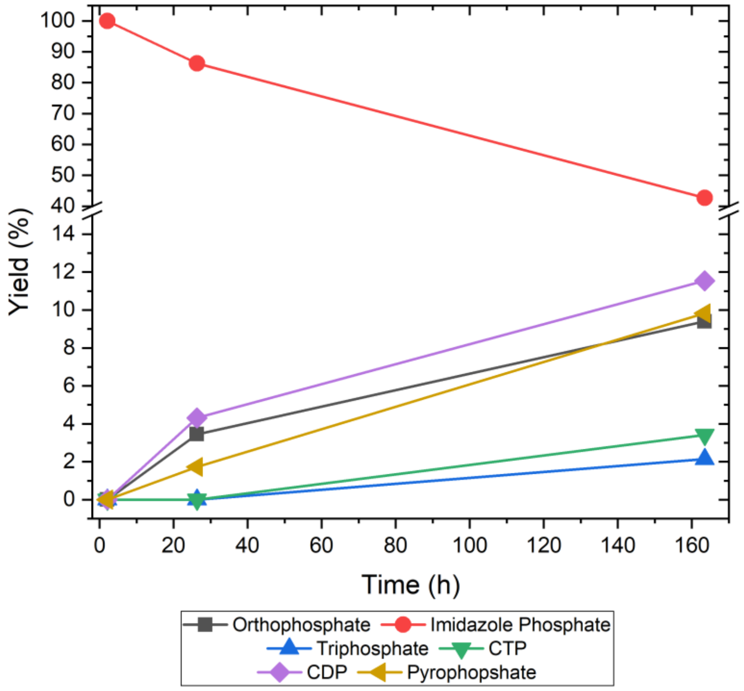

Supplementary Figure 56: Changes in yield over time for the reaction of 0.13 mmol calcium imidazole phosphate + 0.13 mmol cytidine 5'-monophosphate in paste conditions at 80 °C.

S5.5.3 Phosphorylation of guanosine-5'-monophosphate

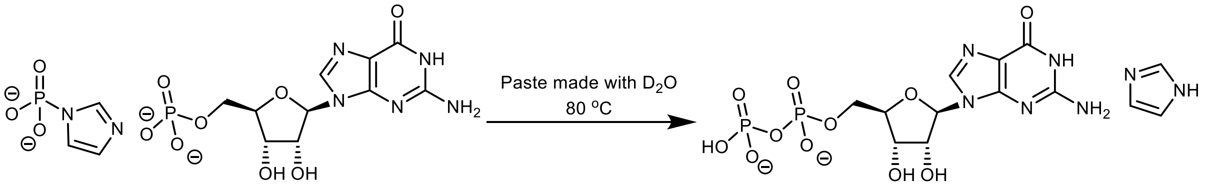

This reaction was performed according to the procedure in Section S5.1 but with 24. 2 mg (0.13 mmol) of calcium imidazole phosphate and with the ribose replaced by 52.2 mg (0.13 mmol) guanosine-5'-monophosphate disodium salt.

Formation of guanosine-5'-diphosphate occurred in the paste from the reaction between imidazole phosphate and guanosine-5'-monophosphate with the highest observed yield of 3.7 % guanosine-5'-diphosphate and after heating the paste at 80 °C for 163.5 h (Supplementary Figure 57 and 58).

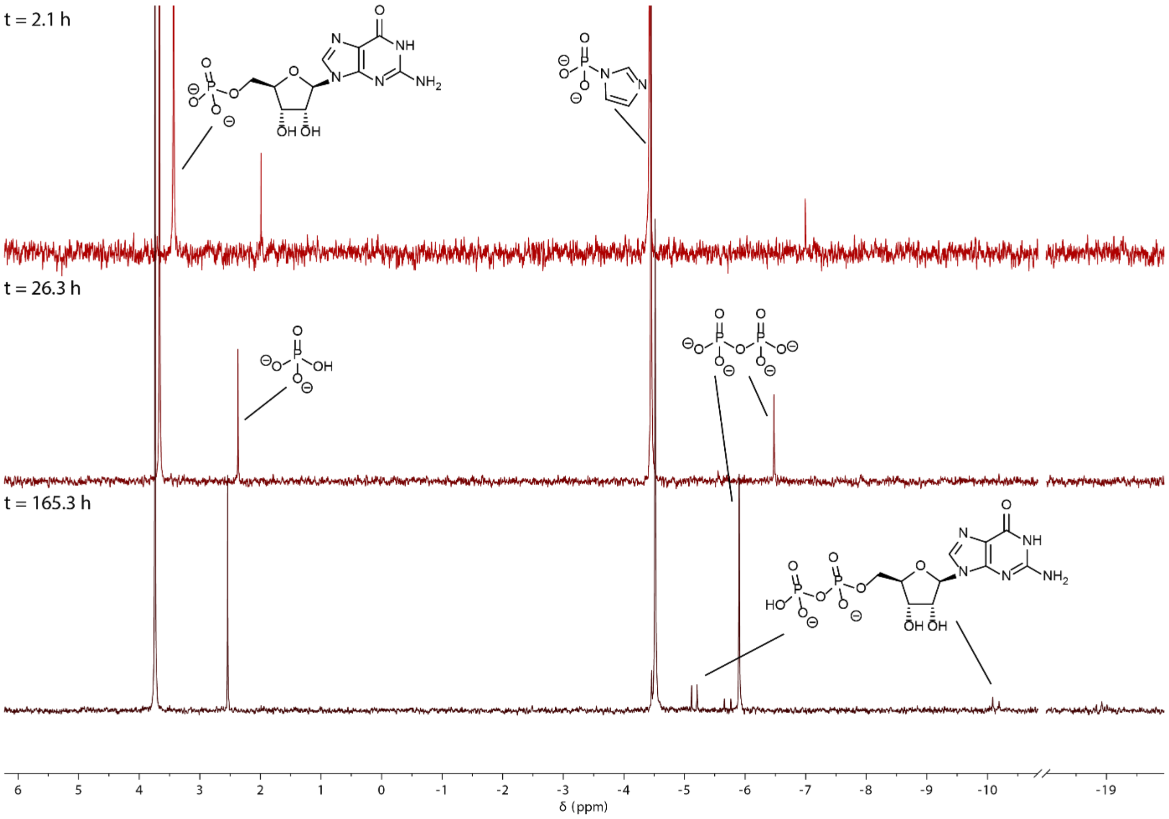

Supplementary Figure 57: Representative ^{31}P NMR spectra over time for the reaction of 0.13 mmol calcium imidazole phosphate + 0.13 mmol guanosine 5'-monophosphate in paste conditions at 80 °C. All NMR spectra were measured in a 0.5 M citric acid buffer at pH 6.85 9 : 1 H_2O : D_2O. The chemical shift of the pyrophosphate peak is variable at pH 6.85 due to the pyrophosphate pK_a = 6.6.

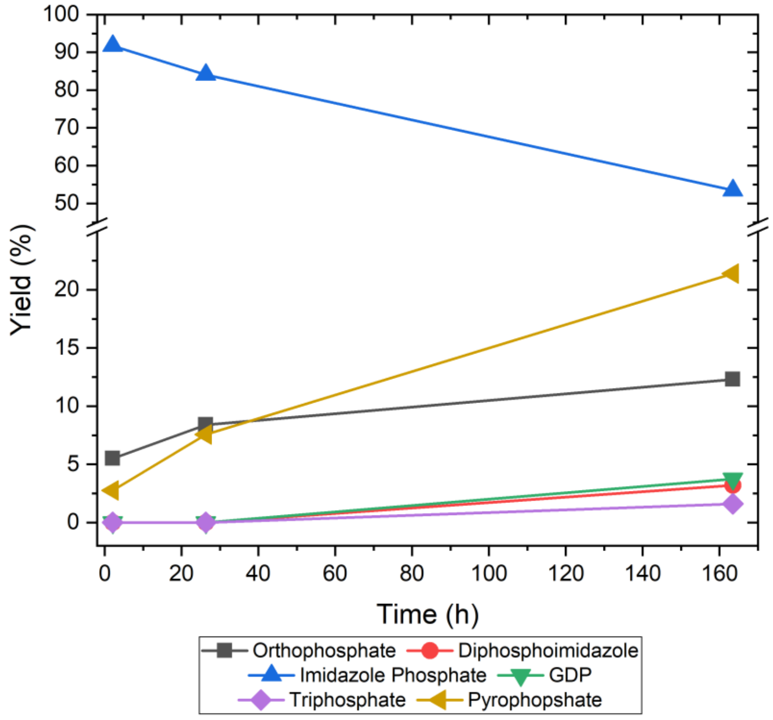

Supplementary Figure 58: Changes in yield over time for the reaction of 0.13 mmol calcium imidazole phosphate + 0.13 mmol guanosine 5'-monophosphate in paste conditions at 80 °C.

S5.5.4 Phosphorylation of adenosine-5'-diphosphate

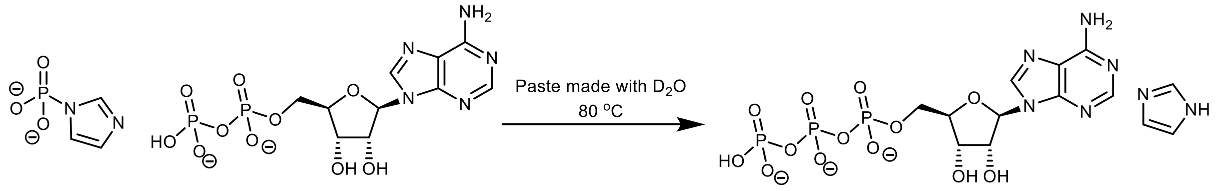

This reaction was performed according to the procedure in Section S5.1 but with 24. 2 mg (0.13 mmol) of calcium imidazole phosphate and with the ribose replaced by 58.7 mg (0.13 mmol) adenosine-5'-diphosphate trisodium salt.

Formation of adenosine-5'-triphosphate occurred in the paste from the reaction between imidazole phosphate and adenosine-5'-diphosphate with the highest observed yield of 3.8 % adenosine-5'-triphosphate after heating the paste at 80 °C for 163.5 h (Supplementary Figure 59 and 60).

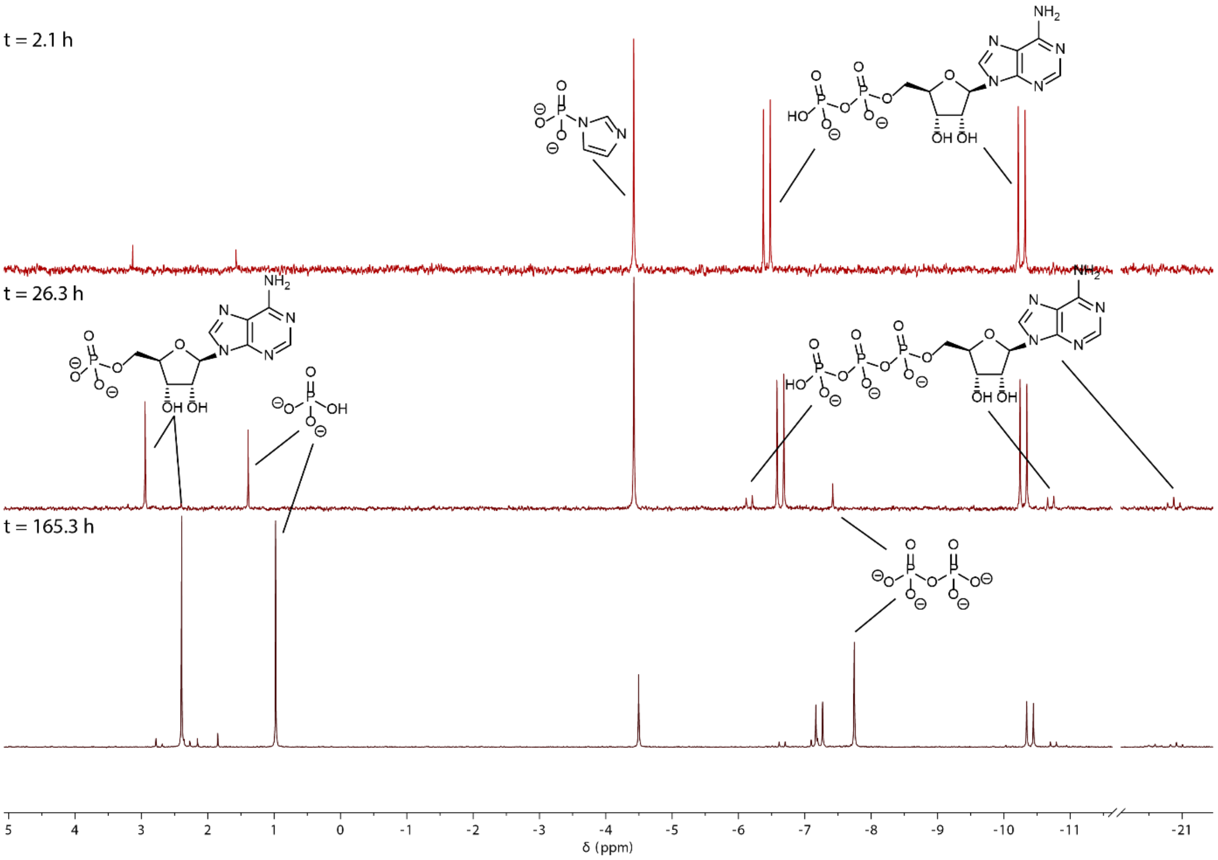

Supplementary Figure 59: Representative ^{31}P NMR spectra over time for the reaction of 0.13 mmol calcium imidazole phosphate + 0.13 mmol adenosine 5'-diphosphate in paste conditions at 80 °C. All NMR spectra were measured in a 0.5 M citric acid buffer at pH 6.85 9 : 1 H_2O : D_2O. The chemical shift of the pyrophosphate peak is variable at pH 6.85 due to the pyrophosphate pK_a = 6.6.

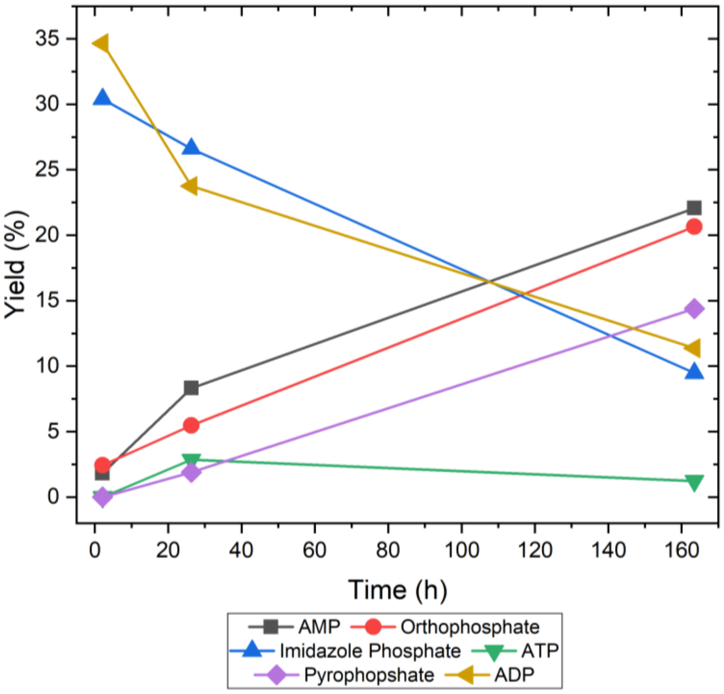

Supplementary Figure 60: Changes in yield over time for the reaction of 0.13 mmol calcium imidazole phosphate + 0.13 mmol adenosine 5'-diphosphate in paste conditions at 80 °C.

S5.6 Phosphorylation of Sugars

S5.6.1 Phosphorylation of ribose

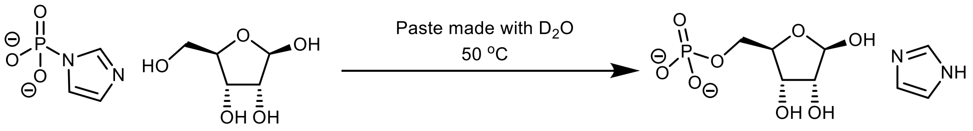

This reaction was performed according to the procedure in Section S5.1. Note that the use of the ribose-5-phosphate structure in the scheme above is to indicate phosphorylated ribose and not to identify the specific species.

Formation of phosphorylated ribose was observed in the paste from the reaction between imidazole phosphate and ribose. Multiple peaks for phosphorylated ribose were observed in the ^{31}P NMR spectra. The summation of all the peaks gave the highest observed yield of 41.5 % phosphorylated ribose after heating the paste at 50 °C for 22.4 h (Supplementary Figure 61 and 62).

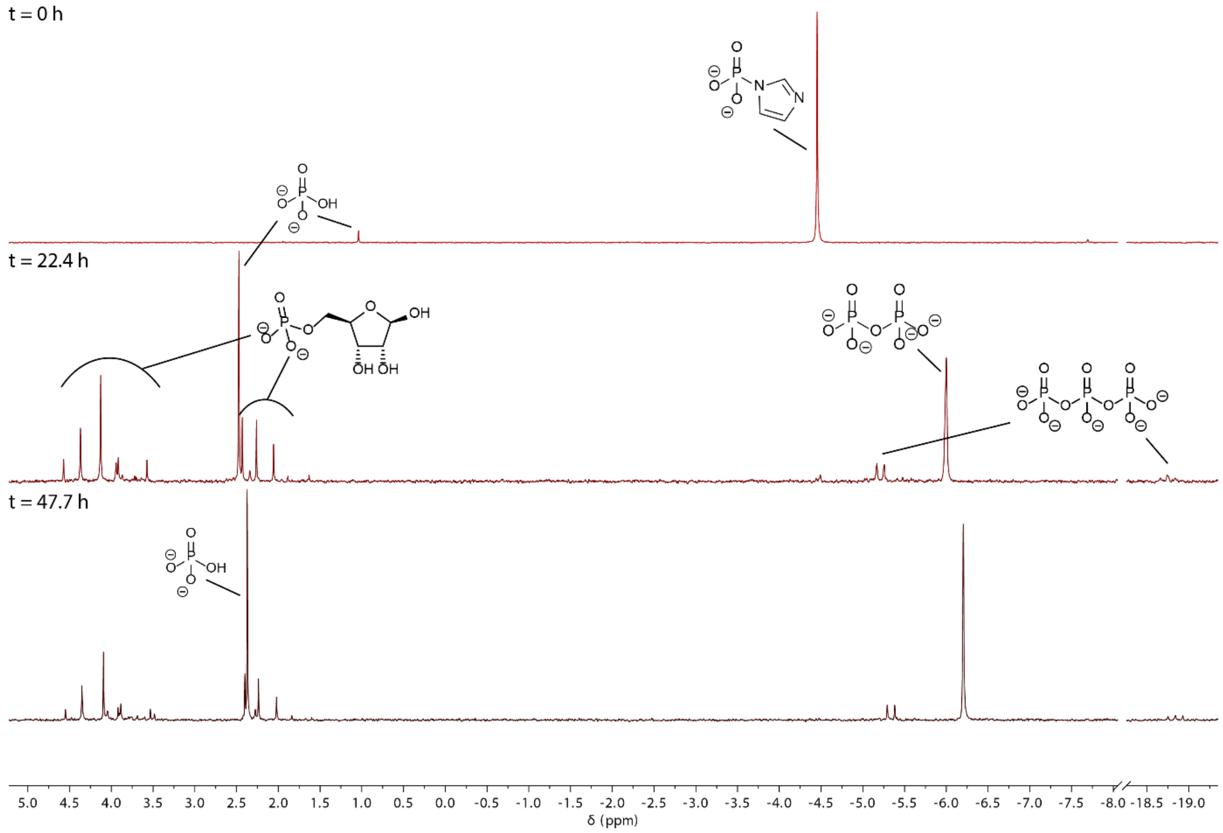

Supplementary Figure 61: Representative ^{31}P NMR spectra over time for the reaction of 0.26 mmol calcium imidazole phosphate + 0.26 mmol ribose in paste conditions at 50 °C. All NMR spectra were measured in a 0.5 M citric acid buffer at pH 6.85 9 : 1 H_2O : D_2O. The chemical shift of the pyrophosphate peak is variable at pH 6.85 due to the pyrophosphate pK_a = 6.6. Use of ribose-5-phosphate structure is to indicate phosphorylated ribose and not to identify the specific species.

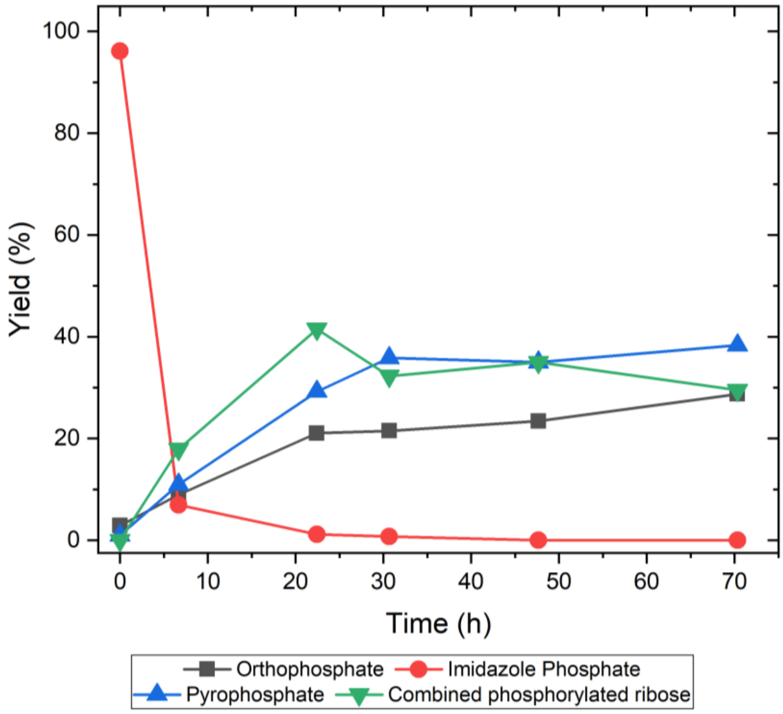

Supplementary Figure 62: Changes in yield over time for the reaction of 0.26 mmol calcium imidazole phosphate + 0.26 mmol ribose in paste conditions at 50 °C.

S5.6.2 Phosphorylation of glucose

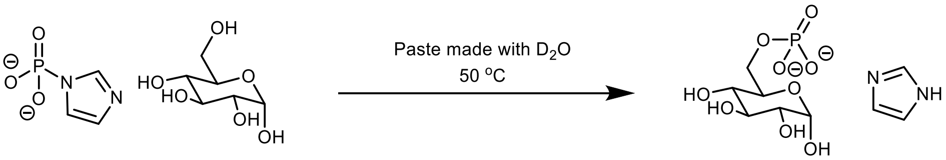

This reaction was performed according to the procedure in Section S5.1 but with the ribose replaced by 46.9 mg (0.26 mmol) glucose. Note that the use of the glucose-6-phosphate structure in the scheme above is to indicate phosphorylated glucose and not to identify the specific species.

Formation of phosphorylated glucose in the paste from the reaction between imidazole phosphate and glucose. Multiple peaks for phosphorylated glucose were observed in the ^{31}P NMR spectra. The summation of all the peaks gave the highest observed yield of 23.7 % phosphorylated glucose and after heating paste at 50 °C for 70.3 h (Supplementary Figure 63 and 64).

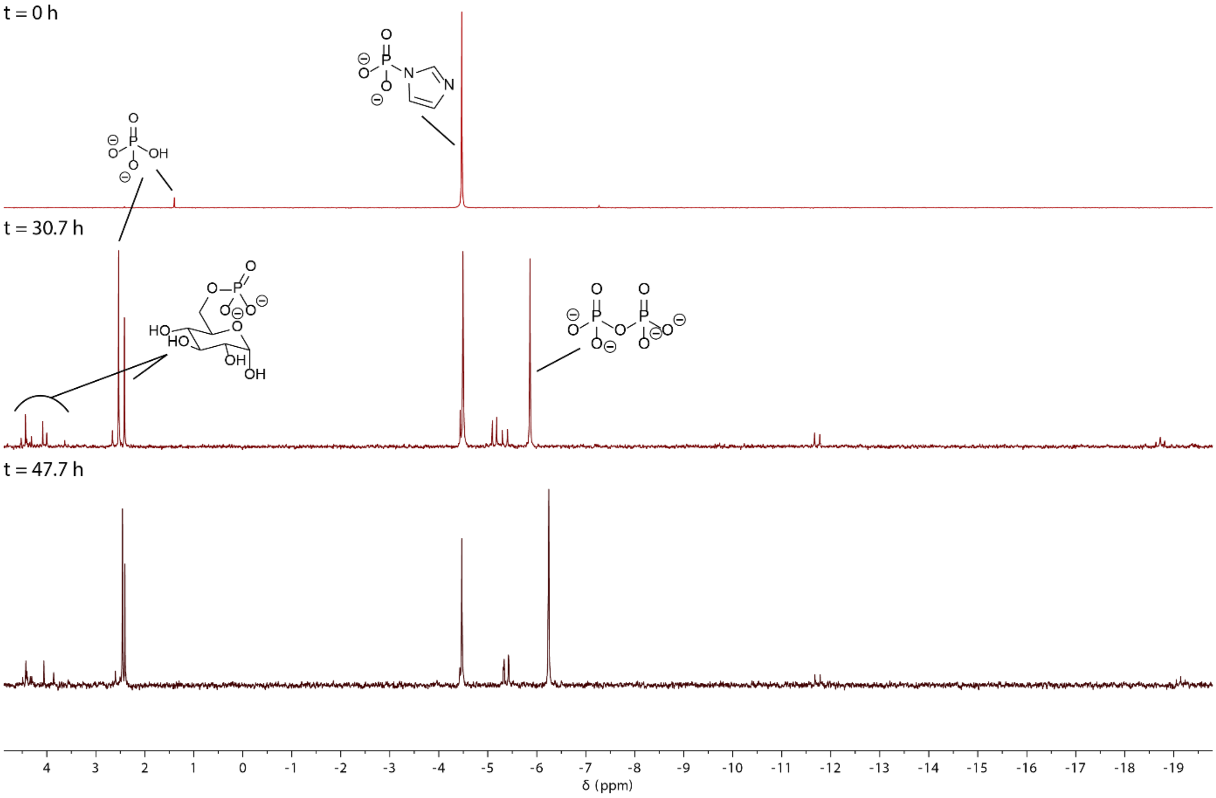

Supplementary Figure 63: Representative ^{31}P NMR spectra over time for the reaction of 0.26 mmol calcium imidazole phosphate + 0.26 mmol glucose in paste conditions at 50 °C. All NMR spectra were measured in a 0.5 M citric acid buffer at pH 6.85 9 : 1 H_2O : D_2O. The chemical shift of the pyrophosphate peak is variable at pH 6.85 due to the pyrophosphate pK_a = 6.6. Use of glucose-6-phosphate structure is to indicate phosphorylated glucose and not to identify the specific species.

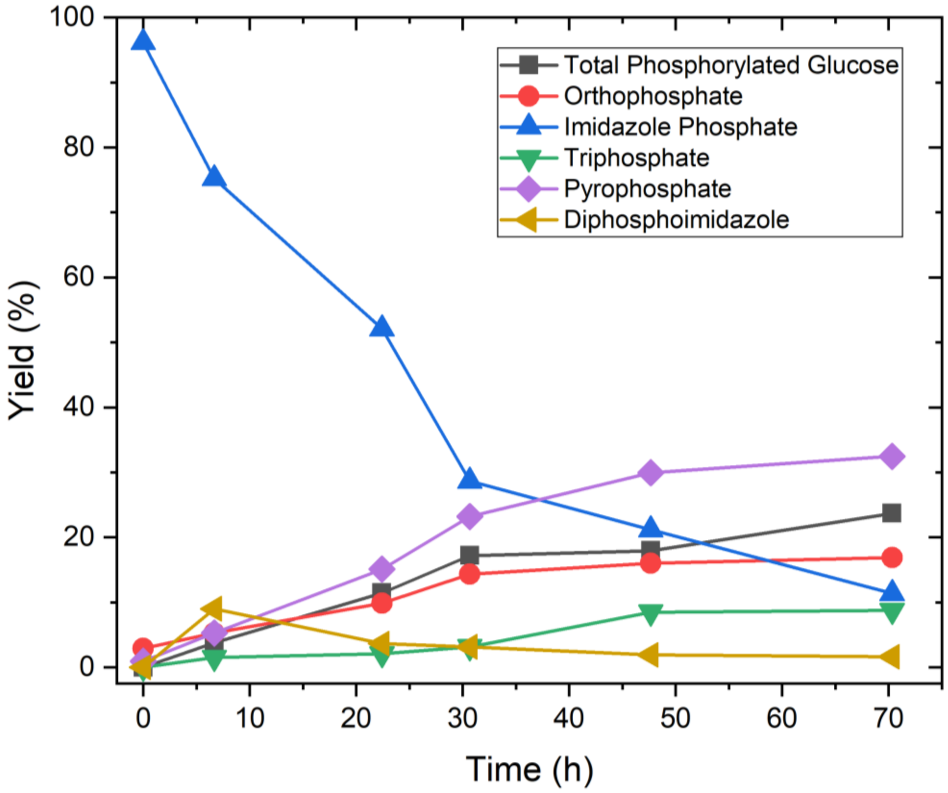

Supplementary Figure 64: Changes in yield over time for the reaction of 0.26 mmol calcium imidazole phosphate + 0.26 mmol glucose in paste conditions at 50 °C.

S6 Wet-to-dry transition prior to phosphorylation of prebiotically important organic compounds (Main Text Table 1)

S6.1 Supplementary experimental method for wet/dry cycle followed by phosphorylating in a paste

24.2 mg (0.13 mmol, 1 eq) of calcium imidazole phosphate (see SI Section S3 for the synthesis of calcium imidazole phosphate) and 98.5 mg (0.65 mmol, 5 eq) of D-ribose were dissolved in 2.0 mL of MilliQ water to give a 65 mM calcium imidazole phosphate and a 325 mM D-ribose solution. The solution was then added to a petri dish and left with the lid off to dry at 22 °C for 24 h. The resulting paste was scraped from the petri dish and placed into an Eppendorf. The Eppendorf was spun on a centrifuge for 5 min at 5590 g in order to move all paste to the bottom of the Eppendorf and compact it together. An approximately 10 mg sample of the paste was taken to record the extent of phosphorylation after the wet-to-dry transition to a paste. The Eppendorf was placed in a thermoshaker at 50 °C and 600 rpm for up to 168 h. The reaction was followed by periodically removing samples (approximately 10 mg), dissolving them in 0.5 mL of 0.5 M citric acid buffer at pH 6.85 and 9 : 1 H_2O : D_2O and analysing them with ^{31}P-NMR and ^{1}H-NMR and ^{1}H ^{31}P HMBC NMR spectroscopy

S6.2 Phosphorylation of Amphiphiles

S6.2.1 Phosphorylation of glycerol

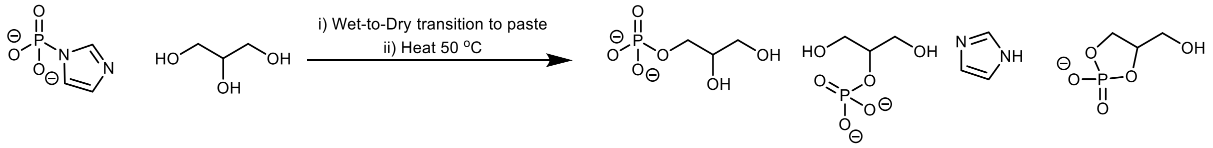

This reaction was performed according to the procedure in Section S6.1 but with the ribose replaced by 61.3 mg (0.65 mmol) glycerol.

Formation of glycerol-1-phosphate and glycerol-2-phosphate occurred in the paste from the reaction between imidazole phosphate and glycerol with the highest observed yield of 23.8 % glycerol-1-

phosphate and 13.9 % glycerol-2-phosphate after heating paste at 50 °C for 167.3 h (Supplementary Figure 65a, 65b, 65c and 66).

Glycerol-1,2-cyclic phosphate was also observed with the highest observed yield of 6.3 % at 23.4 h (Supplementary Figure 65). The peak for glycerol-1,2-cyclic phosphate has a chemical shift 18.42 ppm in Supplementary Figure 65 and was assigned based upon the peak for glycerol-1,2-cyclic phosphate at in a chemical shift of 18.64 ppm from J. D. Sutherland *et al. Nat. Chem.* **2015**, *7*, 301-307 Supporting Information Supplementary Figure 3.

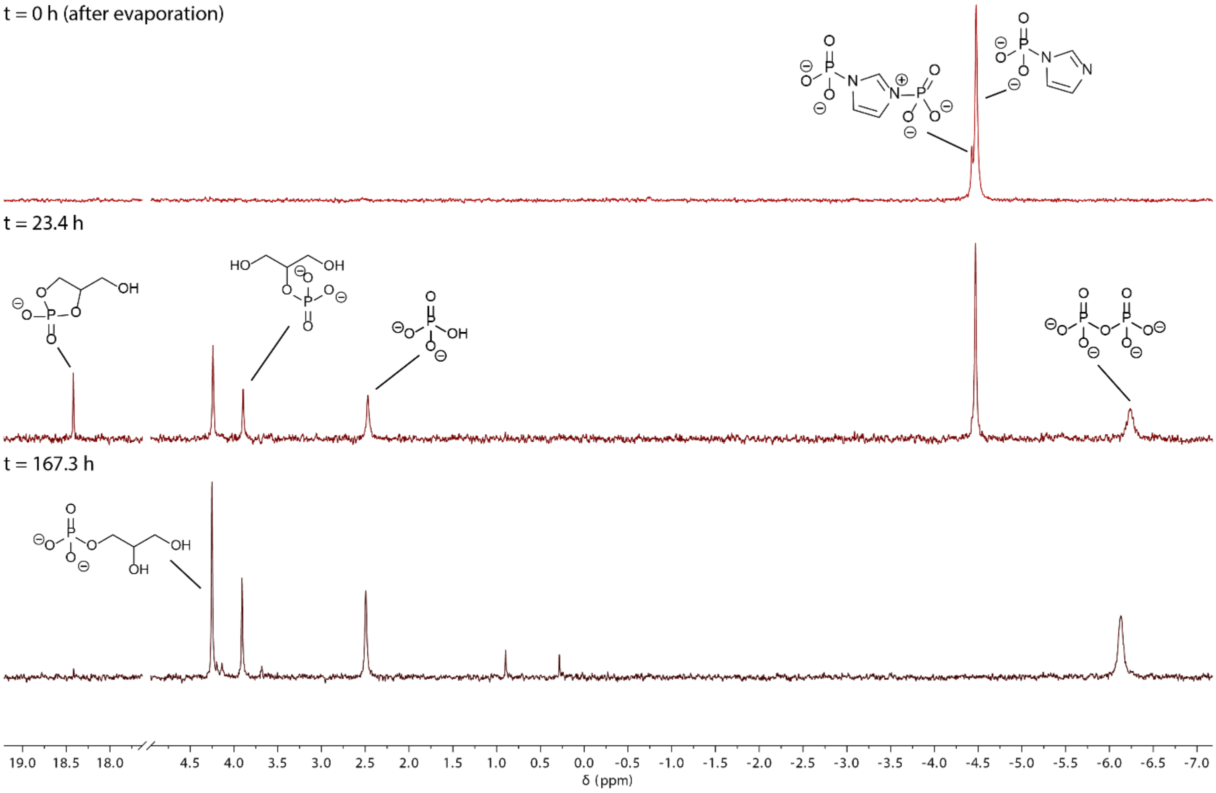

Supplementary Figure 65a: Representative ^{31}P NMR spectra over time for the reaction of 0.13 mmol calcium imidazole phosphate + 0.65 mmol glycerol in paste conditions at 50 °C. Top spectrum was taken after evaporation to form a paste.

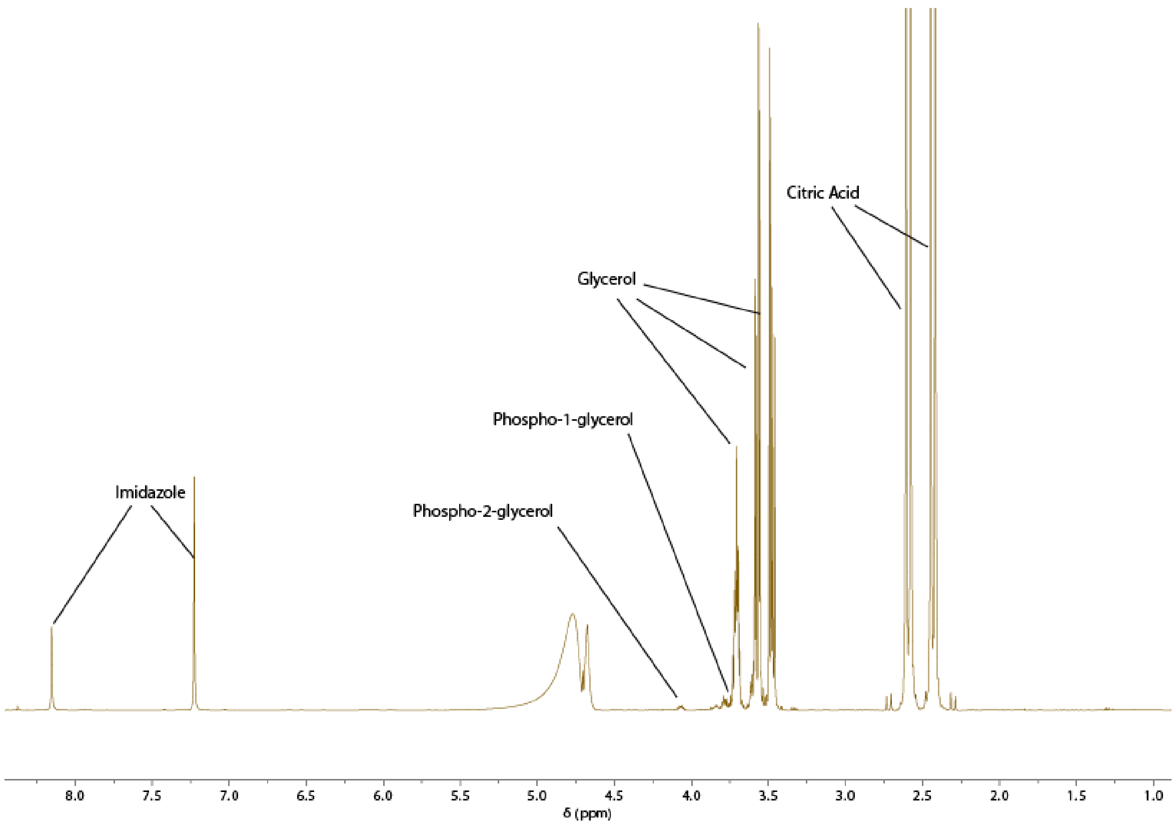

Supplementary Figure 65b: ^{1}H NMR spectrum at 167.3 h for the reaction of 0.13 mmol calcium imidazole phosphate + 0.65 mmol glycerol in paste conditions at 50 °C.

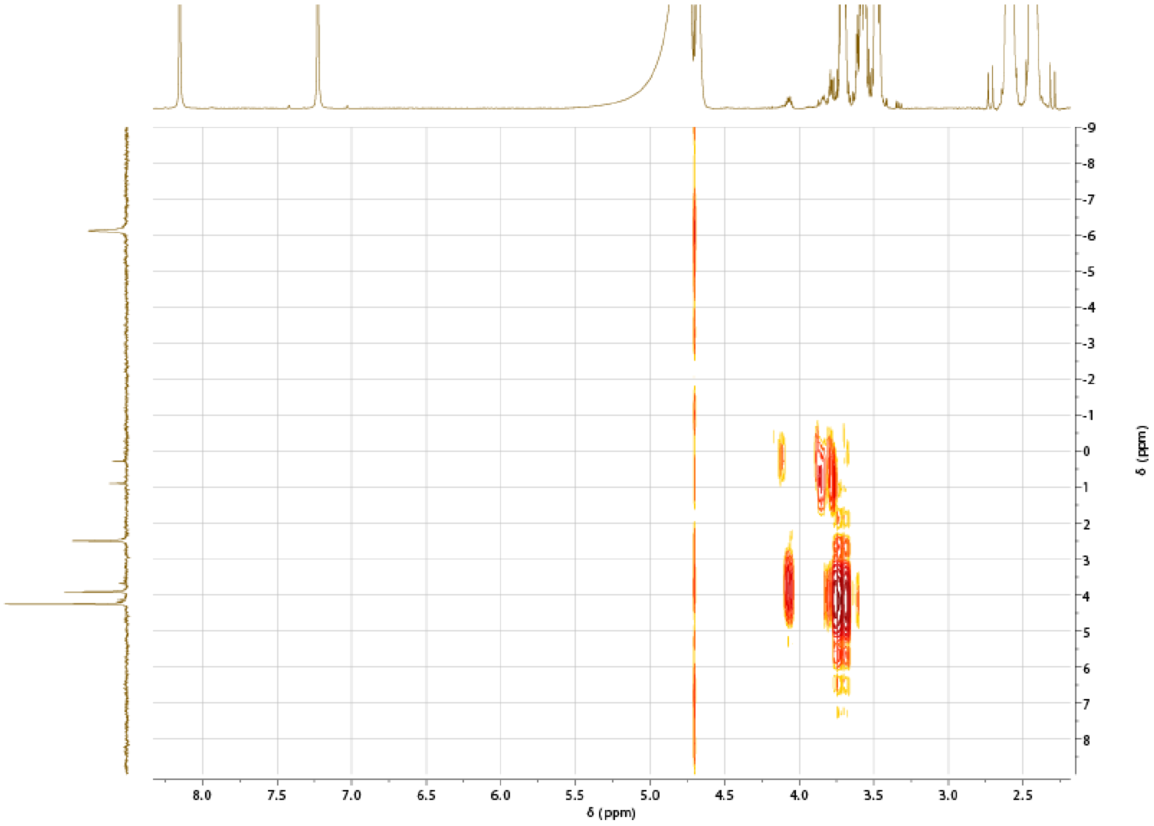

Supplementary Figure 65b: ^{1}H ^{31}P HMBC NMR spectrum at 167.3 h for the reaction of 0.13 mmol calcium imidazole phosphate + 0.65 mmol glycerol in paste conditions at 50 °C.

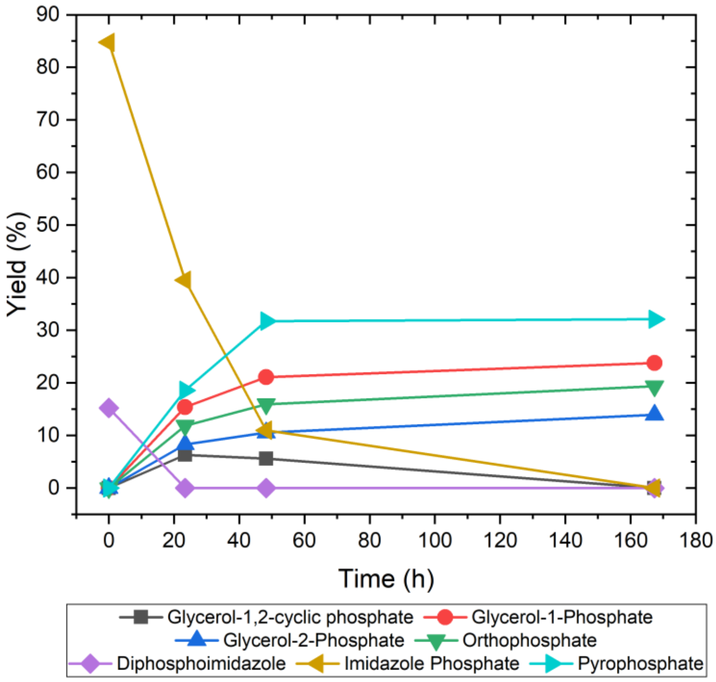

Supplementary Figure 66: Changes in yield over time for the reaction of 0.13 mmol calcium imidazole phosphate + 0.65 mmol glycerol in paste conditions at 50 °C.

A spiking experiment with genuine samples of glycerol-1-phosphate and glycerol-2-phosphate confirmed their formation from the reaction of calcium imidazole phosphate and glycerol (Supplementary Figure 67).

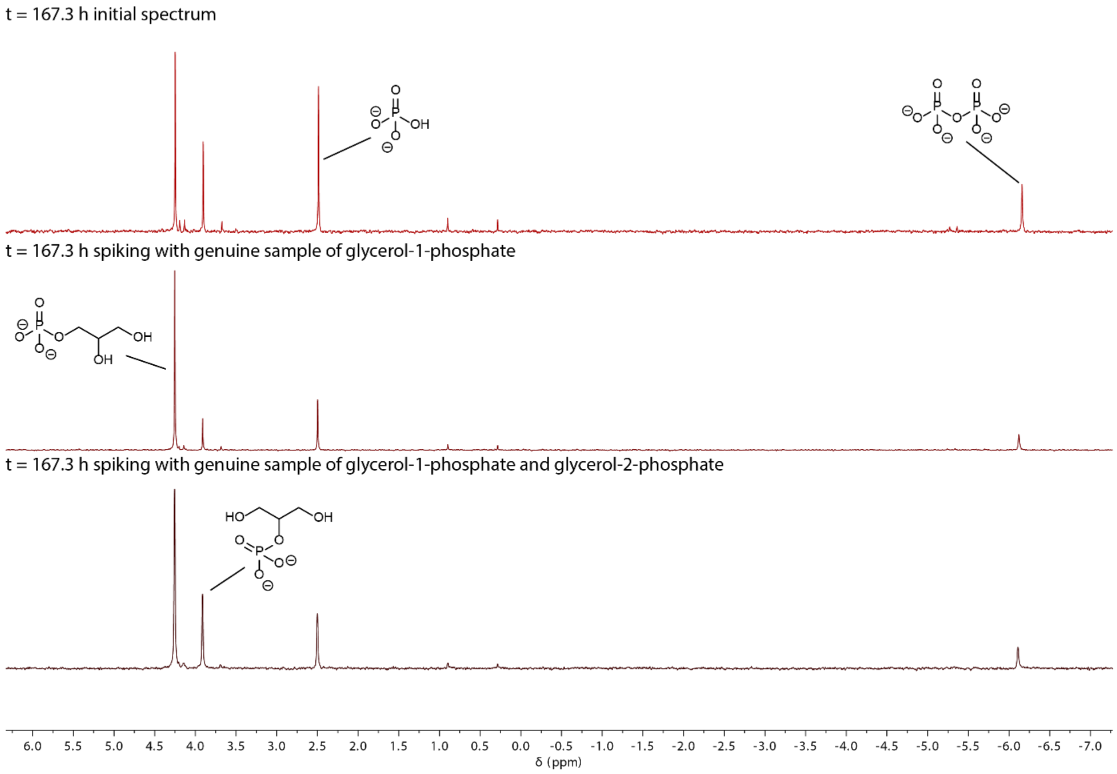

Supplementary Figure 67: ^{31}P NMR spectra of a spiking experiment for the reaction of 0.13 mmol calcium imidazole phosphate + 0.65 mmol glycerol in paste conditions at 50 °C. Top spectrum is unspiked. Middle spectrum is with the addition of genuine glycerol-1-phosphate. Bottom spectrum is with the addition of genuine glycerol-2-phosphate.

S6.3 Phosphorylation of Amino acids + Peptides

S6.3.1 Phosphorylation of serine methyl ester

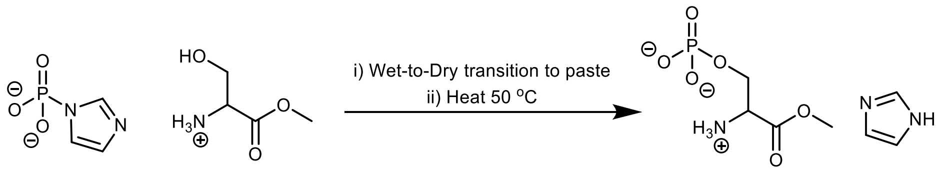

This reaction was performed according to the procedure in Section S6.1 but with the ribose replaced by 101.4 mg (0.65 mmol) serine methyl ester·HCl.

Formation of O-phosphorylated serine methyl ester and O-phosphorylated serine occurred in the paste from the reaction between imidazole phosphate and serine methyl ester. Hydrolysis of the serine methyl ester also occurred in the paste. The highest observed yield of 2.3 % O-phosphorylated serine methyl ester and 9.4 % O-phosphorylated serine were obtained after heating the paste at 50 °C for 169.1 h (Supplementary Figure 68a, 68b, 68c and 69).

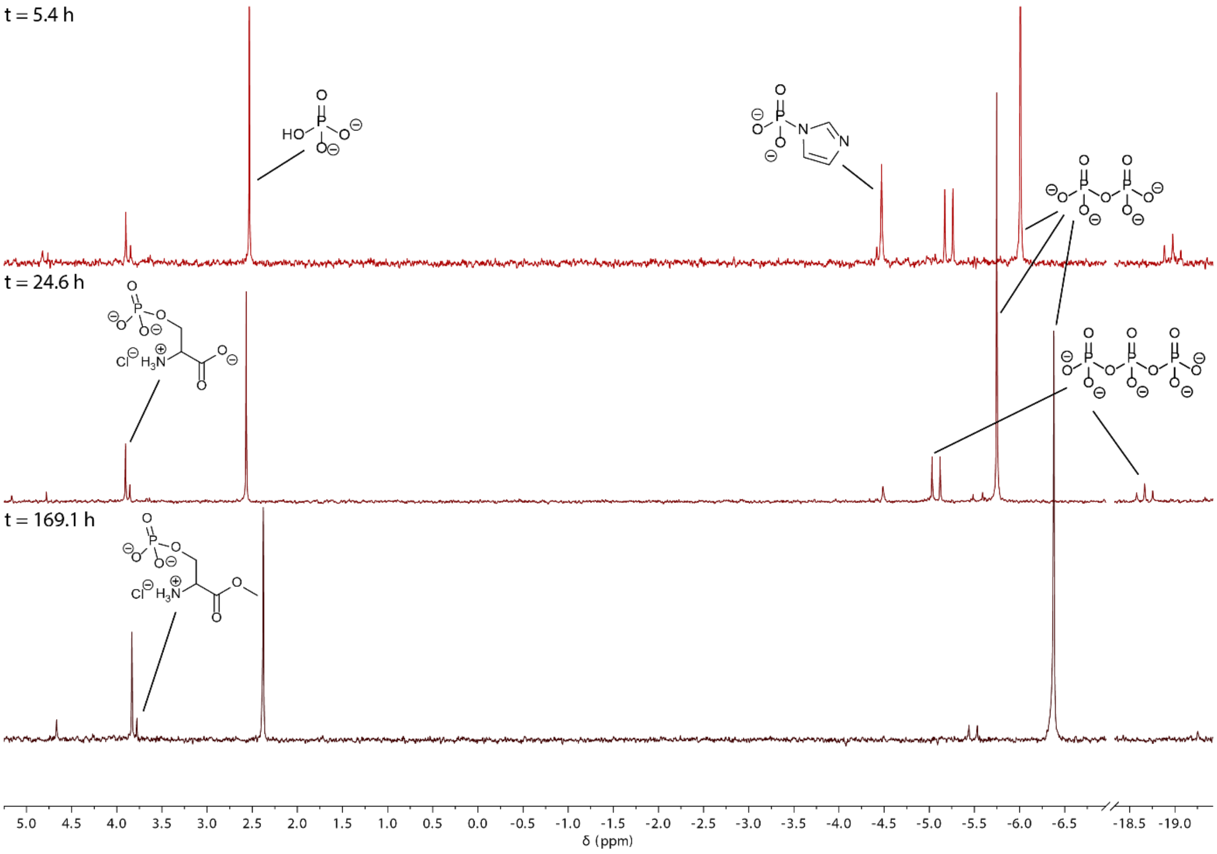

Supplementary Figure 68a: Representative ^{31}P NMR spectra over time for the reaction of 0.13 mmol calcium imidazole phosphate + 0.65 mmol serine methyl ester in paste conditions at 50 °C. Top spectrum was taken after evaporation to form a paste.

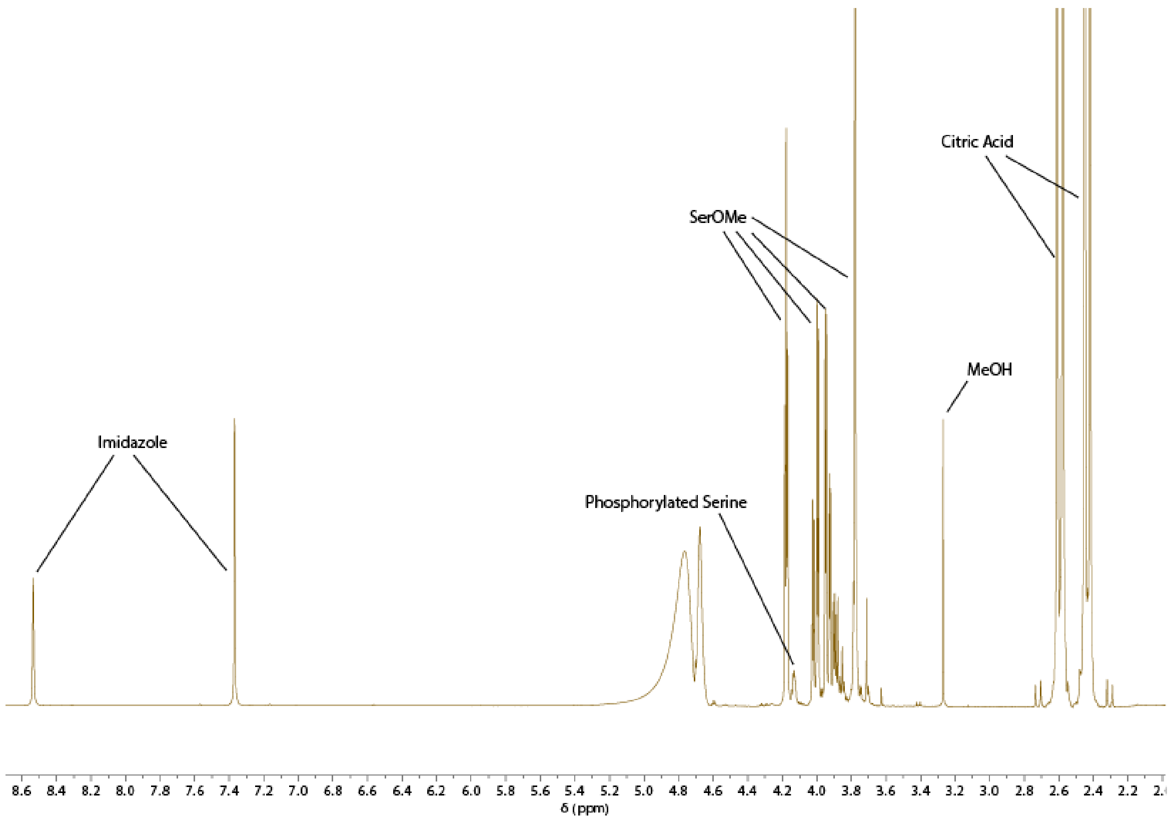

Supplementary Figure 68b: ^{1}H NMR spectrum at 167.1 h for the reaction of 0.13 mmol calcium imidazole phosphate + 0.65 mmol serine methyl ester in paste conditions at 50 °C.

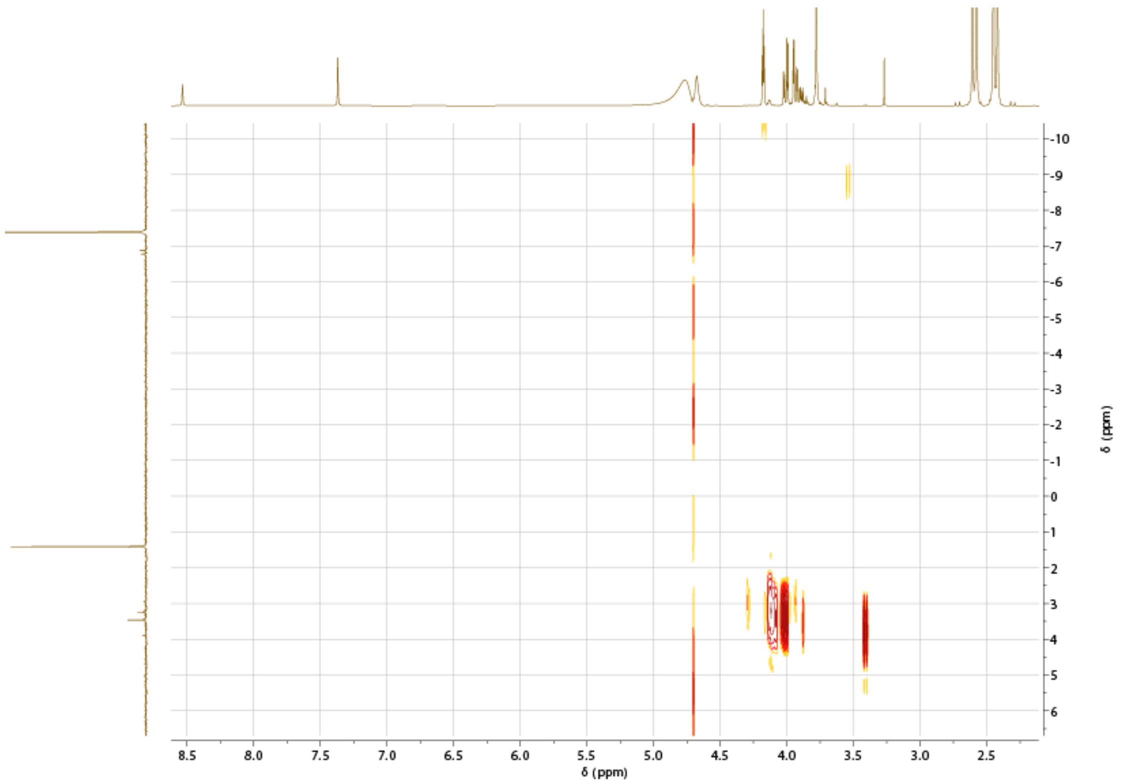

Supplementary Figure 68c: ^{1}H ^{31}P HMBC spectrum at 167.1 h for the reaction of 0.13 mmol calcium imidazole phosphate + 0.65 mmol serine methyl ester in paste conditions at 50 °C.

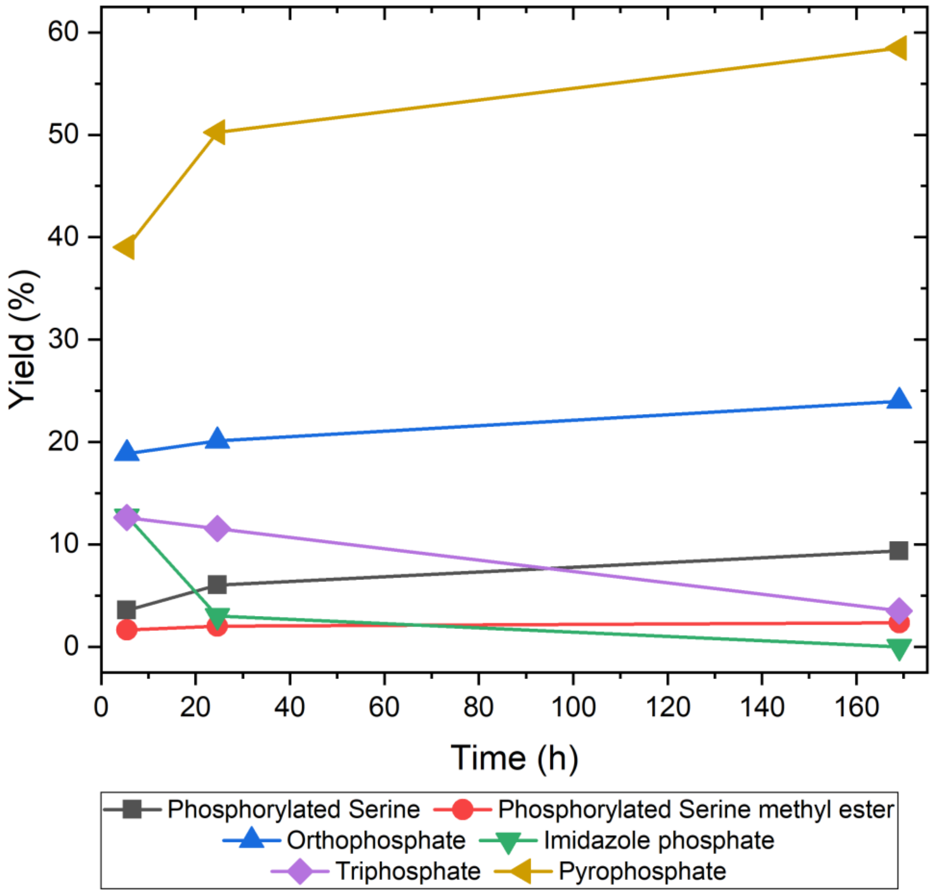

Supplementary Figure 69: Changes in yield over time for the reaction of 0.13 mmol calcium imidazole phosphate + 0.65 mmol serine methyl ester in paste conditions at 50 °C.

A spiking experiment with genuine samples of O-phosphorylated serine methyl ester and O-phosphorylated serine confirmed the formation of O-phosphorylated serine methyl ester and O-phosphorylated serine from the reaction of calcium imidazole phosphate and serine methyl ester (Supplementary Figure 70).

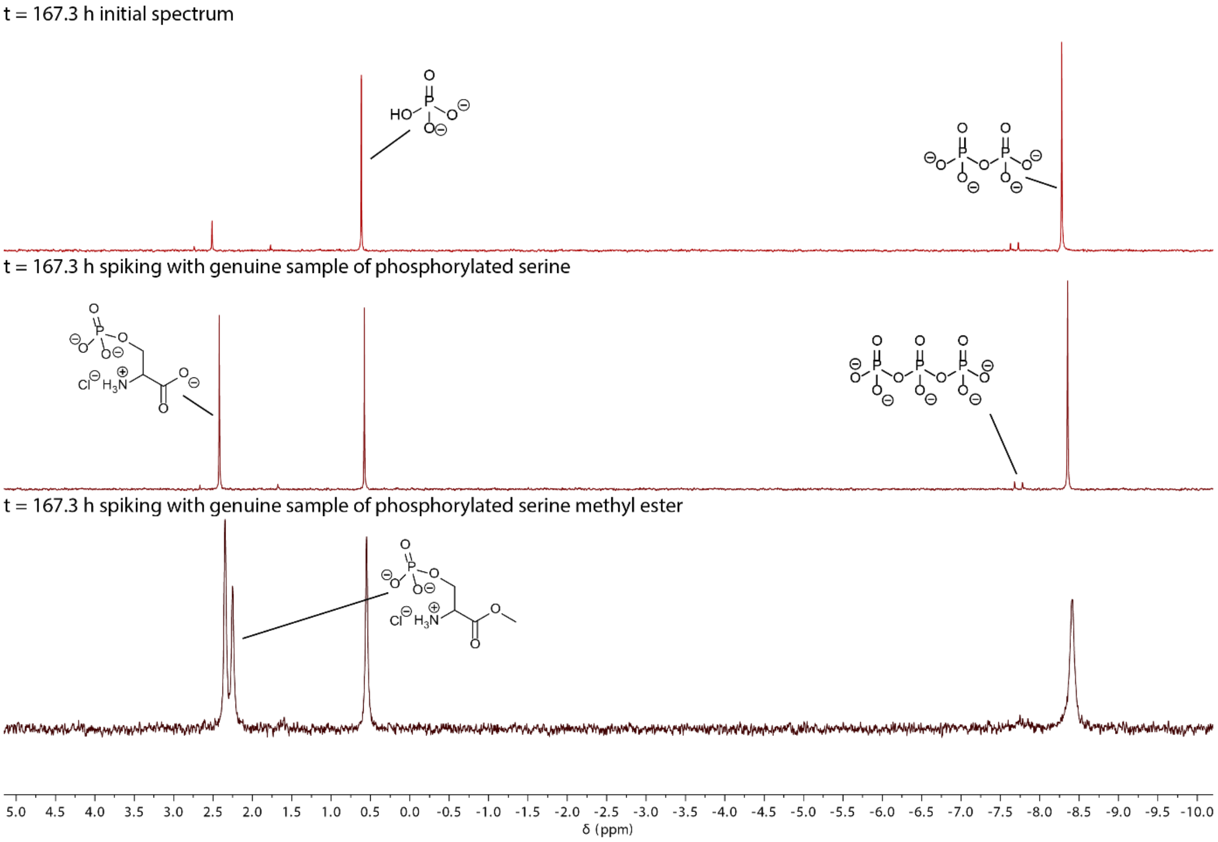

Supplementary Figure 70: ^{31}P NMR spectra of a spiking experiment for the reaction of 0.13 mmol calcium imidazole phosphate + 0.65 mmol serine in paste conditions at 50 °C. Top spectrum is unspiked. Middle spectrum is with the addition of genuine O-phosphorylated serine. Bottom spectrum is with the addition of genuine O-phosphorylated serine methyl ester.

S6.3.2 Phosphorylation of serine

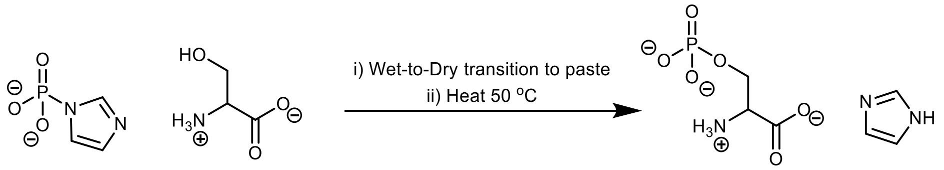

This reaction was performed according to the procedure in Section S6.1 but with the ribose replaced by 67.9 mg (0.65 mmol) serine.

Formation of O-phosphorylated serine occurred in the paste from the reaction between imidazole phosphate and serine with the highest observed yield of 16.5 % after heating the paste at 50 °C for 45.5 h (Supplementary Figure 71a, 71b, 71c and 72).

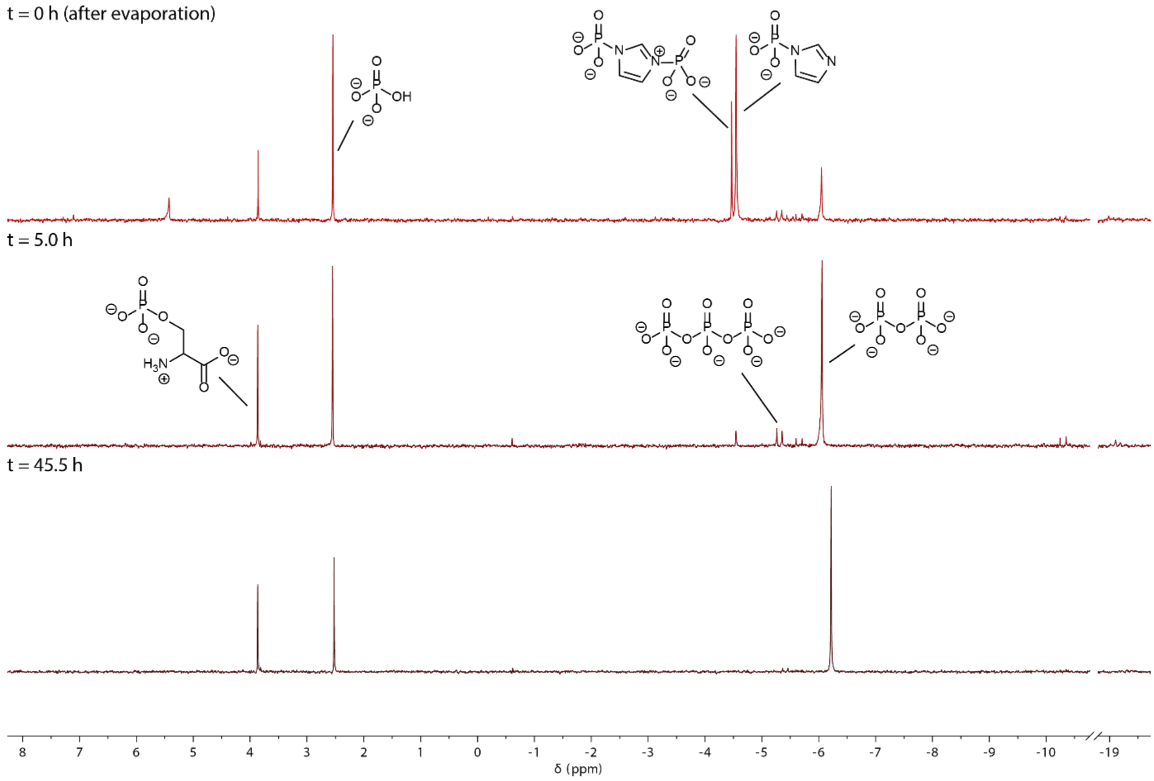

Supplementary Figure 71a: Representative ^{31}P NMR spectra over time for the reaction of 0.13 mmol calcium imidazole phosphate + 0.65 mmol serine in paste conditions at 50 °C. Top spectrum was taken after evaporation to form a paste.

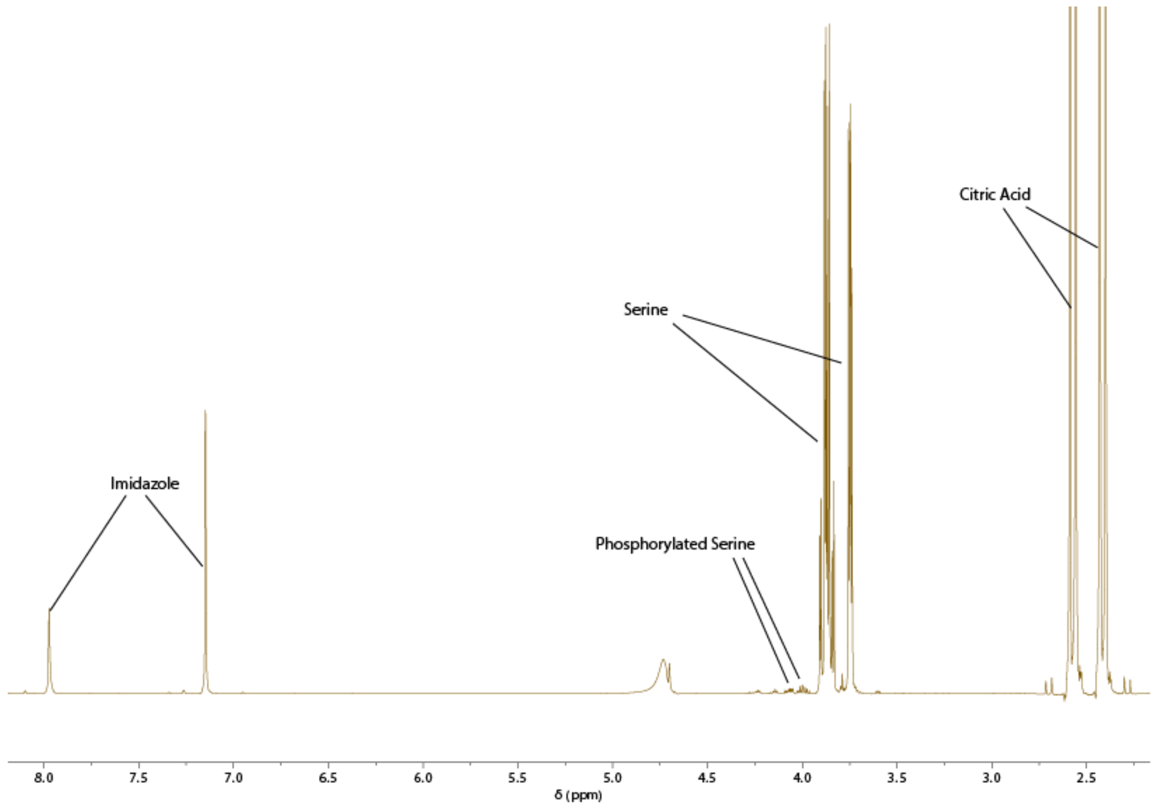

Supplementary Figure 71b: ^{1}H NMR spectrum at 5.0 h for the reaction of 0.13 mmol calcium imidazole phosphate + 0.65 mmol serine in paste conditions at 50 °C.

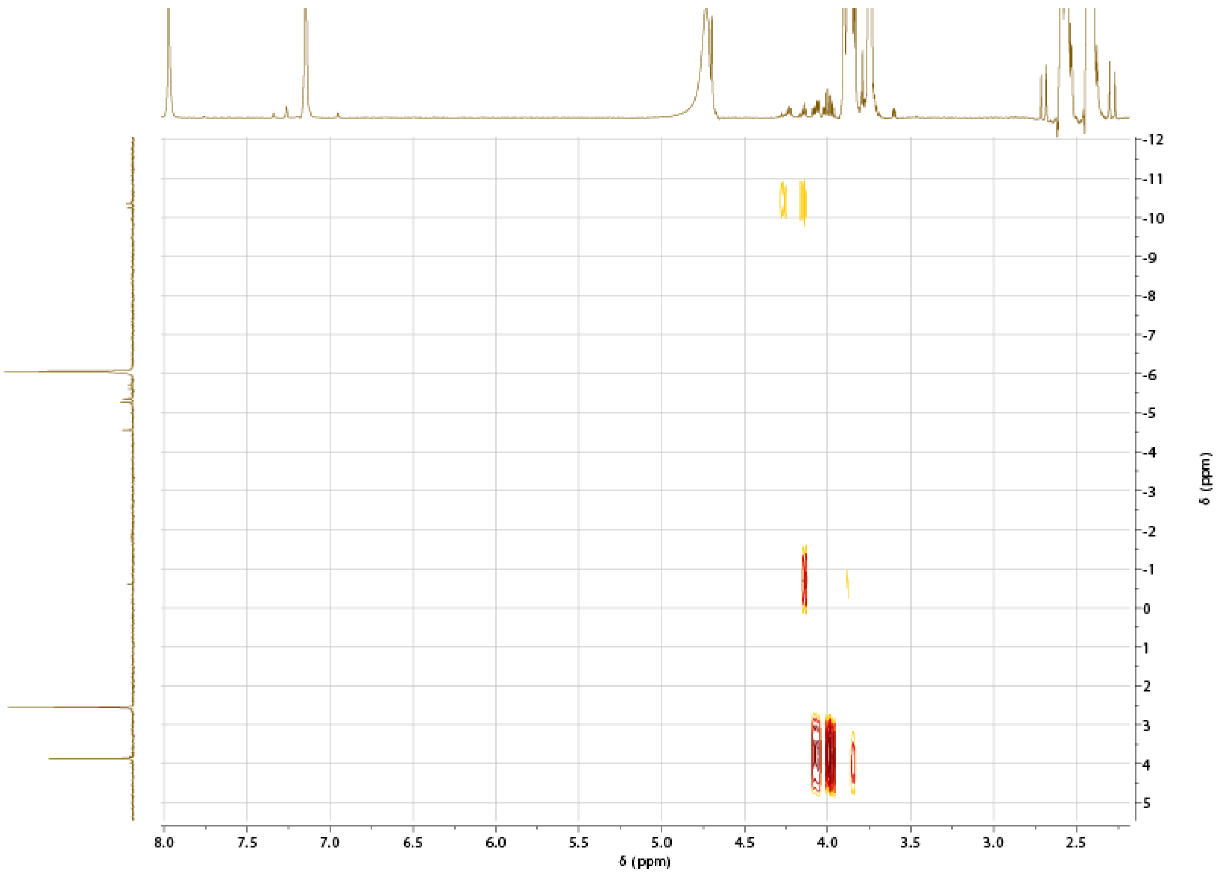

Supplementary Figure 71c: ^{1}H ^{31}P HMBC NMR spectrum at 5.0 h for the reaction of 0.13 mmol calcium imidazole phosphate + 0.65 mmol serine in paste conditions at 50 °C.

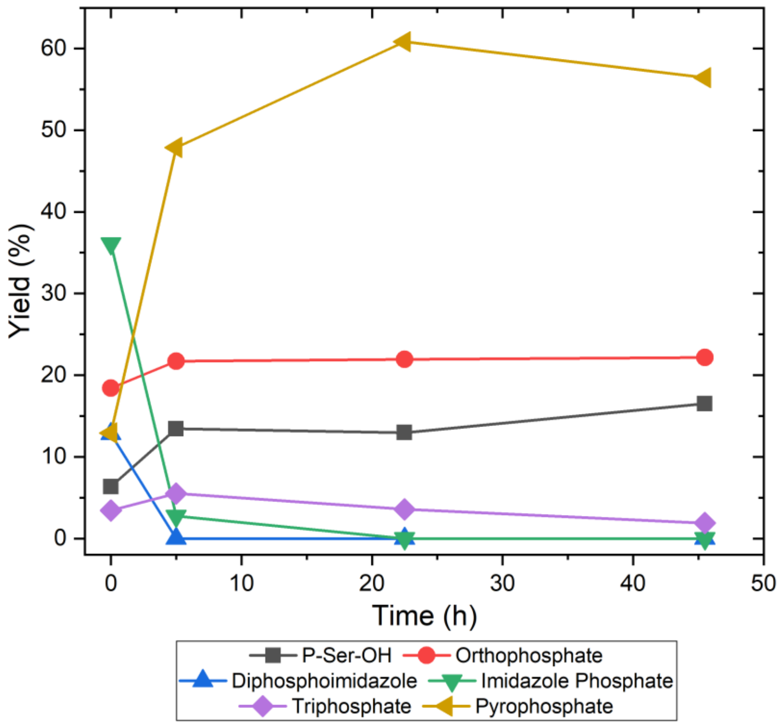

Supplementary Figure 72: Changes in yield over time for the reaction of 0.13 mmol calcium imidazole phosphate + 0.65 mmol serine in paste conditions at 50 °C.

A spiking experiment with a genuine sample of O-phosphorylated serine confirmed the formation of O-phosphorylated serine from the reaction of calcium imidazole phosphate and serine (Supplementary Figure 73).

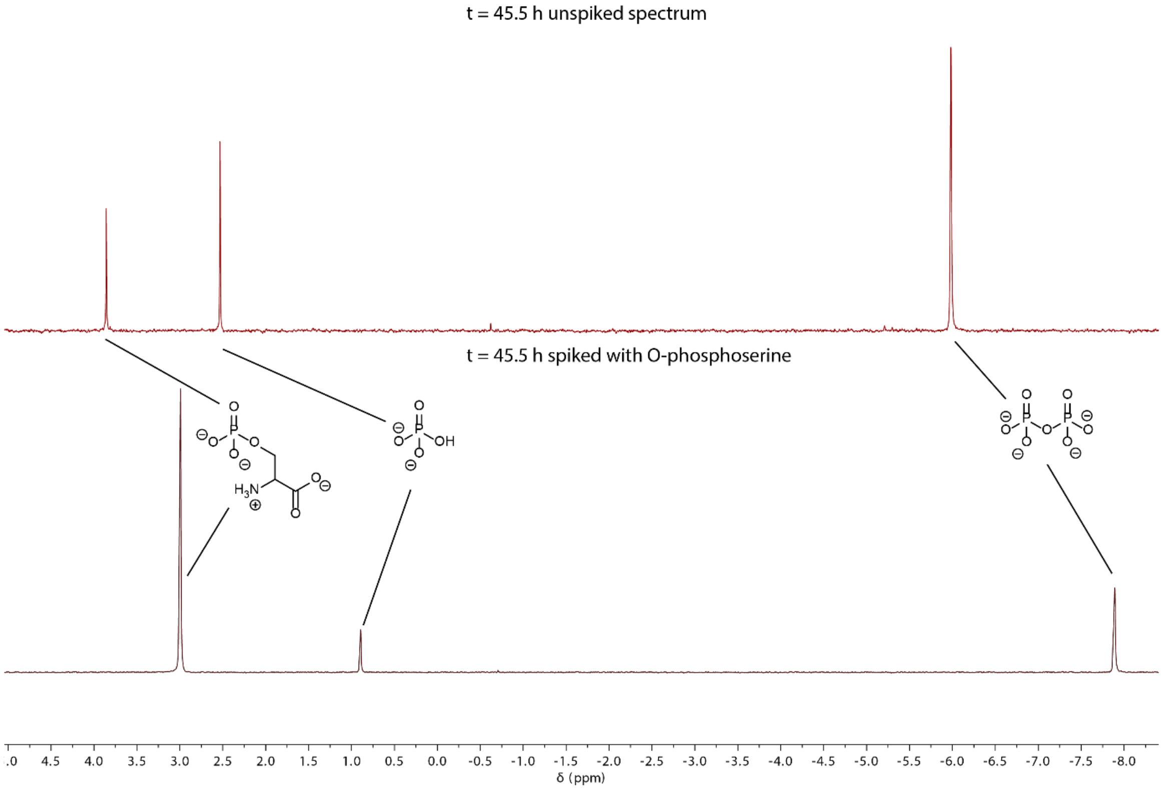

Supplementary Figure 73: ^{31}P NMR spectra of a spiking experiment for the reaction of 0.13 mmol calcium imidazole phosphate + 0.65 mmol serine in paste conditions at 50 °C. Top spectrum is unspiked. Bottom spectrum is with the addition of genuine O-phosphorylated serine. The addition of the genuine sample of O-phosphoserine caused a change in the pH of the solution which changed the chemical shifts of all ^{31}P peaks.

S6.3.3 Phosphorylation of threonine methyl ester

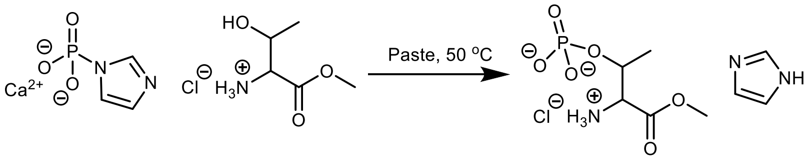

This reaction was performed according to the procedure in Section S6.1 but with the ribose replaced by 77.2 mg (0.65 mmol) threonine methyl ester.

Formation of phosphorylated threonine methyl ester occurred in the paste from the reaction between imidazole phosphate and threonine methyl ester with the highest observed yield of 4.3 % after heating the paste at 50 °C for 47.2 h (Supplementary Figure 74a, 74b, 74c and 75). Hydrolysis of the threonine methyl ester also occurred and phosphorylated threonine was also observed.

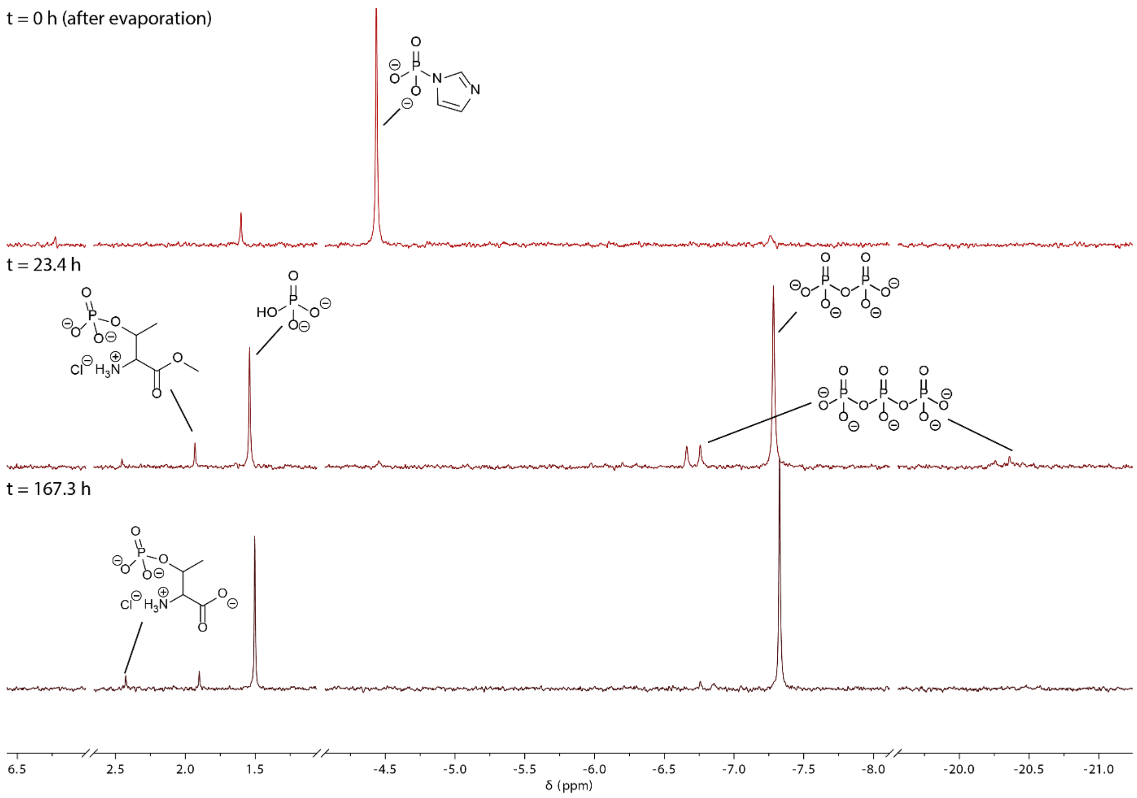

Supplementary Figure 74a: Representative ^{31}P NMR spectra over time for the reaction of 0.13 mmol calcium imidazole phosphate + 0.65 mmol threonine methyl ester in paste conditions at 50 °C. Top spectrum was taken after evaporation to form a paste.

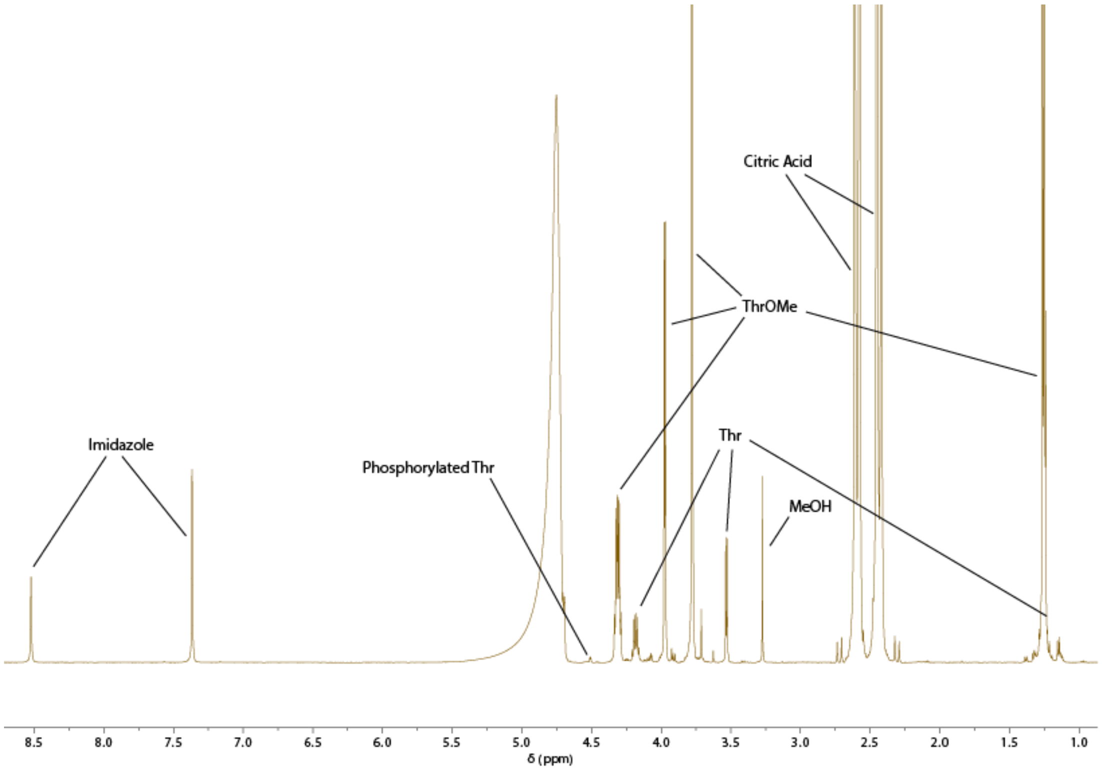

Supplementary Figure 74b: ^{1}H NMR spectrum at 167.3 h for the reaction of 0.13 mmol calcium imidazole phosphate + 0.65 mmol threonine methyl ester in paste conditions at 50 °C.

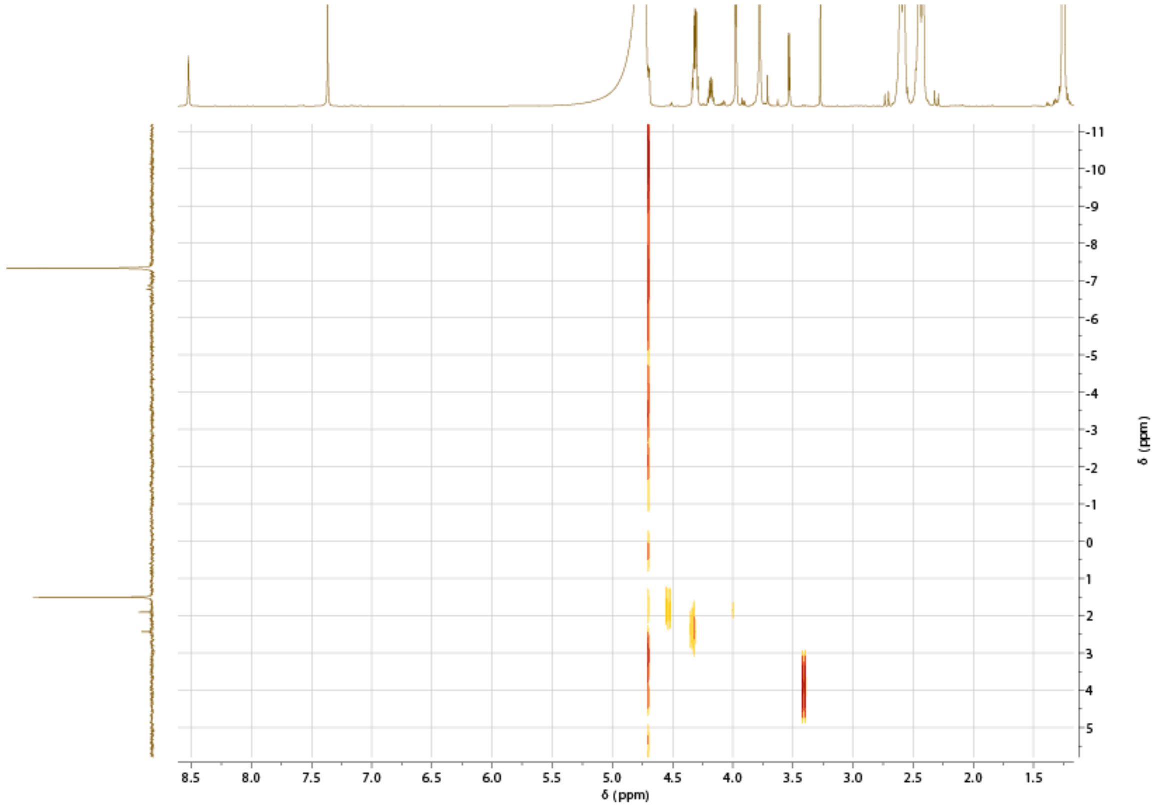

Supplementary Figure 74c: ^{1}H ^{31}P HMBC spectrum at 167.3 h for the reaction of 0.13 mmol calcium imidazole phosphate + 0.65 mmol threonine methyl ester in paste conditions at 50 °C.

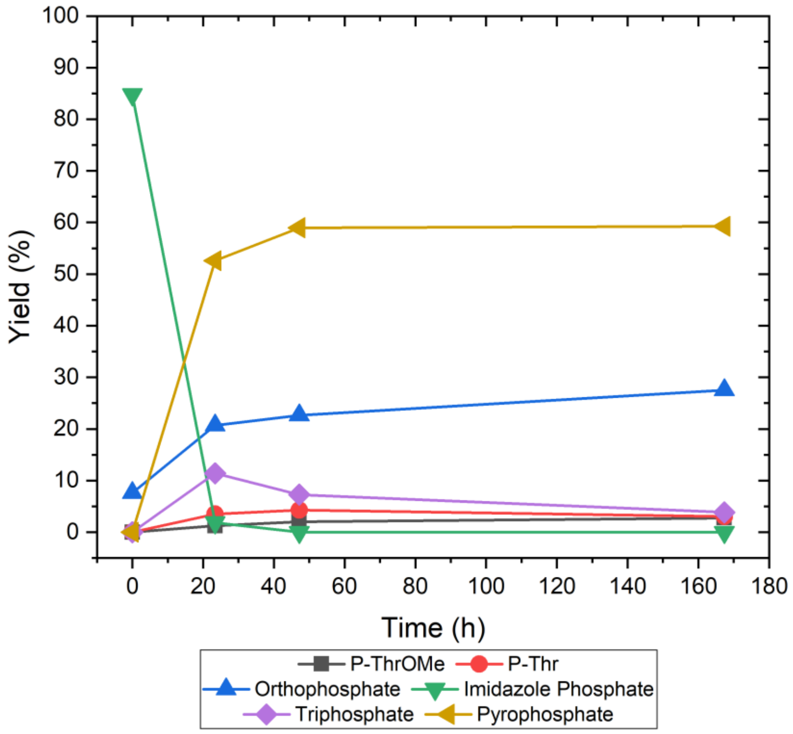

Supplementary Figure 75: Changes in yield over time for the reaction of 0.13 mmol calcium imidazole phosphate + 0.65 mmol threonine methyl ester in paste conditions at 50 °C.

S6.3.4 Phosphorylation of Ser-His

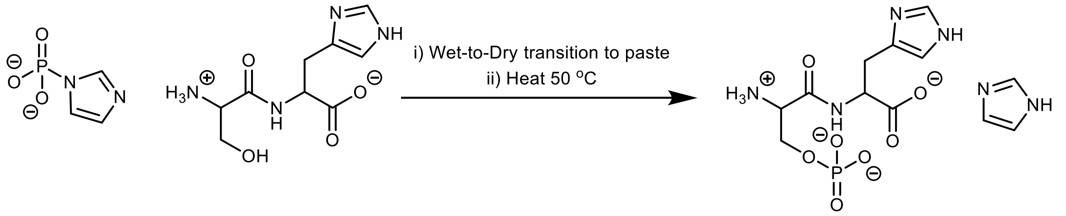

This reaction was performed according to the procedure in Section S6.1 but with the ribose replaced by 94.3 mg (0.325 mmol) Ser-His acetate salt.

Formation of phosphorylated Ser-His occurred in the paste from the reaction between imidazole phosphate and Ser-His with the highest observed yield of 5.6 % after heating the paste at 50 °C for 45.5 h (Supplementary Figure 76a, 76b, 76c and 77).

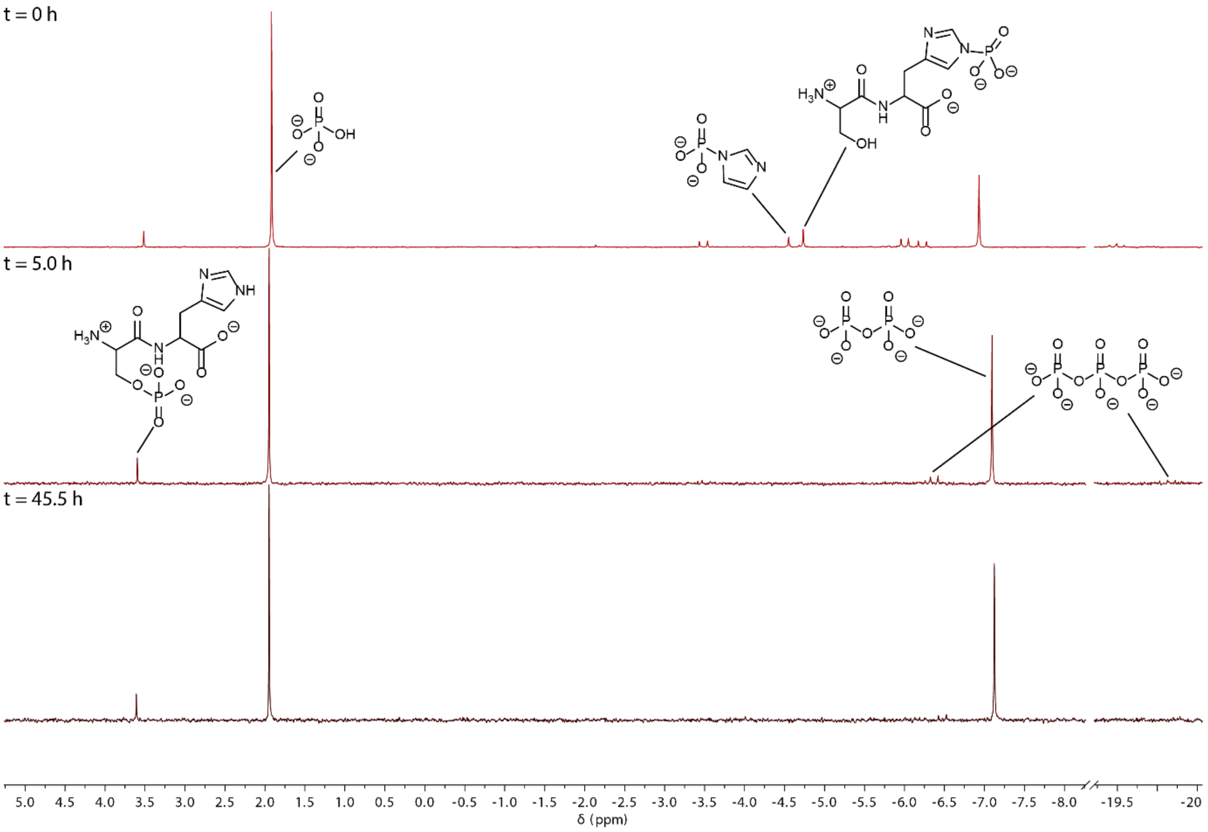

Supplementary Figure 76a: Representative ^{31}P NMR spectra over time for the reaction of 0.13 mmol calcium imidazole phosphate + 0.325 mmol Ser-His in paste conditions at 50 °C. Top spectrum was taken after evaporation to form a paste.

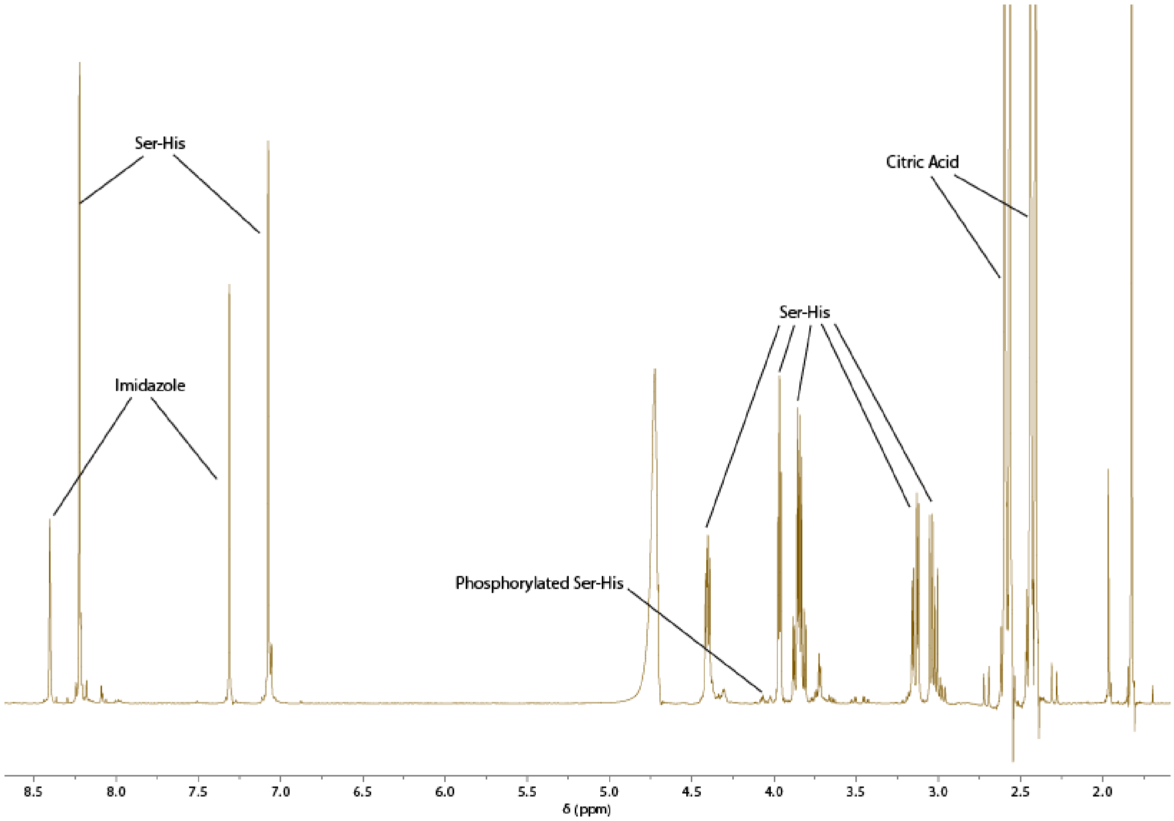

Supplementary Figure 76b: ^{1}H NMR spectrum at 5.0 h for the reaction of 0.13 mmol calcium imidazole phosphate + 0.325 mmol Ser-His in paste conditions at 50 °C.

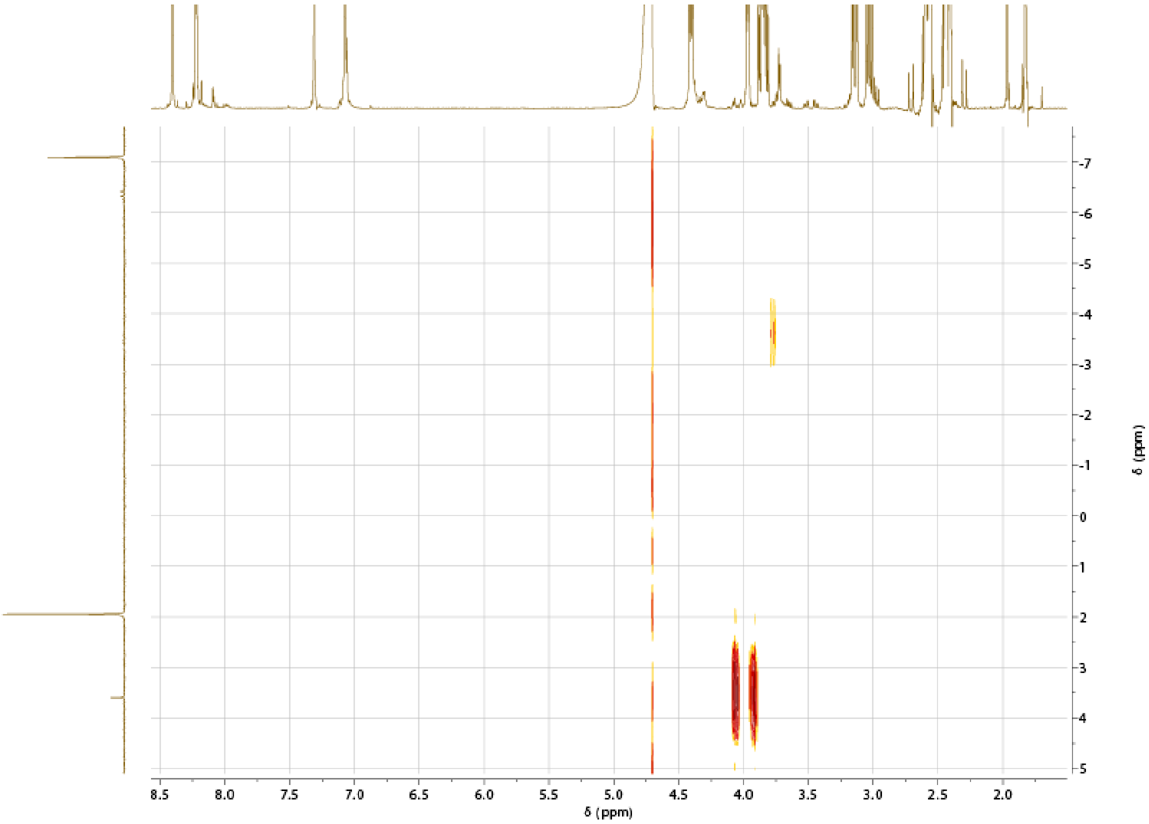

Supplementary Figure 76c: ^{1}H ^{31}P HMBC NMR spectrum at 5.0 h for the reaction of 0.13 mmol calcium imidazole phosphate + 0.325 mmol Ser-His in paste conditions at 50 °C.

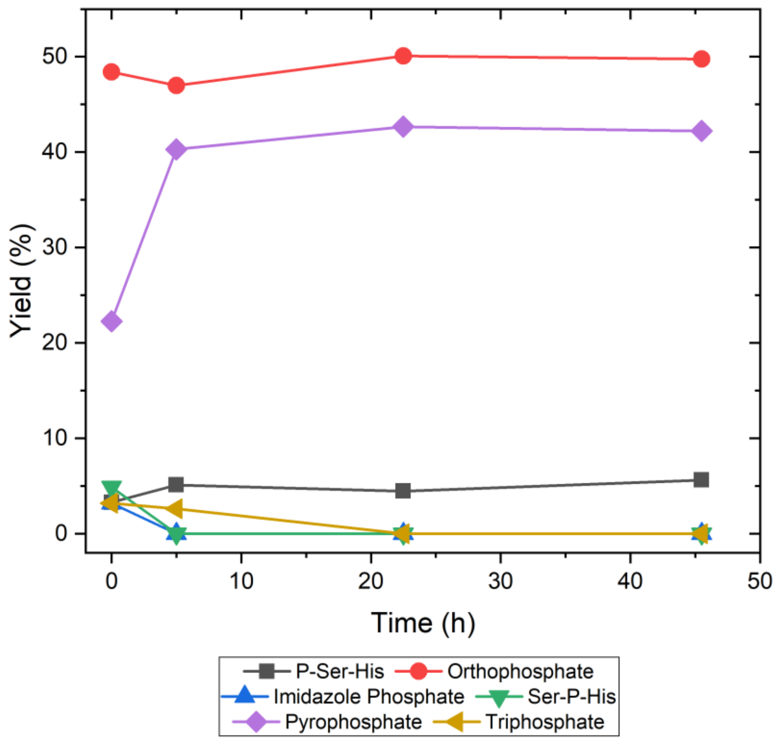

Supplementary Figure 77: Changes in yield over time for the reaction of 0.13 mmol calcium imidazole phosphate + 0.325 mmol Ser-His in paste conditions at 50 °C.

S6.4 Phosphorylation of Metabolites

S6.4.1 Phosphorylation of sodium glycolate

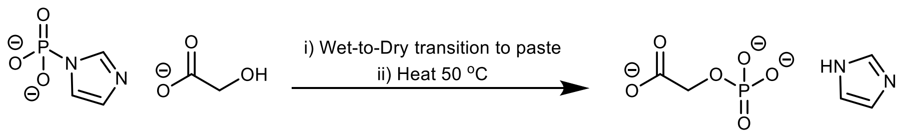

This reaction was performed according to the procedure in Section S6.1 but with the ribose replaced by 63.7 mg (0.65 mmol) sodium glycolate.

Formation of glycolate-2-phosphate occurred in the paste from the reaction between imidazole phosphate and sodium glycolate with the highest observed yield of 31.3 % glycolate-2-phosphate after heating the paste at 50 °C for 24.0 h (Supplementary Figure 78a, 78b, 78c and 79).

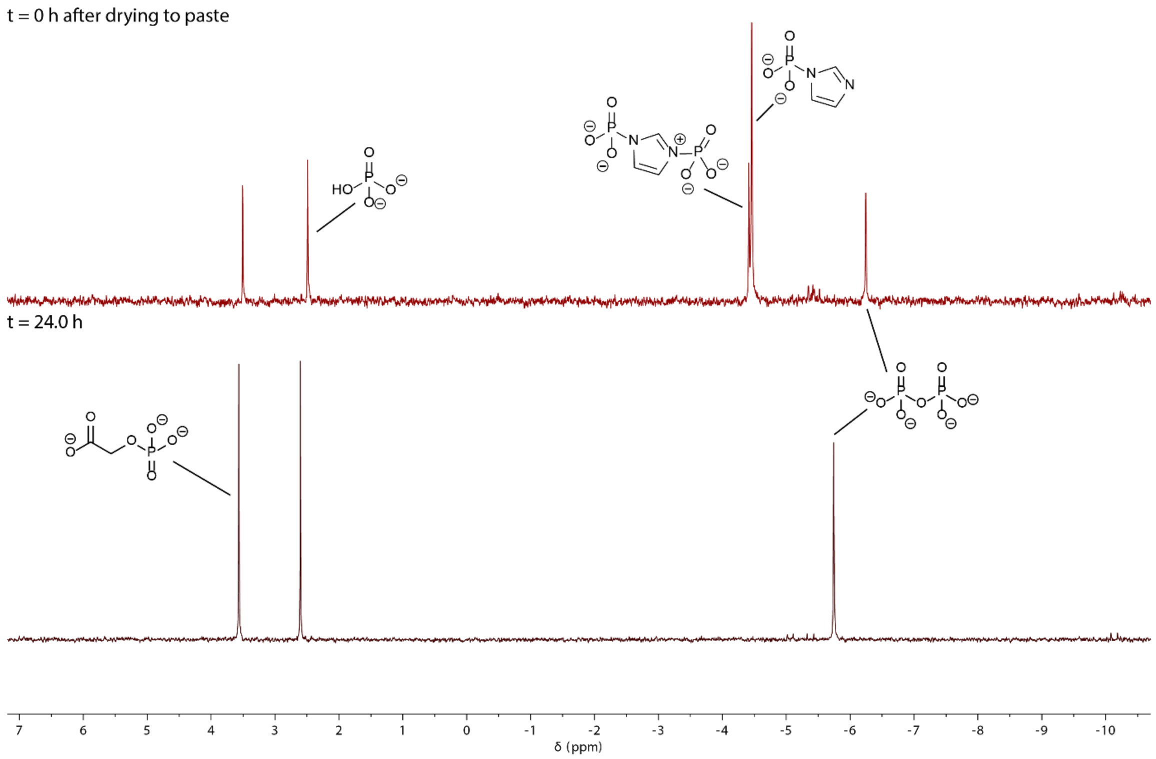

Supplementary Figure 78a: Representative ^{31}P NMR spectra over time for the reaction of 0.13 mmol calcium imidazole phosphate + 0.65 mmol sodium glycolate in paste conditions at 50 °C. Top spectrum was taken after evaporation to form a paste.

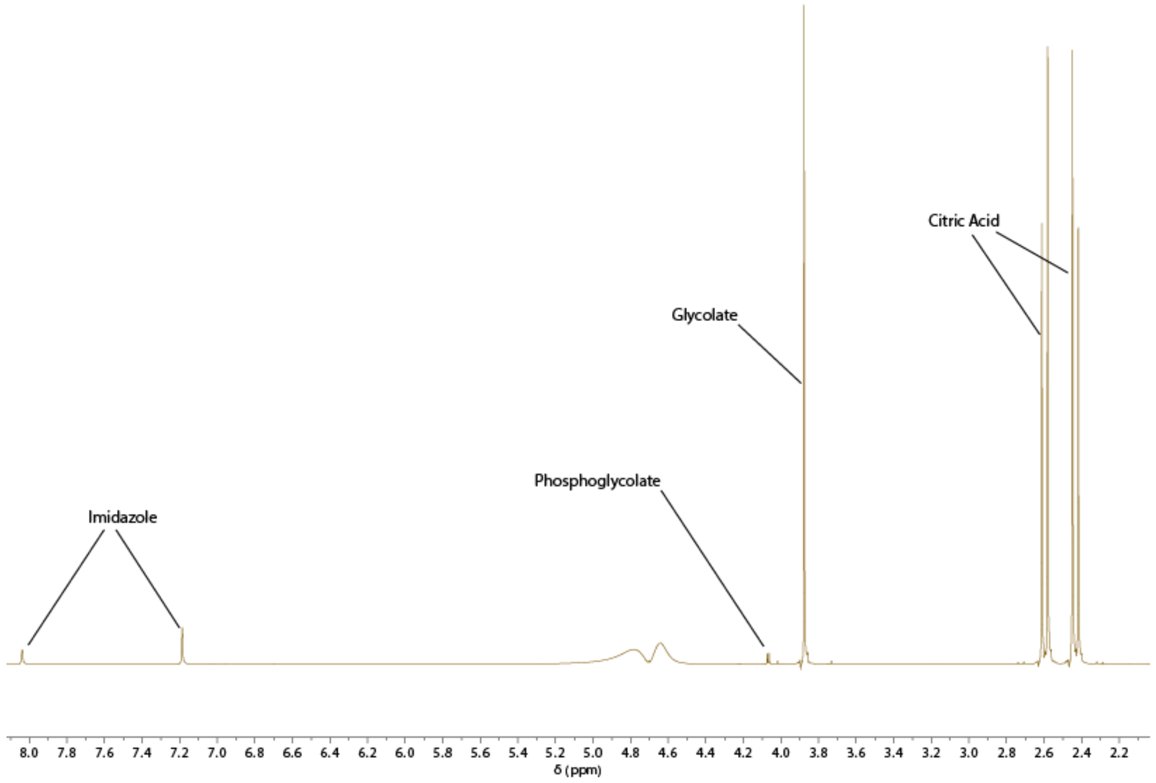

Supplementary Figure 78b: ^{1}H NMR spectrum at 24.0 h for the reaction of 0.13 mmol calcium imidazole phosphate + 0.65 mmol sodium glycolate in paste conditions at 50 °C.

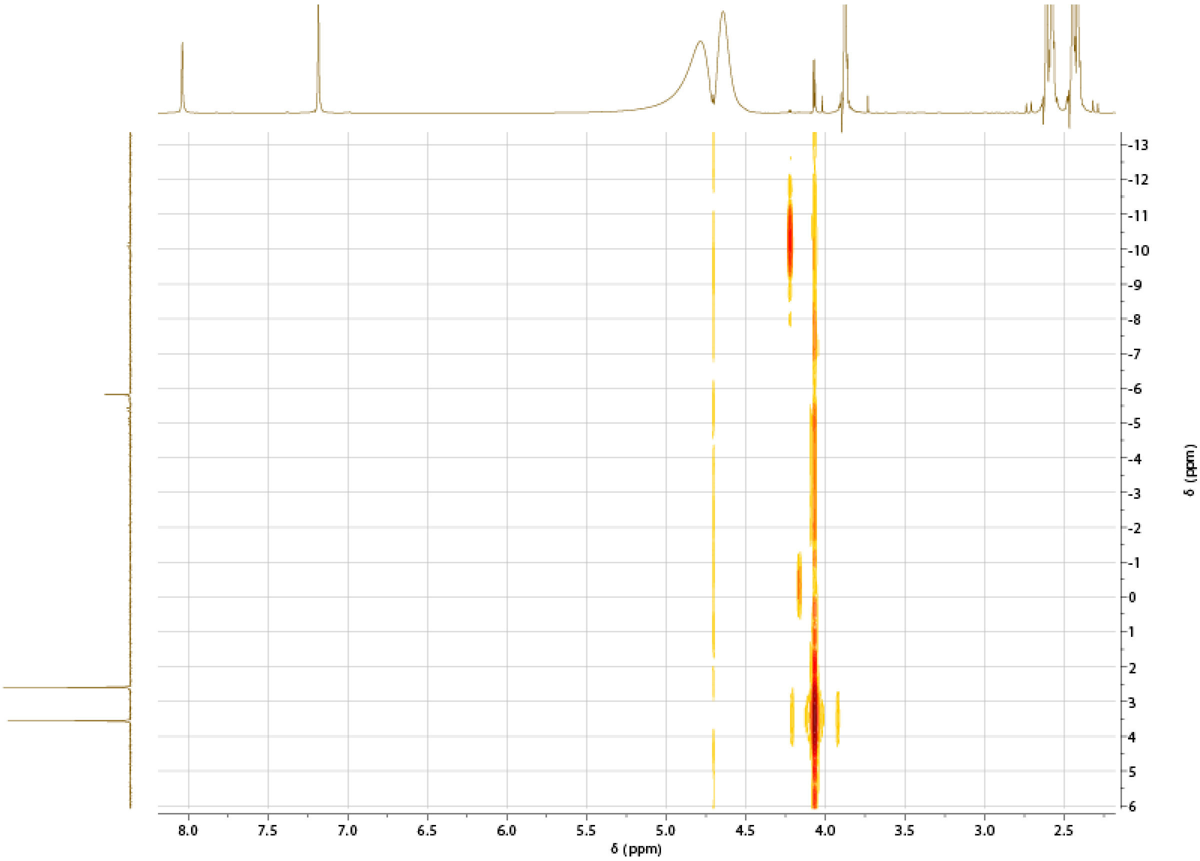

Supplementary Figure 78c: ^{1}H ^{31}P HMBC NMR spectrum at 24.0 h for the reaction of 0.13 mmol calcium imidazole phosphate + 0.65 mmol sodium glycolate in paste conditions at 50 °C.

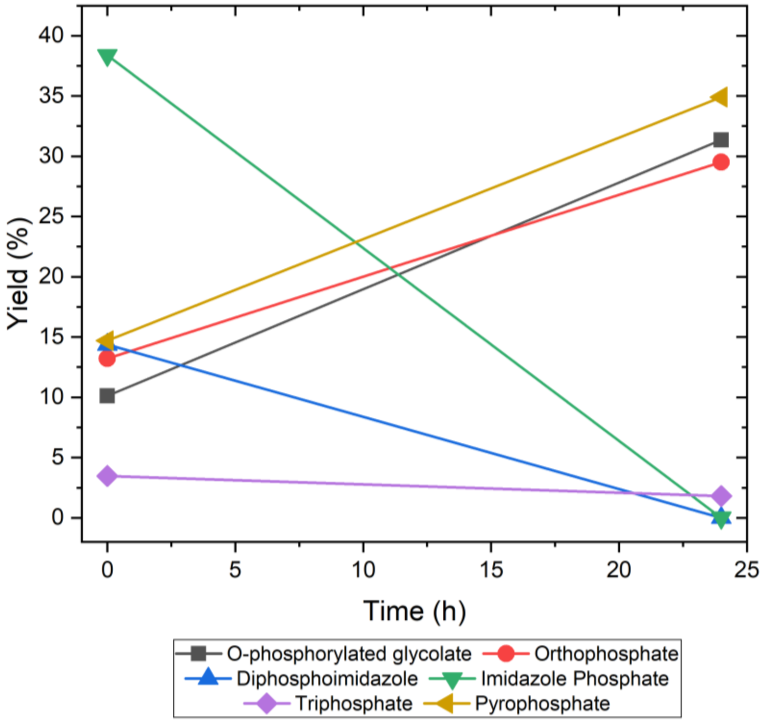

Supplementary Figure 79: Changes in yield over time for the reaction of 0.13 mmol calcium imidazole phosphate + 0.65 mmol sodium glycolate in paste conditions at 50 °C.

A spiking experiment with a genuine sample of glycolate-2-phosphate confirmed their formation from the reaction of calcium imidazole phosphate and sodium glycolate (Supplementary Figure 80).

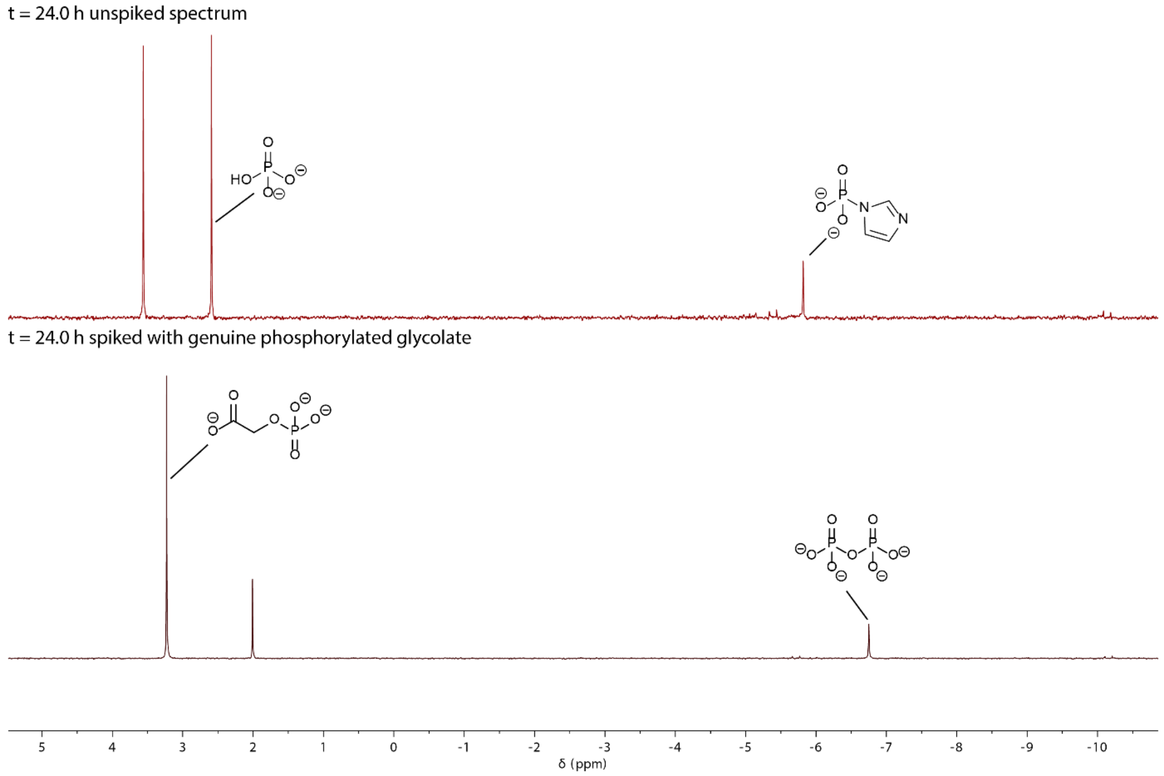

Supplementary Figure 80: ^{31}P NMR spectra of a spiking experiment for the reaction of 0.13 mmol calcium imidazole phosphate + 0.65 mmol sodium glycolate in paste conditions at 50 °C. Top spectrum is unspiked. Bottom spectrum is with the addition of genuine glycolate-2-phosphate.

S6.4.2 Phosphorylation of sodium lactate

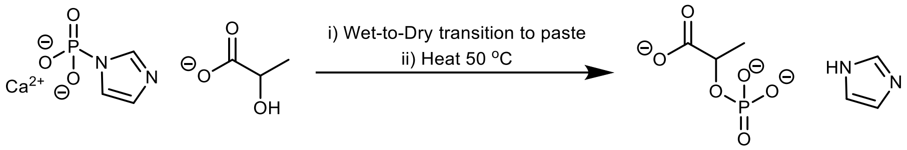

This reaction was performed according to the procedure in Section S6.1 but with the ribose replaced by 72.8 mg (0.65 mmol) sodium lactate.

Formation of lactate-2-phosphate occurred in the paste from the reaction between imidazole phosphate and sodium lactate with the highest observed yield of 10.0 % lactate-2-phosphate after heating the paste at 50 °C for 24.0 h (Supplementary Figure 81a, 81b, 81c and 82).

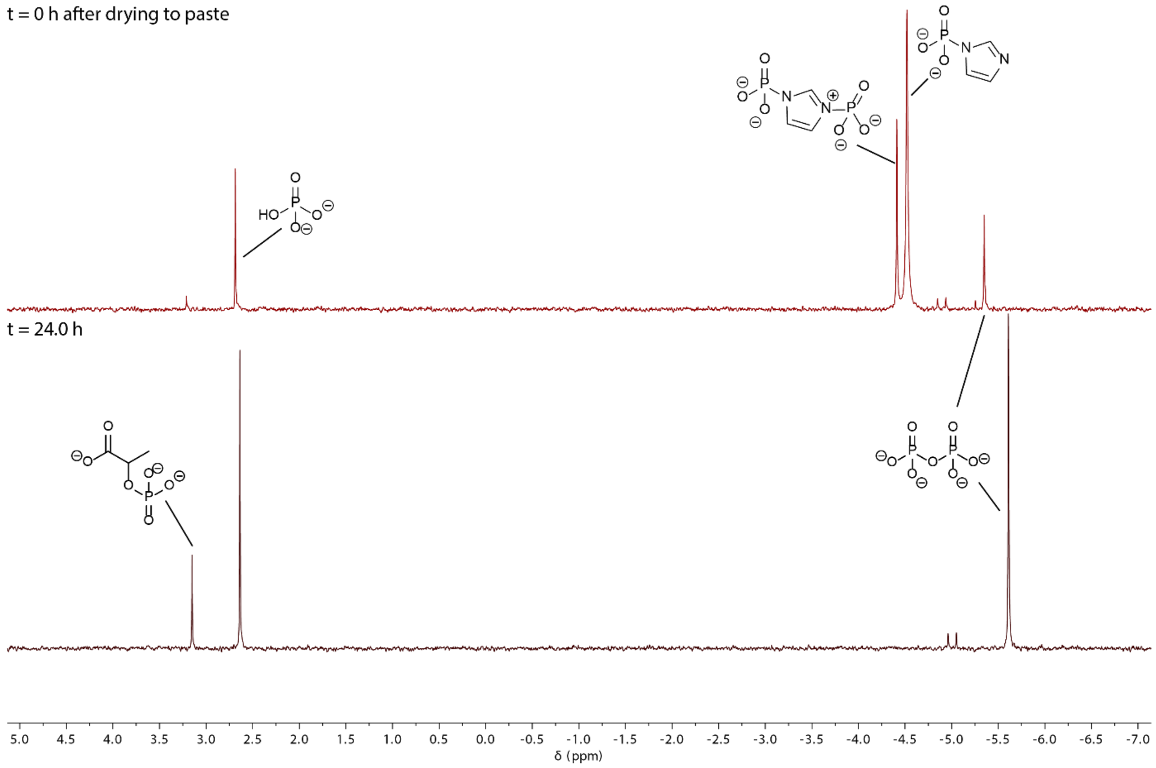

Supplementary Figure 81a: Representative ^{31}P NMR spectra over time for the reaction of 0.13 mmol calcium imidazole phosphate + 0.65 mmol sodium lactate in paste conditions at 50 °C. Top spectrum was taken after evaporation to form a paste.

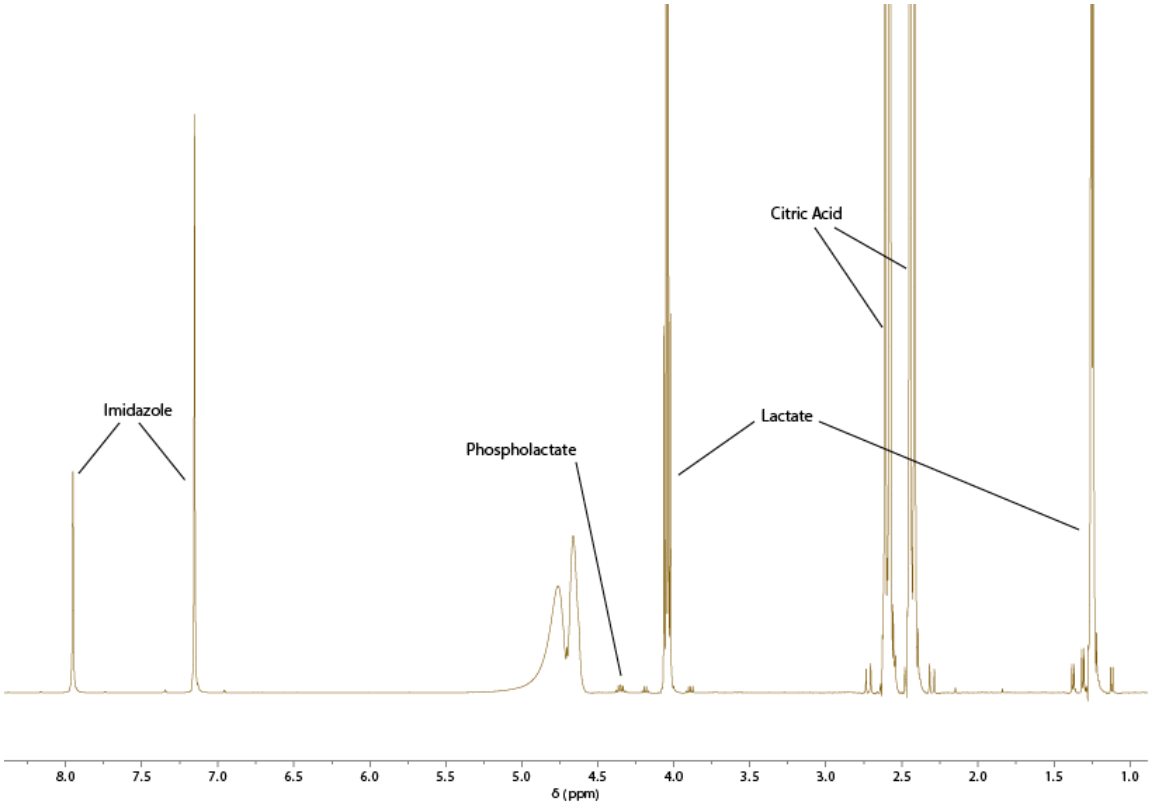

Supplementary Figure 81b: ^{1}H NMR spectrum at 24.0 h for the reaction of 0.13 mmol calcium imidazole phosphate + 0.65 mmol sodium lactate in paste conditions at 50 °C.

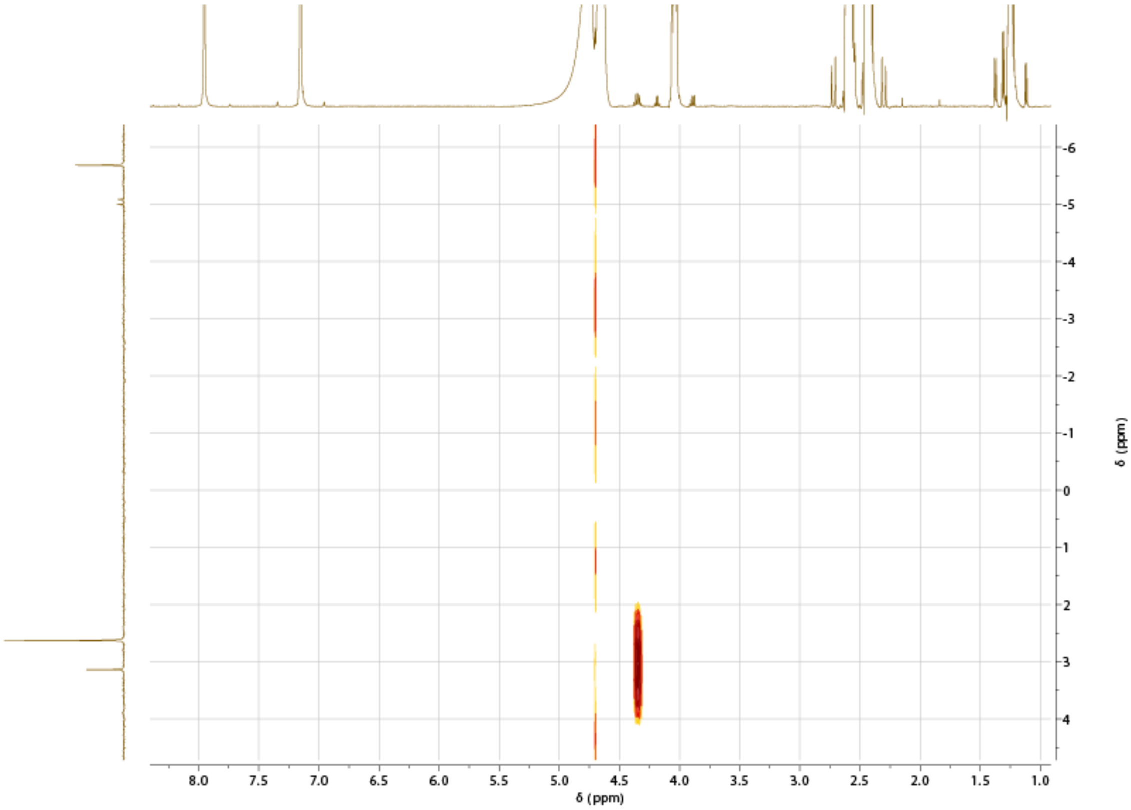

Supplementary Figure 81c: ^{1}H ^{31}P NMR spectrum at 24.0 h for the reaction of 0.13 mmol calcium imidazole phosphate + 0.65 mmol sodium lactate in paste conditions at 50 °C.

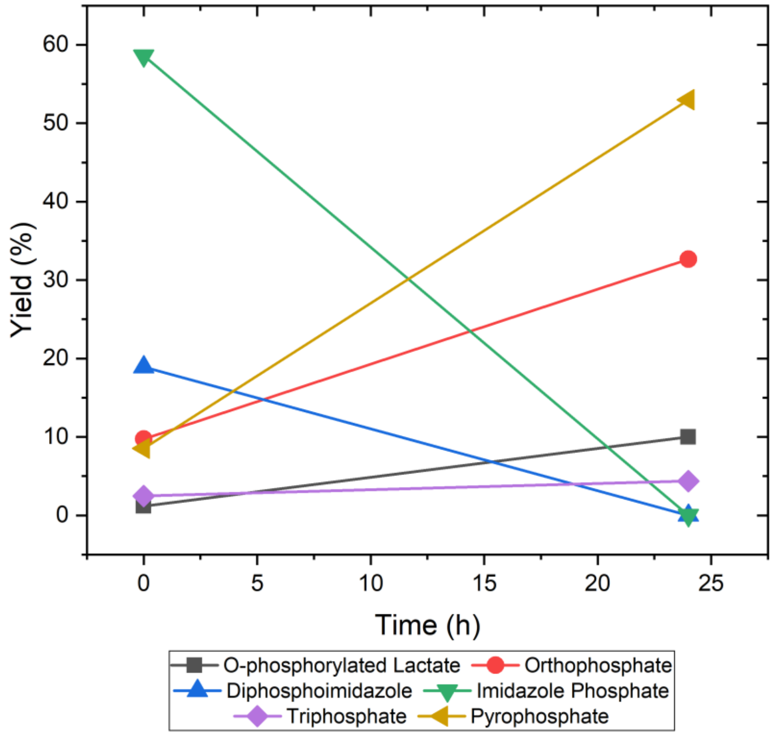

Supplementary Figure 82: Changes in yield over time for the reaction of 0.13 mmol calcium imidazole phosphate + 0.65 mmol sodium lactate in paste conditions at 50 °C.

S6.4.3 Phosphorylation of calcium glycerate

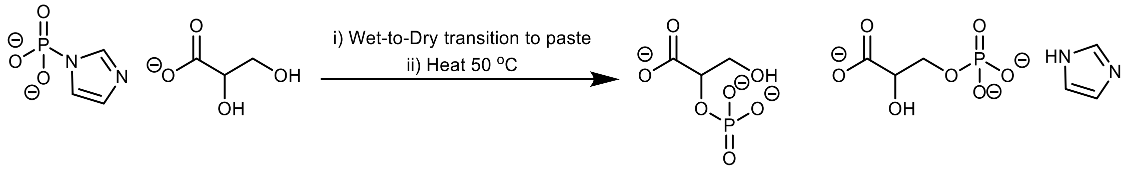

This reaction was performed according to the procedure in Section S6.1 but with the ribose replaced by 186.9 mg (0.75 mmol) calcium glycerate.

Formation of glycerate-2-phosphate and glycerate-3-phosphate occurred in the paste from the reaction between imidazole phosphate and calcium glycerate with the highest observed yield of 26.3 % glycerate-2-phosphate and 7.6 % glycerate-2-phosphate after heating the paste at 50 °C for 167.3 h (Supplementary Figure 83a, 83b, 83c and 84).

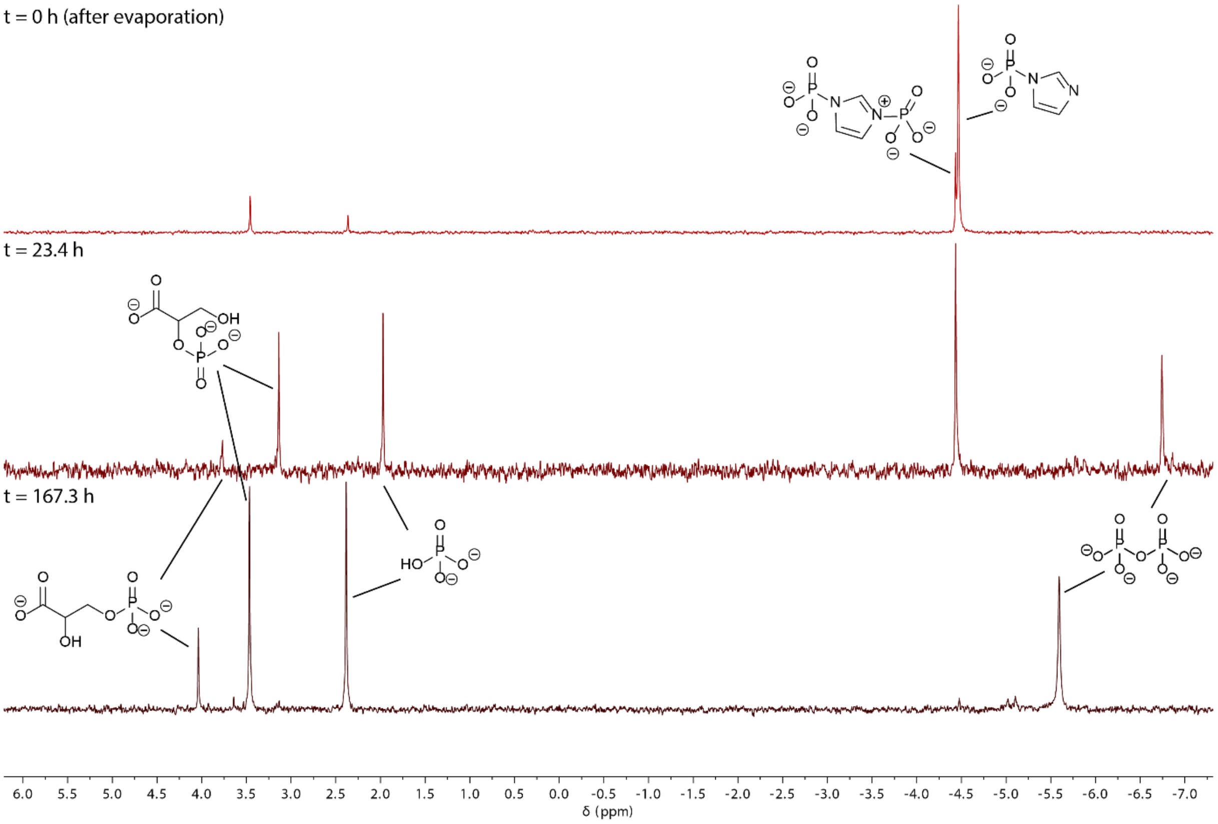

Supplementary Figure 83a: Representative ^{31}P NMR spectra over time for the reaction of 0.13 mmol calcium imidazole phosphate + 0.75 mmol calcium glycerate in paste conditions at 50 °C. Top spectrum was taken after evaporation to form a paste. All NMR spectra were measured in a 0.5 M citric acid buffer at pH 6.85 9 : 1 H_2O : D_2O. The chemical shift of the pyrophosphate peak is variable at pH 6.85 due to the pyrophosphate pK_a = 6.6.

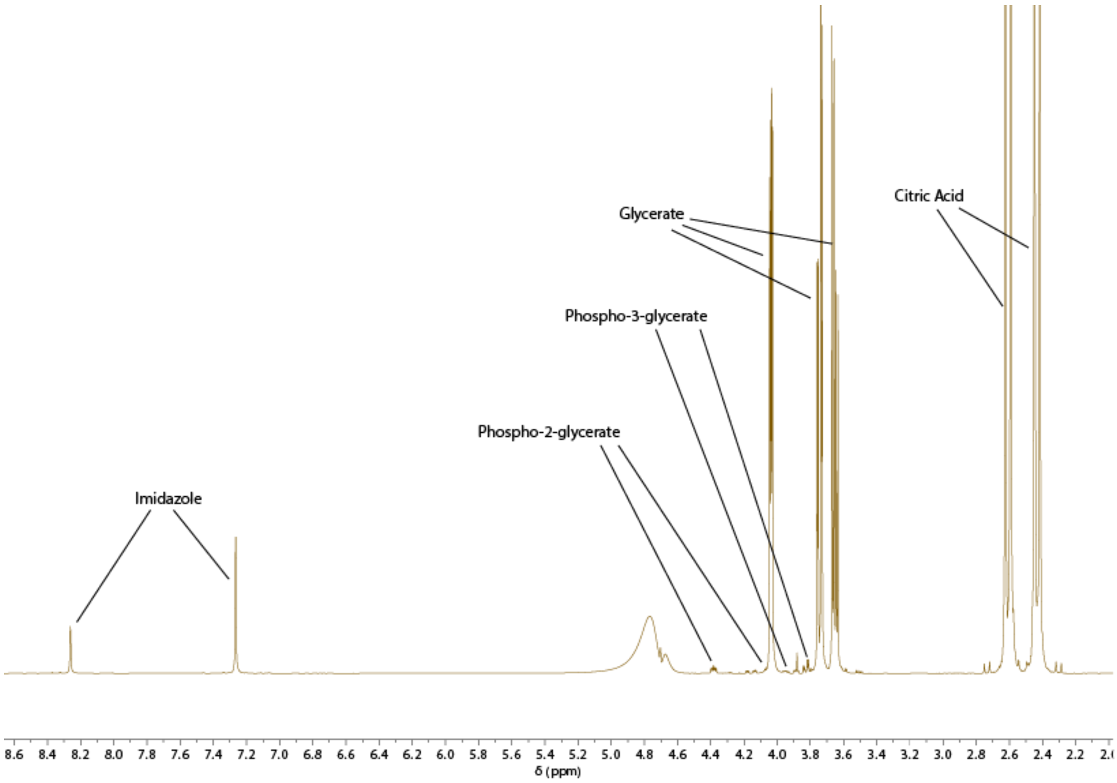

Supplementary Figure 83b: ^{1}H NMR spectrum at 167.3 h for the reaction of 0.13 mmol calcium imidazole phosphate + 0.75 mmol calcium glycerate in paste conditions at 50 °C.

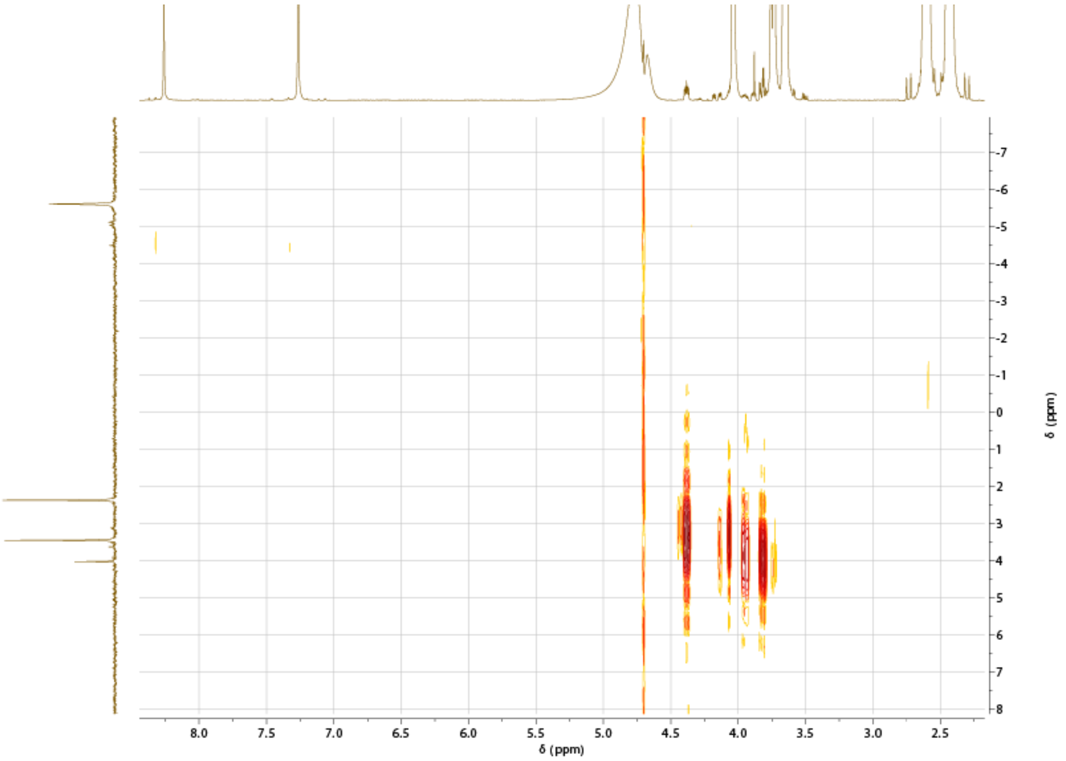

Supplementary Figure 83c: ^{1}H ^{31}P HMBC NMR spectrum at 167.3 h for the reaction of 0.13 mmol calcium imidazole phosphate + 0.75 mmol calcium glycerate in paste conditions at 50 °C.

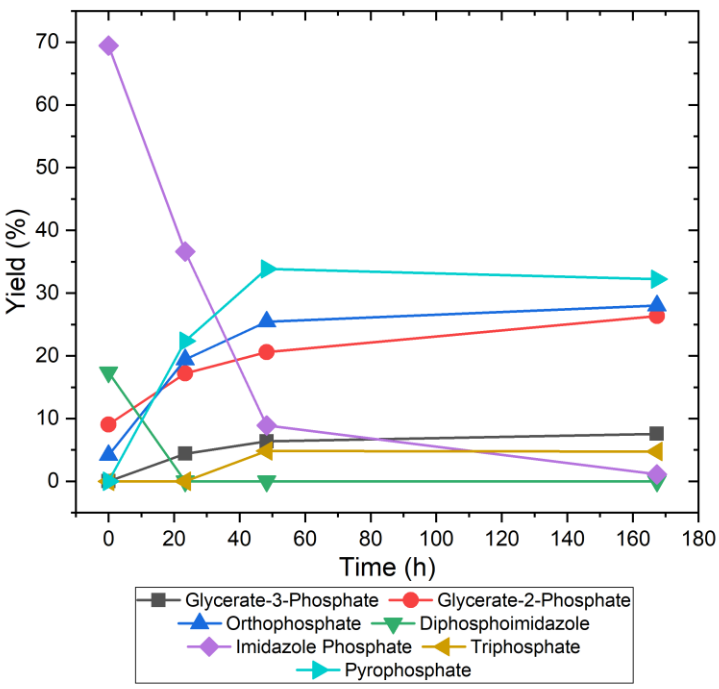

Supplementary Figure 84: Changes in yield over time for the reaction of 0.13 mmol calcium imidazole phosphate + 0.75 mmol calcium glycerate in paste conditions at 50 °C.

A spiking experiment with genuine samples of glycerate-2-phosphate and glycerate-3-phosphate confirmed their formation from the reaction of calcium imidazole phosphate and calcium glycerate (Supplementary Figure 85).

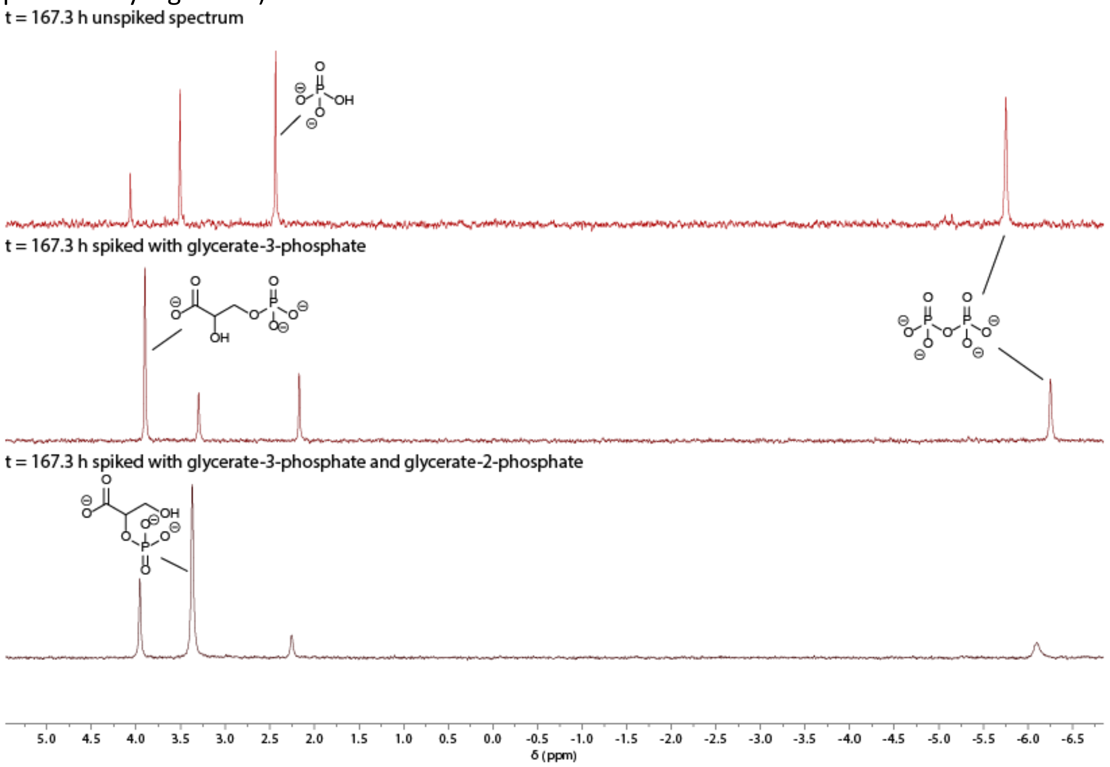

Supplementary Figure 85: ^{31}P NMR spectra of a spiking experiment for the reaction of 0.13 mmol calcium imidazole phosphate + 0.75 mmol calcium glycerate in paste conditions at 50 °C. Top spectrum is unspiked. Middle spectrum is with the addition of genuine glycerate-2-phosphate. Bottom spectrum is with the addition of genuine glycerate-3-phosphate.

S6.4.4 Phosphorylation of sodium glycerate

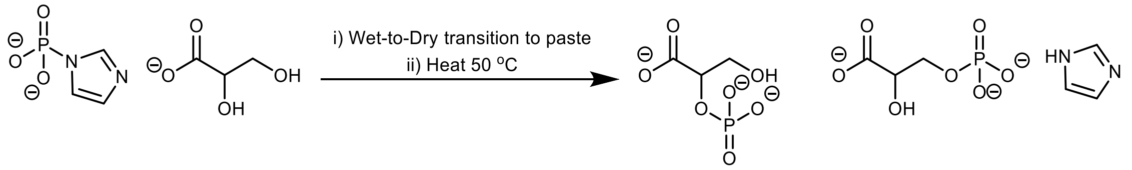

This reaction was performed according to the procedure in Section S6.1 but with the ribose replaced by 67.9 mg (0.65 mmol) sodium glycerate.

Formation of glycerate-2-phosphate and glycerate-3-phosphate occurred in the paste from the reaction between imidazole phosphate and sodium glycerate with the highest observed yield of 15.8 % glycerate-2-phosphate (yield of glycerate-3-phosphate = 7.4 %) after heating paste at 50 °C for 23.4 h and the highest observed yield of 8.6 % glycerate-3-phosphate (yield of glycerate-2-phosphate = 14.7 %) after heating the paste at 50 °C for 167.3 h (Supplementary Figure 86a, 86b, 86c and 87).

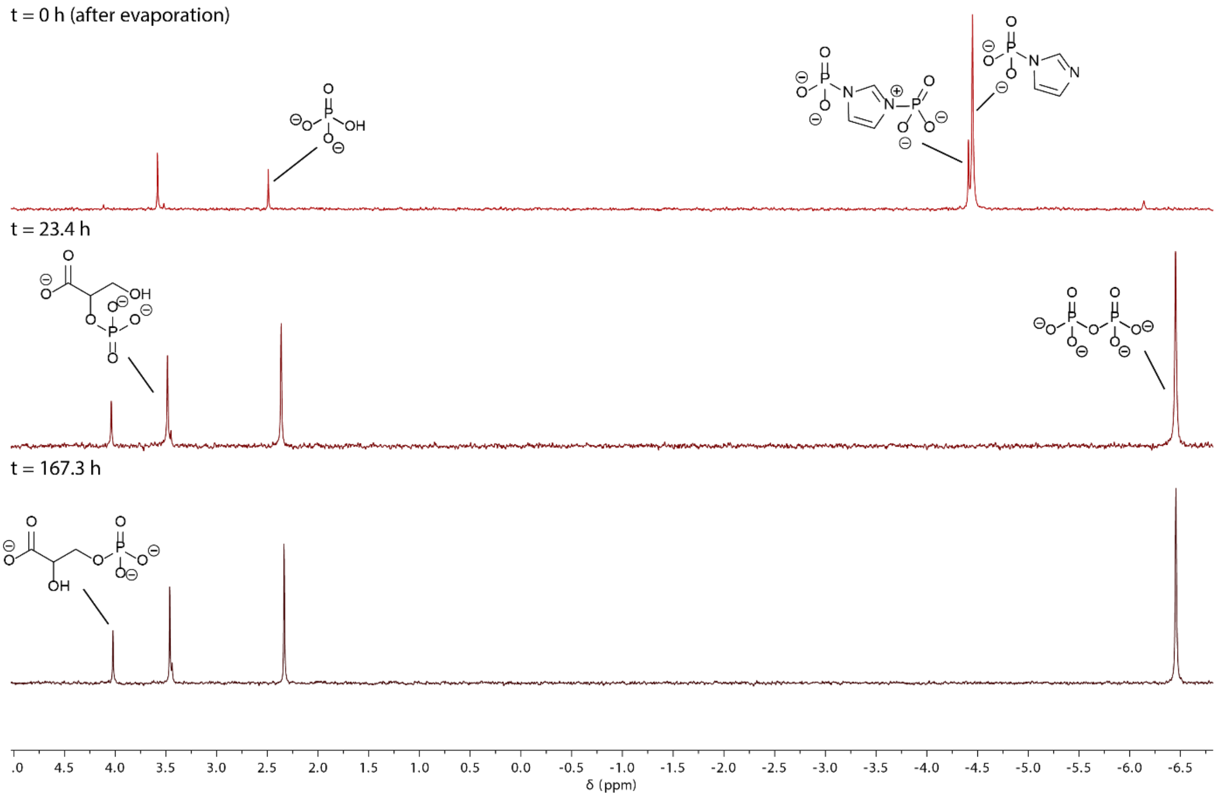

Supplementary Figure 86a: Representative ^{31}P NMR spectra over time for the reaction of 0.13 mmol calcium imidazole phosphate + 0.65 mmol sodium glycerate in paste conditions at 50 °C. Top spectrum was taken after evaporation to form a paste.

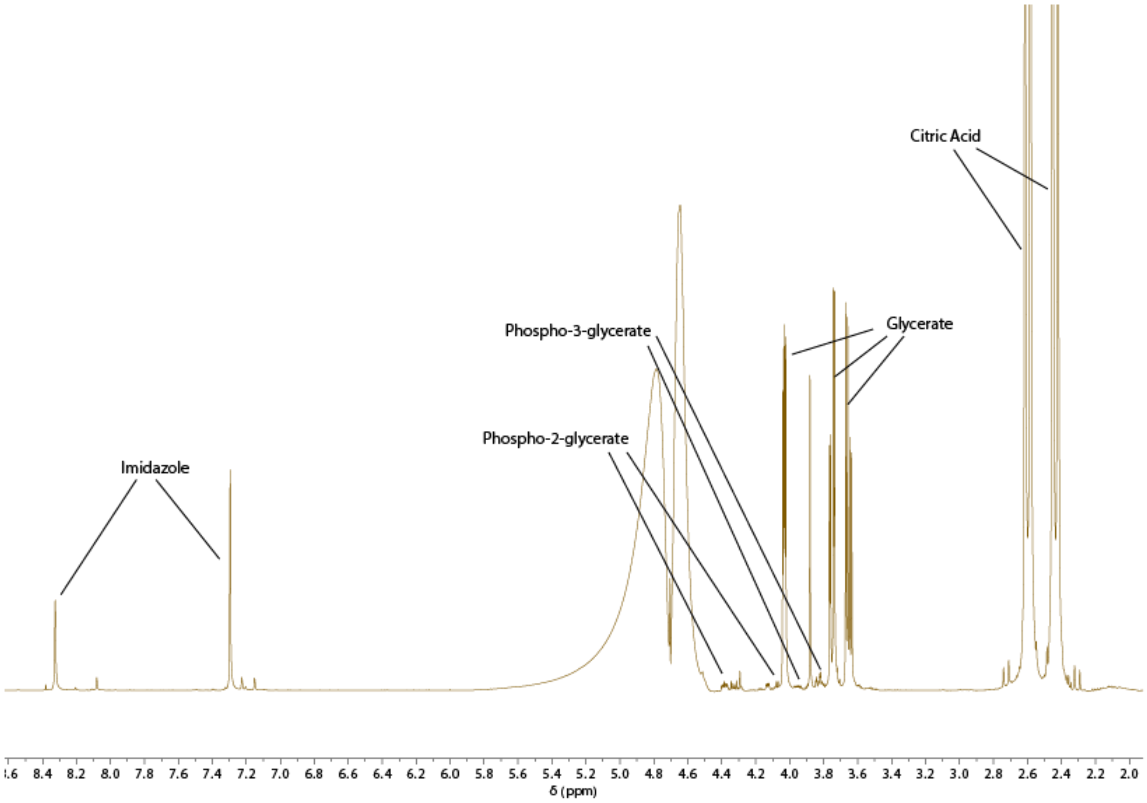

Supplementary Figure 86b: ^{1}H NMR spectrum at 167.3 h for the reaction of 0.13 mmol calcium imidazole phosphate + 0.65 mmol sodium glycerate in paste conditions at 50 °C.

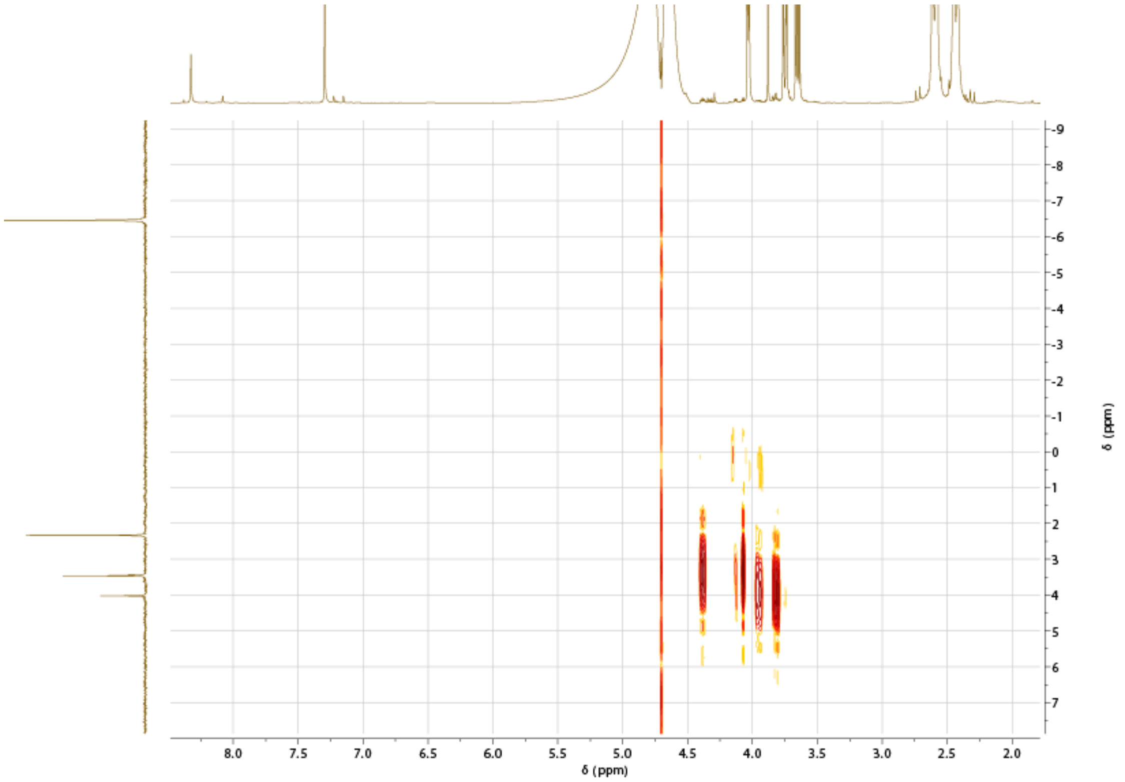

Supplementary Figure 86c: ^{1}H ^{31}P HMBC NMR spectrum at 167.3 h for the reaction of 0.13 mmol calcium imidazole phosphate + 0.65 mmol sodium glycerate in paste conditions at 50 °C.

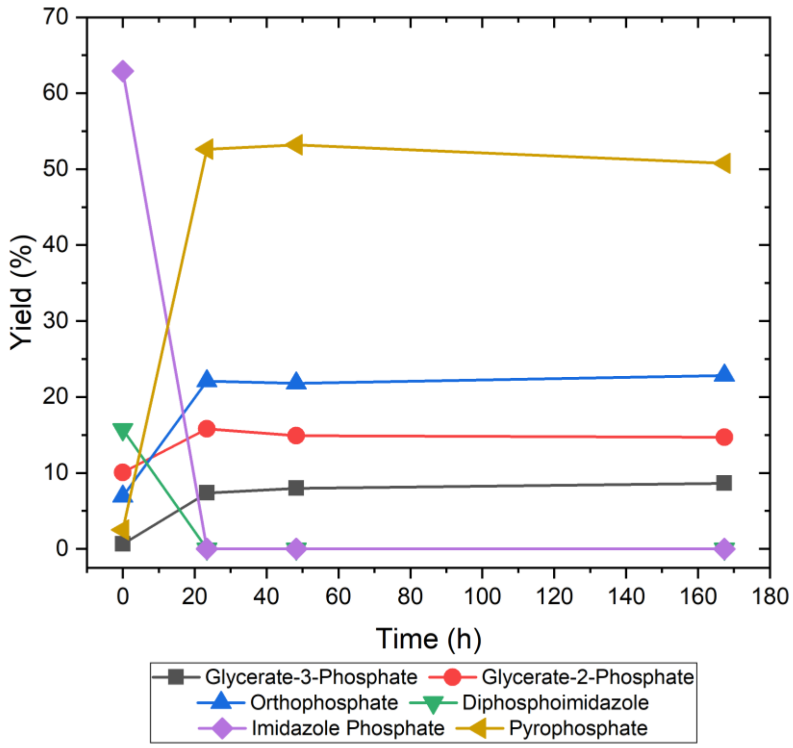

Supplementary Figure 87: Changes in yield over time for the reaction of 0.13 mmol calcium imidazole phosphate + 0.65 mmol sodium glycerate in paste conditions at 50 °C.

A spiking experiment with genuine samples of glycerate-2-phosphate and glycerate-3-phosphate confirmed their formation from the reaction of calcium imidazole phosphate and sodium glycerate (Supplementary Figure 88).

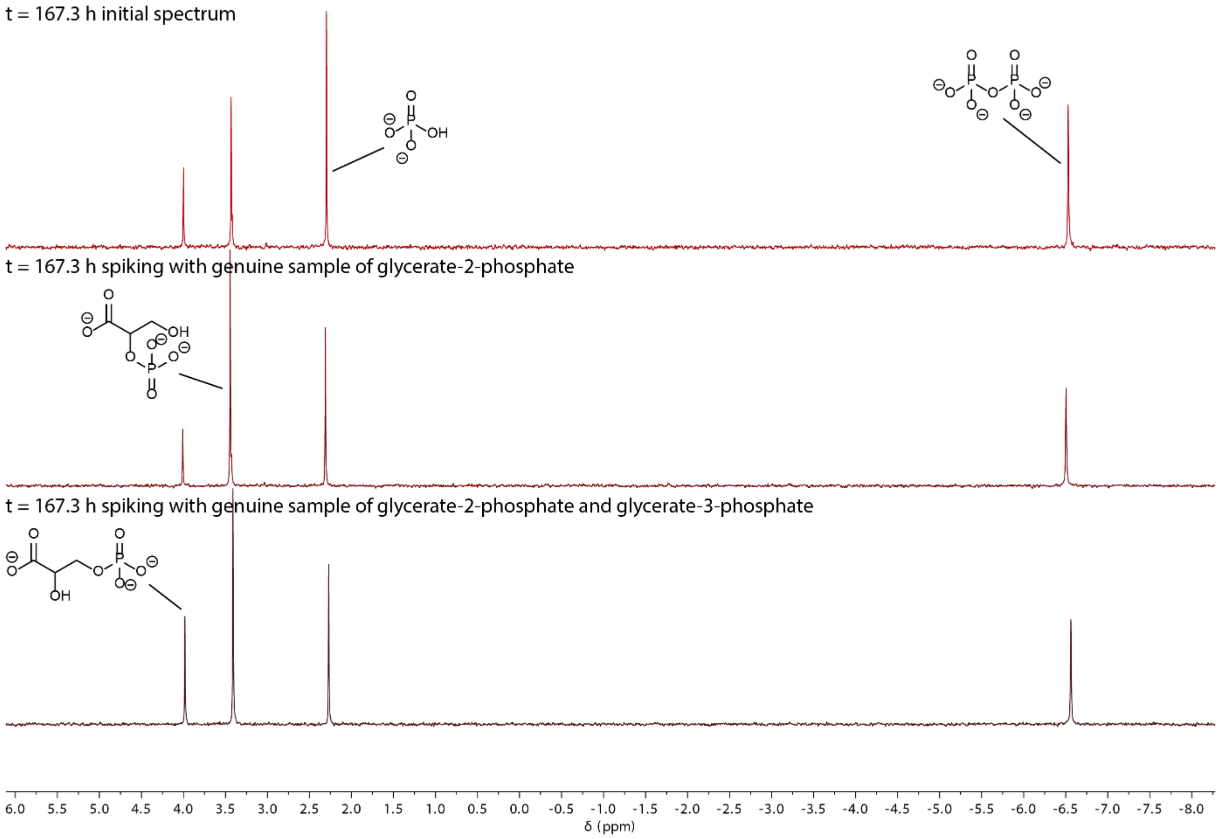

Supplementary Figure 88: ^{31}P NMR spectra of a spiking experiment for the reaction of 0.13 mmol calcium imidazole phosphate + 0.65 mmol sodium glycerate in paste conditions at 50 °C. Top spectrum is unspiked. Middle spectrum is with the addition of genuine glycerate-2-phosphate. Bottom spectrum is with the addition of genuine glycerate-3-phosphate.

S6.4.5 Phosphorylation of lactamide

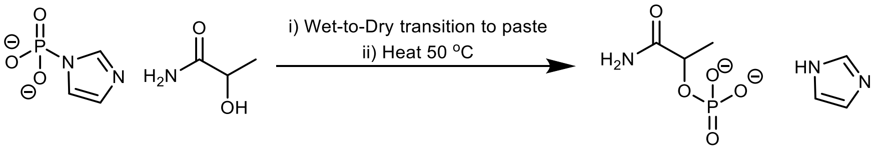

This reaction was performed according to the procedure in Section S6.1 but with the ribose replaced by 58.6 mg (0.65 mmol) lactamide.

Formation of phosphorylated lactamide occurred in the paste from the reaction between imidazole phosphate and lactamide with the highest observed yield of 11.1 % after heating the paste at 50 °C for 167.3 h (Supplementary Figure 89a, 89b, 89c and 90).

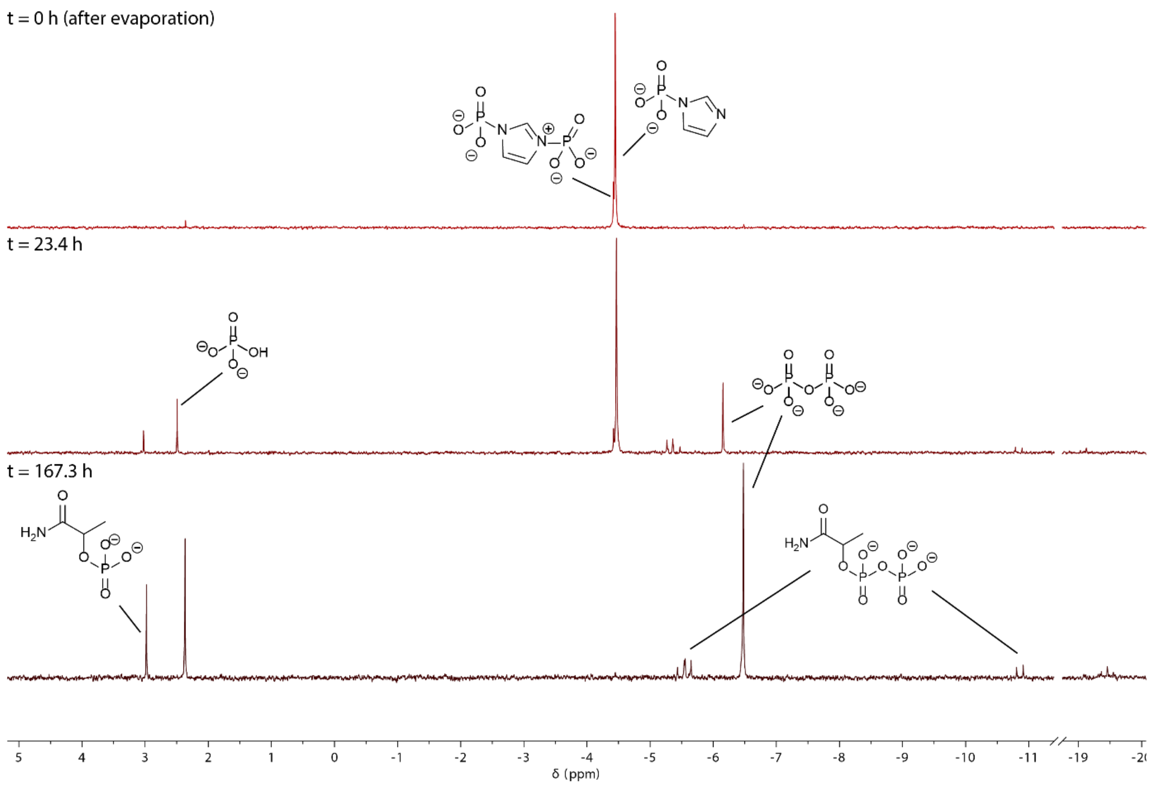

Supplementary Figure 89a: Representative ^{31}P NMR spectra over time for the reaction of 0.13 mmol calcium imidazole phosphate + 0.65 mmol lactamide in paste conditions at 50 °C. Top spectrum was taken after evaporation to form a paste. All NMR spectra were measured in a 0.5 M citric acid buffer at pH 6.85 9 : 1 H_2O : D_2O. The chemical shift of the pyrophosphate peak is variable at pH 6.85 due to the pyrophosphate pK_a = 6.6.

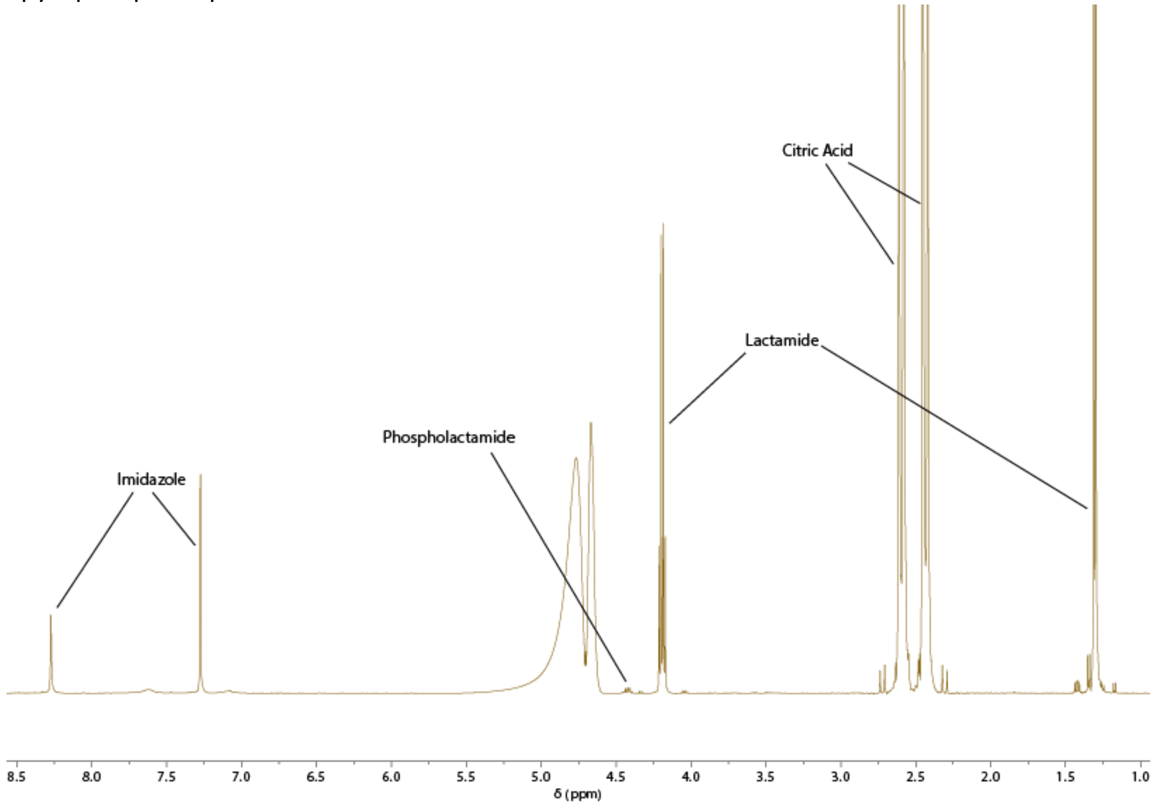

Supplementary Figure 89b: ^{1}H NMR spectrum at 167.3 h for the reaction of 0.13 mmol calcium imidazole phosphate + 0.65 mmol lactamide in paste conditions at 50 °C.

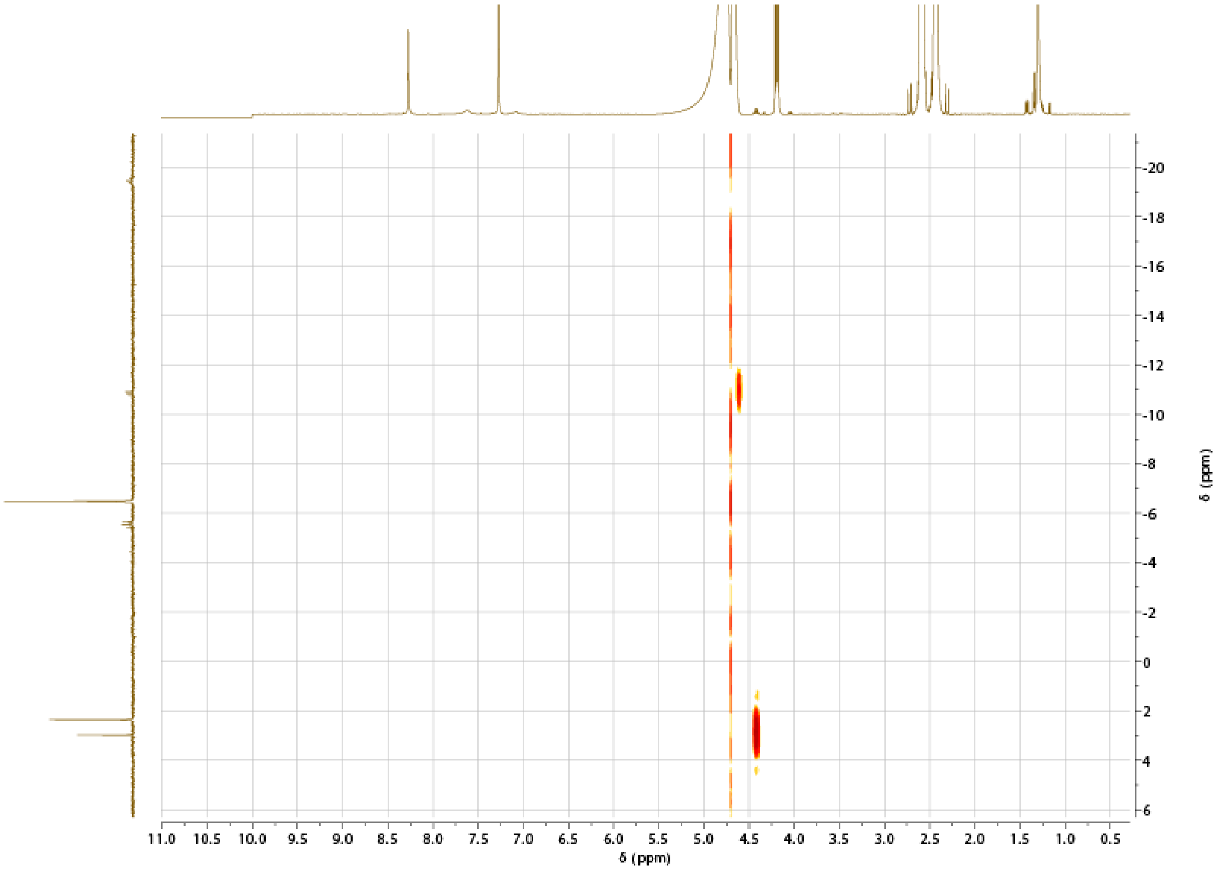

Supplementary Figure 89c: ^{1}H ^{31}P HMBC NMR spectrum at 167.3 h for the reaction of 0.13 mmol calcium imidazole phosphate + 0.65 mmol lactamide in paste conditions at 50 °C.

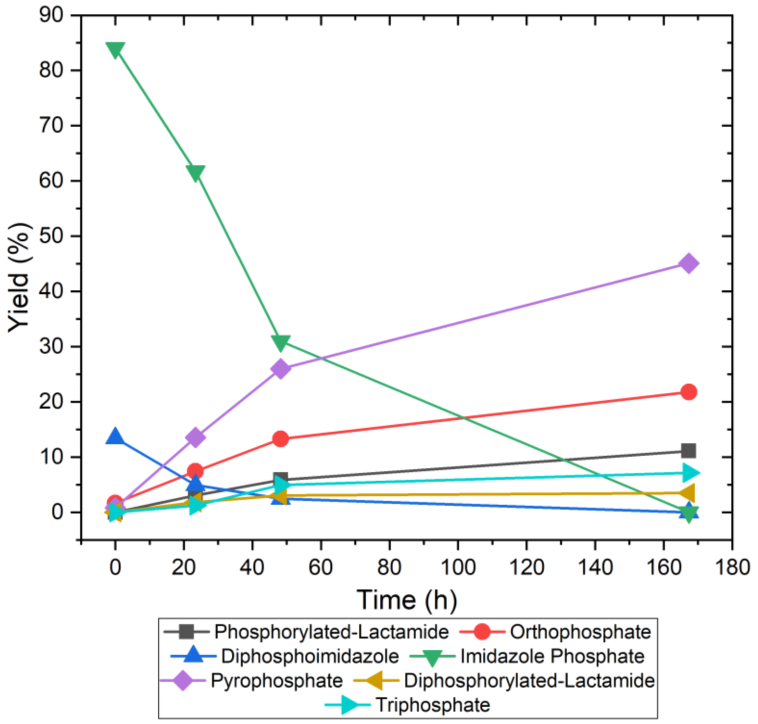

Supplementary Figure 90: Changes in yield over time for the reaction of 0.13 mmol calcium imidazole phosphate + 0.65 mmol lactamide in paste conditions at 50 °C.

S6.5 Phosphorylation of Nucleosides

S6.5.1 Phosphorylation of adenosine

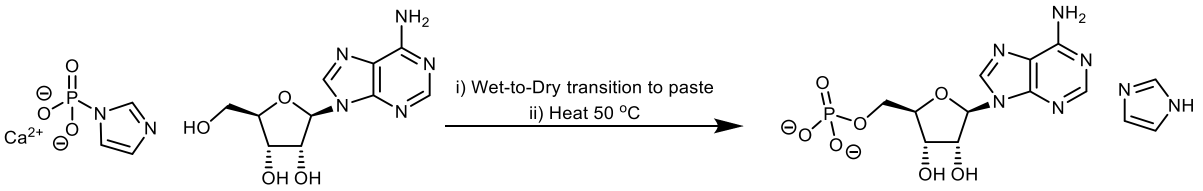

This reaction was performed according to the procedure in Section S6.1 but with the ribose replaced by 200.9 mg (0.65 mmol) adenosine.

Formation of adenosine-5′-monophosphate occurred in the paste from the reaction between imidazole phosphate and adenosine with the highest observed yield of 1.8 % after heating the paste at 50 °C for 22.4 h (Supplementary Figure 91 and 92).

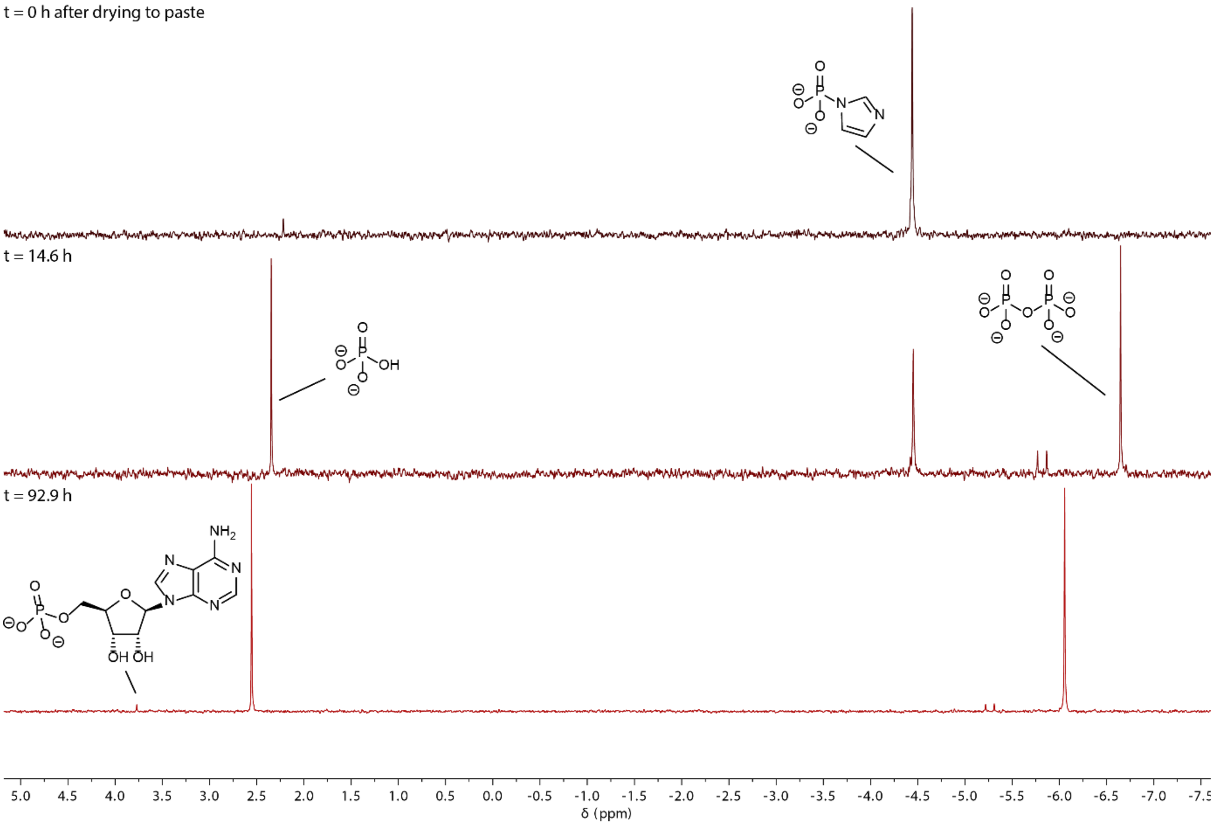

Supplementary Figure 91: Representative ^{31}P NMR spectra over time for the reaction of 0.13 mmol calcium imidazole phosphate + 0.65 mmol adenosine in paste conditions at 50 °C. Top spectrum was taken after evaporation to form a paste. All NMR spectra were measured in a 0.5 M citric acid buffer at pH 6.85 9 : 1 H_2O : D_2O. The chemical shift of the pyrophosphate peak is variable at pH 6.85 due to the pyrophosphate pK_a = 6.6.

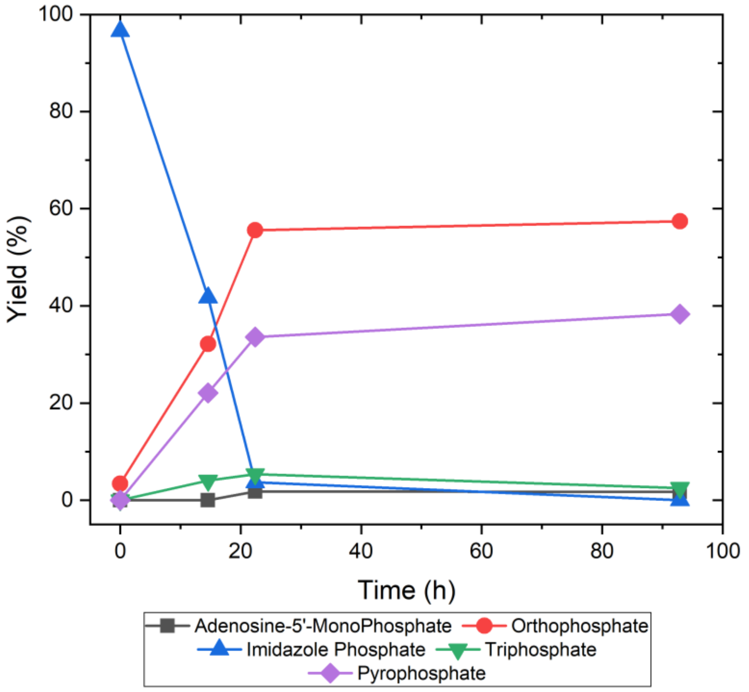

Supplementary Figure 92: Changes in yield over time for the reaction of 0.13 mmol calcium imidazole phosphate + 0.65 mmol adenosine in paste conditions at 50 °C.

A spiking experiment with a genuine sample of adenosine-5’-monophosphate confirmed the formation of adenosine-5’-monophosphate from the reaction of calcium imidazole phosphate and adenosine (Supplementary Figure 93).

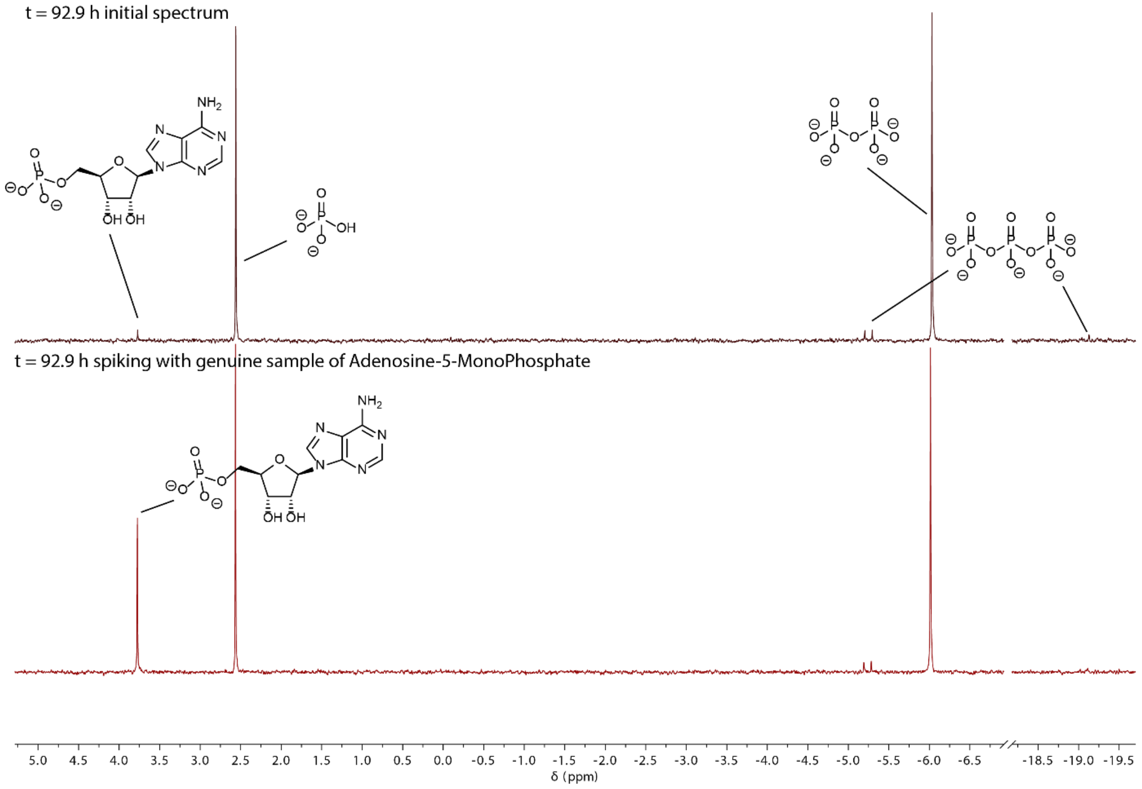

Supplementary Figure 93: ^{31}P NMR spectra of a spiking experiment for the reaction of 0.13 mmol calcium imidazole phosphate + 0.65 mmol adenosine in paste conditions at 50 °C. Top spectrum is unspiked. Bottom spectrum is with the addition of genuine adenosine-5’-monophosphate.

S6.5.2 Phosphorylation of cytidine

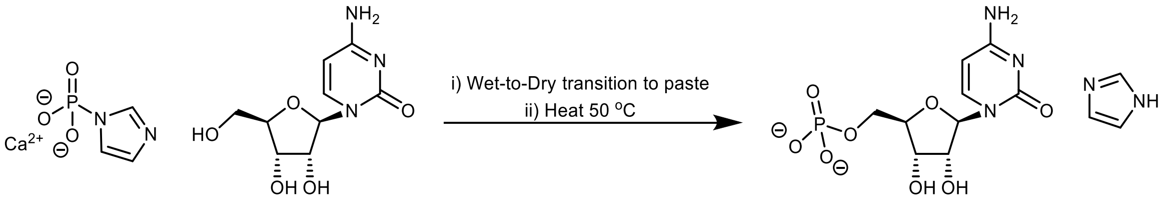

This reaction was performed according to the procedure in Section S6.1 but with the ribose replaced by 158.5 mg (0.65 mmol) cytidine.

Formation of cytidine-5’-monophosphate occurred in the paste from the reaction between imidazole phosphate and cytidine with the highest observed yield of 6.7 % yield cytidine-2’/3’-monophosphate, 3.6 % yield cytidine-3’/2’-monophosphate and 2.6 % yield cytidine-5'-monophosphate after heating the paste at 50 °C for 92.9 h (Supplementary Figure 94 and 95).

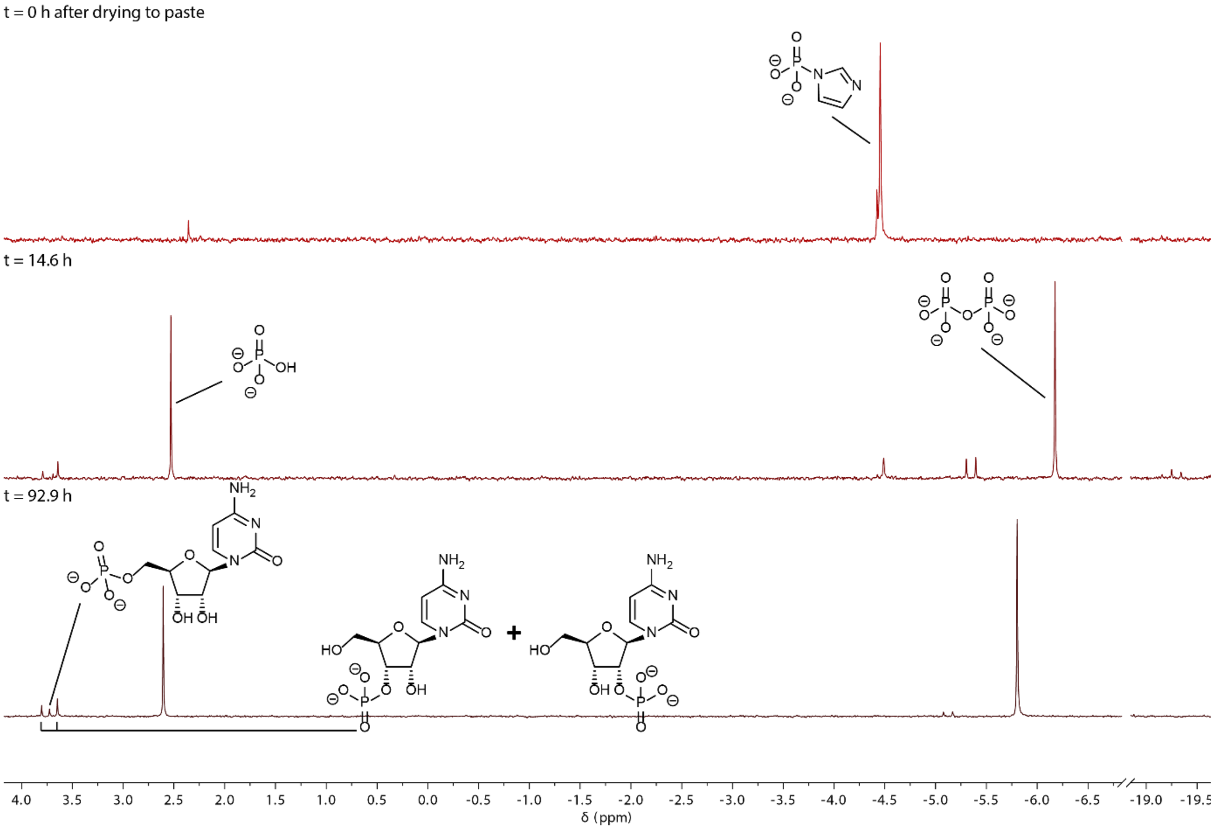

Supplementary Figure 94: Representative ^{31}P NMR spectra over time for the reaction of 0.13 mmol calcium imidazole phosphate + 0.65 mmol cytidine in paste conditions at 50 °C. Top spectrum was taken after evaporation to form a paste. All NMR spectra were measured in a 0.5 M citric acid buffer at pH 6.85 9 : 1 H_2O : D_2O. The chemical shift of the pyrophosphate peak is variable at pH 6.85 due to the pyrophosphate pK_a = 6.6.

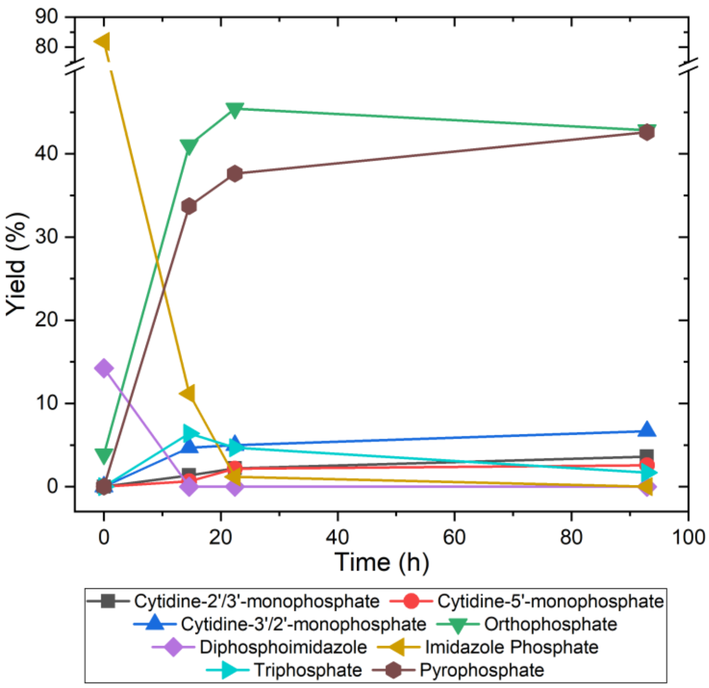

Supplementary Figure 95: Changes in yield over time for the reaction of 0.13 mmol calcium imidazole phosphate + 0.65 mmol cytidine in paste conditions at 50 °C.

A spiking experiment with a genuine sample of cytidine-5'-monophosphate confirmed the formation of cytidine-5'-monophosphate from the reaction of calcium imidazole phosphate and cytidine (Supplementary Figure 96).

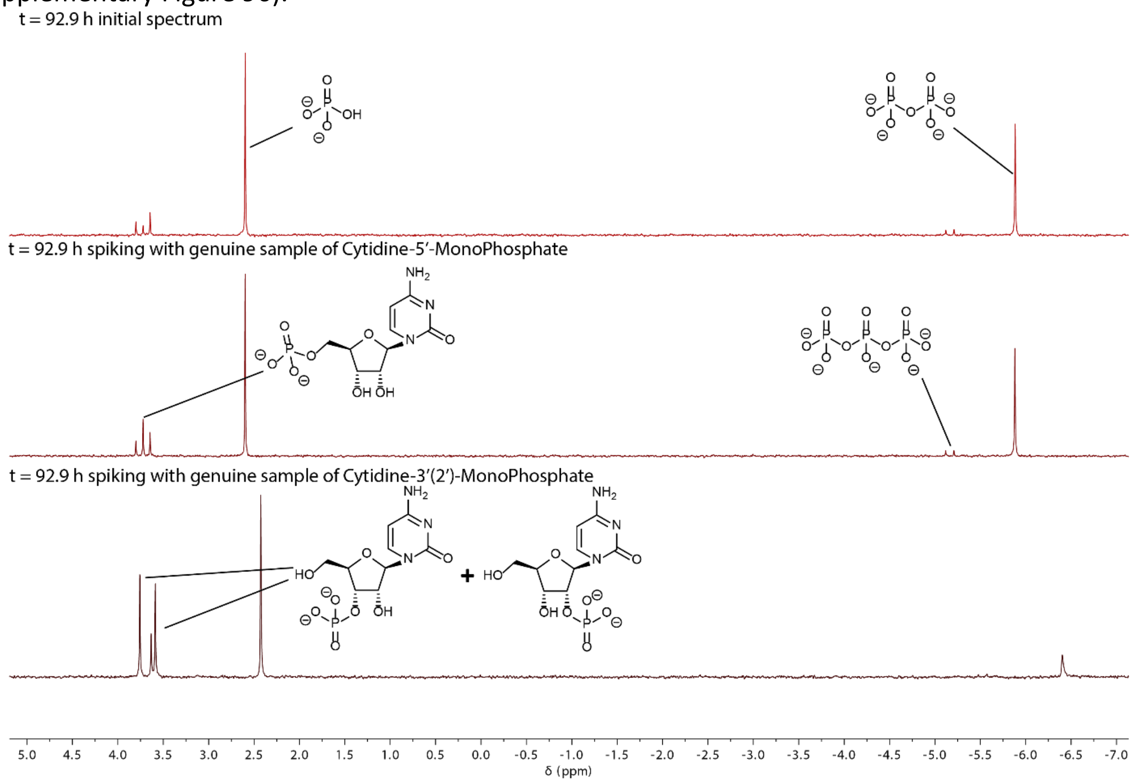

Supplementary Figure 96: ^{31}P NMR spectra of a spiking experiment for the reaction of 0.13 mmol calcium imidazole phosphate + 0.65 mmol cytidine in paste conditions at 50 °C. Top spectrum is unspiked. Middle spectrum is with the addition of genuine cytidine-5'-monophosphate. Bottom spectrum is with the addition of genuine cytidine-3'(2')-monophosphate.

S6.5.3 Phosphorylation of guanosine

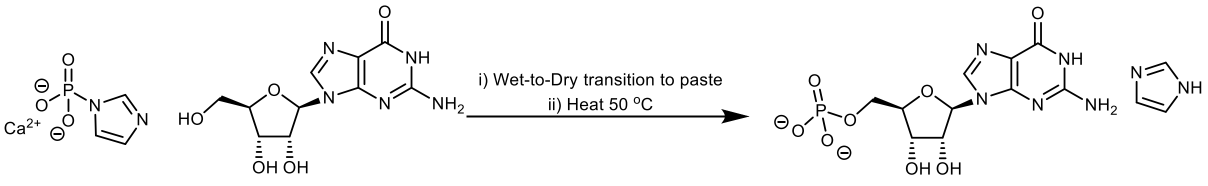

This reaction was performed according to the procedure in Section S6.1 but with the ribose replaced by 212.0 mg (0.65 mmol) guanosine.

Formation of guanosine-5′-monophosphate occurred in the paste from the reaction between imidazole phosphate and guanosine with the highest observed yield of 1.1 % yield guanosine-5'-monophosphate after heating the paste at 50 °C for 92.9 h (Supplementary Figure 97 and 98).

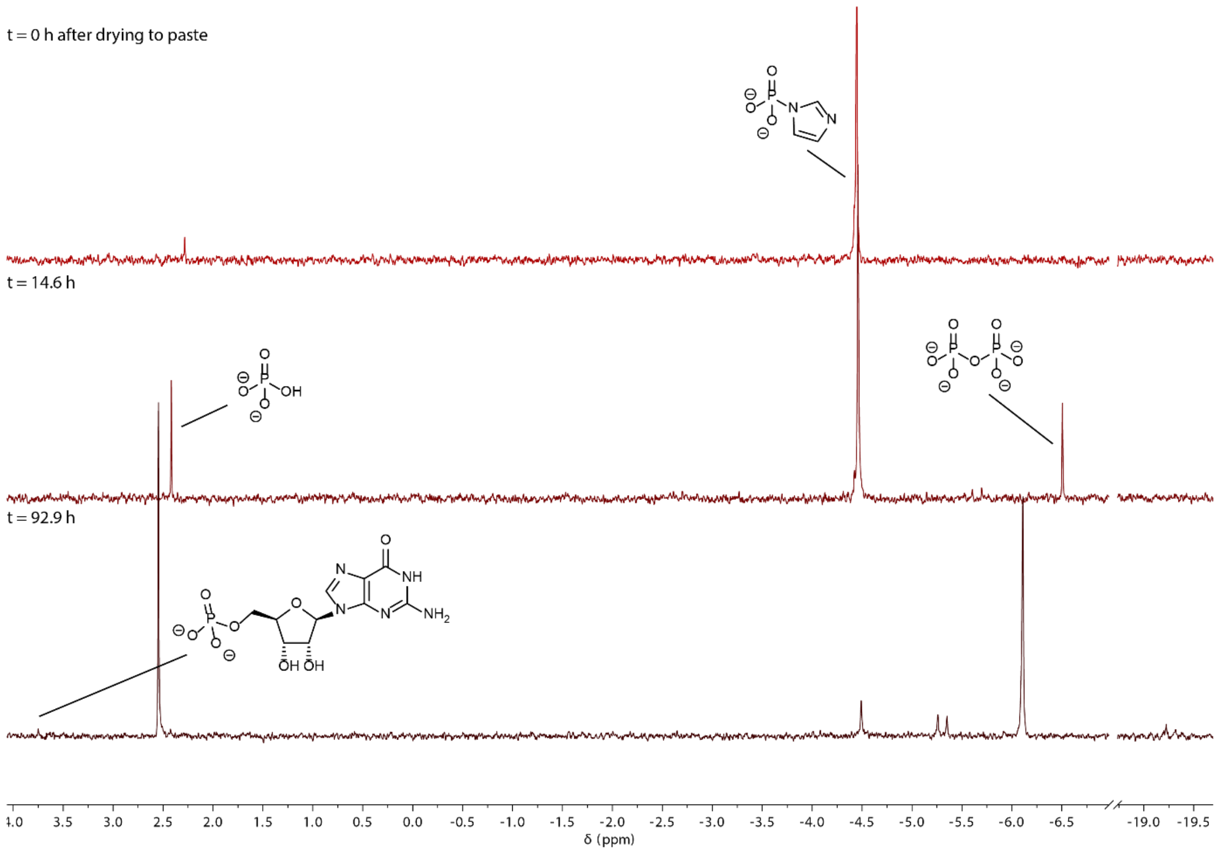

Supplementary Figure 97: Representative ^{31}P NMR spectra over time for the reaction of 0.13 mmol calcium imidazole phosphate + 0.65 mmol guanosine in paste conditions at 50 °C. Top spectrum was taken after evaporation to form a paste. All NMR spectra were measured in a 0.5 M citric acid buffer at pH 6.85 9 : 1 H_2O : D_2O. The chemical shift of the pyrophosphate peak is variable at pH 6.85 due to the pyrophosphate pK_a = 6.6.

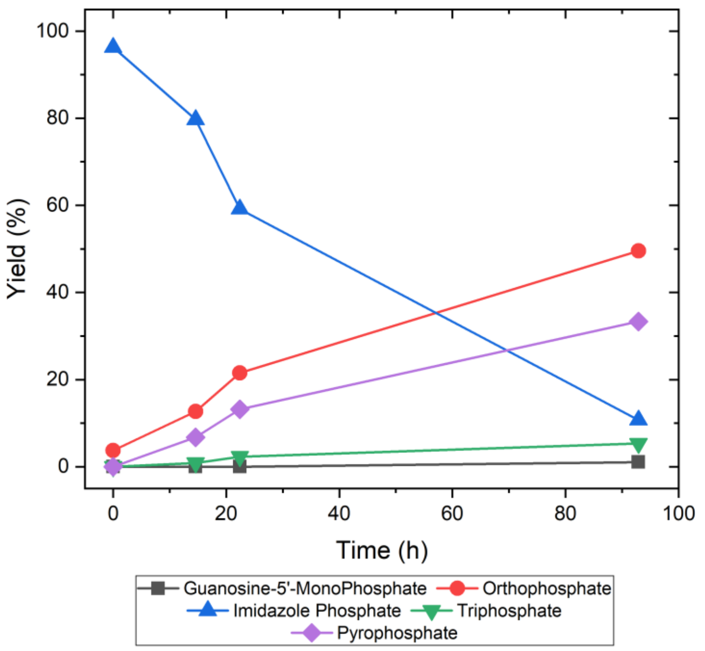

Supplementary Figure 98: Changes in yield over time for the reaction of 0.13 mmol calcium imidazole phosphate + 0.65 mmol guanosine in paste conditions at 50 °C.

A spiking experiment with a genuine sample of guanosine-5'-monophosphate confirmed the formation of guanosine-5'-monophosphate from the reaction of calcium imidazole phosphate and guanosine (Supplementary Figure 99).

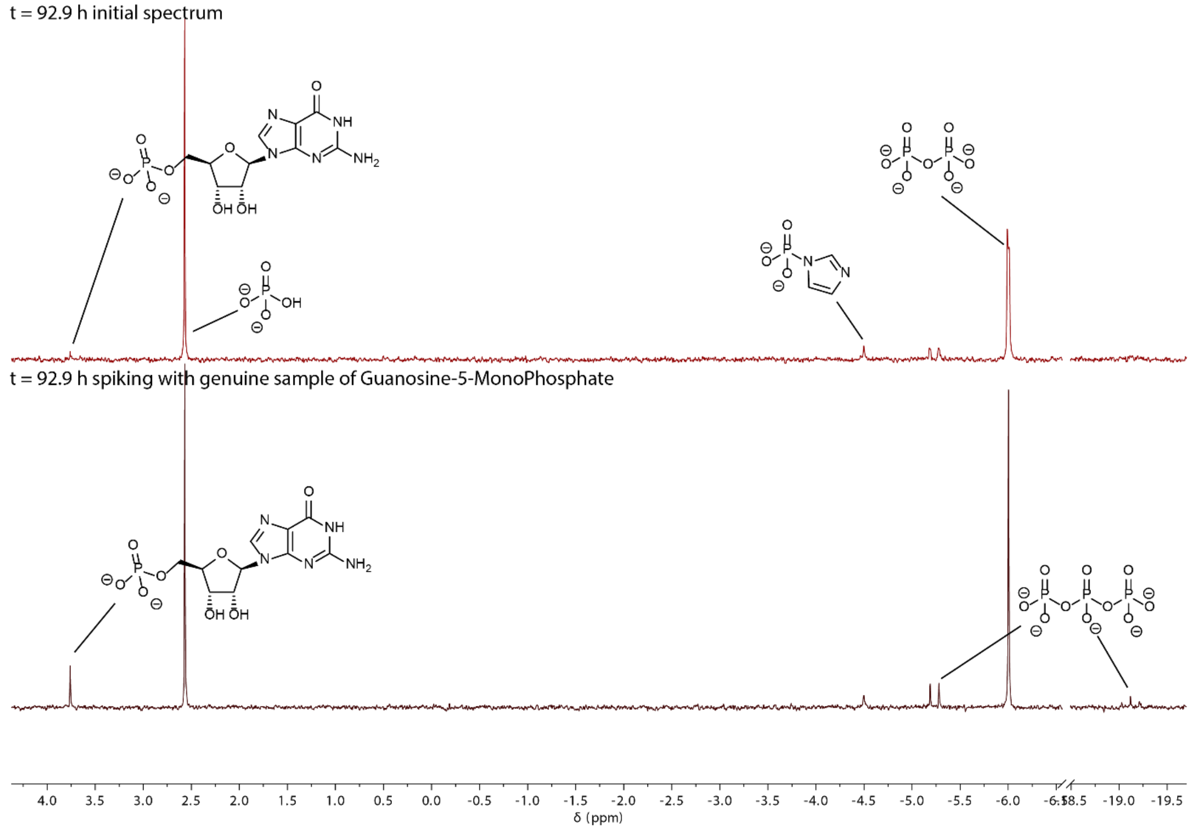

Supplementary Figure 99: ^{31}P NMR spectra of a spiking experiment for the reaction of 0.13 mmol calcium imidazole phosphate + 0.65 mmol guanosine in paste conditions at 50 °C. Top spectrum is unspiked. Bottom spectrum is with the addition of genuine guanosine-5'-monophosphate.

S6.5.4 Phosphorylation of uridine

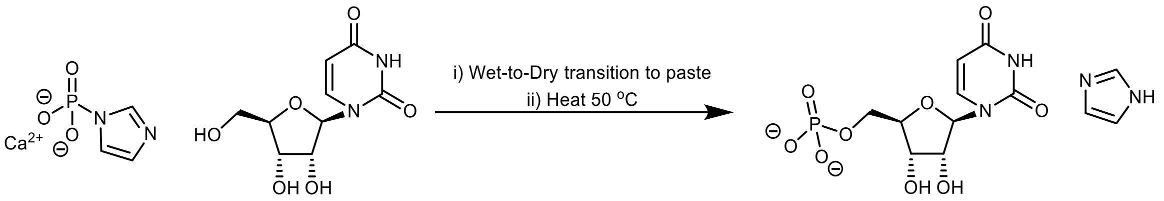

This reaction was performed according to the procedure in Section S6.1 but with the ribose replaced by 183.0 mg (0.65 mmol) uridine.

Formation of uridine-5′-monophosphate occurred in the paste from the reaction between imidazole phosphate and uridine with the highest observed yield of 11.2 % yield uridine-5'-monophosphate and 6.9 % yield uridine-3′-monophosphate after heating the paste at 50 °C for 22.4 h (Supplementary Figure 100 and 101).

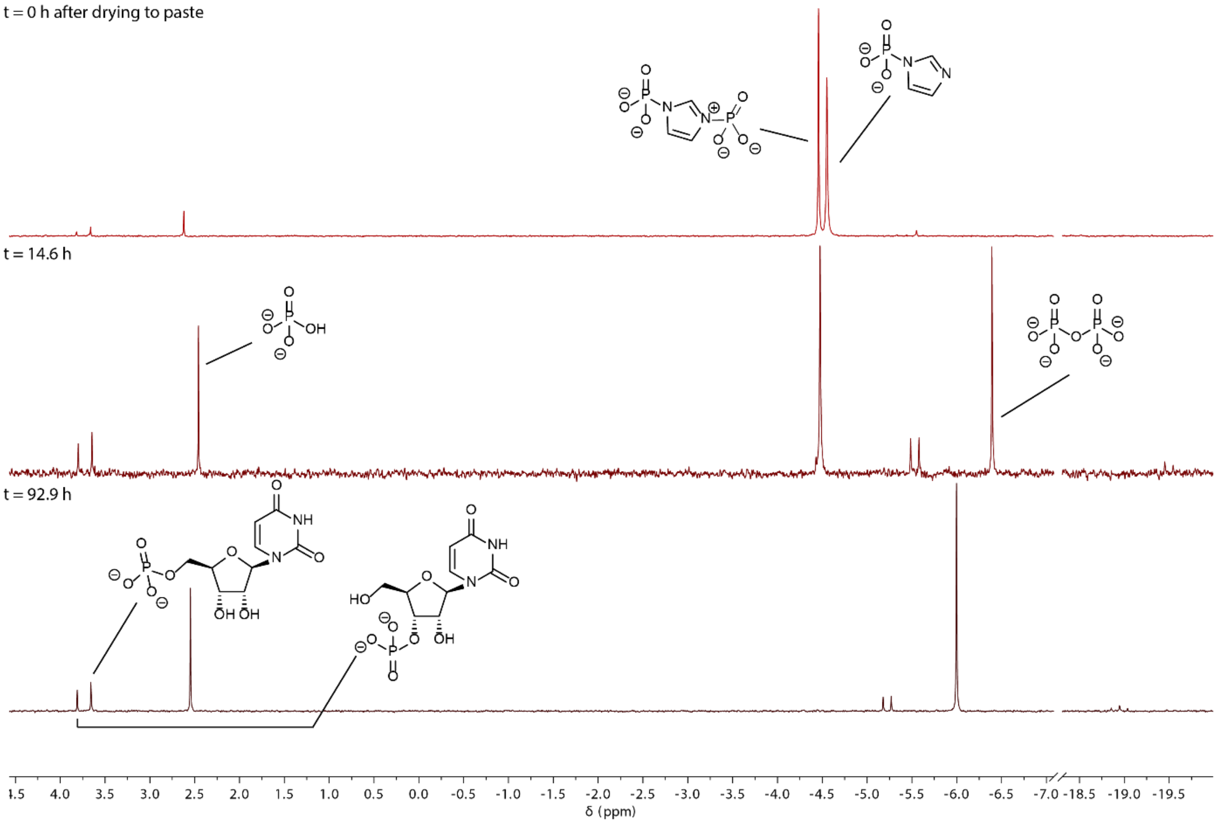

Supplementary Figure 100: Representative ^{31}P NMR spectra over time for the reaction of 0.13 mmol calcium imidazole phosphate + 0.65 mmol uridine in paste conditions at 50 °C. Top spectrum was taken after evaporation to form a paste. All NMR spectra were measured in a 0.5 M citric acid buffer at pH 6.85 9 : 1 H_2O : D_2O. The chemical shift of the pyrophosphate peak is variable at pH 6.85 due to the pyrophosphate pK_a = 6.6.

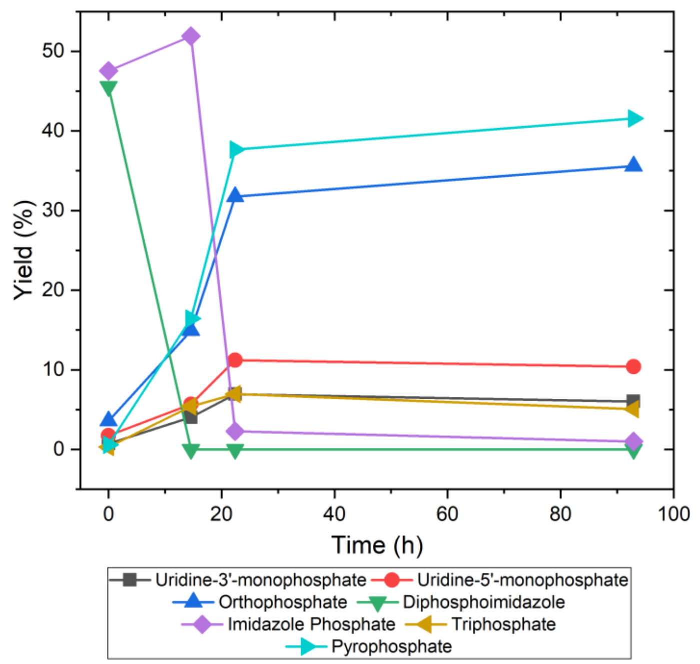

Supplementary Figure 101: Changes in yield over time for the reaction of 0.13 mmol calcium imidazole phosphate + 0.65 mmol uridine in paste conditions at 50 °C.

A spiking experiment with a genuine sample of uridine-5’-monophosphate confirmed the formation of uridine-5’-monophosphate from the reaction of calcium imidazole phosphate and uridine (Supplementary Figure 102).

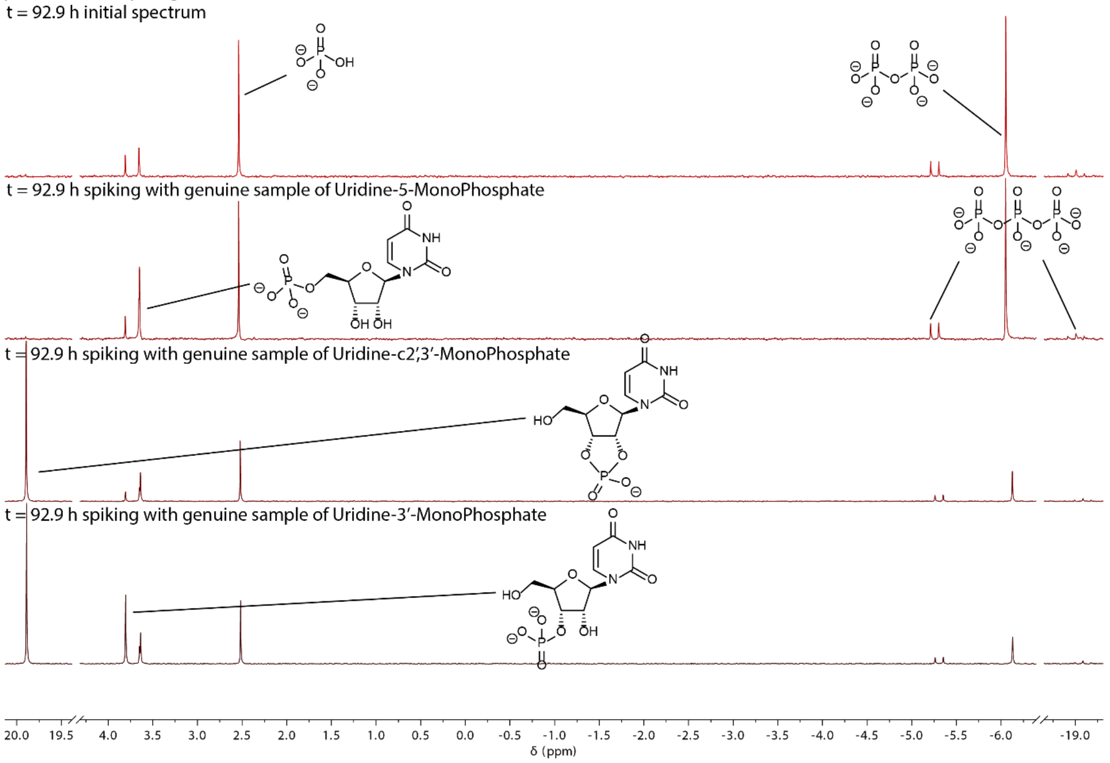

Supplementary Figure 102: ^{31}P NMR spectra of a spiking experiment for the reaction of 0.13 mmol calcium imidazole phosphate + 0.65 mmol uridine in paste conditions at 50 °C. Top spectrum is unspiked. Second from top spectrum is with the addition of genuine uridine-5’-monophosphate. Third from top spectrum is with the addition of genuine uridine-c2’,3’-monophosphate. Bottom spectrum is with the addition of genuine uridine-3’-monophosphate.

S6.6 Phosphorylation of Nucleotides

S6.6.1 Phosphorylation of adenosine-5'-monophosphate

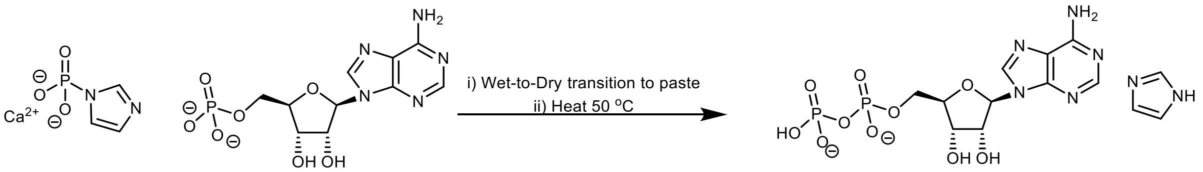

This reaction was performed according to the procedure in Section S6.1 but with the ribose replaced by 254.4 mg (0.65 mmol) adenosine-5'-monophosphate disodium salt.

Formation of adenosine-5'-diphosphate occurred in the paste from the reaction between imidazole phosphate and adenosine-5'-monophosphate with the highest observed yield of 20.2 % after heating the paste at 50 °C for 4.3 h (Supplementary Figure 103 and 104). Excessive heating of the paste did cause hydrolysis of the ADP formed (Supplementary Figure 104).

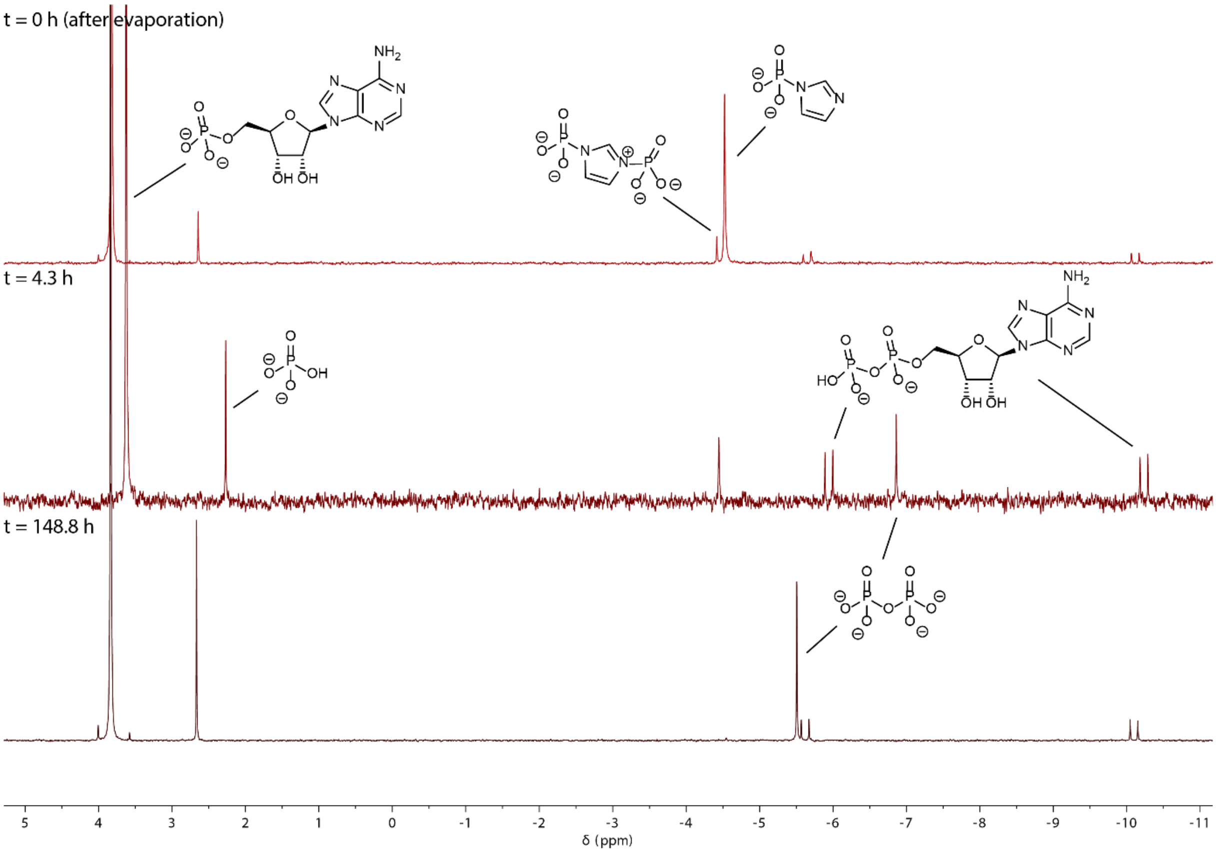

Supplementary Figure 103: Representative ^{31}P NMR spectra over time for the reaction of 0.13 mmol calcium imidazole phosphate + 0.65 mmol adenosine-5'-monophosphate in paste conditions at 50 °C. Top spectrum was taken after evaporation to form a paste. All NMR spectra were measured in a 0.5 M citric acid buffer at pH 6.85 9 : 1 H_2O : D_2O. The chemical shift of the pyrophosphate peak is variable at pH 6.85 due to the pyrophosphate pK_a = 6.6.

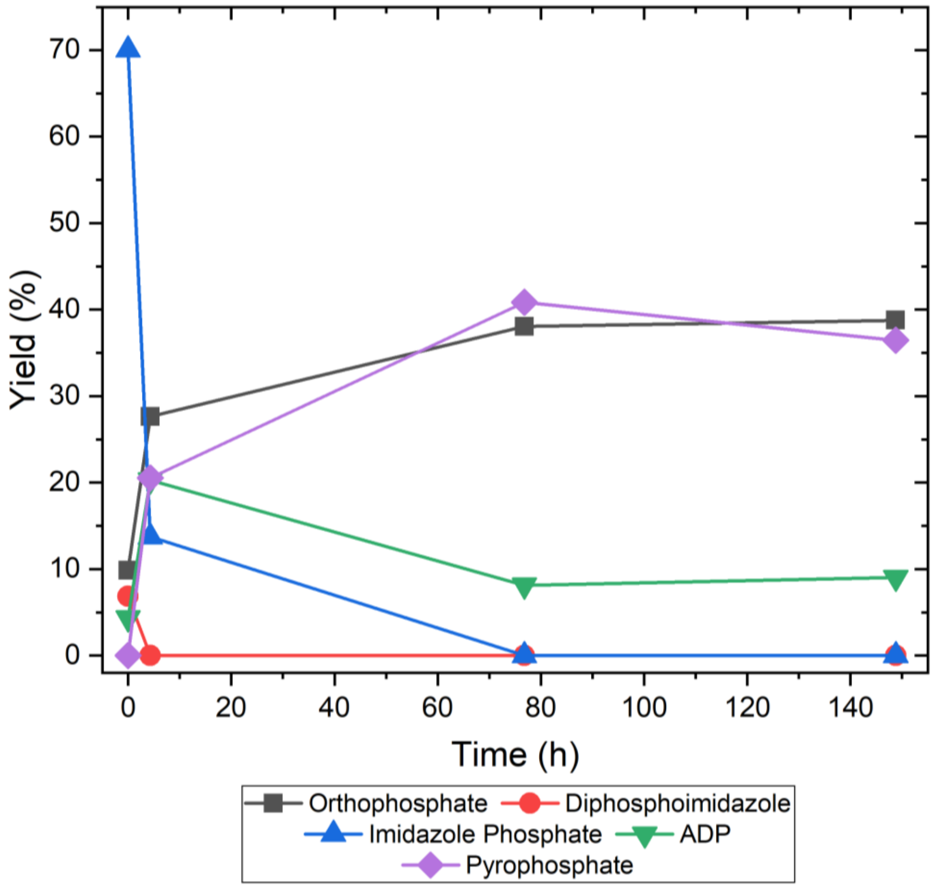

Supplementary Figure 104: Changes in yield over time for the reaction of 0.13 mmol calcium imidazole phosphate + 0.65 mmol adenosine-5’-monophosphate in paste conditions at 50 °C.

A spiking experiment with a genuine sample of adenosine-5’-diphosphate confirmed the formation of adenosine-5’-diphosphate from the reaction of calcium imidazole phosphate and adenosine-5’-monophosphate (Supplementary Figure 105).

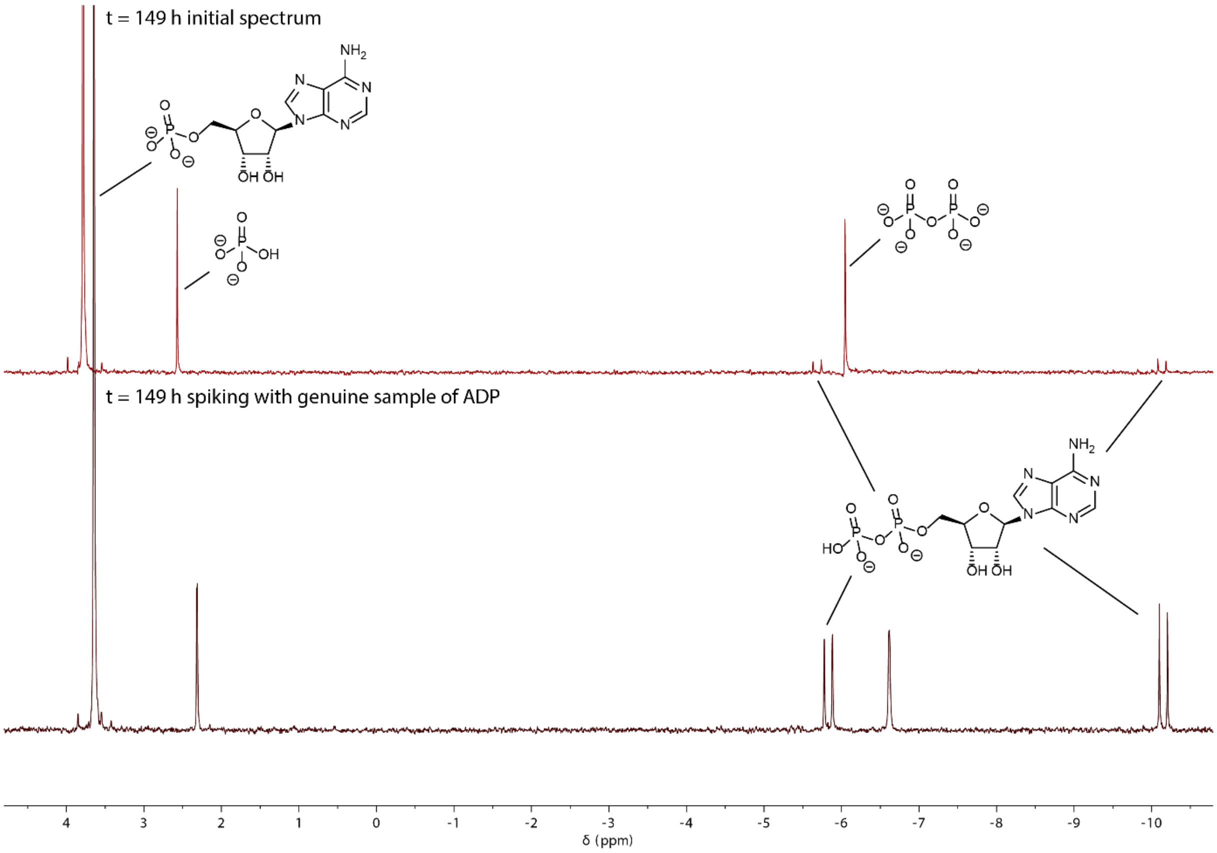

Supplementary Figure 105: ^{31}P NMR spectra of a spiking experiment for the reaction of 0.13 mmol calcium imidazole phosphate + 0.65 mmol adenosine-5'-monophosphate in paste conditions at 50 °C. Top spectrum is unspiked. Bottom spectrum is with the addition of genuine adenosine-5'-monophosphate. All NMR spectra were measured in a 0.5 M citric acid buffer at pH 6.85 9 : 1 H_2O : D_2O. The chemical shift of the pyrophosphate peak is variable at pH 6.85 due to the pyrophosphate pK_a = 6.6.

S6.6.1.1 Control reaction for the phosphorylation of adenosine-5'-monophosphate

A control reaction was performed in the absence of calcium imidazole phosphate in order to determine if any adenosine-5'-diphosphate formation could have formed from a phosphate transfer reaction from one adenosine-5'-monophosphate molecule to another. Supplementary Figure 106 showed that no ADP was formed from the reaction of adenosine-5'-monophosphate with itself.

31.8 mg of adenosine-5'-monophosphate (0.0813 mmol) was dissolved in 0.5 mL of MilliQ and the solution was left to dry in a petri dish overnight. The paste in the morning had dried out and 0.1 µL / mg of D_2O was added to rewet the sample and form a paste. An NMR sample of the paste was taken by dissolving a portion of the paste in a 0.5 M citric acid buffer at pH 6.85 in 9 : 1 H_2O : D_2O.

The paste was placed in an Eppendorf and spun on a centrifuge for 3 min at 10 k rpm in order to move the paste at the bottom of the Eppendorf. The sample was then placed on a thermoshaker at 50 °C and 600 rpm for 24.7 h. An NMR sample of the paste was taken after 24.7 h by again dissolving a portion of the paste in a 0.5 M citric acid buffer at pH 6.85 in 9 : 1 H_2O : D_2O.

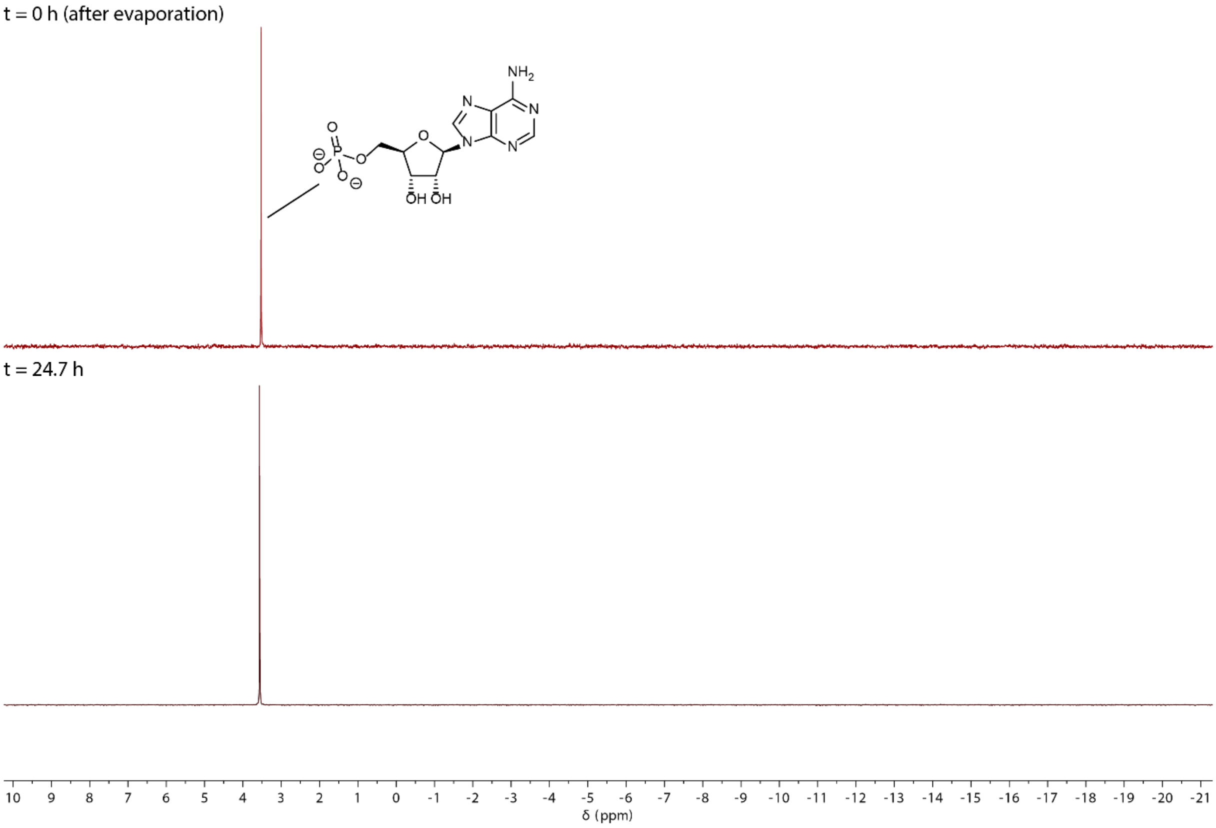

Supplementary Figure 106: ^{31}P NMR spectra of a control reaction performed by heating 0.081 mmol adenosine-5'-monophosphate in paste conditions at 50 °C in the absence of calcium imidazole phosphate.

S6.6.2 Phosphorylation of adenosine-5'-diphosphate

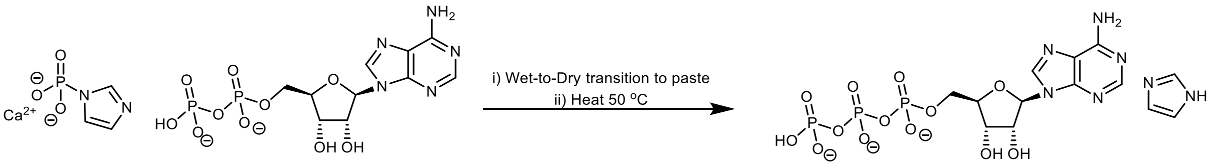

This reaction was performed according to the procedure in Section S6.1 but with the ribose replaced by 306.3 mg (0.65 mmol) adenosine-5'-diphosphate disodium salt hydrate.

Formation of adenosine-5'-triphosphate occurred in the paste from the reaction between imidazole phosphate and adenosine-5'-diphosphate with the highest observed yield of 18.2 % after drying to a

paste at 0 h (Supplementary Figure 107 and S108). Excessive heating of the paste did cause hydrolysis of both the ADP to AMP and hydrolysis of formed ATP (Supplementary Figure 108).

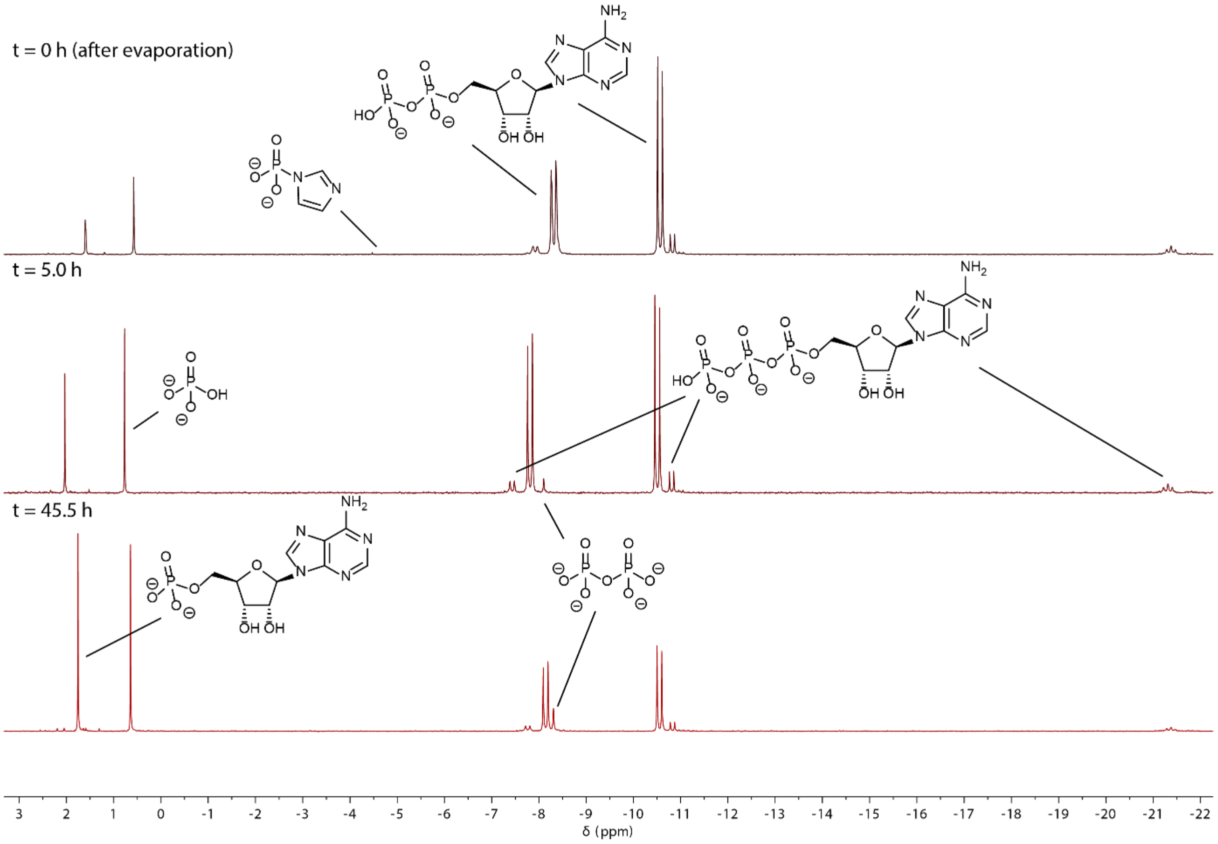

Supplementary Figure 107: Representative ^{31}P NMR spectra over time for the reaction of 0.13 mmol calcium imidazole phosphate + 0.65 mmol adenosine-5’-diphosphate in paste conditions at 50 °C. Top spectrum was taken after evaporation to form a paste.

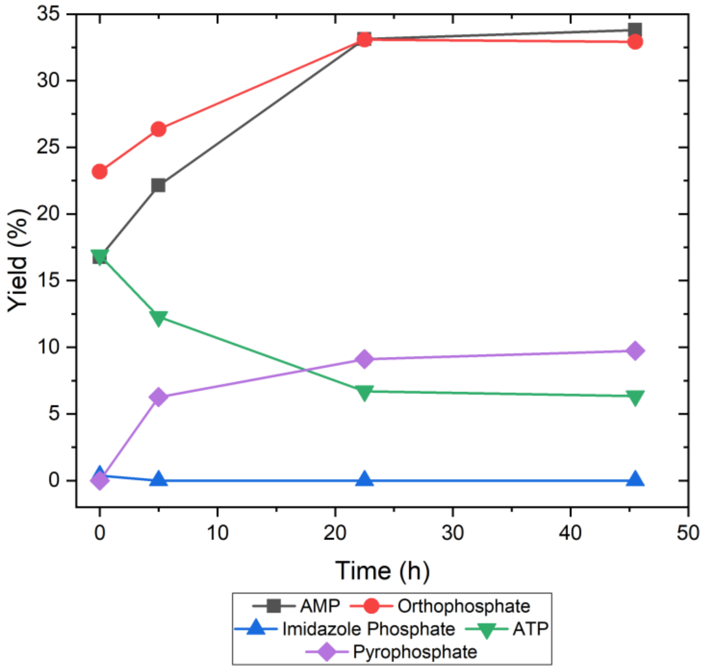

Supplementary Figure 108: Changes in yield over time for the reaction of 0.13 mmol calcium imidazole phosphate + 0.65 mmol adenosine-5’-diphosphate in paste conditions at 50 °C.

A spiking experiment with a genuine sample of adenosine-5′-triphosphate confirmed the formation of adenosine-5′-triphosphate from the reaction of calcium imidazole phosphate and adenosine-5′-diphosphate (Supplementary Figure 109).

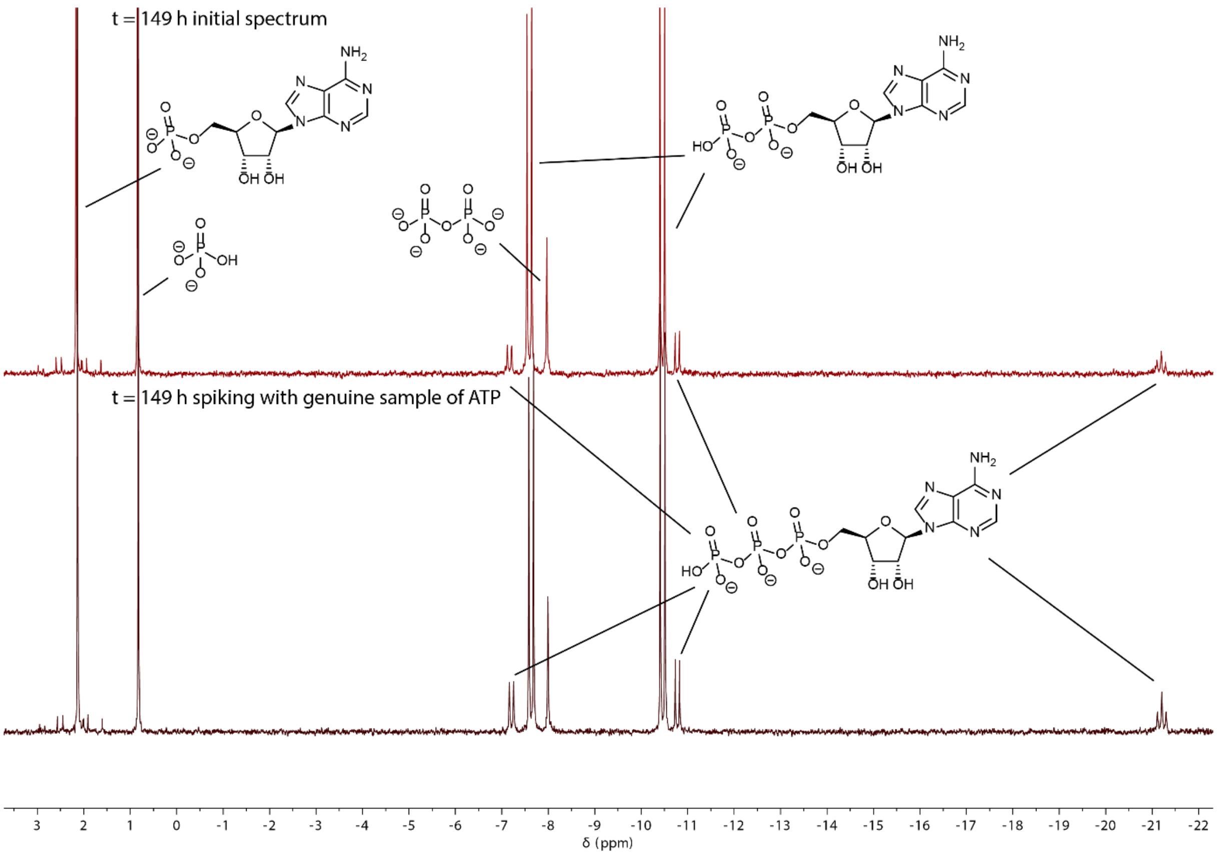

Supplementary Figure 109: ^{31}P NMR spectra of a spiking experiment for the reaction of 0.13 mmol calcium imidazole phosphate + 0.65 mmol adenosine-5′-diphosphate in paste conditions at 50 °C. Top spectrum is unspiked. Bottom spectrum is with the addition of genuine adenosine-5′-triphosphate.

S6.6.2.1 Control reaction for the phosphorylation of adenosine-5′-diphosphate

A control reaction was performed in the absence of calcium imidazole phosphate in order to determine if any adenosine-5′-triphosphate could have formed from a phosphate transfer reaction from one adenosine-5′-diphosphate molecule to another. Supplementary Figure 110 showed that ATP was formed from the reaction of adenosine-5′-diphosphate with itself in a 3.5 % yield after heating at 50 °C for 24.7 h. However, this yield is considerably lower than the 18.2 % yield recorded for the reaction in the presence of imidazole phosphate indicating that the ATP was predominantly formed from a reaction between imidazole phosphate and ADP.

152.9 mg of adenosine-5’-diphosphate (0.325 mmol) was dissolved in 2.0 mL of MilliQ and the solution was left to dry in a petri dish overnight. The paste had dried out in the morning and 0.1 µL / mg of D_2O was added to rewet the sample and form a paste. An NMR sample of the paste was taken by dissolving a portion of the paste in a 0.5 M citric acid buffer at pH 6.85 in 9 : 1 H_2O : D_2O.

The paste was placed in an Eppendorf and spun on a centrifuge for 3 min at 5590 g in order to move the paste at the bottom of the Eppendorf. The sample was then placed on a thermoshaker at 50 °C and 600 rpm for 45.5 h. NMR samples of the paste were taken at 5.0 h, 22.5 h and 45.5 h by again dissolving a portion of the paste in a 0.5 M citric acid buffer at pH 6.85 in 9 : 1 H_2O : D_2O.

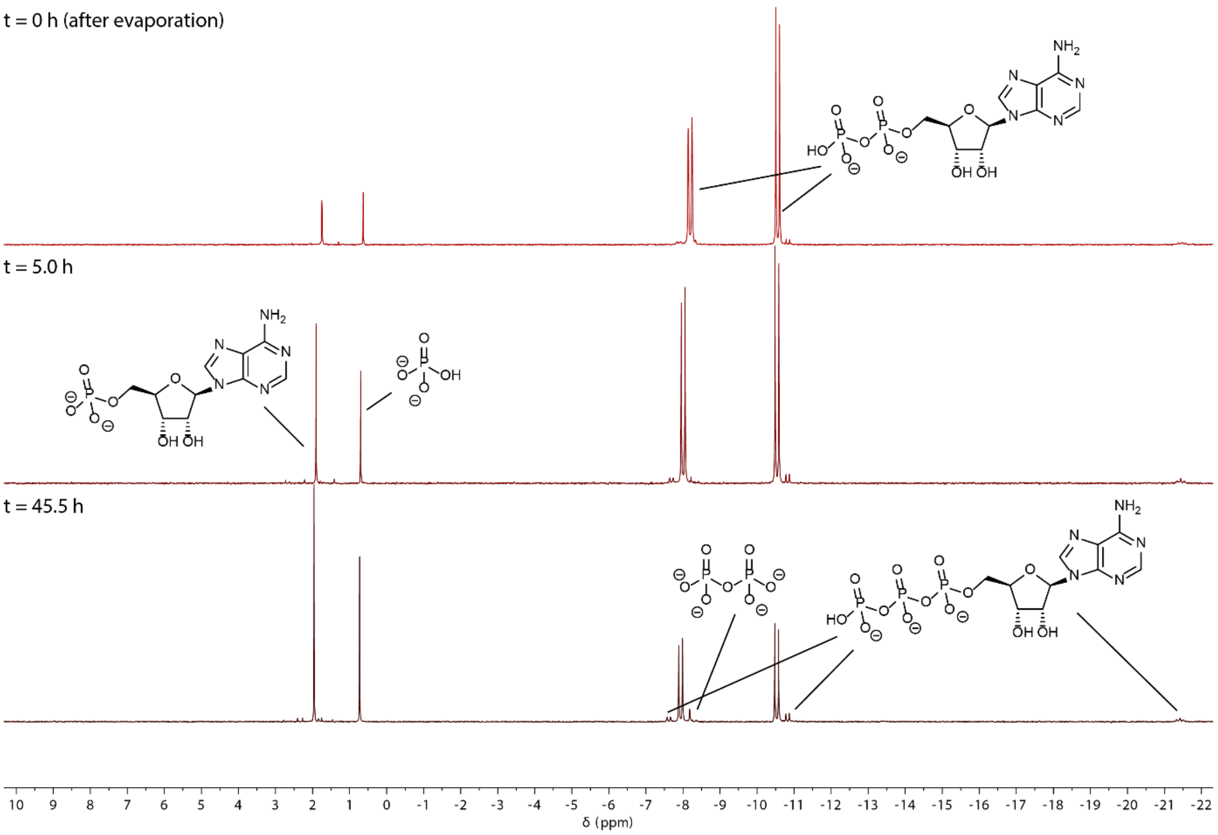

Supplementary Figure 110: ^{31}P NMR spectra of a control reaction performed by heating 0.325 mmol adenosine-5’-diphosphate in paste conditions at 50 °C in the absence of calcium imidazole phosphate.

S6.6.3 Phosphorylation of cytidine-5'-monophosphate

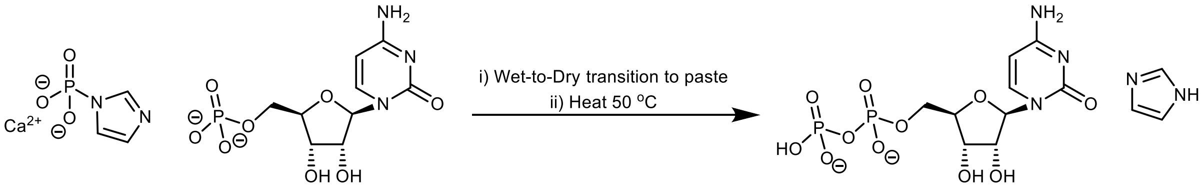

This reaction was performed according to the procedure in Section S6.1 but with the ribose replaced by 238.0 mg (0.65 mmol) cytidine-5'-monophosphate disodium salt hydrate.

Formation of cytidine-5'-diphosphate occurred in the paste from the reaction between imidazole phosphate and cytidine-5'-monophosphate with the highest observed yield of 4.3 % after heating the paste at 50 °C for 5.0 h (Supplementary Figure 111 and 112).

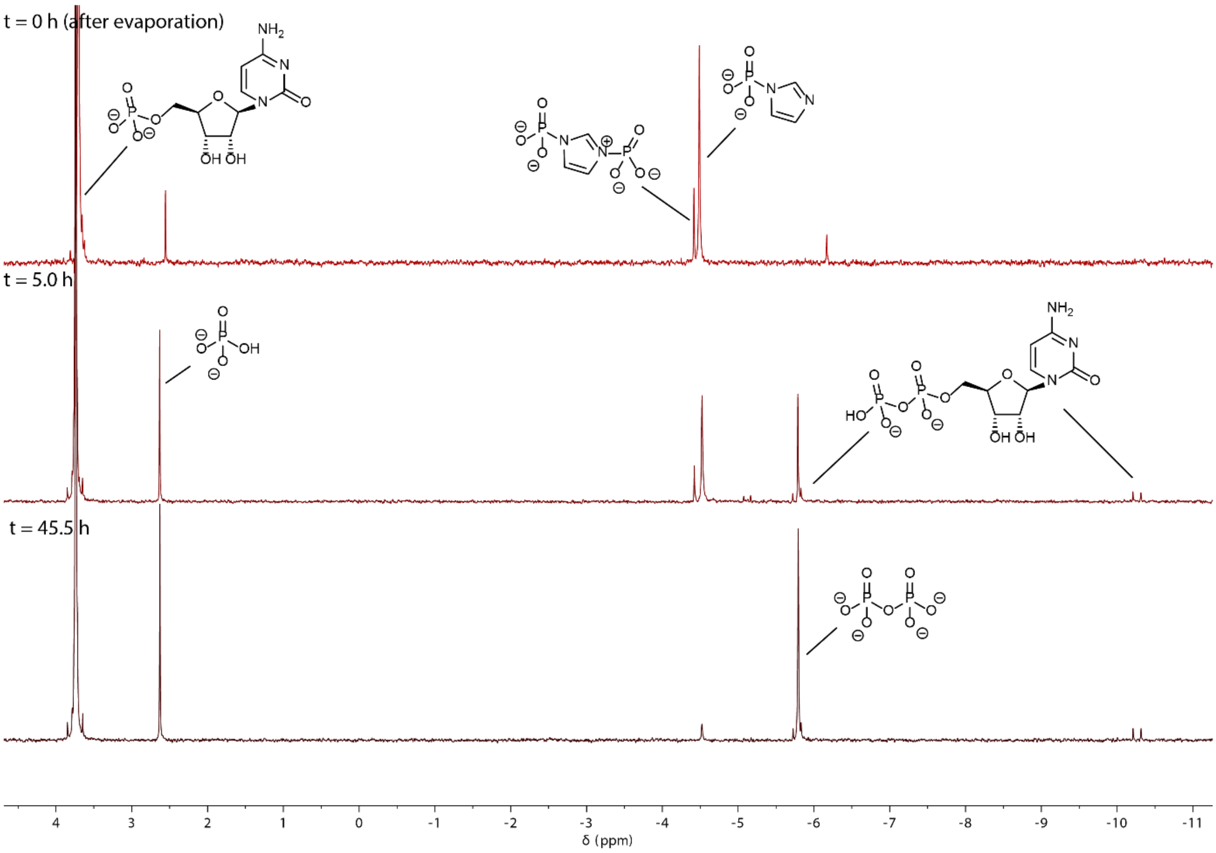

Supplementary Figure 111: Representative ^{31}P NMR spectra over time for the reaction of 0.13 mmol calcium imidazole phosphate + 0.65 mmol cytidine-5'-monophosphate in paste conditions at 50 °C. Top spectrum was taken after evaporation to form a paste. All NMR spectra were measured in a 0.5 M citric acid buffer at pH 6.85 9 : 1 H_2O : D_2O. The chemical shift of the pyrophosphate peak is variable at pH 6.85 due to the pyrophosphate pK_a = 6.6.

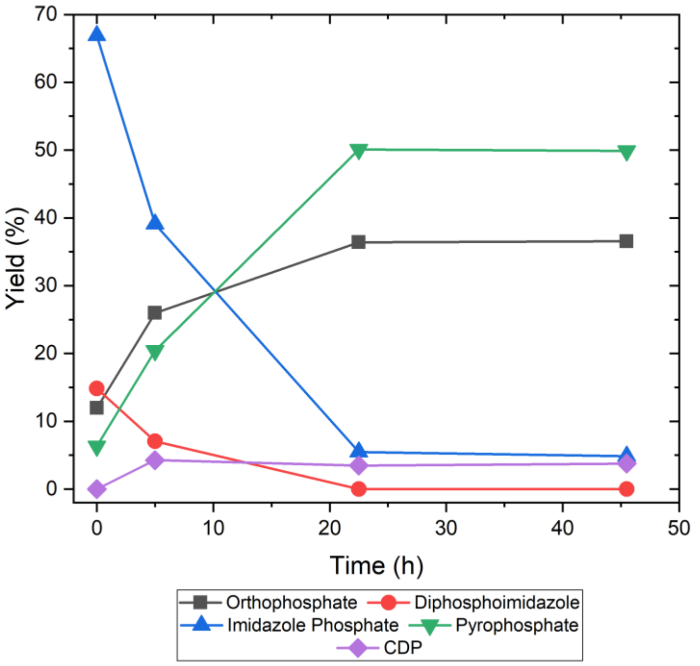

Supplementary Figure 112: Changes in yield over time for the reaction of 0.13 mmol calcium imidazole phosphate + 0.65 mmol cytidine-5’-monophosphate in paste conditions at 50 °C.

A spiking experiment with a genuine sample of cytidine-5’-diphosphate confirmed the formation of cytidine-5’-diphosphate from the reaction of calcium imidazole phosphate and cytidine-5’-monophosphate (Supplementary Figure 113).

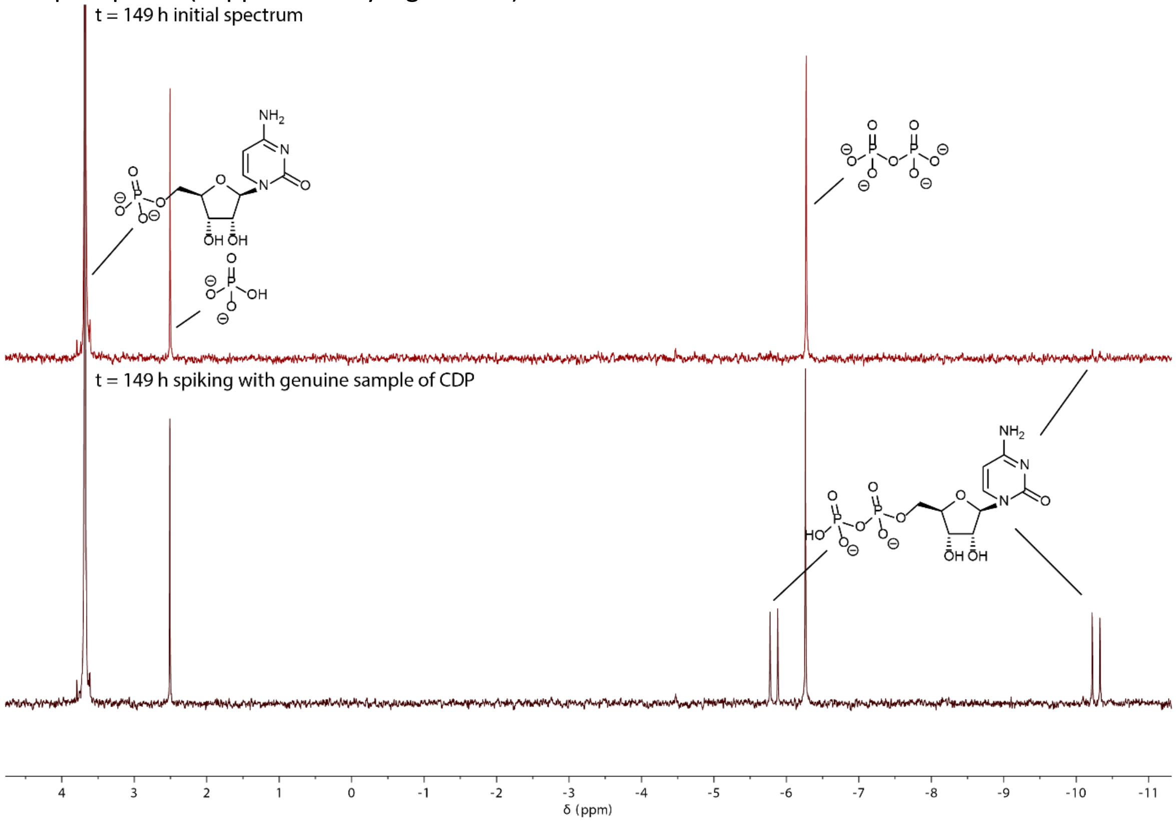

Supplementary Figure 113: ^{31}P NMR spectra of a spiking experiment for the reaction of 0.13 mmol calcium imidazole phosphate + 0.65 mmol cytidine-5’-monophosphate in paste conditions at 50 °C. Top spectrum is unspiked. Bottom spectrum is with the addition of genuine cytidine-5’-diphosphate.

S6.6.3.1 Control reaction for the phosphorylation of cytidine-5'-monophosphate

A control reaction was performed in the absence of calcium imidazole phosphate in order to determine if any cytidine-5'-diphosphate formation could have formed from a phosphate transfer reaction from one cytidine-5'-monophosphate molecule to another. Supplementary Figure 114 showed that no cytidine-5'-diphosphate was formed from the reaction of cytidine-5'-monophosphate with itself.

118.9 mg of cytidine-5'-monophosphate (0.325 mmol) was dissolved in 2.0 mL of MilliQ and the solution was left to dry in a petri dish overnight. In the morning a highly viscous yellow paste had formed. An NMR sample of the paste was taken by dissolving a portion of the paste in a 0.5 M citric acid buffer at pH 6.85 in 9 : 1 H_2O : D_2O.
The paste was placed in an Eppendorf and spun on a centrifuge for 3 min at 10 k rpm in order to move the paste at the bottom of the Eppendorf. The sample was then placed on a thermoshaker at 50 °C and 600 rpm for 24.7 h. NMR samples of the paste were taken at 5.0 h, 22.5 h and 45.5 h by again dissolving a portion of the paste in a 0.5 M citric acid buffer at pH 6.85 in 9 : 1 H_2O : D_2O.

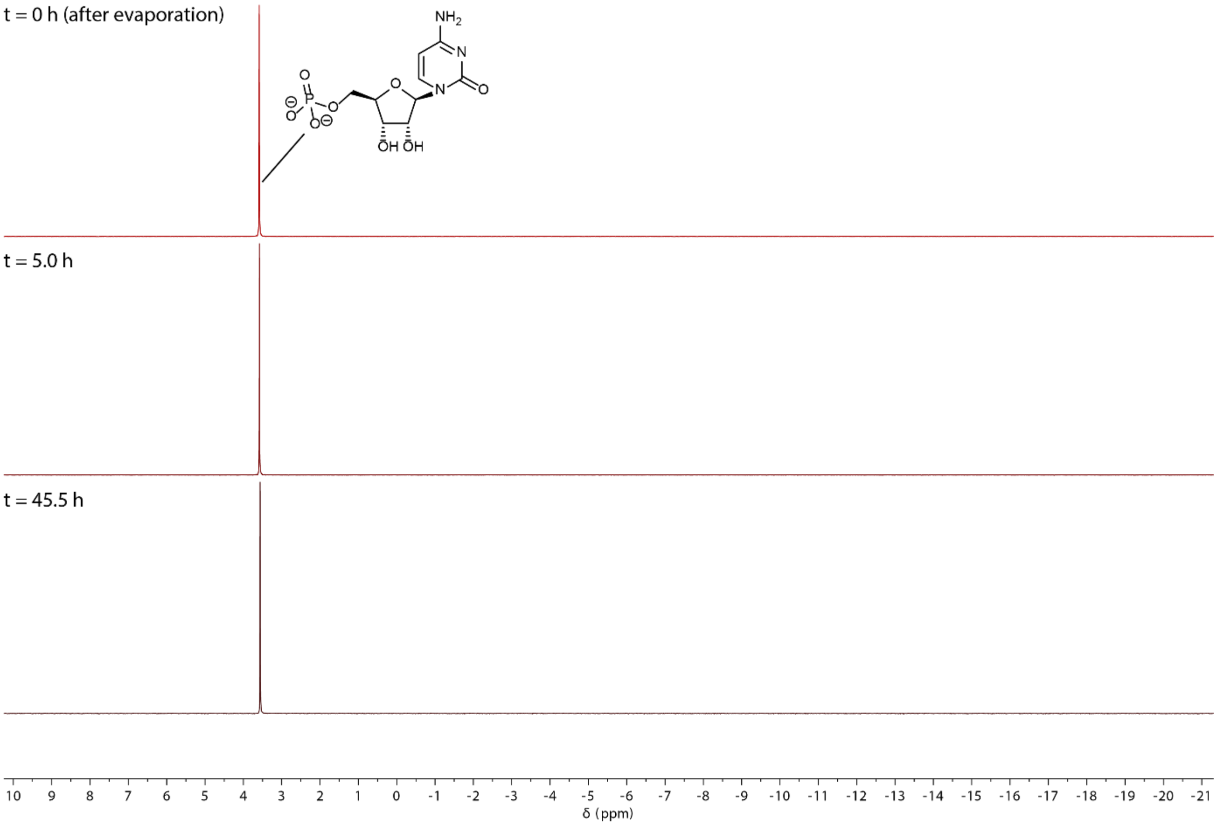

Supplementary Figure 114: ^{31}P NMR spectra of a control reaction performed by heating 0.325 mmol cytidine-5'-monophosphate in paste conditions at 50 °C in the absence of calcium imidazole phosphate.

S6.6.4 Phosphorylation of cytidine-5'-diphosphate

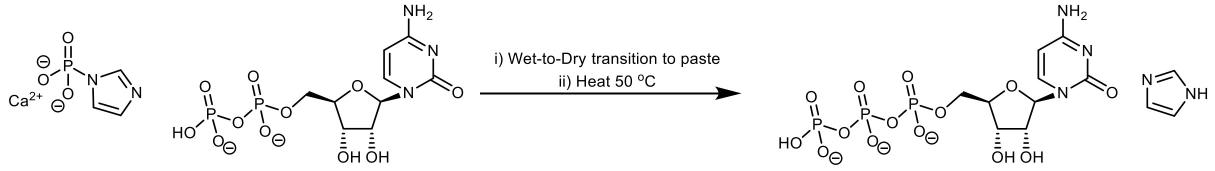

This reaction was performed according to the procedure in Section S6.1 but with the ribose replaced by 305 mg (0.65 mmol) cytidine-5'-diphosphate disodium salt dihydrate.

Formation of cytidine-5'-triphosphate occurred in the paste from the reaction between imidazole phosphate and cytidine-5'-diphosphate with the highest observed yield of 11.6 % after heating the paste at 50 °C for 4.3 h (Supplementary Figure 115 and S116). Excessive heating of the paste did cause hydrolysis of both the CDP to CMP and hydrolysis of formed CTP (Supplementary Figure 116).

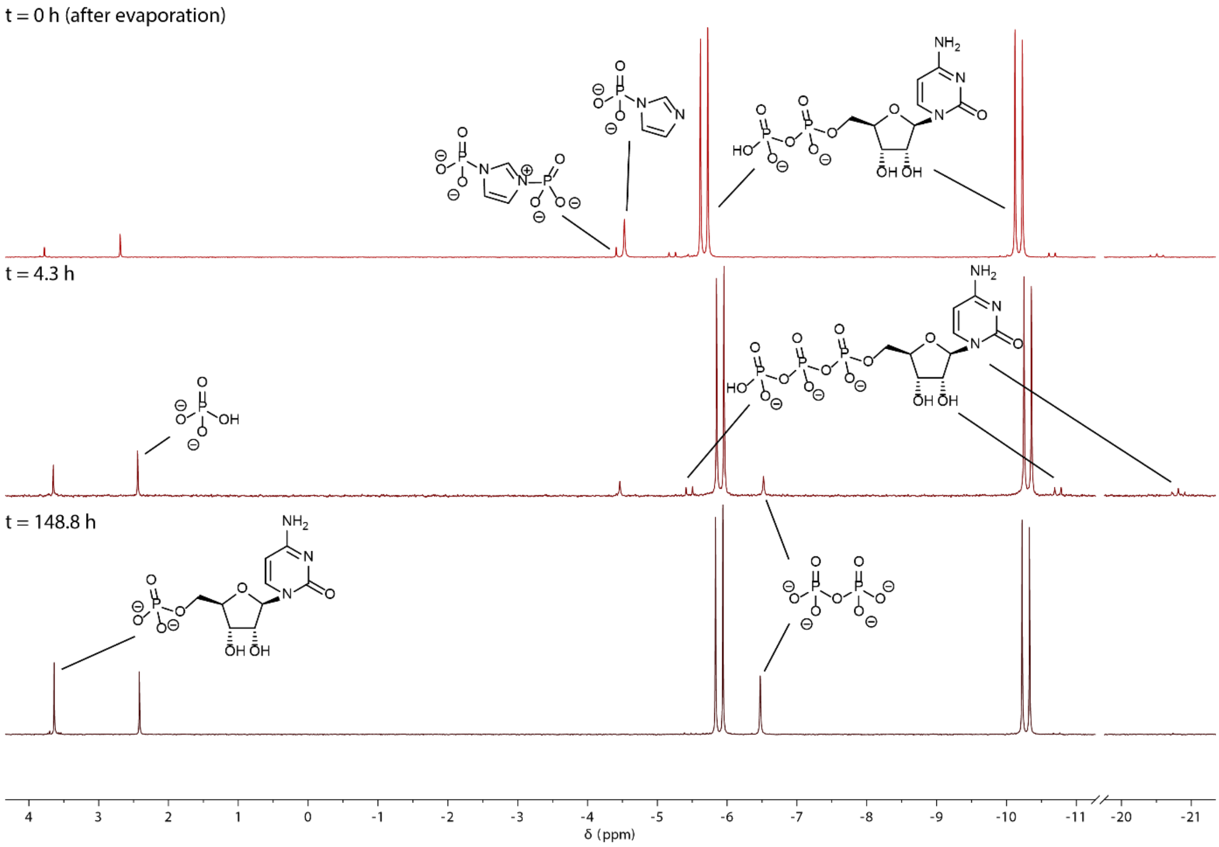

Supplementary Figure 115: Representative ^{31}P NMR spectra over time for the reaction of 0.13 mmol calcium imidazole phosphate + 0.65 mmol cytidine-5'-diphosphate in paste conditions at 50 °C. Top spectrum was taken after evaporation to form a paste.

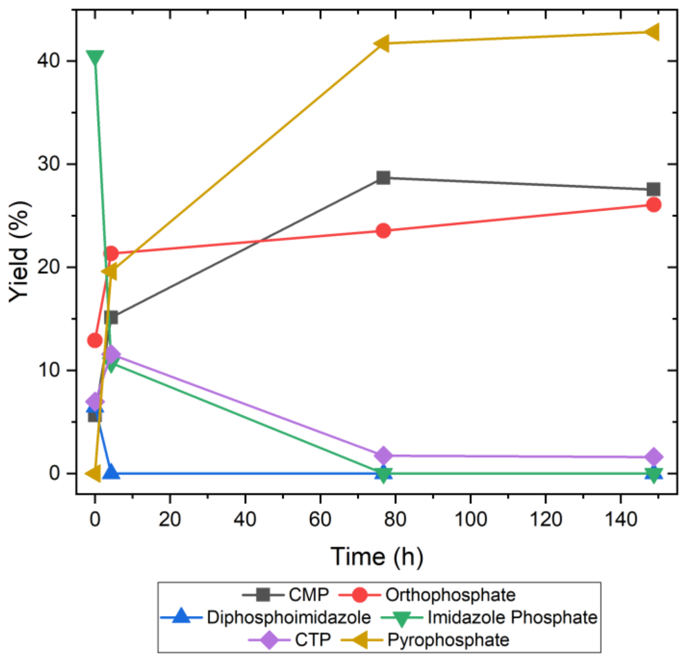

Supplementary Figure 116: Changes in yield over time for the reaction of 0.13 mmol calcium imidazole phosphate + 0.65 mmol cytidine-5’-diphosphate in paste conditions at 50 °C.

A spiking experiment with a genuine sample of cytidine-5’-triphosphate confirmed the formation of cytidine-5’-triphosphate from the reaction of calcium imidazole phosphate and cytidine-5’-diphosphate (Supplementary Figure 117).

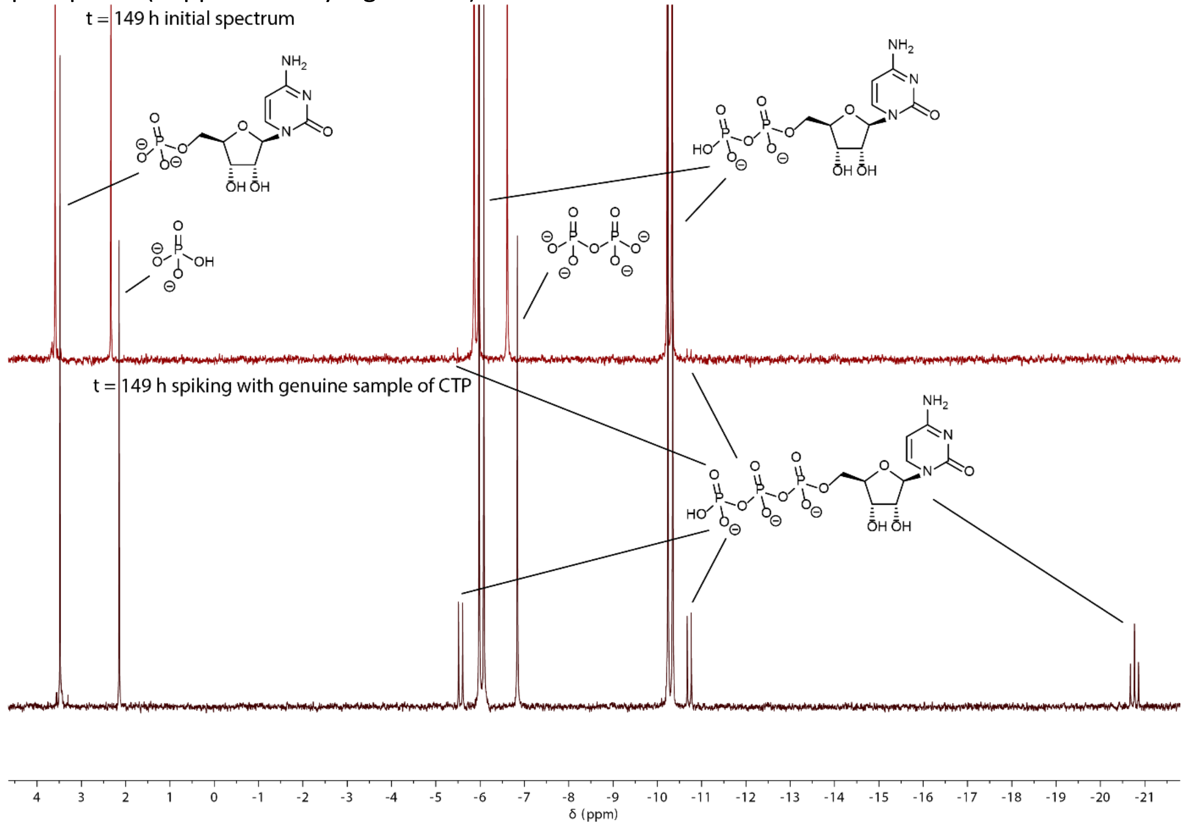

Supplementary Figure 117: ^{31}P NMR spectra of a spiking experiment for the reaction of 0.13 mmol calcium imidazole phosphate + 0.65 mmol cytidine-5’-diphosphate in paste conditions at 50 °C. Top spectrum is unspiked. Bottom spectrum is with the addition of genuine cytidine-5’-triphosphate.

S6.6.4.1 Control reaction for the phosphorylation of cytidine-5'-diphosphate

A control reaction was performed in the absence of calcium imidazole phosphate in order to determine if any cytidine-5'-triphosphate could have formed from a phosphate transfer reaction from one cytidine-5'-diphosphate molecule to another. Supplementary Figure 118 showed that no CTP was formed from the reaction of cytidine-5'-diphosphate with itself. The only reaction that did occur was hydrolysis of the CDP into CMP and orthophosphate.

The reaction was performed using 38.1 mg of cytidine-5'-diphosphate (0.0813 mmol) and an identical procedure was used as detailed in Section S6.6.1.1.

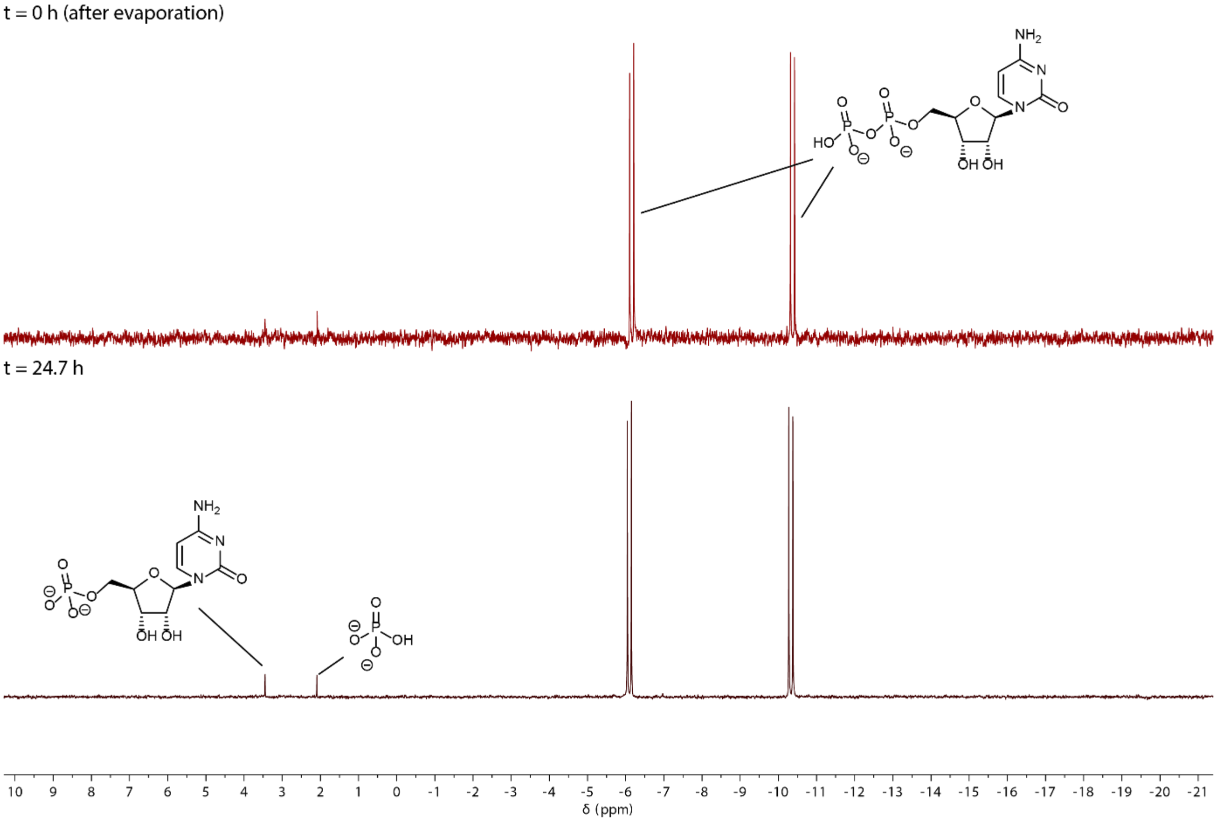

Supplementary Figure 118: ^{31}P NMR spectra of a control reaction performed by heating 0.0813 mmol cytidine-5'-diphosphate in paste conditions at 50 °C in the absence of calcium imidazole phosphate.

S6.6.5 Phosphorylation of guanosine-5'-monophosphate

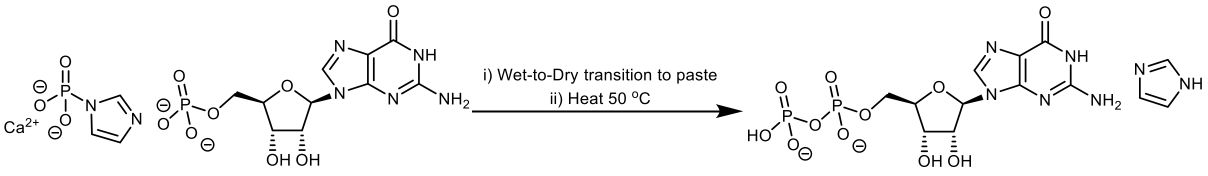

This reaction was performed according to the procedure in Section S6.1 but with the ribose replaced by 265.0 mg (0.65 mmol) guanosine-5'-monophosphate disodium salt.

Formation of guanosine-5'-diphosphate occurred in the paste from the reaction between imidazole phosphate and guanosine-5'-monophosphate with the highest observed yield of 14.6 % after heating the paste at 50 °C for 4.3 h (Supplementary Figure 119 and 120). Excessive heating of the paste did cause hydrolysis of the GDP formed (Supplementary Figure 120).

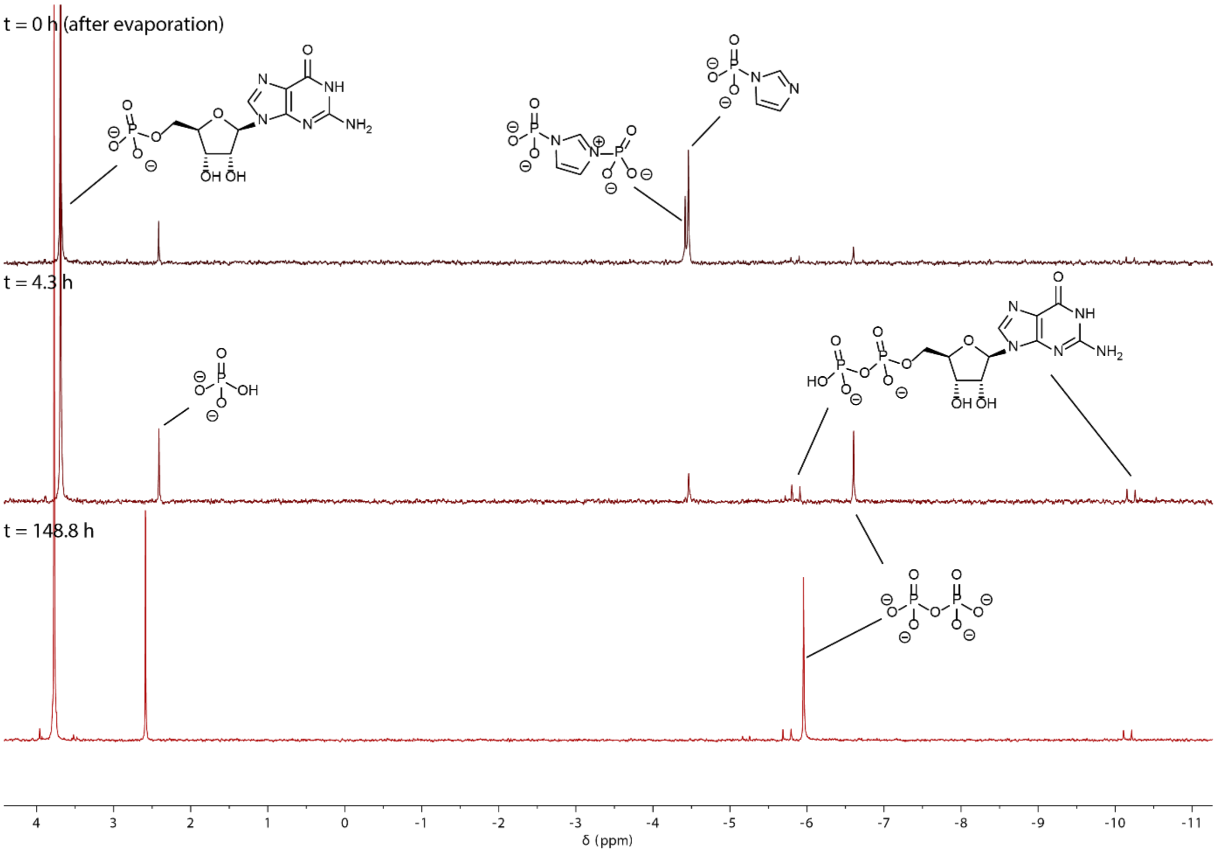

Supplementary Figure 119: Representative ^{31}P NMR spectra over time for the reaction of 0.13 mmol calcium imidazole phosphate + 0.65 mmol guanosine-5'-monophosphate in paste conditions at 50 °C. Top spectrum was taken after evaporation to form a paste. All NMR spectra were measured in a 0.5 M citric acid buffer at pH 6.85 9 : 1 H_2O : D_2O. The chemical shift of the pyrophosphate peak is variable at pH 6.85 due to the pyrophosphate pK_a = 6.6.

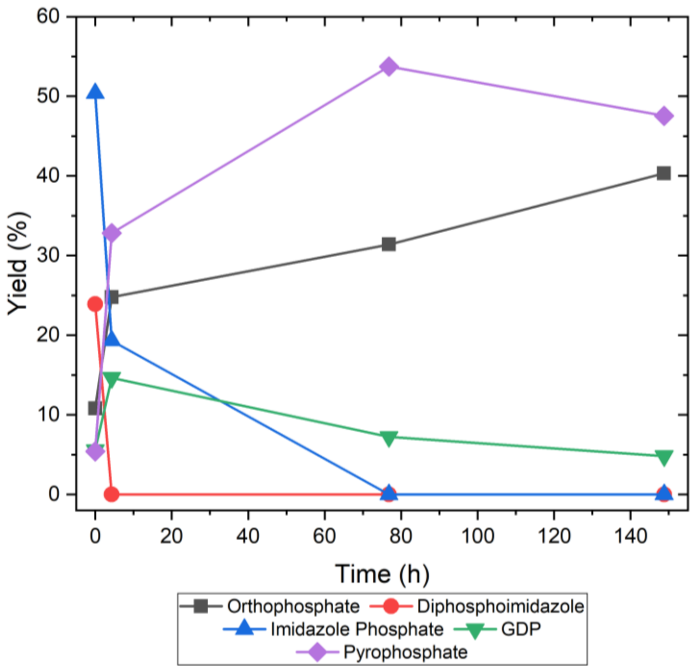

Supplementary Figure 120: Changes in yield over time for the reaction of 0.13 mmol calcium imidazole phosphate + 0.65 mmol guanosine-5'-monophosphate in paste conditions at 50 °C.

A spiking experiment with a genuine sample of guanosine-5'-diphosphate confirmed the formation of guanosine-5'-diphosphate from the reaction of calcium imidazole phosphate and guanosine-5'-monophosphate (Supplementary Figure 121).

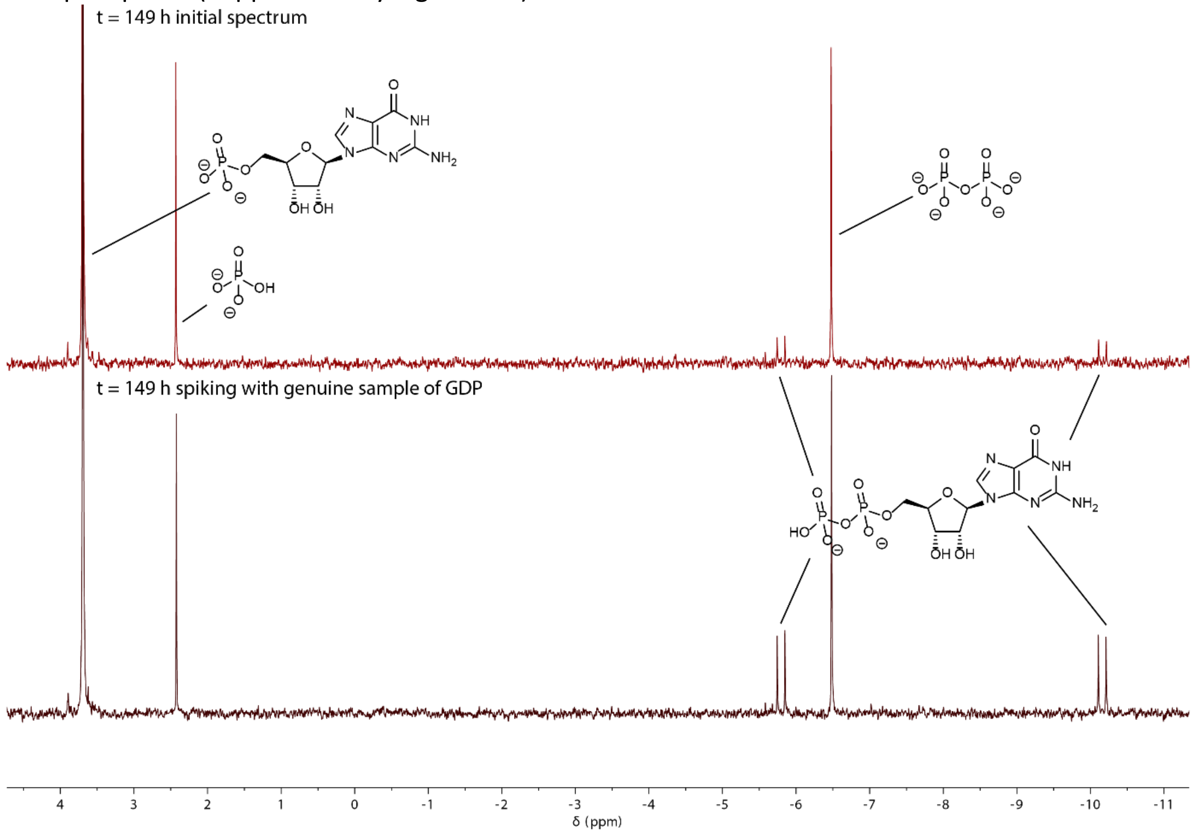

Supplementary Figure 121: ^{31}P NMR spectra of a spiking experiment for the reaction of 0.13 mmol calcium imidazole phosphate + 0.65 mmol guanosine-5'-monophosphate in paste conditions at 50 °C. Top spectrum is unspiked. Bottom spectrum is with the addition of genuine guanosine-5'-diphosphate.

S6.6.5.1 Control reaction for the phosphorylation of guanosine-5'-monophosphate

A control reaction was performed in the absence of calcium imidazole phosphate in order to determine if any guanosine-5'-diphosphate could have formed from a phosphate transfer reaction from one guanosine-5'-monophosphate molecule to another. Supplementary Figure 122 showed that no GDP was formed from the reaction of guanosine-5'-monophosphate with itself. Only a minor amount of hydrolysis of GMP to produce orthophosphate was observed.

The reaction was performed using 33.1 mg of guanosine-5'-monophosphate (0.0813 mmol) and an identical procedure was used as detailed in Section S6.6.1.1 except that after drying to a paste the resulting dried solid was rewetted using 0.2 µL / mg of D_2O.

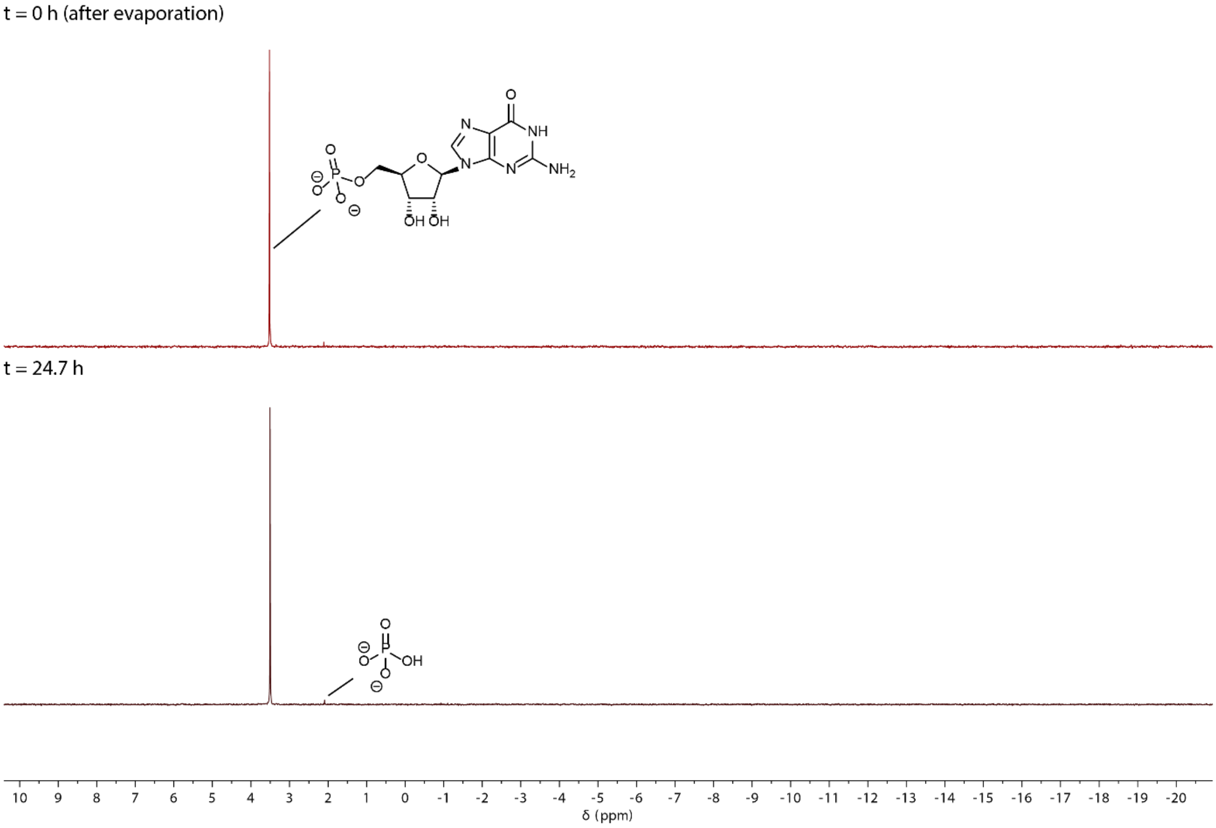

Supplementary Figure 122: ^{31}P NMR spectra of a control reaction performed by heating 0.081 mmol guanosine-5'-monophosphate in paste conditions at 50 °C in the absence of calcium imidazole phosphate.

S6.6.6 *Phosphorylation of guanosine-5'-diphosphate*

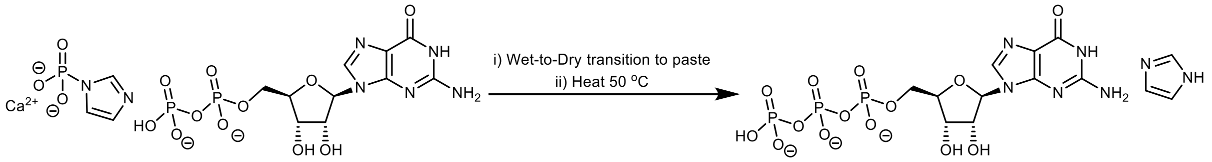

This reaction was performed according to the procedure in Section S6.1 but with the ribose replaced by 306.3 mg (0.65 mmol) guanosine-5'-diphosphate disodium salt hydrate.

Formation of guanosine-5'-triphosphate occurred in the paste from the reaction between imidazole phosphate and guanosine-5'-diphosphate with the highest observed yield of 18.4 % after drying to a paste at 0 h (Supplementary Figure 123 and S124). Excessive heating of the paste did cause hydrolysis of both the GDP to GMP and hydrolysis of formed GTP (Supplementary Figure 124).

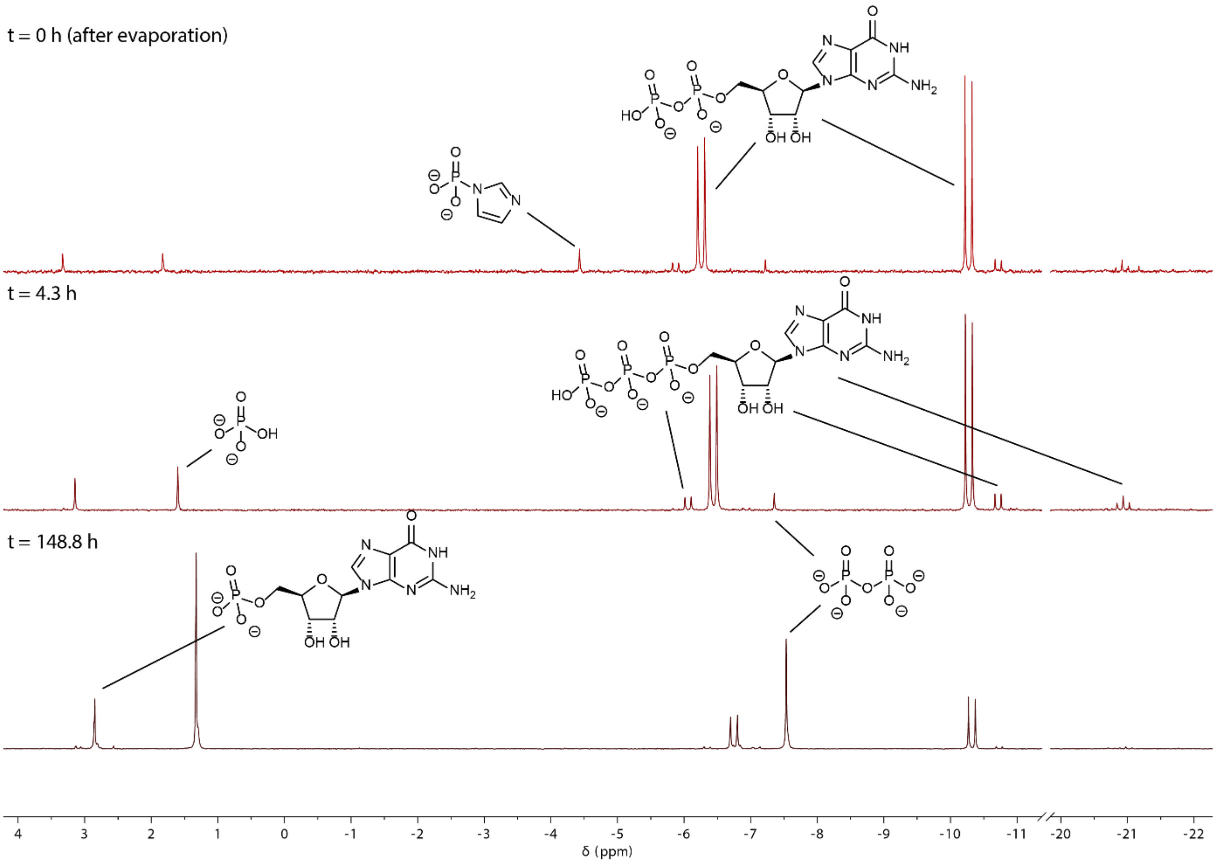

Supplementary Figure 123: Representative ^{31}P NMR spectra over time for the reaction of 0.13 mmol calcium imidazole phosphate + 0.65 mmol guanosine-5'-diphosphate in paste conditions at 50 °C. Top spectrum was taken after evaporation to form a paste.

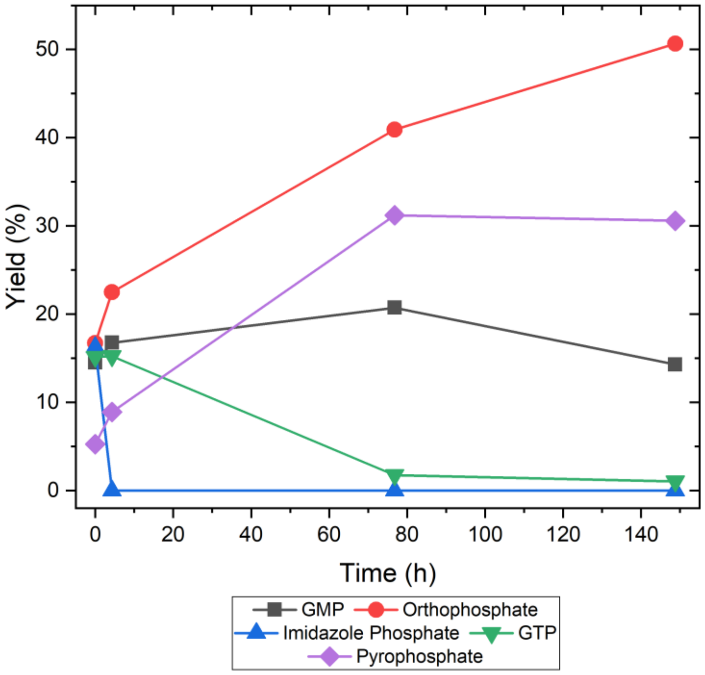

Supplementary Figure 124: Changes in yield over time for the reaction of 0.13 mmol calcium imidazole phosphate + 0.65 mmol guanosine-5’-diphosphate in paste conditions at 50 °C.

A spiking experiment with a genuine sample of guanosine-5’-triphosphate confirmed the formation of guanosine-5’-triphosphate from the reaction of calcium imidazole phosphate and guanosine-5’-diphosphate (Supplementary Figure 125).

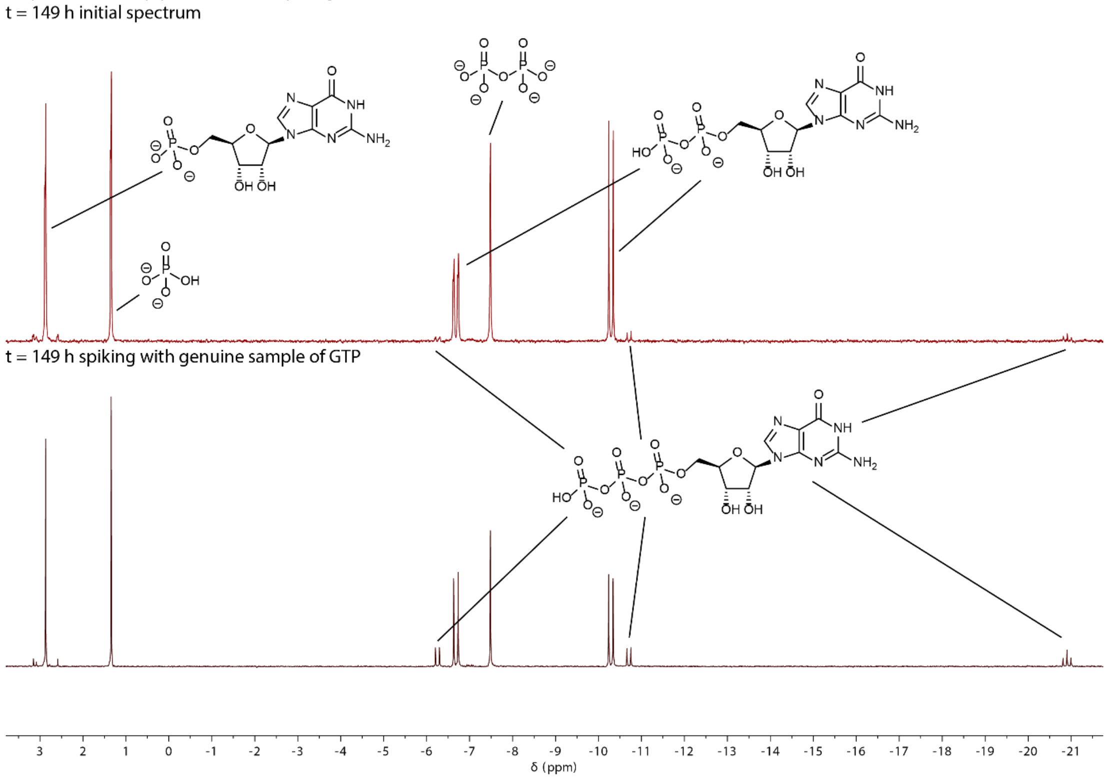

Supplementary Figure 125: ^{31}P NMR spectra of a spiking experiment for the reaction of 0.13 mmol calcium imidazole phosphate + 0.65 mmol guanosine-5’-diphosphate in paste conditions at 50 °C. Top spectrum is unspiked. Bottom spectrum is with the addition of genuine guanosine-5’-triphosphate.

S6.6.6.1 Control reaction for the phosphorylation of guanosine-5'-diphosphate

A control reaction was performed in the absence of calcium imidazole phosphate in order to determine if any guanosine-5'-triphosphate could have formed from a phosphate transfer reaction from one guanosine-5'-diphosphate molecule to another. Supplementary Figure 126 showed that GTP was formed from the reaction of guanosine-5'-diphosphate with itself in a 3.0 % yield after heating at 50 °C for 24.7 h. However, this yield is considerably lower than the 18.4 % yield recorded for the reaction in the presence of imidazole phosphate indicating that the GTP was predominantly formed from a reaction between imidazole phosphate and GDP.

The reaction was performed using 39.4 mg of guanosine-5'-diphosphate (0.0813 mmol) and an identical procedure was used as detailed in Section S6.6.1.1 except that after drying to a paste the resulting dried solid was rewetted using 0.2 µL / mg of D_2O.

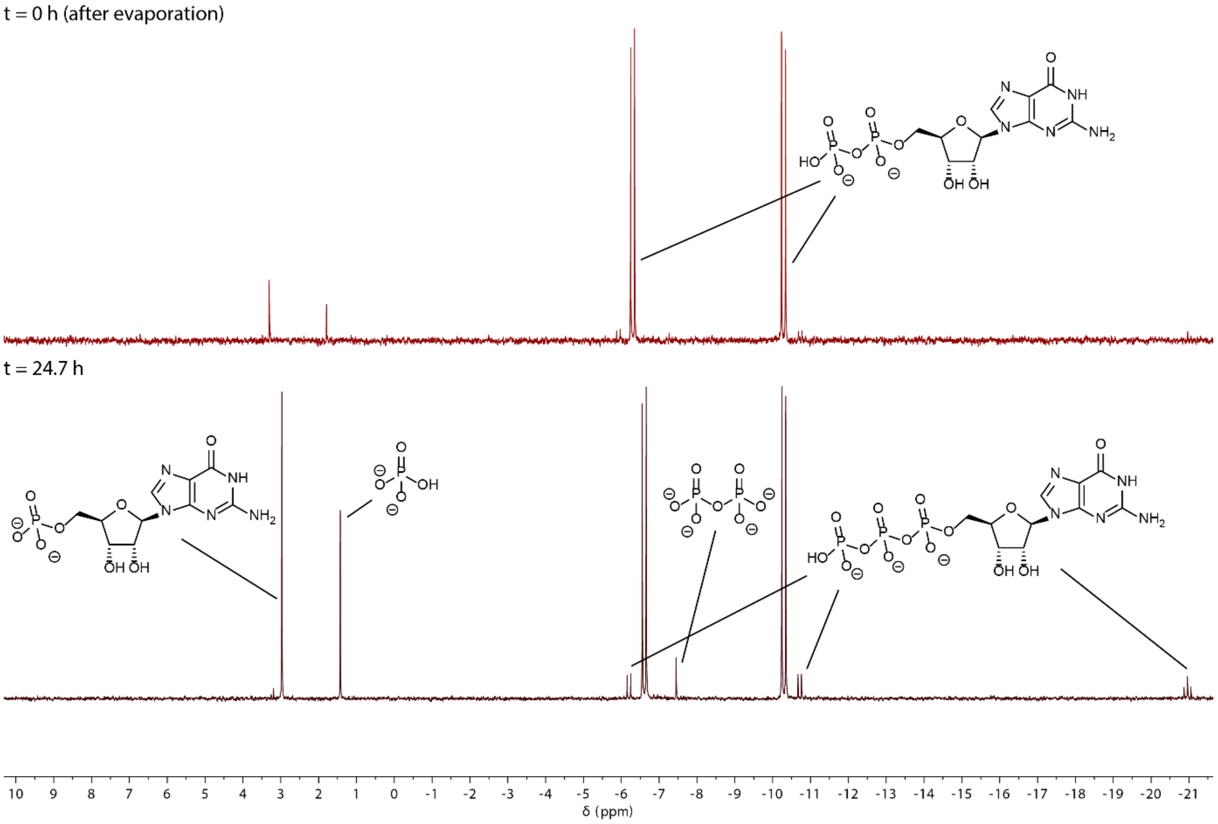

Supplementary Figure 126: ^{31}P NMR spectra of a control reaction performed by heating 0.0813 mmol guanosine-5'-diphosphate in paste conditions at 50 °C in the absence of calcium imidazole phosphate.

S6.6.7 Phosphorylation of uridine-5'-monophosphate

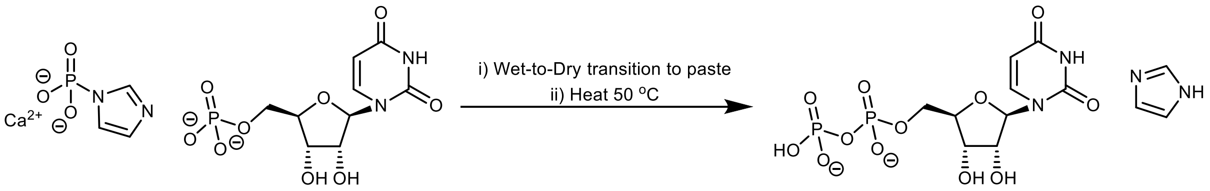

This reaction was performed according to the procedure in Section S6.1 but with the ribose replaced by 239.0 mg (0.65 mmol) uridine-5'-monophosphate disodium salt.

Formation of uridine-5'-diphosphate occurred in the paste from the reaction between imidazole phosphate and uridine-5'-monophosphate with the highest observed yield of 4.1 % after drying to a paste at 0 h (Supplementary Figure 127 and 128). Excessive heating of the paste did cause hydrolysis of the UDP formed (Supplementary Figure 128).

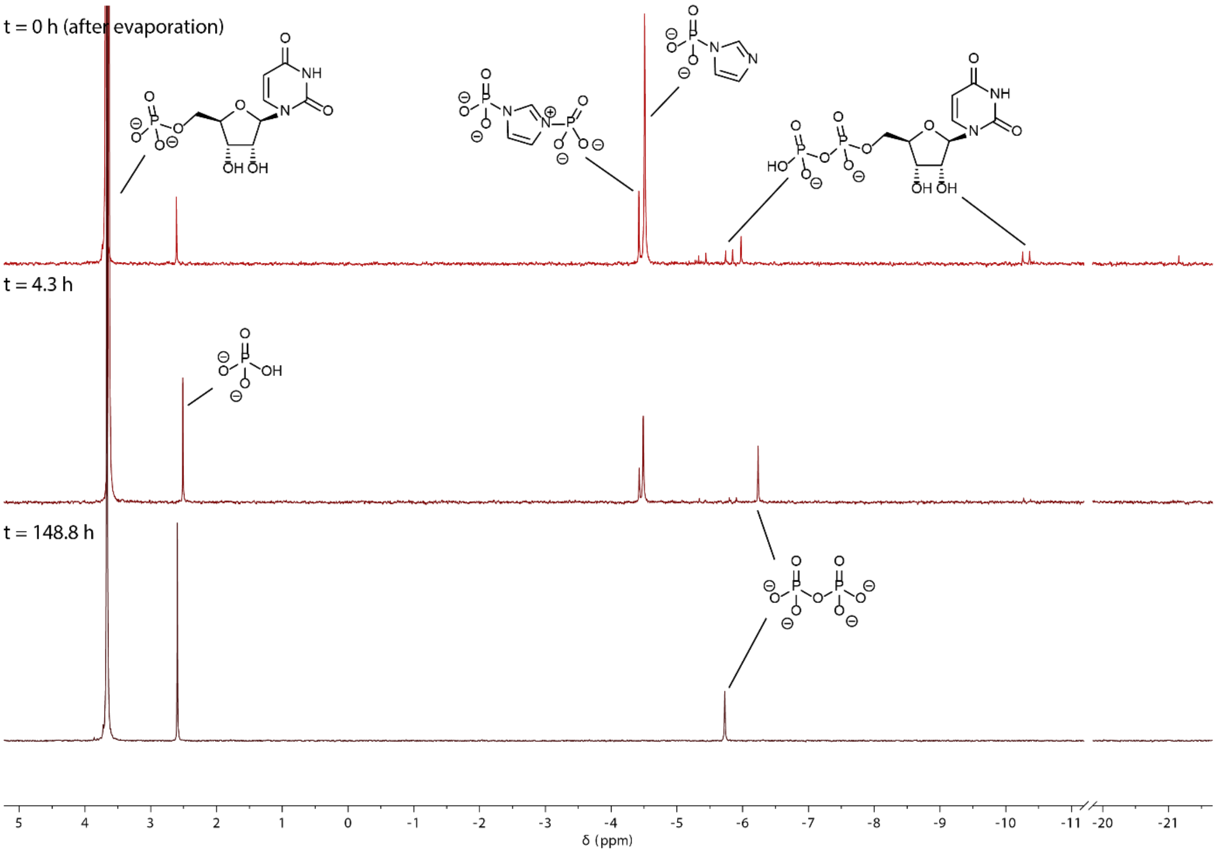

Supplementary Figure 127: Representative ^{31}P NMR spectra over time for the reaction of 0.13 mmol calcium imidazole phosphate + 0.65 mmol uridine-5'-monophosphate in paste conditions at 50 °C. Top spectrum was taken after evaporation to form a paste.

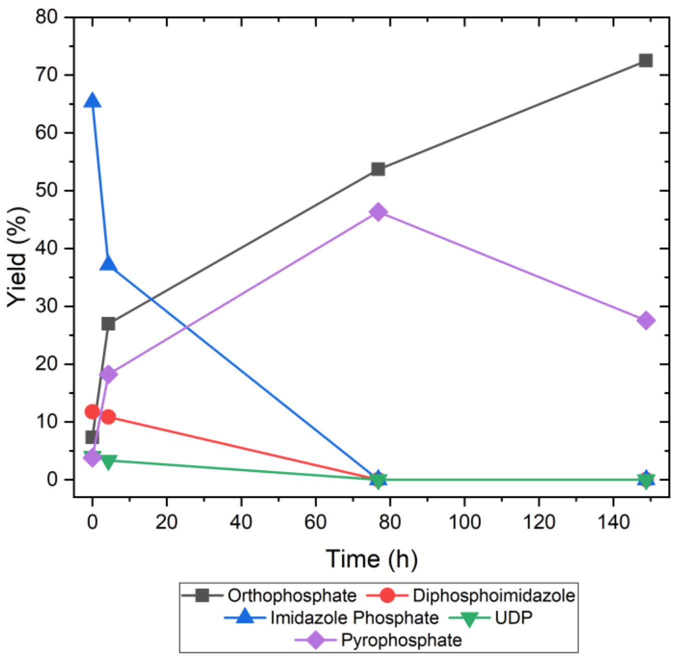

Supplementary Figure 128: Changes in yield over time for the reaction of 0.13 mmol calcium imidazole phosphate + 0.65 mmol uridine-5'-monophosphate in paste conditions at 50 °C.

A spiking experiment with a genuine sample of uridine-5'-diphosphate confirmed the formation of uridine-5'-diphosphate from the reaction of calcium imidazole phosphate and uridine-5'-monophosphate (Supplementary Figure 129).

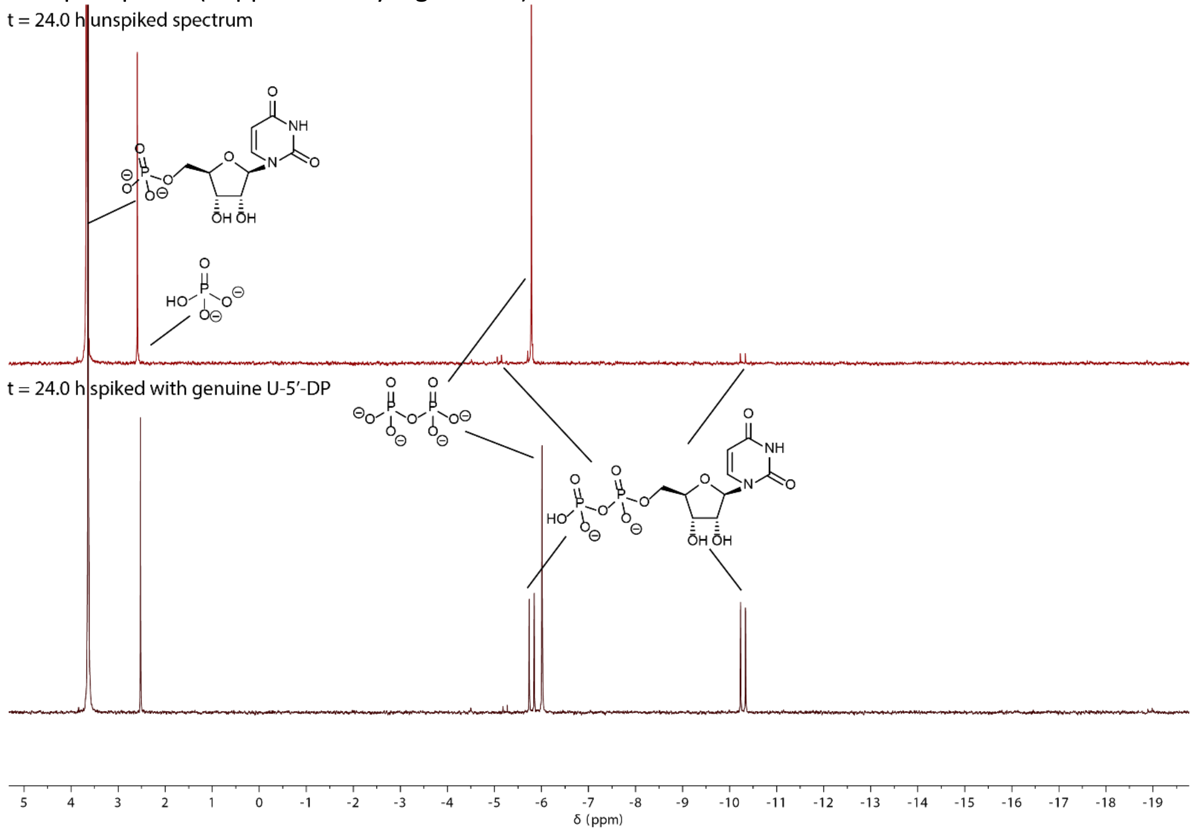

Supplementary Figure 129: ^{31}P NMR spectra of a spiking experiment for the reaction of 0.13 mmol calcium imidazole phosphate + 0.65 mmol uridine-5'-monophosphate in paste conditions at 50 °C. Top spectrum is unspiked. Bottom spectrum is with the addition of genuine uridine-5'-diphosphate.

S6.6.7.1 Control reaction for the phosphorylation of uridine-5′-monophosphate

A control reaction was performed in the absence of calcium imidazole phosphate in order to determine if any uridine-5′-diphosphate could have formed from a phosphate transfer reaction from one uridine-5′-monophosphate molecule to another. Supplementary Figure 130 showed that no UMP was formed from the reaction of uridine-5′-monophosphate with itself.

The reaction was performed using 29.7 mg of uridine-5′-monophosphate (0.0813 mmol) and an identical procedure was used as detailed in Section S6.6.1.1.

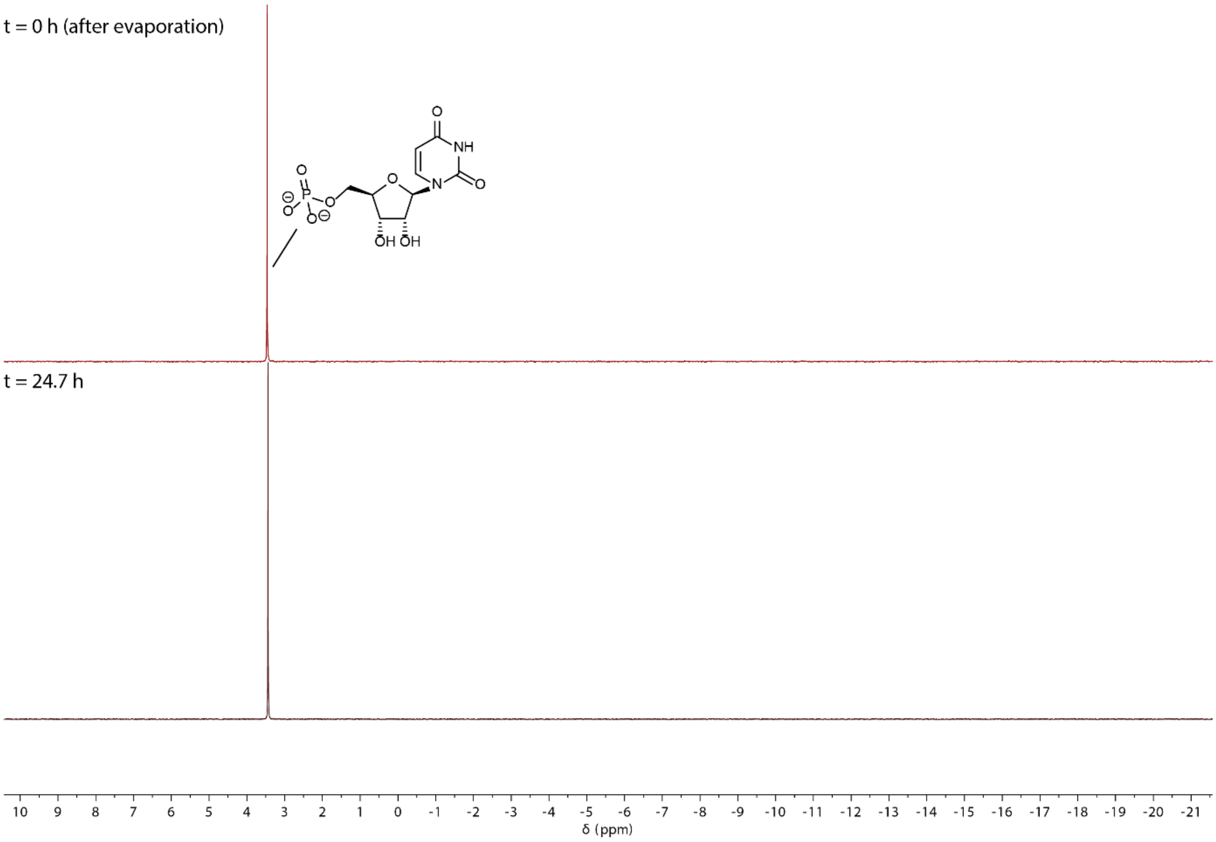

Supplementary Figure 130: ^{31}P NMR spectra of a control reaction performed by heating 0.081 mmol uridine-5′-monophosphate in paste conditions at 50 °C in the absence of calcium imidazole phosphate.

S6.6.8 Phosphorylation of uridine-5'-diphosphate

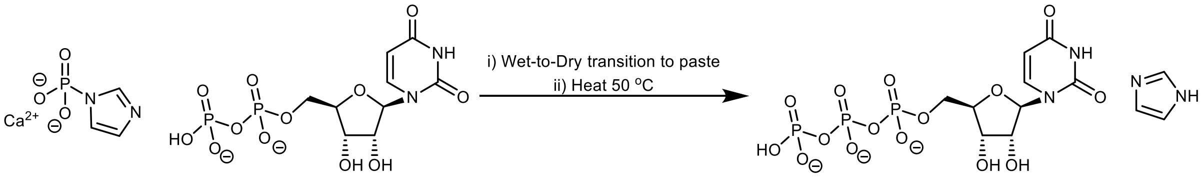

This reaction was performed according to the procedure in Section S6.1 but with the ribose replaced by 291.2 mg (0.65 mmol) uridine-5'-diphosphate disodium salt hydrate.

Formation of uridine-5'-triphosphate occurred in the paste from the reaction between imidazole phosphate and uridine-5'-diphosphate with the highest observed yield of 25.1 % after drying to a paste at 0 h (Supplementary Figure 131 and 132). Excessive heating of the paste did cause hydrolysis of both the UDP to UMP and hydrolysis of formed UTP (Supplementary Figure 132).

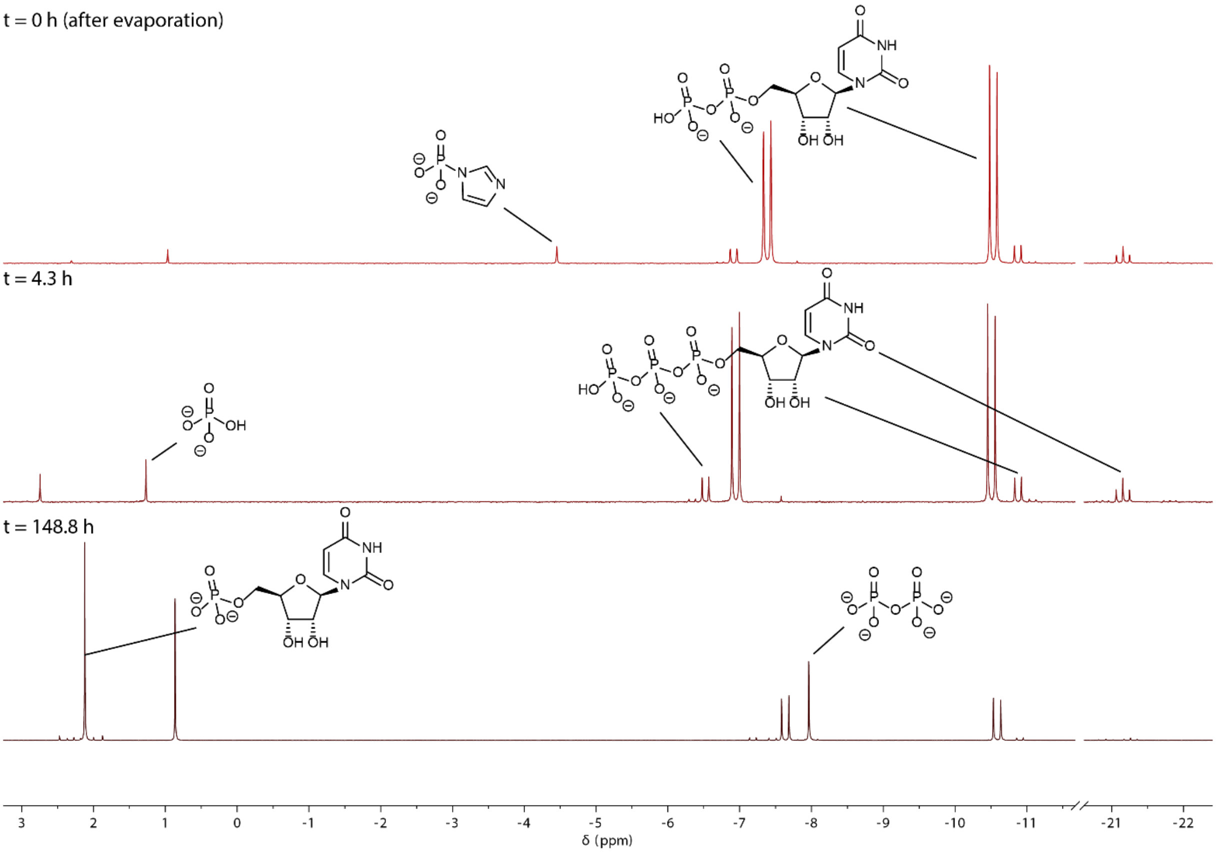

Supplementary Figure 132: Representative ^{31}P NMR spectra over time for the reaction of 0.13 mmol calcium imidazole phosphate + 0.65 mmol uridine-5'-diphosphate in paste conditions at 50 °C. Top spectrum was taken after evaporation to form a paste.

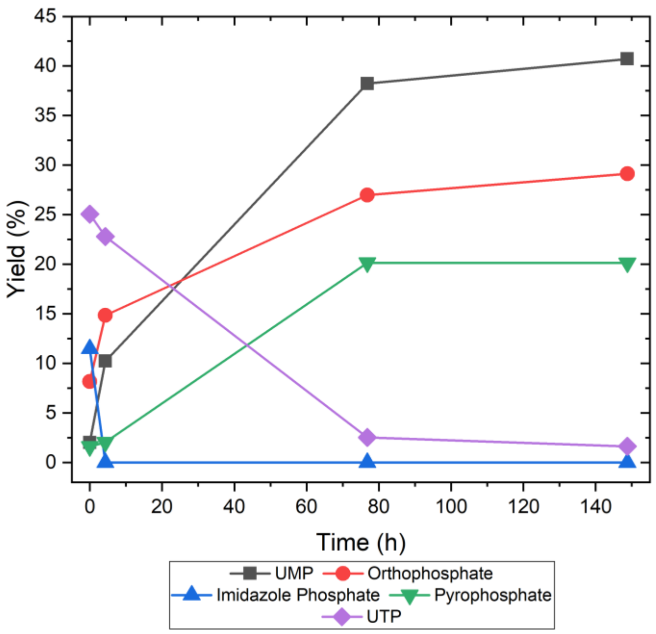

Supplementary Figure 132: Changes in yield over time for the reaction of 0.13 mmol calcium imidazole phosphate + 0.65 mmol uridine-5'-diphosphate in paste conditions at 50 °C.

A spiking experiment with a genuine sample of uridine-5'-triphosphate confirmed the formation of uridine-5'-triphosphate from the reaction of calcium imidazole phosphate and uridine-5'-diphosphate (Supplementary Figure 133).

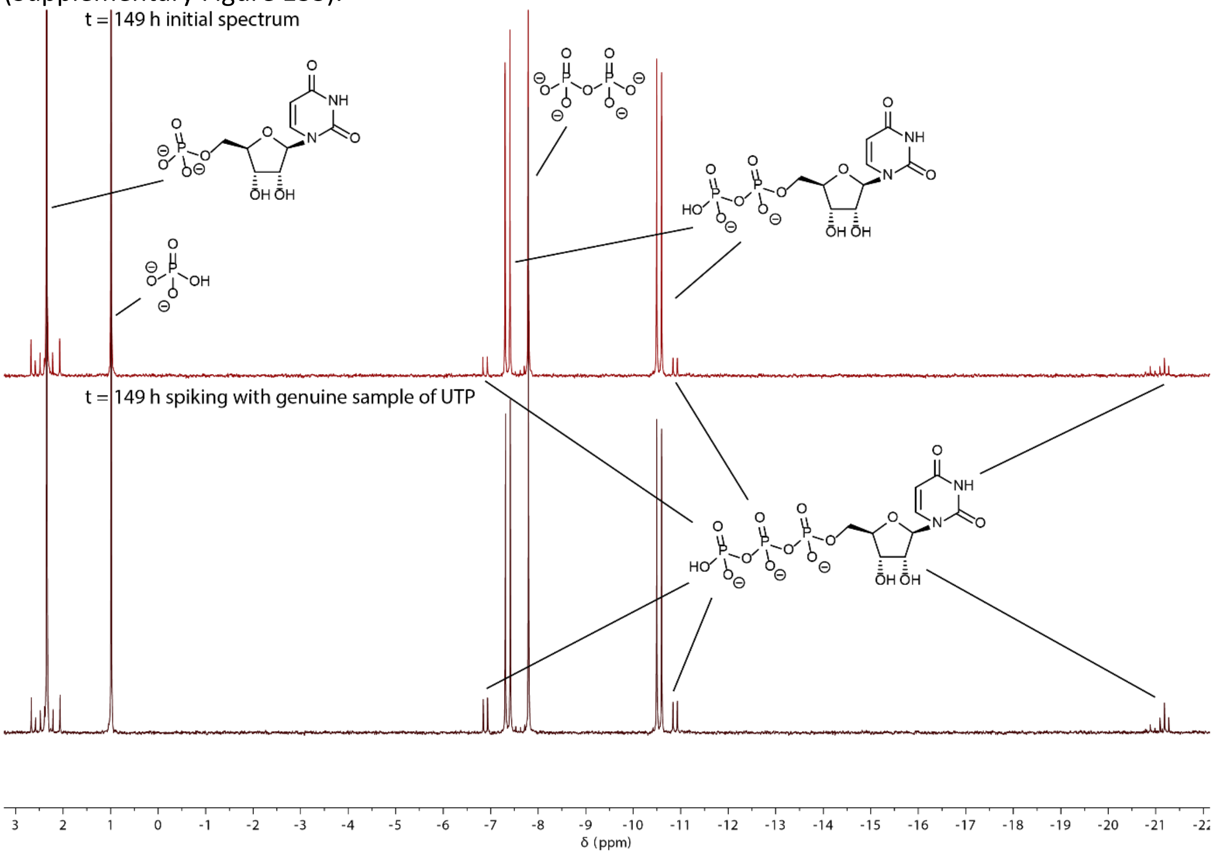

Supplementary Figure 133: ^{31}P NMR spectra of a spiking experiment for the reaction of 0.13 mmol calcium imidazole phosphate + 0.65 mmol uridine-5'-diphosphate in paste conditions at 50 °C. Top spectrum is unspiked. Bottom spectrum is with the addition of genuine uridine-5'-triphosphate.

S6.6.8.1 Control reaction for the phosphorylation of uridine-5'-diphosphate

A control reaction was performed in the absence of calcium imidazole phosphate in order to determine if any uridine-5'-triphosphate could have formed from a phosphate transfer reaction from one uridine-5'-diphosphate molecule to another. Supplementary Figure 134 showed that no UTP was formed from the reaction of uridine-5'-diphosphate with itself.

The reaction was performed using 29.7 mg of uridine-5'-diphosphate (0.0813 mmol) and an identical procedure was used as detailed in Section S6.6.1.1.

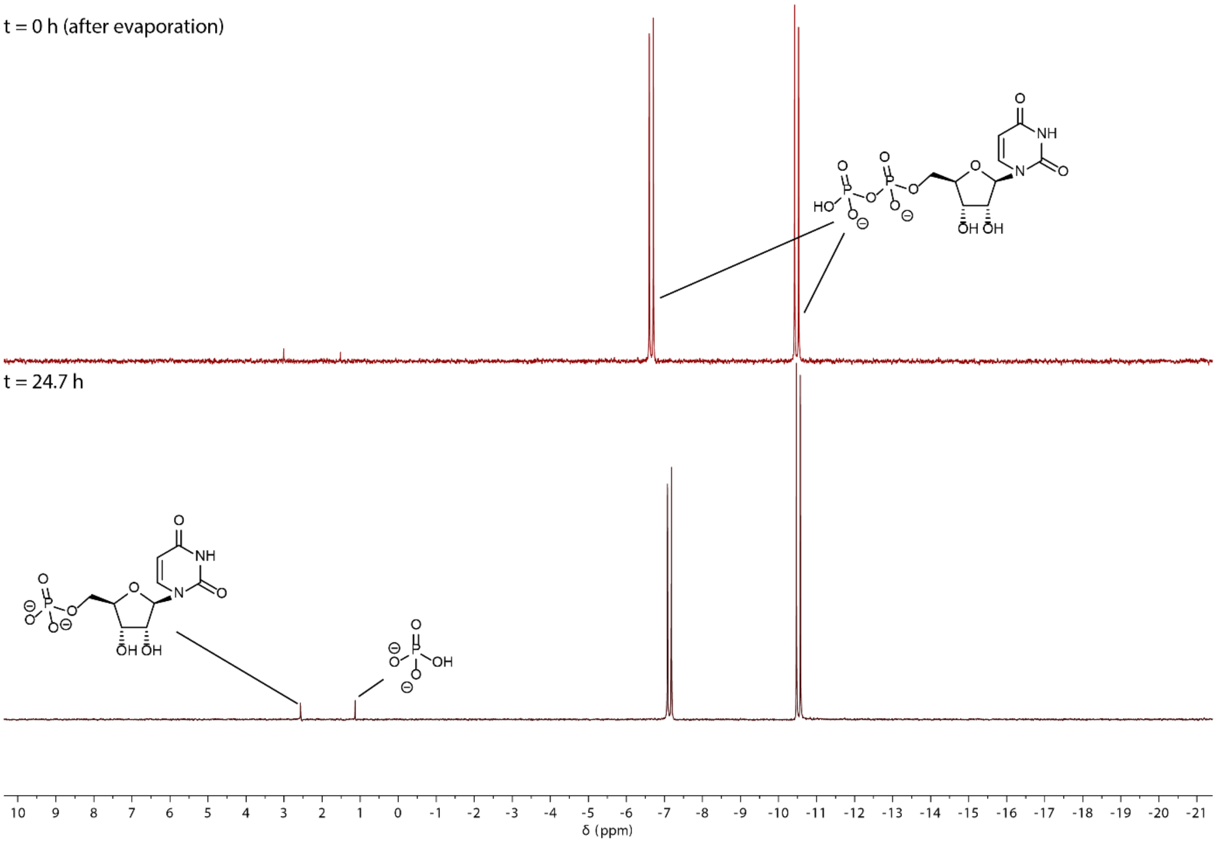

Supplementary Figure 134: ^{31}P NMR spectra of a control reaction performed by heating 0.0813 mmol uridine-5'-diphosphate in paste conditions at 50 °C in the absence of calcium imidazole phosphate.

S6.7 Phosphorylation of Sugars

S6.7.1 Phosphorylation of ribose

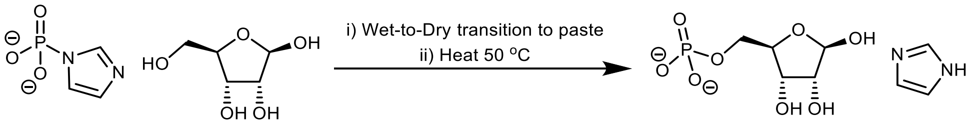

This reaction was performed according to the procedure in Section S6.1. Note that the use of the ribose-5-phosphate structure in the scheme above is to indicate phosphorylated ribose and not to identify the specific species.

Formation of phosphorylated ribose occurred in the paste from the reaction between imidazole phosphate and ribose. Multiple peaks for phosphorylated ribose were observed in the ^{31}P NMR spectra. The highest observed yield of 61 % ribose-phosphate was obtained after heating the paste at 50 °C for 167.3 h (Supplementary Figure 135a, 135b, 135c and 136).

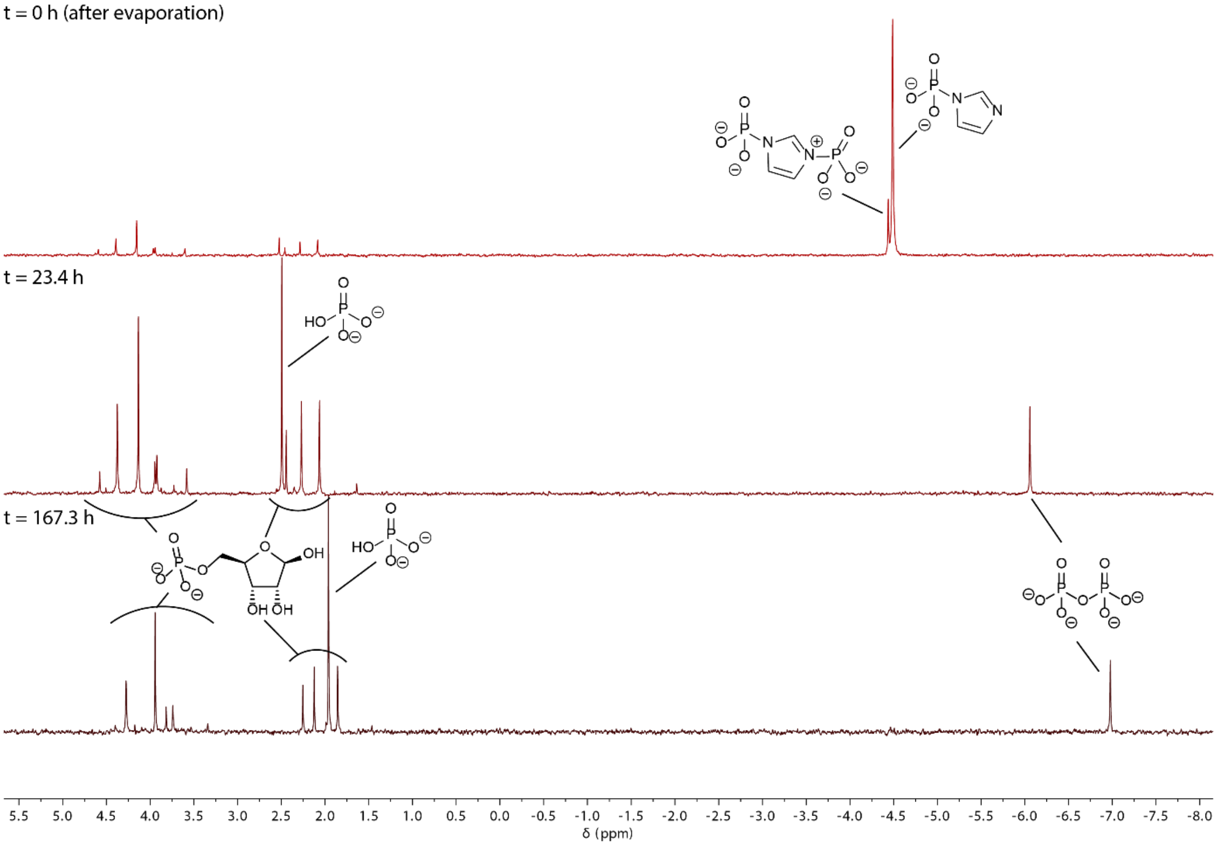

Supplementary Figure 135a: Representative ^{31}P NMR spectra over time for the reaction of 0.13 mmol calcium imidazole phosphate + 0.65 mmol ribose in paste conditions at 50 °C. Top spectrum was taken after evaporation to form a paste. All NMR spectra were measured in a 0.5 M citric acid buffer at pH 6.85 9 : 1 H_2O : D_2O. The chemical shift of the pyrophosphate peak is variable at pH 6.85 due to the pyrophosphate pK_a = 6.6. Use of ribose-5-phosphate structure is to indicate phosphorylated ribose and not to identify the specific species.

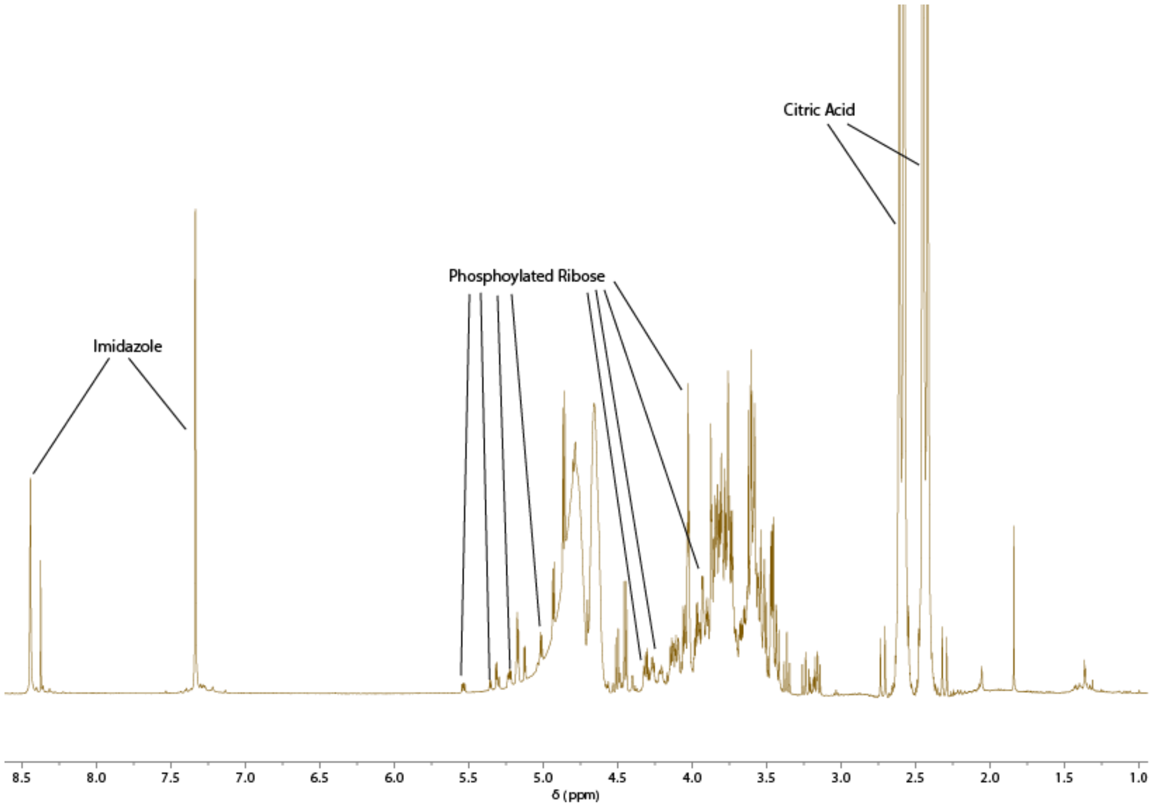

Supplementary Figure 135b: ^{31}P NMR spectrum at 167.3 h for the reaction of 0.13 mmol calcium imidazole phosphate + 0.65 mmol ribose in paste conditions at 50 °C.

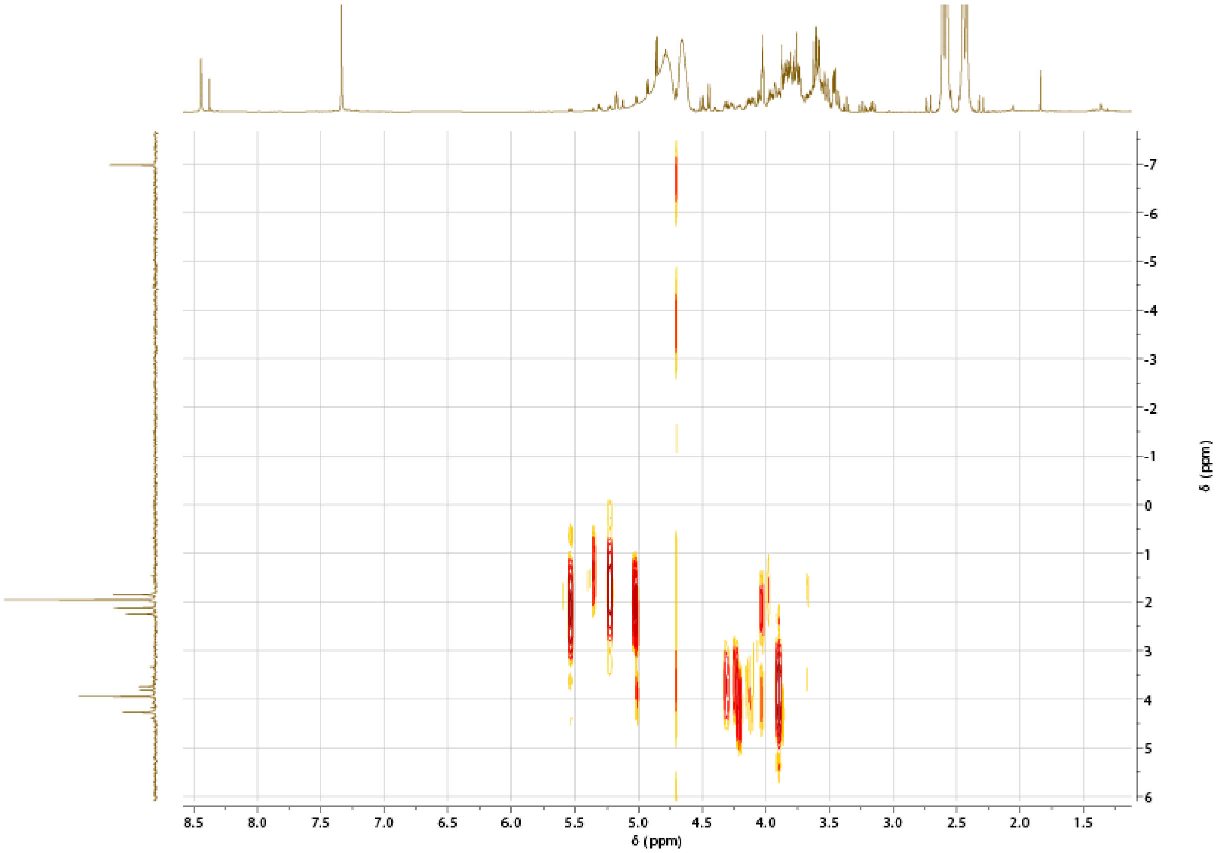

Supplementary Figure 135c: ^{1}H ^{31}P HMBC NMR spectrum at 167.3 h for the reaction of 0.13 mmol calcium imidazole phosphate + 0.65 mmol ribose in paste conditions at 50 °C.

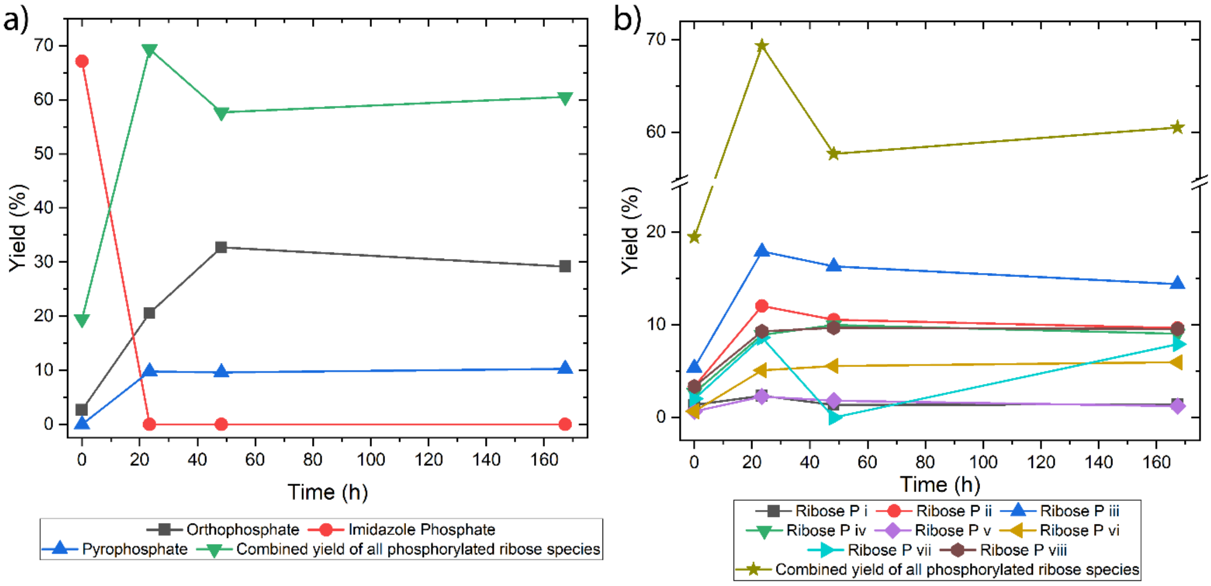

Supplementary Figure 136: Changes in yield over time for the reaction of 0.13 mmol calcium imidazole phosphate + 0.65 mmol ribose in paste conditions at 50 °C. For clarity the plot for this experiment is divided in two: a) All non-ribose based phosphate species and the combined yield of all phosphorylated ribose products. b) All phosphorylated ribose species and the combined yield of all phosphorylated ribose products.

A spiking experiment with genuine samples of ribose-1-phosphate and ribose-5-phosphate confirmed the formation of ribose-5-phosphate and seven other phosphorylated ribose species from the reaction of calcium imidazole phosphate and ribose (Supplementary Figure 137).

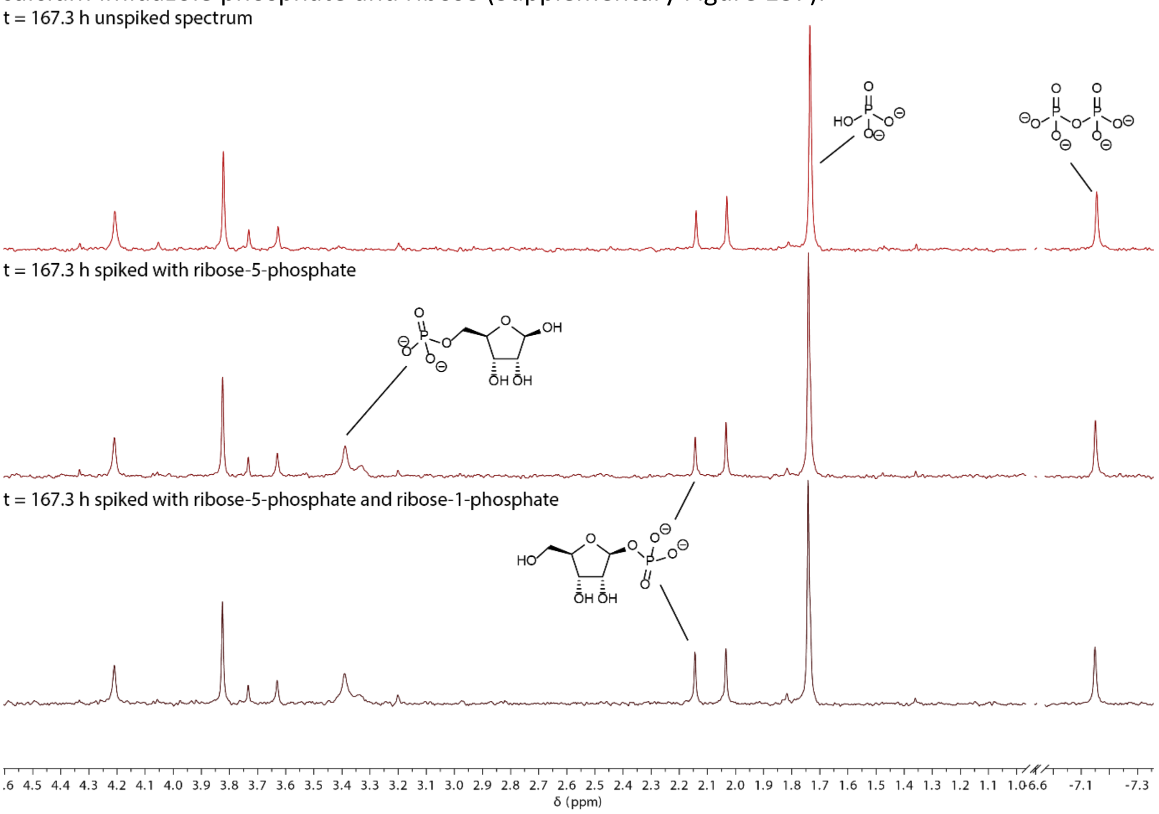

Supplementary Figure 137: ^{31}P NMR spectra of a spiking experiment for the reaction of 0.13 mmol calcium imidazole phosphate + 0.65 mmol ribose in paste conditions at 50 °C. Top spectrum is unspiked. Middle spectrum is with the addition of genuine ribose-5-phosphate. Bottom spectrum is with the addition of genuine ribose-1-phosphate.

S6.7.2 Phosphorylation of glucose

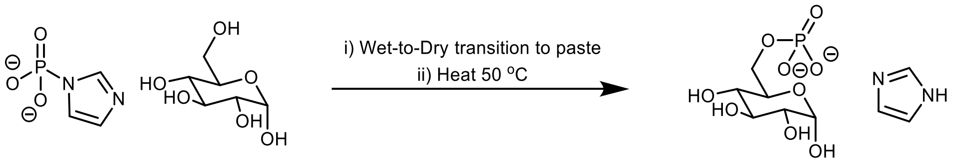

This reaction was performed according to the procedure in Section S6.1 but with the ribose replaced by 117.6 mg (0.65 mmol) glucose. Note that the use of the glucose-6-phosphate structure in the scheme above is to indicate phosphorylated glucose and not to identify the specific species.

Formation of phosphorylated glucose including glucose-1-phosphate and glucose-6-phosphate occurred in the paste from the reaction between imidazole phosphate and glucose with the highest observed yield of 39.3 % glucose phosphate and after heating paste at 50 °C for 23.4 h (Supplementary Figure 138a, 138b, 138c and 139).

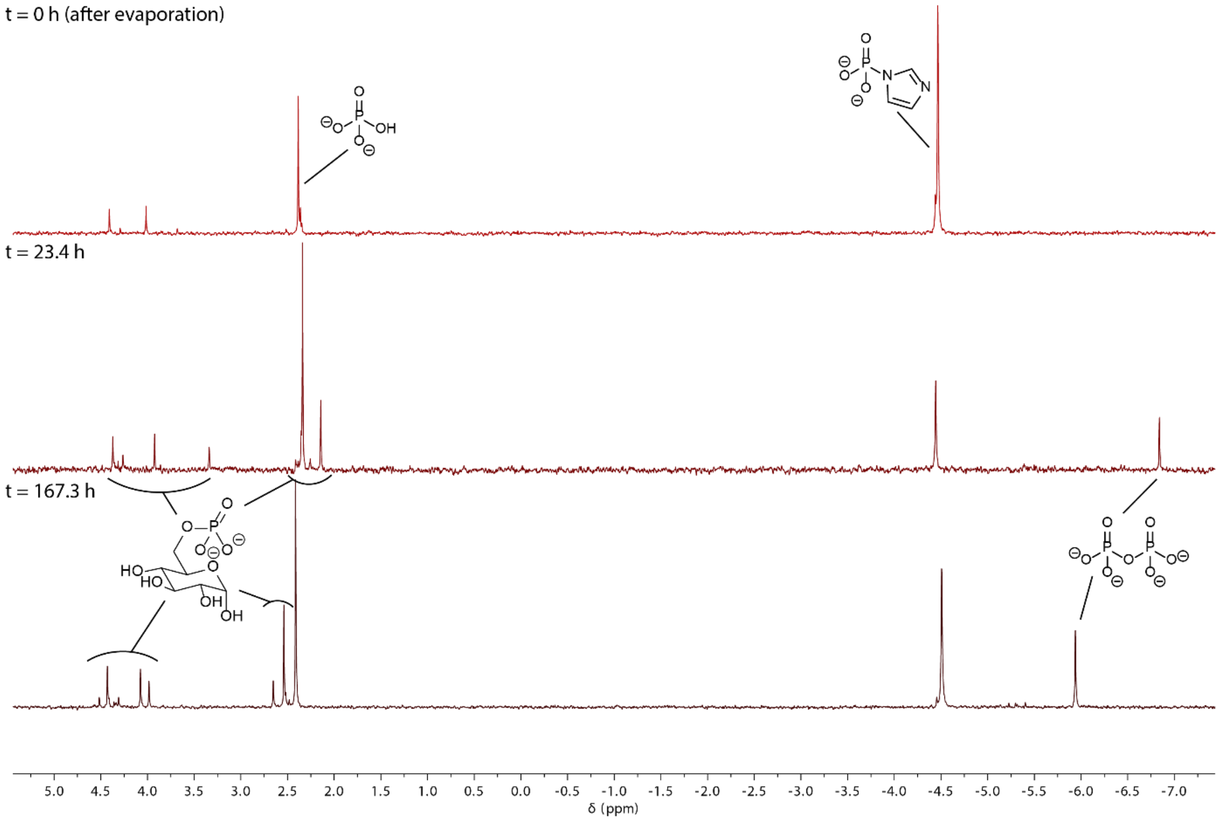

Supplementary Figure 138a: Representative ^{31}P NMR spectra over time for the reaction of 0.13 mmol calcium imidazole phosphate + 0.65 mmol glucose in paste conditions at 50 °C. Top spectrum was taken after evaporation to form a paste. All NMR spectra were measured in a 0.5 M citric acid buffer at pH 6.85 9 : 1 H_2O : D_2O. The chemical shift of the pyrophosphate peak is variable at pH 6.85 due to the pyrophosphate pK_a = 6.6. Use of glucose-6-phosphate structure is to indicate phosphorylated glucose and not to identify the specific species.

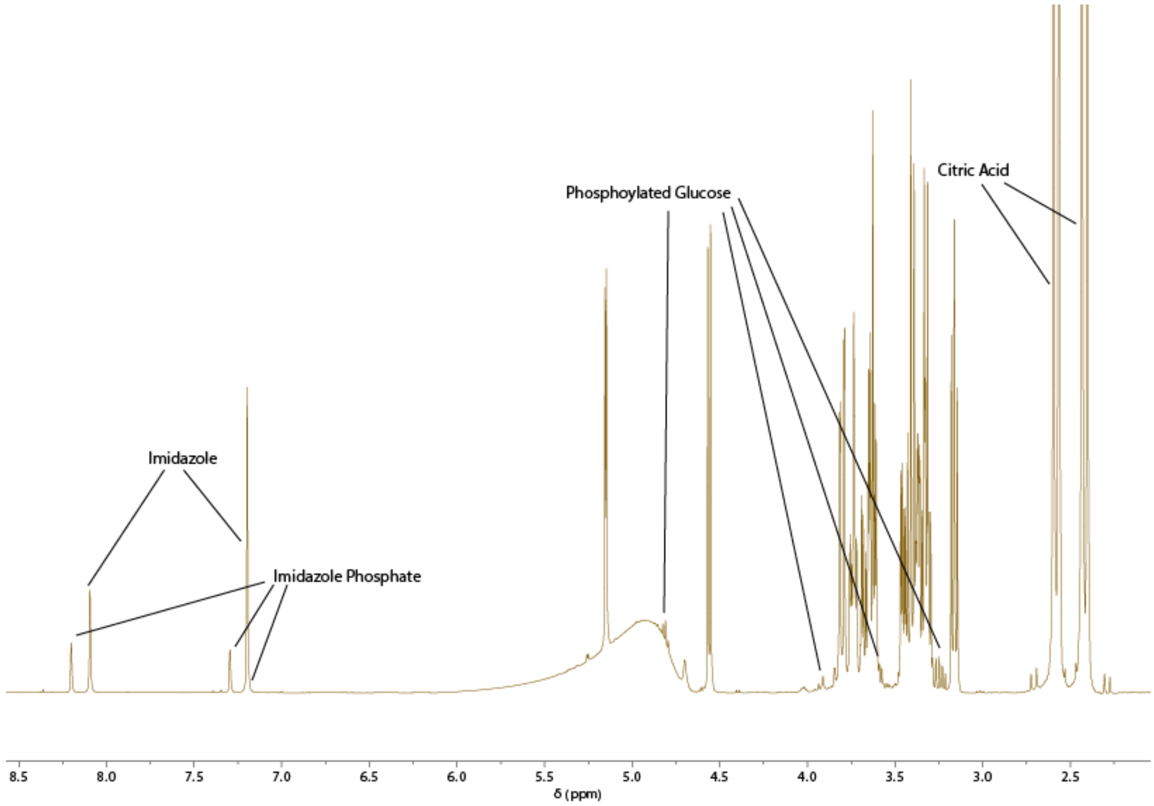

Supplementary Figure 138b: ^{1}H NMR spectra at 167.3 h for the reaction of 0.13 mmol calcium imidazole phosphate + 0.65 mmol glucose in paste conditions at 50 °C.

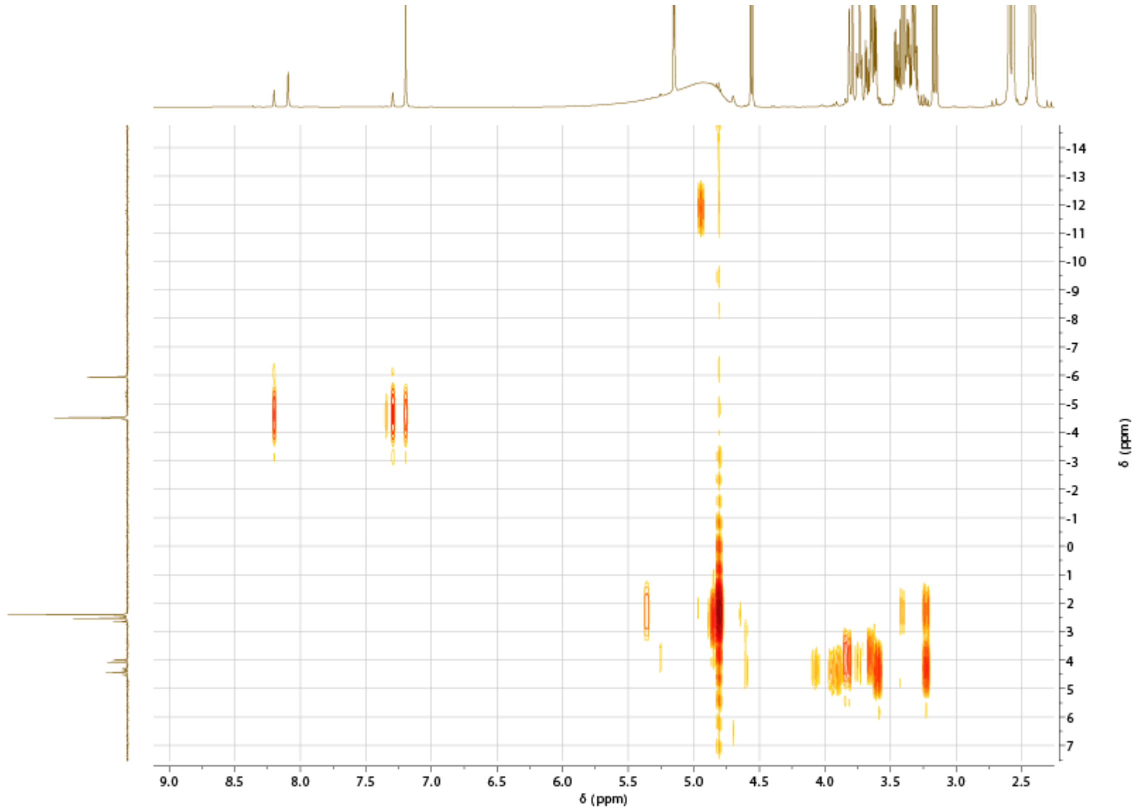

Supplementary Figure 138c: ^{1}H ^{31}P HMBC NMR spectra at 167.3 h for the reaction of 0.13 mmol calcium imidazole phosphate + 0.65 mmol glucose in paste conditions at 50 °C.

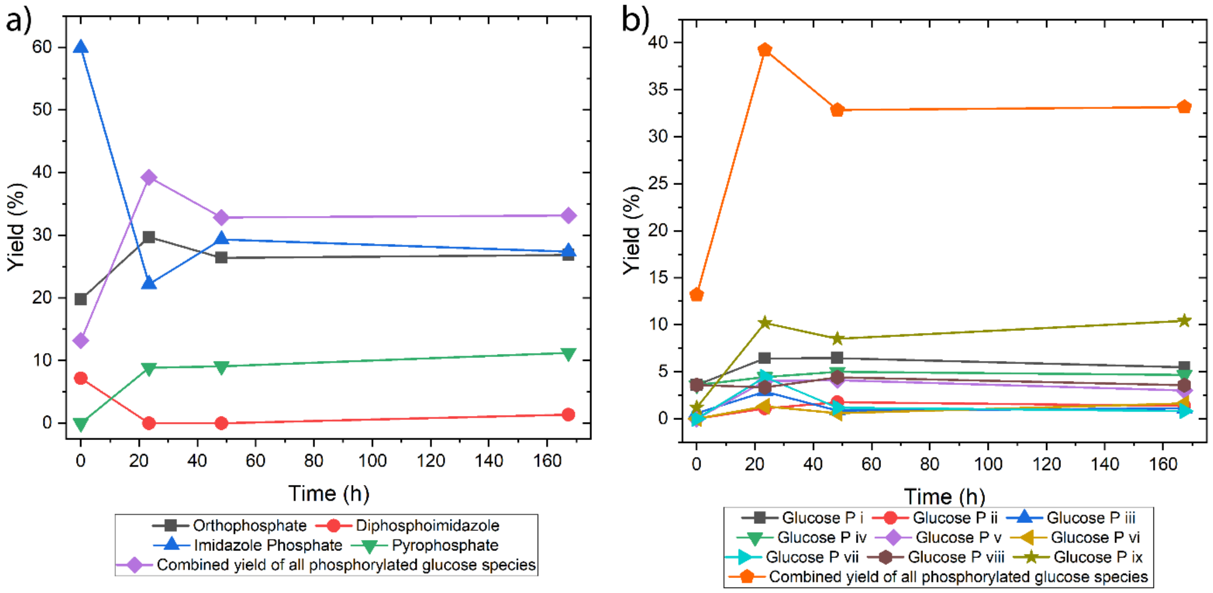

Supplementary Figure 139: Changes in yield over time for the reaction of 0.13 mmol calcium imidazole phosphate + 0.65 mmol glucose in paste conditions at 50 °C. For clarity the plot for this experiment is divided in two: a) All non-glucose based phosphate species and the combined yield of all phosphorylated glucose products. b) All phosphorylated glucose species and the combined yield of all phosphorylated glucose products.

A spiking experiment with genuine samples of glucose-1-phosphate and glucose-6-phosphate confirmed their formation and seven other phosphorylated glucose species from the reaction of calcium imidazole phosphate and glucose (Supplementary Figure 140).

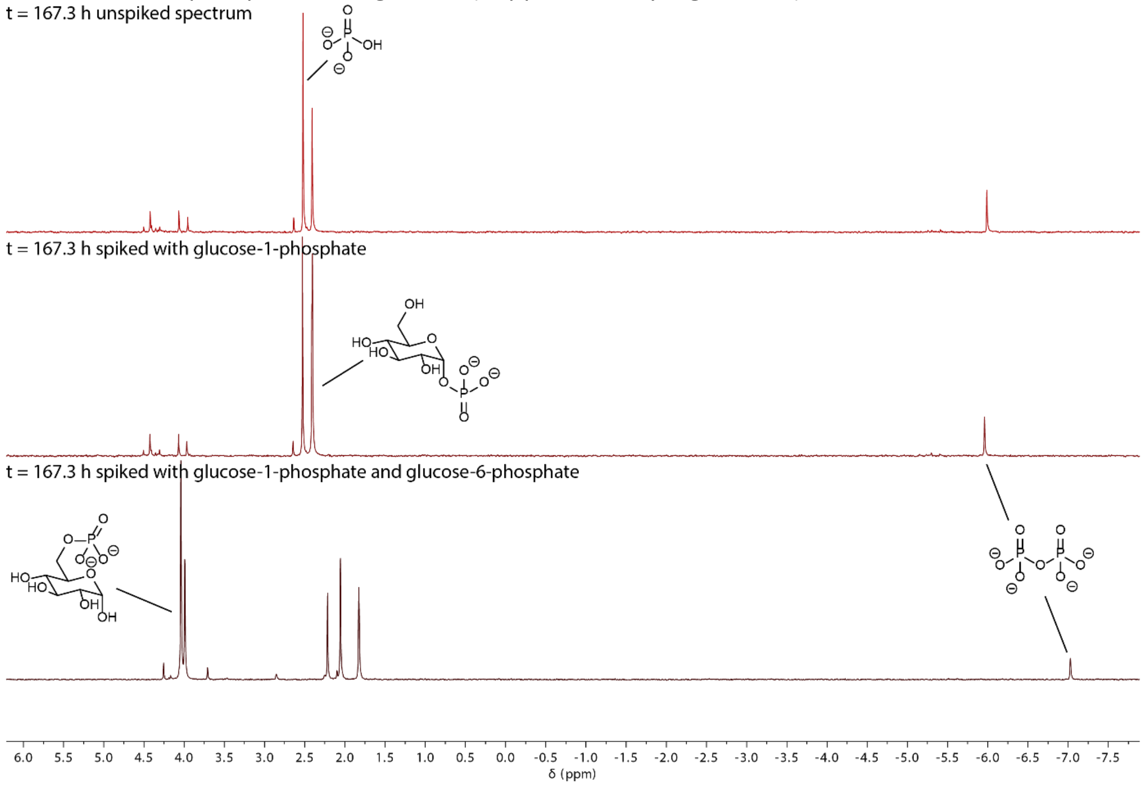

Supplementary Figure 140: ^{31}P NMR spectra of a spiking experiment for the reaction of 0.13 mmol calcium imidazole phosphate + 0.65 mmol glucose in paste conditions at 50 °C. Top spectrum is unspiked. Middle spectrum is with the addition of genuine glucose-1-phosphate. Bottom spectrum is with the addition of genuine glucose-6-phosphate.

S6.7.3 Phosphorylation of glucose

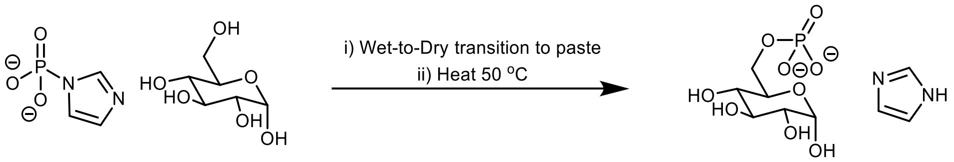

This reaction was performed according to the procedure in Section S6.1 but with the ribose replaced by 117.1 mg (0.65 mmol) glucose. Note that the use of the glucose-6-phosphate structure in the scheme above is to indicate phosphorylated glucose and not to identify the specific species.

Formation of phosphorylated glucose including glucose-1-phosphate and glucose-6-phosphate occurred in the paste from the reaction between imidazole phosphate and glucose with the highest observed yield of 70.0 % glucose phosphate and after heating paste at 50 °C for 24.0 h (Supplementary Figure 141 and 142).

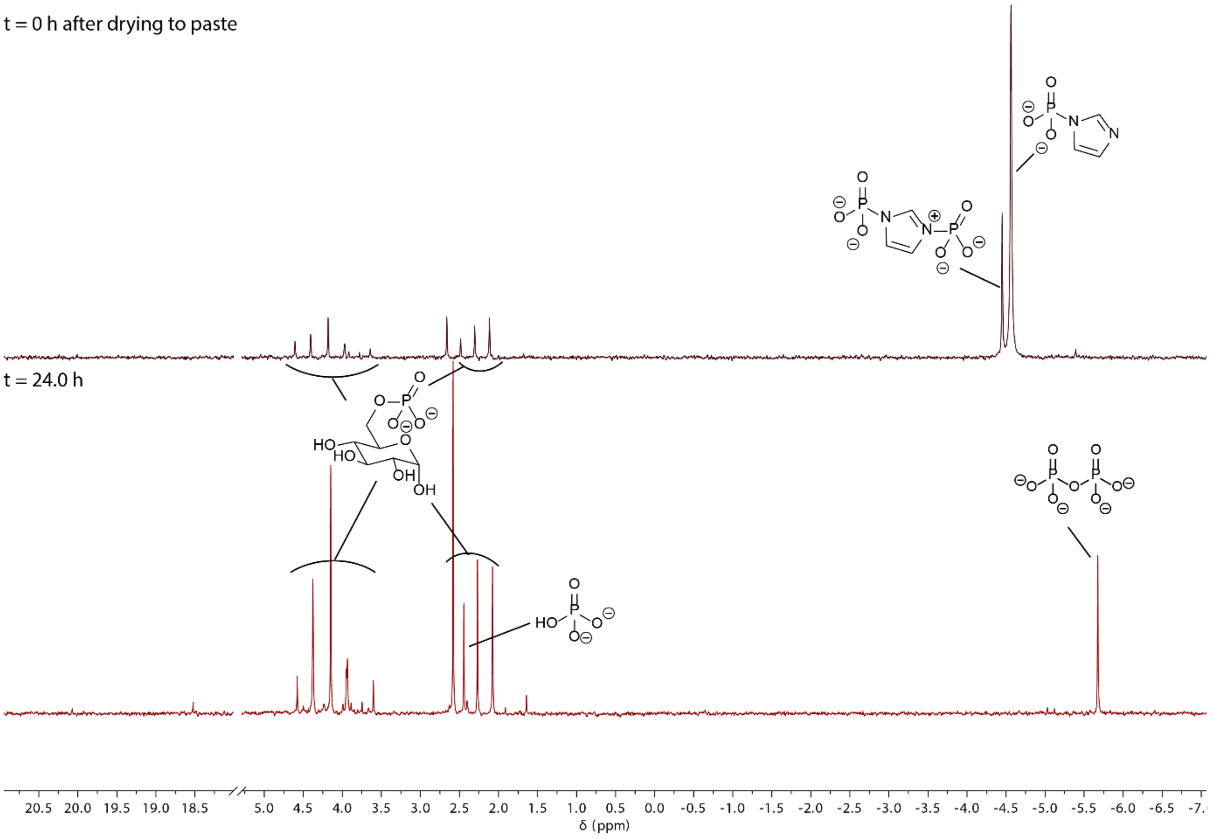

Supplementary Figure 141: Representative ^{31}P NMR spectra over time for the reaction of 0.13 mmol calcium imidazole phosphate + 0.65 mmol glucose in paste conditions at 50 °C. Top spectrum was taken after evaporation to form a paste. All NMR spectra were measured in a 0.5 M citric acid buffer at pH 6.85 9 : 1 H_2O : D_2O. Use of glucose-6-phosphate structure is to indicate phosphorylated glucose and not to identify the specific species.

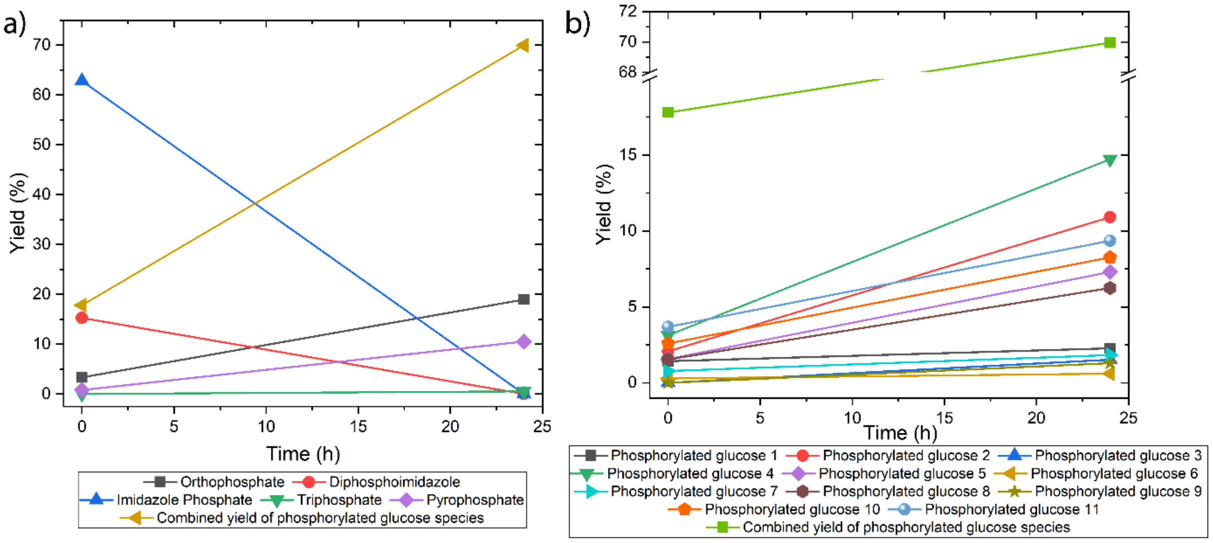

Supplementary Figure 142: Changes in yield over time for the reaction of 0.13 mmol calcium imidazole phosphate + 0.65 mmol glucose in paste conditions at 50 °C. For clarity the plot for this experiment is divided in two: a) All non-glucose based phosphate species and the combined yield of all phosphorylated glucose products. b) All phosphorylated glucose species and the combined yield of all phosphorylated glucose products.

A spiking experiment with a genuine samples of glucose-1-phosphate and glucose-6-phosphate confirmed the formation of glucose-1-phosphate and ten other phosphorylated glucose species from the reaction of calcium imidazole phosphate and glucose (Supplementary Figure 143).

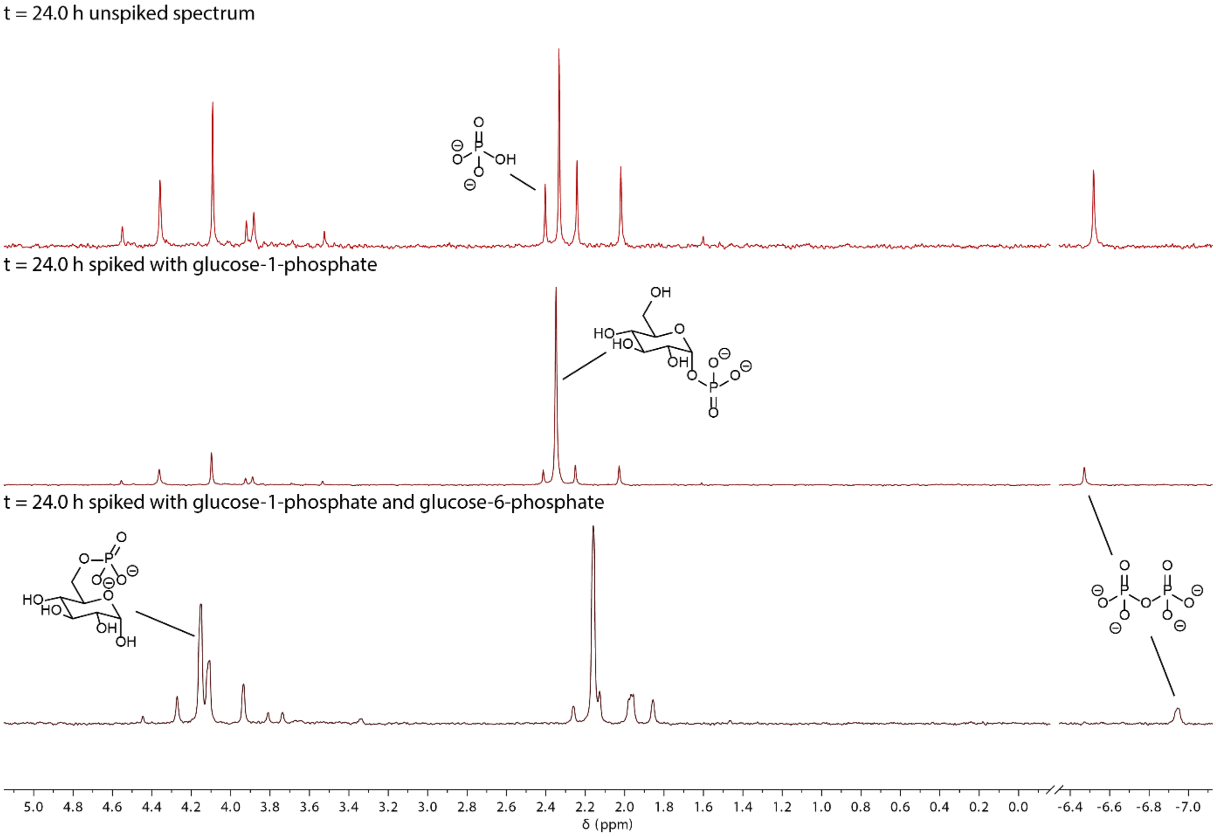

Supplementary Figure 143: ^{31}P NMR spectra of a spiking experiment for the reaction of 0.13 mmol calcium imidazole phosphate + 0.65 mmol glucose in paste conditions at 50 °C. Top spectrum is unspiked. Middle spectrum is with the addition of genuine glucose-1-phosphate. Bottom spectrum is with the addition of genuine glucose-6-phosphate.

S6.8 Results of all paste reactions and a comparison between the two methods used to perform the paste reaction.

Two different methods were used to prepare the pastes for the phosphorylation paste reaction between calcium imidazole phosphate and the nucleophiles. Namely, the procedure detailed in S5.1 where pastes were made by mixing solid calcium imidazole phosphate and solid/liquid nucleophile with a small amount of water (0.1 - 0.2 μL mg^{-1}), and, the procedure detailed in S6.1 where the calcium imidazole phosphate and nucleophile were dissolved in water and the solution was allowed to evaporate into a paste. In Supplementary Table 2 the yields of phosphorylated organic compounds for each method are summarised so that a comparison can made between the two methods.

Supplementary Table 2: The amphiphiles, amino acids + peptides, metabolites, nucleosides, nucleotides and sugars phosphorylated by imidazole phosphate under paste conditions (see SI Section S5 + S6).

	Nucleophile	Nucleophile (mmol)	Calcium Imidazole Phosphate (mmol)	Method	T (°C)	Duration (h)	Yield (%)
Amphiphile precurcors	Glycerol	0.13	0.13	S5.1	50	30	19 % Glycerol-1-phosphate<br>12 % Glycerol-2-phosphate
		0.65	0.13	S5.1	80	5	29 % Glycerol-1-phosphate<br>23 % Glycerol-2-phosphate
		0.65	0.13	S6.1	50	167	24 % Glycerol-1-phosphate<br>14 % Glycerol-2-phosphate<br>(6 % Glycerol-1,2-cyclic phosphate) [a]
	Pentane-1,2-diol	0.65	0.13	S5.1	80	54	34 % Pentane-1,2-diol-1-Phosphate<br>14 % Pentane-1,2-diol-2-Phosphate
Amino	Serine	0.65	0.13	S6.1	50	46	17 % O-Phosphoserine
	SerOMe.HCl	0.65	0.13	S6.1	50	169	9 % O-Phosphoserine

							2 % O-Phosphoserine methyl ester
	ThrOMe.HCl	0.65	0.13	S6.1	50	48	7 % O-Phosphothreonine
	Ser-His	0.33	0.13	S6.1	50	46	6 % O-PhosphoSer-His
Metabolites	Calcium Glycerate	0.65	0.13	S5.1	80	54	8 % 2-Phosphoglycerate<br>16 % 3-Phosphoglycerate
		0.65	0.13	S6.1	50	167	26 % 2-Phosphoglycerate<br>8 % 3-Phosphoglycerate
	Sodium Glycerate	0.65	0.13	S6.1	50	23	9 % 2-Phosphoglycerate<br>16 % 3-Phosphoglycerate
	Sodium Lactate	0.65	0.13	S5.1	80	22	11 % 2-Phospholactate
		0.65	0.13	S6.1	50	24	10 % 2-Phospholactate
	Sodium Glycolate	0.65	0.13	S5.1	80	54	15 % 2-Phosphoglycolate
		0.65	0.13	S6.1	50	24	31 % 2-Phosphoglycolate
	Lactamide	0.65	0.13	S6.1	50	167	11 % Phospholactamide<br>7 % Pyrophospholactamide
Nucleosides	Adenosine	0.65	0.13	S6.1	50	22	2 % A-5’-MP
	Cytidine	0.65	0.13	S6.1	50	93	7 % C-2’-MP<br>4 % C-3’-MP<br>3 % C-5’-MP
	Guanosine	0.65	0.13	S6.1	50	93	1 % G-5’-MP
	Uridine	0.65	0.13	S6.1	50	22	11 % U-5’-MP<br>7 % U-3’-MP
Nucleotides	AMP	0.13	0.13	S5.1	80	54	9 % ADP
		0.65	0.13	S6.1	50	4	20 % ADP
	ADP	0.13	0.13	S5.1	80	165	4 % ATP
		0.65	0.13	S6.1	50	0 [c]	18 % ATP
	CMP	0.13	0.13	S5.1	80	165	12 % CDP<br>3 % CTP
		0.65	0.13	S6.1	50	5	4 % CDP
	CDP	0.65	0.13	S6.1	50	4	12 % CTP
	GMP	0.13	0.13	S5.1	80	165	4 % GDP
		0.65	0.13	S6.1	50	4	15 % GDP
	GDP	0.65	0.13	S6.1	50	0 [c]	18 % GTP
	UMP	0.65	0.13	S6.1	50	4	4 % UDP
	UDP	0.65	0.13	S6.1	50	0 [c]	25 % UTP

**Sugars**	D-Ribose	0.26	0.26	S5.1	50	22	42 % [d]
					80	21	19 % [d]
		0.65	0.13	S6.1	50	23	69 % [d]
	D-Glucose	0.26	0.26	S5.1	50	70	24 % [d]
					80	21	13 % [d]
		0.65	0.13	S6.1	50	24	70 % [d]
						48	33 % [d]

[a] The formation of Glycerol-1,2-cyclic phosphate was observed after 24 h. However, this signal disappeared after heating for 167 h (see Fig. S65). [b] Sodium salt of imidazole phosphate used. [c] t = 0 h is after drying to paste. [d] For ribose and glucose multiple peaks in the ^{31}P NMR spectra for phosphorylated sugars were observed (see Fig. S61-S64 and S135-S143). The yield quoted is the summation of integrals from all of these peaks.

S7 Demonstration of physicochemical orthophosphate cycle which recycles orthophosphate in a wet/dry cycle (Main Text Figure 3)

S7.1 Supplementary experimental Method for orthophosphate recycling with a wet/dry cycle

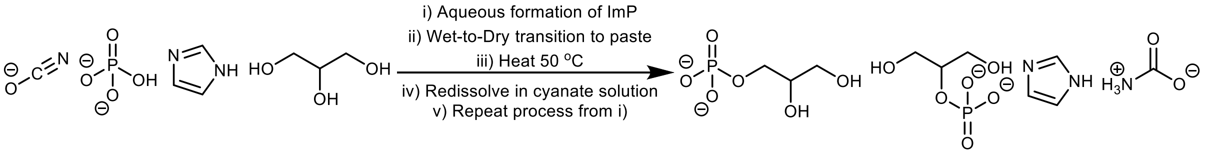

4.085 g (60 mmol) of imidazole was dissolved in 60 mL of 9 : 1 H_2O : D_2O to give a 1.0 M imidazole solution. 730.1 mg (9.0 mmol) of potassium cyanate and 534.0 mg (3.0 mmol) of sodium phosphate dibasic dihydrate salt were dissolved in 20.0 mL of 1.0 M imidazole solution to give a 450 mM potassium cyanate and a 150 mM sodium phosphate dibasic solution. 276.3 mg (3.0 mmol) of glycerol was dissolved in 4.0 mL of 450 mM potassium cyanate, 150 mM sodium phosphate dibasic and 1.0 M imidazole solution. The pH of the solution was measured and was typically in the region of pH 8.5-10.0. The pH of the solution was then adjusted to pH 7.3 using 5 M hydrochloric acid and 5 M sodium hydroxide solutions. The solution was left to react for 24 h in order to accumulate imidazole phosphate. 500 μL of the solution was removed and put in an NMR tube and the reaction was followed by ^{31}P-NMR and ^{1}H-NMR spectroscopy.

After 24 h the solution in the NMR tube was recombined with the rest of the solution and the solution was pipetted as small droplets into a petri dish (15 cm diameter) and left with the lid off to dry at 22 °C for 24 h. Small droplets were used so as to increase the surface area of the air/water interface in order to accelerate evaporation, see Supplementary Figure 144 for a photo. The resulting paste was then scraped from the petri dish and placed into a 2.5 mL Eppendorf (an NMR sample of the paste was also taken). The Eppendorf was then spun on a centrifuge for 3 min at 5590 g in order to move all solid to the bottom of the Eppendorf and compact it together. The Eppendorf was then placed on a thermoshaker at 50 °C and 600 rpm for 24 h. The paste reaction was followed with ^{31}P-NMR and ^{1}H-NMR spectroscopy. A sample of approximately 10 mg was removed periodically and dissolved in 0.5 mL of 0.5 M citric acid buffer at pH 6.85 9 : 1 H_2O : D_2O. NMR spectra were then obtained using this solution. After 24 h the reaction was removed from the thermoshaker and allowed to cool to room temperature. This completed the first cycle.

For the second cycle a 450 mM potassium cyanate solution was first prepared by dissolving 730.1 mg (9.0 mmol) of potassium cyanate in 20.0 mL of 9 : 1 H_2O : D_2O. The paste was then redissolved in 4.0 mL of the 450 mM potassium cyanate solution, adjusted to pH 7.3 and the same steps of leaving to react in solution for 24 h, drying to a paste for 24 h and heating to 50 °C for 24 h, as detailed above, were then repeated. After the second cycle was complete a third cycle was then performed using an identical method.

Reactions with other nucleophiles were conducted using an identical procedure unless otherwise specified.

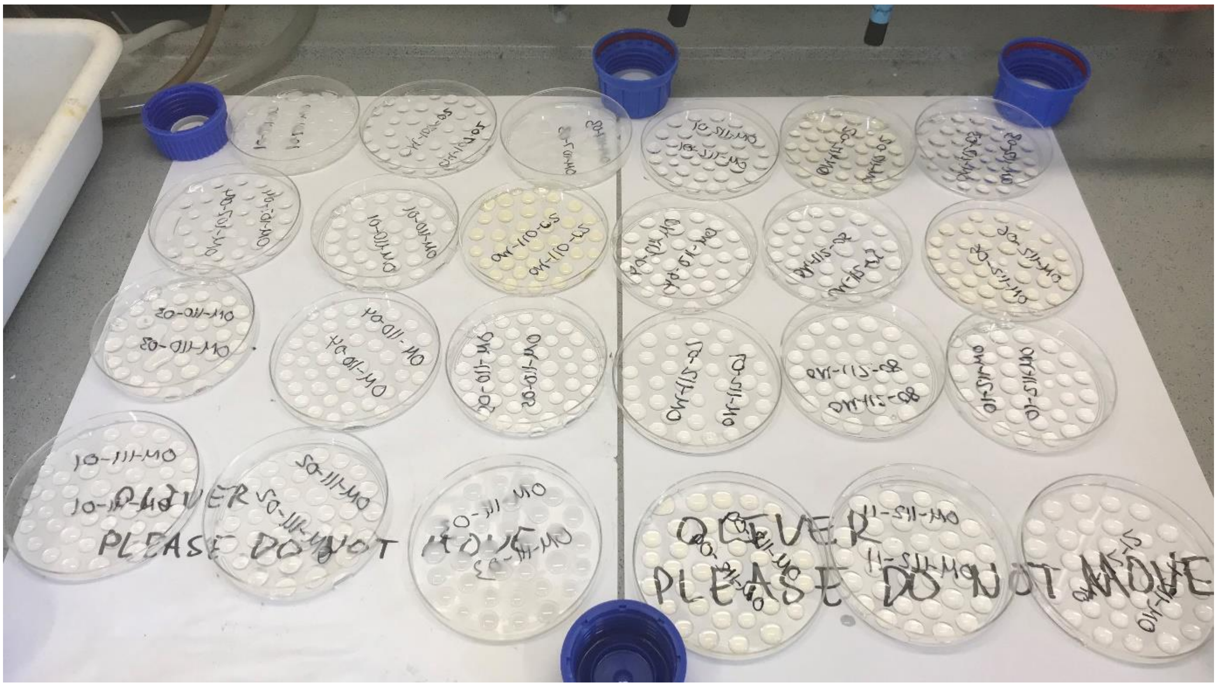

Supplementary Figure 144: Drying solutions to pastes in petri dishes.

S7.2 Phosphorylation of glycerol with orthophosphate recycling

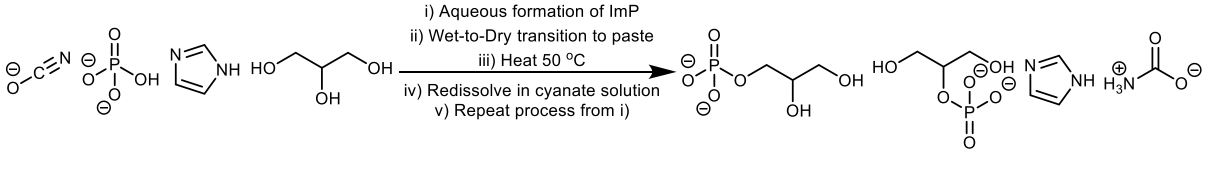

S7.2.1 Phosphorylation of glycerol with 150 mM orthophosphate

This reaction was performed according to the procedure in Section S7.1.

A total of three cycles were performed. Formation of imidazole phosphate was observed in solution and remained present after drying the solution to a paste. Formation of glycerol-1-phosphate and glycerol-2-phosphate occurred during both the drying to the paste over a period of ~24 h and during the heating of the paste to 50 °C for ~24 h (Supplementary Figure 145, 146 and 147).

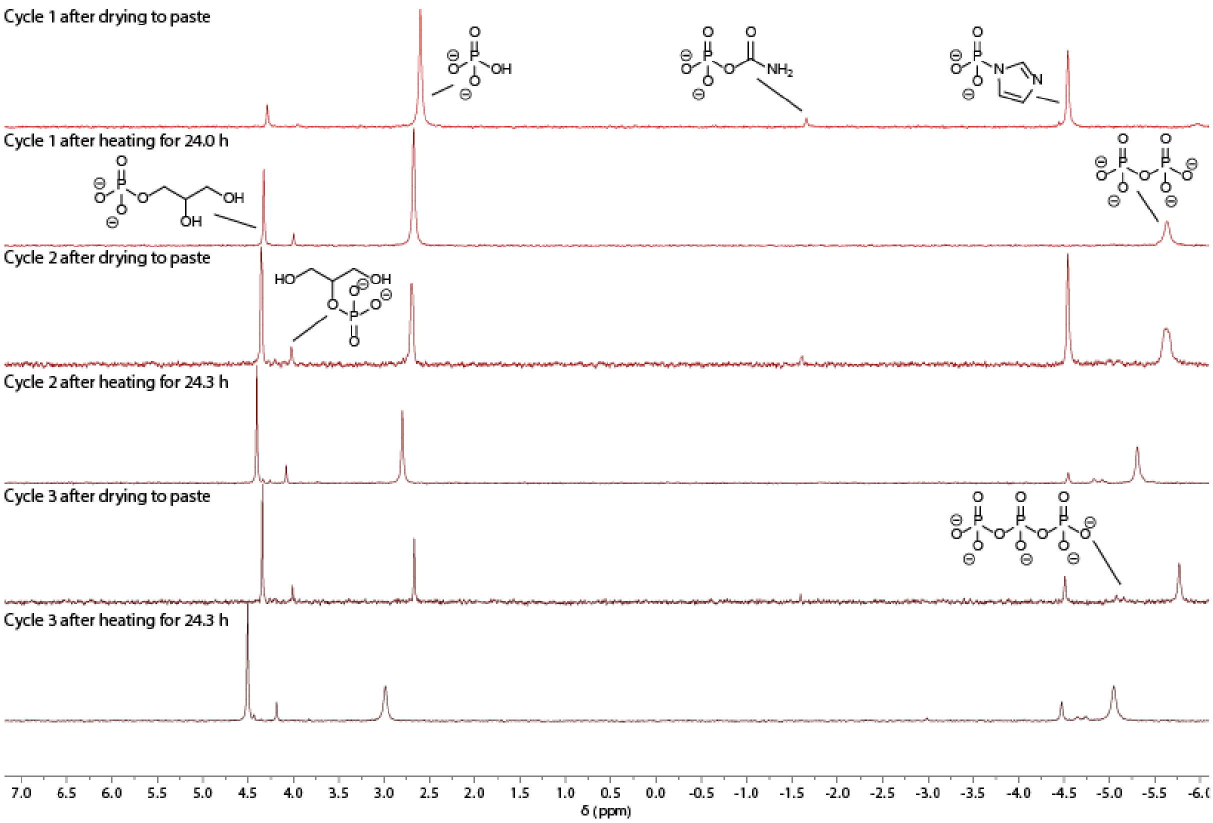

Supplementary Figure 145: Representative ^{31}P NMR spectra for the three cycles with 750 mM glycerol, 450 mM potassium cyanate and 150 mM sodium phosphate in 1.0 M imidazole buffer. For each cycle the ^{31}P NMR spectrum after the reaction had been dried to a paste and the ^{31}P NMR spectrum after heating the reaction to 50 °C for ~24 h are shown. All NMR spectra were measured in a 0.5 M citric acid buffer at pH 6.85 9 : 1 H_2O : D_2O. The chemical shift of the pyrophosphate peak is variable at pH 6.85 due to the pyrophosphate pK_a = 6.6.

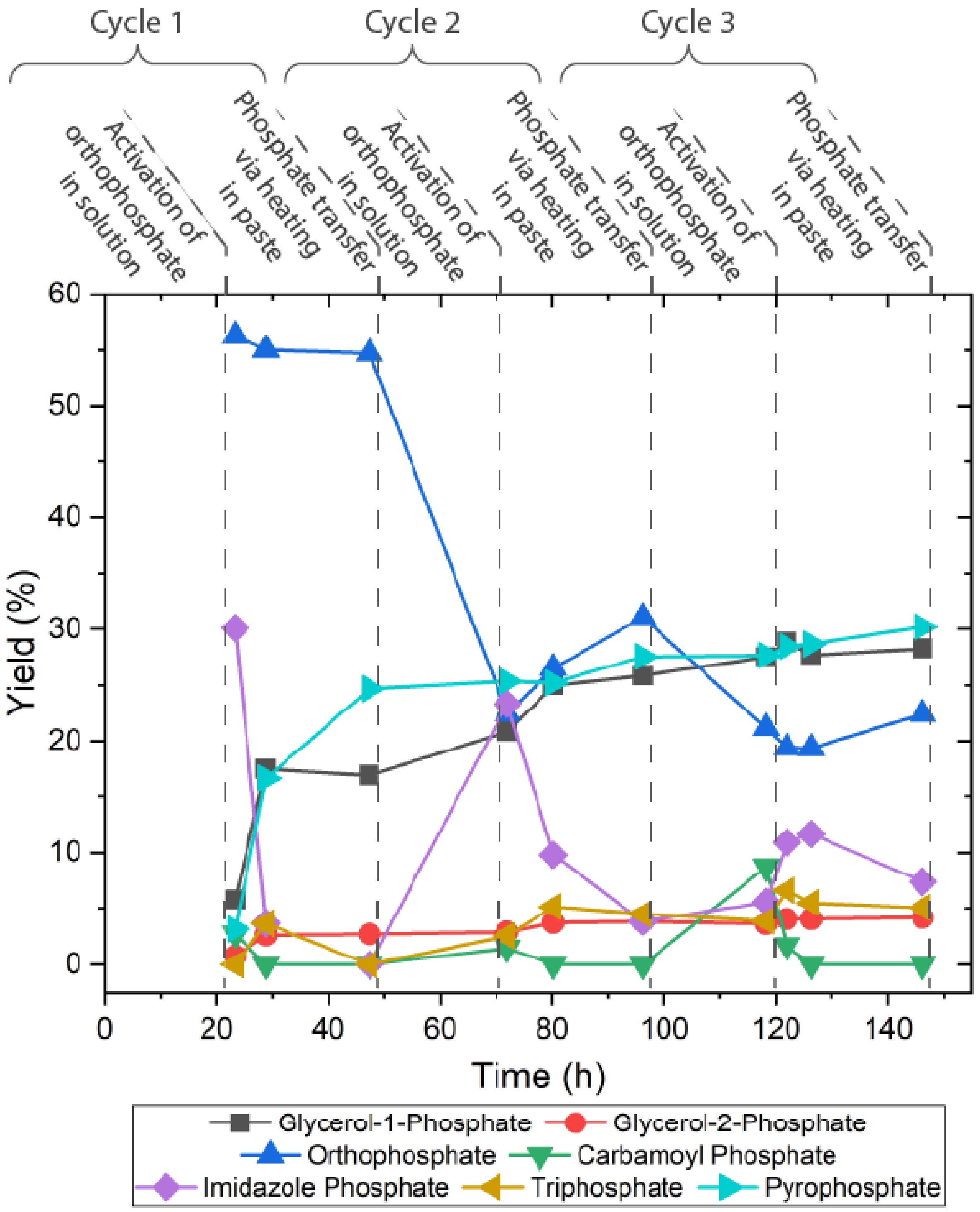

Supplementary Figure 146: Changes in yield for all phosphorous species over the course of three cycles for the reaction with 750 mM glycerol, 450 mM potassium cyanate and 150 mM sodium phosphate in 1.0 M imidazole buffer.

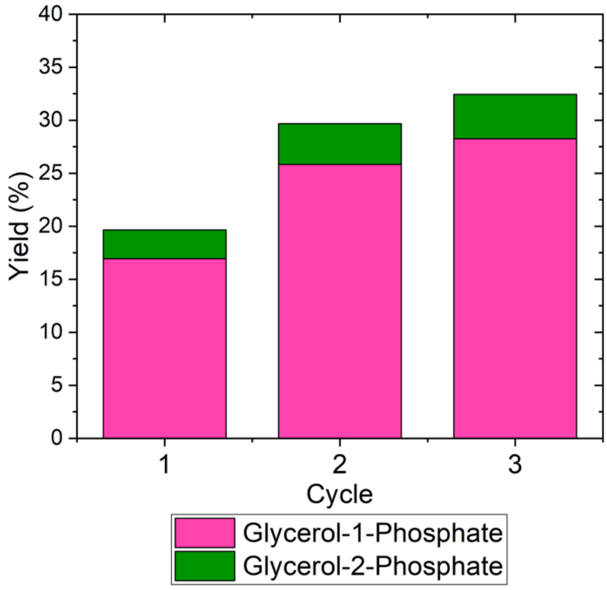

Supplementary Figure 147: Yield of glycerol-1-phosphate and glycerol-2-phosphate after each cycle for the reaction with 750 mM glycerol, 450 mM potassium cyanate and 150 mM sodium phosphate in 1.0 M imidazole buffer.

A spiking experiment with genuine samples of glycerol-1-phosphate and glycerol-2-phosphate confirmed their formation (Supplementary Figure 148).

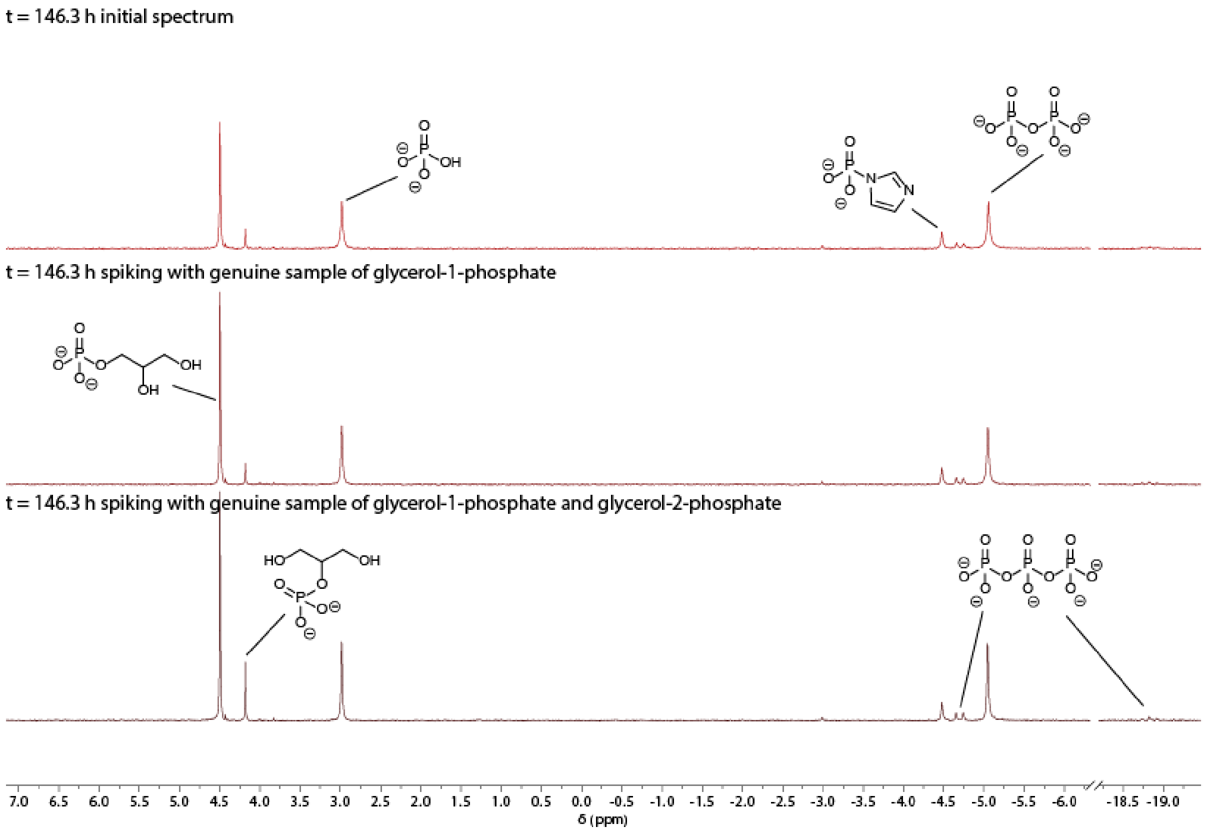

Supplementary Figure 148: ^{31}P NMR spectra of a spiking experiment for the third cycle of the reaction with 750 mM glycerol, 450 mM potassium cyanate and 150 mM sodium phosphate in 1.0 M imidazole buffer. Top spectrum is unspiked. Middle spectrum is with the addition of genuine glycerol-1-phosphate. Bottom spectrum is with the addition of genuine glycerol-2-phosphate.

S7.2.2 *Phosphorylation of glycerol with 150 mM orthophosphate repeat 1*

This reaction was performed according to the procedure in Section S7.1.

A total of three cycles were performed. Formation of imidazole phosphate was observed in solution and remained present after drying the solution to a paste. Formation of glycerol-1-phosphate and glycerol-2-phosphate occurred during both the drying to the paste over a period of ~24 h and during the heating of the paste to 50 °C for ~24 h (Supplementary Figure 149, 150 and 151).

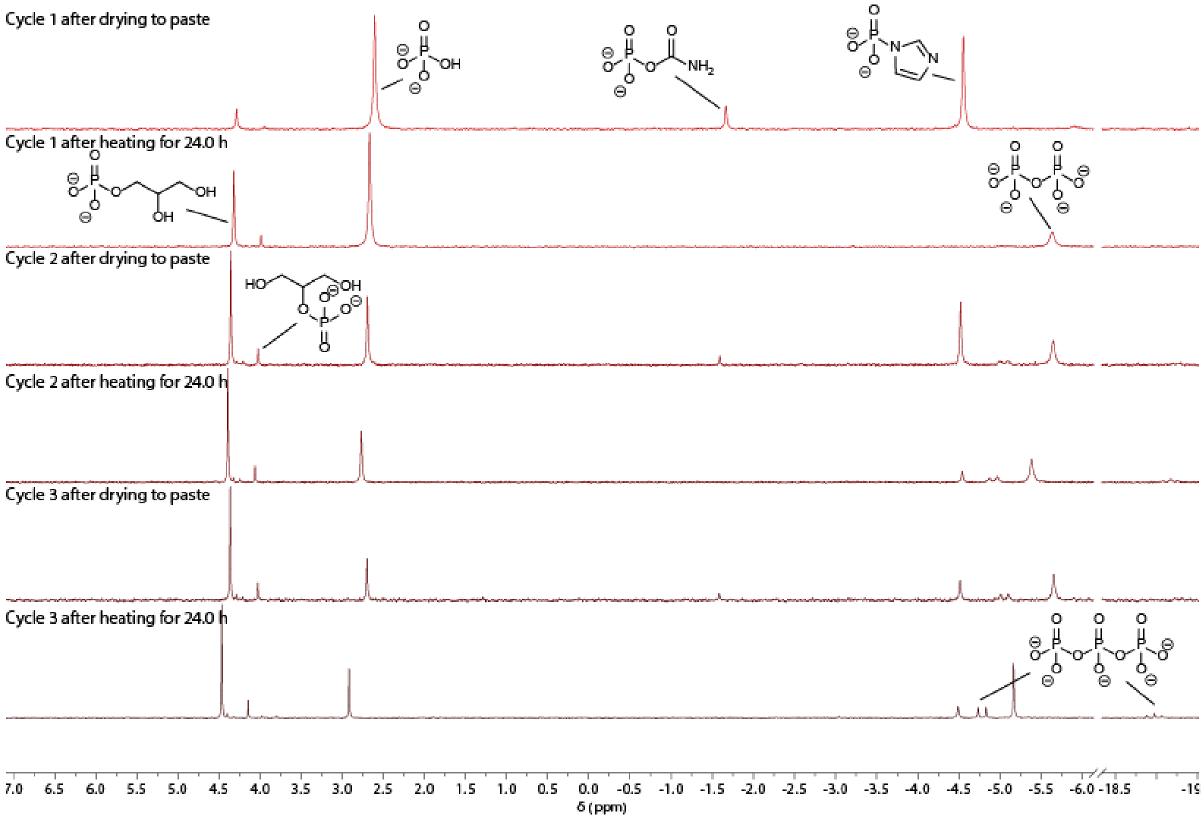

Supplementary Figure 149: Representative ^{31}P NMR spectra for the three cycles with 750 mM glycerol, 450 mM potassium cyanate and 150 mM sodium phosphate in 1.0 M imidazole buffer. For each cycle the ^{31}P NMR spectrum after the reaction had been dried to a paste and the ^{31}P NMR spectrum after heating the reaction to 50 °C for ~24 h are shown. All NMR spectra were measured in a 0.5 M citric acid buffer at pH 6.85 9 : 1 H_2O : D_2O. The chemical shift of the pyrophosphate peak is variable at pH 6.85 due to the pyrophosphate pK_a = 6.6.

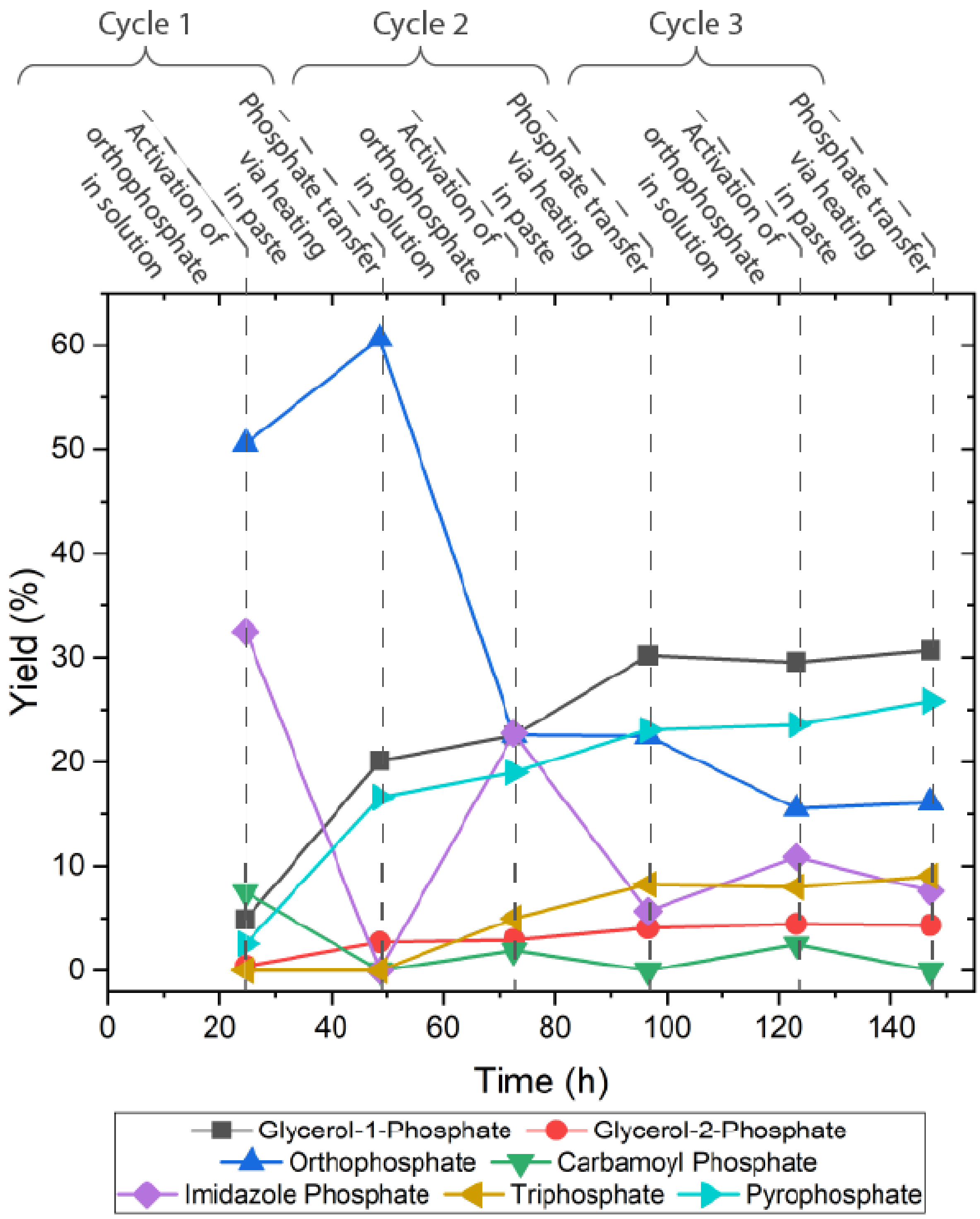

Supplementary Figure 150: Changes in yield for all phosphorous species over the course of three cycles for the reaction with 750 mM glycerol, 450 mM potassium cyanate and 150 mM sodium phosphate in 1.0 M imidazole buffer.

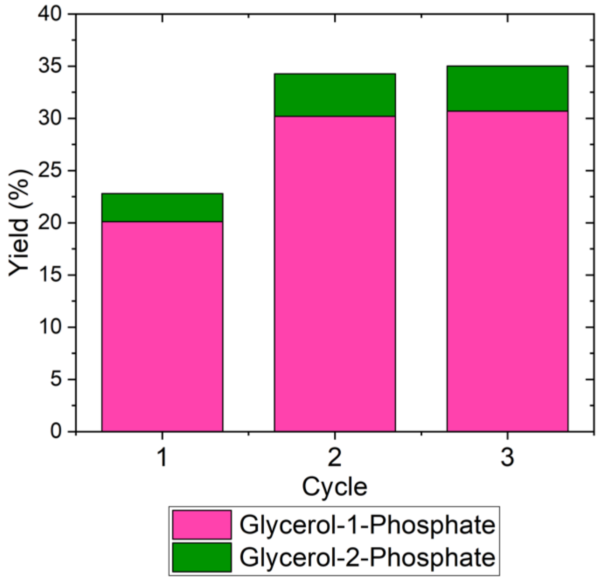

Supplementary Figure 151: Yield of glycerol-1-phosphate and glycerol-2-phosphate after each cycle for the reaction with 750 mM glycerol, 450 mM potassium cyanate and 150 mM sodium phosphate in 1.0 M imidazole buffer.

S7.2.3 Phosphorylation of glycerol with 150 mM orthophosphate repeat 2

This reaction was performed according to the procedure in Section S7.1.

A total of three cycles were performed. Formation of imidazole phosphate was observed in solution and remained present after drying the solution to a paste. Formation of glycerol-1-phosphate and glycerol-2-phosphate occurred during both the drying to the paste over a period of ~24 h and during the heating of the paste to 50 °C for ~24 h (Supplementary Figure 152, 153 and 154).

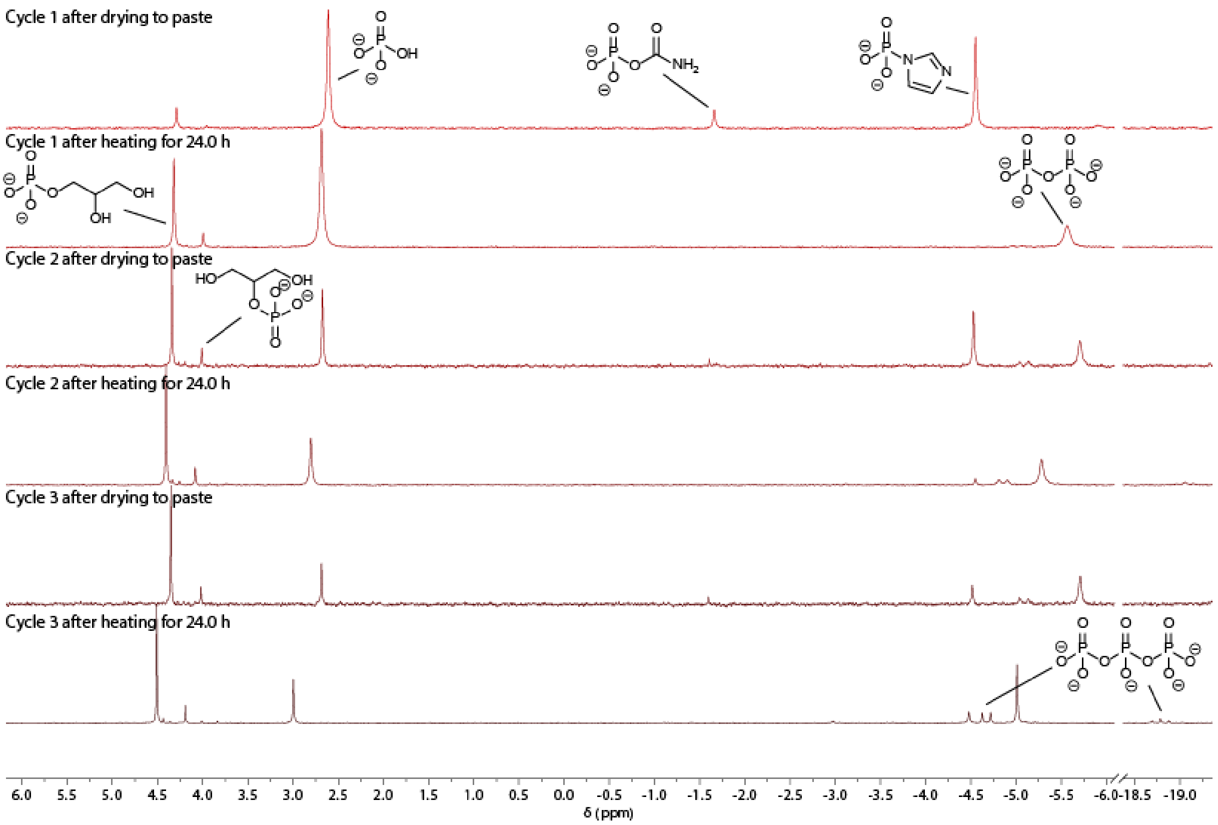

Supplementary Figure 152: Representative ^{31}P NMR spectra for the three cycles with 750 mM glycerol, 450 mM potassium cyanate and 150 mM sodium phosphate in 1.0 M imidazole buffer. For each cycle the ^{31}P NMR spectrum after the reaction had been dried to a paste and the ^{31}P NMR spectrum after heating the reaction to 50 °C for ~24 h are shown. All NMR spectra were measured in a 0.5 M citric acid buffer at pH 6.85 9 : 1 H_2O : D_2O. The chemical shift of the pyrophosphate peak is variable at pH 6.85 due to the pyrophosphate pK_a = 6.6.

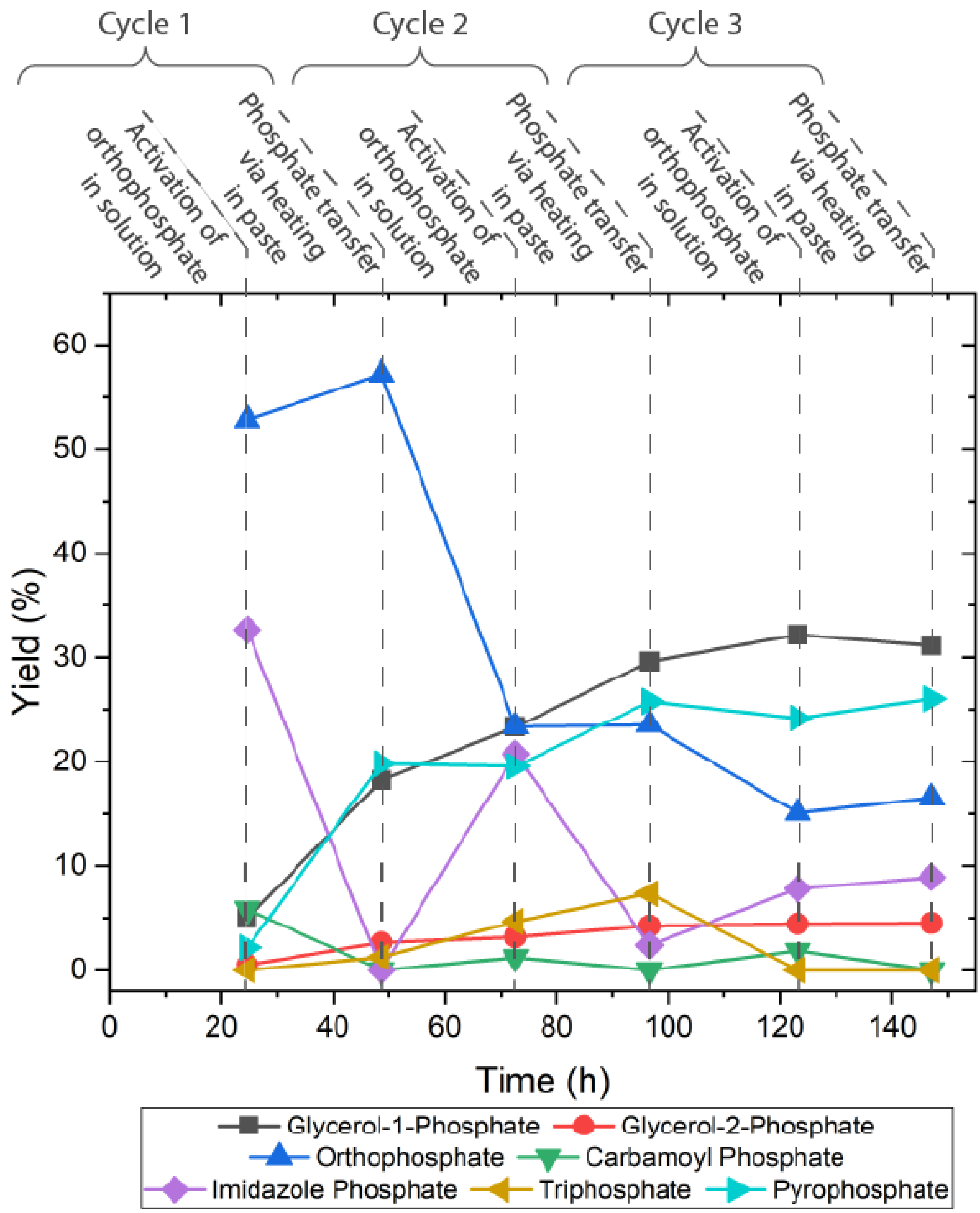

Supplementary Figure 153: Changes in yield for all phosphorous species over the course of three cycles for the reaction with 750 mM glycerol, 450 mM potassium cyanate and 150 mM sodium phosphate in 1.0 M imidazole buffer.

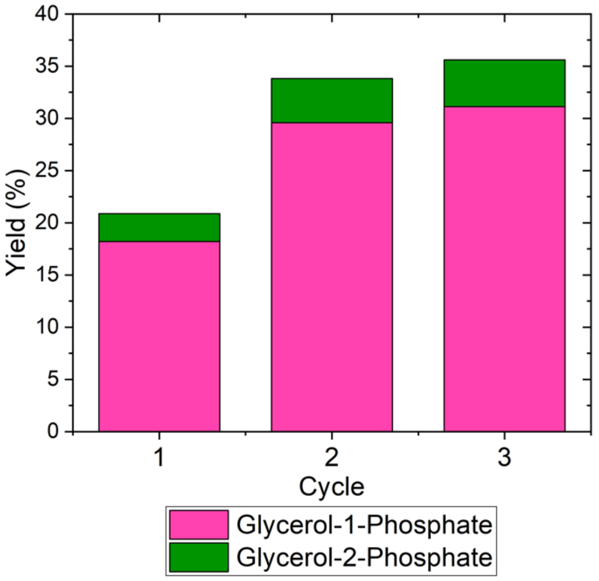

Supplementary Figure 154: Yield of glycerol-1-phosphate and glycerol-2-phosphate after each cycle for the reaction with 750 mM glycerol, 450 mM potassium cyanate and 150 mM sodium phosphate in 1.0 M imidazole buffer.

S7.2.4 Phosphorylation of glycerol with 50 mM orthophosphate

This reaction was performed according to the procedure in Section S7.1 but with 50 mM of sodium phosphate dibasic dihydrate salt (178.0 mg, 1.0 mmol).

A total of three cycles were performed. Formation of imidazole phosphate was observed in solution and remained present after drying the solution to a paste. Formation of glycerol-1-phosphate and glycerol-2-phosphate occurred during both the drying to the paste over a period of ~24 h and during the heating of the paste to 50 °C for ~24 h (Supplementary Figure 155, 156 and 157).

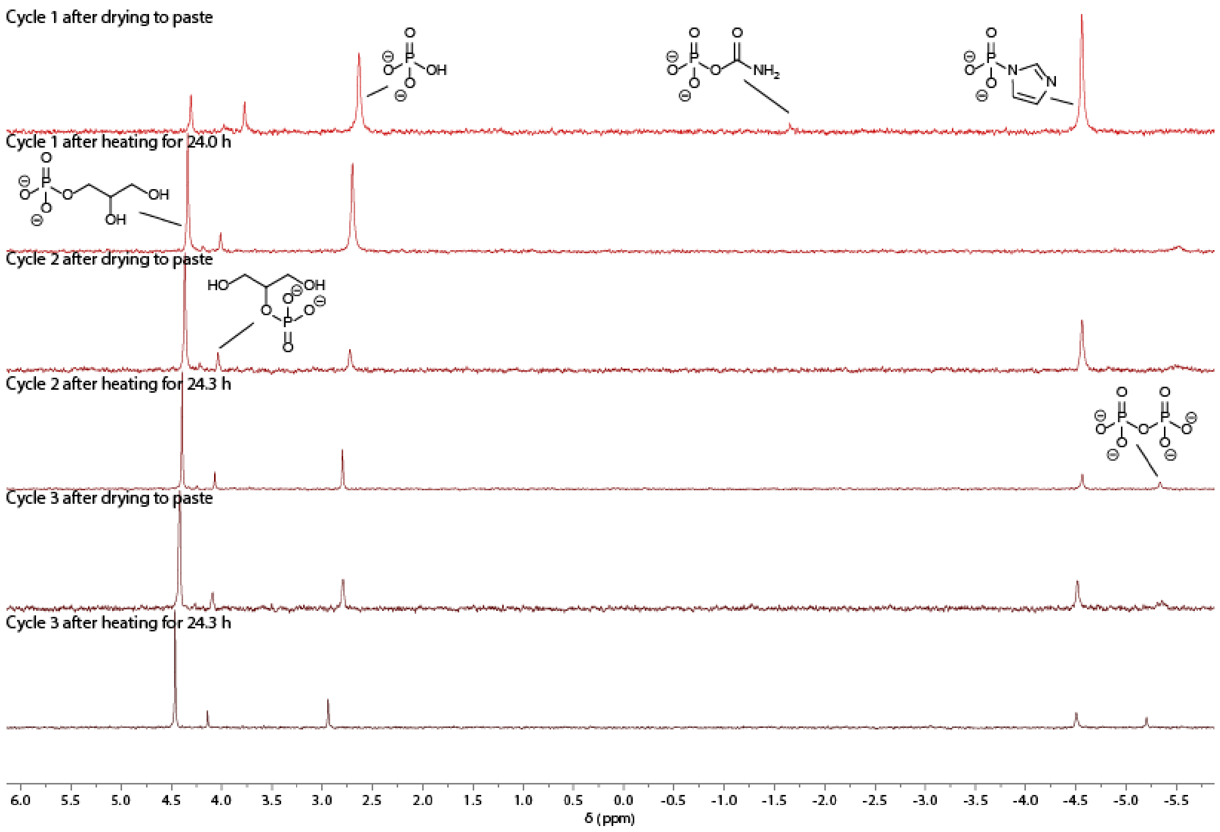

Supplementary Figure 155: Representative ^{31}P NMR spectra for the three cycles with 750 mM glycerol, 450 mM potassium cyanate and 50 mM sodium phosphate in 1.0 M imidazole buffer. For each cycle the ^{31}P NMR spectrum after the reaction had been dried to a paste and the ^{31}P NMR spectrum after heating the reaction to 50 °C for ~24 h are shown.

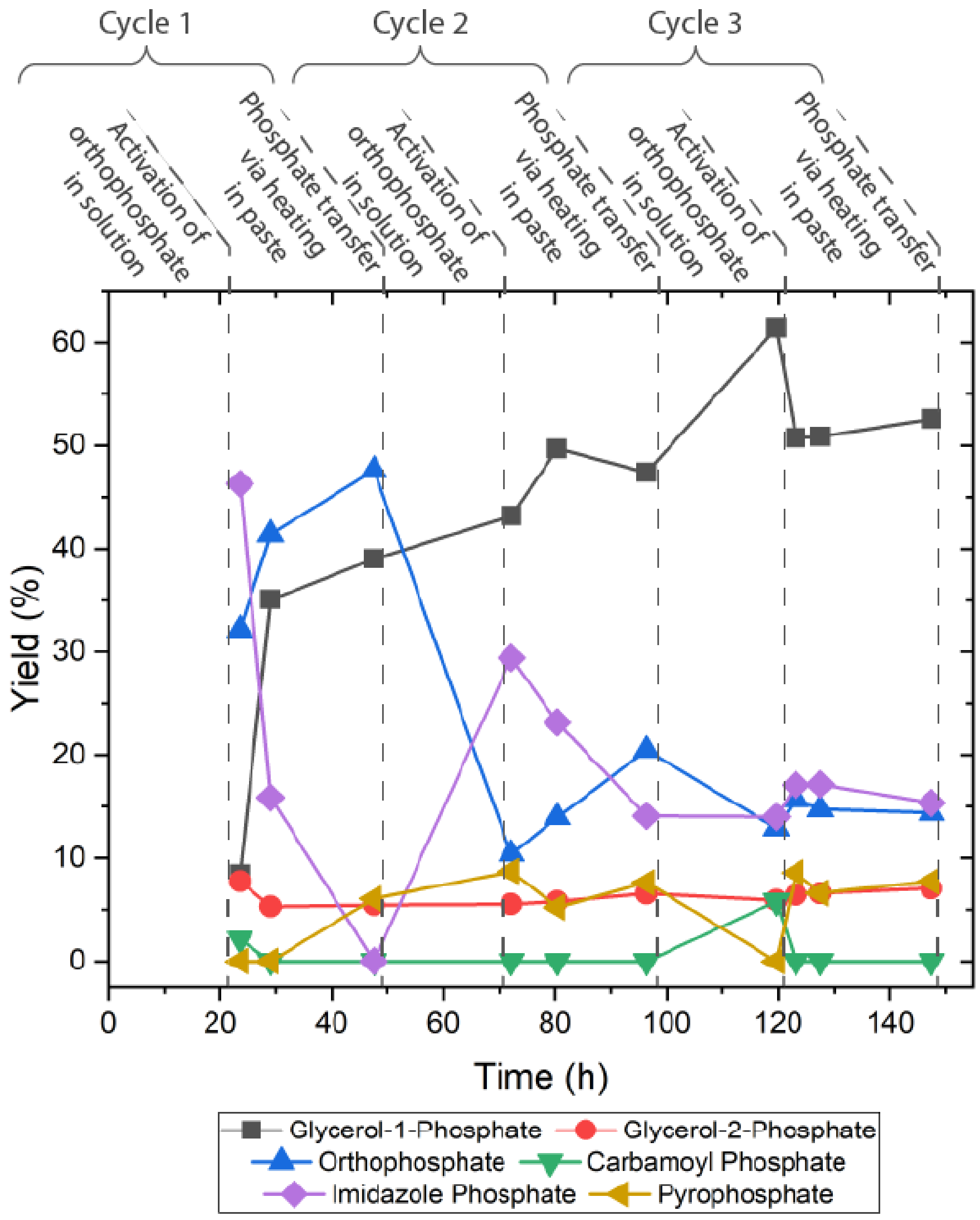

Supplementary Figure 156: Changes in yield for all phosphorous species over the course of three cycles for the reaction with 750 mM glycerol, 450 mM potassium cyanate and 50 mM sodium phosphate in 1.0 M imidazole buffer.

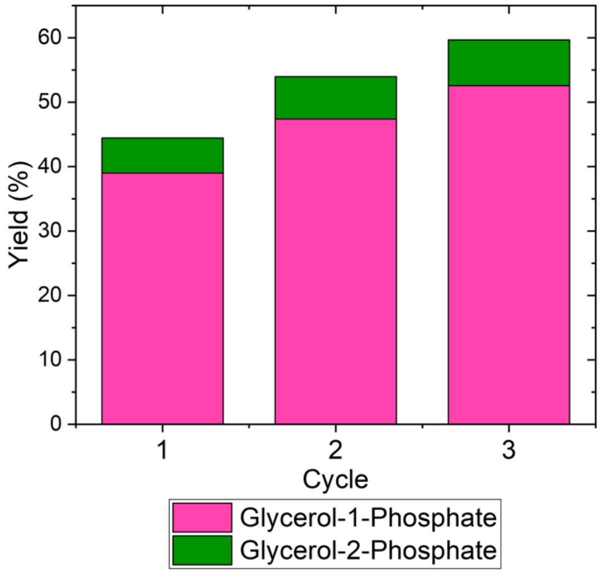

Supplementary Figure 157: Yield of glycerol-1-phosphate and glycerol-2-phosphate after each cycle for the reaction with 750 mM glycerol, 450 mM potassium cyanate and 50 mM sodium phosphate in 1.0 M imidazole buffer.

A spiking experiment with genuine samples of glycerol-1-phosphate and glycerol-2-phosphate confirmed their formation (Supplementary Figure 158).

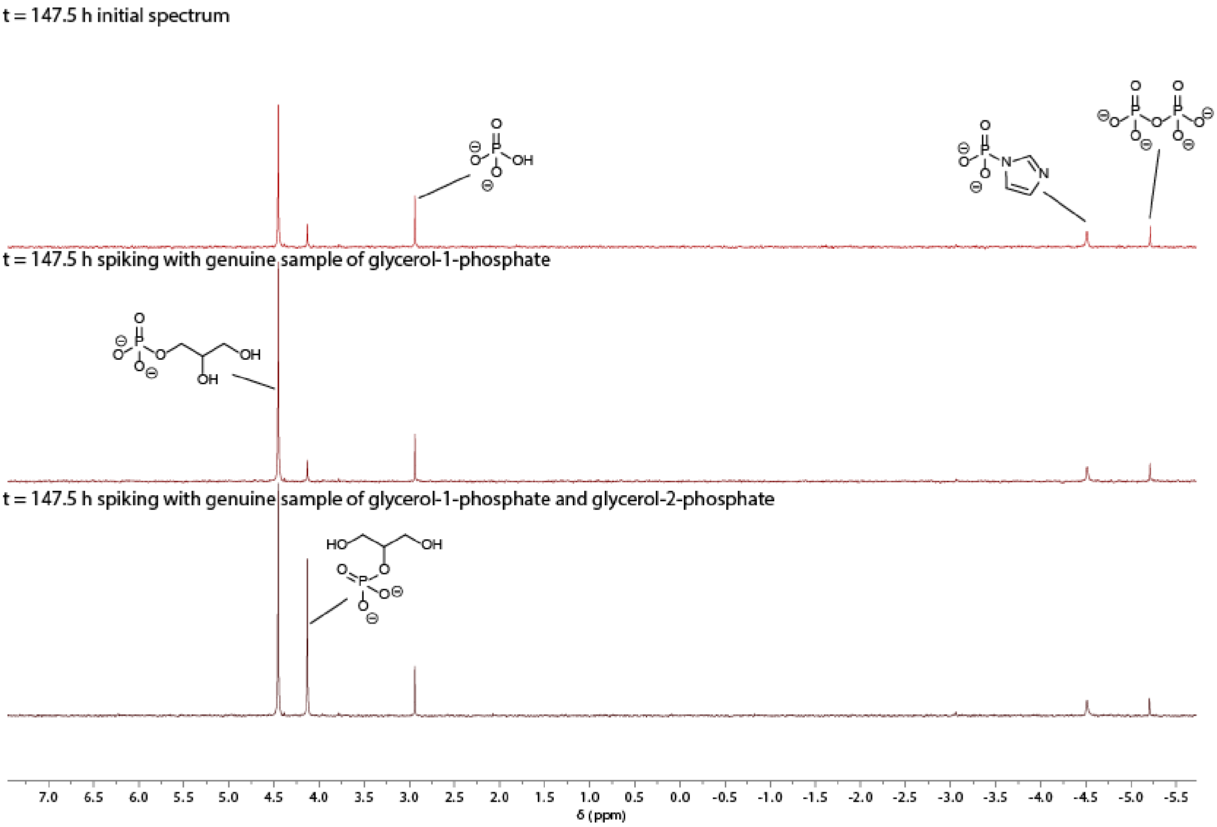

Supplementary Figure 158: ^{31}P NMR spectra of a spiking experiment for the third cycle of the reaction with 750 mM glycerol, 450 mM potassium cyanate and 50 mM sodium phosphate in 1.0 M imidazole buffer. Top spectrum is unspiked. Middle spectrum is with the addition of genuine glycerol-1-phosphate. Bottom spectrum is with the addition of genuine glycerol-2-phosphate.

S7.2.5 Phosphorylation of glycerol with 50 mM orthophosphate repeat 1

This reaction was performed according to the procedure in Section S7.1 but with 50 mM of sodium phosphate dibasic dihydrate salt (178.0 mg, 1.0 mmol).

A total of three cycles were performed. Formation of imidazole phosphate was observed in solution and remained present after drying the solution to a paste. Formation of glycerol-1-phosphate and glycerol-2-phosphate occurred during both the drying to the paste over a period of ~24 h and during the heating of the paste to 50 °C for ~24 h (Supplementary Figure 159, 160 and 161).

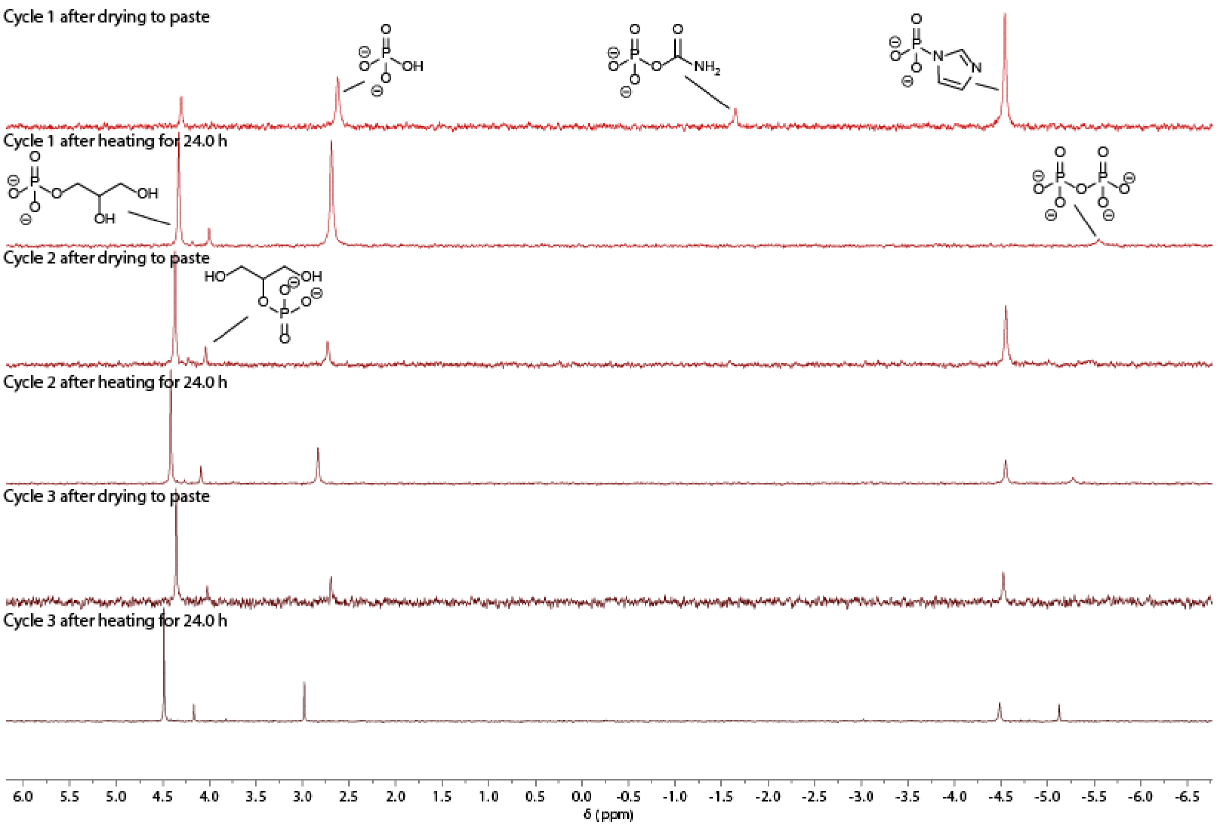

Supplementary Figure 159: Representative ^{31}P NMR spectra for the three cycles with 750 mM glycerol, 450 mM potassium cyanate and 50 mM sodium phosphate in 1.0 M imidazole buffer. For each cycle the ^{31}P NMR spectrum after the reaction had been dried to a paste and the ^{31}P NMR spectrum after heating the reaction to 50 °C for ~24 h are shown. All NMR spectra were measured in a 0.5 M citric acid buffer at pH 6.85 9 : 1 H_2O : D_2O. The chemical shift of the pyrophosphate peak is variable at pH 6.85 due to the pyrophosphate pK_a = 6.6.

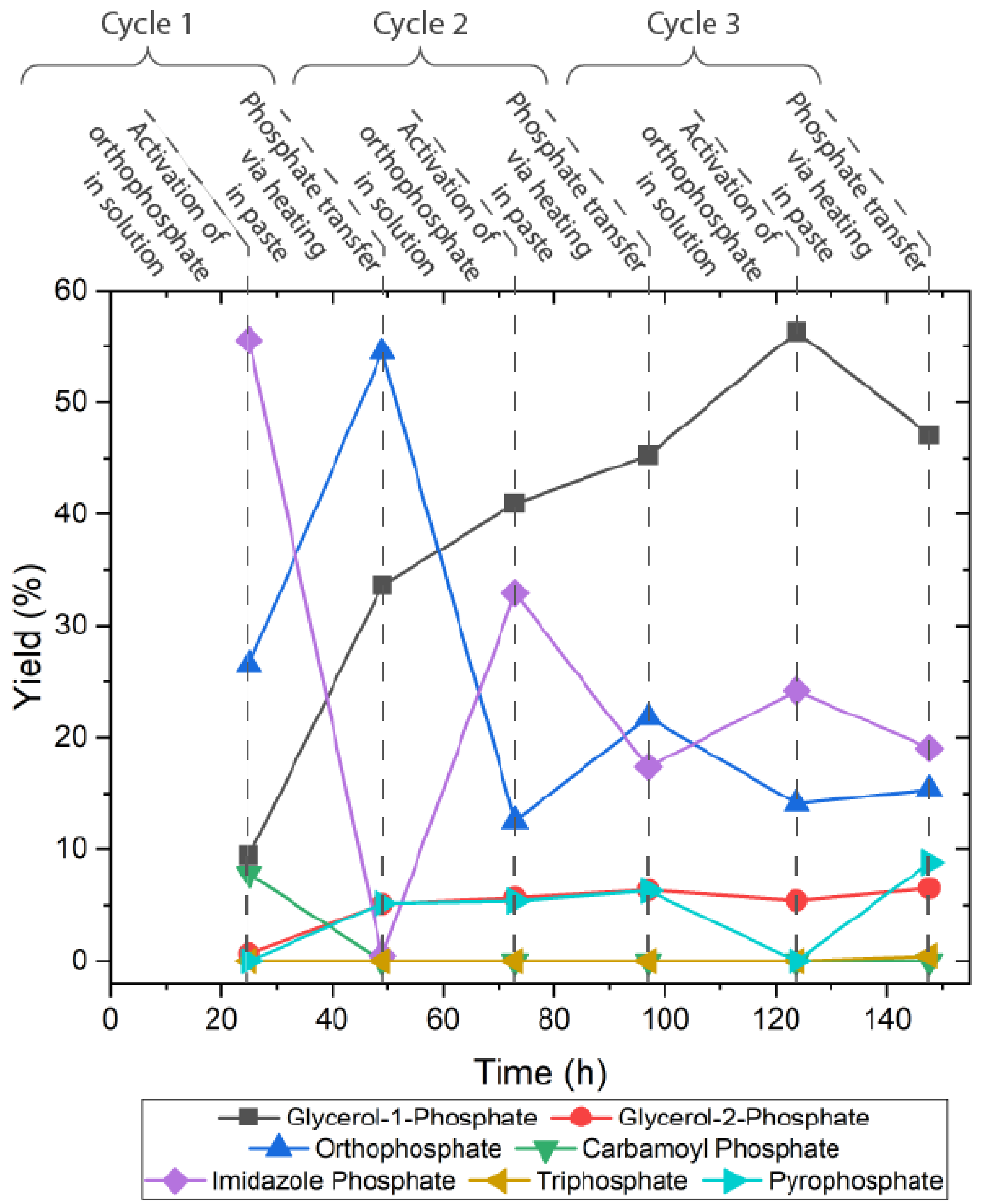

Supplementary Figure 160: Changes in yield for all phosphorous species over the course of three cycles for the reaction with 750 mM glycerol, 450 mM potassium cyanate and 50 mM sodium phosphate in 1.0 M imidazole buffer.

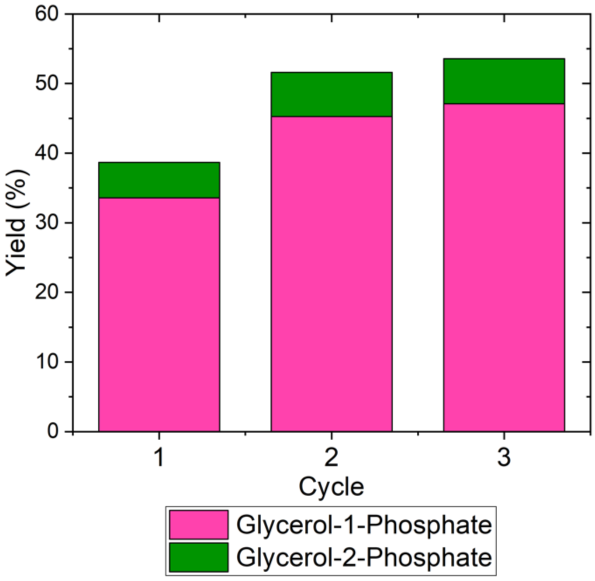

Supplementary Figure 161: Yield of glycerol-1-phosphate and glycerol-2-phosphate after each cycle for the reaction with 750 mM glycerol, 450 mM potassium cyanate and 50 mM sodium phosphate in 1.0 M imidazole buffer.

S7.2.6 Phosphorylation of glycerol with 50 mM orthophosphate repeat 2

This reaction was performed according to the procedure in Section S7.1 but with 50 mM of sodium phosphate dibasic dihydrate salt (178.0 mg, 1.0 mmol).

A total of three cycles were performed. Formation of imidazole phosphate was observed in solution and remained present after drying the solution to a paste. Formation of glycerol-1-phosphate and glycerol-2-phosphate occurred during both the drying to the paste over a period of ~24 h and during the heating of the paste to 50 °C for ~24 h (Supplementary Figure 162, 163 and 164).

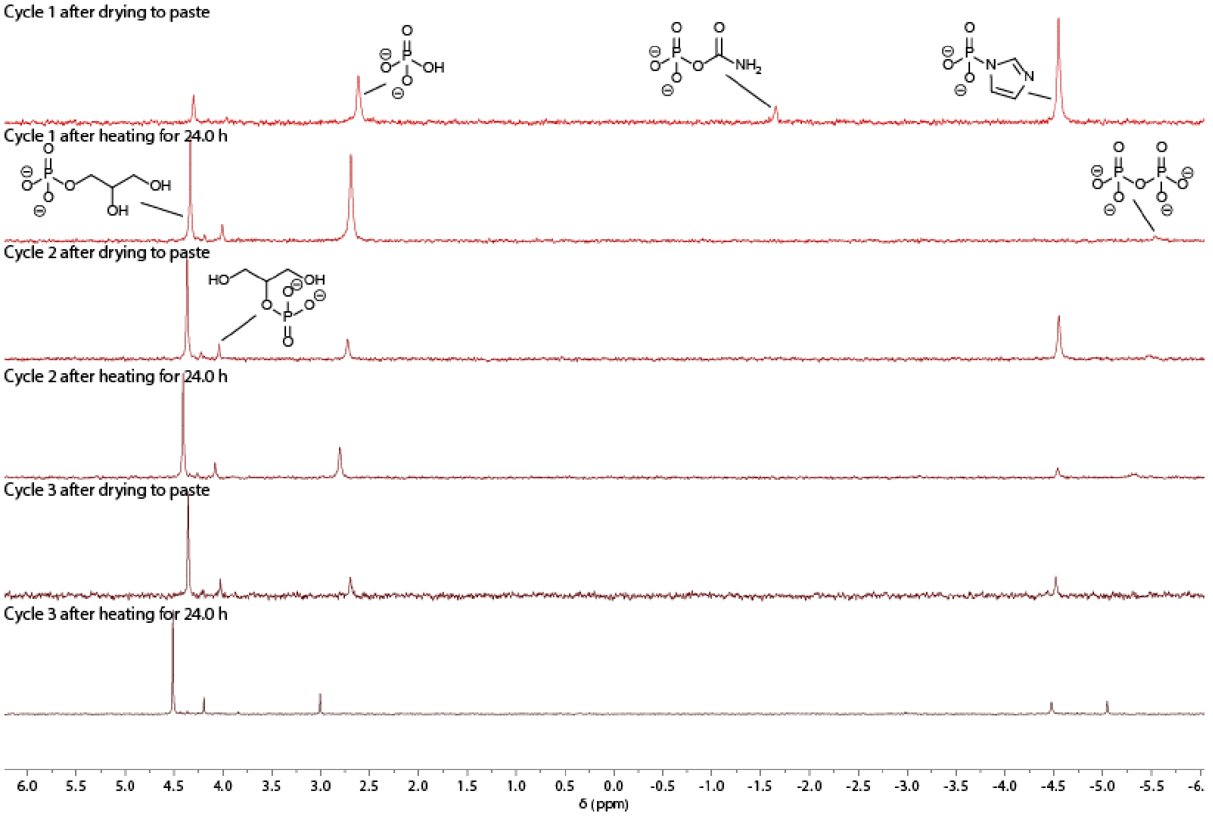

Supplementary Figure 162: Representative ^{31}P NMR spectra for the three cycles with 750 mM glycerol, 450 mM potassium cyanate and 50 mM sodium phosphate in 1.0 M imidazole buffer. For each cycle the ^{31}P NMR spectrum after the reaction had been dried to a paste and the ^{31}P NMR spectrum after heating the reaction to 50 °C for ~24 h are shown. All NMR spectra were measured in a 0.5 M citric acid buffer at pH 6.85 9 : 1 H_2O : D_2O. The chemical shift of the pyrophosphate peak is variable at pH 6.85 due to the pyrophosphate pK_a = 6.6.

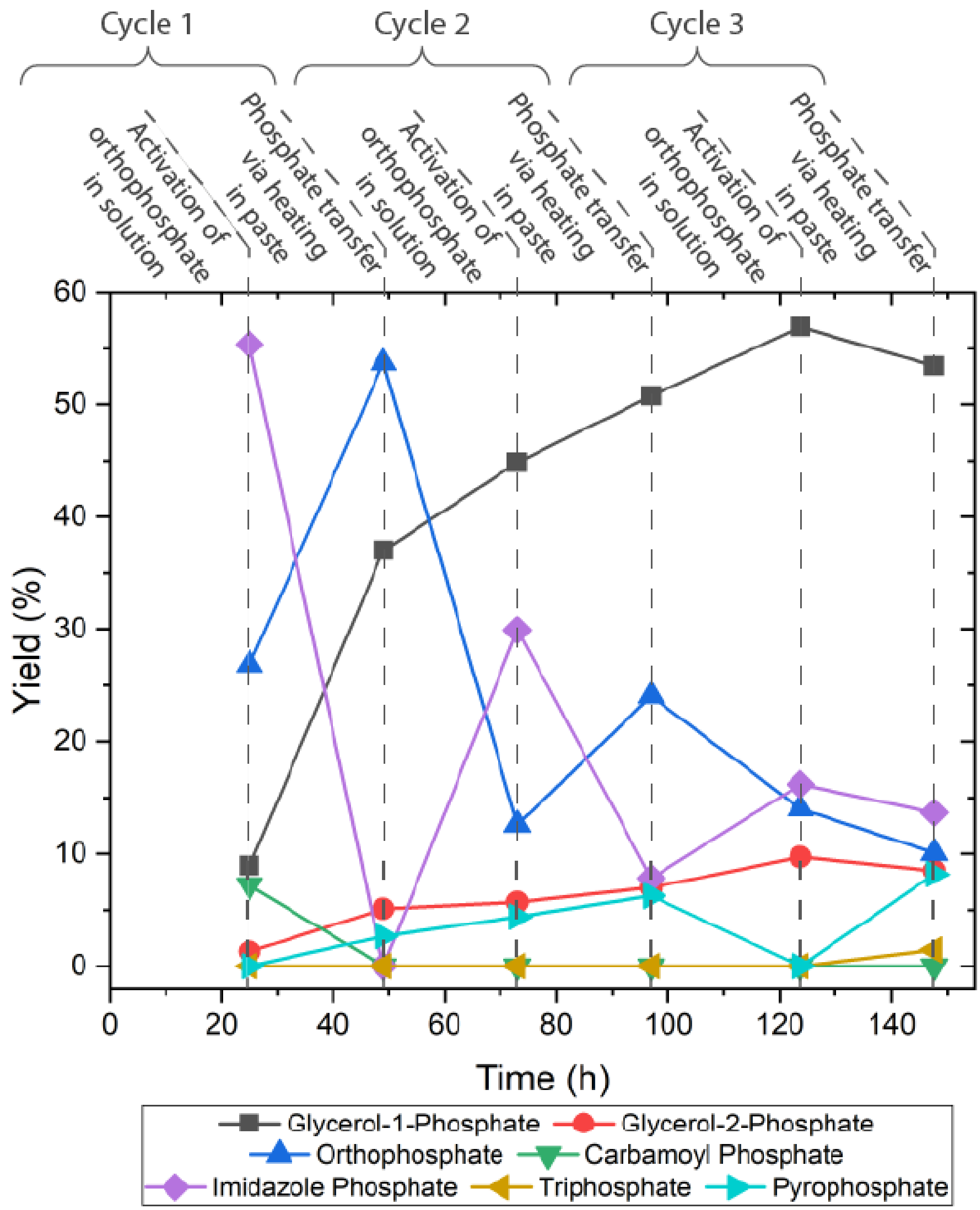

Supplementary Figure 163: Changes in yield for all phosphorous species over the course of three cycles for the reaction with 750 mM glycerol, 450 mM potassium cyanate and 50 mM sodium phosphate in 1.0 M imidazole buffer.

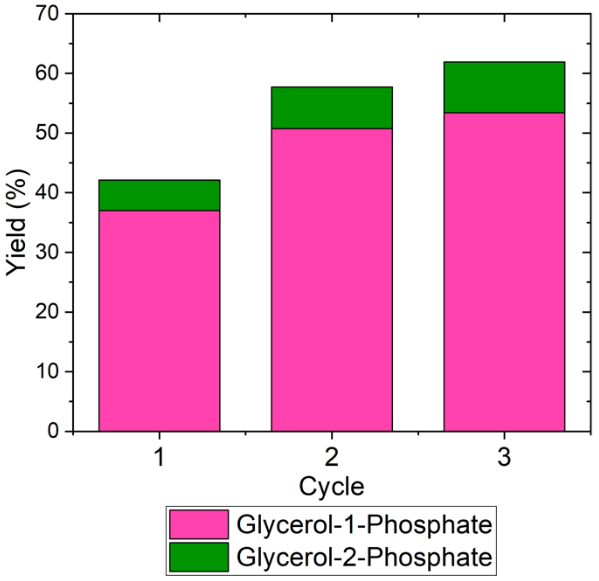

Supplementary Figure 164: Yield of glycerol-1-phosphate and glycerol-2-phosphate after each cycle for the reaction with 750 mM glycerol, 450 mM potassium cyanate and 50 mM sodium phosphate in 1.0 M imidazole buffer.

S7.2.7 Phosphorylation of glycerol with 20 mM orthophosphate

This reaction was performed according to the procedure in Section S7.1 but with 20 mM of sodium phosphate dibasic dihydrate salt (71.2 mg, 0.4 mmol).

A total of three cycles were performed. Formation of imidazole phosphate was observed in solution and remained present after drying the solution to a paste. Formation of glycerol-1-phosphate and glycerol-2-phosphate occurred during both the drying to the paste over a period of ~24 h and during the heating of the paste to 50 °C for ~24 h (Supplementary Figure 165, 166 and 167).

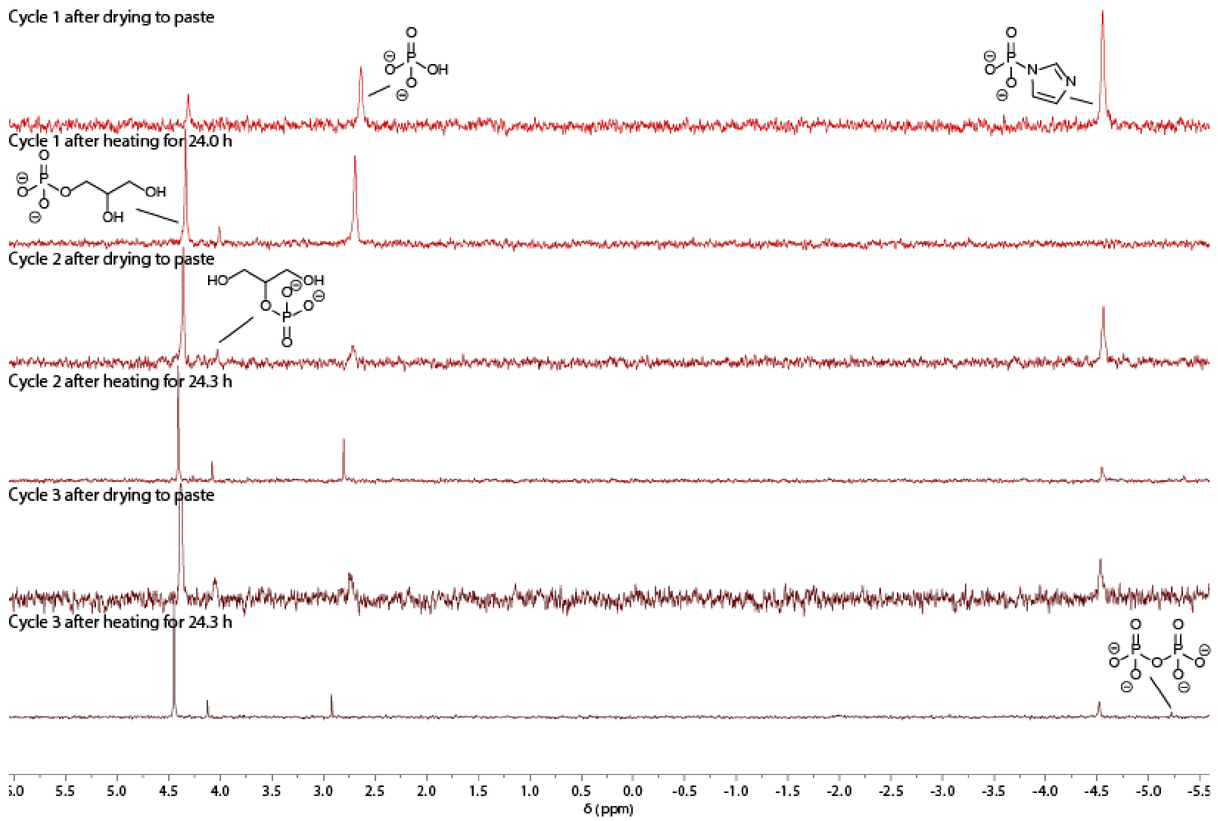

Supplementary Figure 165: Representative ^{31}P NMR spectra for the three cycles with 750 mM glycerol, 450 mM potassium cyanate and 20 mM sodium phosphate in 1.0 M imidazole buffer. For each cycle the ^{31}P NMR spectrum after the reaction had been dried to a paste and the ^{31}P NMR spectrum after heating the reaction to 50 °C for ~24 h are shown.

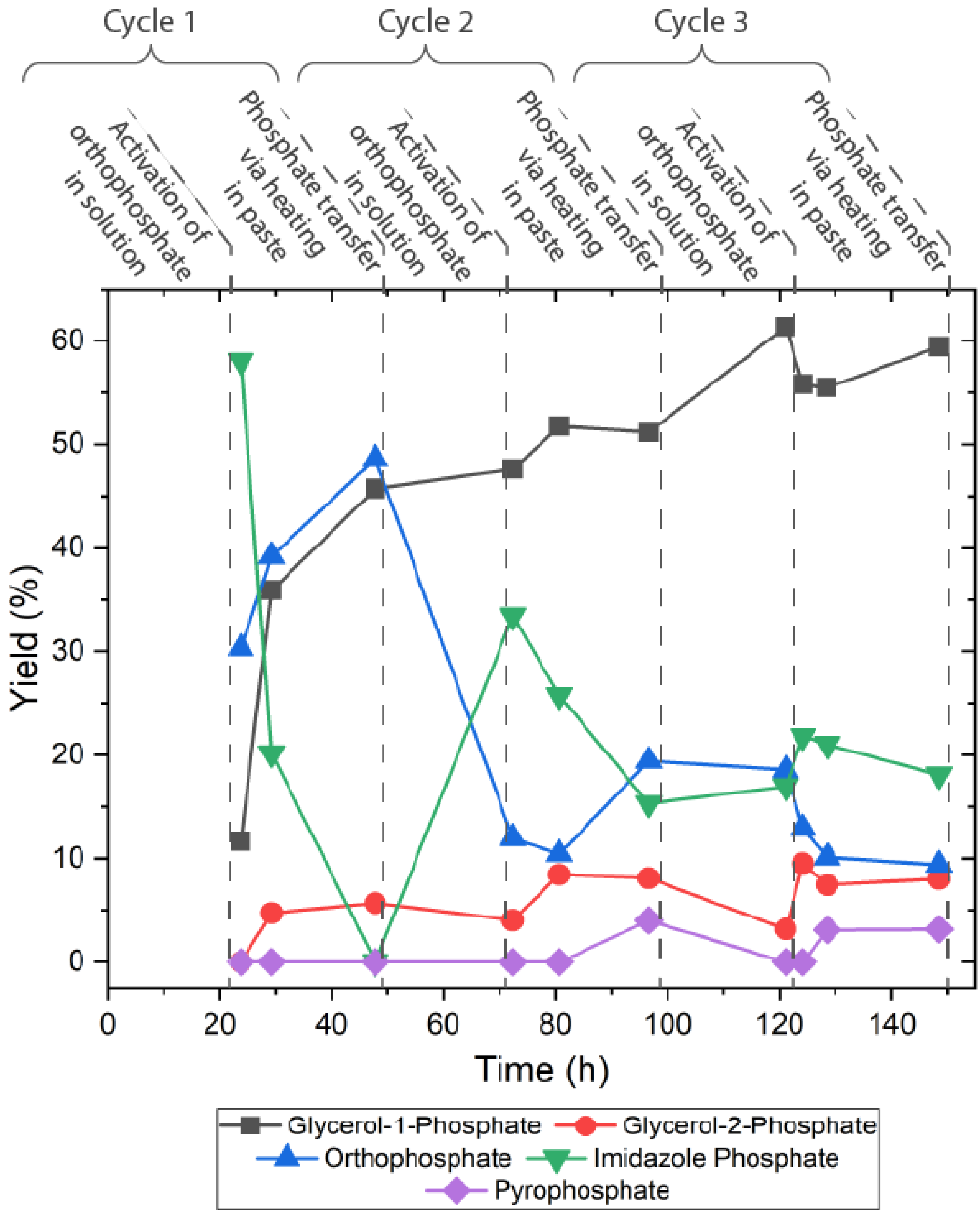

Supplementary Figure 166: Changes in yield for all phosphorous species over the course of three cycles for the reaction with 750 mM glycerol, 450 mM potassium cyanate and 20 mM sodium phosphate in 1.0 M imidazole buffer.

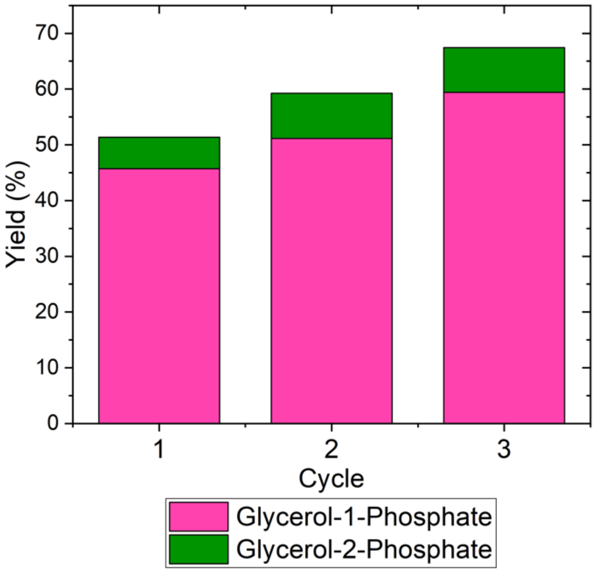

Supplementary Figure 167: Yield of glycerol-1-phosphate and glycerol-2-phosphate after each cycle for the reaction with 750 mM glycerol, 450 mM potassium cyanate and 20 mM sodium phosphate in 1.0 M imidazole buffer.

A spiking experiment with genuine samples of glycerol-1-phosphate and glycerol-2-phosphate confirmed their formation (Supplementary Figure 168).

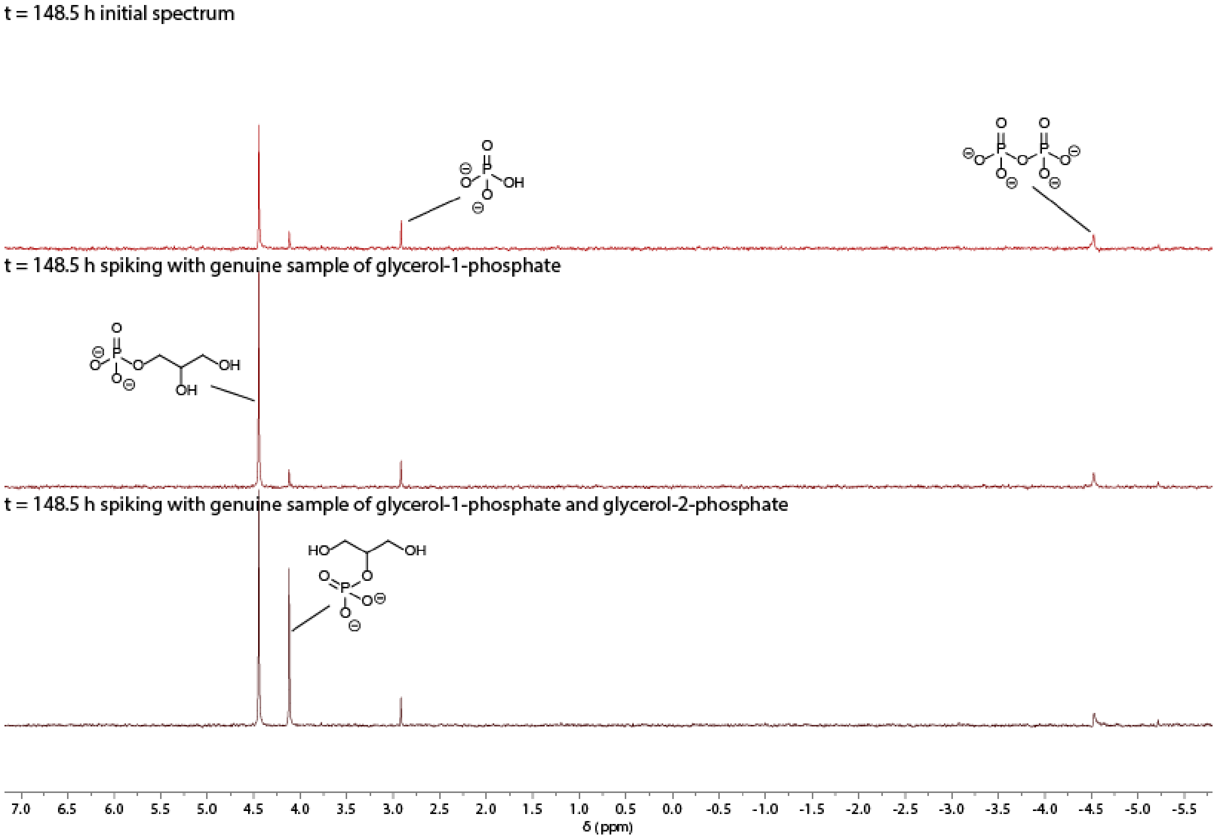

Supplementary Figure 168: ^{31}P NMR spectra of a spiking experiment for the third cycle of the reaction with 750 mM glycerol, 450 mM potassium cyanate and 20 mM sodium phosphate in 1.0 M imidazole buffer. Top spectrum is unspiked. Middle spectrum is with the addition of genuine glycerol-1-phosphate. Bottom spectrum is with the addition of genuine glycerol-2-phosphate.

S7.2.8 Phosphorylation of glycerol with 20 mM orthophosphate repeat 1

This reaction was performed according to the procedure in Section S7.1 but with 20 mM of sodium phosphate dibasic dihydrate salt (71.2 mg, 0.4 mmol).

A total of three cycles were performed. Formation of imidazole phosphate was observed in solution and remained present after drying the solution to a paste. Formation of glycerol-1-phosphate and glycerol-2-phosphate occurred during both the drying to the paste over a period of ~24 h and during the heating of the paste to 50 °C for ~24 h (Supplementary Figure 169, 170 and 171).

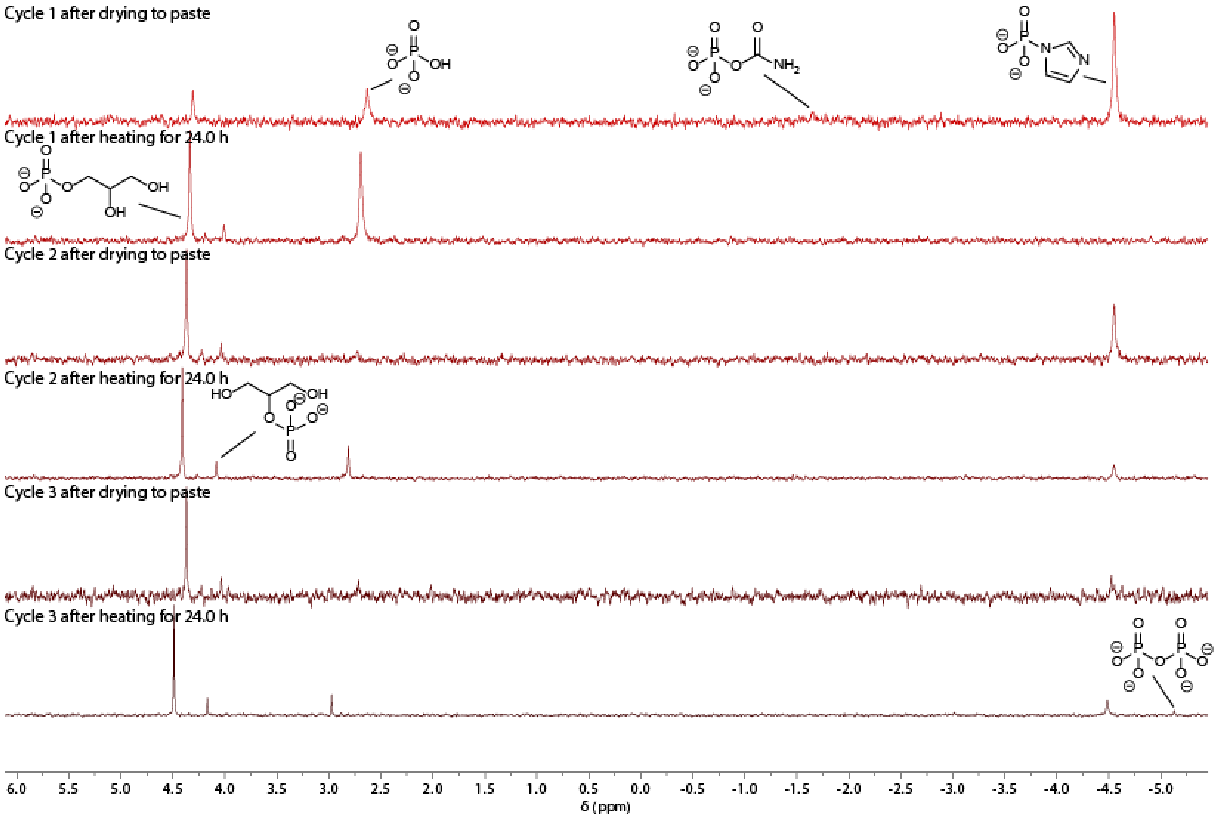

Supplementary Figure 169: Representative ^{31}P NMR spectra for the three cycles with 750 mM glycerol, 450 mM potassium cyanate and 20 mM sodium phosphate in 1.0 M imidazole buffer. For each cycle the ^{31}P NMR spectrum after the reaction had been dried to a paste and the ^{31}P NMR spectrum after heating the reaction to 50 °C for ~24 h are shown. All NMR spectra were measured in a 0.5 M citric acid buffer at pH 6.85 9 : 1 H_2O : D_2O. The chemical shift of the pyrophosphate peak is variable at pH 6.85 due to the pyrophosphate pK_a = 6.6.

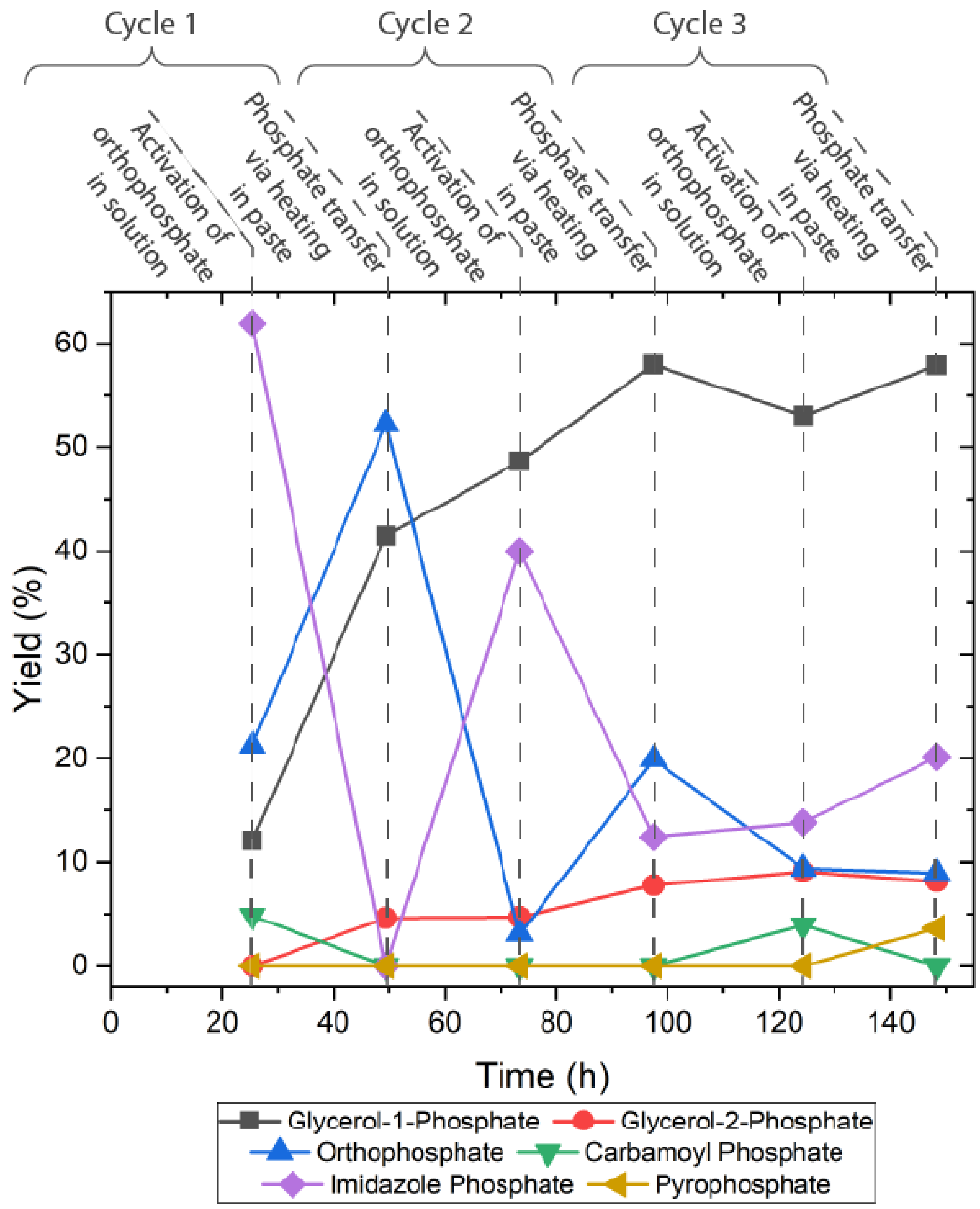

Supplementary Figure 170: Changes in yield for all phosphorous species over the course of three cycles for the reaction with 750 mM glycerol, 450 mM potassium cyanate and 20 mM sodium phosphate in 1.0 M imidazole buffer.

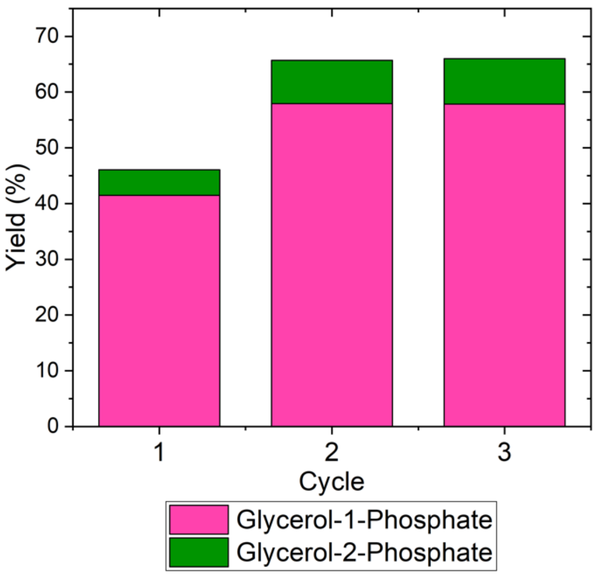

Supplementary Figure 171: Yield of glycerol-1-phosphate and glycerol-2-phosphate after each cycle for the reaction with 750 mM glycerol, 450 mM potassium cyanate and 20 mM sodium phosphate in 1.0 M imidazole buffer.

S7.2.9 Phosphorylation of glycerol with 20 mM orthophosphate repeat 2

This reaction was performed according to the procedure in Section S7.1 but with 20 mM of sodium phosphate dibasic dihydrate salt (71.2 mg, 0.4 mmol).

A total of three cycles were performed. Formation of imidazole phosphate was observed in solution and remained present after drying the solution to a paste. Formation of glycerol-1-phosphate and glycerol-2-phosphate occurred during both the drying to the paste over a period of ~24 h and during the heating of the paste to 50 °C for ~24 h (Supplementary Figure 172, 173 and 174).

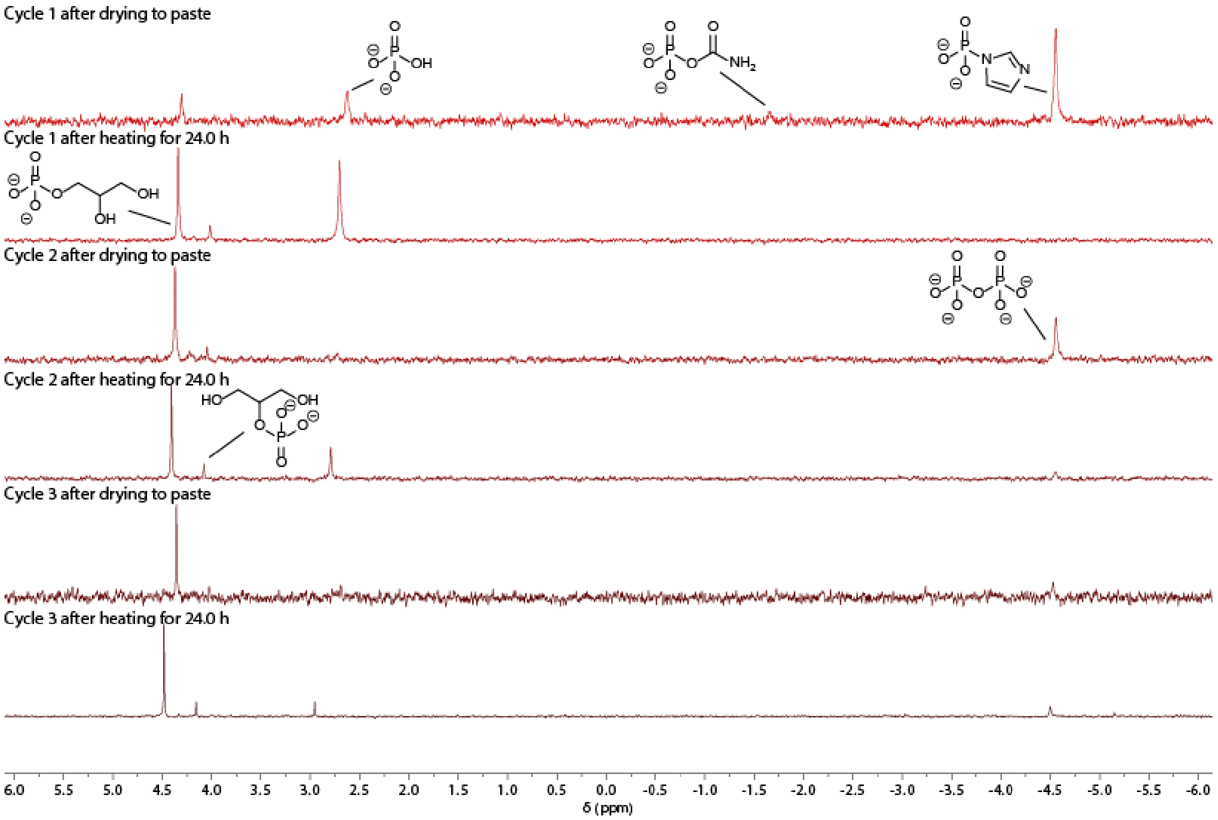

Supplementary Figure 172: Representative ^{31}P NMR spectra for the three cycles with 750 mM glycerol, 450 mM potassium cyanate and 20 mM sodium phosphate in 1.0 M imidazole buffer. For each cycle the ^{31}P NMR spectrum after the reaction had been dried to a paste and the ^{31}P NMR spectrum after heating the reaction to 50 °C for ~24 h are shown. All NMR spectra were measured in a 0.5 M citric acid buffer at pH 6.85 9 : 1 H_2O : D_2O. The chemical shift of the pyrophosphate peak is variable at pH 6.85 due to the pyrophosphate pK_a = 6.6.

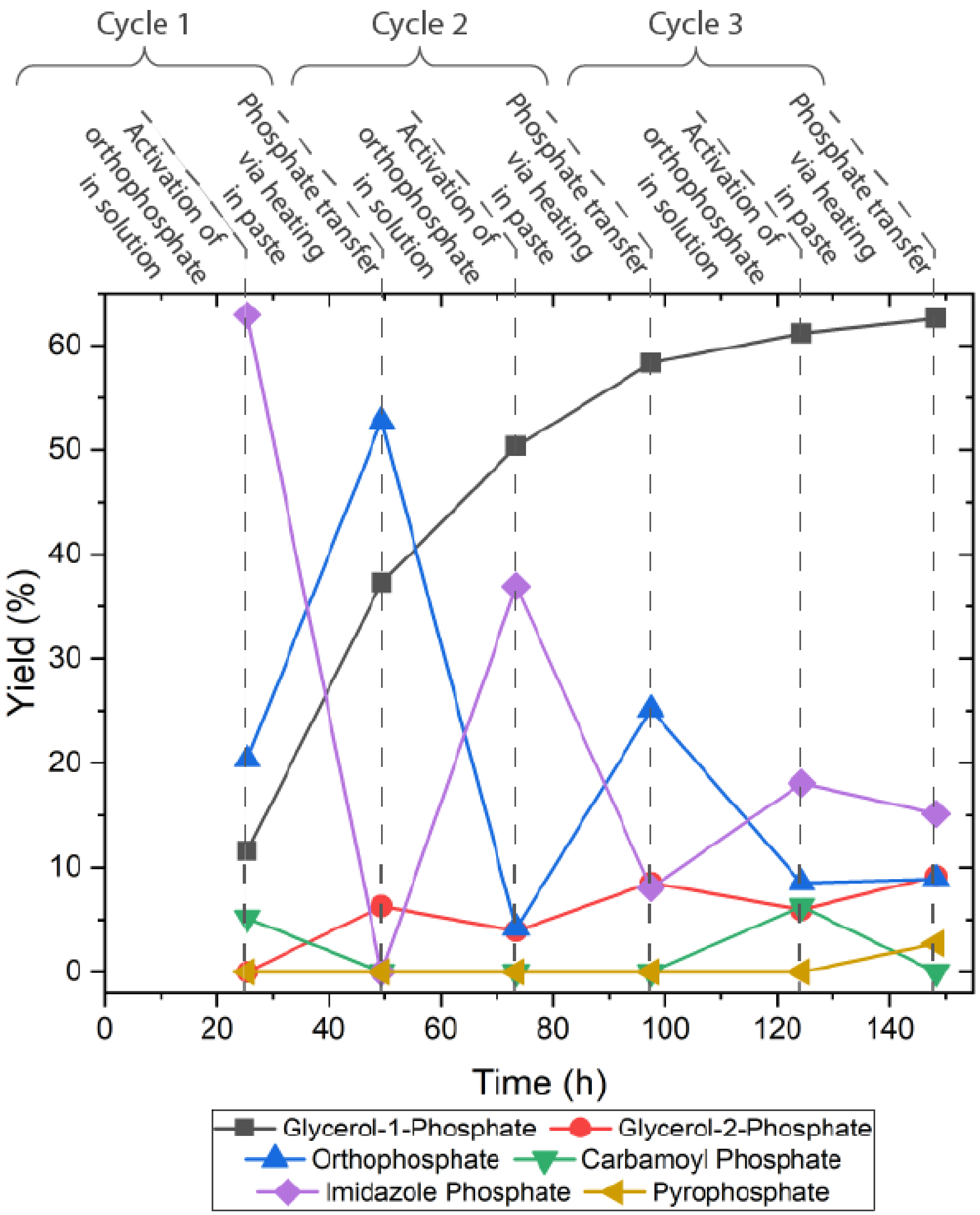

Supplementary Figure 173: Changes in yield for all phosphorous species over the course of three cycles for the reaction with 750 mM glycerol, 450 mM potassium cyanate and 20 mM sodium phosphate in 1.0 M imidazole buffer.

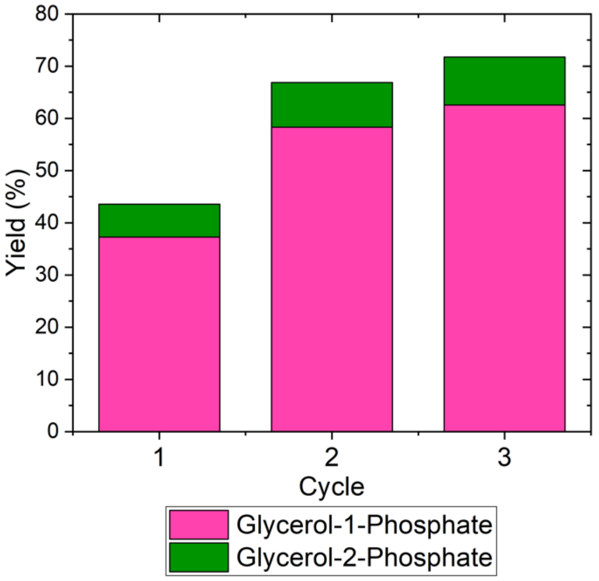

Supplementary Figure 174: Yield of glycerol-1-phosphate and glycerol-2-phosphate after each cycle for the reaction with 750 mM glycerol, 450 mM potassium cyanate and 20 mM sodium phosphate in 1.0 M imidazole buffer.

S7.3 Phosphorylation of glycerate with orthophosphate recycling

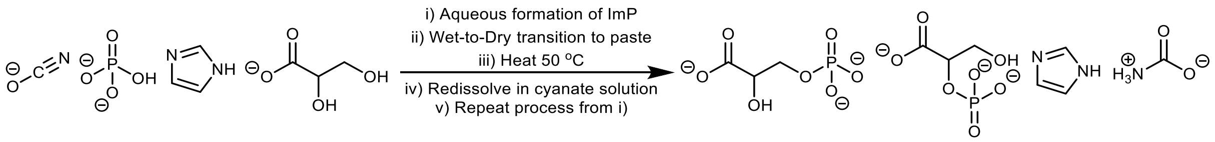

S7.3.1 Phosphorylation of glycerate with 150 mM orthophosphate

This reaction was performed according to the procedure in Section S7.1 but with 750 mM of sodium glycerate (318.2 mg, 3.0 mmol).

A total of three cycles were performed. Formation of imidazole phosphate was observed in solution and remained present after drying the solution to a paste. Formation of glycerate-2-phosphate and glycerate-3-phosphate occurred during both the drying to the paste over a period of ~24 h and during the heating of the paste to 50 °C for ~24 h (Supplementary Figure 175, 176 and 177).

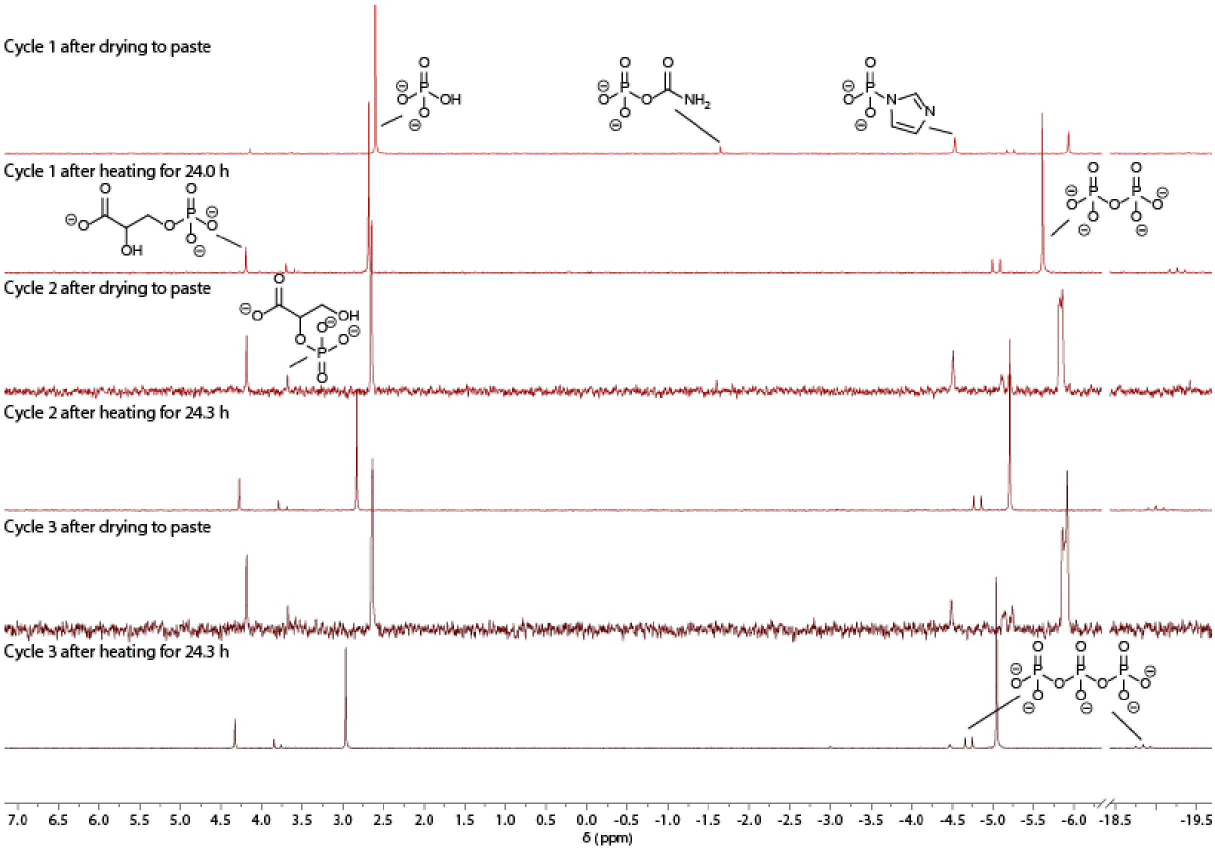

Supplementary Figure 175: Representative ^{31}P NMR spectra for the three cycles with 750 mM sodium glycerate, 450 mM potassium cyanate and 150 mM sodium phosphate in 1.0 M imidazole buffer. For each cycle the ^{31}P NMR spectrum after the reaction had been dried to a paste and the ^{31}P NMR spectrum after heating the reaction to 50 °C for ~24 h are shown. All NMR spectra were measured in a 0.5 M citric acid buffer at pH 6.85 9 : 1 H_2O : D_2O. The chemical shift of the pyrophosphate peak is variable at pH 6.85 due to the pyrophosphate pK_a = 6.6.

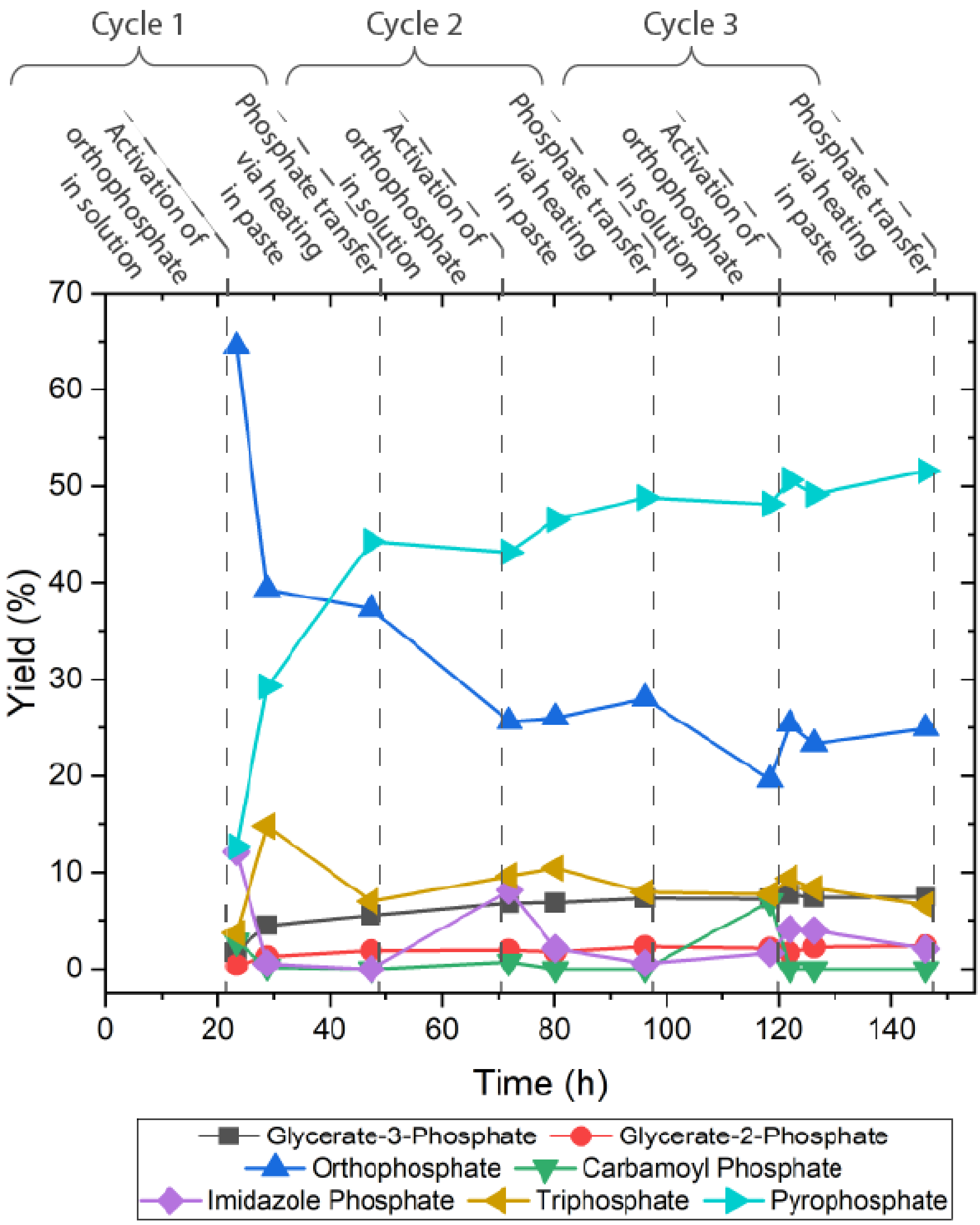

Supplementary Figure 176: Changes in yield for all phosphorous species over the course of three cycles for the reaction with 750 mM sodium glycerate, 450 mM potassium cyanate and 150 mM sodium phosphate in 1.0 M imidazole buffer.

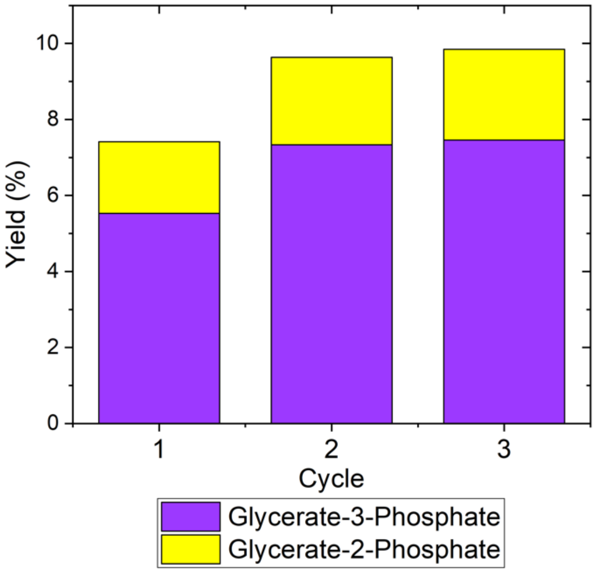

Supplementary Figure 177: Yield of glycerate-2-phosphate and glycerate-3-phosphate after each cycle for the reaction with 750 mM sodium glycerate, 450 mM potassium cyanate and 150 mM sodium phosphate in 1.0 M imidazole buffer.

A spiking experiment with genuine samples of glycerate-2-phosphate and glycerate-3-phosphate confirmed their formation (Supplementary Figure 178).

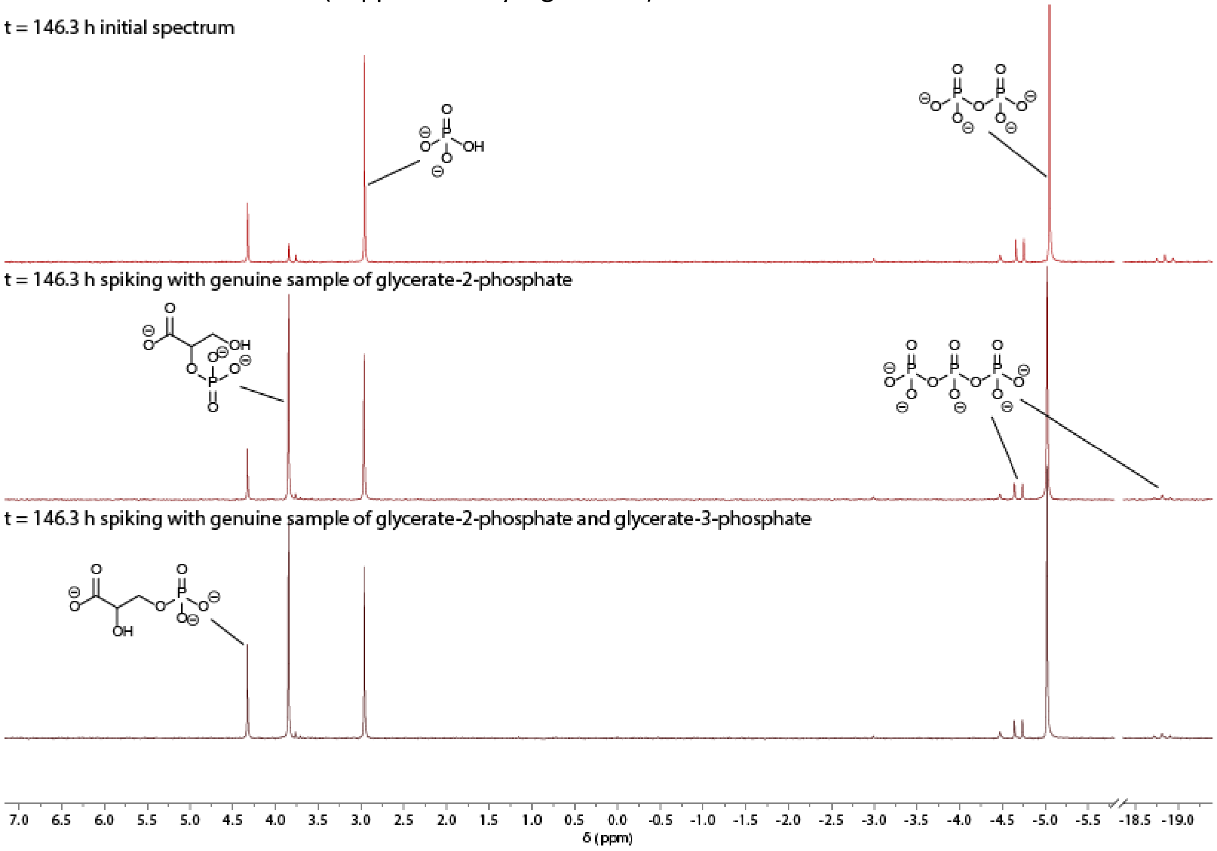

Supplementary Figure 178: ^{31}P NMR spectra of a spiking experiment for the third cycle of the reaction with 750 mM sodium glycerate, 450 mM potassium cyanate and 150 mM sodium phosphate in 1.0 M imidazole buffer. Top spectrum is unspiked. Middle spectrum is with the addition of genuine glycerate-2-phosphate. Bottom spectrum is with the addition of genuine glycerate-3-phosphate.

S7.3.2 Phosphorylation of glycerate with 150 mM orthophosphate repeat 1

This reaction was performed according to the procedure in Section S7.1 but with 750 mM of sodium glycerate (318.2 mg, 3.0 mmol).

A total of three cycles were performed. Formation of imidazole phosphate was observed in solution and remained present after drying the solution to a paste. Formation of glycerate-2-phosphate and glycerate-3-phosphate occurred during both the drying to the paste over a period of ~24 h and during the heating of the paste to 50 °C for ~24 h (Supplementary Figure 179, 180 and 181).

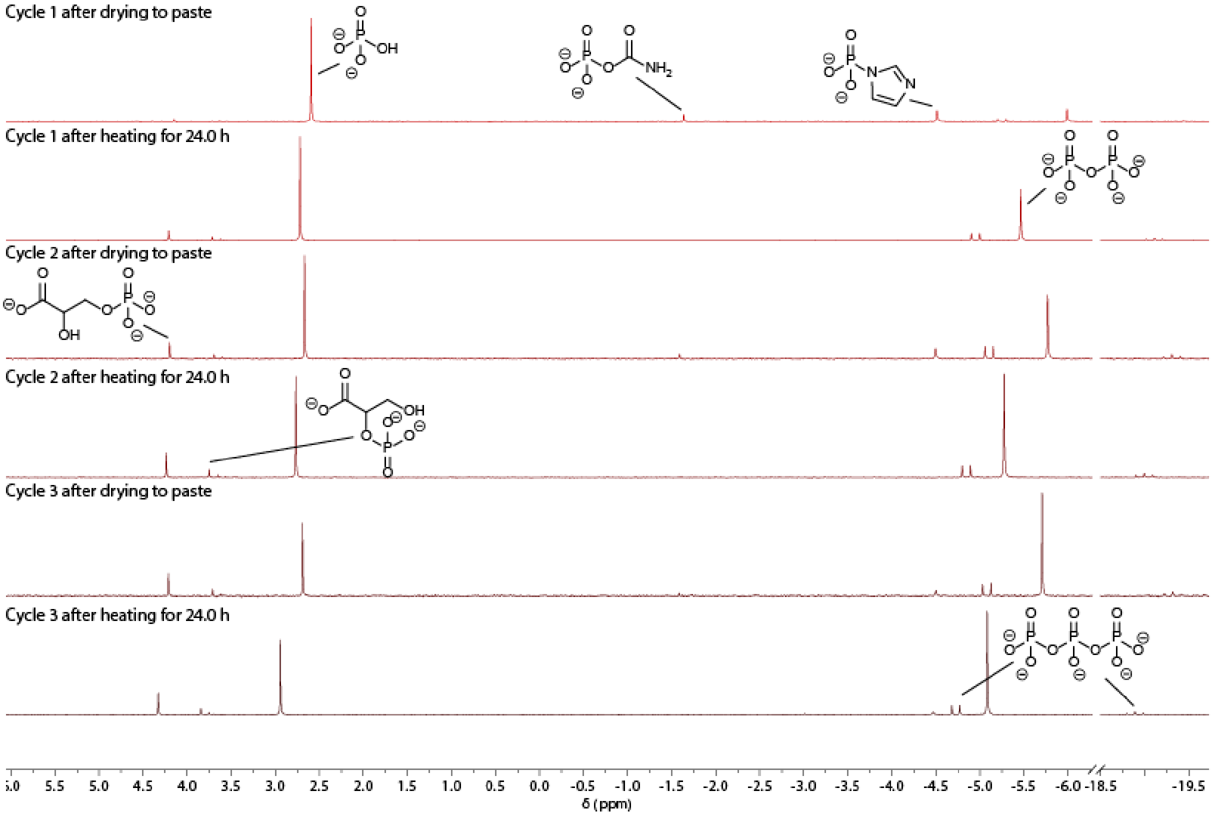

Supplementary Figure 179: Representative ^{31}P NMR spectra for the three cycles with 750 mM sodium glycerate, 450 mM potassium cyanate and 150 mM sodium phosphate in 1.0 M imidazole buffer. For each cycle the ^{31}P NMR spectrum after the reaction had been dried to a paste and the ^{31}P NMR spectrum after heating the reaction to 50 °C for ~24 h are shown. All NMR spectra were measured in a 0.5 M citric acid buffer at pH 6.85 9 : 1 H_2O : D_2O. The chemical shift of the pyrophosphate peak is variable at pH 6.85 due to the pyrophosphate pK_a = 6.6.

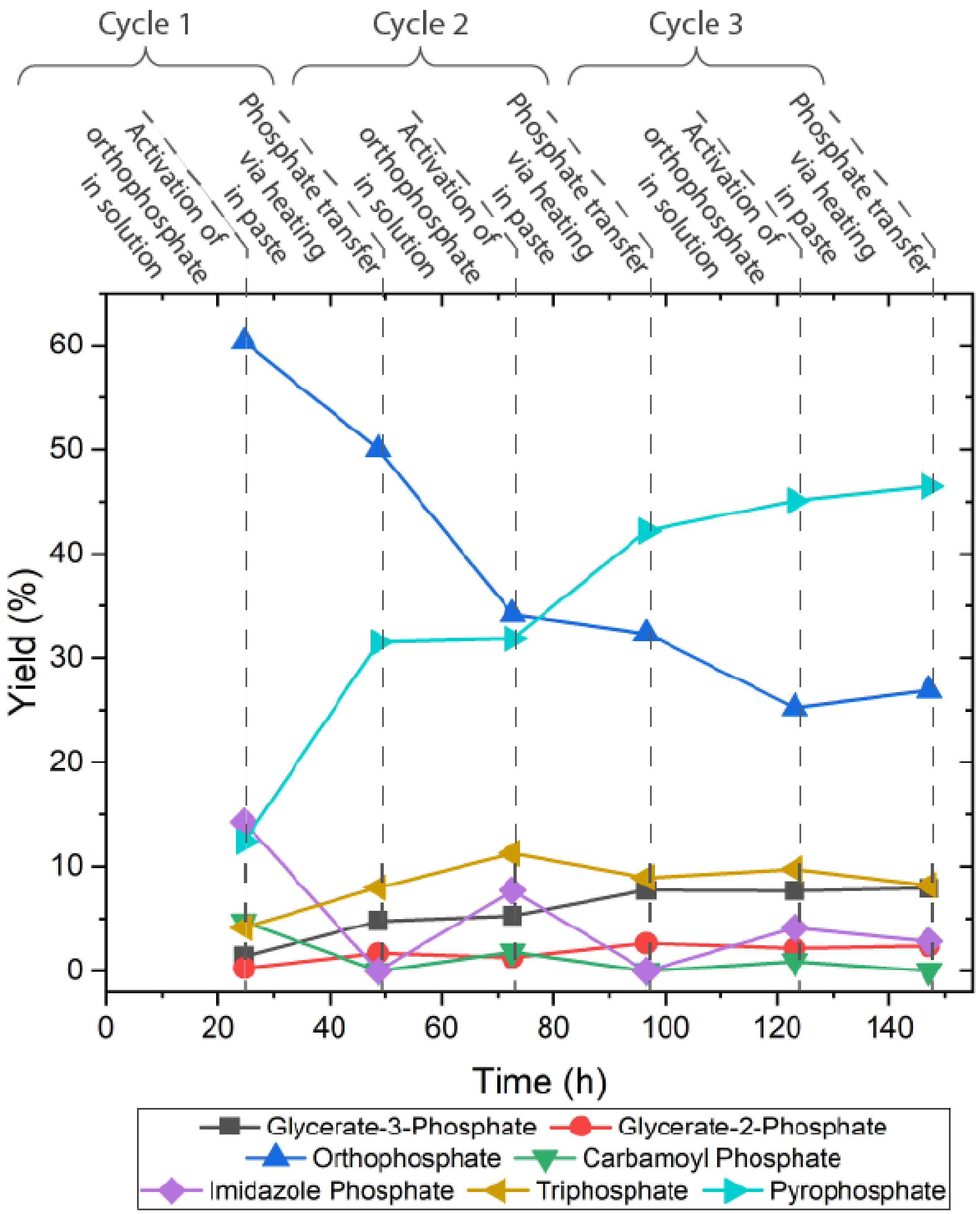

Supplementary Figure 180: Changes in yield for all phosphorous species over the course of three cycles for the reaction with 750 mM sodium glycerate, 450 mM potassium cyanate and 150 mM sodium phosphate in 1.0 M imidazole buffer.

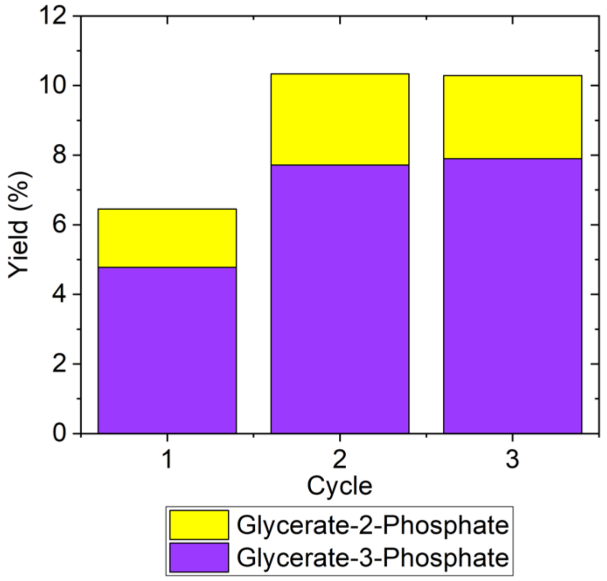

Supplementary Figure 181: Yield of glycerate-2-phosphate and glycerate-3-phosphate after each cycle for the reaction with 750 mM sodium glycerate, 450 mM potassium cyanate and 150 mM sodium phosphate in 1.0 M imidazole buffer.

S7.3.3 Phosphorylation of glycerate with 150 mM orthophosphate repeat 2

This reaction was performed according to the procedure in Section S7.1 but with 750 mM of sodium glycerate (318.2 mg, 3.0 mmol).

A total of three cycles were performed. Formation of imidazole phosphate was observed in solution and remained present after drying the solution to a paste. Formation of glycerate-2-phosphate and glycerate-3-phosphate occurred during both the drying to the paste over a period of ~24 h and during the heating of the paste to 50 °C for ~24 h (Supplementary Figure 182, 183 and 184).

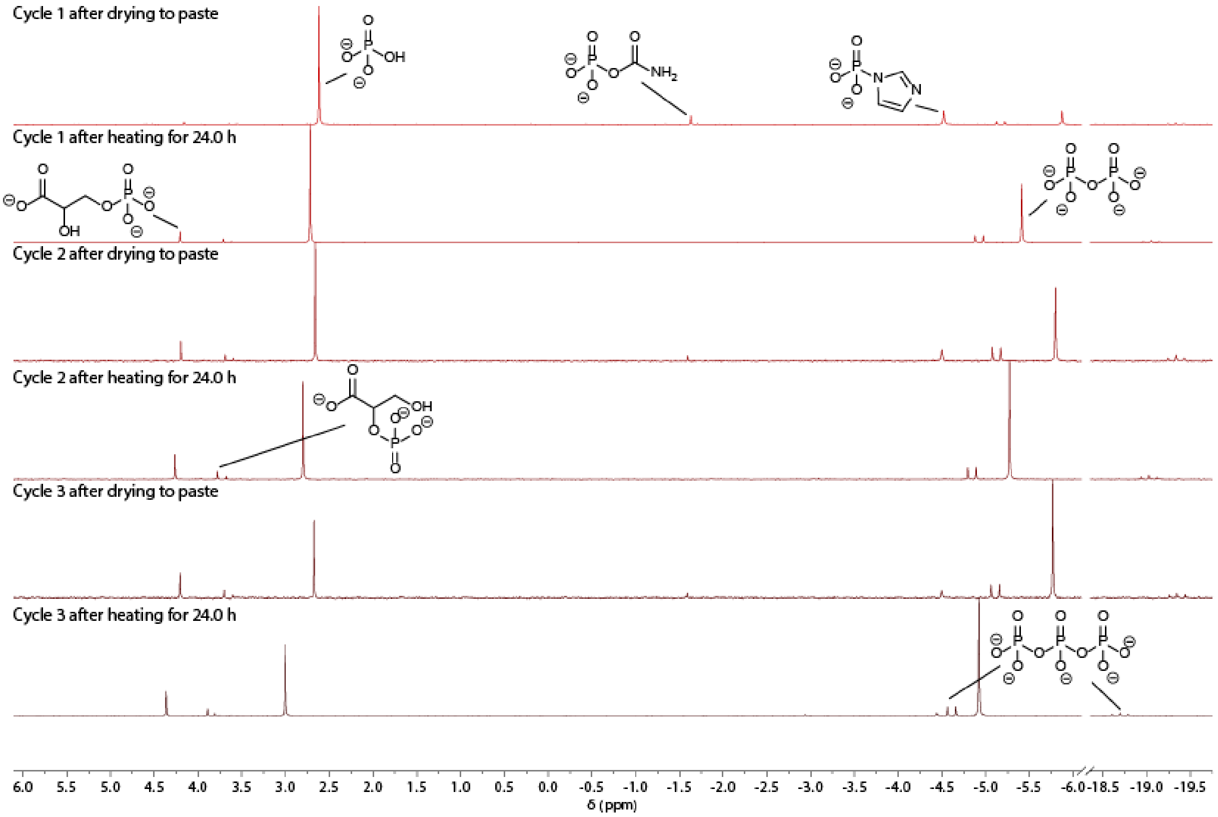

Supplementary Figure 182: Representative ^{31}P NMR spectra for the three cycles with 750 mM sodium glycerate, 450 mM potassium cyanate and 150 mM sodium phosphate in 1.0 M imidazole buffer. For each cycle the ^{31}P NMR spectrum after the reaction had been dried to a paste and the ^{31}P NMR spectrum after heating the reaction to 50 °C for ~24 h are shown. All NMR spectra were measured in a 0.5 M citric acid buffer at pH 6.85 9 : 1 H_2O : D_2O. The chemical shift of the pyrophosphate peak is variable at pH 6.85 due to the pyrophosphate pK_a = 6.6.

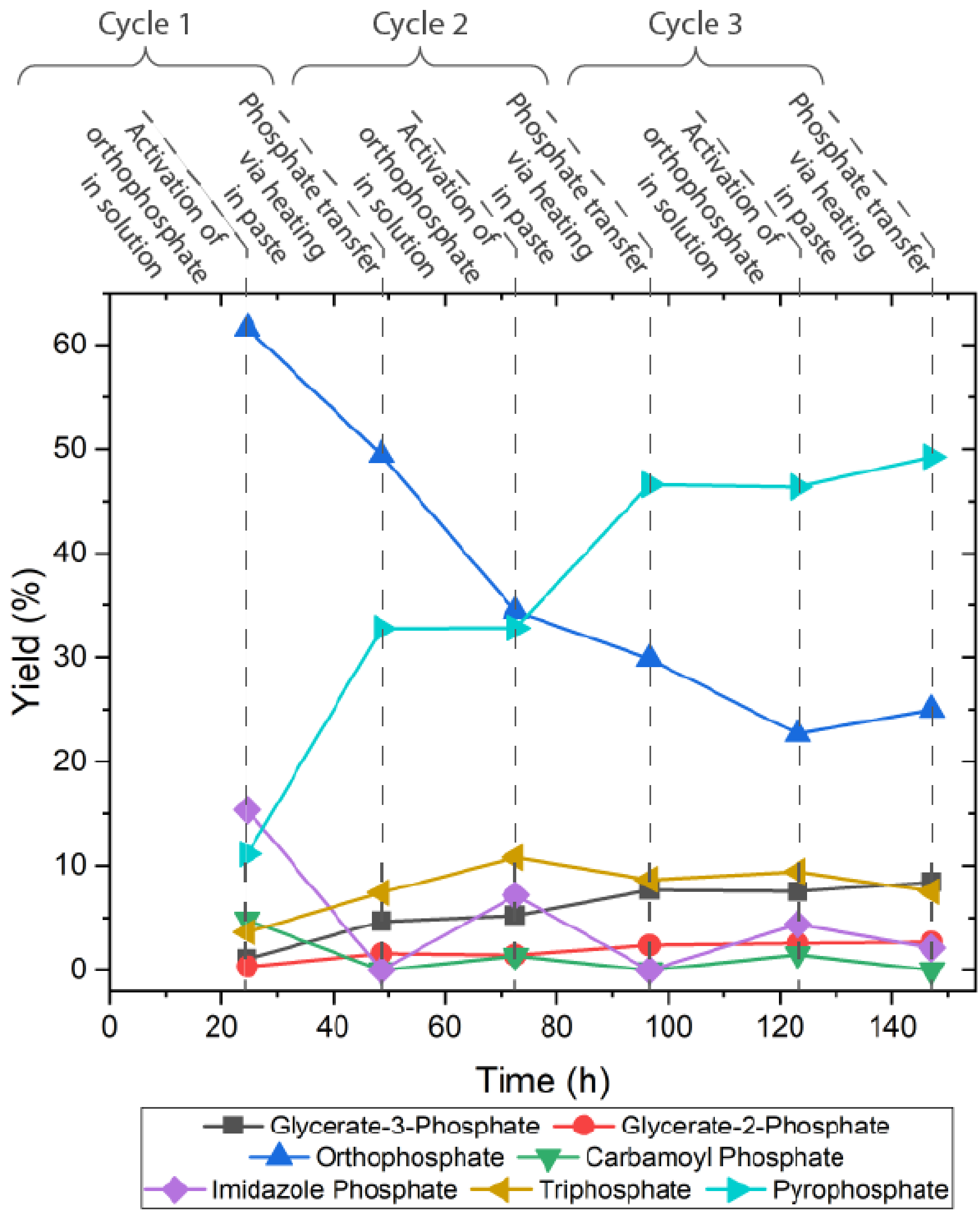

Supplementary Figure 183: Changes in yield for all phosphorous species over the course of three cycles for the reaction with 750 mM sodium glycerate, 450 mM potassium cyanate and 150 mM sodium phosphate in 1.0 M imidazole buffer.

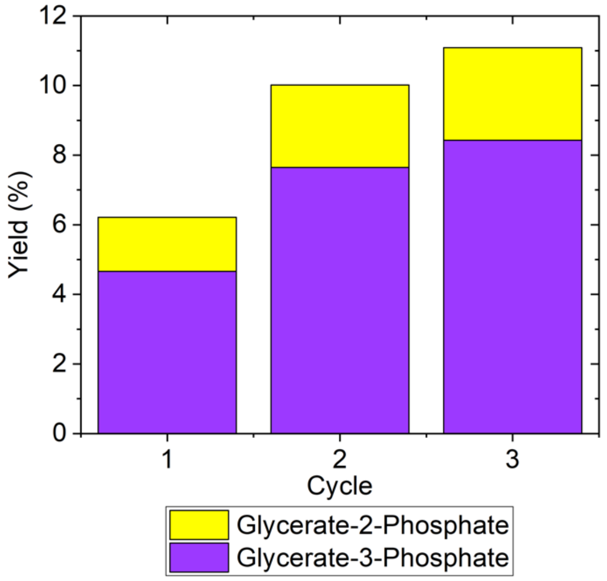

Supplementary Figure 184: Yield of glycerate-2-phosphate and glycerate-3-phosphate after each cycle for the reaction with 750 mM sodium glycerate, 450 mM potassium cyanate and 150 mM sodium phosphate in 1.0 M imidazole buffer.

S7.3.4 Phosphorylation of glycerate with 50 mM orthophosphate

This reaction was performed according to the procedure in Section S7.1 but with 750 mM of sodium glycerate (318.2 mg, 3.0 mmol) and 50 mM sodium phosphate dibasic dihydrate salt (178.0 mg, 1.0 mmol).

A total of three cycles were performed. Formation of imidazole phosphate was observed in solution and remained present after drying the solution to a paste. Formation of glycerate-2-phosphate and glycerate-3-phosphate occurred during both the drying to the paste over a period of ~24 h and during the heating of the paste to 50 °C for ~24 h (Supplementary Figure 185, 186 and 187).

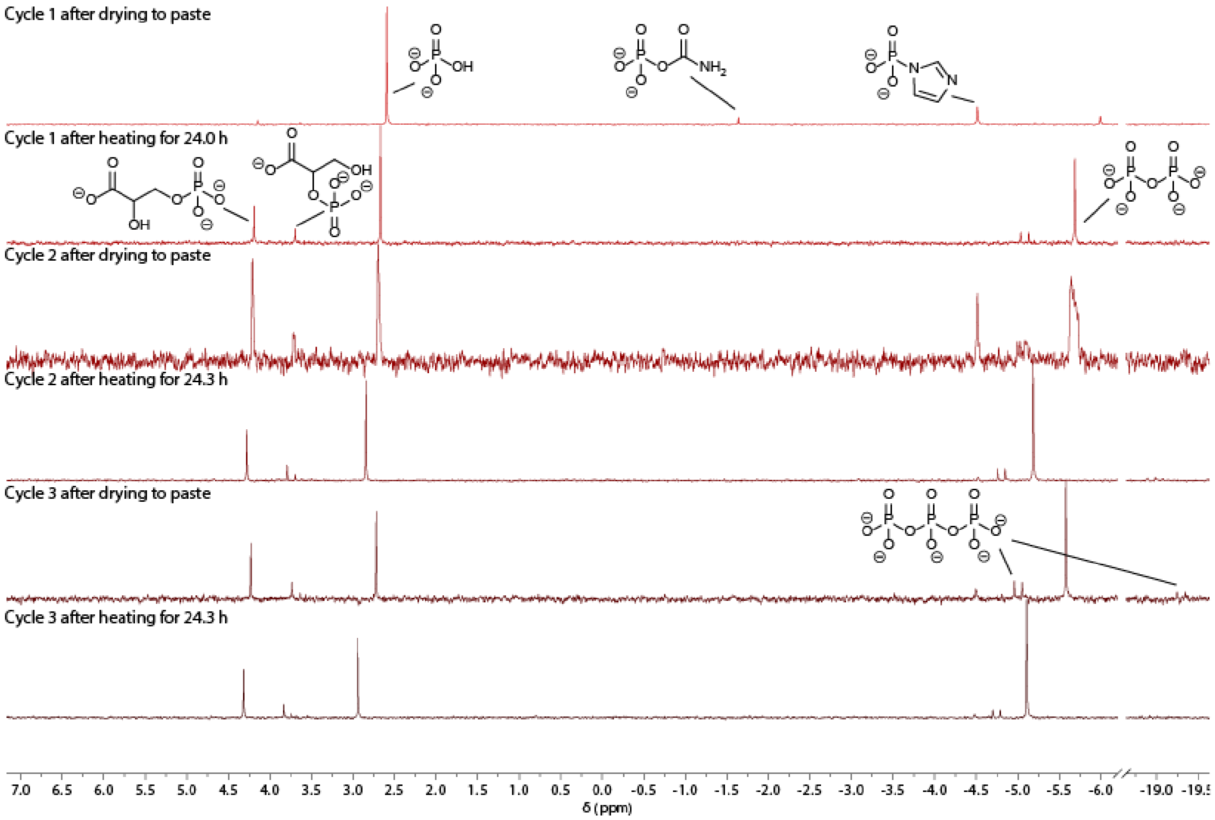

Supplementary Figure 185: Representative ^{31}P NMR spectra for the three cycles with 750 mM sodium glycerate, 450 mM potassium cyanate and 50 mM sodium phosphate in 1.0 M imidazole buffer. For each cycle the ^{31}P NMR spectrum after the reaction had been dried to a paste and the ^{31}P NMR spectrum after heating the reaction to 50 °C for ~24 h are shown. All NMR spectra were measured in a 0.5 M citric acid buffer at pH 6.85 9 : 1 H_2O : D_2O. The chemical shift of the pyrophosphate peak is variable at pH 6.85 due to the pyrophosphate pK_a = 6.6.

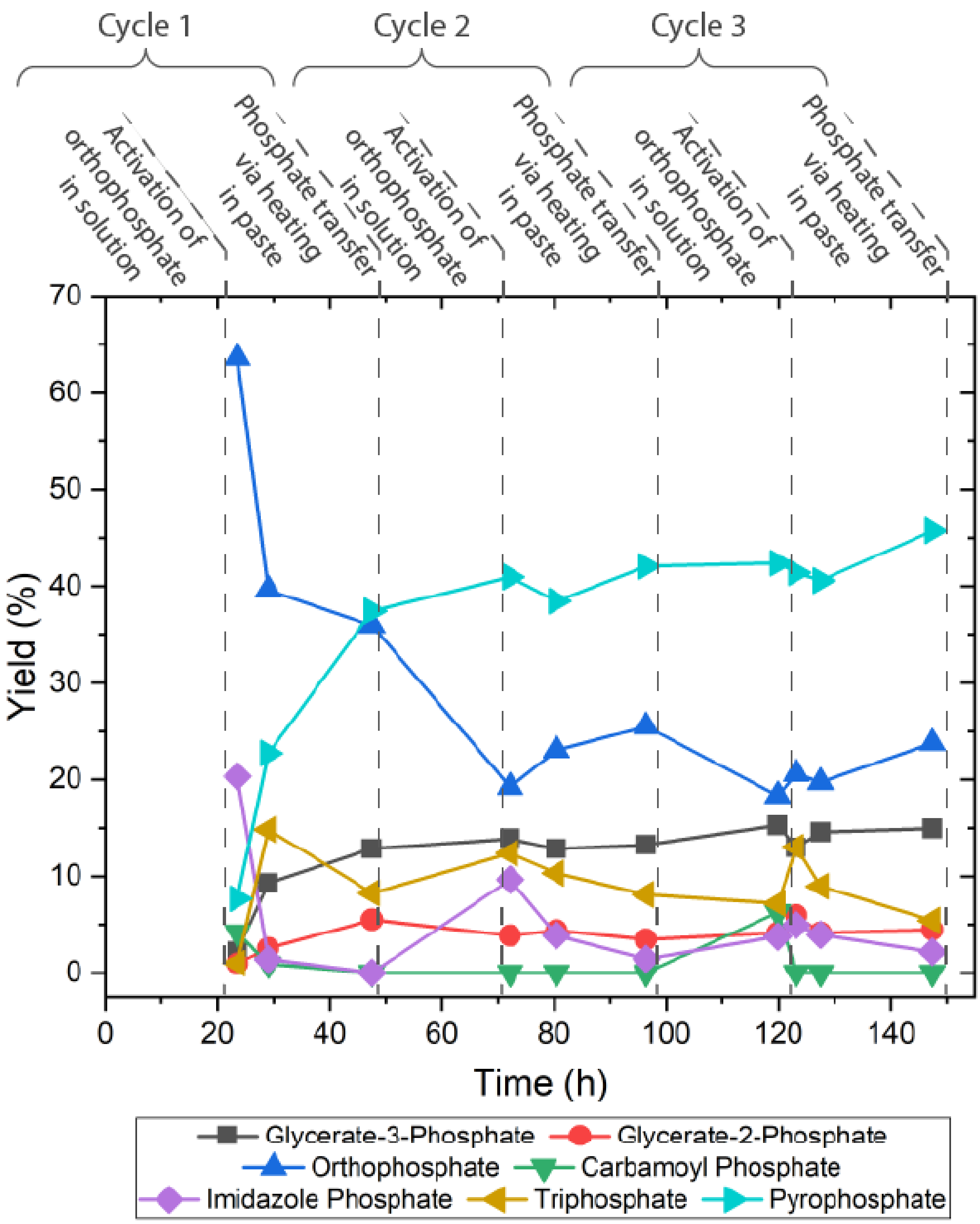

Supplementary Figure 186: Changes in yield for all phosphorous species over the course of three cycles for the reaction with 750 mM sodium glycerate, 450 mM potassium cyanate and 50 mM sodium phosphate in 1.0 M imidazole buffer.

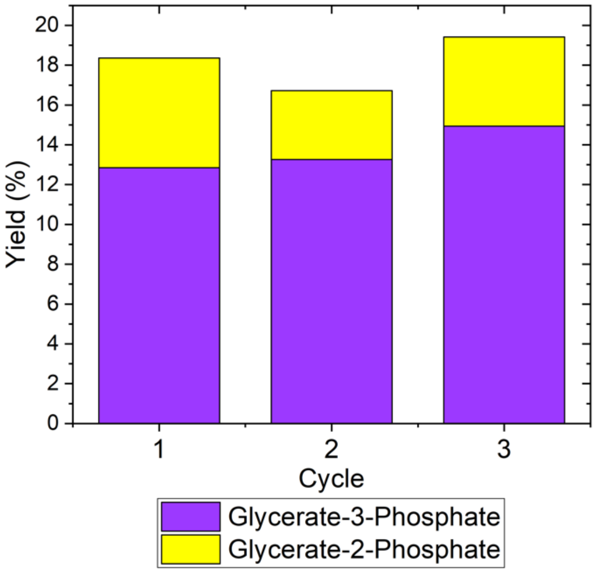

Supplementary Figure 187: Yield of glycerate-2-phosphate and glycerate-3-phosphate after each cycle for the reaction with 750 mM sodium glycerate, 450 mM potassium cyanate and 50 mM sodium phosphate in 1.0 M imidazole buffer.

A spiking experiment with genuine samples of glycerate-2-phosphate and glycerate-3-phosphate confirmed their formation (Supplementary Figure 188).

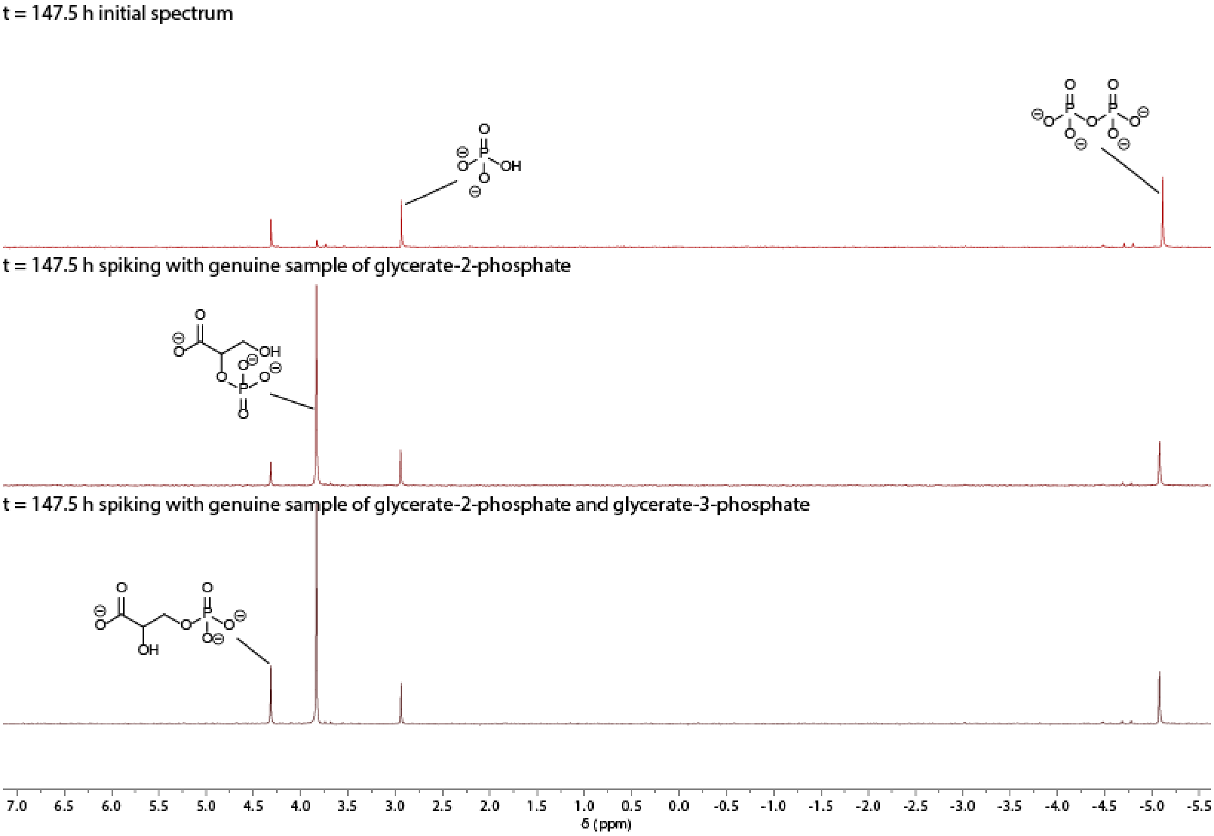

Supplementary Figure 188: ^{31}P NMR spectra of a spiking experiment for the third cycle of the reaction with 750 mM sodium glycerate, 450 mM potassium cyanate and 50 mM sodium phosphate in 1.0 M imidazole buffer. Top spectrum is unspiked. Middle spectrum is with the addition of genuine glycerate-2-phosphate. Bottom spectrum is with the addition of genuine glycerate-3-phosphate.

S7.3.5 Phosphorylation of glycerate with 50 mM orthophosphate repeat 1

This reaction was performed according to the procedure in Section S7.1 but with 750 mM of sodium glycerate (318.2 mg, 3.0 mmol) and 50 mM sodium phosphate dibasic dihydrate salt (178.0 mg, 1.0 mmol).

A total of three cycles were performed. Formation of imidazole phosphate was observed in solution and remained present after drying the solution to a paste. Formation of glycerate-2-phosphate and glycerate-3-phosphate occurred during both the drying to the paste over a period of ~24 h and during the heating of the paste to 50 °C for ~24 h (Supplementary Figure 189, 190 and 191).

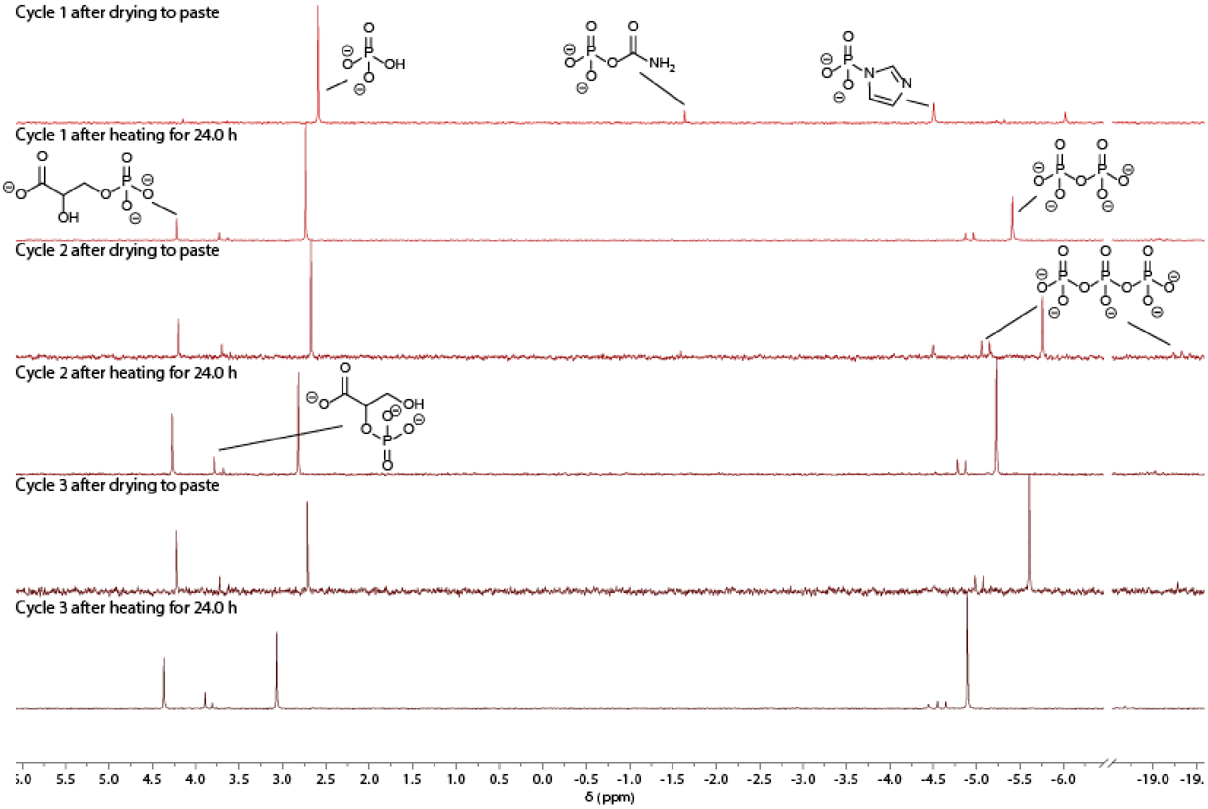

Supplementary Figure 189: Representative ^{31}P NMR spectra for the three cycles with 750 mM sodium glycerate, 450 mM potassium cyanate and 50 mM sodium phosphate in 1.0 M imidazole buffer. For each cycle the ^{31}P NMR spectrum after the reaction had been dried to a paste and the ^{31}P NMR spectrum after heating the reaction to 50 °C for ~24 h are shown. All NMR spectra were measured in a 0.5 M citric acid buffer at pH 6.85 9 : 1 H_2O : D_2O. The chemical shift of the pyrophosphate peak is variable at pH 6.85 due to the pyrophosphate pK_a = 6.6.

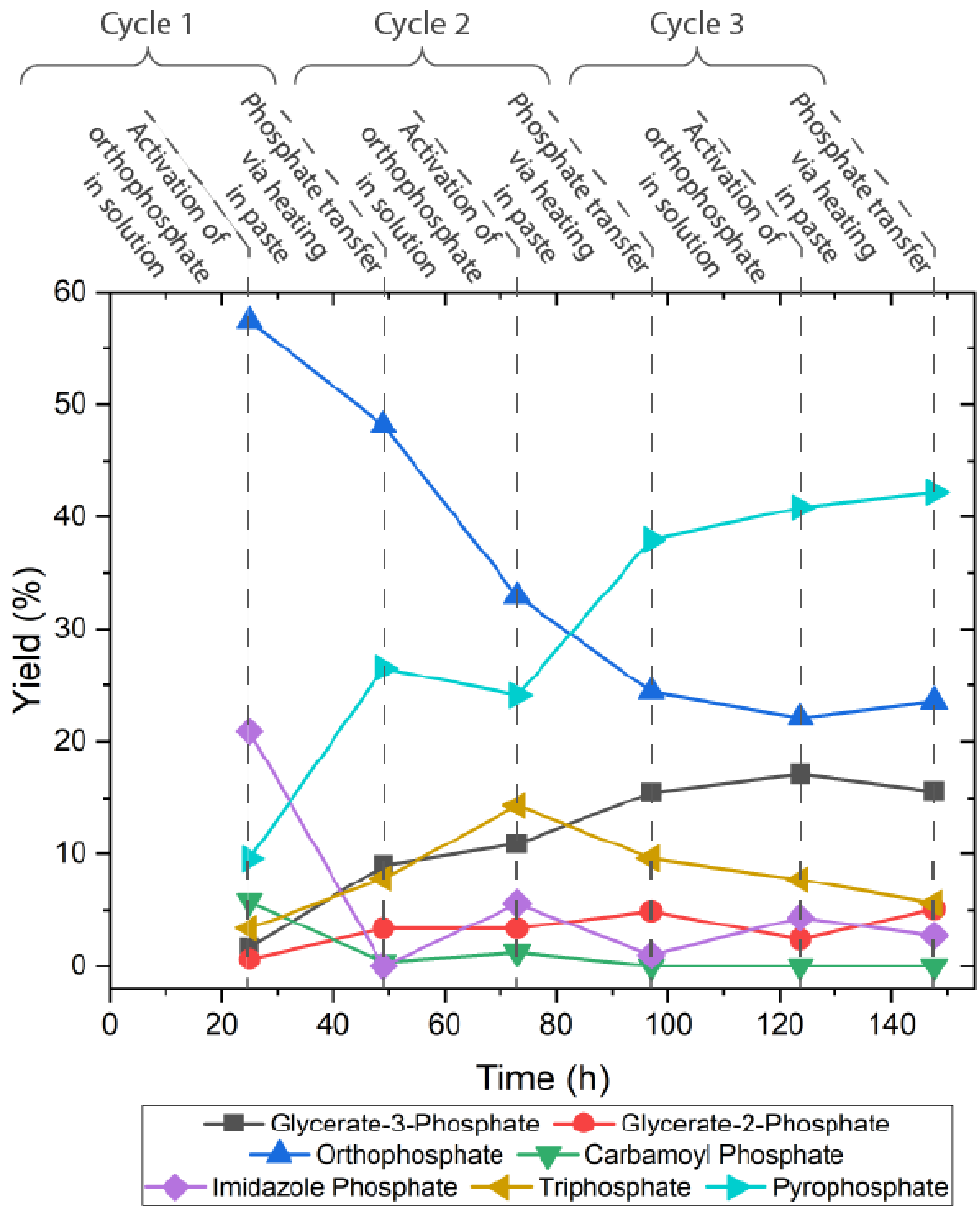

Supplementary Figure 190: Changes in yield for all phosphorous species over the course of three cycles for the reaction with 750 mM sodium glycerate, 450 mM potassium cyanate and 50 mM sodium phosphate in 1.0 M imidazole buffer.

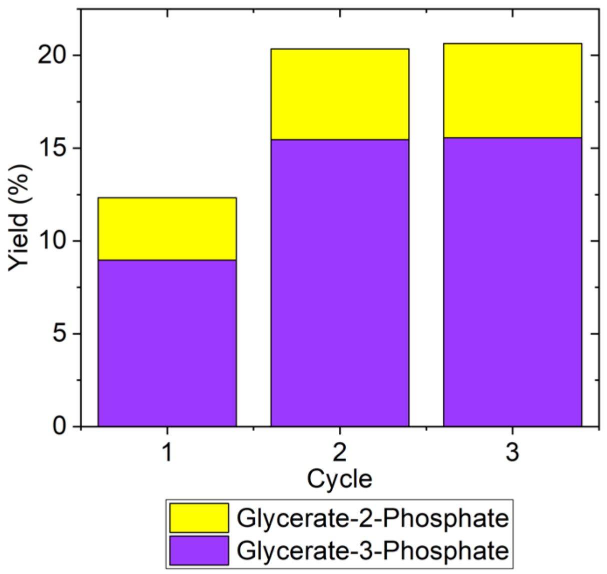

Supplementary Figure 191: Yield of glycerate-2-phosphate and glycerate-3-phosphate after each cycle for the reaction with 750 mM sodium glycerate, 450 mM potassium cyanate and 50 mM sodium phosphate in 1.0 M imidazole buffer.

S7.3.6 Phosphorylation of glycerate with 50 mM orthophosphate repeat 2

This reaction was performed according to the procedure in Section S7.1 but with 750 mM of sodium glycerate (318.2 mg, 3.0 mmol) and 50 mM sodium phosphate dibasic dihydrate salt (178.0 mg, 1.0 mmol).

A total of three cycles were performed. Formation of imidazole phosphate was observed in solution and remained present after drying the solution to a paste. Formation of glycerate-2-phosphate and glycerate-3-phosphate occurred during both the drying to the paste over a period of ~24 h and during the heating of the paste to 50 °C for ~24 h (Supplementary Figure 192, 193 and 194).

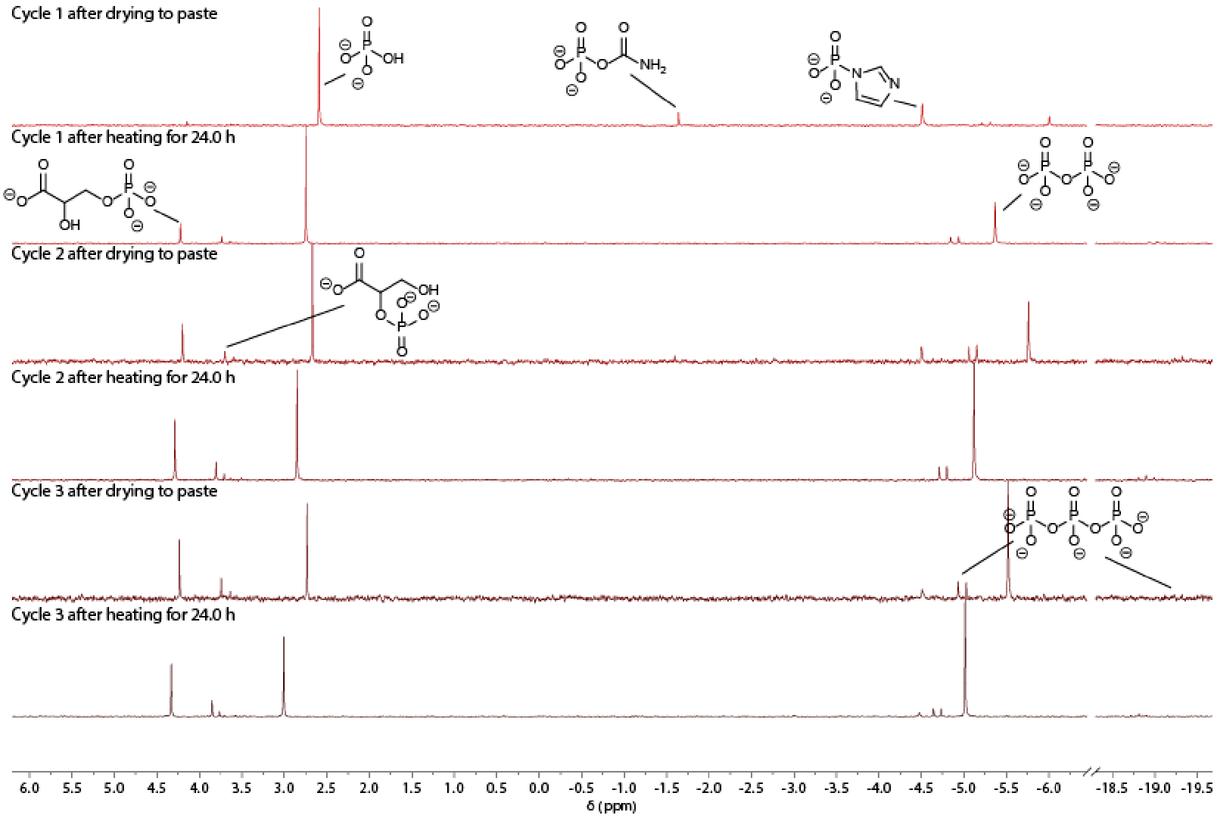

Supplementary Figure 192: Representative ^{31}P NMR spectra for the three cycles with 750 mM sodium glycerate, 450 mM potassium cyanate and 50 mM sodium phosphate in 1.0 M imidazole buffer. For each cycle the ^{31}P NMR spectrum after the reaction had been dried to a paste and the ^{31}P NMR spectrum after heating the reaction to 50 °C for ~24 h are shown. All NMR spectra were measured in a 0.5 M citric acid buffer at pH 6.85 9 : 1 H_2O : D_2O. The chemical shift of the pyrophosphate peak is variable at pH 6.85 due to the pyrophosphate pK_a = 6.6.

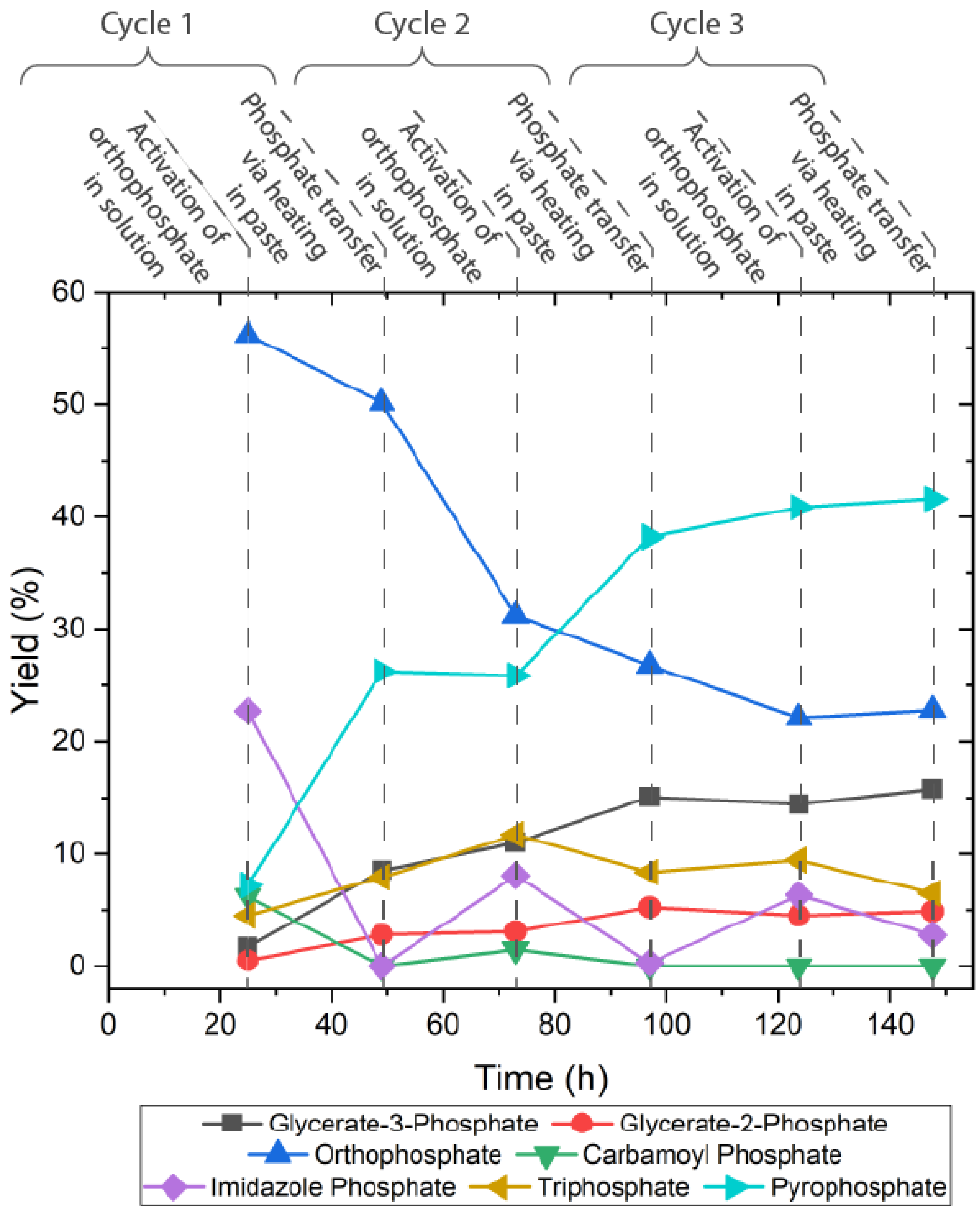

Supplementary Figure 193: Changes in yield for all phosphorous species over the course of three cycles for the reaction with 750 mM sodium glycerate, 450 mM potassium cyanate and 50 mM sodium phosphate in 1.0 M imidazole buffer.

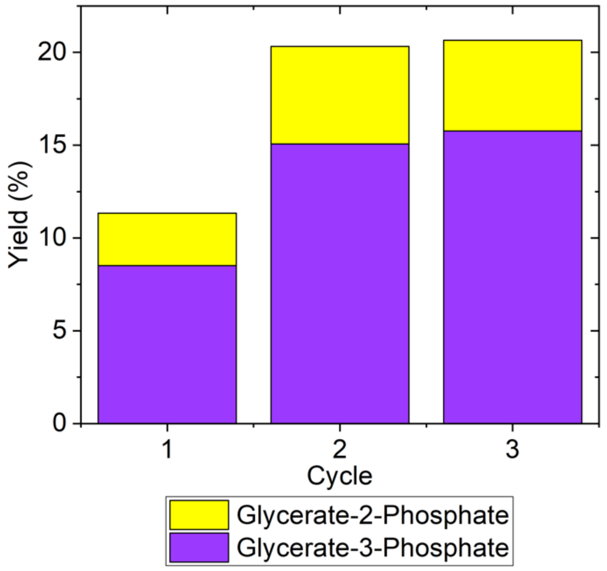

Supplementary Figure 194: Yield of glycerate-2-phosphate and glycerate-3-phosphate after each cycle for the reaction with 750 mM sodium glycerate, 450 mM potassium cyanate and 50 mM sodium phosphate in 1.0 M imidazole buffer.

S7.3.7 Phosphorylation of glycerate with 20 mM orthophosphate

This reaction was performed according to the procedure in Section S7.1 but with 750 mM of sodium glycerate (318.2 mg, 3.0 mmol) and 20 mM sodium phosphate dibasic dihydrate salt (71.2 mg, 0.4 mmol).

A total of three cycles were performed. Formation of imidazole phosphate was observed in solution and remained present after drying the solution to a paste. Formation of glycerate-2-phosphate and glycerate-3-phosphate occurred during both the drying to the paste over a period of ~24 h and during the heating of the paste to 50 °C for ~24 h (Supplementary Figure 195, 196 and 197).

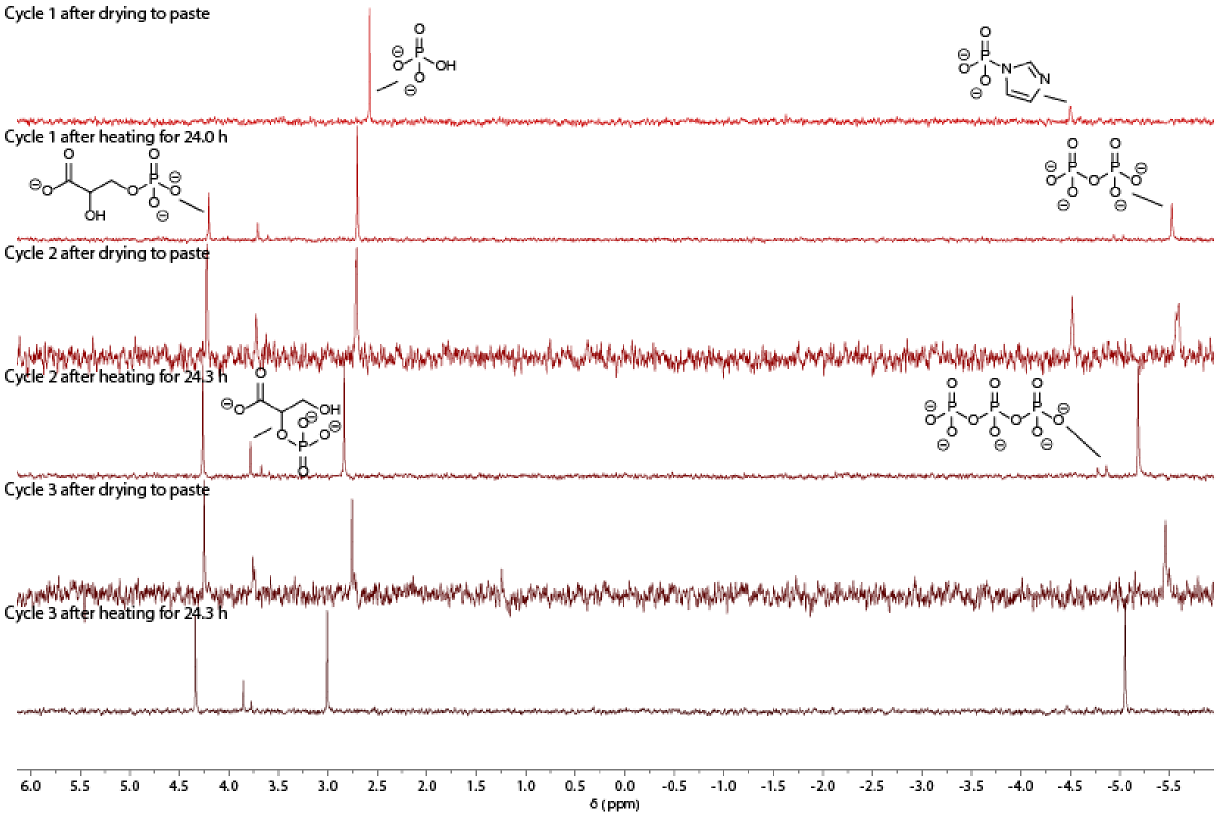

Supplementary Figure 195: Representative ^{31}P NMR spectra for the three cycles with 750 mM sodium glycerate, 450 mM potassium cyanate and 20 mM sodium phosphate in 1.0 M imidazole buffer. For each cycle the ^{31}P NMR spectrum after the reaction had been dried to a paste and the ^{31}P NMR spectrum after heating the reaction to 50 °C for ~24 h are shown. All NMR spectra were measured in a 0.5 M citric acid buffer at pH 6.85 9 : 1 H_2O : D_2O. The chemical shift of the pyrophosphate peak is variable at pH 6.85 due to the pyrophosphate pK_a = 6.6.

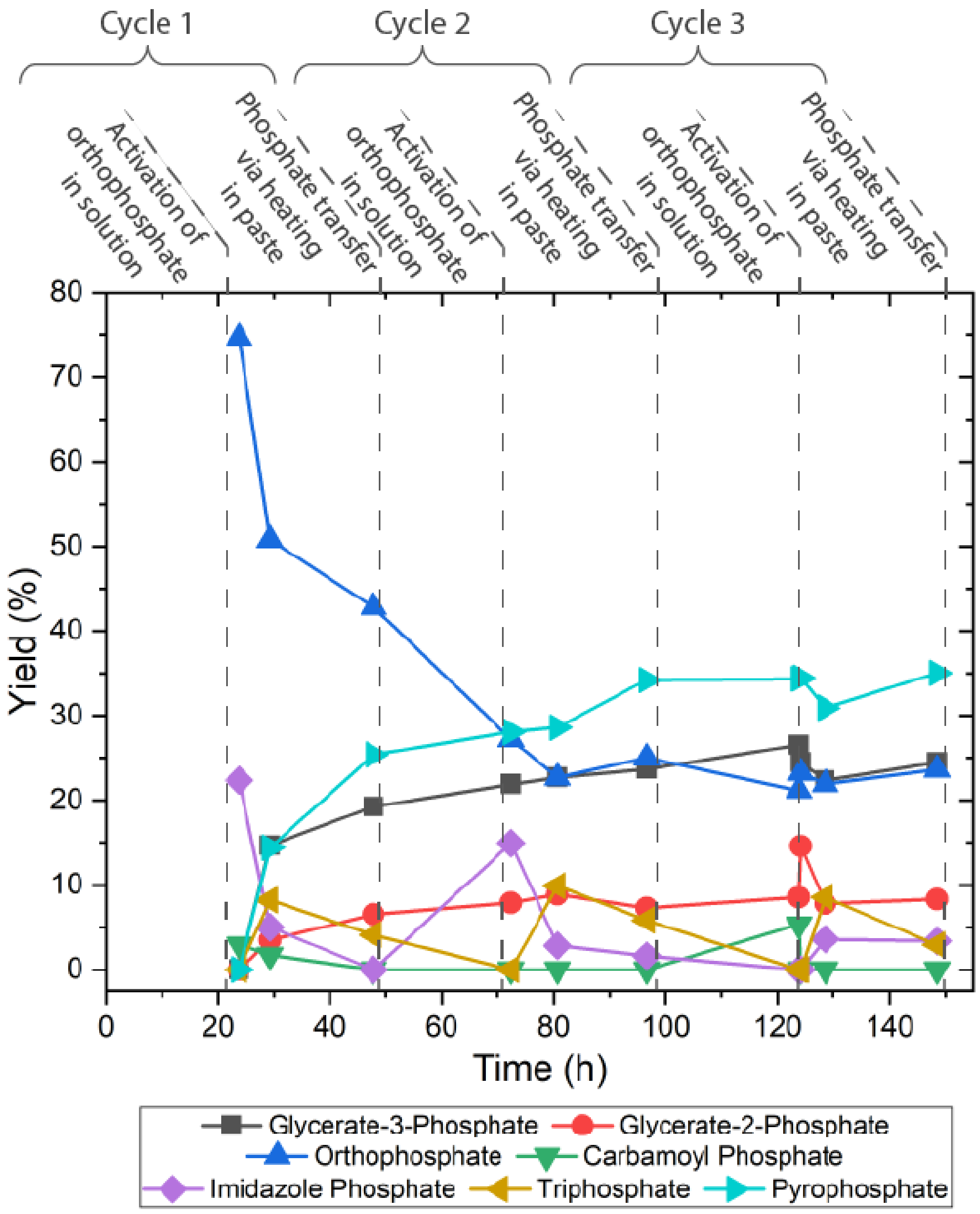

Supplementary Figure 196: Changes in yield for all phosphorous species over the course of three cycles for the reaction with 750 mM sodium glycerate, 450 mM potassium cyanate and 20 mM sodium phosphate in 1.0 M imidazole buffer.

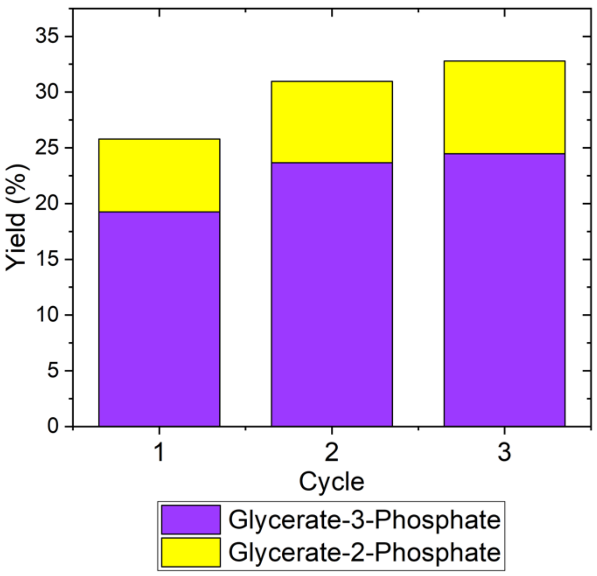

Supplementary Figure 197: Yield of glycerate-2-phosphate and glycerate-3-phosphate after each cycle for the reaction with 750 mM sodium glycerate, 450 mM potassium cyanate and 20 mM sodium phosphate in 1.0 M imidazole buffer.

A spiking experiment with genuine samples of glycerate-2-phosphate and glycerate-3-phosphate confirmed their formation (Supplementary Figure 198).

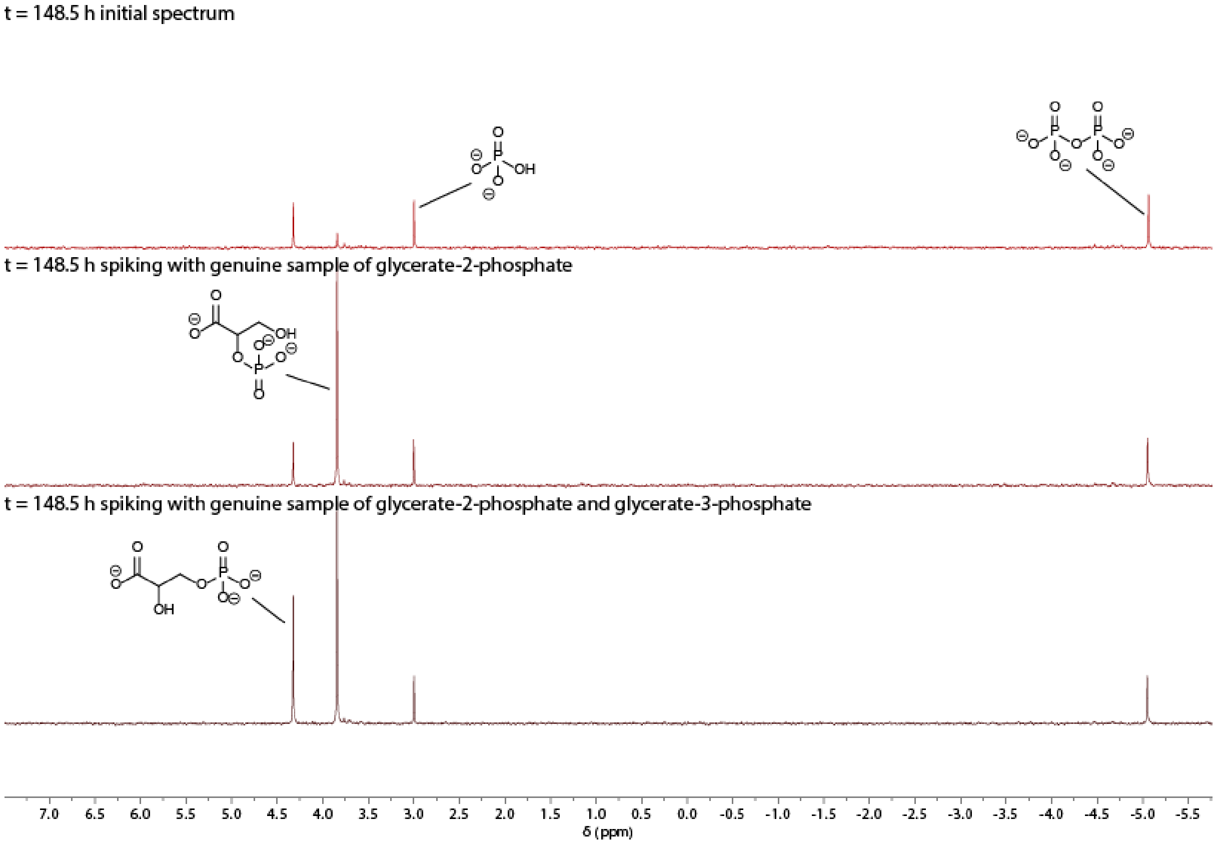

Supplementary Figure 198: ^{31}P NMR spectra of a spiking experiment for the third cycle of the reaction with 750 mM sodium glycerate, 450 mM potassium cyanate and 20 mM sodium phosphate in 1.0 M imidazole buffer. Top spectrum is unspiked. Middle spectrum is with the addition of genuine glycerate-2-phosphate. Bottom spectrum is with the addition of genuine glycerate-3-phosphate.

S7.3.8 Phosphorylation of glycerate with 20 mM orthophosphate repeat 1

This reaction was performed according to the procedure in Section S7.1 but with 750 mM of sodium glycerate (318.2 mg, 3.0 mmol) and 20 mM sodium phosphate dibasic dihydrate salt (71.2 mg, 0.4 mmol).

A total of three cycles were performed. Formation of imidazole phosphate was observed in solution and remained present after drying the solution to a paste. Formation of glycerate-2-phosphate and glycerate-3-phosphate occurred during both the drying to the paste over a period of ~24 h and during the heating of the paste to 50 °C for ~24 h (Supplementary Figure 199, 200 and 201).

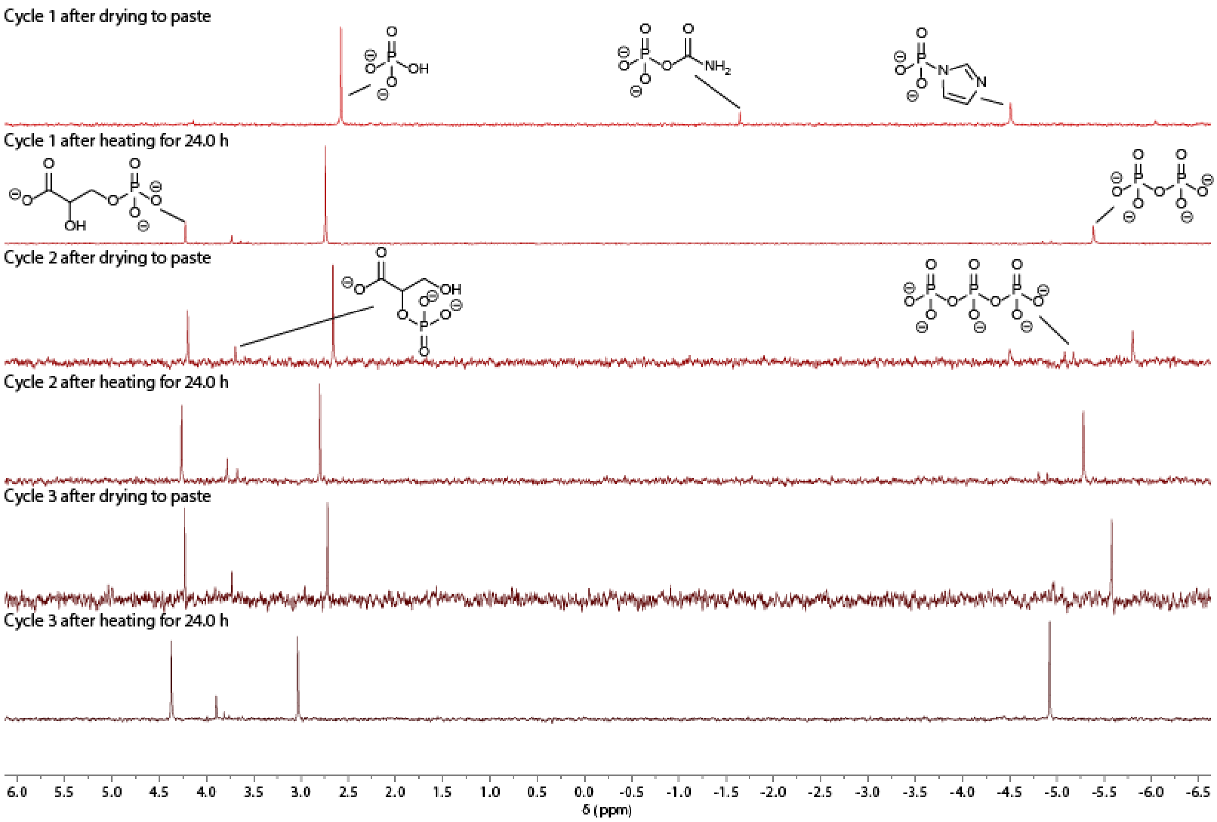

Supplementary Figure 199: Representative ^{31}P NMR spectra for the three cycles with 750 mM sodium glycerate, 450 mM potassium cyanate and 20 mM sodium phosphate in 1.0 M imidazole buffer. For each cycle the ^{31}P NMR spectrum after the reaction had been dried to a paste and the ^{31}P NMR spectrum after heating the reaction to 50 °C for ~24 h are shown. All NMR spectra were measured in a 0.5 M citric acid buffer at pH 6.85 9 : 1 H_2O : D_2O. The chemical shift of the pyrophosphate peak is variable at pH 6.85 due to the pyrophosphate pK_a = 6.6.

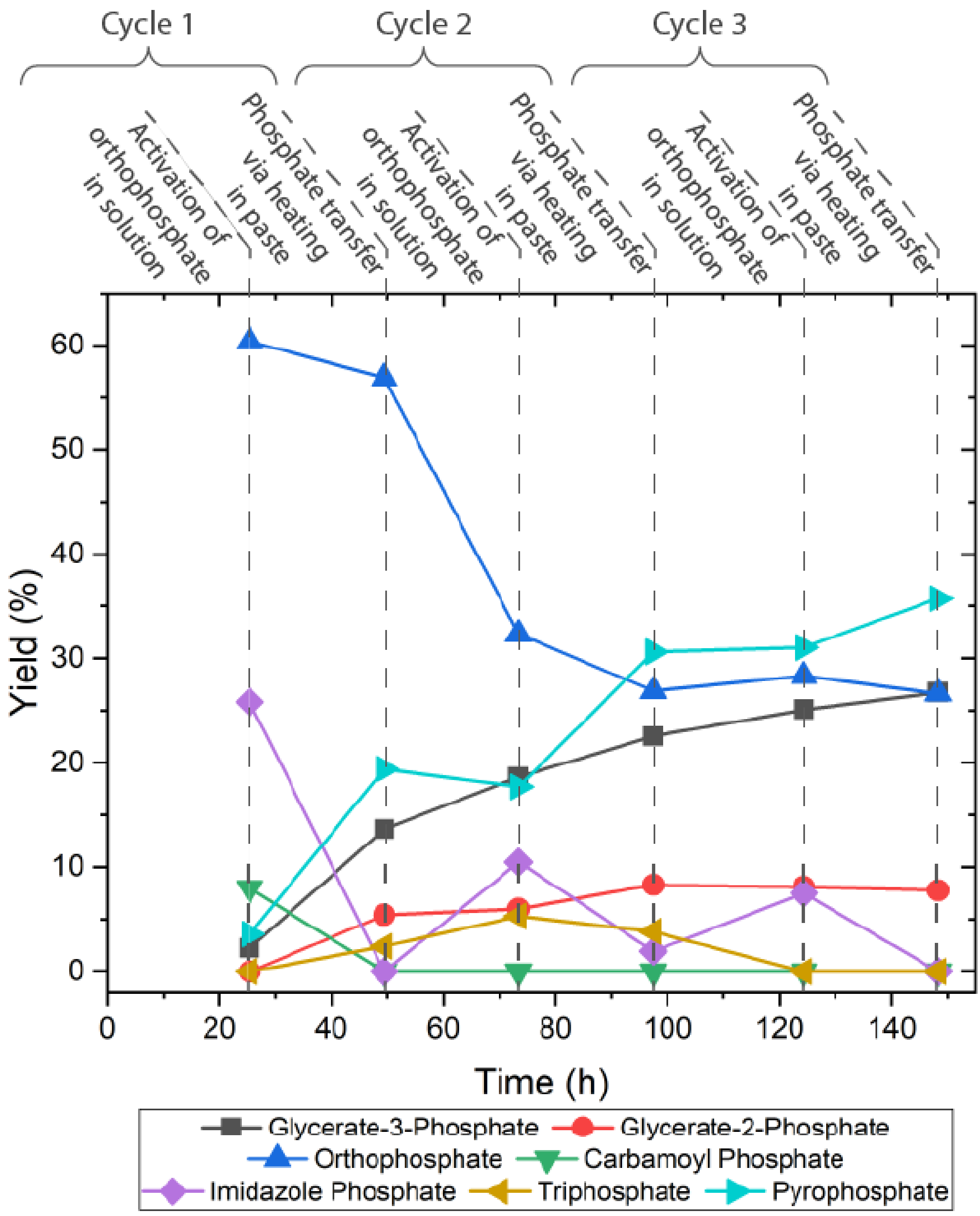

Supplementary Figure 200: Changes in yield for all phosphorous species over the course of three cycles for the reaction with 750 mM sodium glycerate, 450 mM potassium cyanate and 20 mM sodium phosphate in 1.0 M imidazole buffer.

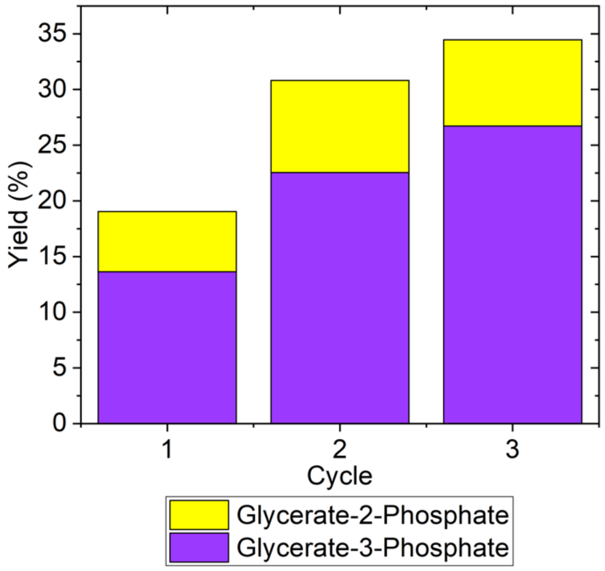

Supplementary Figure 201: Yield of glycerate-2-phosphate and glycerate-3-phosphate after each cycle for the reaction with 750 mM sodium glycerate, 450 mM potassium cyanate and 20 mM sodium phosphate in 1.0 M imidazole buffer.

S7.3.9 Phosphorylation of glycerate with 20 mM orthophosphate repeat 2

This reaction was performed according to the procedure in Section S7.1 but with 750 mM of sodium glycerate (318.2 mg, 3.0 mmol) and 20 mM sodium phosphate dibasic dihydrate salt (71.2 mg, 0.4 mmol).

A total of three cycles were performed. Formation of imidazole phosphate was observed in solution and remained present after drying the solution to a paste. Formation of glycerate-2-phosphate and glycerate-3-phosphate occurred during both the drying to the paste over a period of ~24 h and during the heating of the paste to 50 °C for ~24 h (Supplementary Figure 202, 203 and 204).

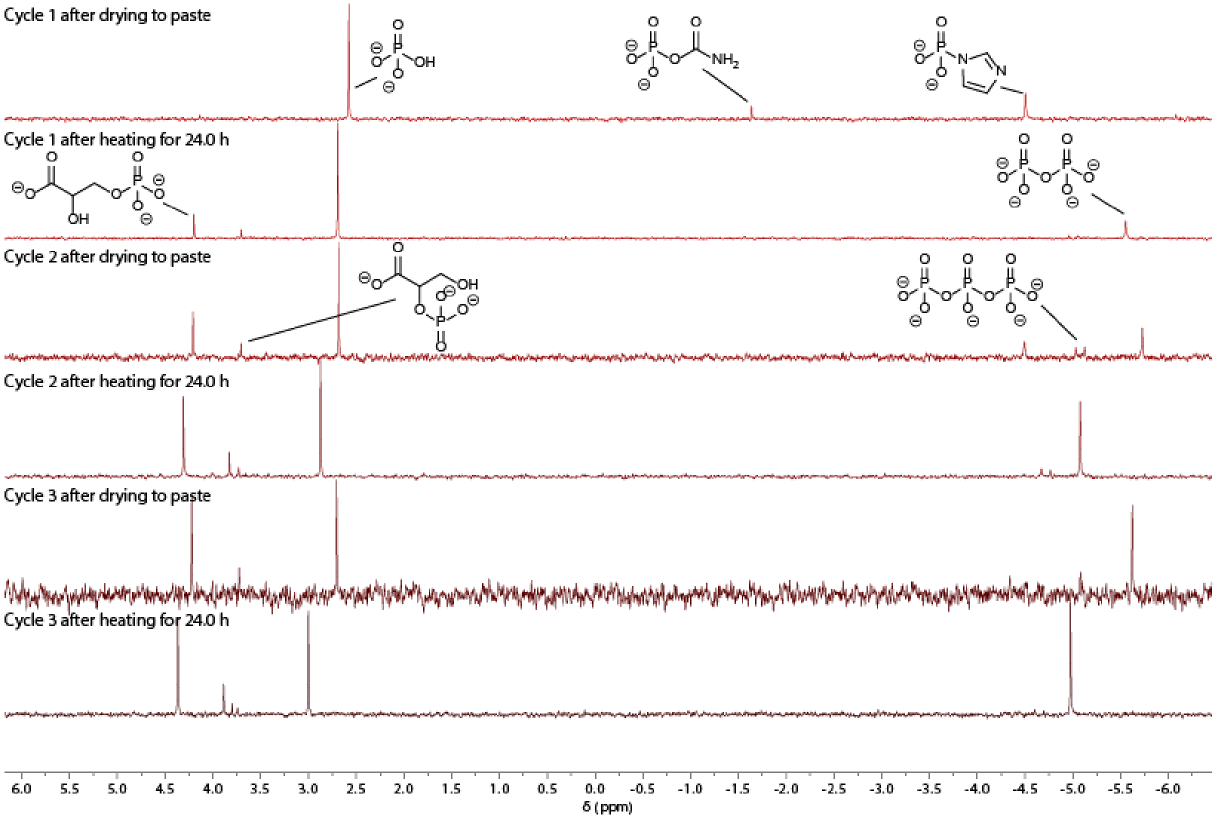

Supplementary Figure 202: Representative ^{31}P NMR spectra for the three cycles with 750 mM sodium glycerate, 450 mM potassium cyanate and 20 mM sodium phosphate in 1.0 M imidazole buffer. For each cycle the ^{31}P NMR spectrum after the reaction had been dried to a paste and the ^{31}P NMR spectrum after heating the reaction to 50 °C for ~24 h are shown. All NMR spectra were measured in a 0.5 M citric acid buffer at pH 6.85 9 : 1 H_2O : D_2O. The chemical shift of the pyrophosphate peak is variable at pH 6.85 due to the pyrophosphate pK_a = 6.6.

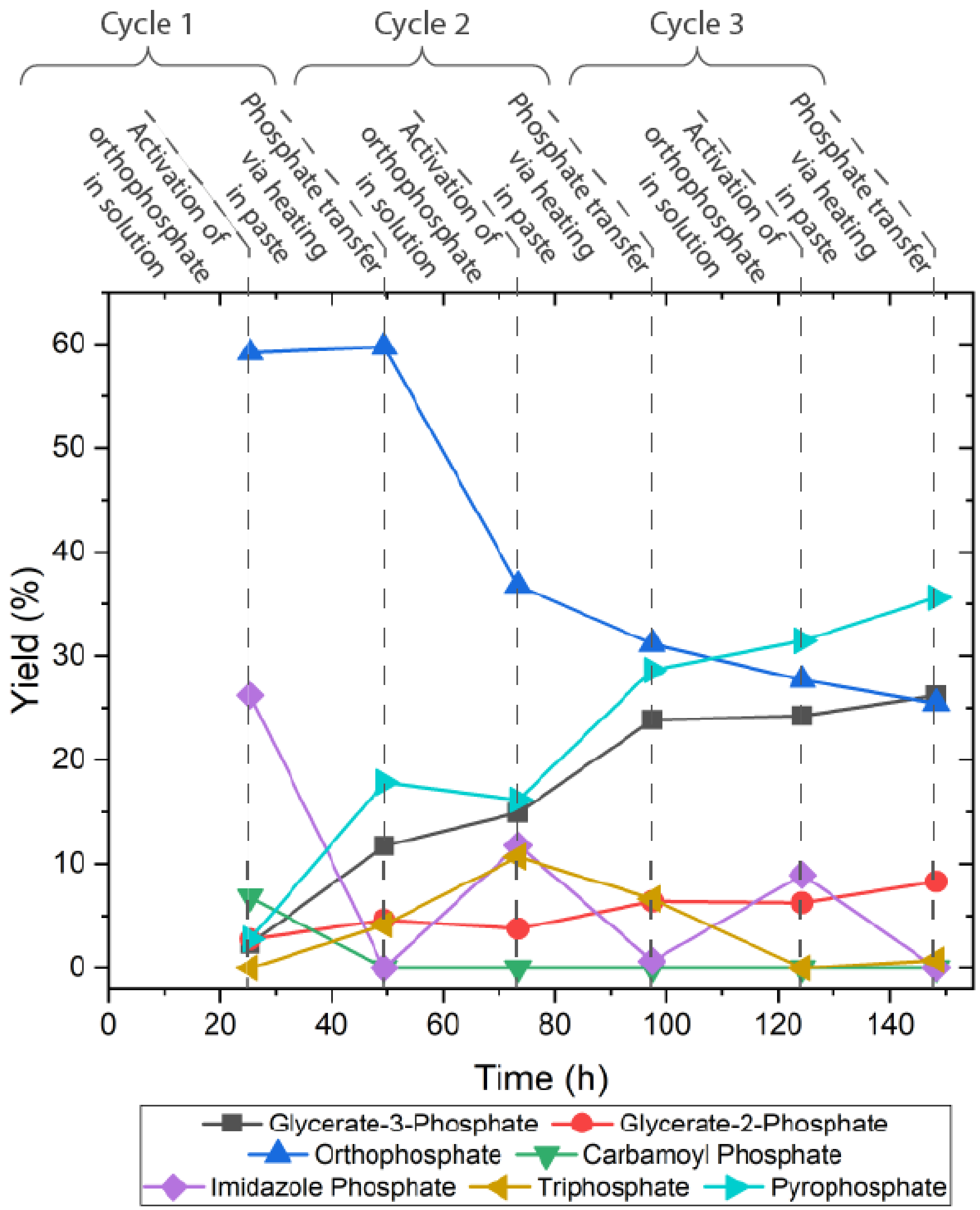

Supplementary Figure 203: Changes in yield for all phosphorous species over the course of three cycles for the reaction with 750 mM sodium glycerate, 450 mM potassium cyanate and 20 mM sodium phosphate in 1.0 M imidazole buffer.

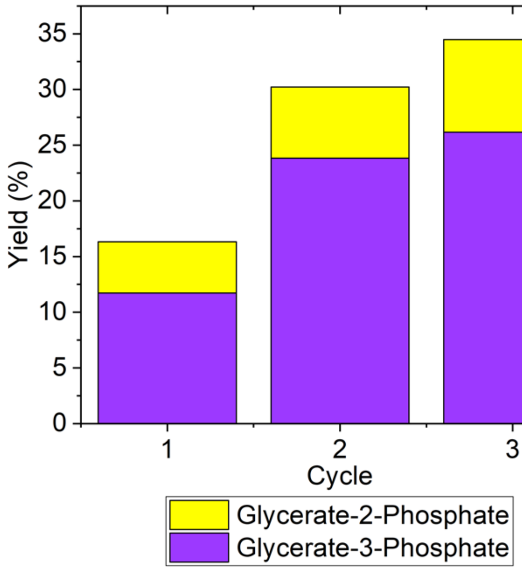

Supplementary Figure 204: Yield of glycerate-2-phosphate and glycerate-3-phosphate after each cycle for the reaction with 750 mM sodium glycerate, 450 mM potassium cyanate and 20 mM sodium phosphate in 1.0 M imidazole buffer.

S7.4 Phosphorylation of adenosine-5'-monophosphate with orthophosphate recycling

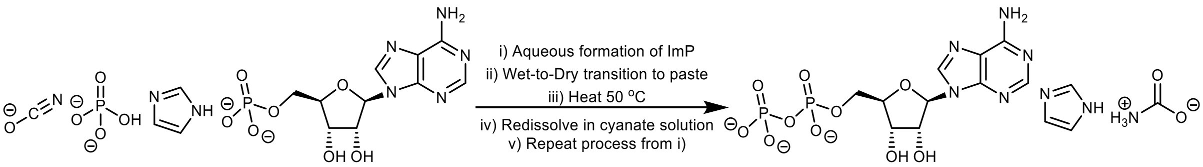

S7.4.1 Phosphorylation of adenosine-5'-monophosphate with 150 mM orthophosphate

This reaction was performed according to the procedure in Section S7.1 but with 750 mM of adenosine-5'-monophosphate disodium salt (1.096 g, 3.0 mmol).

A total of three cycles were performed. Formation of imidazole phosphate was observed in solution and remained present after drying the solution to a paste. Formation of adenosine-5'-diphosphate occurred during both the drying to the paste over a period of ~24 h and during the heating of the paste to 50 °C for ~24 h (Supplementary Figure 205, 206 and 207).

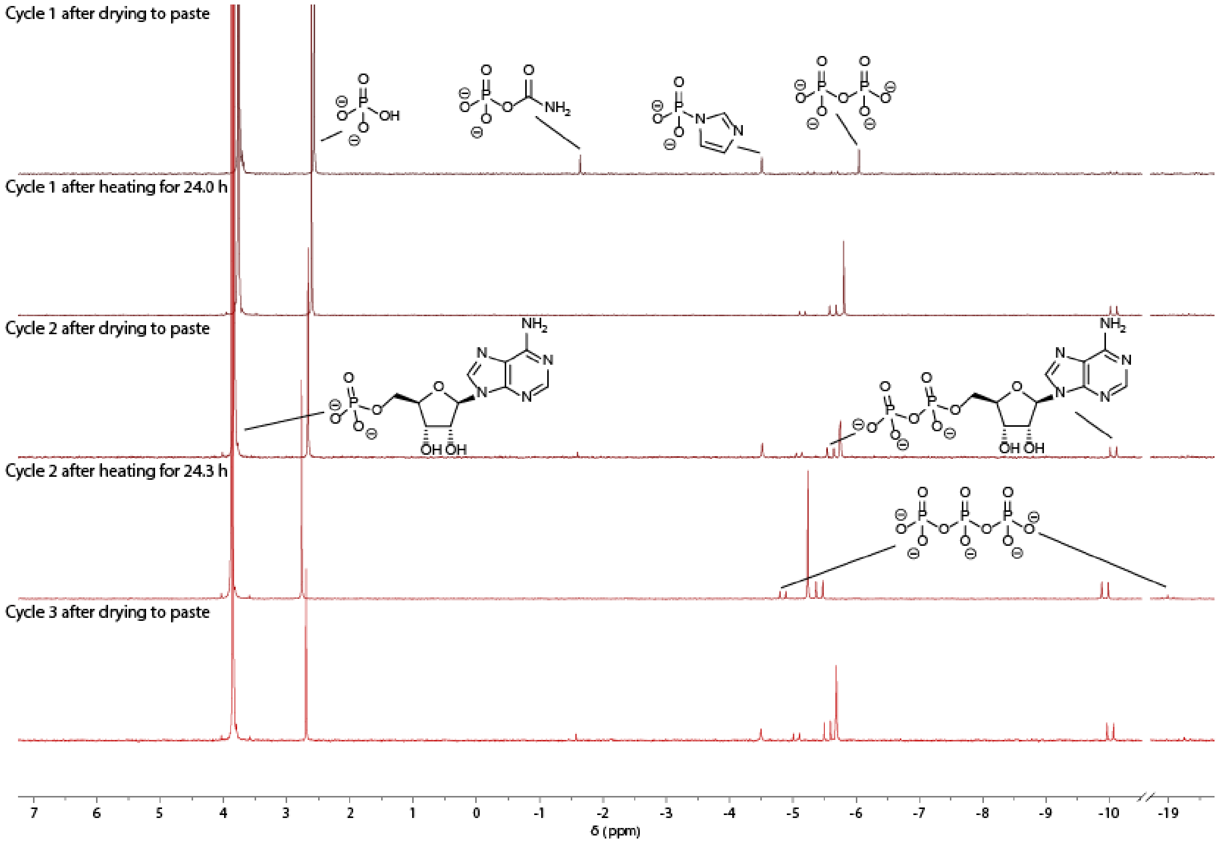

Supplementary Figure 205: Representative ^{31}P NMR spectra for the three cycles with 750 mM adenosine-5'-monophosphate, 450 mM potassium cyanate and 150 mM sodium phosphate in 1.0 M imidazole buffer. For each cycle the ^{31}P NMR spectrum after the reaction had been dried to a paste and the ^{31}P NMR spectrum after heating the reaction to 50 °C for ~24 h are shown. All NMR spectra were measured in a 0.5 M citric acid buffer at pH 6.85 9 : 1 H_2O : D_2O. The chemical shift of the pyrophosphate peak is variable at pH 6.85 due to the pyrophosphate pK_a = 6.6.

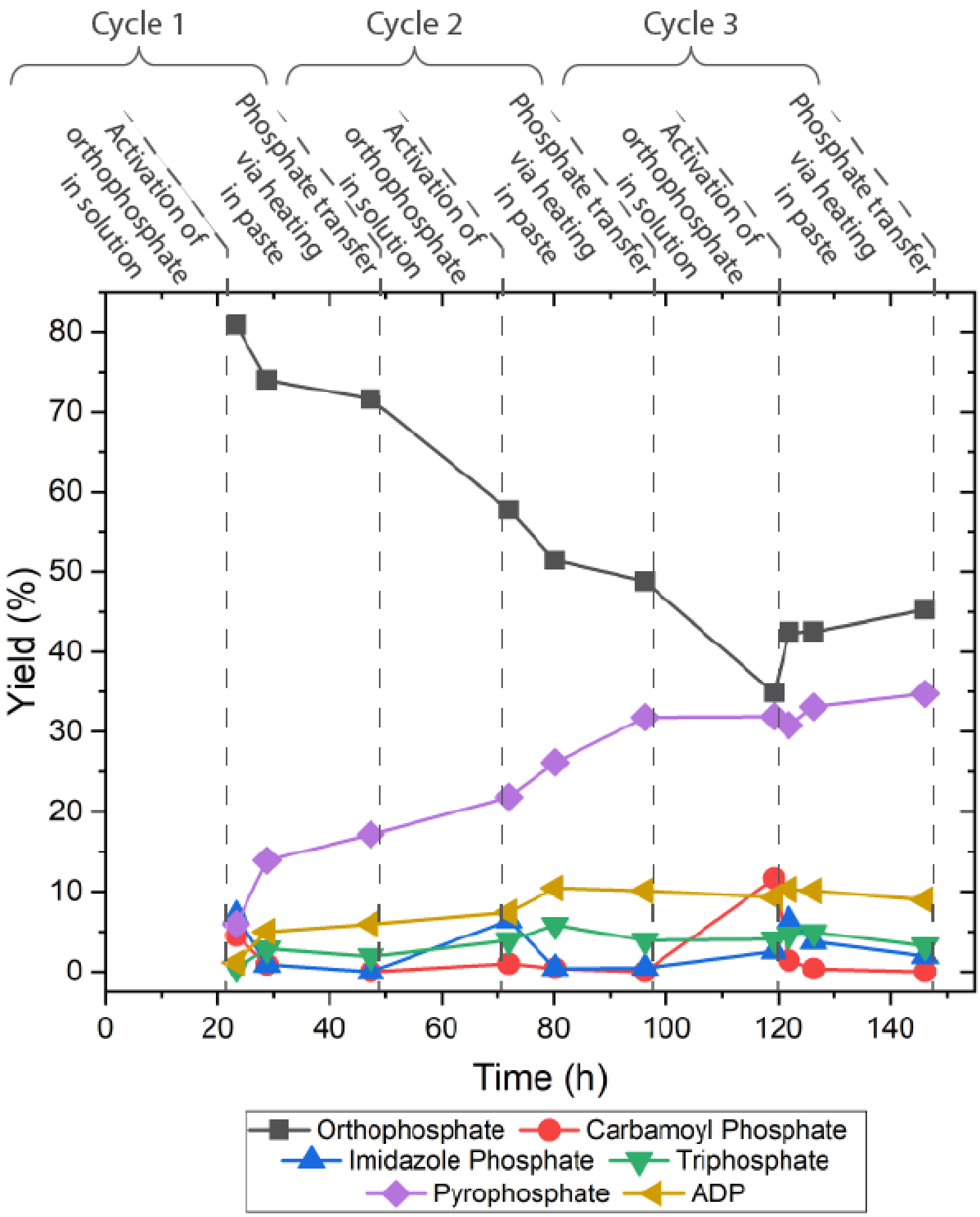

Supplementary Figure 206: Changes in yield for all phosphorous species over the course of three cycles for the reaction with 750 mM adenosine-5'-monophosphate, 450 mM potassium cyanate and 150 mM sodium phosphate in 1.0 M imidazole buffer. A minor amount of Phosphorylation (in summation < 5.5 mM) also occurred at several positions on the ribose ring of AMP and for clarity these have been omitted from this figure.

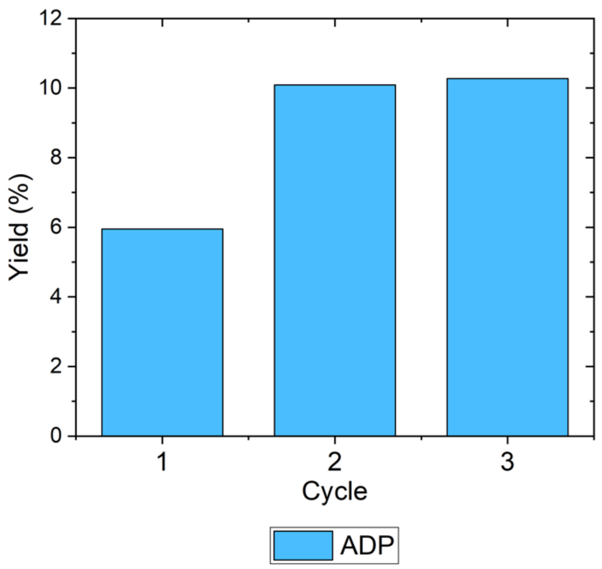

Supplementary Figure 207: Yield of adenosine-5'-diphosphate after each cycle for the reaction with 750 mM adenosine-5'-monophosphate, 450 mM potassium cyanate and 150 mM sodium phosphate in 1.0 M imidazole buffer. Yield at Cycle 3 is after drying to paste and prior to heating which resulted in a marginal loss of ADP.

A spiking experiment with a genuine sample of adenosine-5'-diphosphate confirmed its formation (Supplementary Figure 208).

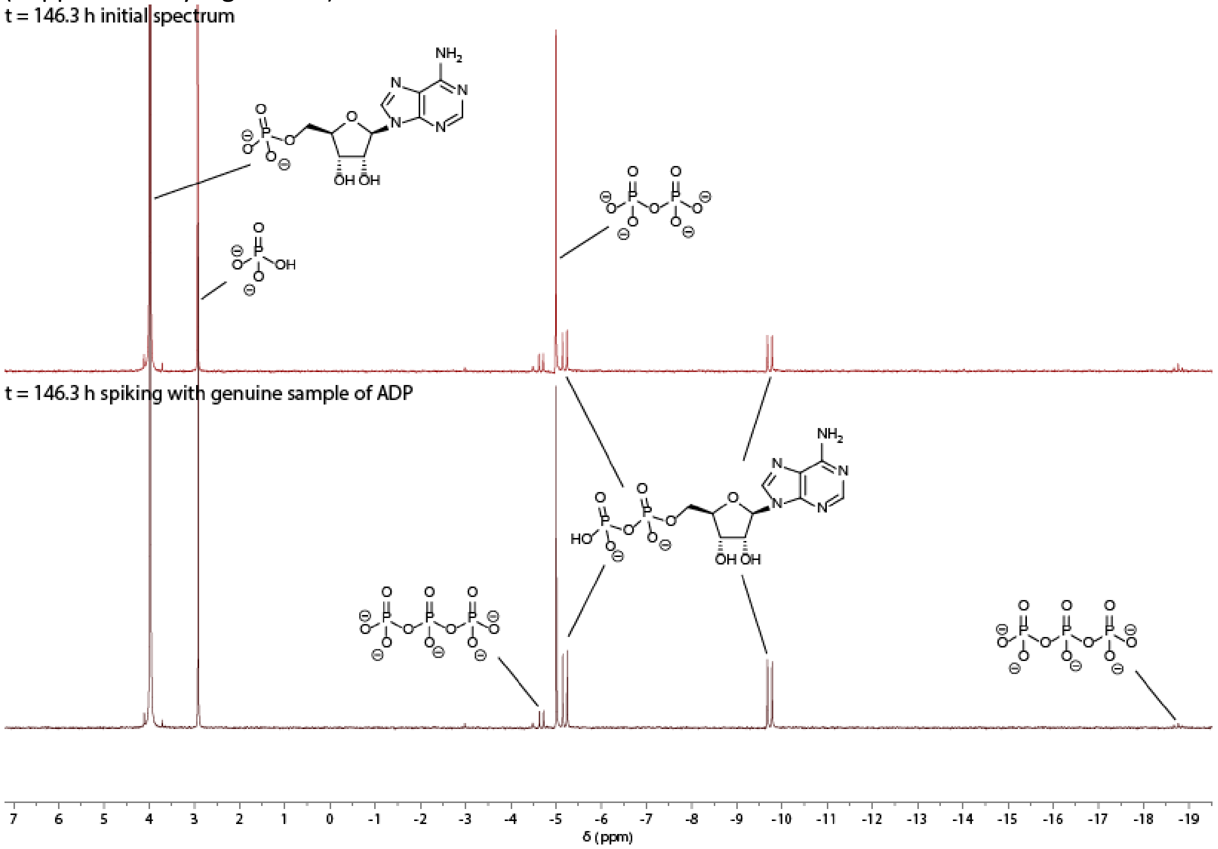

Supplementary Figure 208: ^{31}P NMR spectra of a spiking experiment for the third cycle of the reaction with 750 mM adenosine-5'-monophosphate, 450 mM potassium cyanate and 150 mM sodium phosphate in 1.0 M imidazole buffer. Top spectrum is unspiked. Bottom spectrum is with the addition of genuine adenosine-5'-diphosphate.

S7.4.2 Phosphorylation of adenosine-5'-monophosphate with 150 mM orthophosphate repeat 1

This reaction was performed according to the procedure in Section S7.1 but with 750 mM of adenosine-5'-monophosphate disodium salt (1.096 g, 3.0 mmol).

A total of three cycles were performed. Formation of imidazole phosphate was observed in solution and remained present after drying the solution to a paste. Formation of adenosine-5'-diphosphate occurred during both the drying to the paste over a period of ~24 h and during the heating of the paste to 50 °C for ~24 h (Supplementary Figure 209, 210 and 211).

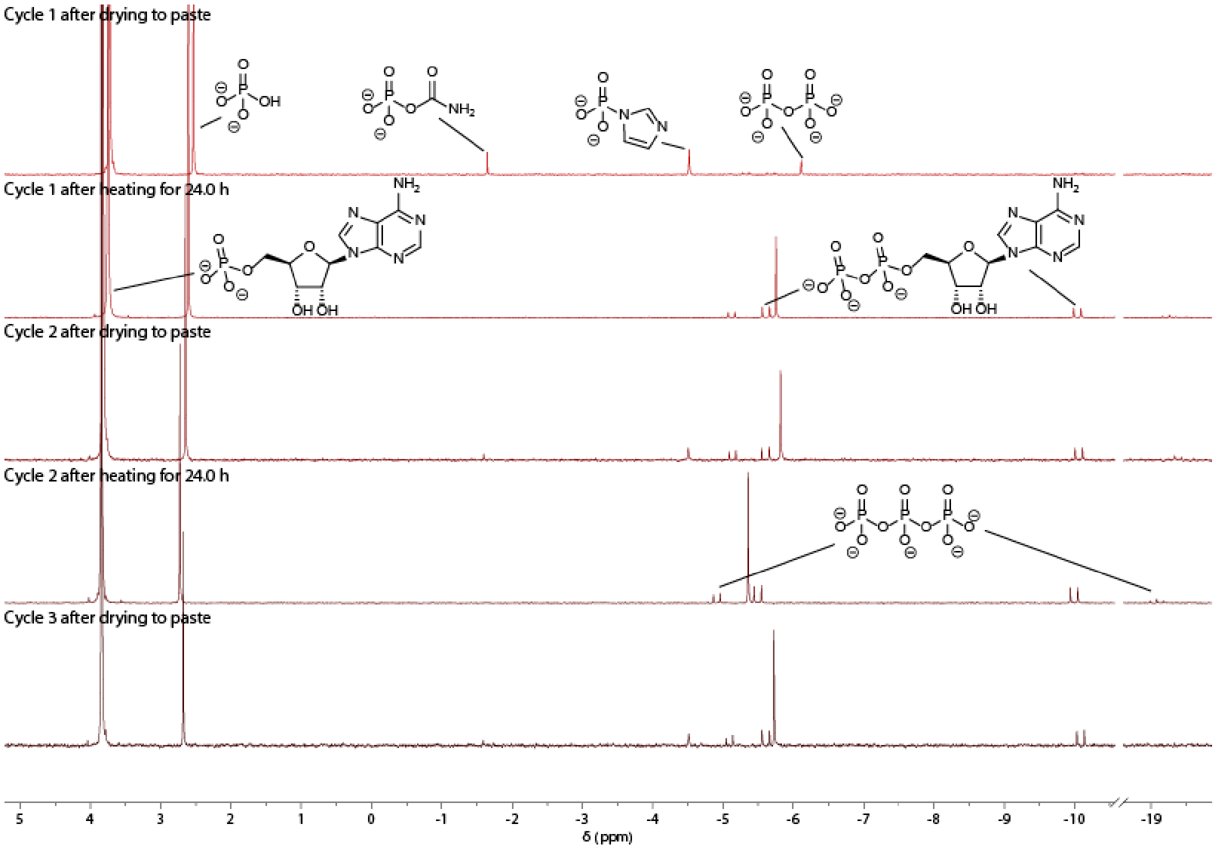

Supplementary Figure 209: Representative ^{31}P NMR spectra for the three cycles with 750 mM adenosine-5'-monophosphate, 450 mM potassium cyanate and 150 mM sodium phosphate in 1.0 M imidazole buffer. For each cycle the ^{31}P NMR spectrum after the reaction had been dried to a paste and the ^{31}P NMR spectrum after heating the reaction to 50 °C for ~24 h are shown. All NMR spectra were measured in a 0.5 M citric acid buffer at pH 6.85 9 : 1 H_2O : D_2O. The chemical shift of the pyrophosphate peak is variable at pH 6.85 due to the pyrophosphate pK_a = 6.6.

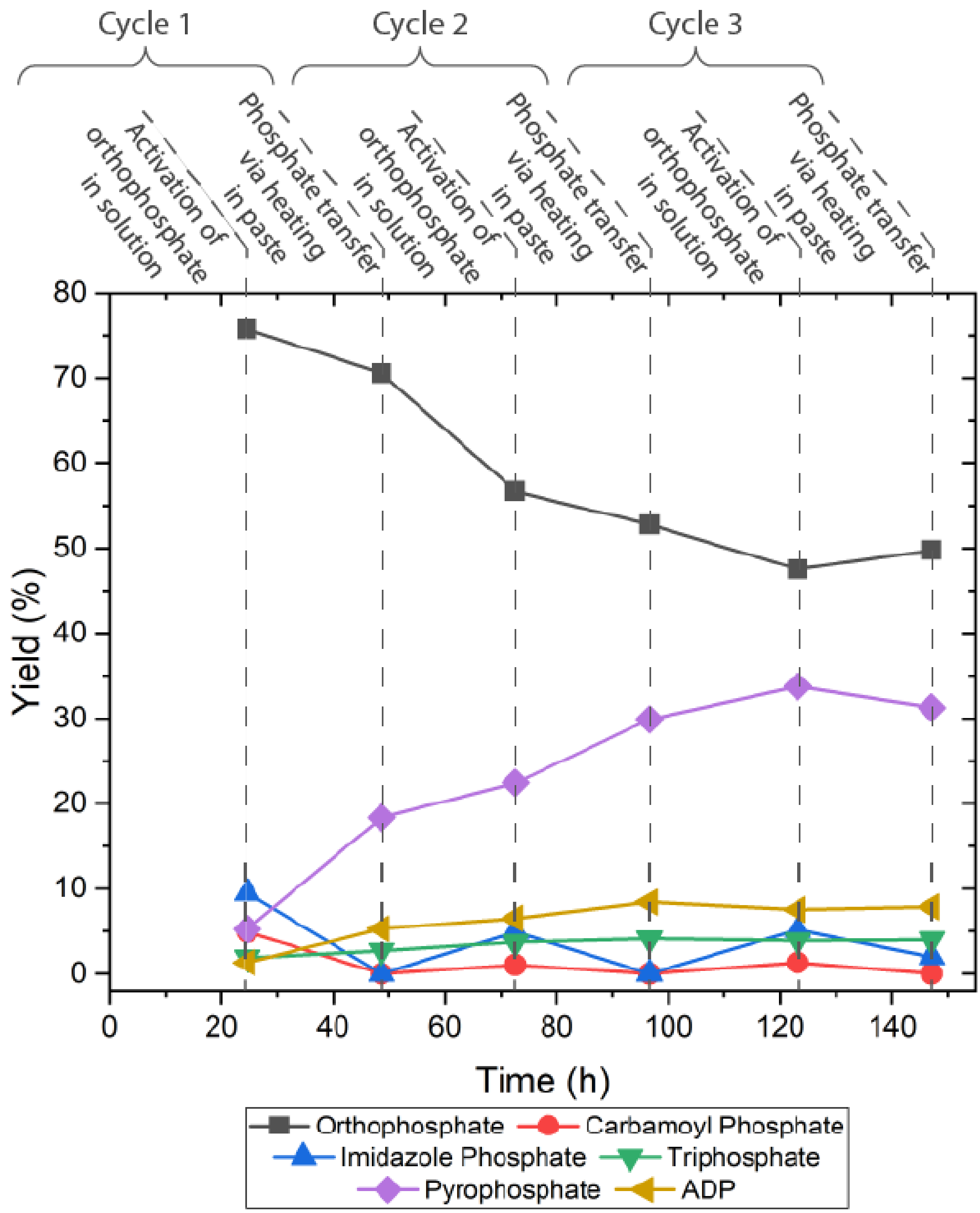

Supplementary Figure 210: Changes in yield for all phosphorous species over the course of three cycles for the reaction with 750 mM adenosine-5'-monophosphate, 450 mM potassium cyanate and 150 mM sodium phosphate in 1.0 M imidazole buffer. A minor amount of phosphorylation (in summation < 5.5 mM) also occurred at several positions on the ribose ring of AMP and for clarity these have been omitted from this figure.

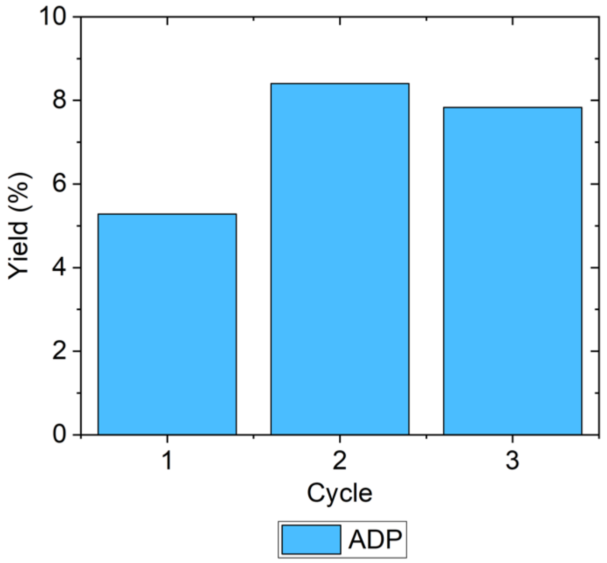

Supplementary Figure 211: Yield of adenosine-5'-diphosphate after each cycle for the reaction with 750 mM adenosine-5'-monophosphate, 450 mM potassium cyanate and 150 mM sodium phosphate in 1.0 M imidazole buffer. Yield at Cycle 3 is after drying to paste and prior to heating which resulted in a marginal loss of ADP.

S7.4.3 Phosphorylation of adenosine-5'-monophosphate with 150 mM orthophosphate repeat 2

This reaction was performed according to the procedure in Section S7.1 but with 750 mM of adenosine-5'-monophosphate disodium salt (1.096 g, 3.0 mmol).

A total of three cycles were performed. Formation of imidazole phosphate was observed in solution and remained present after drying the solution to a paste. Formation of adenosine-5'-diphosphate occurred during both the drying to the paste over a period of ~24 h and during the heating of the paste to 50 °C for ~24 h (Supplementary Figure 212, 213 and 214).

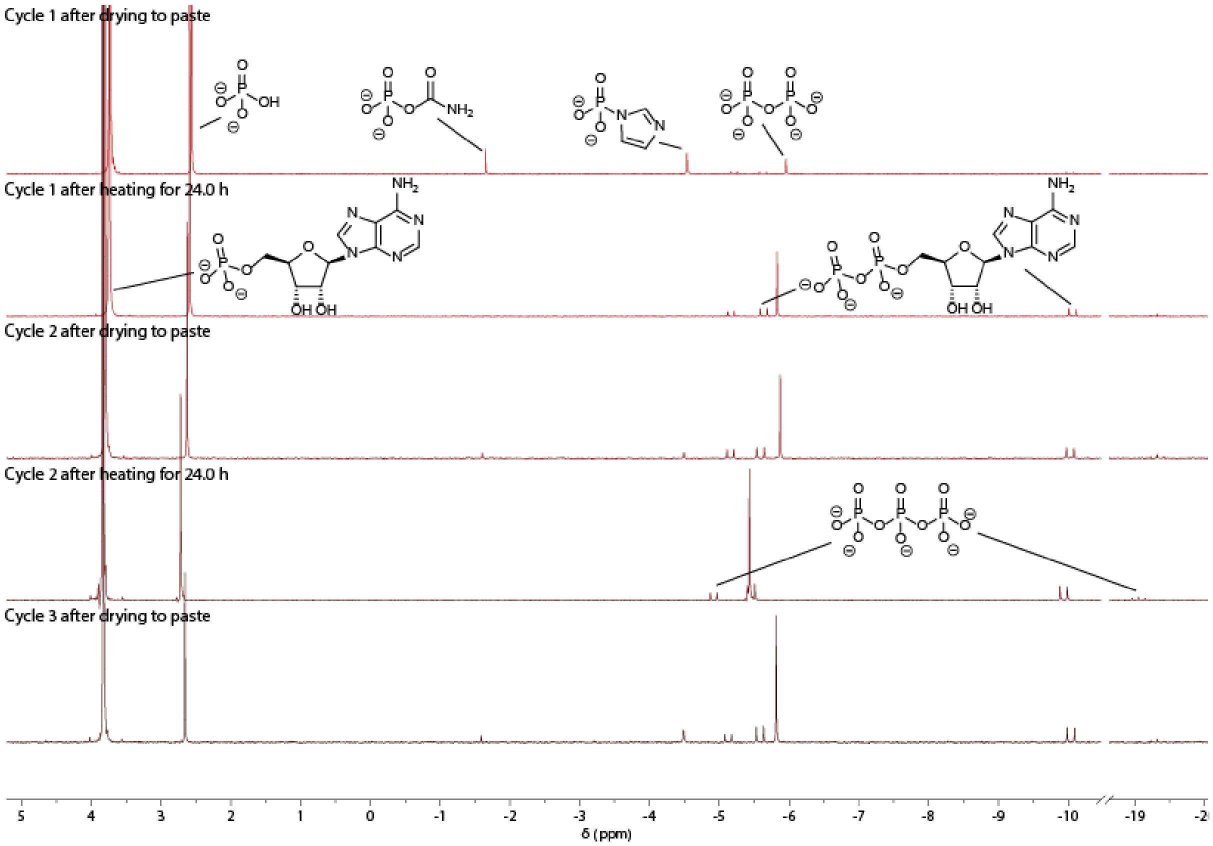

Supplementary Figure 212: Representative ^{31}P NMR spectra for the three cycles with 750 mM adenosine-5’-monophosphate, 450 mM potassium cyanate and 150 mM sodium phosphate in 1.0 M imidazole buffer. For each cycle the ^{31}P NMR spectrum after the reaction had been dried to a paste and the ^{31}P NMR spectrum after heating the reaction to 50 °C for ~24 h are shown. All NMR spectra were measured in a 0.5 M citric acid buffer at pH 6.85 9 : 1 H_2O : D_2O. The chemical shift of the pyrophosphate peak is variable at pH 6.85 due to the pyrophosphate pK_a = 6.6.

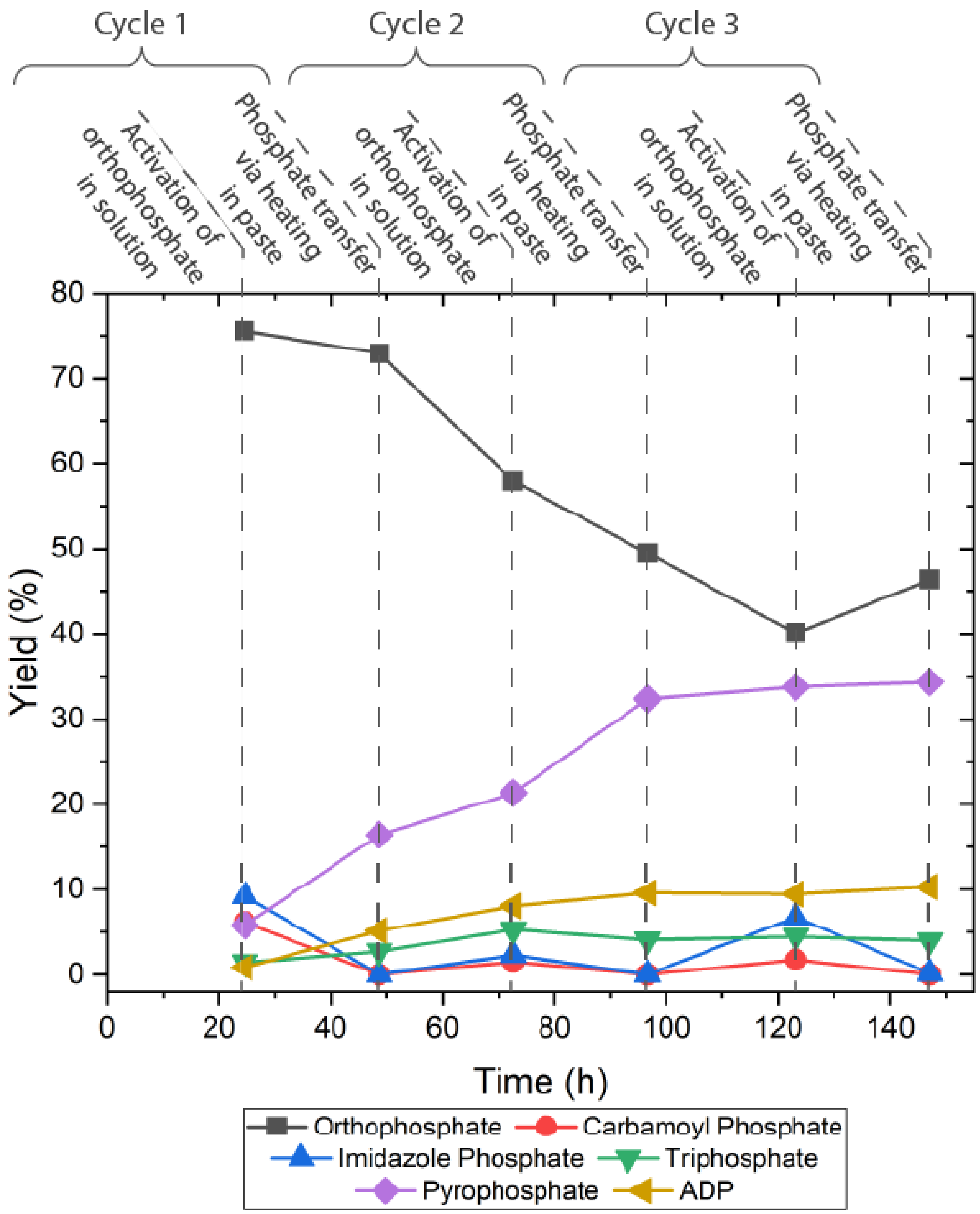

Supplementary Figure 213: Changes in yield for all phosphorous species over the course of three cycles for the reaction with 750 mM adenosine-5'-monophosphate, 450 mM potassium cyanate and 150 mM sodium phosphate in 1.0 M imidazole buffer. A minor amount of phosphorylation (in summation < 5.5 mM) also occurred at several positions on the ribose ring of AMP and for clarity these have been omitted from this figure.

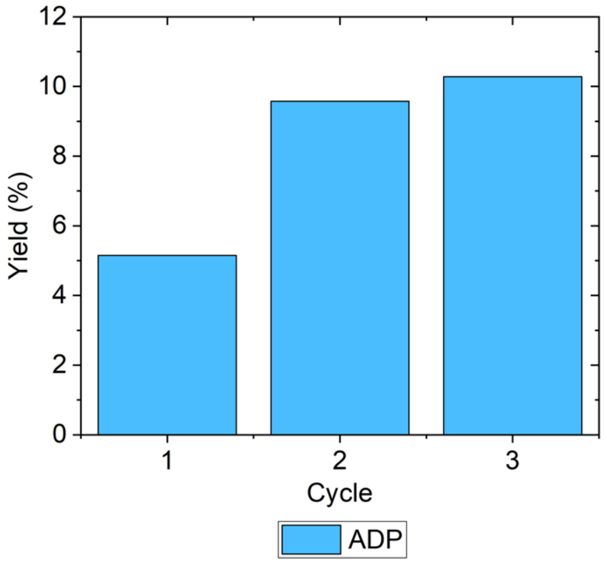

Supplementary Figure 214: Yield of adenosine-5'-diphosphate after each cycle for the reaction with 750 mM adenosine-5'-monophosphate, 450 mM potassium cyanate and 150 mM sodium phosphate in 1.0 M imidazole buffer. Yield at Cycle 3 is after drying to paste and prior to heating which resulted in a marginal loss of ADP.

S7.4.4 Phosphorylation of adenosine-5'-monophosphate with 50 mM orthophosphate

This reaction was performed according to the procedure in Section S7.1 but with 750 mM of adenosine-5'-monophosphate disodium salt (1.096 g, 3.0 mmol) and 50 mM sodium phosphate dibasic dihydrate salt (178.0 mg, 1.0 mmol).

A total of three cycles were performed. Formation of imidazole phosphate was observed in solution and remained present after drying the solution to a paste. Formation of adenosine-5'-diphosphate occurred during both the drying to the paste over a period of ~24 h and during the heating of the paste to 50 °C for ~24 h (Supplementary Figure 215, 216 and 217).

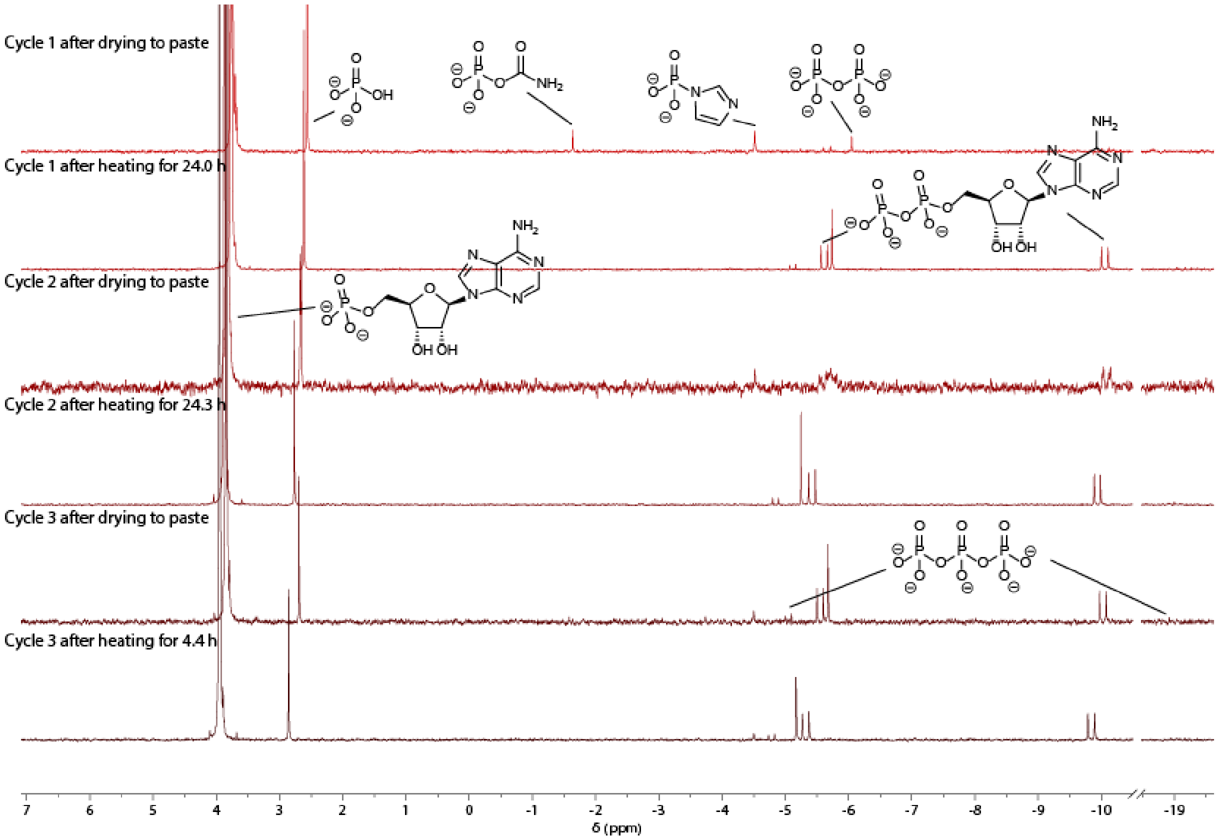

Supplementary Figure 215: Representative ^{31}P NMR spectra for the three cycles with 750 mM adenosine-5′-monophosphate, 450 mM potassium cyanate and 50 mM sodium phosphate in 1.0 M imidazole buffer. For each cycle the ^{31}P NMR spectrum after the reaction had been dried to a paste and the ^{31}P NMR spectrum after heating the reaction to 50 °C for ~24 h are shown. All NMR spectra were measured in a 0.5 M citric acid buffer at pH 6.85 9 : 1 H_2O : D_2O. The chemical shift of the pyrophosphate peak is variable at pH 6.85 due to the pyrophosphate pK_a = 6.6.

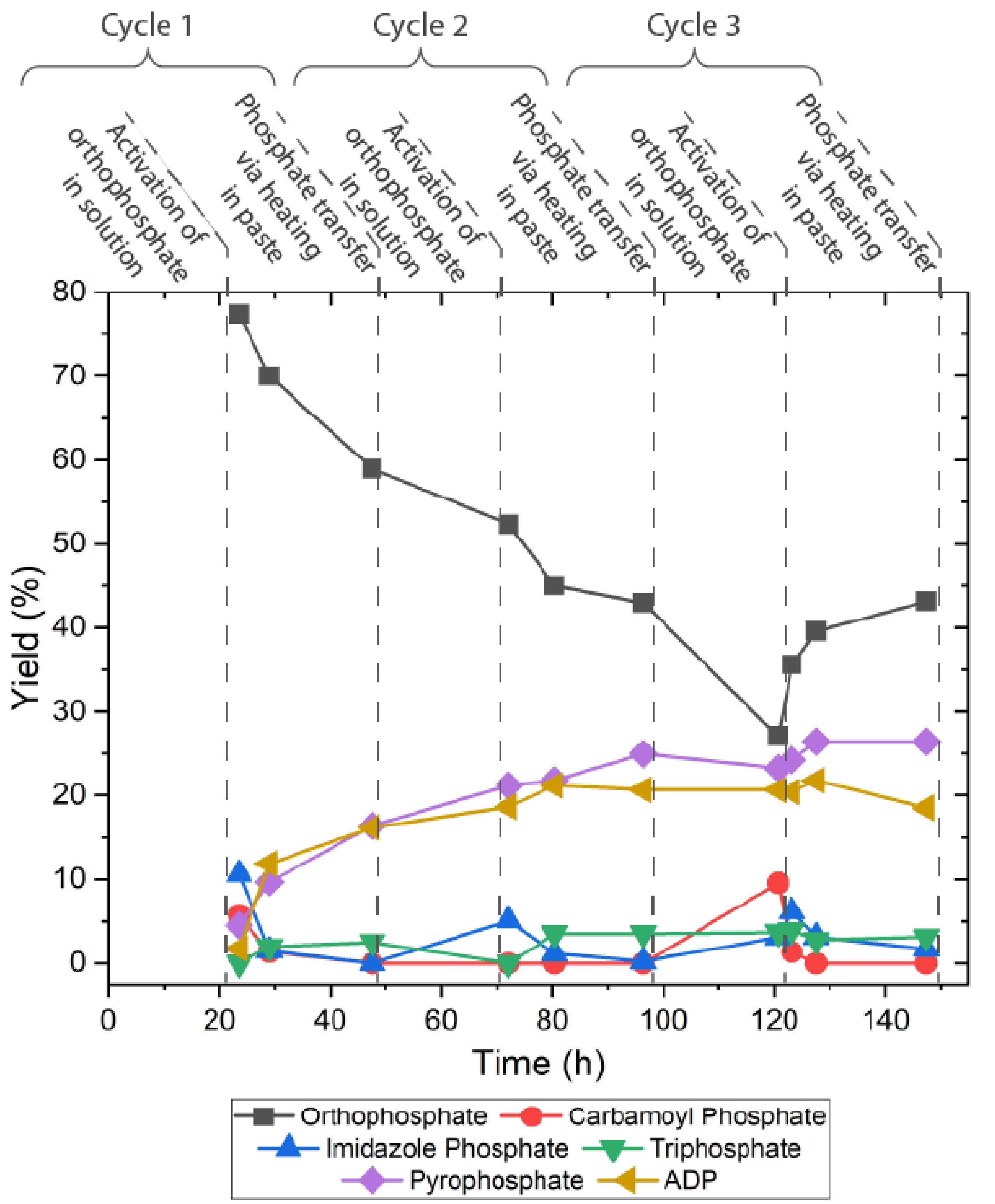

Supplementary Figure 216: Changes in yield for all phosphorous species over the course of three cycles for the reaction with 750 mM adenosine-5'-monophosphate, 450 mM potassium cyanate and 50 mM sodium phosphate in 1.0 M imidazole buffer. A minor amount of phosphorylation (in summation < 3.5 mM) also occurred at several positions on the ribose ring of AMP and for clarity these have been omitted from this figure.

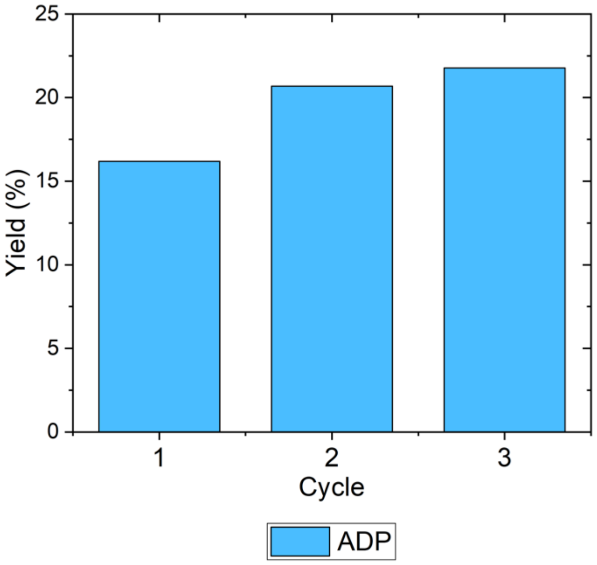

Supplementary Figure 217: Yield of adenosine-5'-diphosphate after each cycle for the reaction with 750 mM adenosine-5'-monophosphate, 450 mM potassium cyanate and 50 mM sodium phosphate in 1.0 M imidazole buffer. Yield at Cycle 3 is after drying to paste and prior to heating which resulted in a marginal loss of ADP.

A spiking experiment with a genuine sample of adenosine-5'-diphosphate confirmed its formation (Supplementary Figure 218).

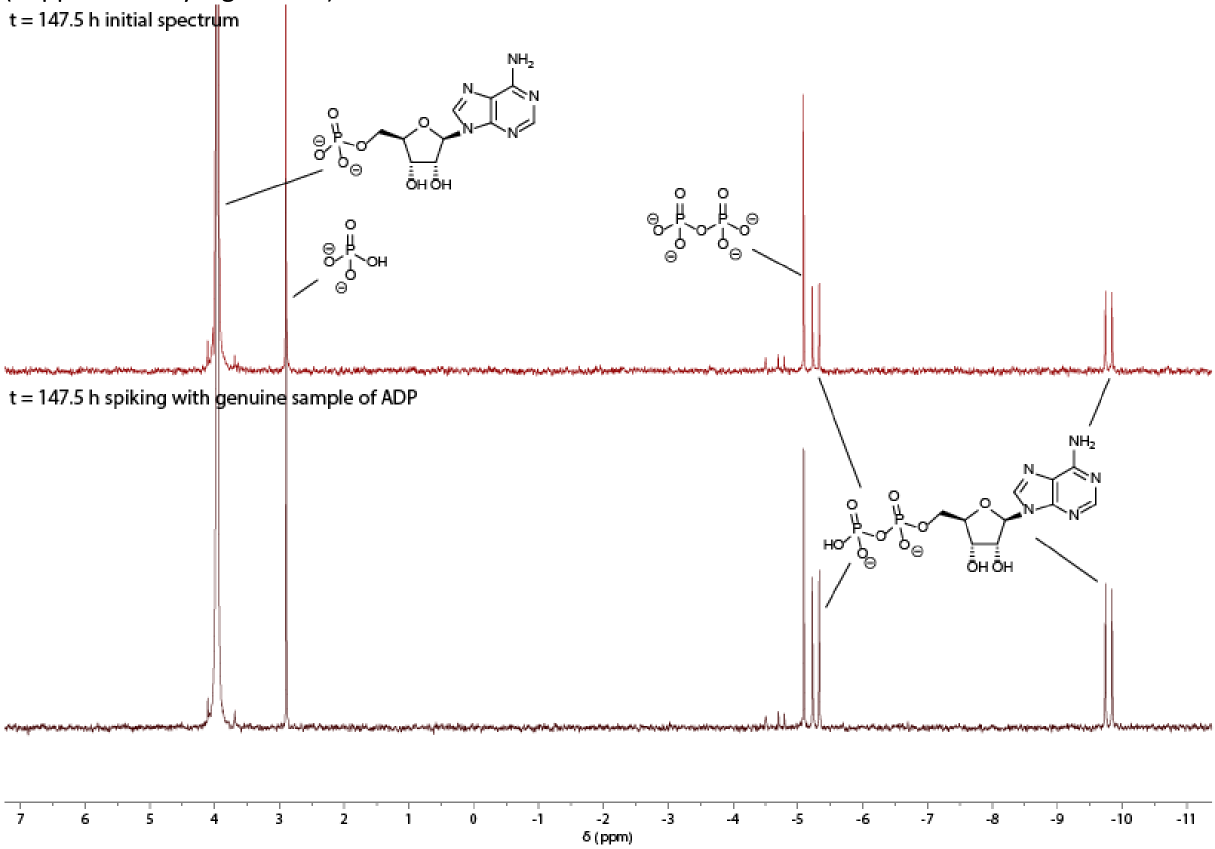

Supplementary Figure 218: ^{31}P NMR spectra of a spiking experiment for the third cycle of the reaction with 750 mM adenosine-5'-monophosphate, 450 mM potassium cyanate and 50 mM sodium phosphate in 1.0 M imidazole buffer. Top spectrum is unspiked. Bottom spectrum is with the addition of genuine adenosine-5'-diphosphate.

S7.4.5 Phosphorylation of adenosine-5'-monophosphate with 50 mM orthophosphate repeat 1

This reaction was performed according to the procedure in Section S7.1 but with 750 mM of adenosine-5'-monophosphate disodium salt (1.096 g, 3.0 mmol) and 50 mM sodium phosphate dibasic dihydrate salt (178.0 mg, 1.0 mmol).

A total of three cycles were performed. Formation of imidazole phosphate was observed in solution and remained present after drying the solution to a paste. Formation of adenosine-5'-diphosphate occurred during both the drying to the paste over a period of ~24 h and during the heating of the paste to 50 °C for ~24 h (Supplementary Figure 219, 220 and 221).

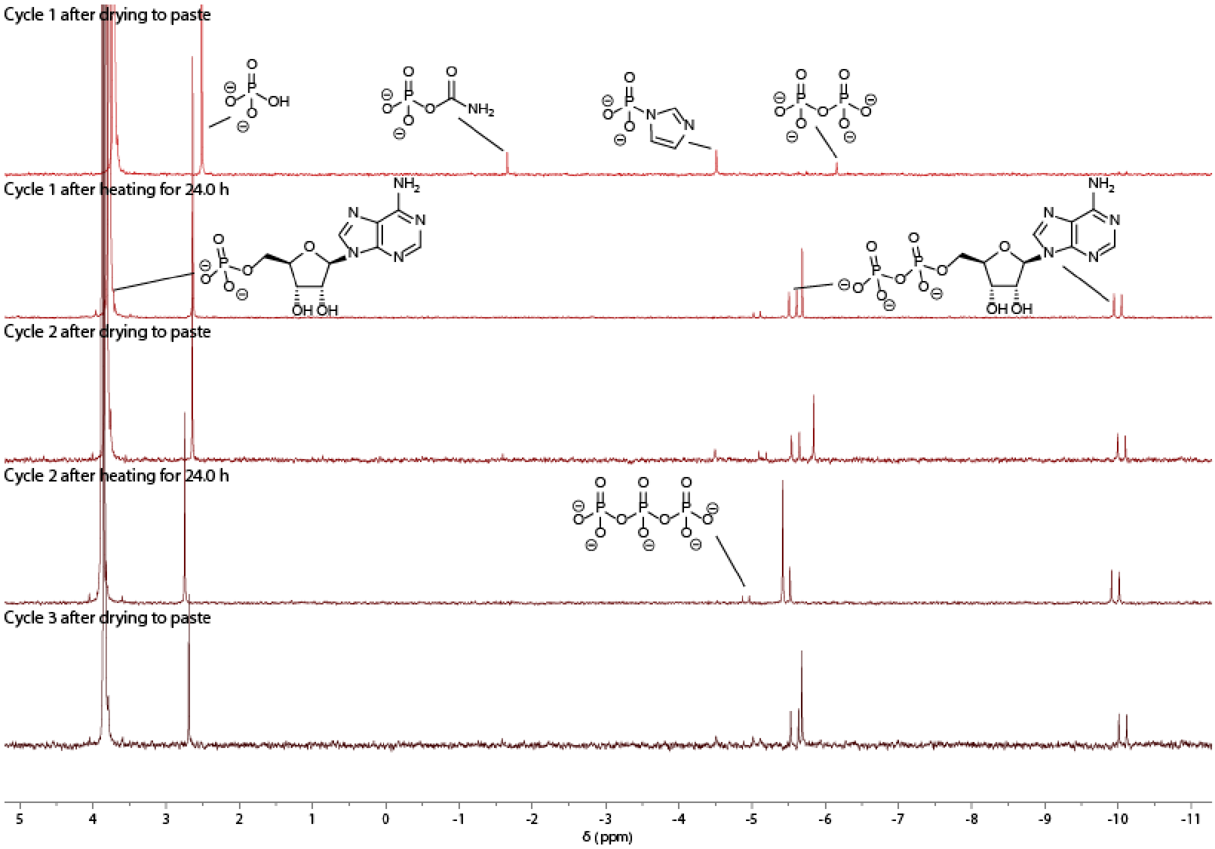

Supplementary Figure 219: Representative ^{31}P NMR spectra for the three cycles with 750 mM adenosine-5'-monophosphate, 450 mM potassium cyanate and 50 mM sodium phosphate in 1.0 M imidazole buffer. For each cycle the ^{31}P NMR spectrum after the reaction had been dried to a paste and the ^{31}P NMR spectrum after heating the reaction to 50 °C for ~24 h are shown. All NMR spectra were measured in a 0.5 M citric acid buffer at pH 6.85 9 : 1 H_2O : D_2O. The chemical shift of the pyrophosphate peak is variable at pH 6.85 due to the pyrophosphate pK_a = 6.6.

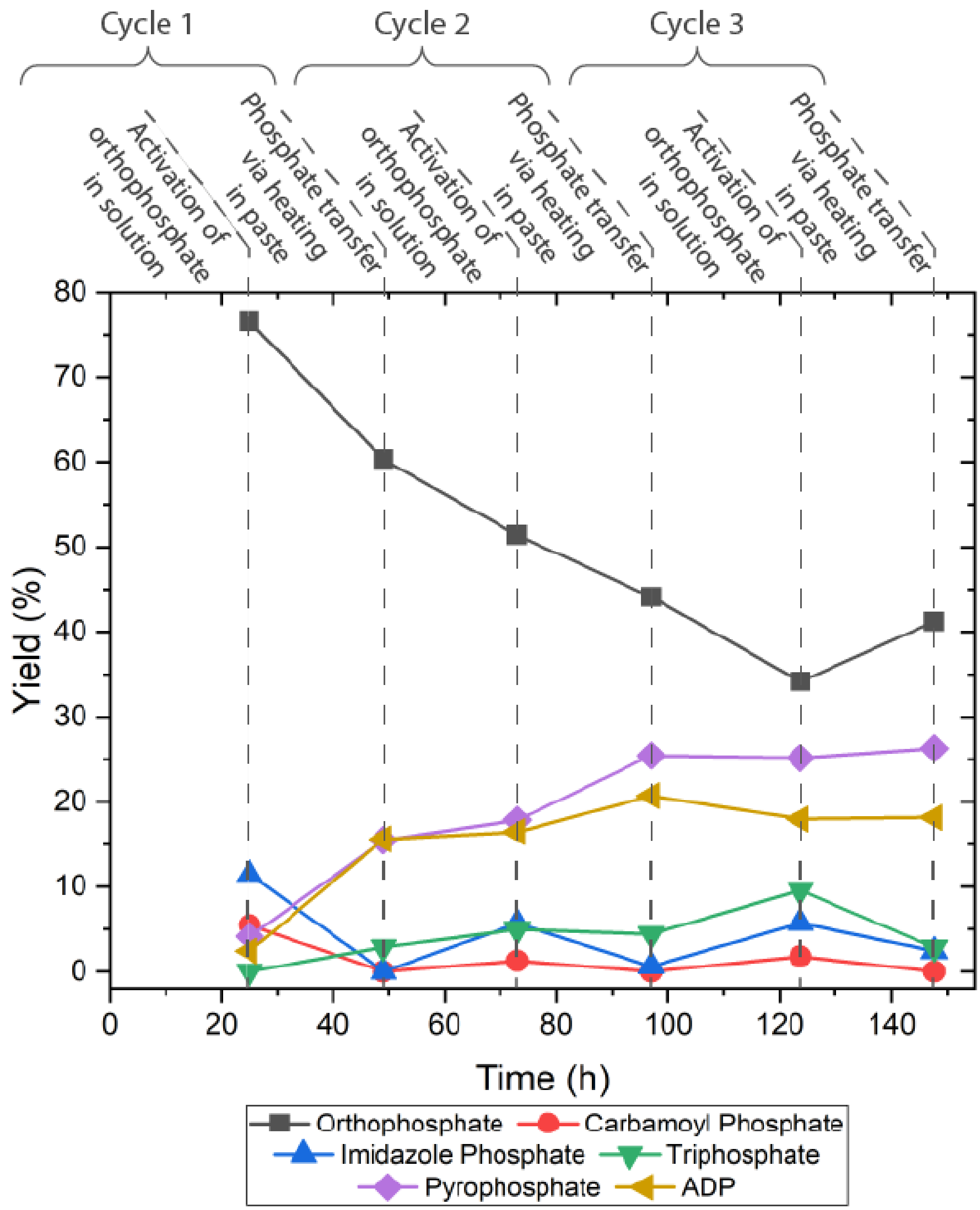

Supplementary Figure 220: Changes in yield for all phosphorous species over the course of three cycles for the reaction with 750 mM adenosine-5'-monophosphate, 450 mM potassium cyanate and 50 mM sodium phosphate in 1.0 M imidazole buffer. A minor amount of phosphorylation (in summation < 5.5 mM) also occurred at several positions on the ribose ring of AMP and for clarity these have been omitted from this figure.

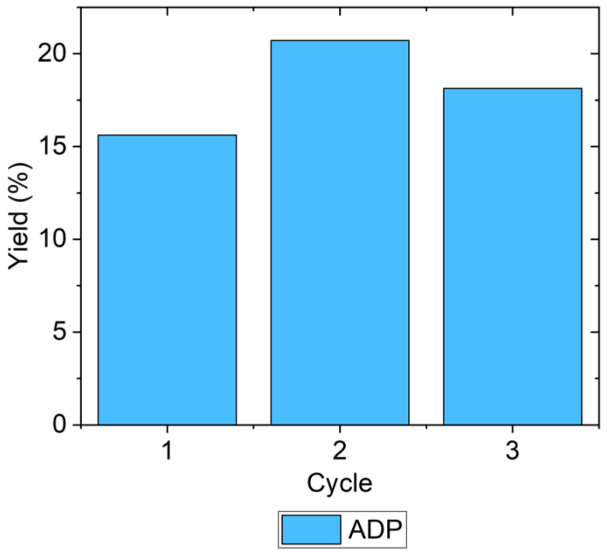

Supplementary Figure 221: Yield of adenosine-5'-diphosphate after each cycle for the reaction with 750 mM adenosine-5'-monophosphate, 450 mM potassium cyanate and 50 mM sodium phosphate in 1.0 M imidazole buffer. Yield at Cycle 3 is after drying to paste and prior to heating which resulted in a marginal loss of ADP.

S7.4.6 Phosphorylation of adenosine-5'-monophosphate with 50 mM orthophosphate repeat 2

This reaction was performed according to the procedure in Section S7.1 but with 750 mM of adenosine-5'-monophosphate disodium salt (1.096 g, 3.0 mmol) and 50 mM sodium phosphate dibasic dihydrate salt (178.0 mg, 1.0 mmol).

A total of three cycles were performed. Formation of imidazole phosphate was observed in solution and remained present after drying the solution to a paste. Formation of adenosine-5'-diphosphate occurred during both the drying to the paste over a period of ~24 h and during the heating of the paste to 50 °C for ~24 h (Supplementary Figure 222, 223 and 224).

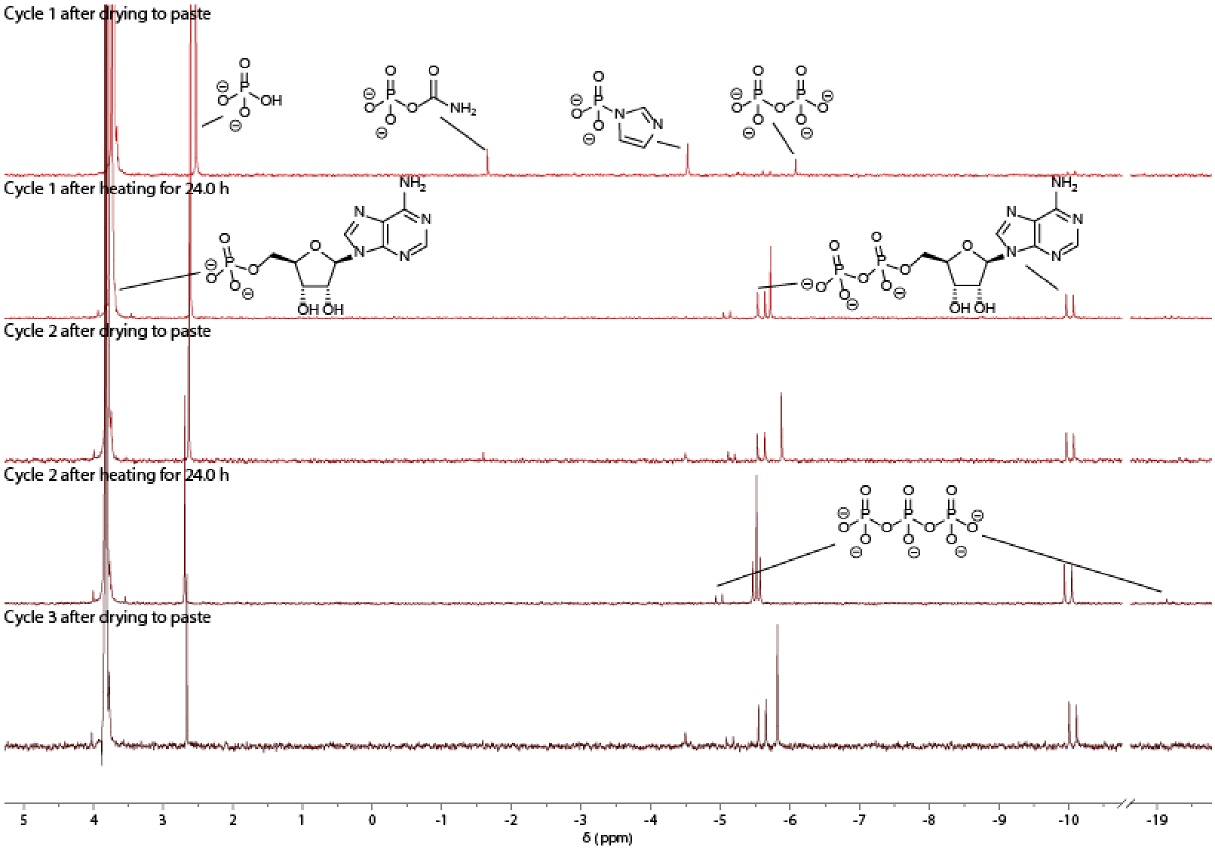

Supplementary Figure 222: Representative ^{31}P NMR spectra for the three cycles with 750 mM adenosine-5'-monophosphate, 450 mM potassium cyanate and 50 mM sodium phosphate in 1.0 M imidazole buffer. For each cycle the ^{31}P NMR spectrum after the reaction had been dried to a paste and the ^{31}P NMR spectrum after heating the reaction to 50 °C for ~24 h are shown. All NMR spectra were measured in a 0.5 M citric acid buffer at pH 6.85 9 : 1 H_2O : D_2O. The chemical shift of the pyrophosphate peak is variable at pH 6.85 due to the pyrophosphate pK_a = 6.6.

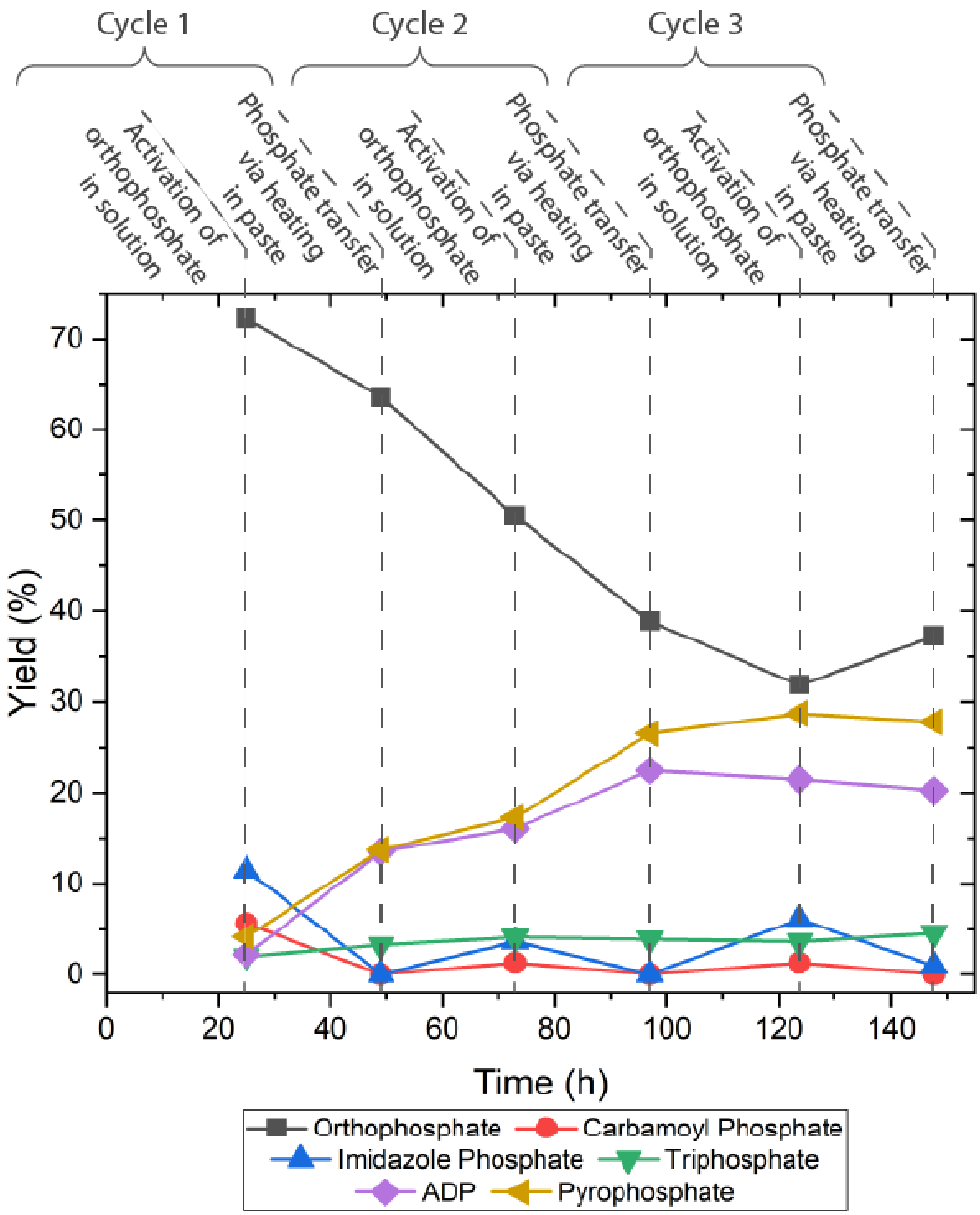

Supplementary Figure 223: Changes in yield for all phosphorous species over the course of three cycles for the reaction with 750 mM adenosine-5'-monophosphate, 450 mM potassium cyanate and 50 mM sodium phosphate in 1.0 M imidazole buffer. A minor amount of phosphorylation (in summation < 5.5 mM) also occurred at several positions on the ribose ring of AMP and for clarity these have been omitted from this figure.

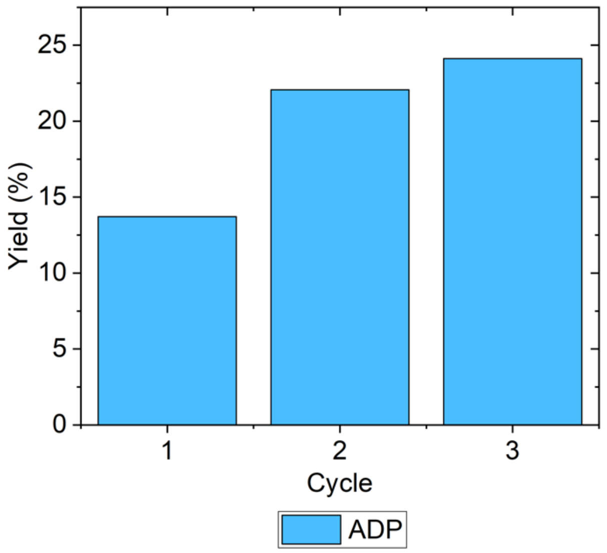

Supplementary Figure 224: Yield of adenosine-5'-diphosphate after each cycle for the reaction with 750 mM adenosine-5'-monophosphate, 450 mM potassium cyanate and 50 mM sodium phosphate in 1.0 M imidazole buffer. Yield at Cycle 3 is after drying to paste and prior to heating which resulted in a marginal loss of ADP.

S7.4.7 Phosphorylation of adenosine-5'-monophosphate with 20 mM orthophosphate

This reaction was performed according to the procedure in Section S7.1 but with 750 mM of adenosine-5'-monophosphate disodium salt (1.096 g, 3.0 mmol) and 20 mM sodium phosphate dibasic dihydrate salt (71.2 mg, 0.4 mmol).

A total of three cycles were performed. Formation of imidazole phosphate was observed in solution and remained present after drying the solution to a paste. Formation of adenosine-5'-diphosphate occurred during both the drying to the paste over a period of ~24 h and during the heating of the paste to 50 °C for ~24 h (Supplementary Figure 225, 226 and 227).

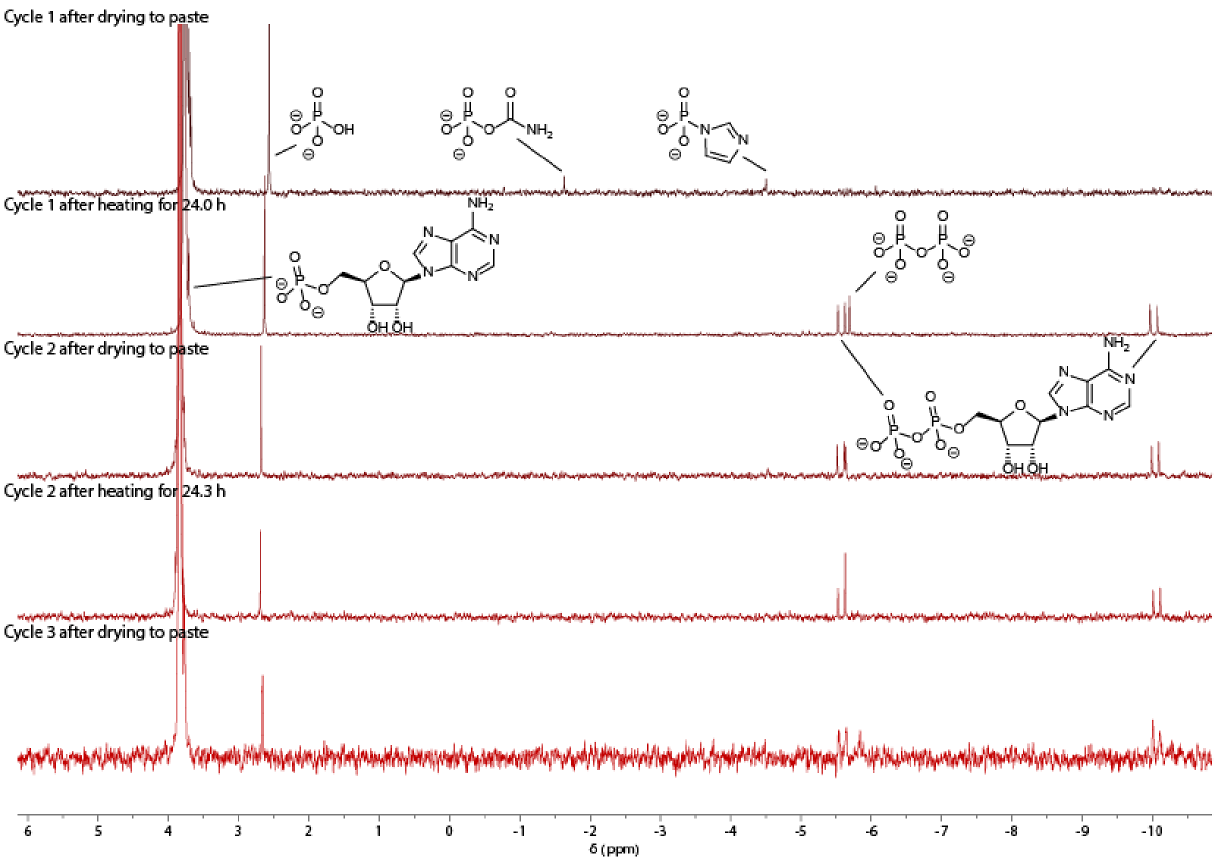

Supplementary Figure 225: Representative ^{31}P NMR spectra for the three cycles with 750 mM adenosine-5’-monophosphate, 450 mM potassium cyanate and 20 mM sodium phosphate in 1.0 M imidazole buffer. For each cycle the ^{31}P NMR spectrum after the reaction had been dried to a paste and the ^{31}P NMR spectrum after heating the reaction to 50 °C for ~24 h are shown.

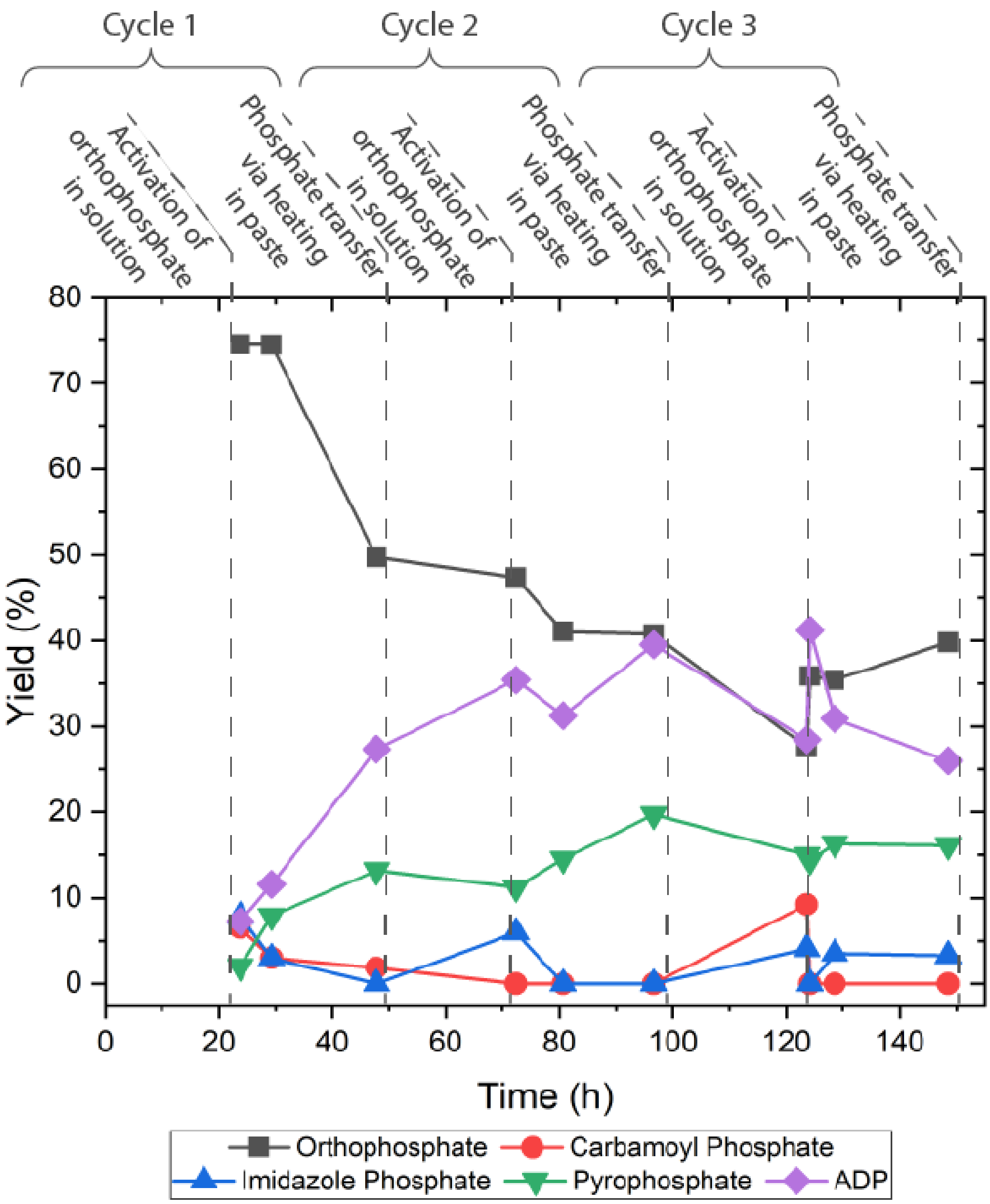

Supplementary Figure 226: Changes in yield for all phosphorous species over the course of three cycles for the reaction with 750 mM adenosine-5'-monophosphate, 450 mM potassium cyanate and 20 mM sodium phosphate in 1.0 M imidazole buffer. A minor amount of phosphorylation (in summation < 2.5 mM) also occurred at several positions on the ribose ring of AMP and for clarity these have been omitted from this figure.

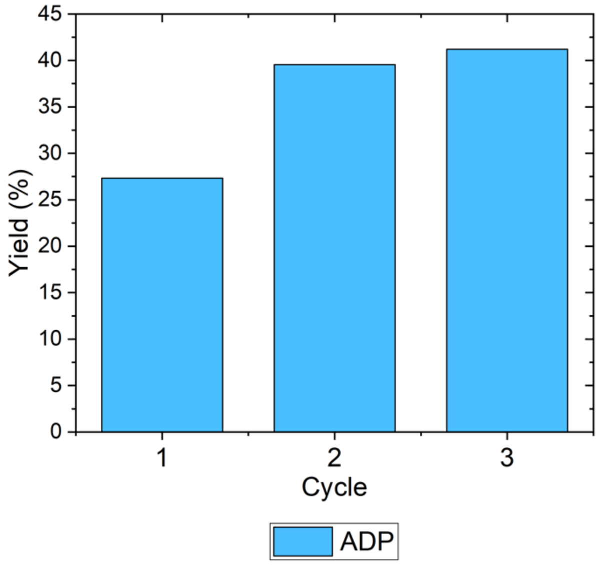

Supplementary Figure 227: Yield of adenosine-5’-diphosphate after each cycle for the reaction with 750 mM adenosine-5’-monophosphate, 450 mM potassium cyanate and 20 mM sodium phosphate in 1.0 M imidazole buffer. Yield at Cycle 3 is after drying to paste and prior to heating which resulted in a marginal loss of ADP.

A spiking experiment with a genuine sample of adenosine-5’-diphosphate confirmed its formation (Supplementary Figure 228).

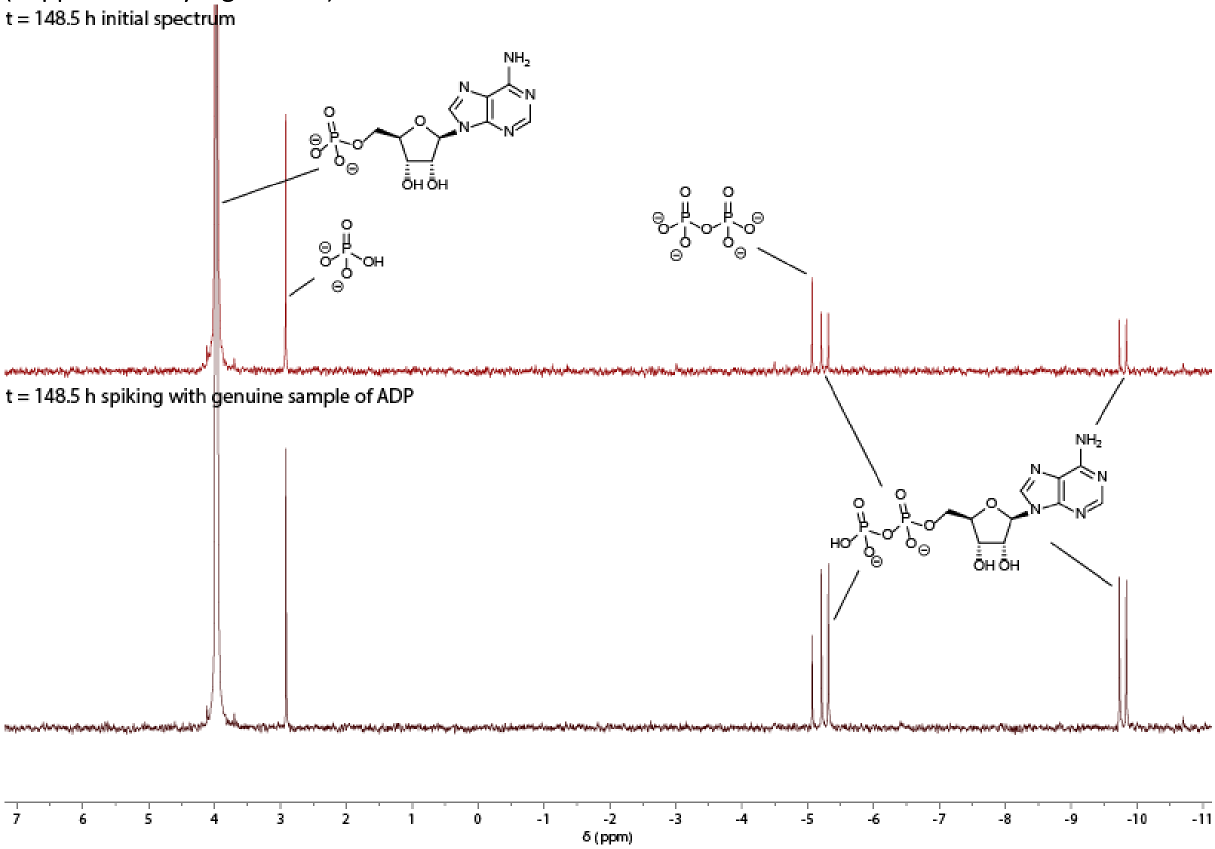

Supplementary Figure 228: ^{31}P NMR spectra of a spiking experiment for the third cycle of the reaction with 750 mM adenosine-5’-monophosphate, 450 mM potassium cyanate and 20 mM sodium phosphate in 1.0 M imidazole buffer. Top spectrum is unspiked. Bottom spectrum is with the addition of genuine adenosine-5’-diphosphate.

S7.4.8 Phosphorylation of adenosine-5'-monophosphate with 20 mM orthophosphate repeat 1

This reaction was performed according to the procedure in Section S7.1 but with 750 mM of adenosine-5'-monophosphate disodium salt (1.096 g, 3.0 mmol) and 20 mM sodium phosphate dibasic dihydrate salt (71.2 mg, 0.4 mmol).

A total of three cycles were performed. Formation of imidazole phosphate was observed in solution and remained present after drying the solution to a paste. Formation of adenosine-5'-diphosphate occurred during both the drying to the paste over a period of ~24 h and during the heating of the paste to 50 °C for ~24 h (Supplementary Figure 229, 230 and 231).

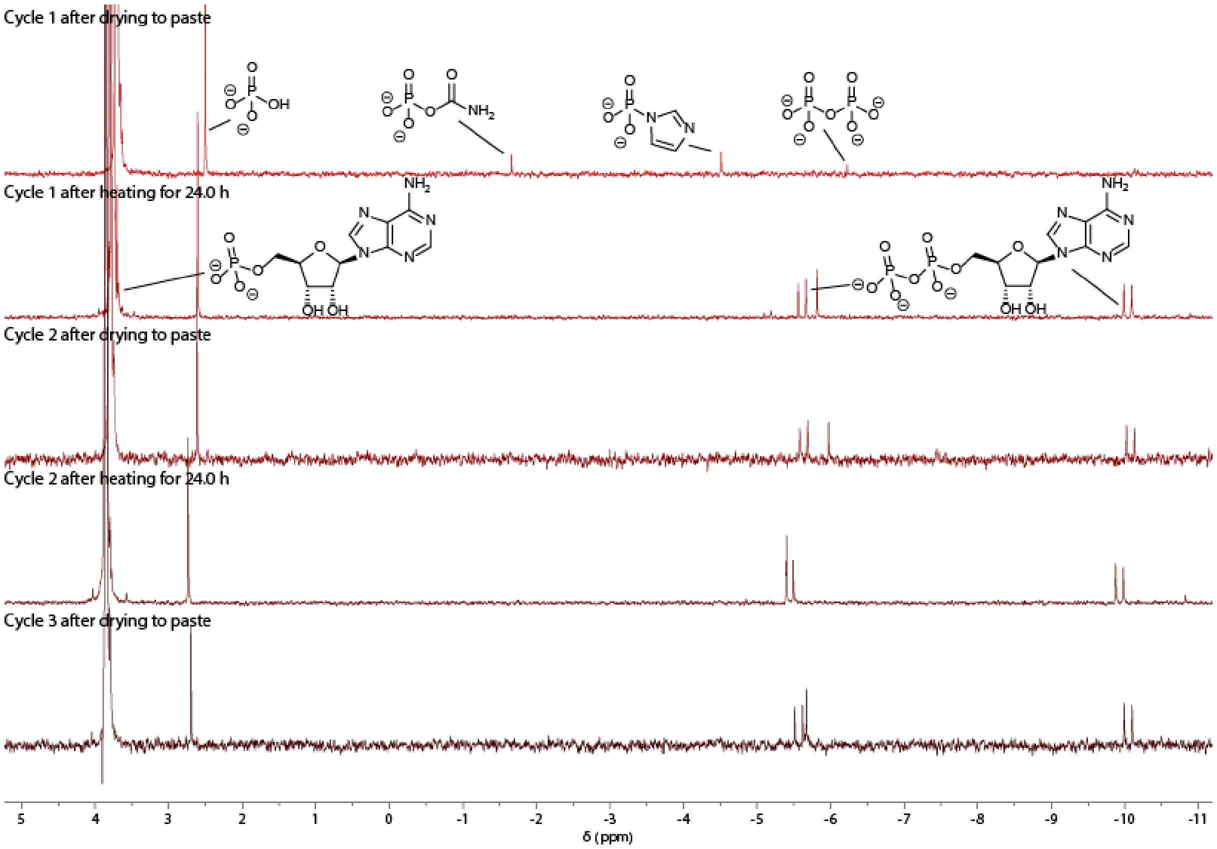

Supplementary Figure 229: Representative ^{31}P NMR spectra for the three cycles with 750 mM adenosine-5'-monophosphate, 450 mM potassium cyanate and 20 mM sodium phosphate in 1.0 M imidazole buffer. For each cycle the ^{31}P NMR spectrum after the reaction had been dried to a paste and the ^{31}P NMR spectrum after heating the reaction to 50 °C for ~24 h are shown. All NMR spectra were measured in a 0.5 M citric acid buffer at pH 6.85 9 : 1 H_2O : D_2O. The chemical shift of the pyrophosphate peak is variable at pH 6.85 due to the pyrophosphate pK_a = 6.6.

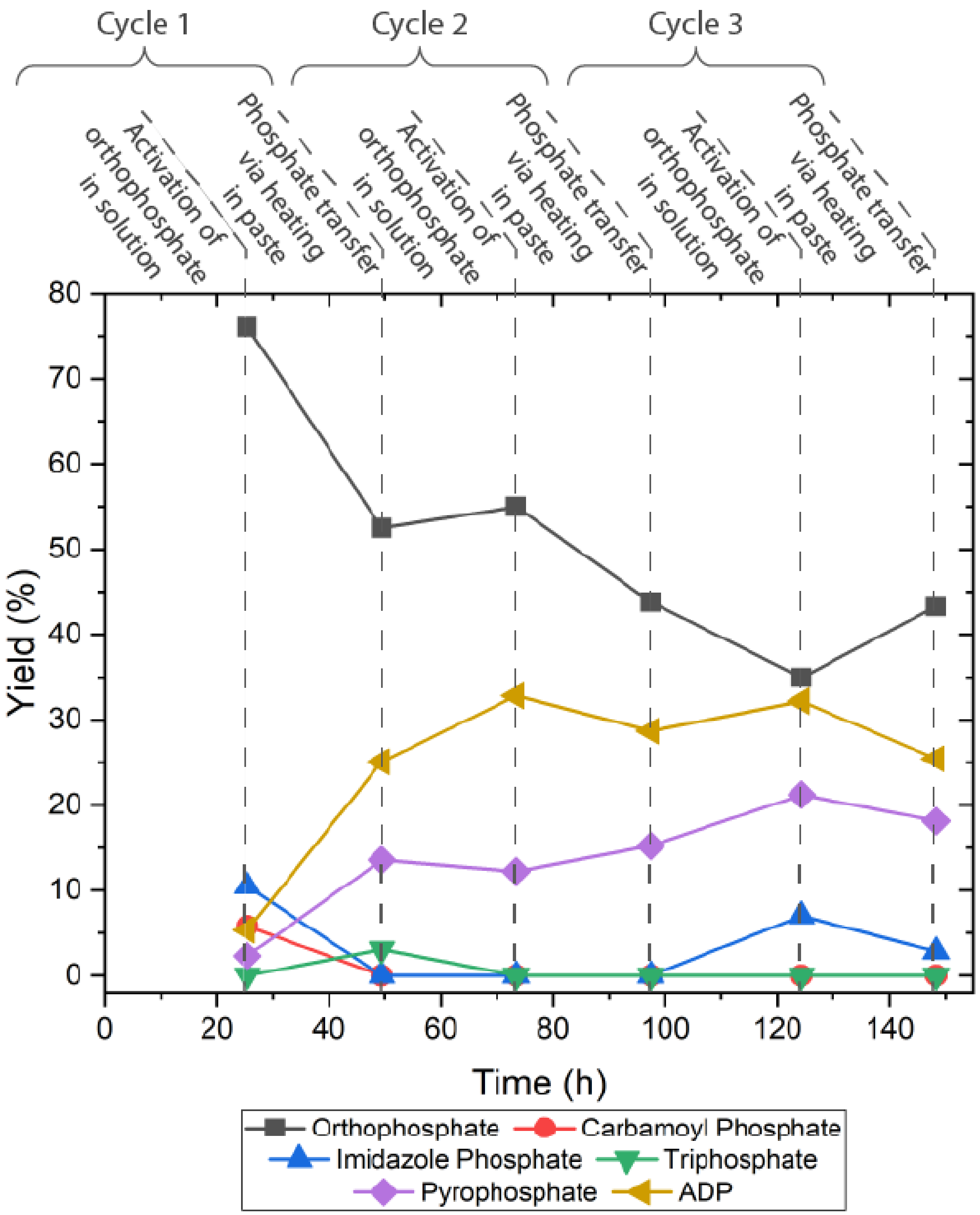

Supplementary Figure 230: Changes in yield for all phosphorous species over the course of three cycles for the reaction with 750 mM adenosine-5'-monophosphate, 450 mM potassium cyanate and 20 mM sodium phosphate in 1.0 M imidazole buffer. A minor amount of phosphorylation (in summation < 5.5 mM) also occurred at several positions on the ribose ring of AMP and for clarity these have been omitted from this figure.

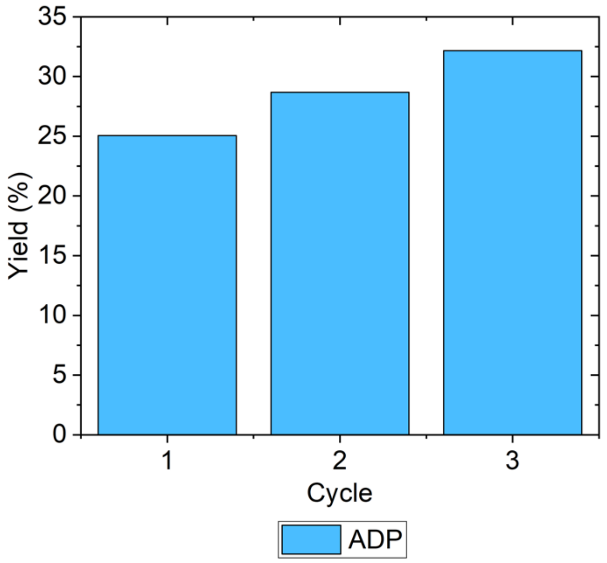

Supplementary Figure 231: Yield of adenosine-5'-diphosphate after each cycle for the reaction with 750 mM adenosine-5'-monophosphate, 450 mM potassium cyanate and 20 mM sodium phosphate in 1.0 M imidazole buffer. Yield at Cycle 3 is after drying to paste and prior to heating which resulted in a marginal loss of ADP.

S7.4.9 Phosphorylation of adenosine-5'-monophosphate with 20 mM orthophosphate repeat 2

This reaction was performed according to the procedure in Section S7.1 but with 750 mM of adenosine-5'-monophosphate disodium salt (1.096 g, 3.0 mmol) and 20 mM sodium phosphate dibasic dihydrate salt (71.2 mg, 0.4 mmol).

A total of three cycles were performed. Formation of imidazole phosphate was observed in solution and remained present after drying the solution to a paste. Formation of adenosine-5'-diphosphate occurred during both the drying to the paste over a period of ~24 h and during the heating of the paste to 50 °C for ~24 h (Supplementary Figure 232, 233 and 234).

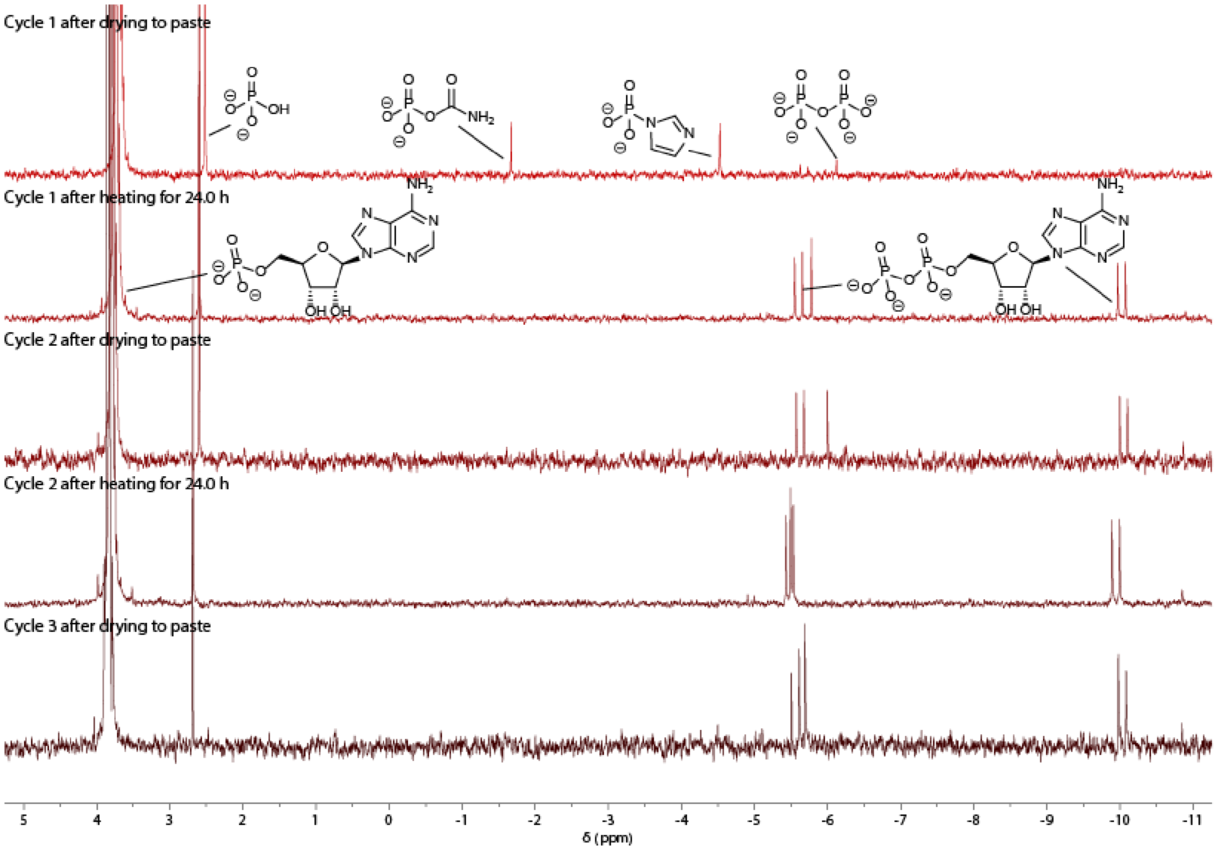

Supplementary Figure 232: Representative ^{31}P NMR spectra for the three cycles with 750 mM adenosine-5'-monophosphate, 450 mM potassium cyanate and 20 mM sodium phosphate in 1.0 M imidazole buffer. For each cycle the ^{31}P NMR spectrum after the reaction had been dried to a paste and the ^{31}P NMR spectrum after heating the reaction to 50 °C for ~24 h are shown. All NMR spectra were measured in a 0.5 M citric acid buffer at pH 6.85 9 : 1 H_2O : D_2O. The chemical shift of the pyrophosphate peak is variable at pH 6.85 due to the pyrophosphate pK_a = 6.6.

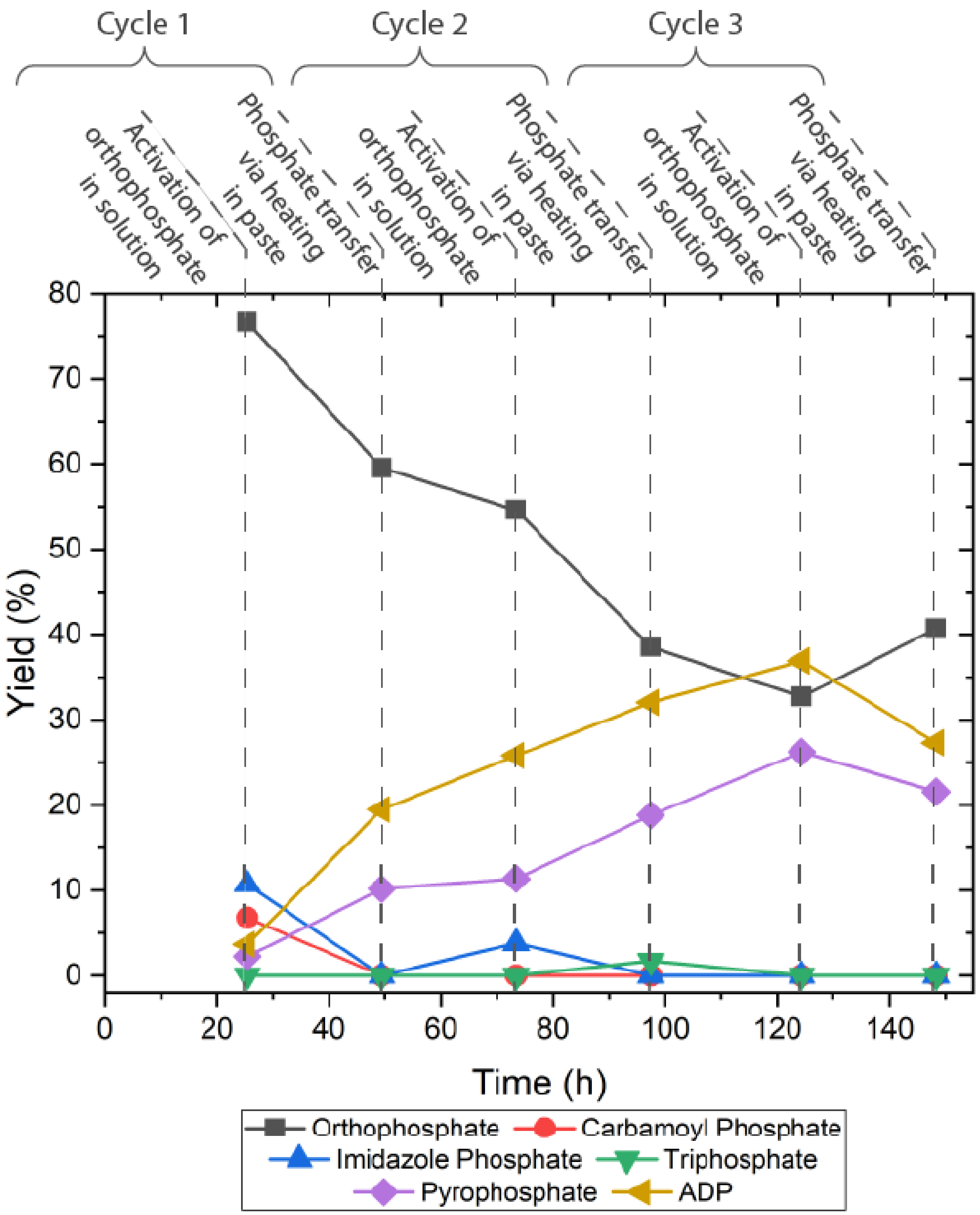

Supplementary Figure 233: Changes in yield for all phosphorous species over the course of three cycles for the reaction with 750 mM adenosine-5'-monophosphate, 450 mM potassium cyanate and 20 mM sodium phosphate in 1.0 M imidazole buffer. A minor amount of phosphorylation (in summation < 5.5 mM) also occurred at several positions on the ribose ring of AMP and for clarity these have been omitted from this figure.

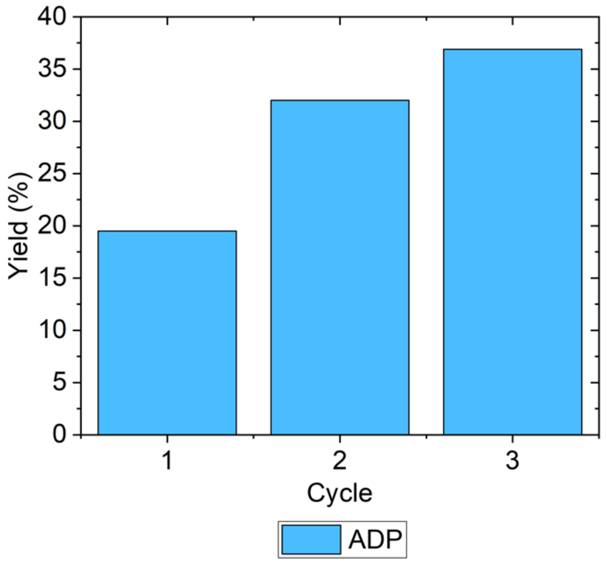

Supplementary Figure 234: Yield of adenosine-5'-diphosphate after each cycle for the reaction with 750 mM adenosine-5'-monophosphate, 450 mM potassium cyanate and 20 mM sodium phosphate in 1.0 M imidazole buffer. Yield at Cycle 3 is after drying to paste and prior to heating which resulted in a marginal loss of ADP.

S7.5 Phosphorylation of serine with orthophosphate recycling

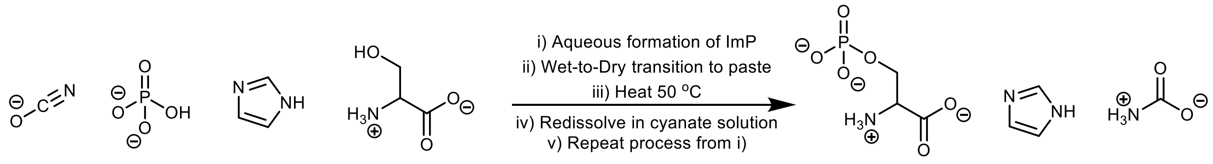

S7.5.1 Phosphorylation of serine with 50 mM orthophosphate

This reaction was performed according to the procedure in Section S7.1 but with 750 mM of serine (276.3 mg, 3.0 mmol) and 50 mM sodium phosphate dibasic dihydrate salt (178.0 mg, 1.0 mmol).

A total of three cycles were performed. Formation of imidazole phosphate was observed in solution and remained present after drying the solution to a paste. Formation of O-phosphorylated serine occurred during both the drying to the paste over a period of ~24 h and during the heating of the paste to 50 °C for ~24 h (Supplementary Figure 235, 236 and 237).

No phosphorylation of serine occurred in the first cycle. In the ^{1}H NMR spectra, multiple peaks consistent with the formation of N-carbamoyl serine and Ser-Ser dipeptide formation were observed.[3]

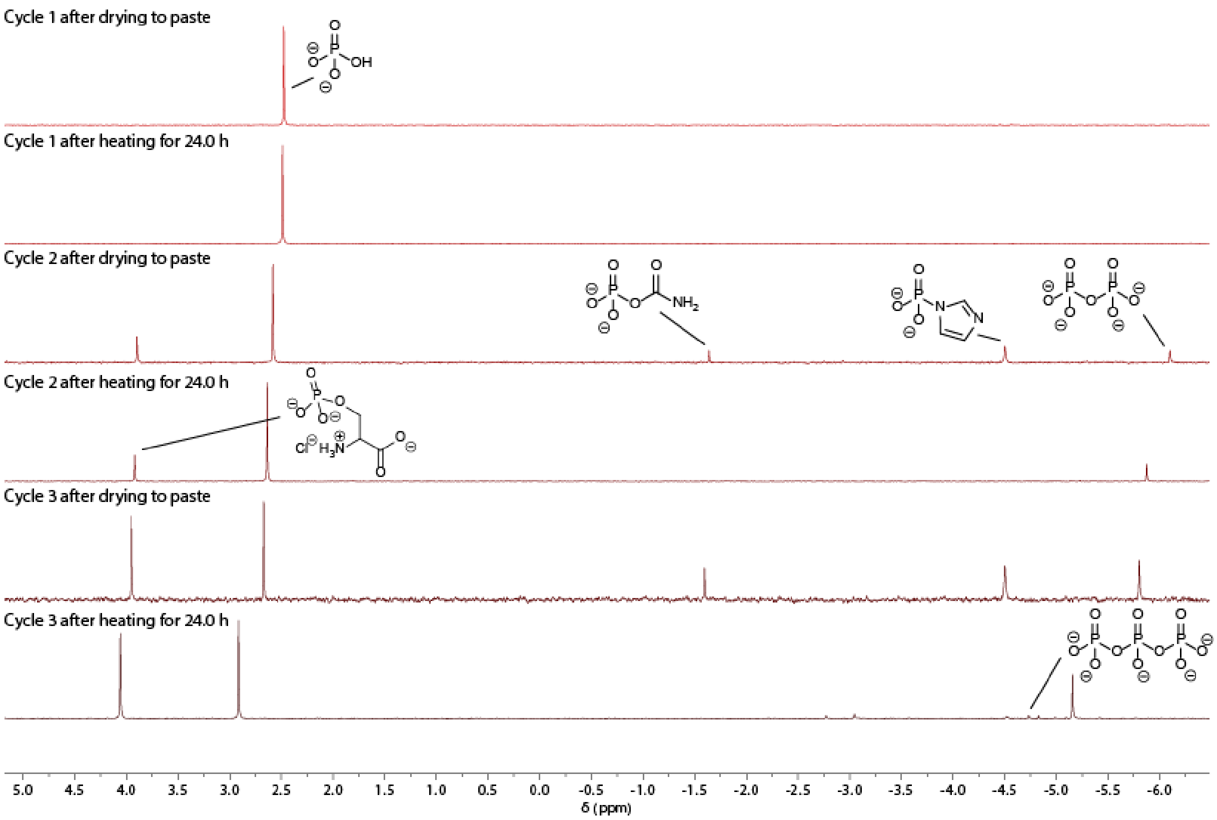

Supplementary Figure 235: Representative ^{31}P NMR spectra for the three cycles with 750 mM serine, 450 mM potassium cyanate and 50 mM sodium phosphate in 1.0 M imidazole buffer. For each cycle the ^{31}P NMR spectrum after the reaction had been dried to a paste and the ^{31}P NMR spectrum after heating the reaction to 50 °C for ~24 h are shown. All NMR spectra were measured in a 0.5 M citric acid buffer at pH 6.85 9 : 1 H_2O : D_2O. The chemical shift of the pyrophosphate peak is variable at pH 6.85 due to the pyrophosphate pK_a = 6.6.

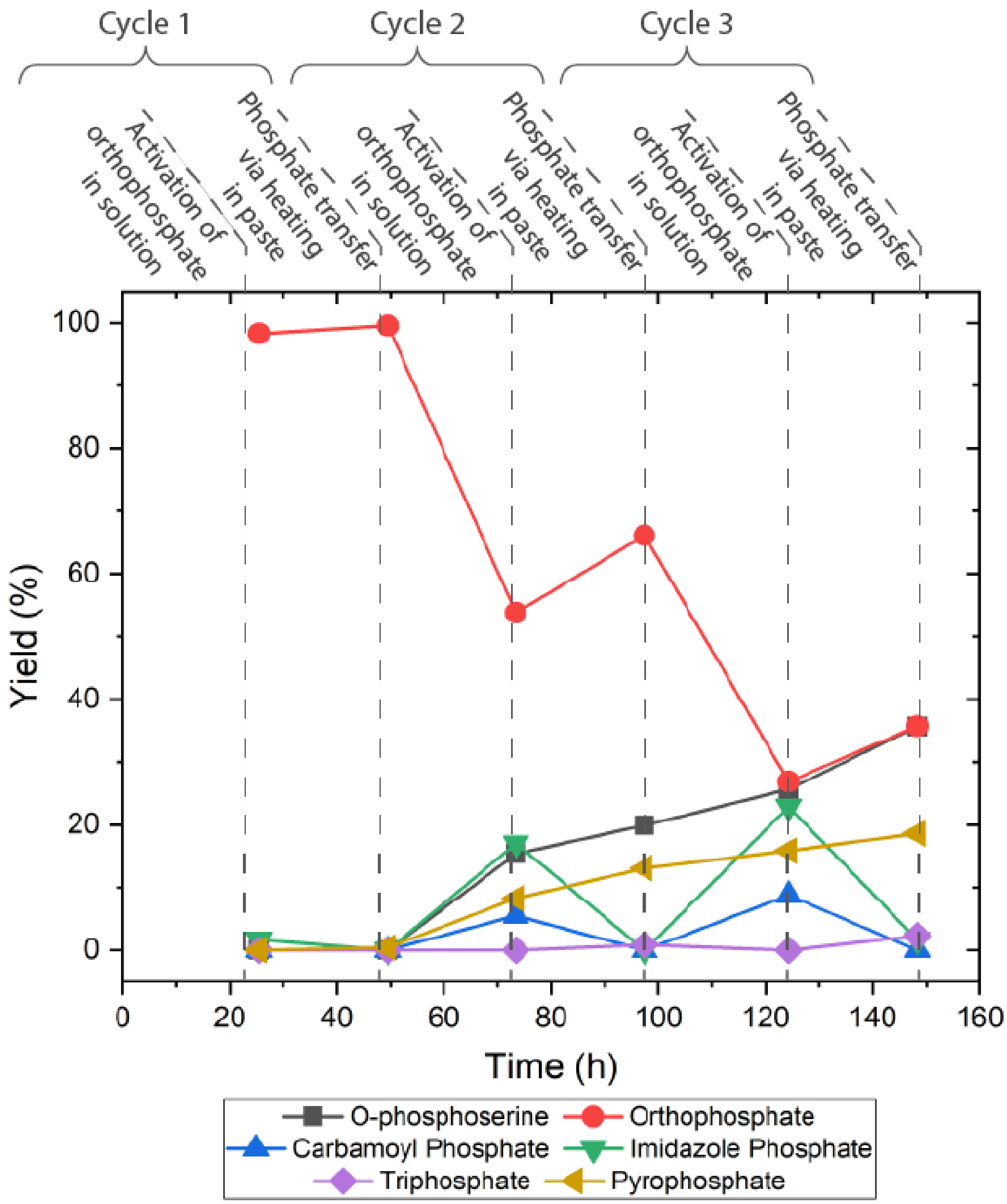

Supplementary Figure 236: Changes in yield for all phosphorous species over the course of three cycles for the reaction with 750 mM serine, 450 mM potassium cyanate and 50 mM sodium phosphate in 1.0 M imidazole buffer.

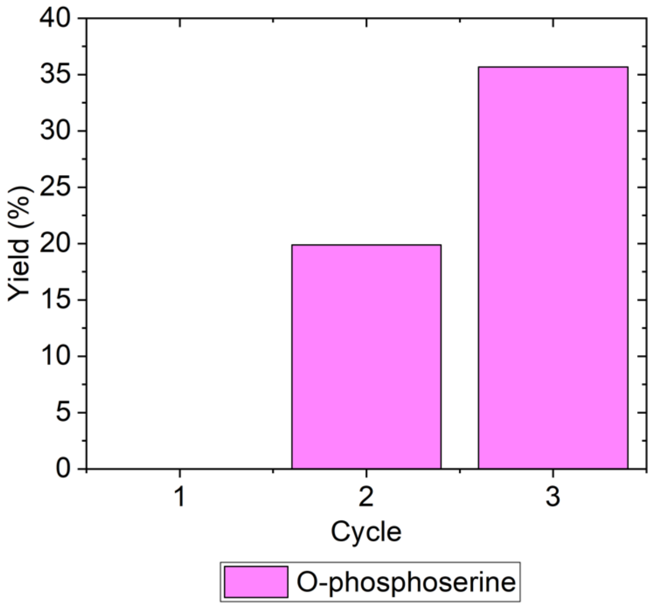

Supplementary Figure 237: Yield of O-phosphorylated serine after each cycle for the reaction with 750 mM serine, 450 mM potassium cyanate and 50 mM sodium phosphate in 1.0 M imidazole buffer.

A spiking experiment with a genuine sample of O-phosphorylated serine confirmed its formation (Supplementary Figure 238).

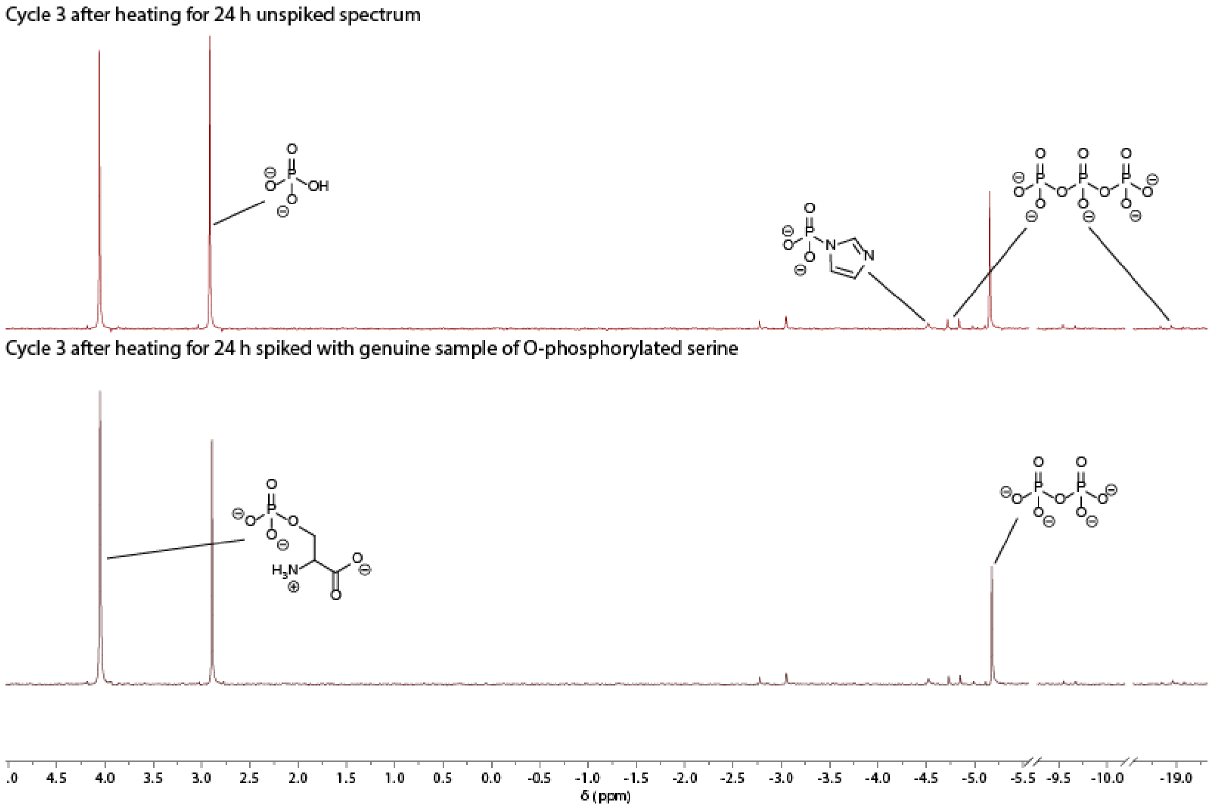

Supplementary Figure 238: ^{31}P NMR spectra of a spiking experiment for the third cycle of the reaction with 750 mM serine, 450 mM potassium cyanate and 50 mM sodium phosphate in 1.0 M imidazole buffer. Top spectrum is unspiked. Bottom spectrum is with the addition of genuine O-phosphorylated serine.

S7.5.2 Phosphorylation of serine with 20 mM orthophosphate

This reaction was performed according to the procedure in Section S7.1 but with 750 mM of serine (276.3 mg, 3.0 mmol) and 20 mM sodium phosphate dibasic dihydrate salt (71.2 mg, 0.4 mmol).

A total of three cycles were performed. Formation of imidazole phosphate was observed in solution and remained present after drying the solution to a paste. Formation of O-phosphorylated serine occurred during both the drying to the paste over a period of ~24 h and during the heating of the paste to 50 °C for ~24 h (Supplementary Figure 239, 240 and 241).

No phosphorylation of serine occurred in the first cycle. In the ^{1}H NMR spectra, multiple peaks consistent with the formation of N-carbamoyl serine and Ser-Ser dipeptide formation were observed.[3]

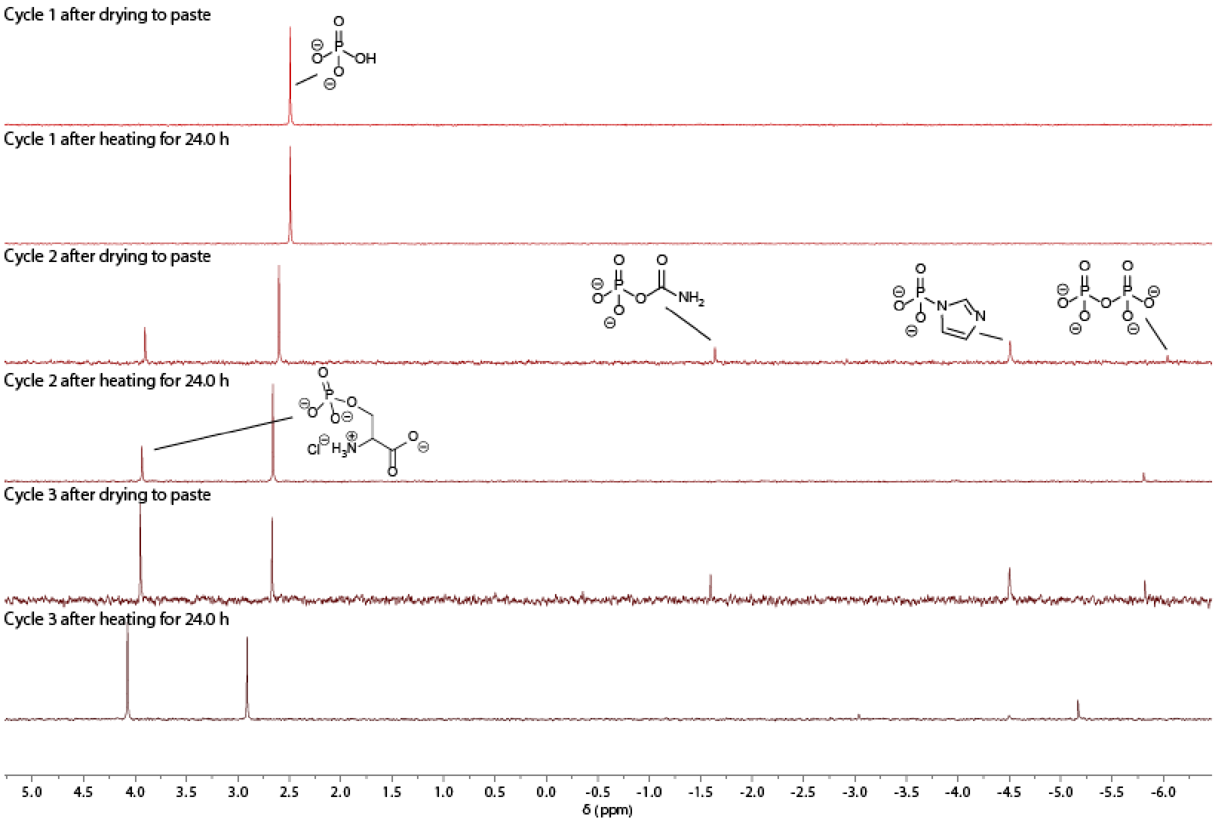

Supplementary Figure 239: Representative ^{31}P NMR spectra for the three cycles with 750 mM serine, 450 mM potassium cyanate and 20 mM sodium phosphate in 1.0 M imidazole buffer. For each cycle the ^{31}P NMR spectrum after the reaction had been dried to a paste and the ^{31}P NMR spectrum after heating the reaction to 50 °C for ~24 h are shown. All NMR spectra were measured in a 0.5 M citric acid buffer at pH 6.85 9 : 1 H_2O : D_2O. The chemical shift of the pyrophosphate peak is variable at pH 6.85 due to the pyrophosphate pK_a = 6.6.

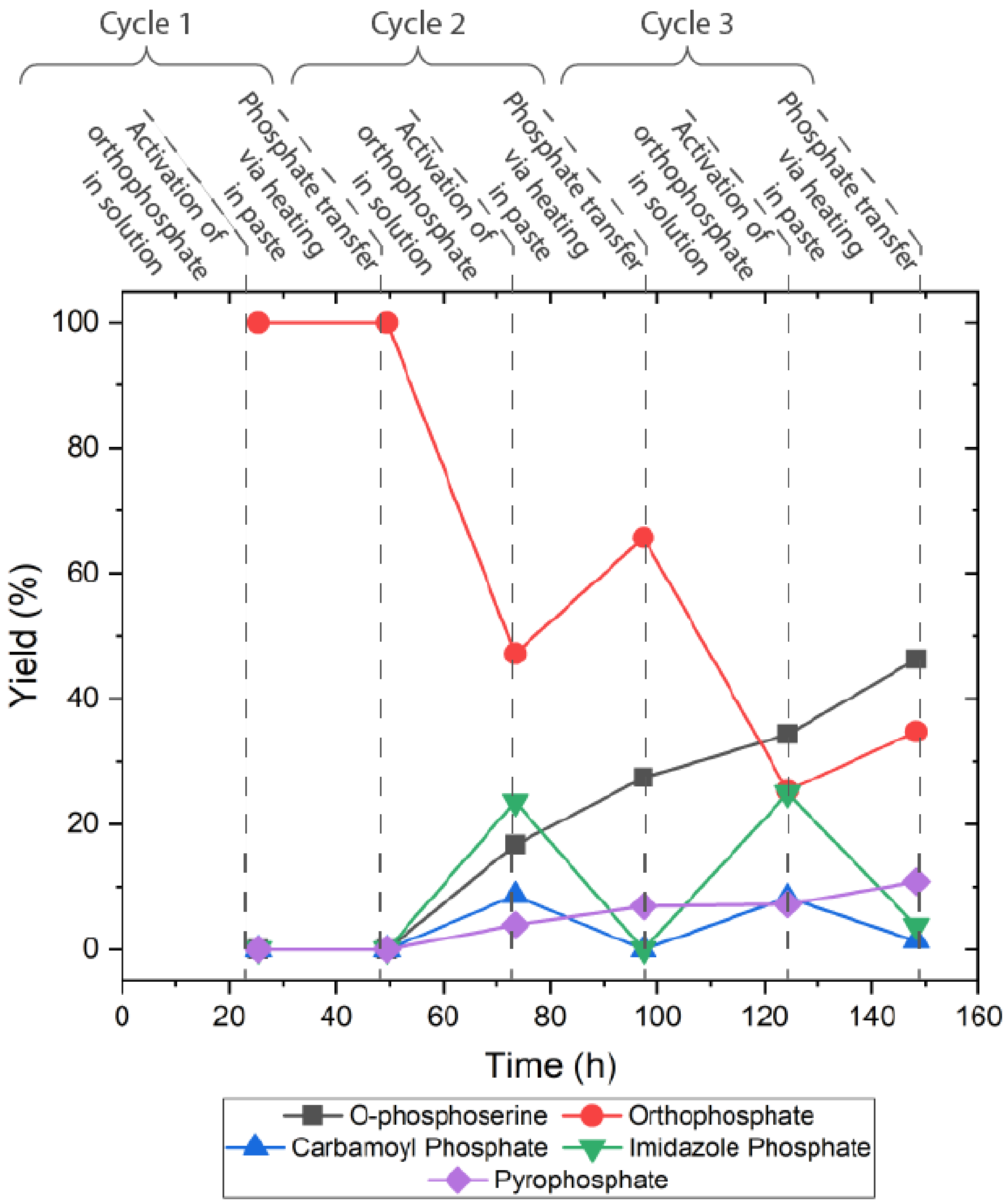

Supplementary Figure 240: Changes in yield for all phosphorous species over the course of three cycles for the reaction with 750 mM serine, 450 mM potassium cyanate and 20 mM sodium phosphate in 1.0 M imidazole buffer.

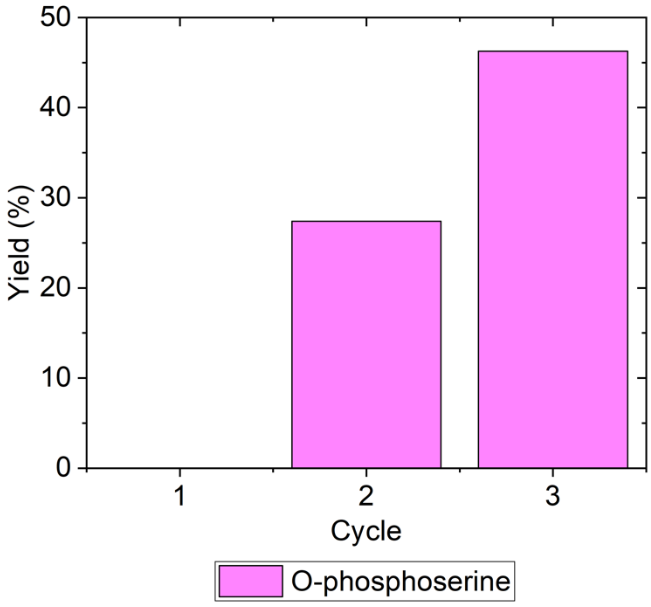

Supplementary Figure 241: Yield of O-phosphorylated serine after each cycle for the reaction with 750 mM serine, 450 mM potassium cyanate and 20 mM sodium phosphate in 1.0 M imidazole buffer.

A spiking experiment with a genuine sample of O-phosphorylated serine confirmed its formation (Supplementary Figure 242).

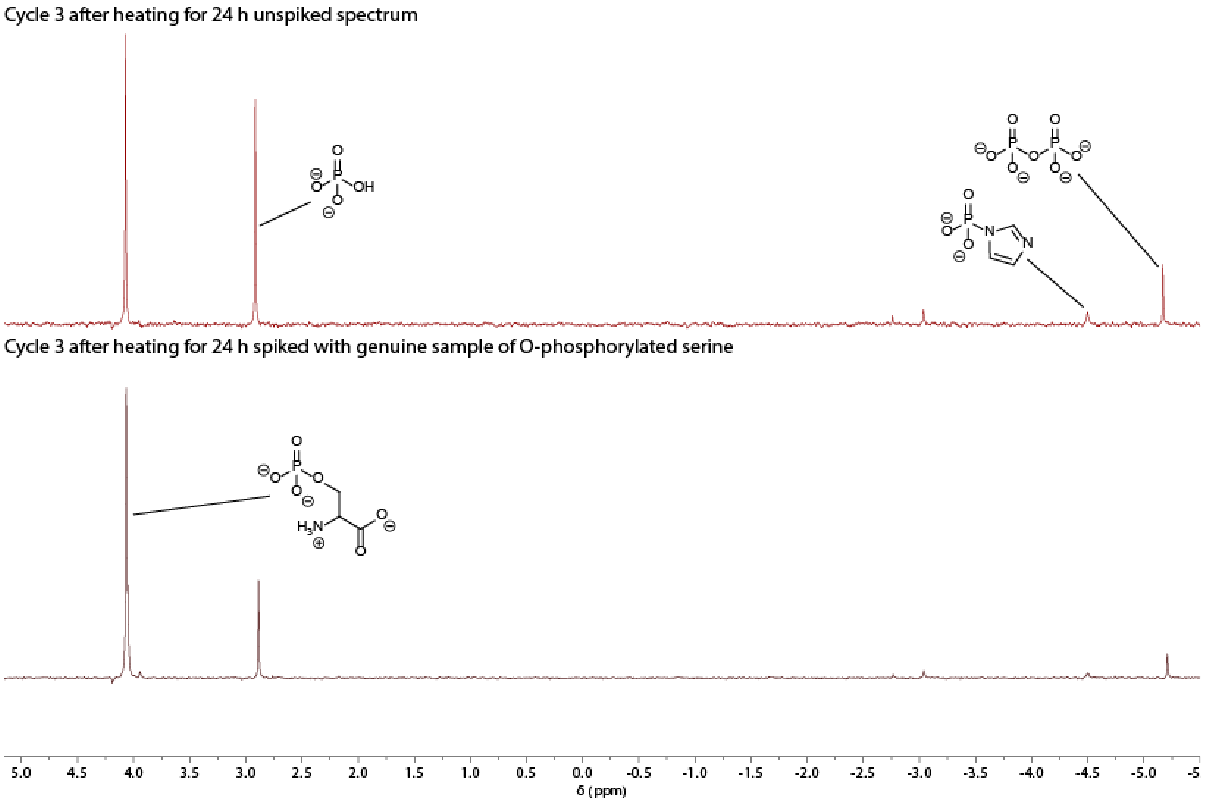

Supplementary Figure 242: ^{31}P NMR spectra of a spiking experiment for the third cycle of the reaction with 750 mM serine, 450 mM potassium cyanate and 20 mM sodium phosphate in 1.0 M imidazole buffer. Top spectrum is unspiked. Bottom spectrum is with the addition of genuine O-phosphorylated serine.

S7.6 Phosphorylation of D-ribose with orthophosphate recycling

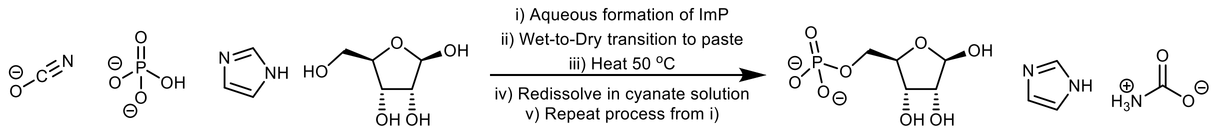

S7.6.1 Phosphorylation of D-ribose with 50 mM orthophosphate

This reaction was performed according to the procedure in Section S7.1 but with 150 mM of D-ribose (63.6 mg, 0.6 mmol) and 50 mM sodium phosphate dibasic dihydrate salt (178.0 mg, 1.0 mmol). Note that the use of the ribose-5-phosphate structure in the scheme above is to indicate phosphorylated ribose and not to identify the specific species.

A total of three cycles were performed. Formation of imidazole phosphate was observed in solution and remained present after drying the solution to a paste. Multiple peaks for phosphorylated ribose were observed in the ^{31}P NMR spectra and occurred during both the drying to the paste over a period of ~24 h and during the heating of the paste to 50 °C for ~24 h (Supplementary Figure 243, 244 and 245).

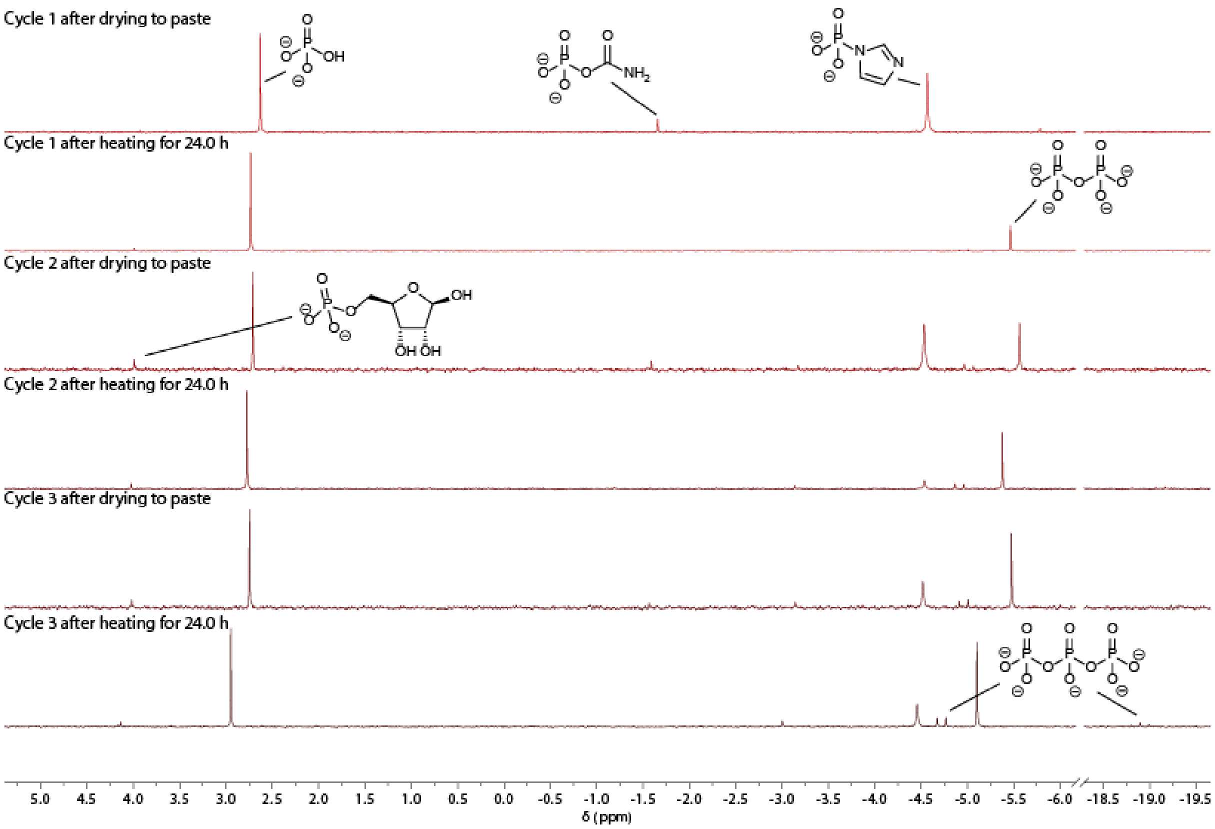

Supplementary Figure 243: Representative ^{31}P NMR spectra for the three cycles with 150 mM of D-ribose, 450 mM potassium cyanate and 50 mM sodium phosphate in 1.0 M imidazole buffer. For each cycle the ^{31}P NMR spectrum after the reaction had been dried to a paste and the ^{31}P NMR spectrum after heating the reaction to 50 °C for ~24 h are shown. All NMR spectra were measured in a 0.5 M citric acid buffer at pH 6.85 9 : 1 H_2O : D_2O. The chemical shift of the pyrophosphate peak is variable at pH 6.85 due to the pyrophosphate pK_a = 6.6. Use of ribose-5-phosphate structure is to indicate phosphorylated ribose and not to identify the specific species.

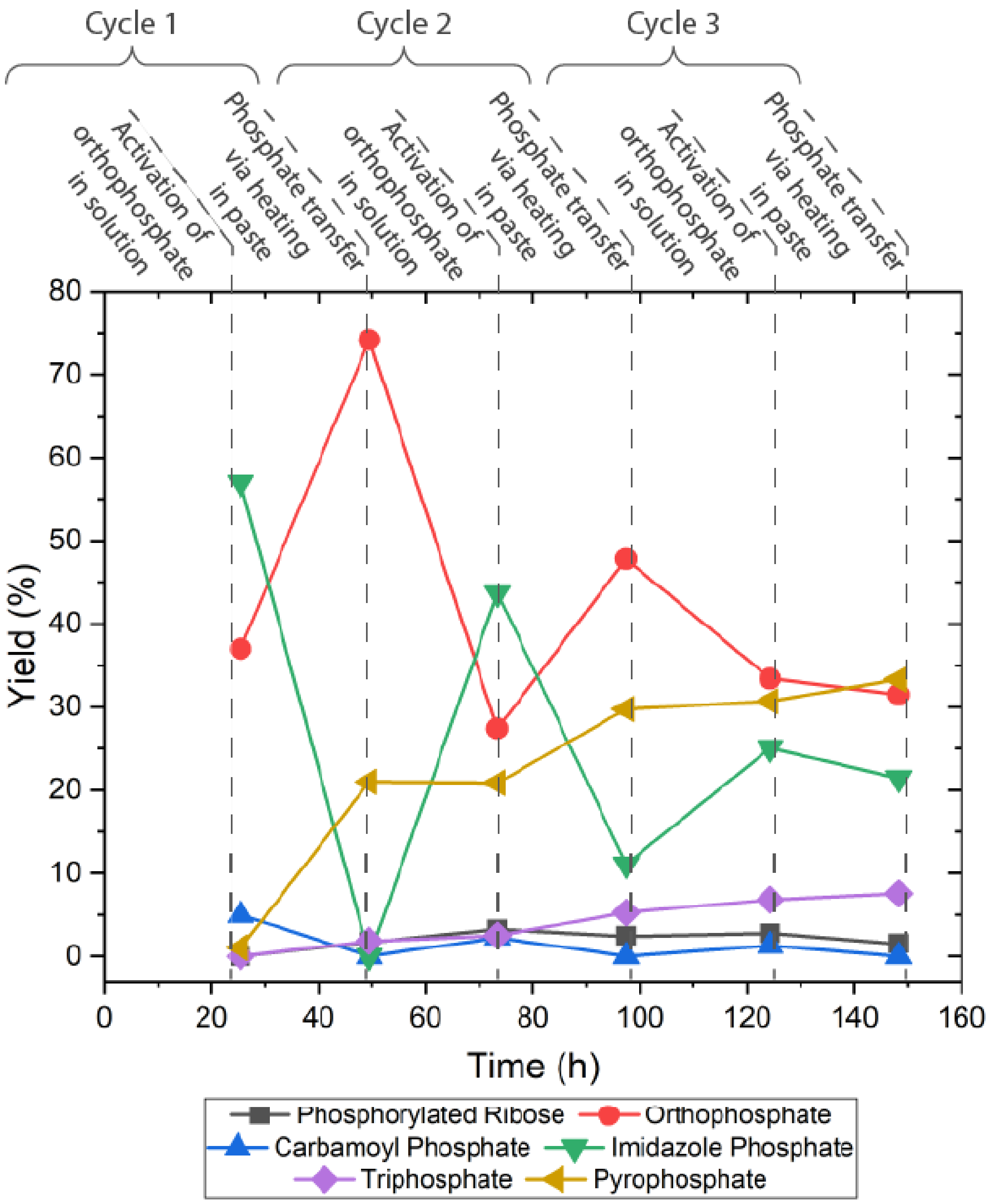

Supplementary Figure 244: Changes in yield for all phosphorous species over the course of three cycles for the reaction with 150 mM of D-ribose, 450 mM potassium cyanate and 50 mM sodium phosphate in 1.0 M imidazole buffer.

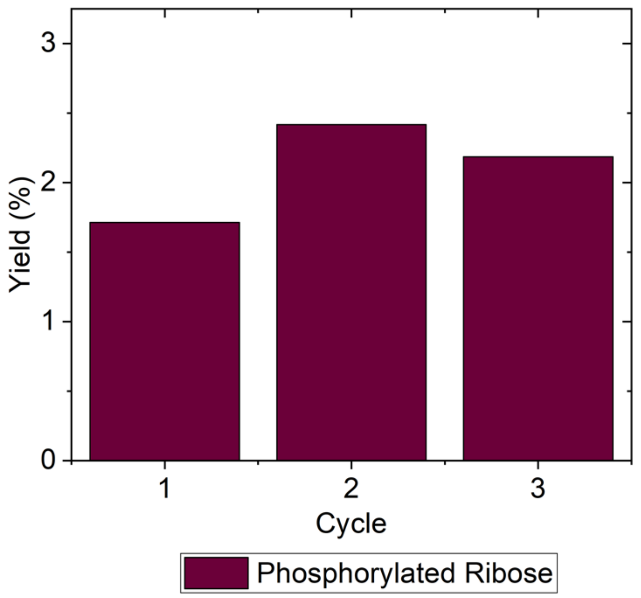

Supplementary Figure 245: Yield of ribose-5-phosphate after each cycle for the reaction with 150 mM D-ribose, 450 mM potassium cyanate and 50 mM sodium phosphate in 1.0 M imidazole buffer.

A spiking experiment with genuine samples of ribose-1-phosphate and ribose-5-phosphate which confirmed the formation of ribose-5-phosphate along with one other phosphorylated ribose species (Supplementary Figure 246).

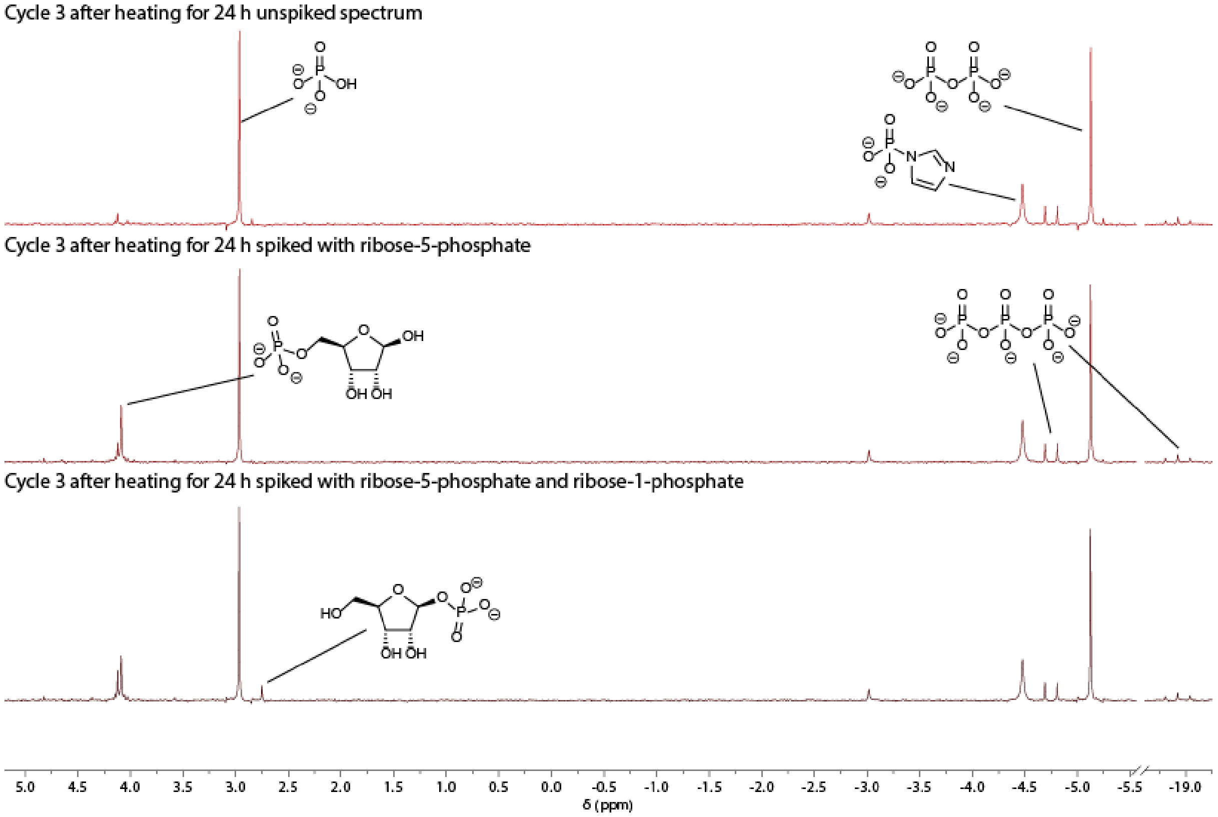

Supplementary Figure 246: ^{31}P NMR spectra of a spiking experiment for the third cycle of the reaction with 150 mM of D-ribose, 450 mM potassium cyanate and 50 mM sodium phosphate in 1.0 M imidazole buffer. Top spectrum is unspiked. Bottom spectrum is with the addition of genuine ribose-5-phosphate.

S7.6.2 *Phosphorylation of D-ribose with 20 mM orthophosphate*

This reaction was performed according to the procedure in Section S7.1 but with 150 mM of D-ribose (63.6 mg, 0.6 mmol) and 20 mM sodium phosphate dibasic dihydrate salt (71.2 mg, 0.4 mmol).

A total of three cycles were performed. Formation of imidazole phosphate was observed in solution and remained present after drying the solution to a paste. Multiple peaks for phosphorylated ribose were observed in the ^{31}P NMR spectra and occurred during both the drying to the paste over a period of ~24 h and during the heating of the paste to 50 °C for ~24 h (Supplementary Figure 247, 248 and 249).

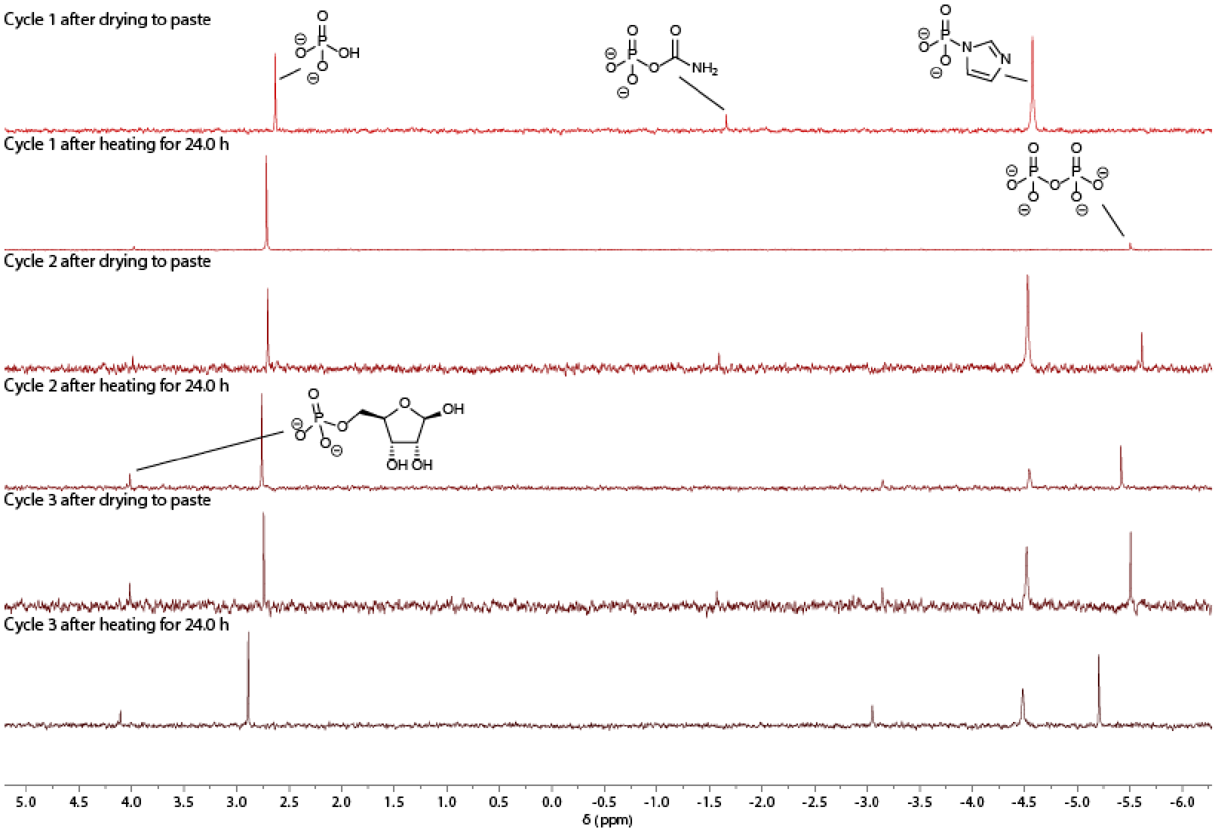

Supplementary Figure 247: Representative ^{31}P NMR spectra for the three cycles with 150 mM of D-ribose, 450 mM potassium cyanate and 20 mM sodium phosphate in 1.0 M imidazole buffer. For each cycle the ^{31}P NMR spectrum after the reaction had been dried to a paste and the ^{31}P NMR spectrum after heating the reaction to 50 °C for ~24 h are shown. All NMR spectra were measured in a 0.5 M citric acid buffer at pH 6.85 9 : 1 H_2O : D_2O. The chemical shift of the pyrophosphate peak is variable at pH 6.85 due to the pyrophosphate pK_a = 6.6. Use of ribose-5-phosphate structure is to indicate phosphorylated ribose and not to identify the specific species.

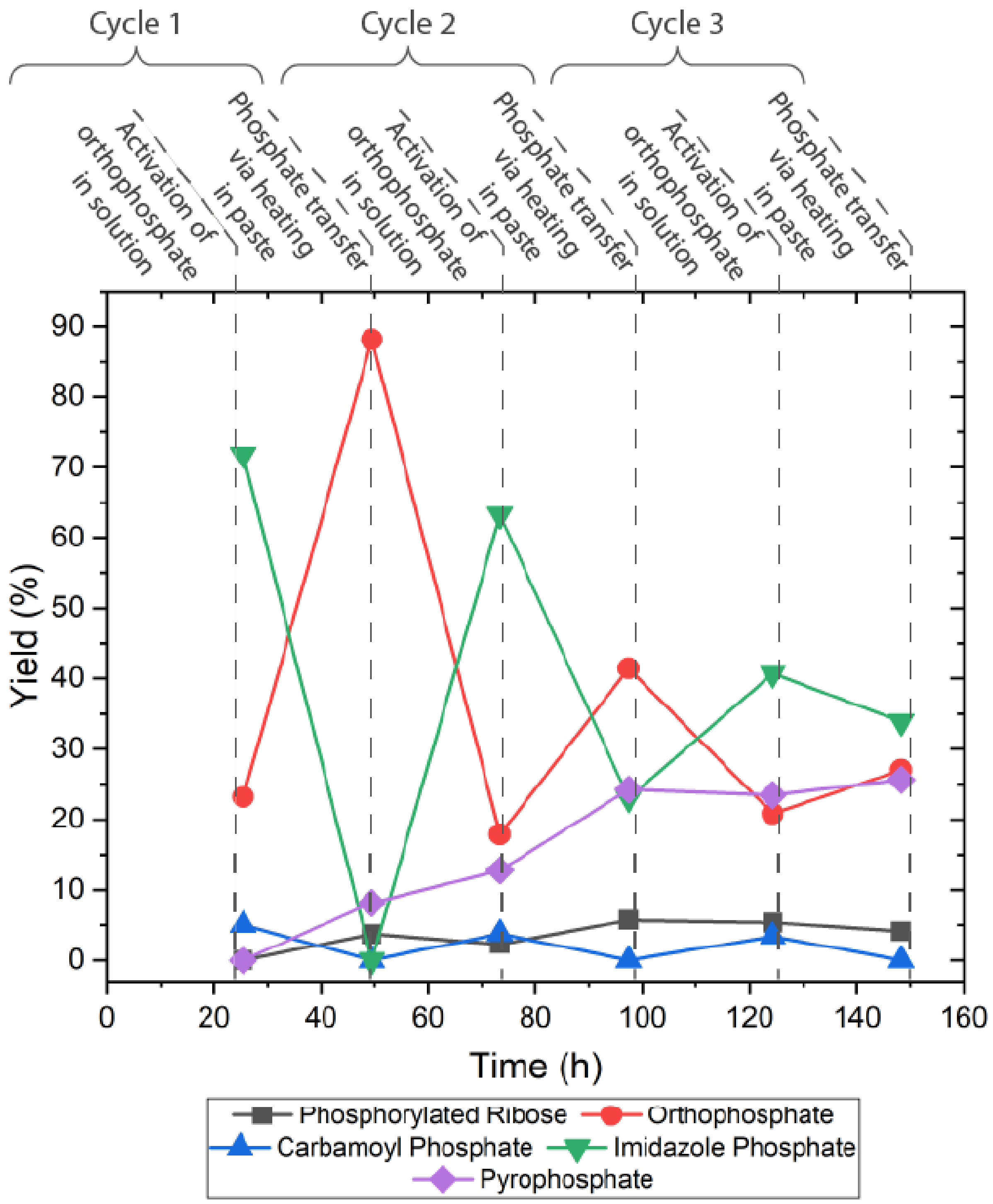

Supplementary Figure 248: Changes in yield for all phosphorous species over the course of three cycles for the reaction with 150 mM of D-ribose, 450 mM potassium cyanate and 20 mM sodium phosphate in 1.0 M imidazole buffer.

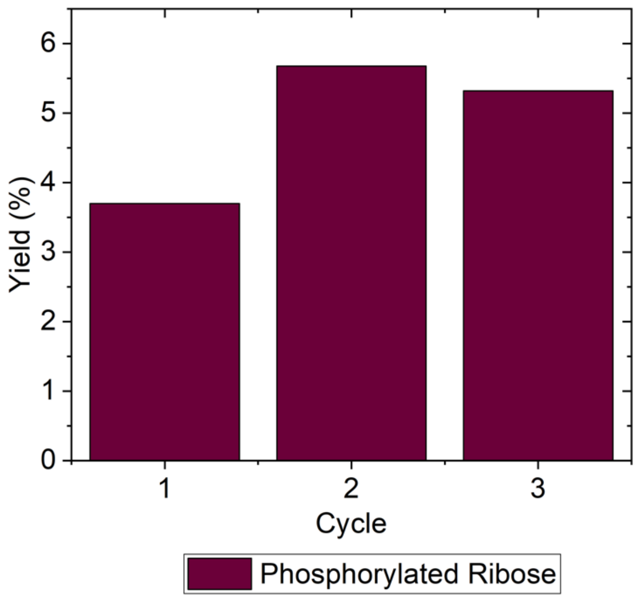

Supplementary Figure 249: Yield of ribose-5-phosphate after each cycle for the reaction with 150 mM of D-ribose, 450 mM potassium cyanate and 20 mM sodium phosphate in 1.0 M imidazole buffer.

A spiking experiment with genuine samples of ribose-1-phosphate and ribose-5-phosphate which confirmed the formation of ribose-5-phosphate along with two other phosphorylated ribose species (Supplementary Figure 250).

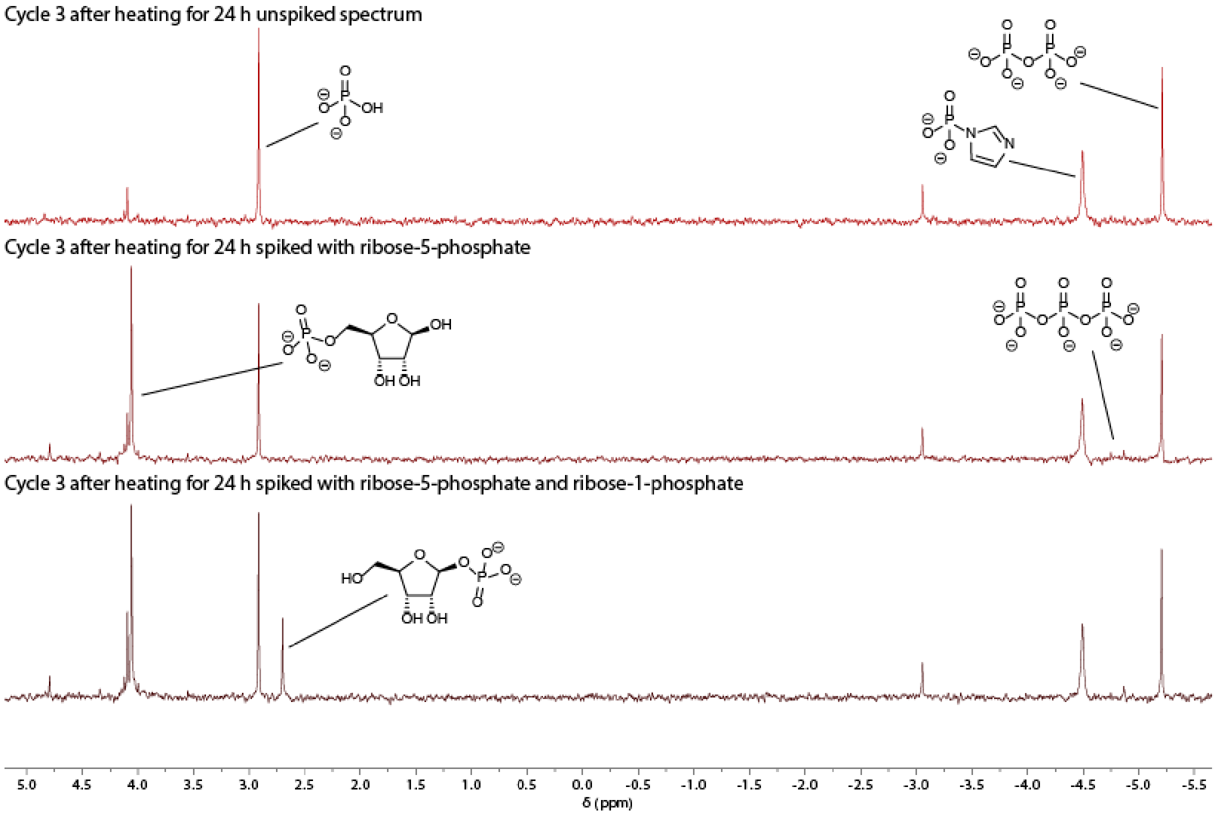

Supplementary Figure 250: ^{31}P NMR spectra of a spiking experiment for the third cycle of the reaction 150 mM of D-ribose, 450 mM potassium cyanate and 20 mM sodium phosphate in 1.0 M imidazole buffer. Top spectrum is unspiked. Bottom spectrum is with the addition of genuine ribose-5-phosphate.

S7.7 Phosphorylation of D-glucose with orthophosphate recycling

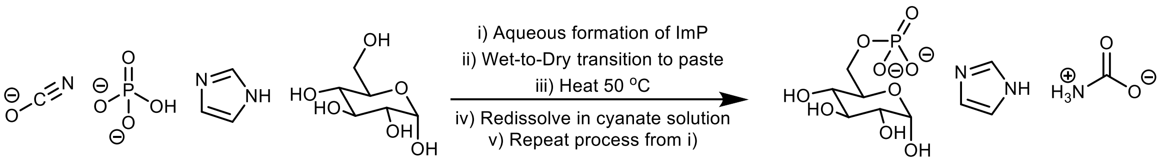

S7.7.1 Phosphorylation of D-glucose with 50 mM orthophosphate

This reaction was performed according to the procedure in Section S7.1 but with 150 mM of D-glucose (93.3 mg, 0.6 mmol) and 50 mM sodium phosphate dibasic dihydrate salt (178.0 mg, 1.0 mmol). Note that the use of the glucose-6-phosphate structure in the scheme above is to indicate phosphorylated glucose and not to identify the specific species.

A total of three cycles were performed. Formation of imidazole phosphate was observed in solution and remained present after drying the solution to a paste. Multiple peaks for phosphorylated glucose were observed in the ^{31}P NMR spectra and occurred during both the drying to the paste over a period of ~24 h and during the heating of the paste to 50 °C for ~24 h (Supplementary Figure 251, 252 and 253).

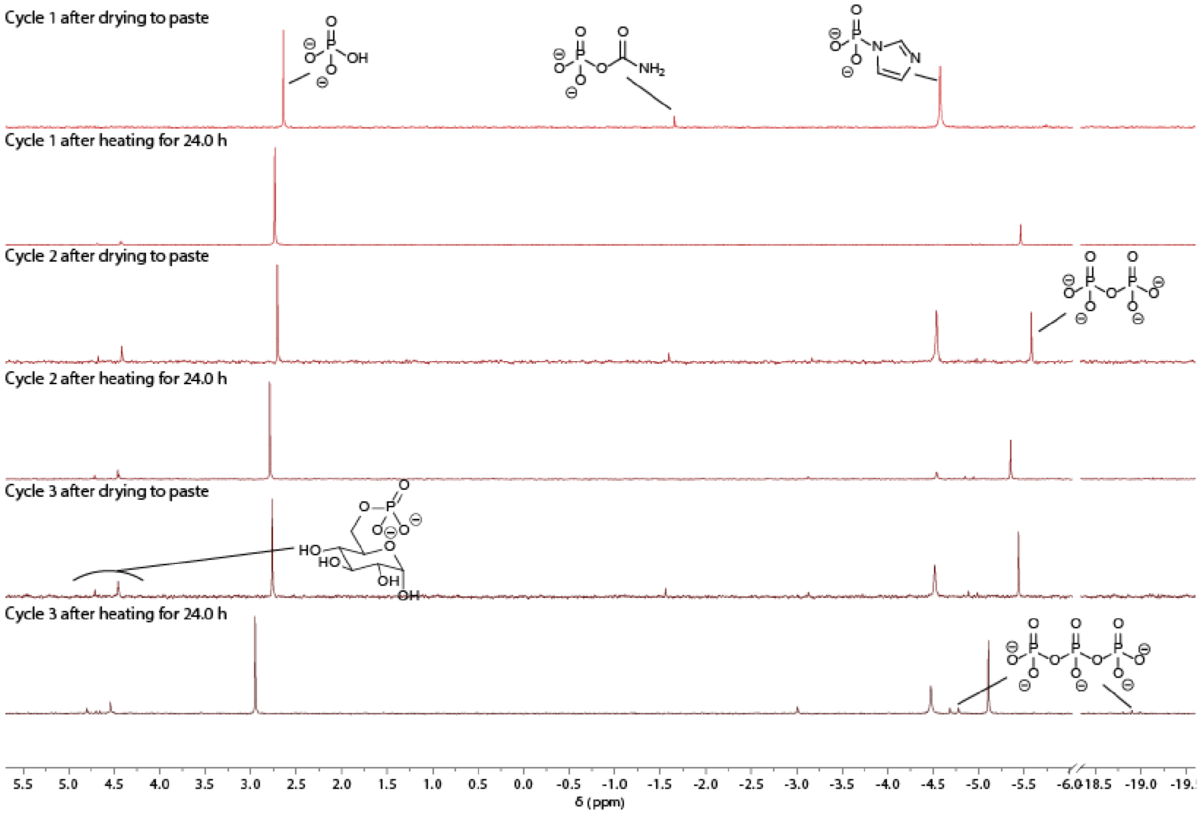

Supplementary Figure 251: Representative ^{31}P NMR spectra for the three cycles with 150 mM of D-glucose, 450 mM potassium cyanate and 50 mM sodium phosphate in 1.0 M imidazole buffer. For each cycle the ^{31}P NMR spectrum after the reaction had been dried to a paste and the ^{31}P NMR spectrum after heating the reaction to 50 °C for ~24 h are shown. All NMR spectra were measured in a 0.5 M citric acid buffer at pH 6.85 9 : 1 H_2O : D_2O. The chemical shift of the pyrophosphate peak is variable at pH 6.85 due to the pyrophosphate pK_a = 6.6. Use of glucose-6-phosphate structure is to indicate phosphorylated glucose and not to identify the specific species.

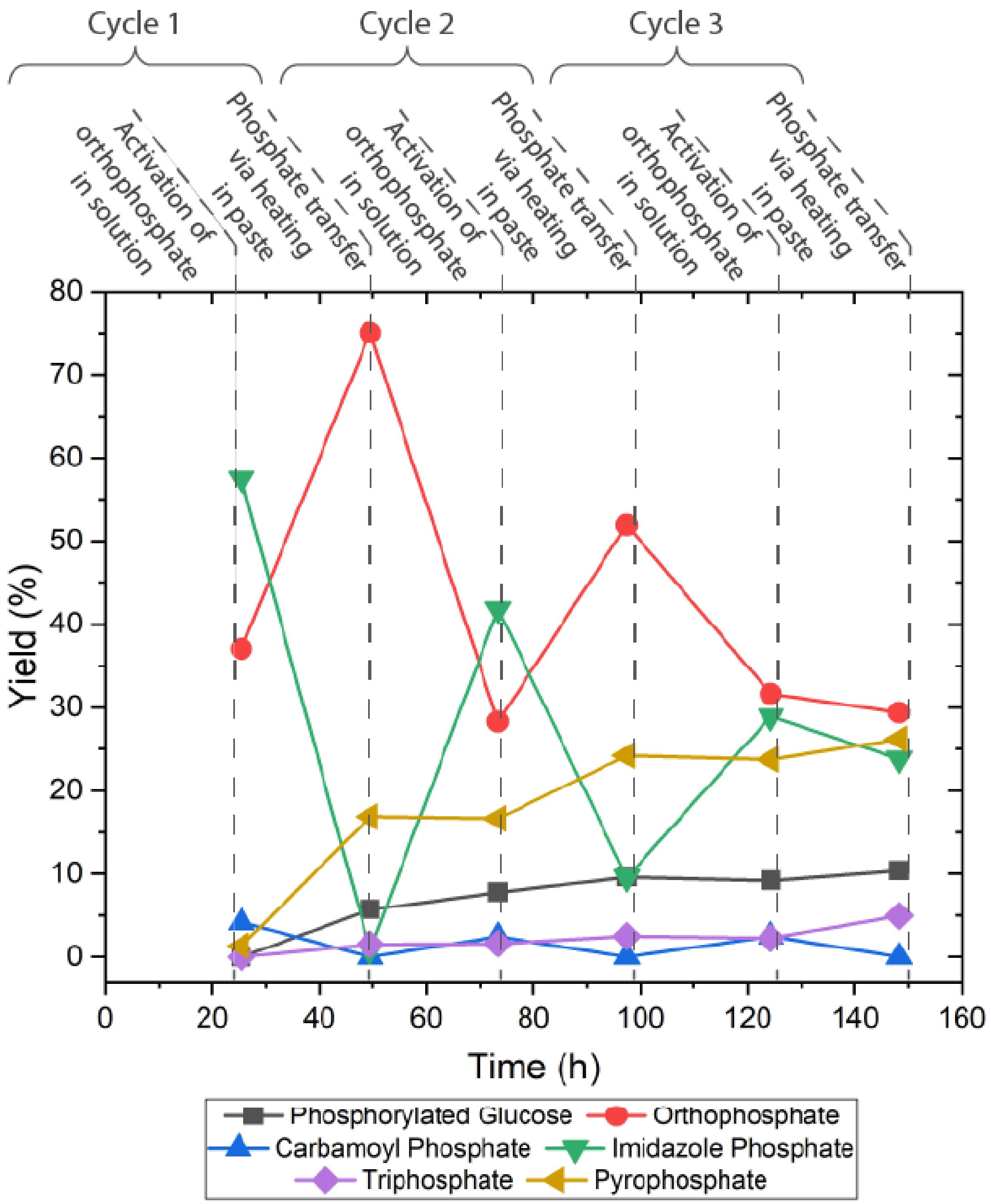

Supplementary Figure 252: Changes in yield for all phosphorous species over the course of three cycles for the reaction with 150 mM of D-glucose, 450 mM potassium cyanate and 50 mM sodium phosphate in 1.0 M imidazole buffer.

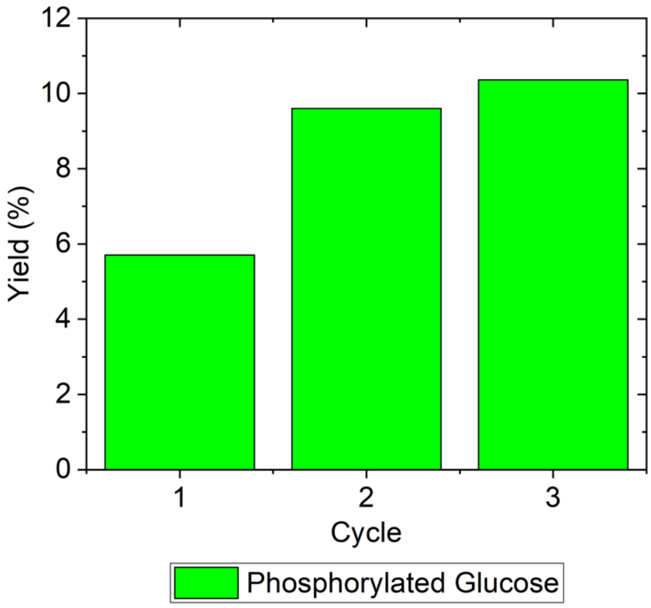

Supplementary Figure 253: Yield of glucose-phosphate after each cycle for the reaction with 150 mM D-glucose, 450 mM potassium cyanate and 50 mM sodium phosphate in 1.0 M imidazole buffer.

A spiking experiment with genuine samples of glucose-1-phosphate and glucose-6-phosphate which confirmed the formation of glucose-6-phosphate and three other phosphorylated glucose species (Supplementary Figure 254).

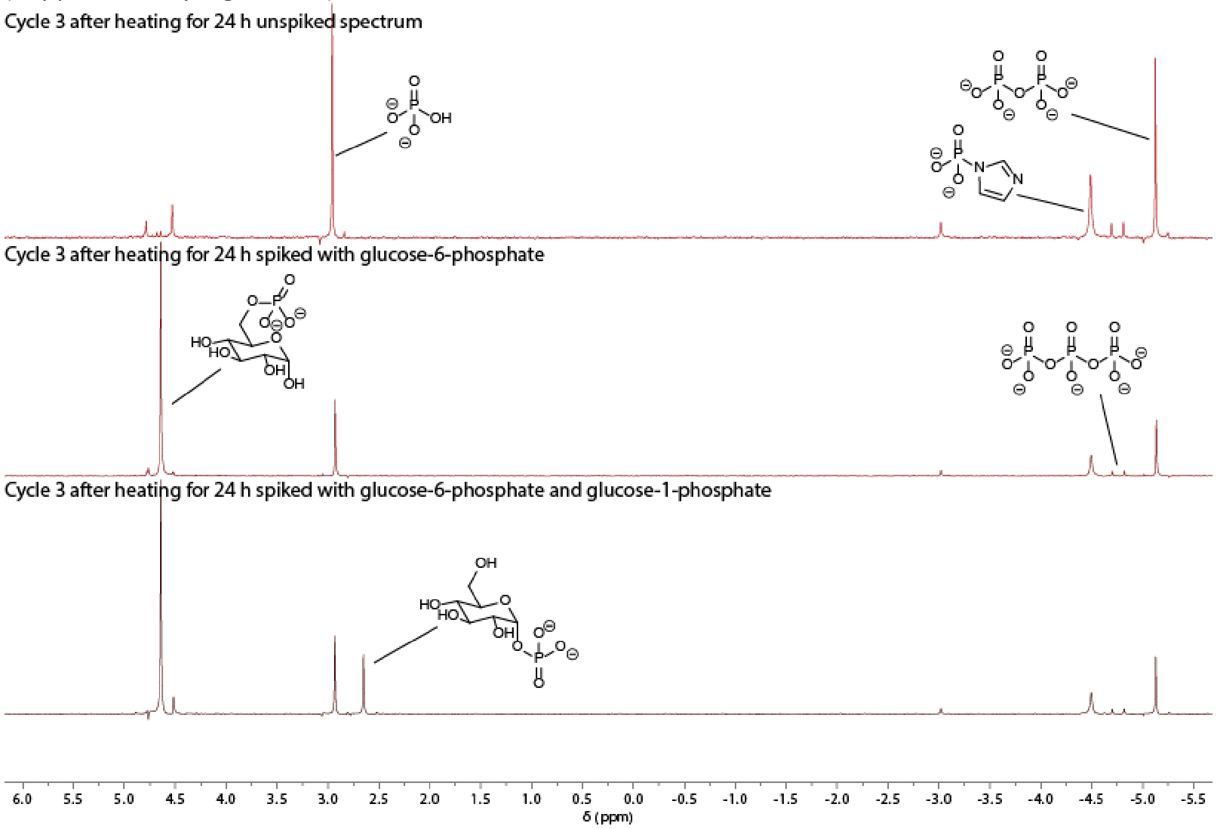

Supplementary Figure 254: ^{31}P NMR spectra of a spiking experiment for the third cycle of the reaction with 150 mM of D-glucose, 450 mM potassium cyanate and 50 mM sodium phosphate in 1.0 M imidazole buffer. Top spectrum is unspiked. Bottom spectrum is with the addition of genuine glucose-6-phosphate.

S7.7.2 Phosphorylation of D-glucose with 20 mM orthophosphate

This reaction was performed according to the procedure in Section S7.1 but with 150 mM of D-glucose (93.3 mg, 0.6 mmol) and 20 mM sodium phosphate dibasic dihydrate salt (71.2 mg, 0.4 mmol).

A total of three cycles were performed. Formation of imidazole phosphate was observed in solution and remained present after drying the solution to a paste. Multiple peaks for phosphorylated glucose were observed in the ^{31}P NMR spectra and occurred during both the drying to the paste over a period of ~24 h and during the heating of the paste to 50 °C for ~24 h (Supplementary Figure 255, 256 and 257).

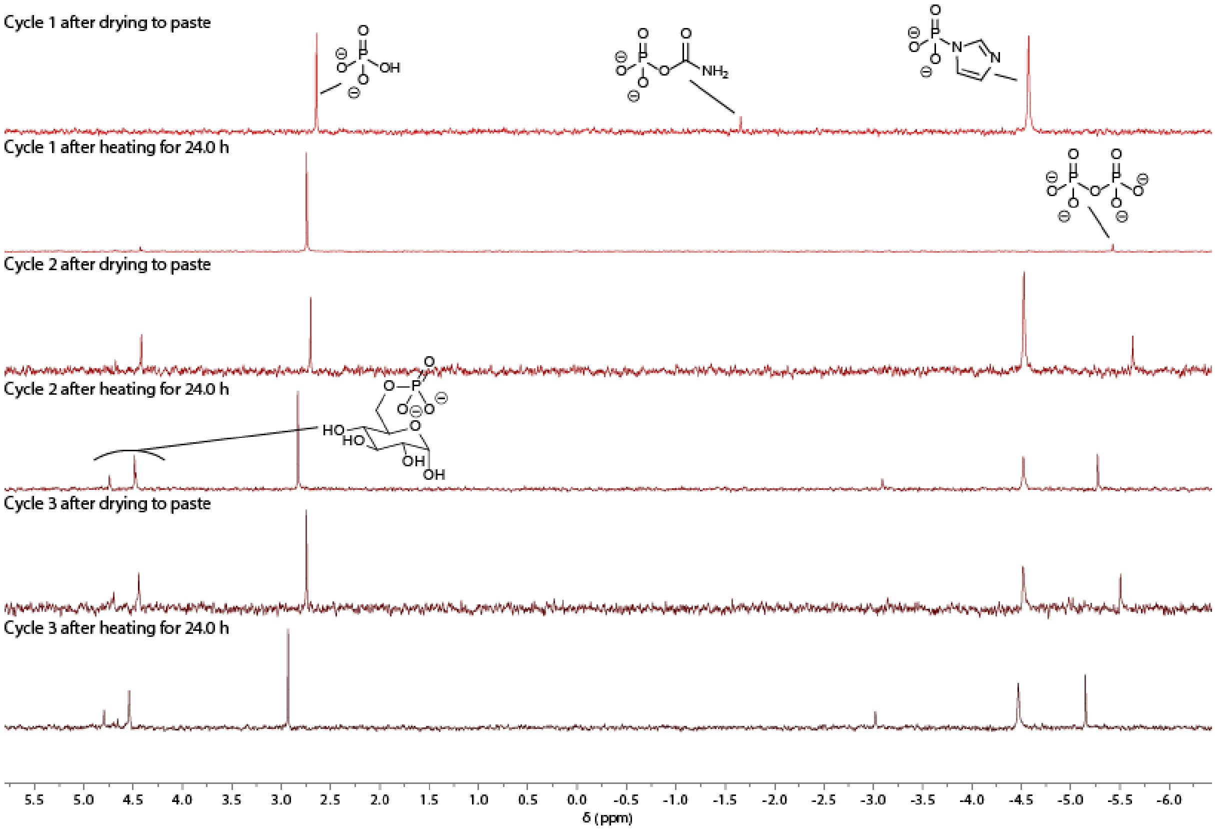

Supplementary Figure 255: Representative ^{31}P NMR spectra for the three cycles with 150 mM of D-glucose, 450 mM potassium cyanate and 20 mM sodium phosphate in 1.0 M imidazole buffer. For each cycle the ^{31}P NMR spectrum after the reaction had been dried to a paste and the ^{31}P NMR spectrum after heating the reaction to 50 °C for ~24 h are shown. All NMR spectra were measured in a 0.5 M citric acid buffer at pH 6.85 9 : 1 H_2O : D_2O. The chemical shift of the pyrophosphate peak is variable at pH 6.85 due to the pyrophosphate pK_a = 6.6. Use of glucose-6-phosphate structure is to indicate phosphorylated glucose and not to identify the specific species.

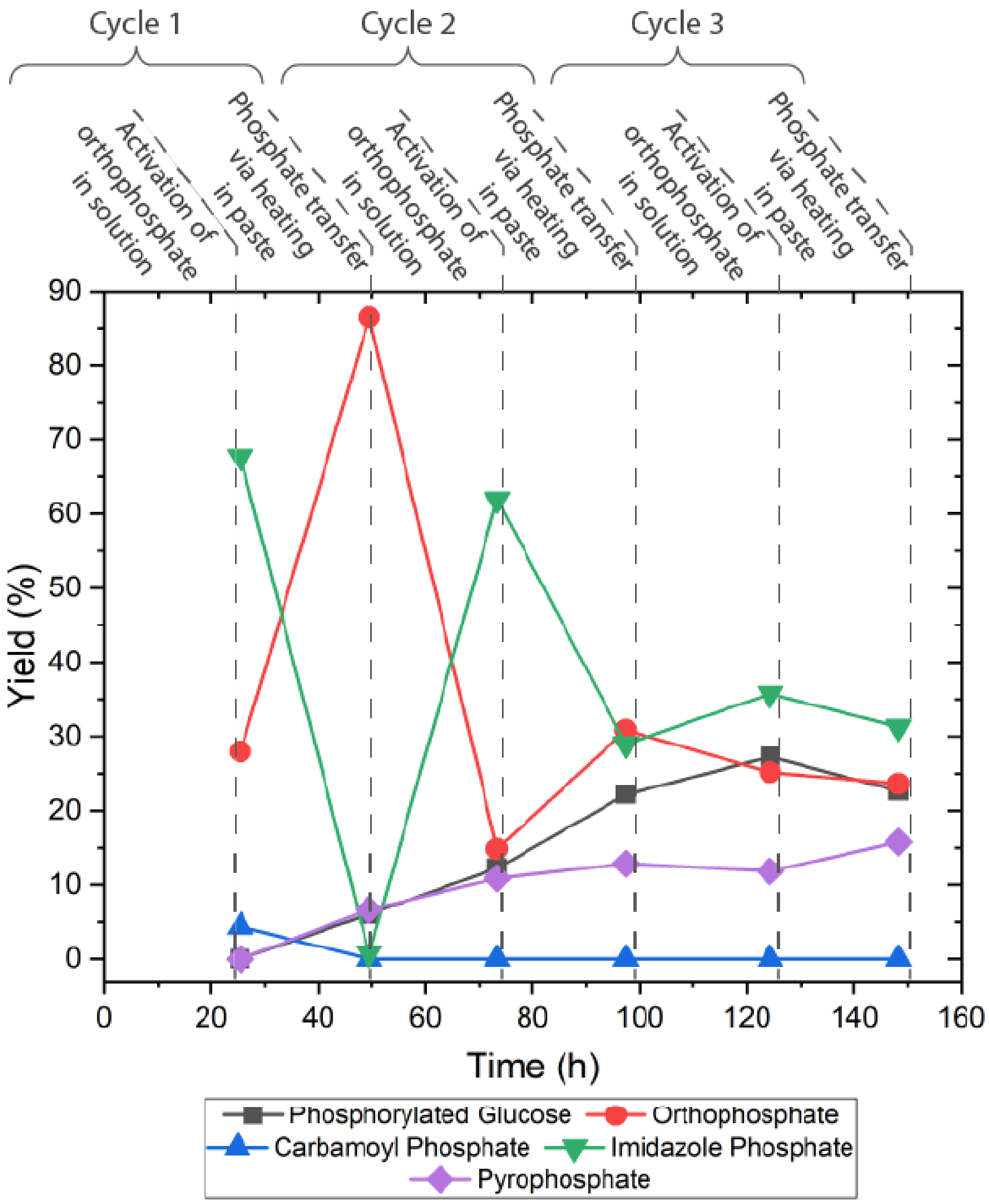

Supplementary Figure 256: Changes in yield for all phosphorous species over the course of three cycles for the reaction with 150 mM of D-glucose, 450 mM potassium cyanate and 20 mM sodium phosphate in 1.0 M imidazole buffer.

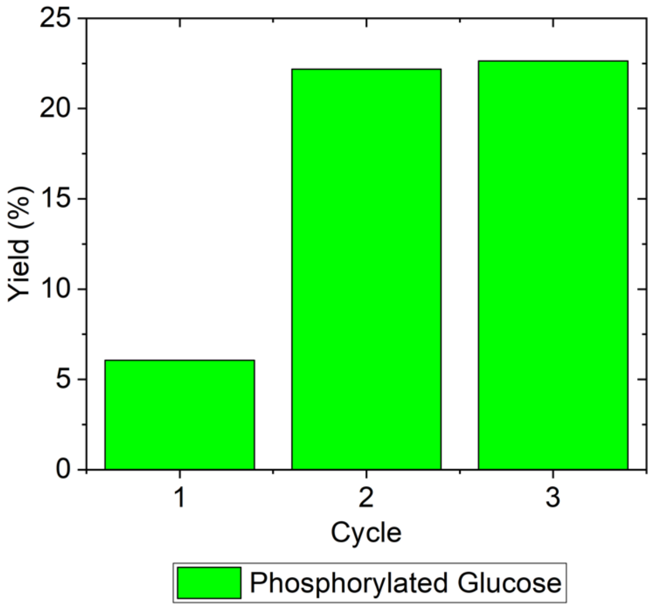

Supplementary Figure 257: Yield of glucose-5-phosphate after each cycle for the reaction with 150 mM of D-ribose, 450 mM potassium cyanate and 20 mM sodium phosphate in 1.0 M imidazole buffer.

A spiking experiment with genuine samples of glucose-1-phosphate and glucose-6-phosphate which confirmed the formation of glucose-6-phosphate and three other phosphorylated glucose species (Supplementary Figure 258).

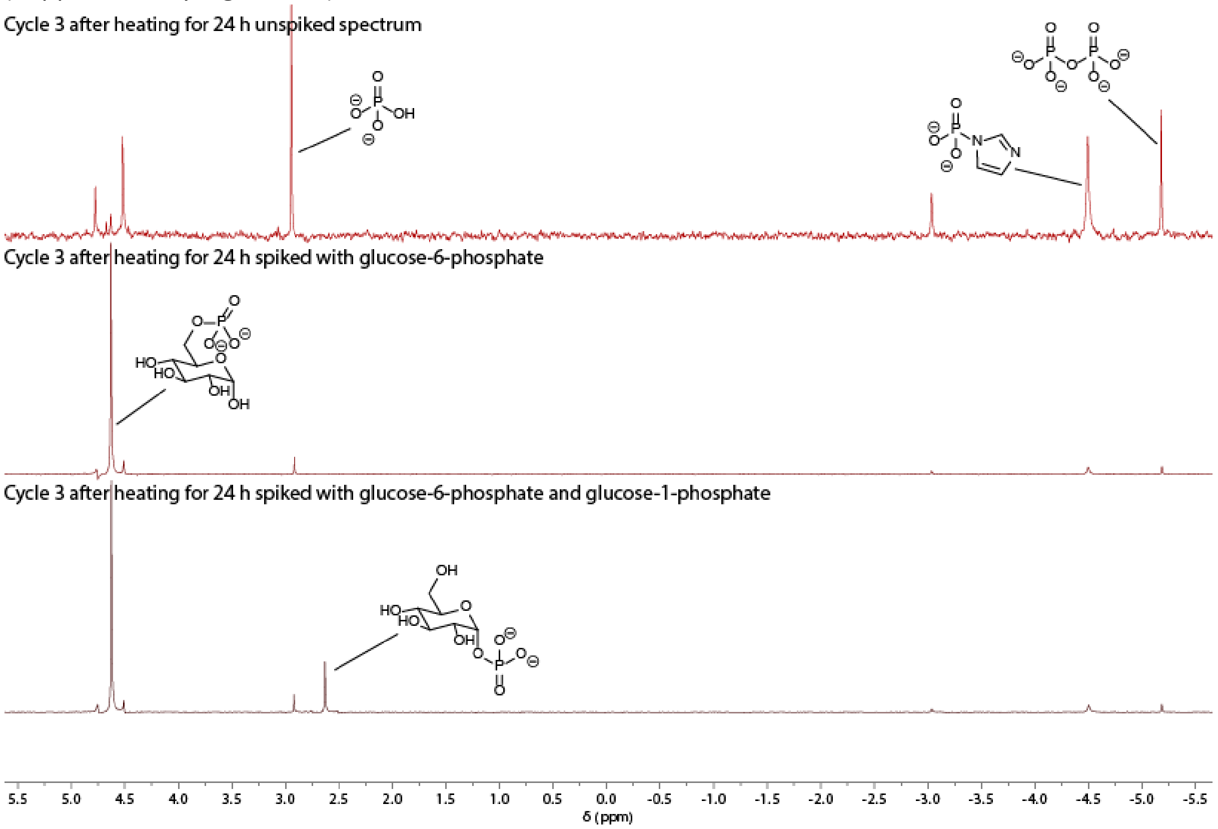

Supplementary Figure 258: ^{31}P NMR spectra of a spiking experiment for the third cycle of the reaction 150 mM of D-glucose, 450 mM potassium cyanate and 20 mM sodium phosphate in 1.0 M imidazole buffer. Top spectrum is unspiked. Bottom spectrum is with the addition of genuine glucose-6-phosphate.

S7.8 Phosphorylation of adenosine with orthophosphate recycling

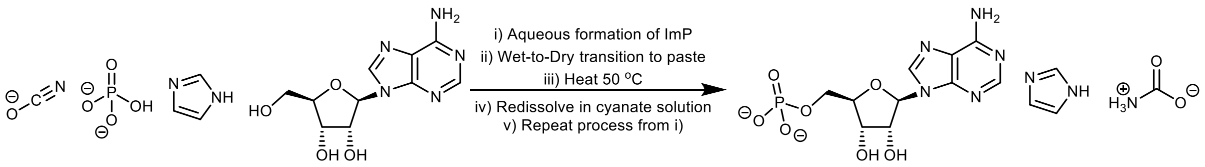

S7.8.1 Phosphorylation of adenosine with 50 mM orthophosphate

This reaction was performed according to the procedure in Section S7.1 but with 750 mM of adenosine (802 mg, 3.0 mmol) and 50 mM sodium phosphate dibasic dihydrate salt (178.0 mg, 1.0 mmol).

A total of three cycles were performed. Formation of imidazole phosphate was observed in solution and remained present after drying the solution to a paste. Formation of adenosine-5'-monophosphate occurred during both the drying to the paste over a period of ~24 h and during the heating of the paste to 50 °C for ~24 h (Supplementary Figure 259, 260 and 261).

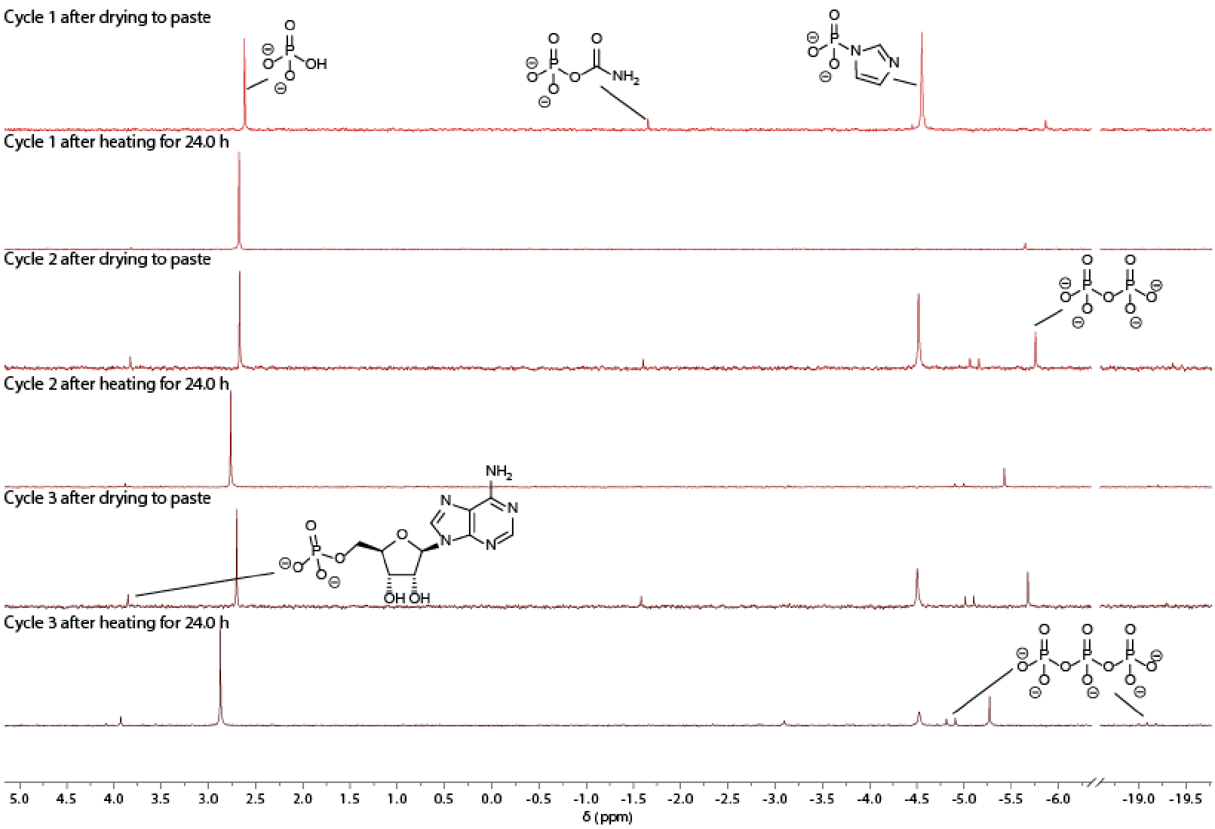

Supplementary Figure 259: Representative ^{31}P NMR spectra for the three cycles with 750 mM of adenosine, 450 mM potassium cyanate and 50 mM sodium phosphate in 1.0 M imidazole buffer. For each cycle the ^{31}P NMR spectrum after the reaction had been dried to a paste and the ^{31}P NMR spectrum after heating the reaction to 50 °C for ~24 h are shown. All NMR spectra were measured in a 0.5 M citric acid buffer at pH 6.85 9 : 1 H_2O : D_2O. The chemical shift of the pyrophosphate peak is variable at pH 6.85 due to the pyrophosphate pK_a = 6.6.

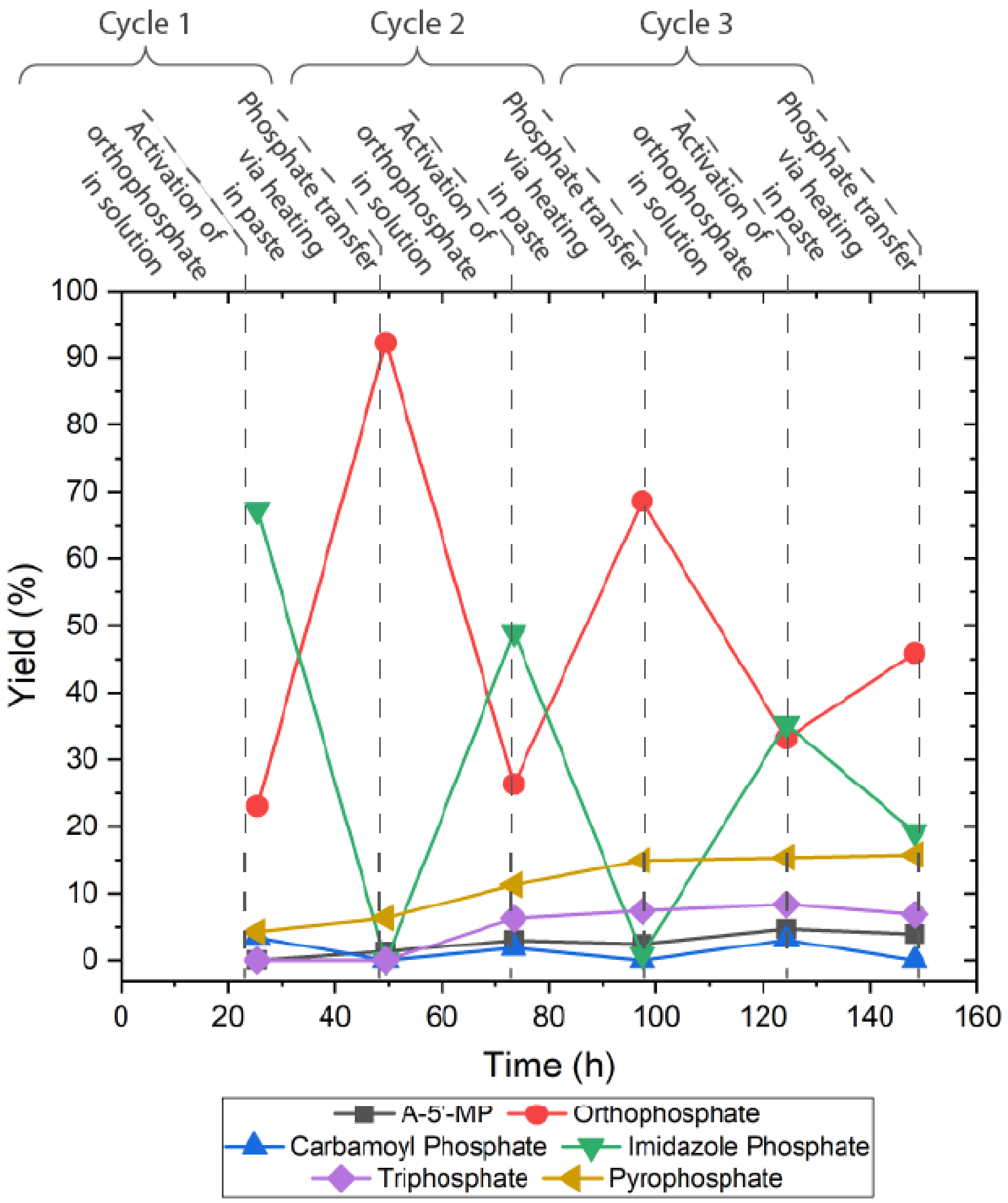

Supplementary Figure 260: Changes in yield for all phosphorous species over the course of three cycles for the reaction with 750 mM of adenosine, 450 mM potassium cyanate and 50 mM sodium phosphate in 1.0 M imidazole buffer.

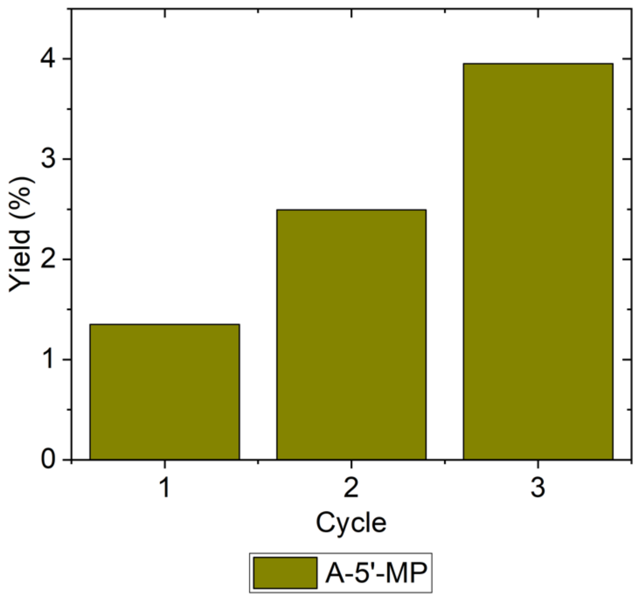

Supplementary Figure 261: Yield of adenosine-5’-monophosphate after each cycle for the reaction with 750 mM adenosine, 450 mM potassium cyanate and 50 mM sodium phosphate in 1.0 M imidazole buffer.

A spiking experiment with a genuine sample of adenosine-5’-monophosphate confirmed its formation (Supplementary Figure 262).

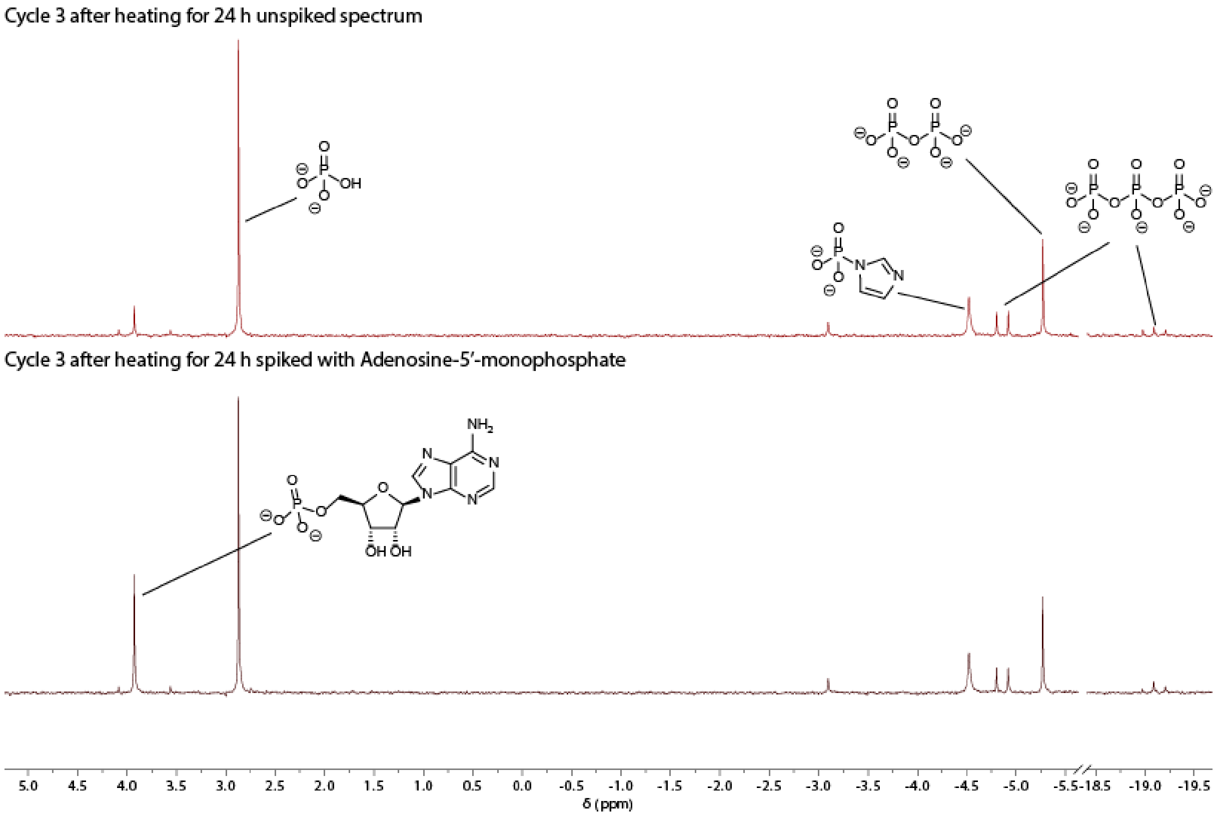

Supplementary Figure 262: ^{31}P NMR spectra of a spiking experiment for the third cycle of the reaction with 750 mM of adenosine, 450 mM potassium cyanate and 50 mM sodium phosphate in 1.0 M imidazole buffer. Top spectrum is unspiked. Bottom spectrum is with the addition of genuine adenosine-5’-monophosphate.

S7.8.2 Phosphorylation of adenosine with 20 mM orthophosphate

This reaction was performed according to the procedure in Section S7.1 but with 750 mM of adenosine (802 mg, 3.0 mmol) and 20 mM sodium phosphate dibasic dihydrate salt (71.2 mg, 0.4 mmol).

A total of three cycles were performed. Formation of imidazole phosphate was observed in solution and remained present after drying the solution to a paste. Formation of adenosine-5'-monophosphate occurred during both the drying to the paste over a period of ~24 h and during the heating of the paste to 50 °C for ~24 h (Supplementary Figure 263, 264 and 265).

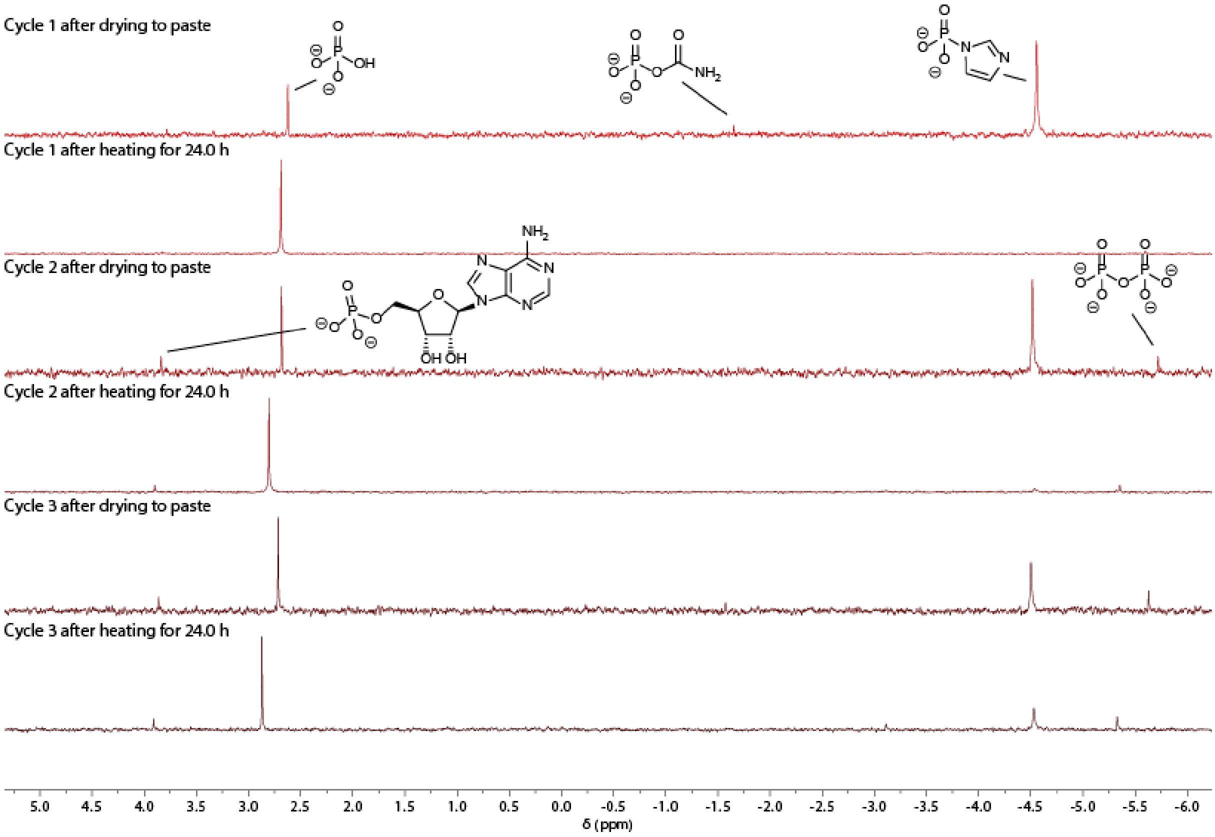

Supplementary Figure 263: Representative ^{31}P NMR spectra for the three cycles with 750 mM of adenosine, 450 mM potassium cyanate and 20 mM sodium phosphate in 1.0 M imidazole buffer. For each cycle the ^{31}P NMR spectrum after the reaction had been dried to a paste and the ^{31}P NMR spectrum after heating the reaction to 50 °C for ~24 h are shown. All NMR spectra were measured in a 0.5 M citric acid buffer at pH 6.85 9 : 1 H_2O : D_2O. The chemical shift of the pyrophosphate peak is variable at pH 6.85 due to the pyrophosphate pK_a = 6.6.

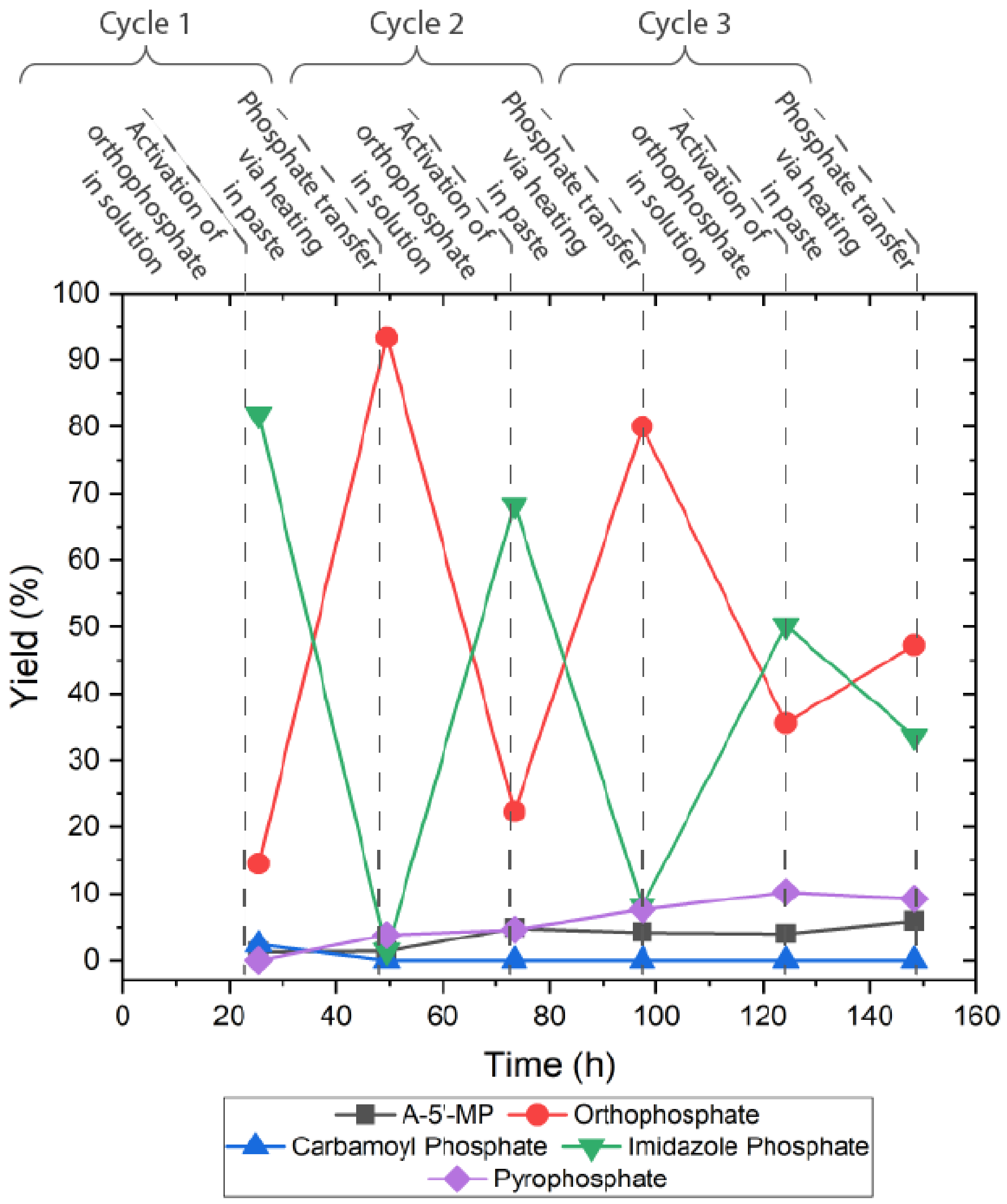

Supplementary Figure 264: Changes in yield for all phosphorous species over the course of three cycles for the reaction with 750 mM of adenosine, 450 mM potassium cyanate and 20 mM sodium phosphate in 1.0 M imidazole buffer.

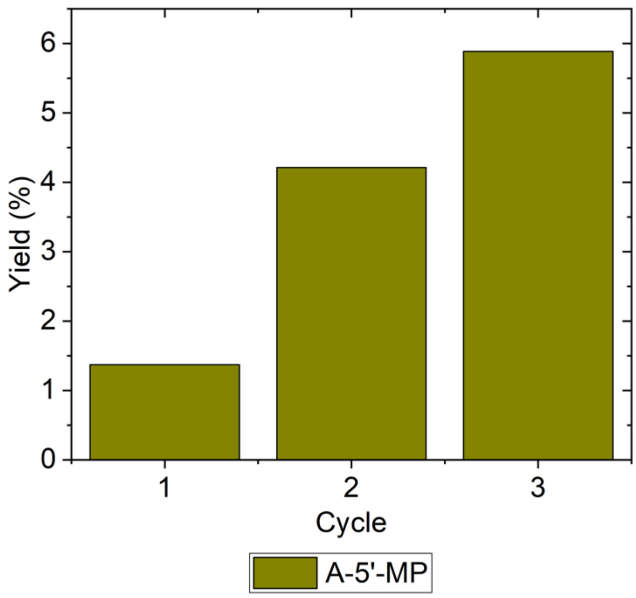

Supplementary Figure 265: Yield of adenosine-5'-monophosphate after each cycle for the reaction with 750 mM of adenosine, 450 mM potassium cyanate and 20 mM sodium phosphate in 1.0 M imidazole buffer.

A spiking experiment with a genuine sample of adenosine-5'-monophosphate confirmed its formation (Supplementary Figure 266).

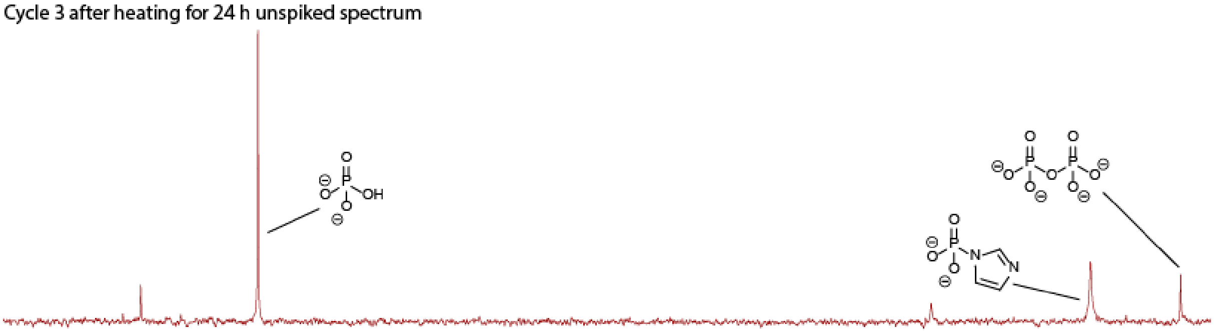

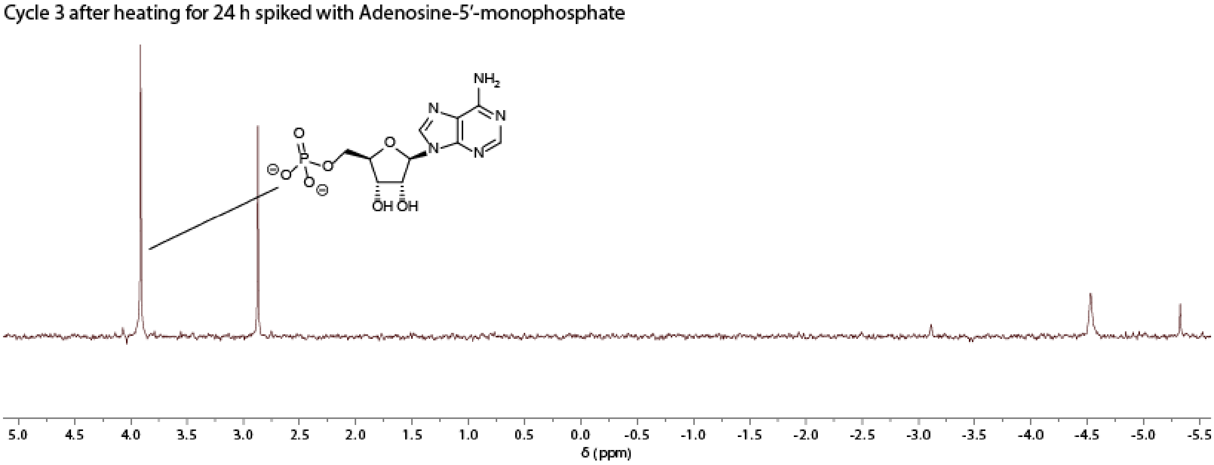

Supplementary Figure 266: ^{31}P NMR spectra of a spiking experiment for the third cycle of the reaction 750 mM of adenosine, 450 mM potassium cyanate and 20 mM sodium phosphate in 1.0 M imidazole buffer. Top spectrum is unspiked. Bottom spectrum is with the addition of genuine adenosine-5'-monophosphate.

S7.9 Phosphorylation of cytidine with orthophosphate recycling

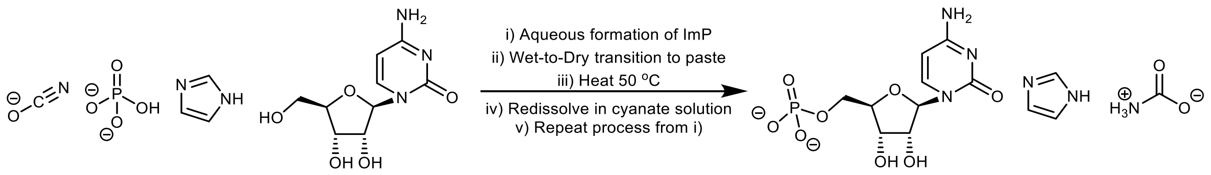

S7.9.1 Phosphorylation of cytidine with 50 mM orthophosphate

This reaction was performed according to the procedure in Section S7.1 but with 750 mM of cytidine (730 mg, 3.0 mmol) and 50 mM sodium phosphate dibasic dihydrate salt (178.0 mg, 1.0 mmol).

A total of three cycles were performed. Formation of imidazole phosphate was observed in solution and remained present after drying the solution to a paste. Formation of cytidine-5'-monophosphate, cytidine-2'-monophosphate and cytidine-3'-monophosphate occurred during both the drying to the paste over a period of ~24 h and during the heating of the paste to 50 °C for ~24 h (Supplementary Figure 267, 268 and 269).

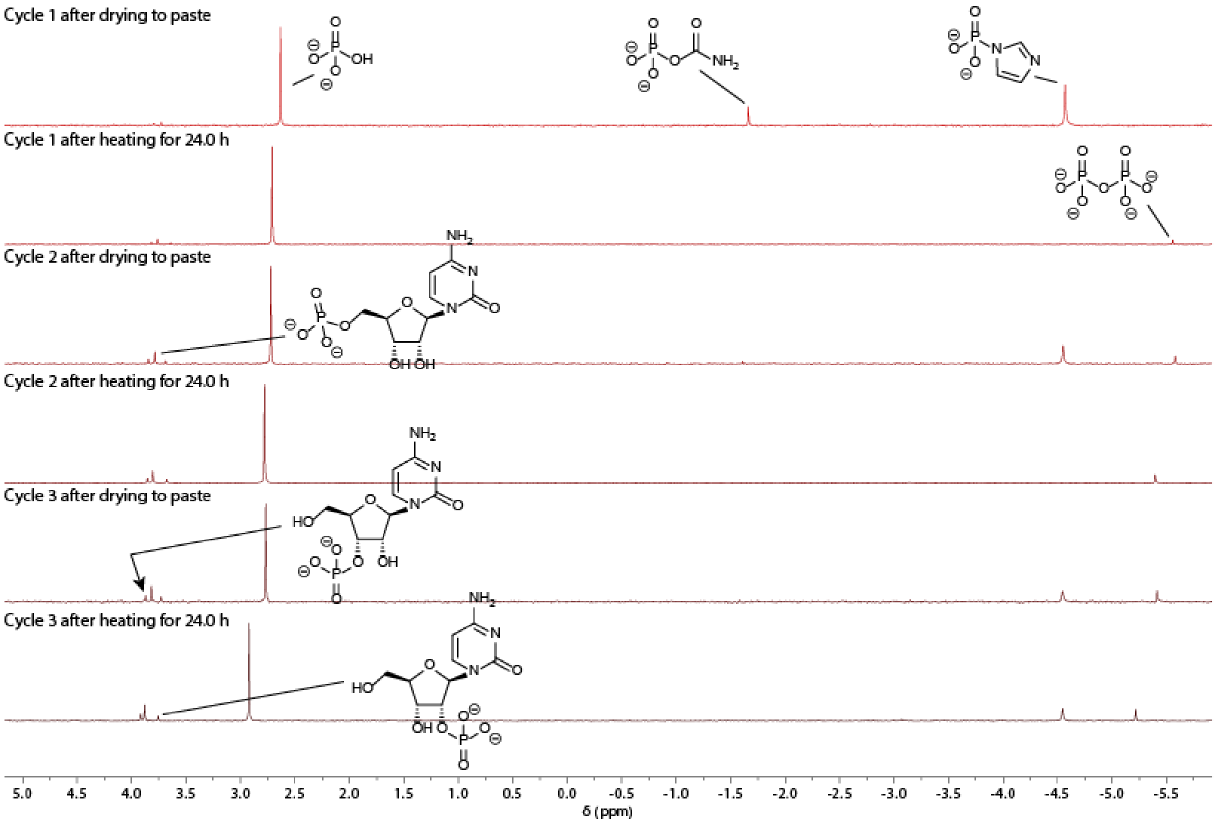

Supplementary Figure 267: Representative ^{31}P NMR spectra for the three cycles with 750 mM of cytidine, 450 mM potassium cyanate and 50 mM sodium phosphate in 1.0 M imidazole buffer. For each cycle the ^{31}P NMR spectrum after the reaction had been dried to a paste and the ^{31}P NMR spectrum after heating the reaction to 50 °C for ~24 h are shown. All NMR spectra were measured in a 0.5 M citric acid buffer at pH 6.85 9 : 1 H_2O : D_2O. The chemical shift of the pyrophosphate peak is variable at pH 6.85 due to the pyrophosphate pK_a = 6.6.

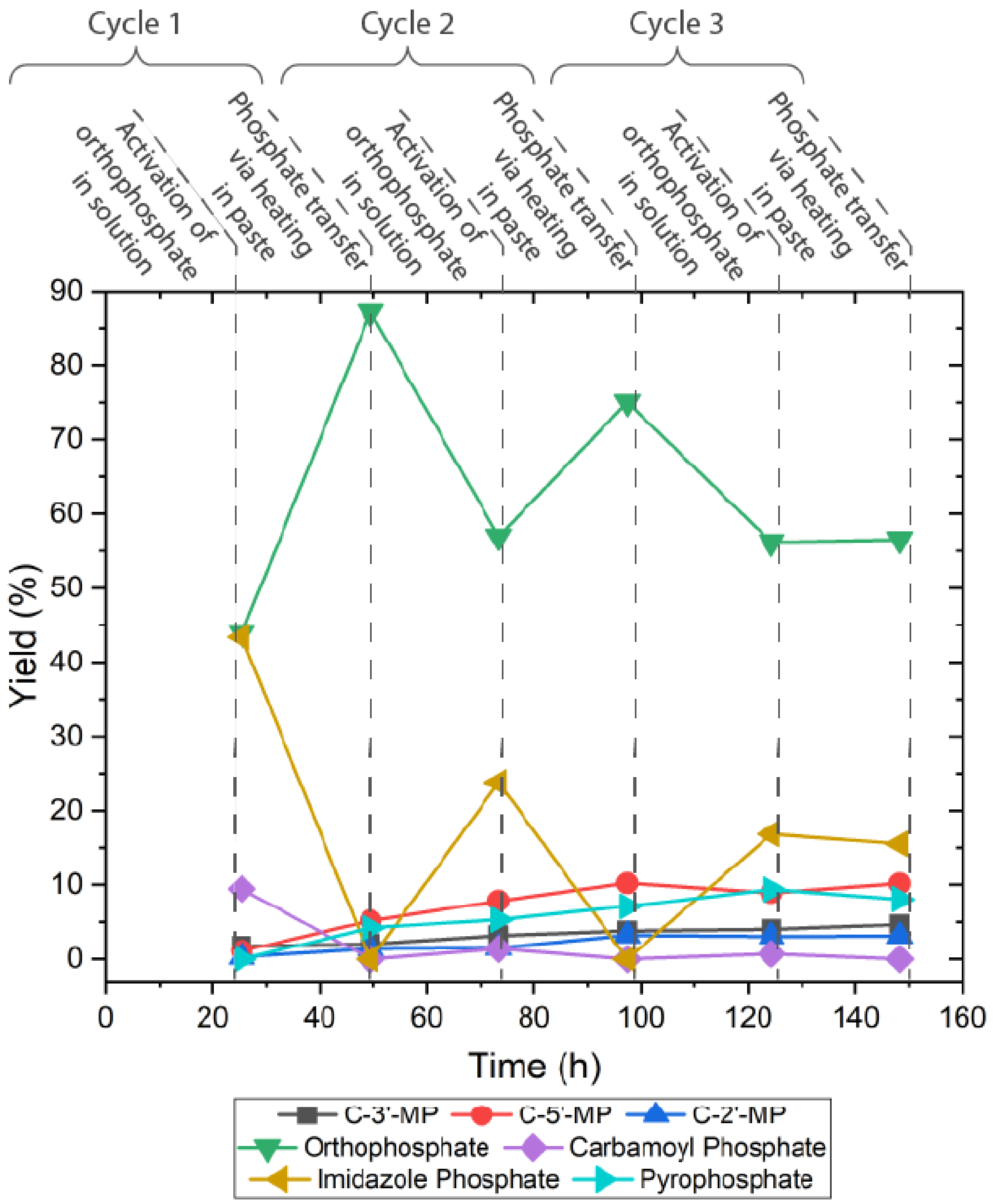

Supplementary Figure 268: Changes in yield for all phosphorous species over the course of three cycles for the reaction with 750 mM of cytidine, 450 mM potassium cyanate and 50 mM sodium phosphate in 1.0 M imidazole buffer.

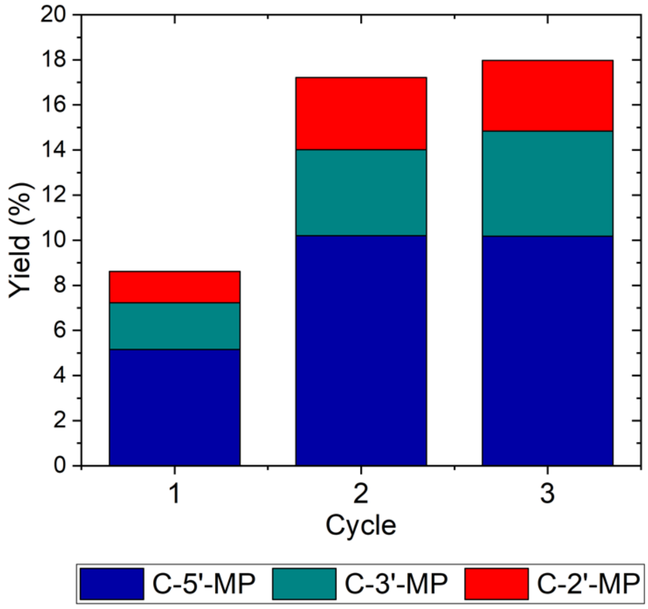

Supplementary Figure 269: Yield of cytidine-5’-monophosphate, cytidine-2’-monophosphate and cytidine-3’-monophosphate after each cycle for the reaction with 750 mM cytidine, 450 mM potassium cyanate and 50 mM sodium phosphate in 1.0 M imidazole buffer.

A spiking experiment with a genuine sample of cytidine-5’-monophosphate, cytidine-2’-monophosphate and cytidine-3’-monophosphate confirmed their formation (Supplementary Figure 270).

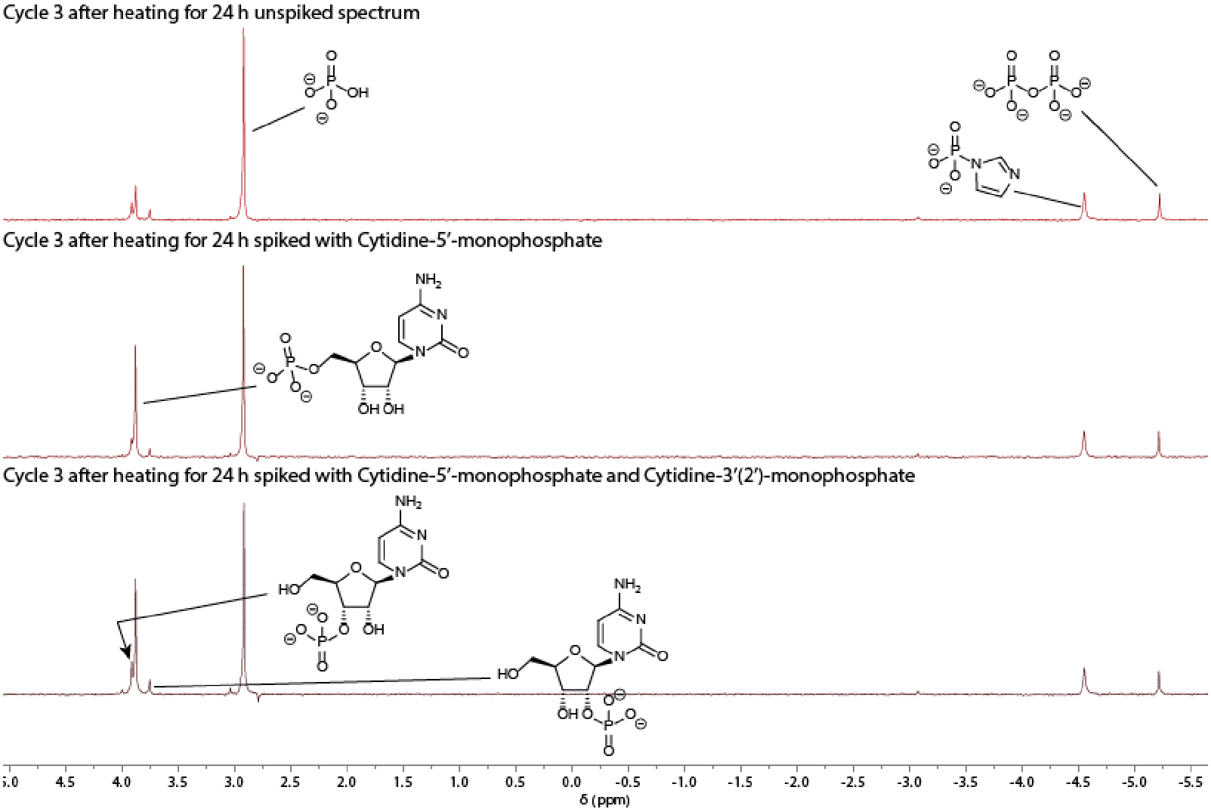

Supplementary Figure 270: ^{31}P NMR spectra of a spiking experiment for the third cycle of the reaction with 750 mM of cytidine, 450 mM potassium cyanate and 50 mM sodium phosphate in 1.0 M imidazole buffer. Top spectrum is unspiked. Middle spectrum is spiked with genuine cytidine-5’-monophosphate. Bottom spectrum is spiked with genuine cytidine-3’(2’)-monophosphate.

S7.9.2 *Phosphorylation of cytidine with 20 mM orthophosphate*

This reaction was performed according to the procedure in Section S7.1 but with 750 mM of cytidine (802 mg, 3.0 mmol) and 20 mM sodium phosphate dibasic dihydrate salt (71.2 mg, 0.4 mmol).

A total of three cycles were performed. Formation of imidazole phosphate was observed in solution and remained present after drying the solution to a paste. Formation of cytidine-5'-monophosphate, cytidine-2'-monophosphate and cytidine-3'-monophosphate occurred during both the drying to the paste over a period of ~24 h and during the heating of the paste to 50 °C for ~24 h (Supplementary Figure 271, 272 and 273).

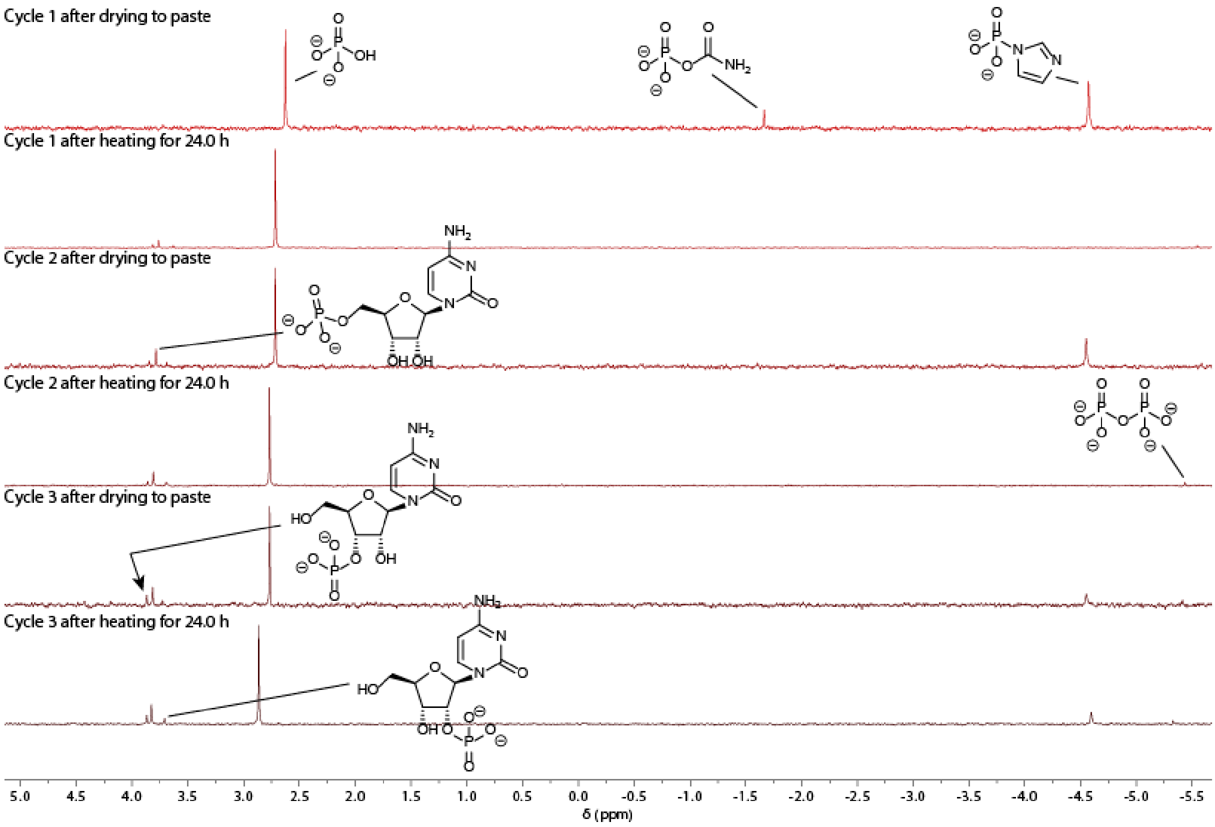

Supplementary Figure 271: Representative ^{31}P NMR spectra for the three cycles with 750 mM of cytidine, 450 mM potassium cyanate and 20 mM sodium phosphate in 1.0 M imidazole buffer. For each cycle the ^{31}P NMR spectrum after the reaction had been dried to a paste and the ^{31}P NMR spectrum after heating the reaction to 50 °C for ~24 h are shown. All NMR spectra were measured in a 0.5 M citric acid buffer at pH 6.85 9 : 1 H_2O : D_2O. The chemical shift of the pyrophosphate peak is variable at pH 6.85 due to the pyrophosphate pK_a = 6.6.

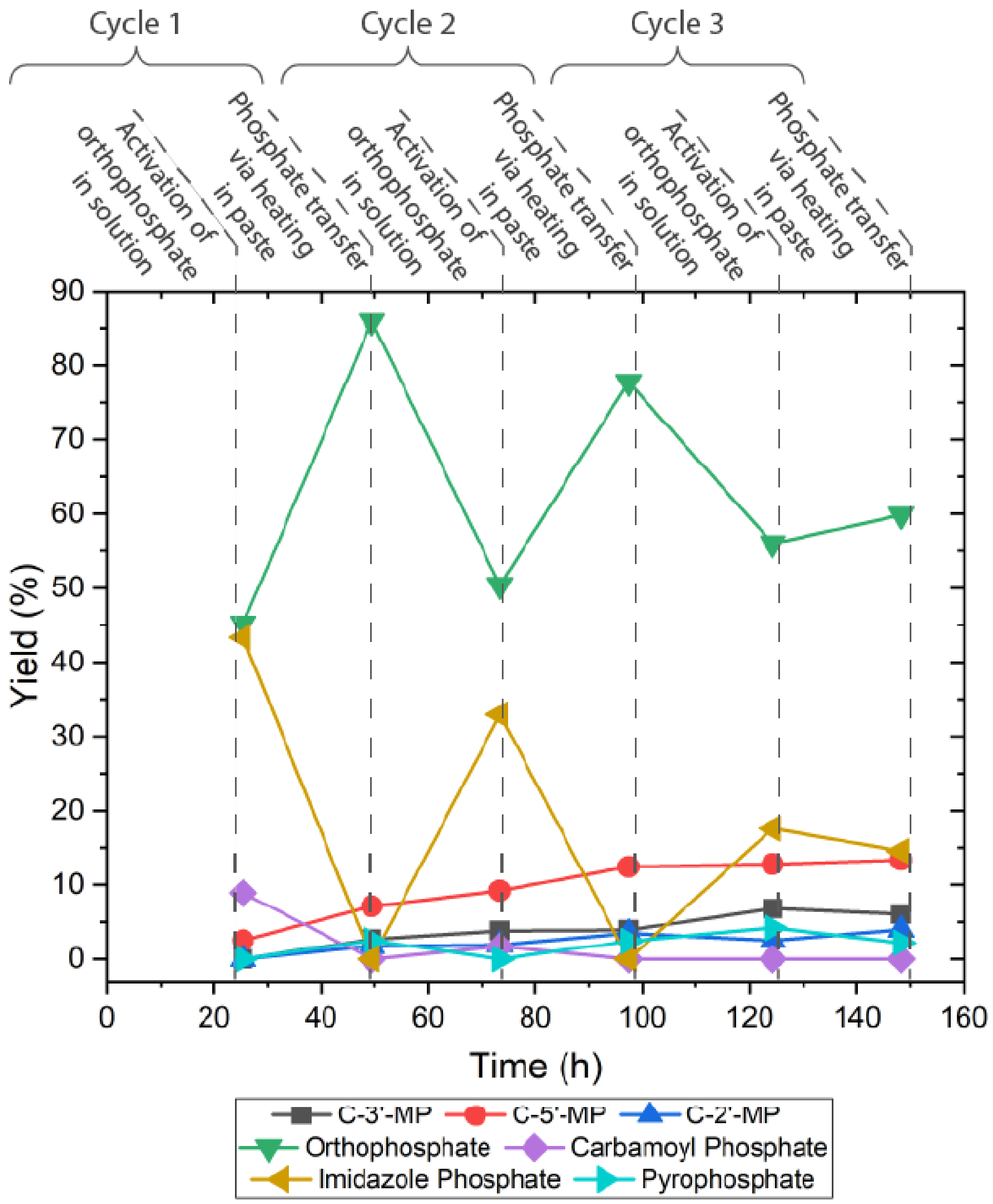

Supplementary Figure 272: Changes in yield for all phosphorous species over the course of three cycles for the reaction with 750 mM of cytidine, 450 mM potassium cyanate and 20 mM sodium phosphate in 1.0 M imidazole buffer.

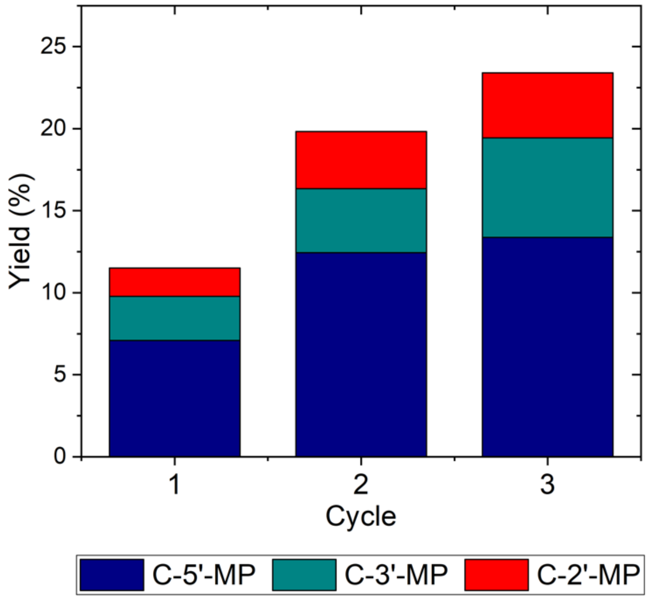

Supplementary Figure 273: Yield of cytidine-5'-monophosphate, cytidine-2'-monophosphate and cytidine-3'-monophosphate after each cycle for the reaction with 750 mM of cytidine, 450 mM potassium cyanate and 20 mM sodium phosphate in 1.0 M imidazole buffer.

A spiking experiment with a genuine sample of cytidine-5'-monophosphate, cytidine-2'-monophosphate and cytidine-3'-monophosphate confirmed their formation (Supplementary Figure 274).

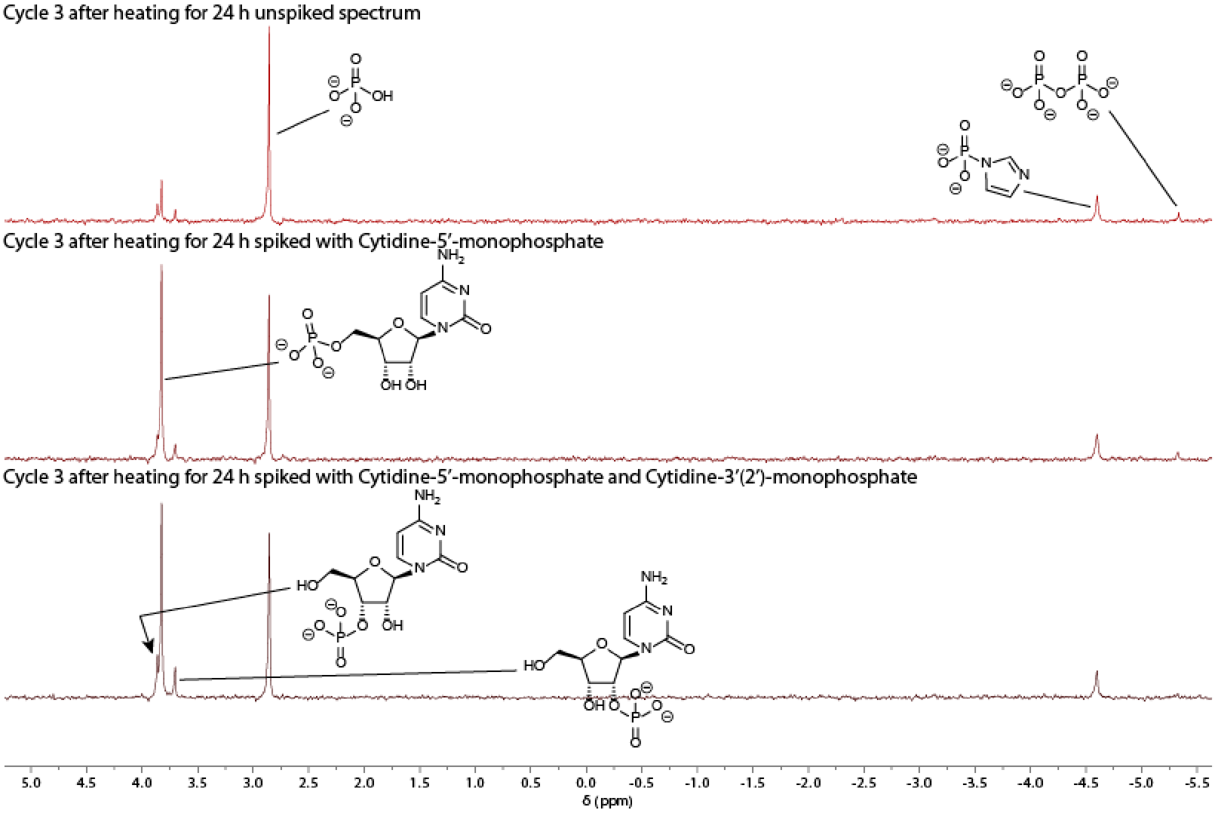

Supplementary Figure 274: ^{31}P NMR spectra of a spiking experiment for the third cycle of the reaction 750 mM of cytidine, 450 mM potassium cyanate and 20 mM sodium phosphate in 1.0 M imidazole buffer. Top spectrum is unspiked. Middle spectrum is spiked with genuine cytidine-5'-monophosphate. Bottom spectrum is spiked with genuine cytidine-3'(2')-monophosphate.

7.10 Control reactions

Control reactions were performed in the absence of imidazole and cyanate in order to demonstrate that the phosphorylation which occurred proceeded via imidazole phosphate and not via carbamoyl phosphate.
The reaction was kept in solution for 1 day before drying to a paste.

S7.10.1 Supplementary experimental method for control reactions in the absence of imidazole

1.460 g (18.0 mmol) of potassium cyanate and 1.068 g (6 mmol) of sodium phosphate dibasic were dissolved in 40.0 mL of 9 : 1 H_2O : D_2O to give a 450 mM potassium cyanate and a 150 mM sodium phosphate solution.
277 mg (3.0 mmol) of glycerol was dissolved in 4.0 mL of 450 mM potassium cyanate and a 150 mM sodium phosphate solution to give a 750 mM glycerol solution in a 15 mL Falcon tube. The pH of the solution was adjusted to 7.3 with 5 M hydrochloric acid and 5 M sodium hydroxide solution. 0.5 mL of the solution was placed into an NMR tube and the reaction was followed with ^{31}P-NMR and ^{1}H-NMR spectroscopy.
After 24 h the solution in the Falcon tube and the solution in the NMR tube were recombined and pipetted into a petri dish (10 cm diameter). The solution was added in small droplets in order to increase the surface area and increase the rate of evaporation. The solution was left to dry for 48 h at 22 °C in a fume hood to ensure continuous air flow across the petri dish.
A ~20 mg sample of the resulting paste was removed and dissolved in 0.5 mL of a 0.5 M citric acid buffer pH 6.85 9 : 1 H_2O : D_2O solution and analysed by ^{31}P-NMR and ^{1}H-NMR spectroscopy. The remainder of the resulting paste was scraped from the petri dish into a 2.5 mL Eppendorf. The Eppendorf was then spun on a centrifuge for 3 min at 5590 g in order to move all solid to the bottom of the Eppendorf and compact it together. The Eppendorf was then placed on a thermoshaker at 50 °C and 600 rpm for 18.1 h. A ~20 mg sample of the paste was removed after 24 h and was dissolved in 0.5 mL of a 0.5 M citric acid buffer pH 6.85 9 : 1 H_2O : D_2O solution and analysed by ^{31}P-NMR and ^{1}H-NMR spectroscopy.

S7.10.2 Control reaction in the absence of imidazole with glycerol

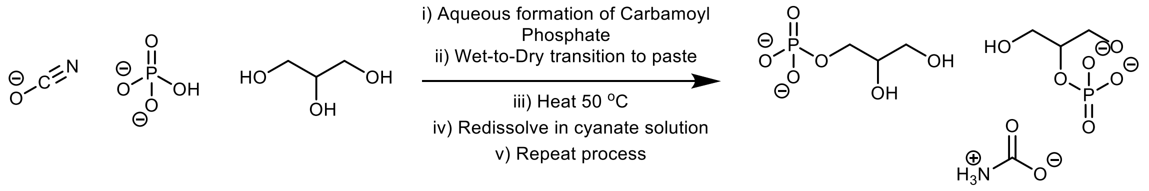

This reaction was performed according to the procedure detailed in Section S7.10.1.

Formation of carbamoyl phosphate was observed in solution (Supplementary Figure 275). However, no phosphorylation of the glycerol occurred in the paste reaction (Supplementary Figure 275).

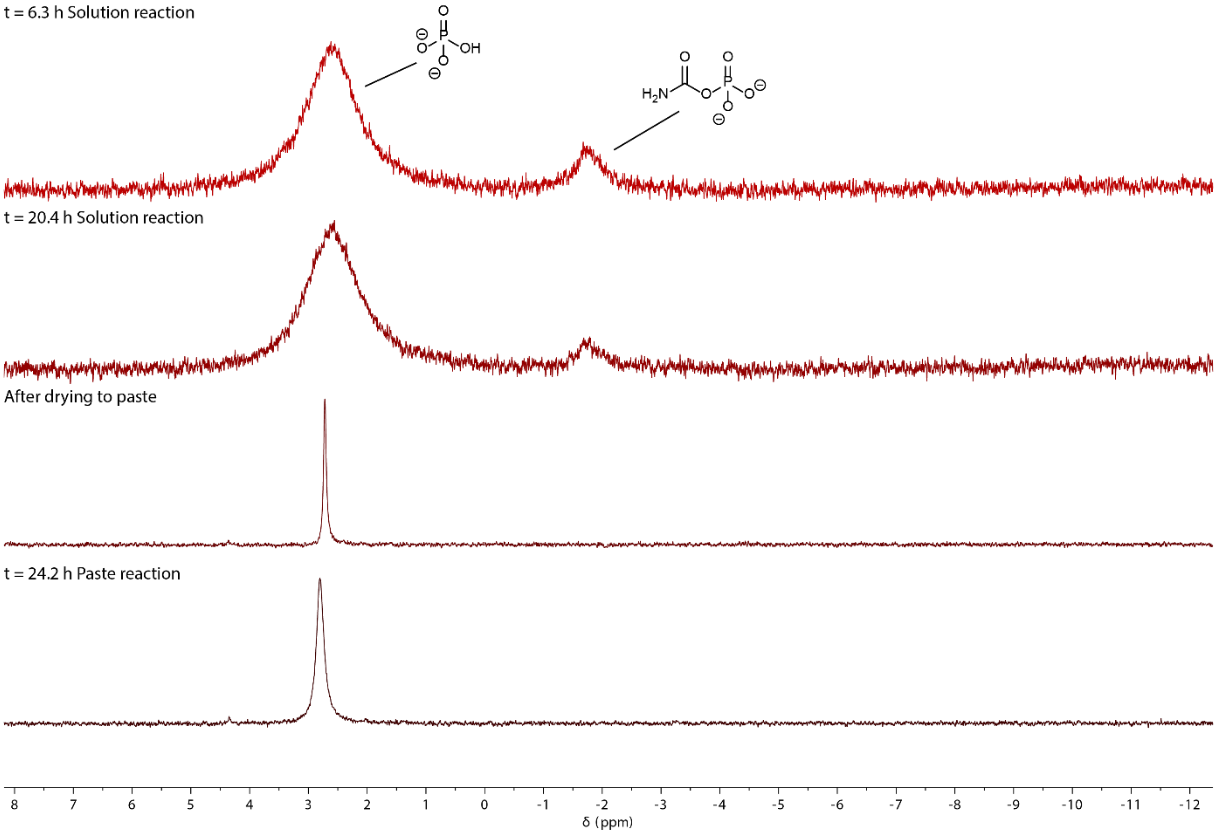

Supplementary Figure 275: Representative ^{31}P NMR spectra over time for the reaction of 750 mM (3.0 mmol) glycerol and 450 mM (1.8 mmol) potassium cyanate and 150 mM (0.6 mmol) sodium phosphate dibasic. No imidazole was added to the experiment. The top two spectra were for the reaction in solution. In the solution reactions the broadening of the ^{31}P peak for orthophosphate and carbamoyl phosphate occurs as a result of the solvent being a water : glycerol mixture. The bottom two spectra were taken after evaporation to form a paste and after heating the paste at 50 °C for 24.2 h.

S7.10.3 Control reaction in the absence of imidazole with ribose

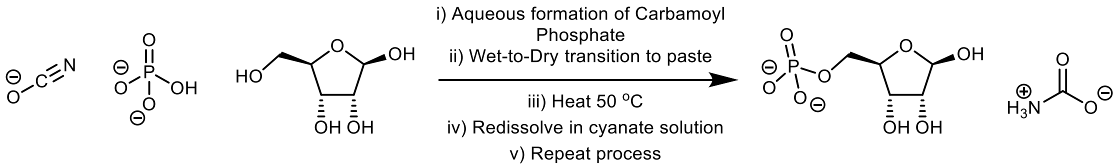

This reaction was performed using an identical procedure as detailed in Section S.7.10.1 but with the addition of 450 mg (3.0 mmol) ribose instead of glycerol. The pH of the solution increased significantly to pH 11.4 after 20.9 h and 5 M hydrochloric acid was added to reduce the pH to 7.5. The increase in pH is believed to come from the ribose catalysing the hydrolysis of the cyanate which releases ammonia and therefore increases the pH.

Formation of carbamoyl phosphate was observed in solution (Supplementary Figure 276). However, no phosphorylation of the ribose occurred in the paste reaction (Supplementary Figure 276).

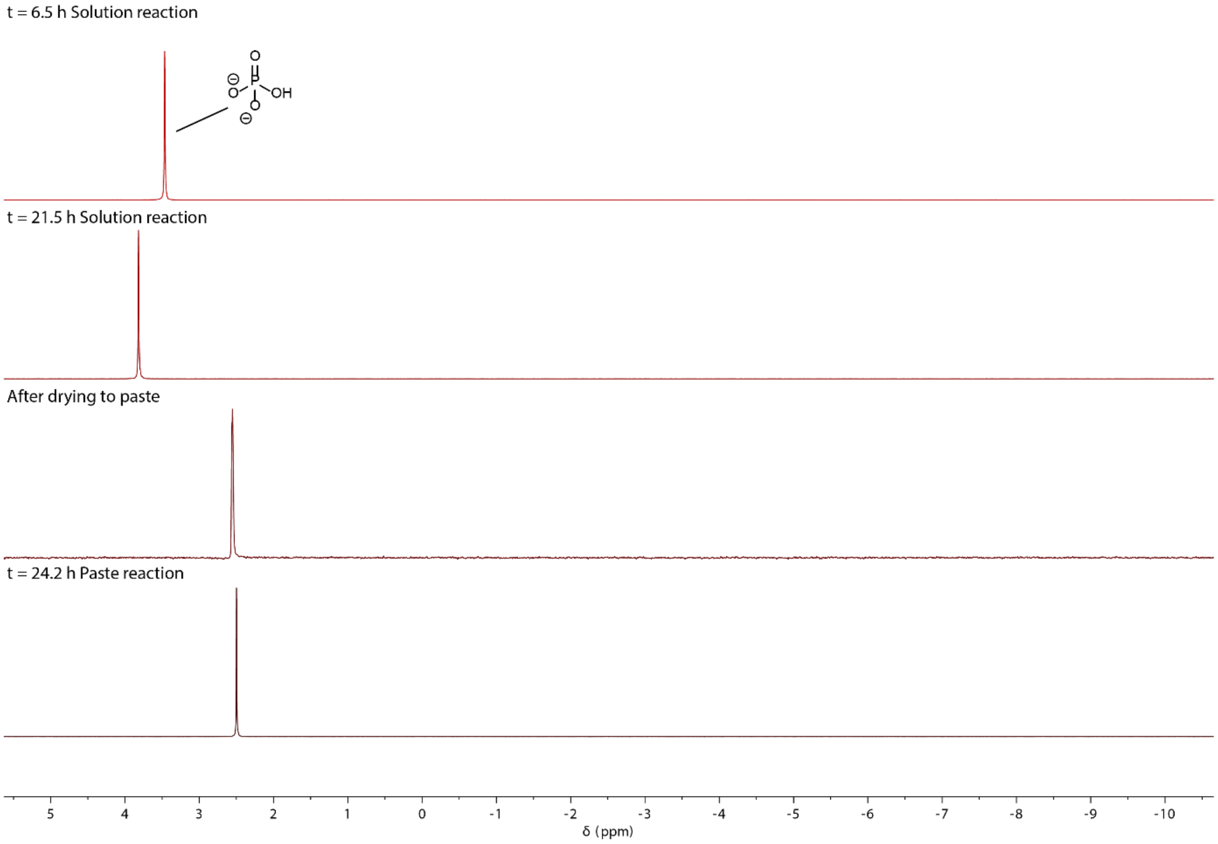

Supplementary Figure 276: Representative ^{31}P NMR spectra over time for the reaction of 750 mM (3.0 mmol) ribose and 450 mM (1.8 mmol) potassium cyanate and 150 mM (0.6 mmol) sodium phosphate dibasic. No imidazole was added to the experiment. The top two spectra were for the reaction in solution. The bottom two spectra were taken after evaporation to form a paste and after heating the paste at 50 °C for 24.2 h. The change in the chemical shift of the orthophosphate peak between spectra are due to differences in pH between the solutions.

S7.10.4 Control reaction in the absence of imidazole with calcium glycerate

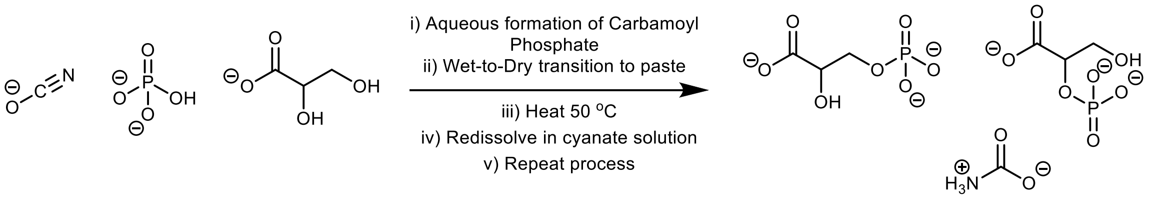

This reaction was performed using an identical procedure as detailed in Section S.7.10.1 but with the addition of 375 mg (1.5 mM - there are two glycerate molecules per Ca^{2+} ion and therefore half the moles were used relative to the other control experiments in this section) calcium glycerate instead of glycerol. Given the high concentration of Ca^{2+} in this reaction precipitation of calcium phosphate was observed which is why the phosphate integrals in the solution reaction in Supplementary Figure 277 are smaller compared to other experiments in this section.

Formation of carbamoyl phosphate was observed in solution (Supplementary Figure 277). However, no phosphorylation of the calcium glycerate occurred in the paste reaction and a minor amount of pyrophosphate was observed to form (Supplementary Figure 277).

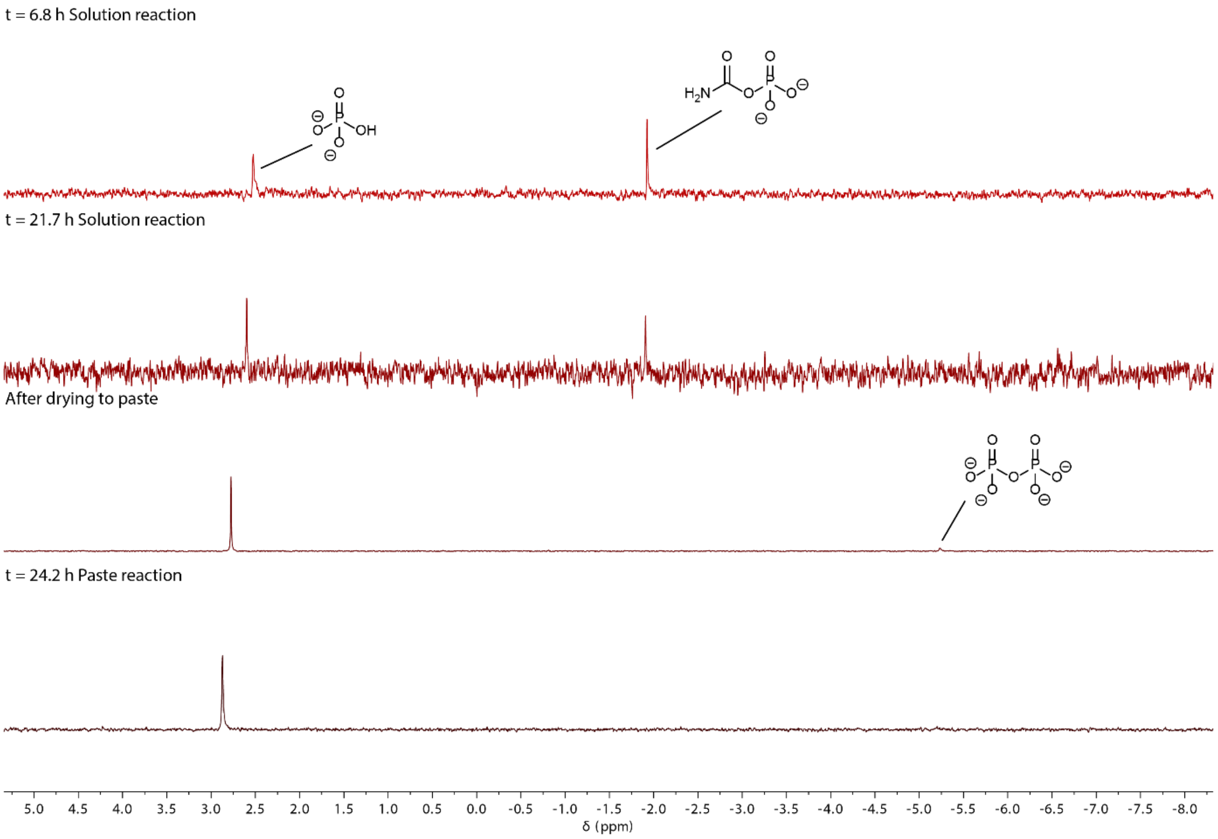

Supplementary Figure 277: Representative ^{31}P NMR spectra over time for the reaction of 750 mM (3.0 mmol) calcium glycerate and 450 mM (1.8 mmol) potassium cyanate and 150 mM (0.6 mmol) sodium phosphate dibasic. No imidazole was added to the experiment. The top two spectra were for the reaction in solution. The bottom two spectra were taken after evaporation to form a paste and after heating the paste at 50 °C for 24.2 h.

S7.10.5 *Control reaction in the absence of imidazole with serine methyl ester*

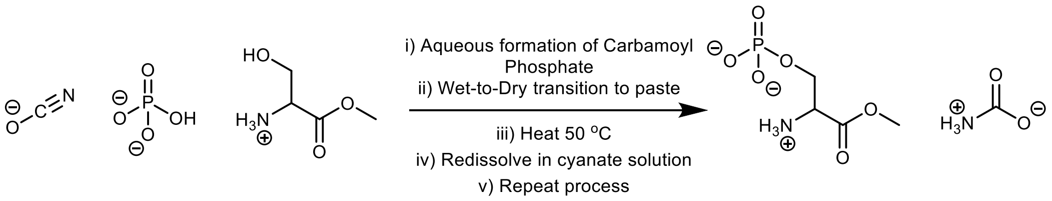

This reaction was performed using an identical procedure as detailed in Section S.7.10.1 but with the addition of 467 mg (3.0 mM) serine methyl ester·HCl instead of glycerol.

Formation of carbamoyl phosphate was observed in solution (Supplementary Figure 278). However, no phosphorylation of the serine methyl ester occurred in the paste reaction (Supplementary Figure 278).

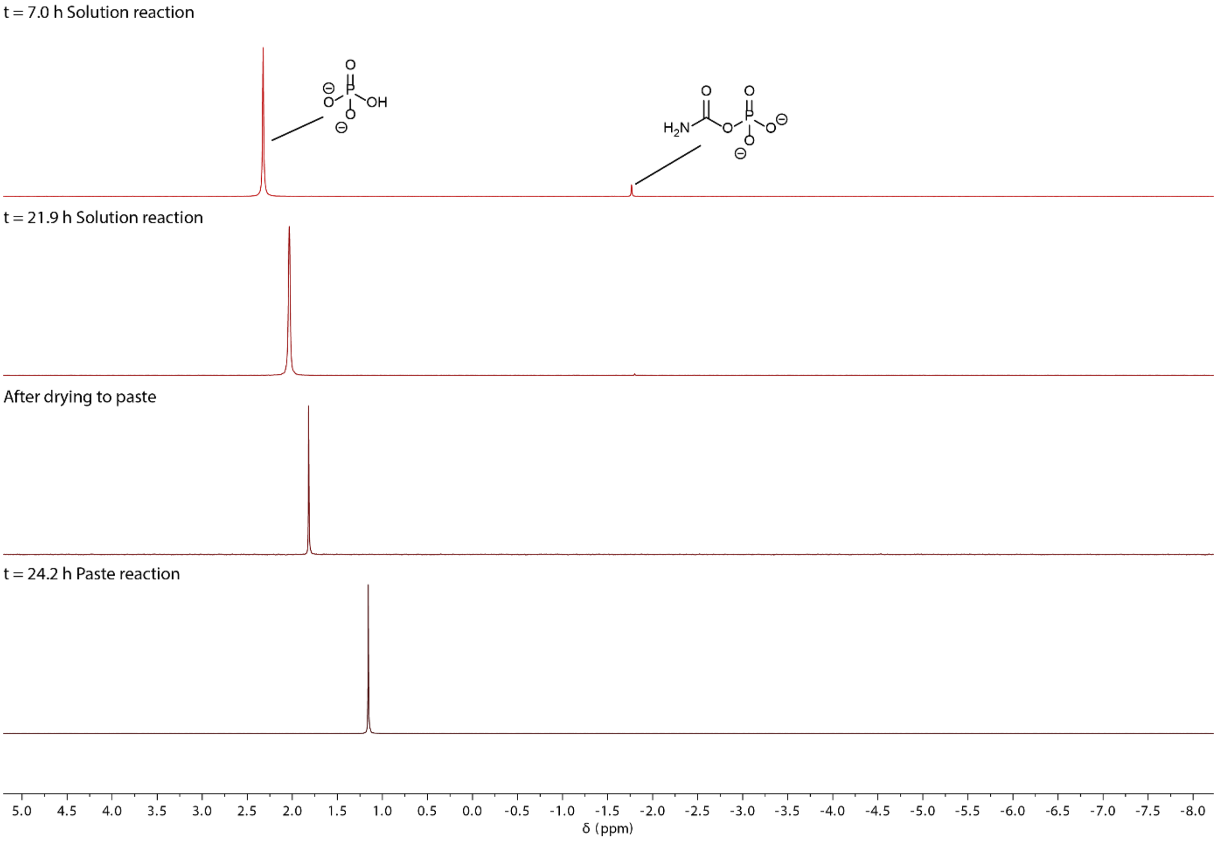

Supplementary Figure 278: Representative ^{31}P NMR spectra over time for the reaction of 750 mM (3.0 mmol) serine methyl ester and 450 mM (1.8 mmol) potassium cyanate and 150 mM (0.6 mmol) sodium phosphate dibasic. No imidazole was added to the experiment. The top two spectra were for the reaction in solution. The bottom two spectra were taken after evaporation to form a paste and after heating the paste at 50 °C for 24.2 h. The changes in the chemical shift of the orthophosphate peak between spectra are due to differences in pH between the solutions.

S7.10.6 Control reaction in the absence of imidazole with adenosine-5’-monophosphate

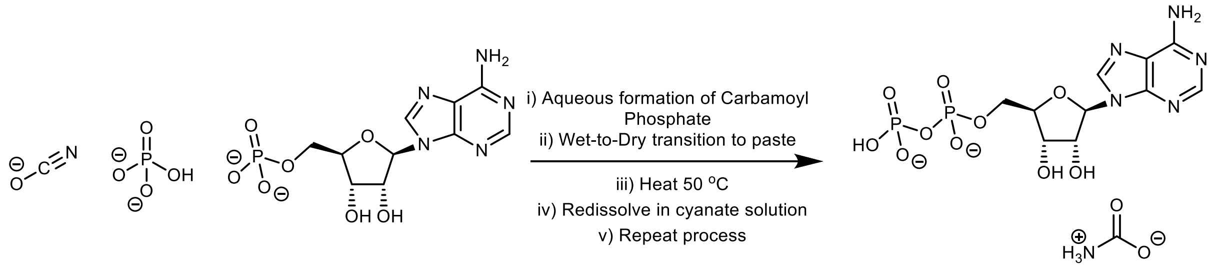

This reaction was performed using an identical procedure as detailed in Section S.7.10.1 but with the addition of 293.4 mg (0.75 mmol) adenosine-5’-monophosphate instead of glycerol.

Formation of carbamoyl phosphate was observed in solution (Supplementary Figure 279). However, no phosphorylation of the adenosine-5’-monophosphate occurred in the paste reaction (Supplementary Figure 279).

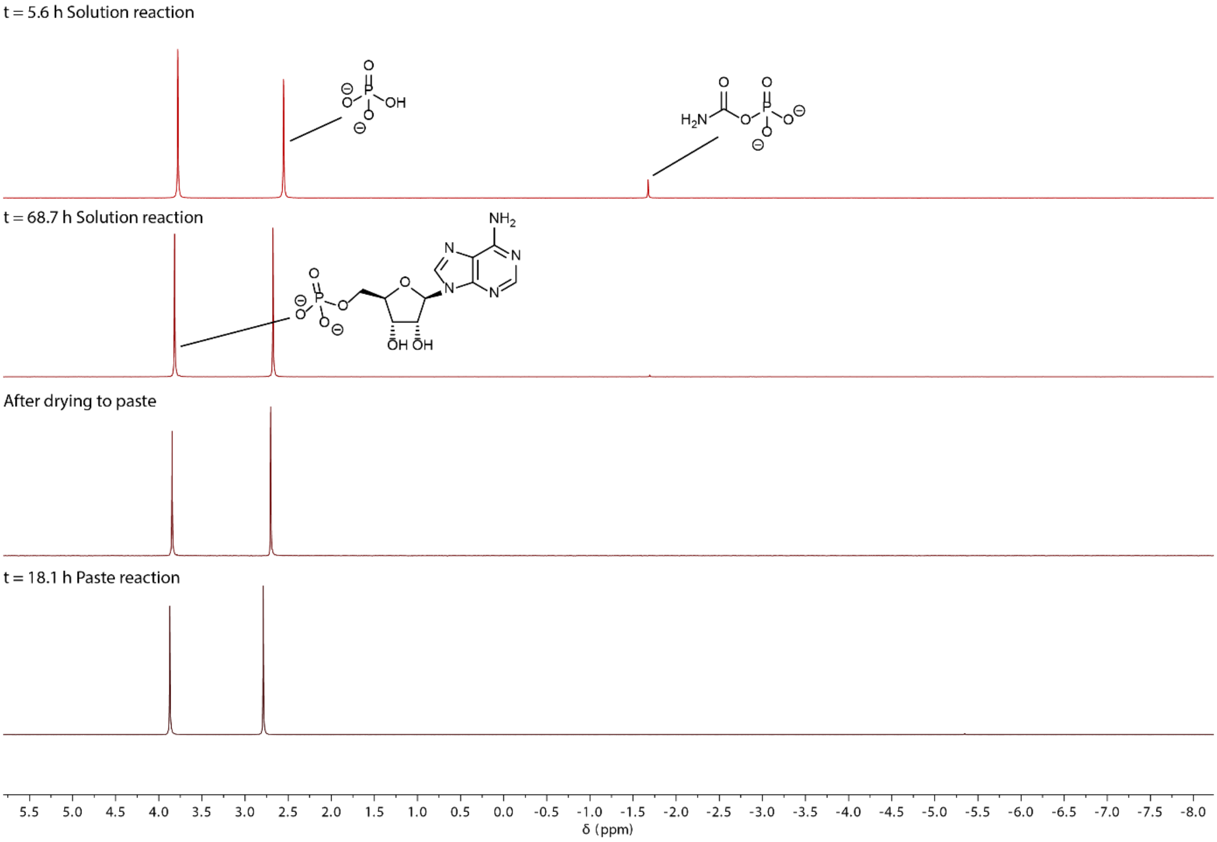

Supplementary Figure 279: Representative ^{31}P NMR spectra over time for the reaction of 188 mM (0.75 mmol) adenosine-5’-monophosphate and 450 mM (1.8 mmol) potassium cyanate and 150 mM (0.6 mmol) sodium phosphate dibasic. No imidazole was added to the experiment. The top two spectra were for the reaction in solution. The bottom two spectra were taken after evaporation to form a paste and after heating the paste at 50 °C for 18.1 h.

S7.11 Orthophosphate recycling with a wet/dry cycle with 100 mM reagent concentrations

S7.11.1 Supplementary experimental method for orthophosphate recycling with a wet/dry cycle with 100 mM reagent concentrations

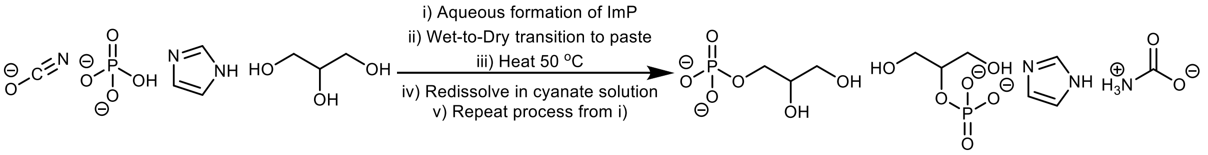

340 mg (5.0 mmol) of imidazole was dissolved in 50 mL of 9 : 1 H_2O : D_2O to give a 100 mM imidazole solution. 406 mg (5.0 mmol) of potassium cyanate and 89 mg (0.5 mmol) of sodium phosphate dibasic dihydrate salt were dissolved in 50.0 mL of 100 mM imidazole solution to give a 100 mM potassium cyanate and a 20 mM sodium phosphate dibasic solution. 37 mg (0.4 mmol) of glycerol was dissolved in 4.0 mL of 100 mM potassium cyanate, 20 mM sodium phosphate dibasic and 100 mM imidazole solution. The pH of the solution was measured and was typically in the region of pH 8.5-10.0. The pH of the solution was then adjusted to pH 7.3 using 5 M hydrochloric acid and 5 M sodium hydroxide solutions. The solution was left to react for 24 h in order to accumulate imidazole phosphate. The reaction was followed by ^{31}P-NMR and ^{1}H-NMR spectroscopy.

After 24 h, the solution was poured into a petri dish (10 cm diameter) as a single puddle and left with the lid off to dry at 22 °C for 24 h. The resulting paste was then scraped from the petri dish and placed into a 2.5 mL Eppendorf. The Eppendorf was then spun on a centrifuge for 3 min at 5590 g in order to move all solid to the bottom of the Eppendorf. The Eppendorf was then placed on a thermoshaker at 50 °C and 600 rpm for 24 h. After 24 h the reaction was removed from the thermoshaker and allowed to cool to room temperature. This completed the first cycle.

For the second cycle a 100 mM potassium cyanate solution was first prepared by dissolving 406 mg (5.0 mmol) of potassium cyanate in 50.0 mL of 9 : 1 H_2O : D_2O. The paste was then redissolved in 4.0 mL of the 100 mM potassium cyanate solution, adjusted to pH 7.3, and the same steps of leaving to react in solution for 24 h, drying to a paste for 24 h and heating to 50 °C for 24 h, as detailed above, were then repeated. The yield of phosphorylated compounds was determined by ^{31}P-NMR and ^{1}H-NMR spectroscopy. After the second cycle was complete a third cycle was then performed using an identical method.

Reactions with other nucleophiles were conducted using an identical procedure.

S7.11.2 Phosphorylation of glycerol with 20 mM orthophosphate recycling and 100 mM reagent concentrations

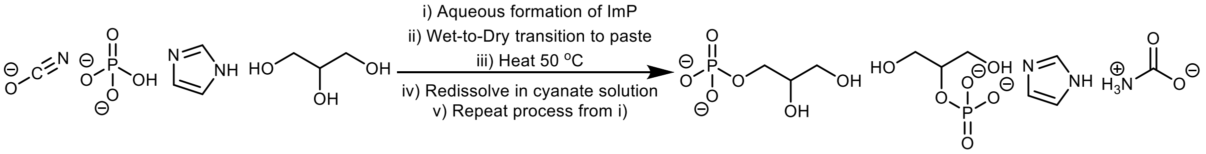

This reaction was performed according to the procedure in Section S7.11.1.

A total of three cycles were performed. Formation of imidazole phosphate was observed in solution and remained present after drying the solution to a paste. Formation of glycerol-1-phosphate and glycerol-2-phosphate occurred during both the drying to the paste over a period of ~24 h and during the heating of the paste to 50 °C for ~24 h (Supplementary Figure 280, 281 and 282).

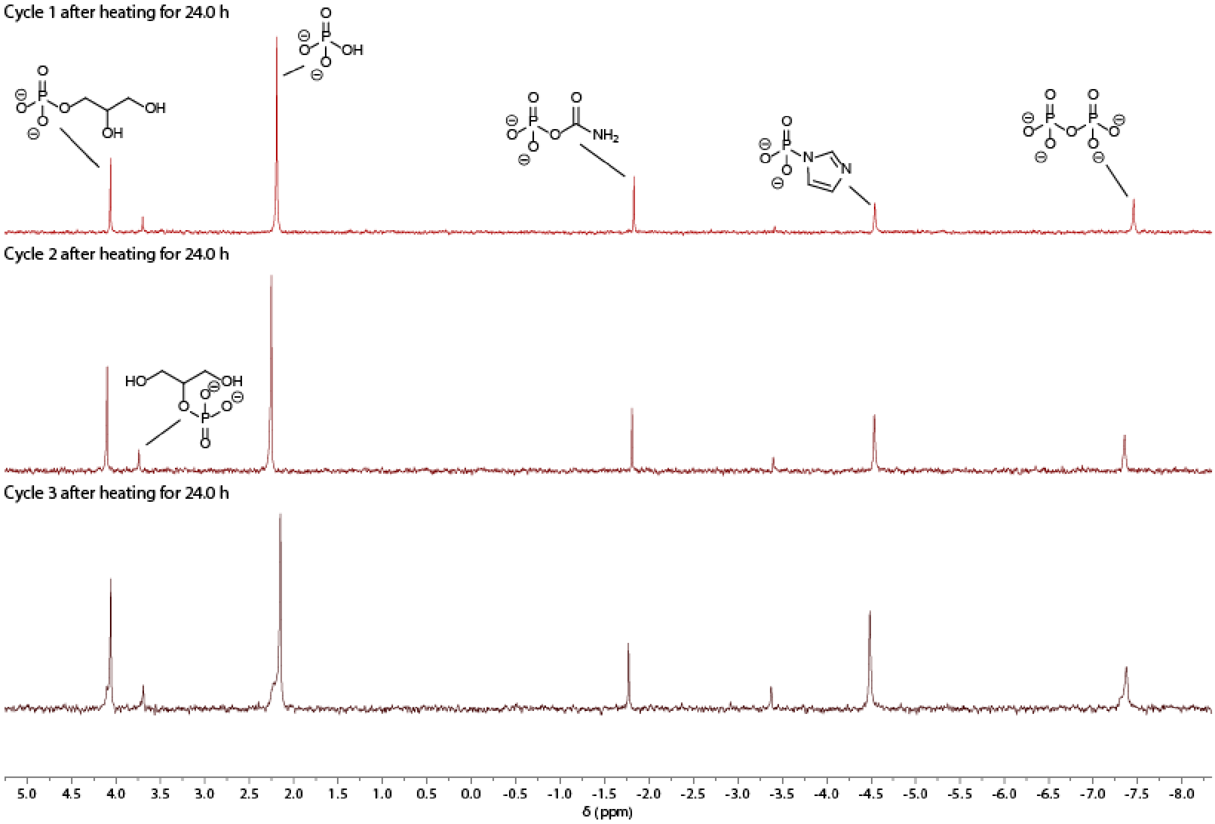

Supplementary Figure 280: Representative ^{31}P NMR spectra for the three cycles with 100 mM glycerol, 100 mM potassium cyanate and 20 mM sodium phosphate in 100 mM imidazole buffer. For each cycle the ^{31}P NMR spectrum after the reaction had been dried to a paste and the ^{31}P NMR spectrum after heating the reaction to 50 °C for ~24 h are shown. All NMR spectra were measured at pH 7.3 in 9 : 1 H_2O : D_2O.

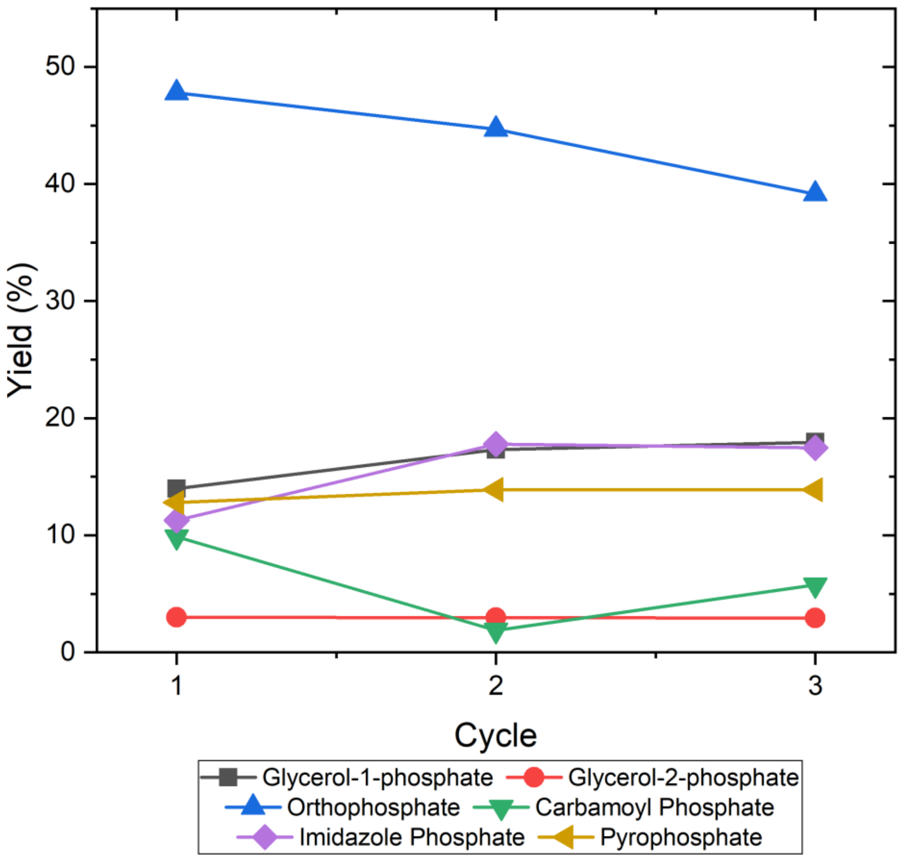

Supplementary Figure 281: Changes in yield for all phosphorous species over the course of three cycles for the reaction with 100 mM glycerol, 100 mM potassium cyanate and 20 mM sodium phosphate in 100 mM imidazole buffer.

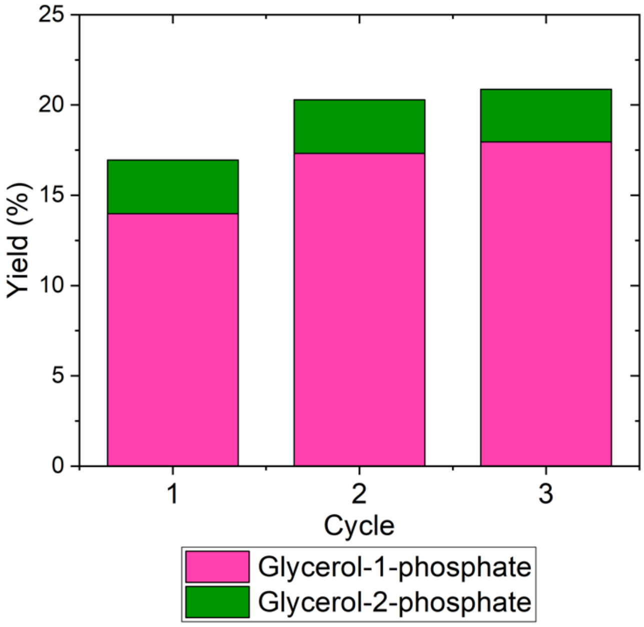

Supplementary Figure 282: Yield of glycerol-1-phosphate and glycerol-2-phosphate after each cycle for the reaction with 100 mM glycerol, 100 mM potassium cyanate and 20 mM sodium phosphate in 100 mM imidazole buffer.

S7.11.3 Phosphorylation of glycerol with 50 mM orthophosphate and 100 mM reagent concentrations

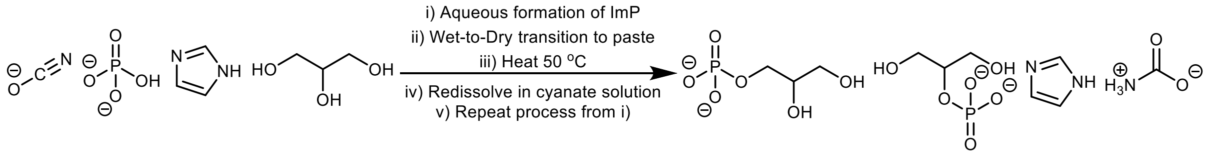

This reaction was performed according to the procedure in Section S7.11.1 but with 50 mM sodium phosphate dibasic dihydrate salt (222.5 mg, 1.25 mmol).

A total of three cycles were performed. Formation of imidazole phosphate was observed in solution and remained present after drying the solution to a paste. Formation of glycerol-1-phosphate and glycerol-2-phosphate occurred during both the drying to the paste over a period of ~24 h and during the heating of the paste to 50 °C for ~24 h (Supplementary Figure 283, 284 and 285).

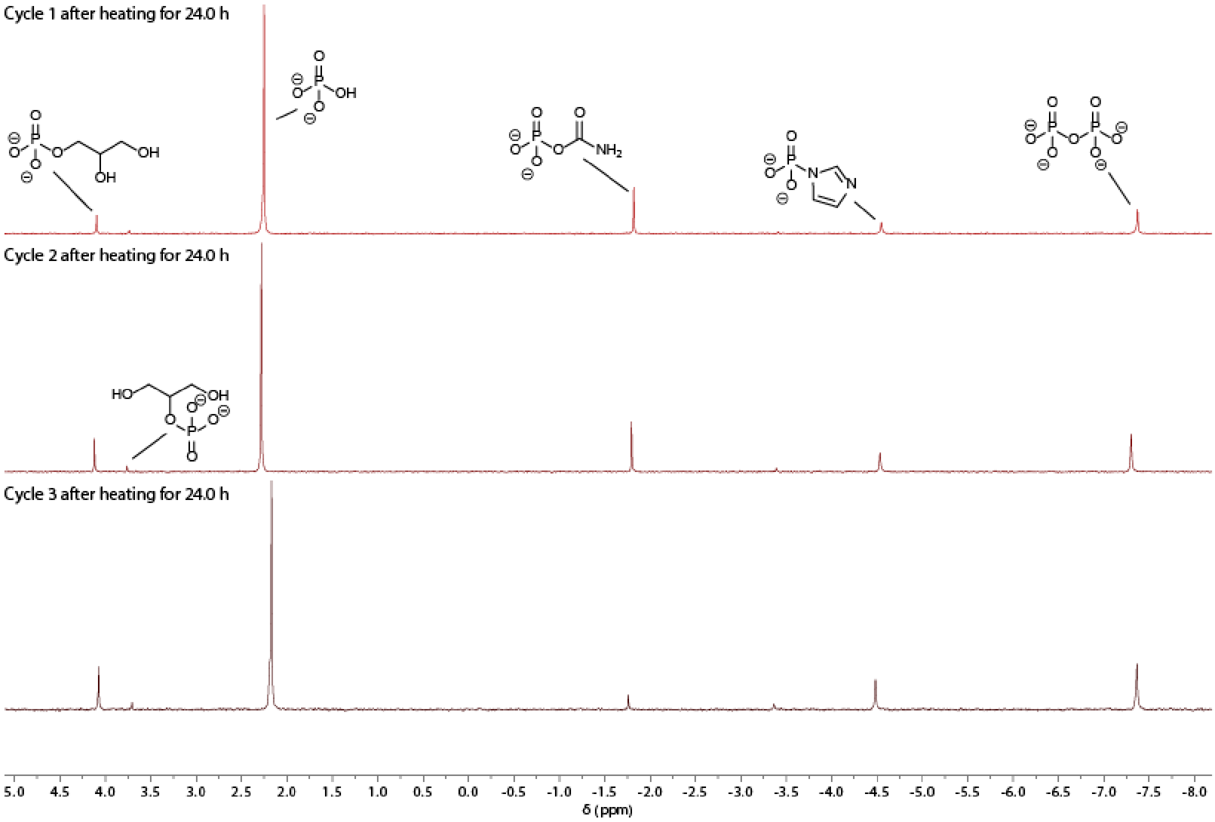

Supplementary Figure 283: Representative ^{31}P NMR spectra for the three cycles with 100 mM glycerol, 100 mM potassium cyanate and 50 mM sodium phosphate in 100 mM imidazole buffer. For each cycle the ^{31}P NMR spectrum after the reaction had been dried to a paste and the ^{31}P NMR spectrum after heating the reaction to 50 °C for ~24 h are shown. All NMR spectra were measured at pH 7.3 in 9 : 1 H_2O : D_2O.

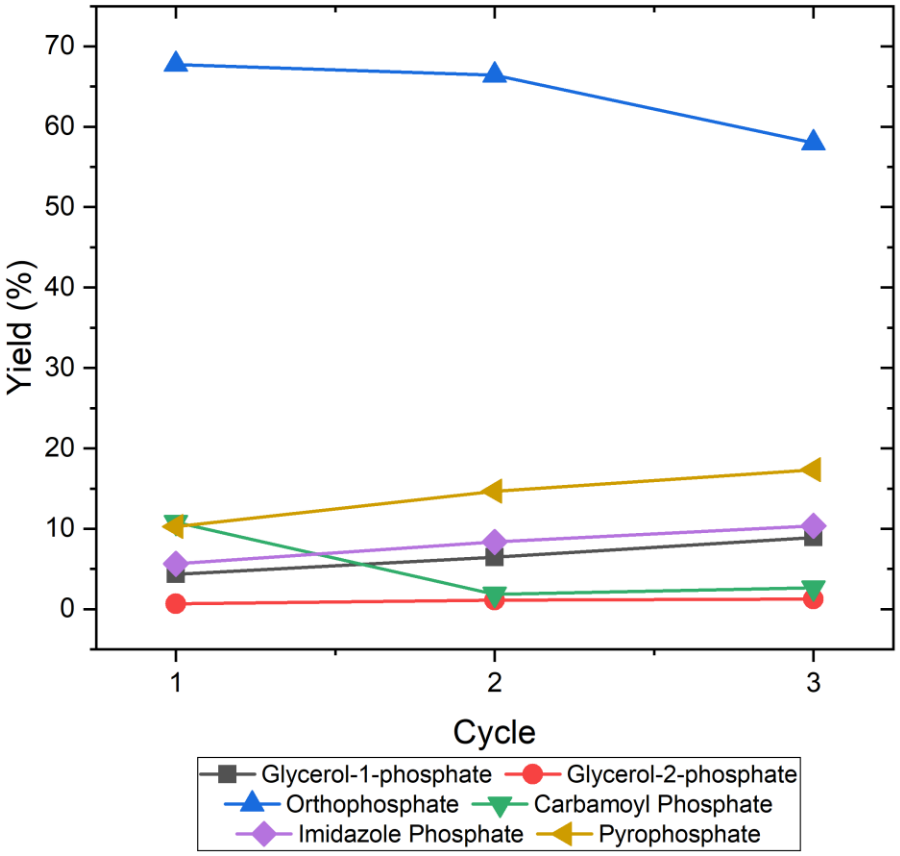

Supplementary Figure 284: Changes in yield for all phosphorous species over the course of three cycles for the reaction with 100 mM glycerol, 100 mM potassium cyanate and 50 mM sodium phosphate in 100 mM imidazole buffer.

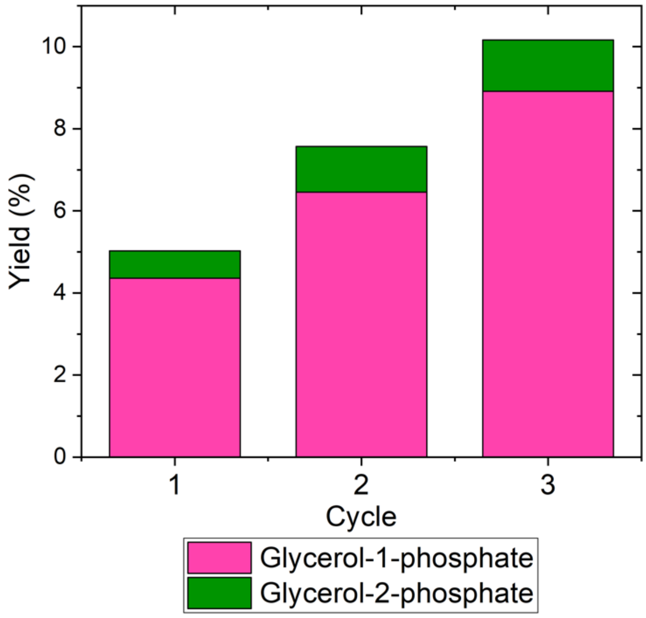

Supplementary Figure 285: Yield of glycerol-1-phosphate and glycerol-2-phosphate after each cycle for the reaction with 100 mM glycerol, 100 mM potassium cyanate and 50 mM sodium phosphate in 100 mM imidazole buffer.

S7.11.4 Phosphorylation of glycerate with 20 mM orthophosphate and 100 mM reagent concentrations

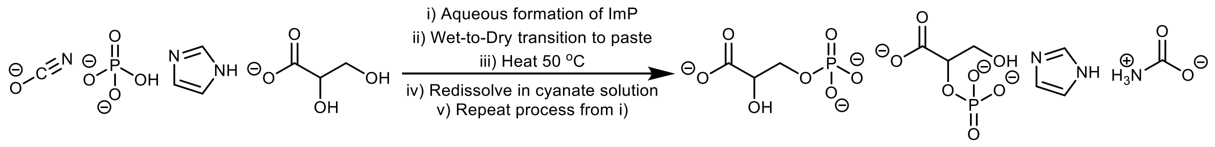

This reaction was performed according to the procedure in Section S7.11.1 but with 100 mM of sodium glycerate (42.4 mg, 0.4 mmol).

A total of three cycles were performed. Formation of imidazole phosphate was observed in solution and remained present after drying the solution to a paste. Formation of glycerate-2-phosphate and glycerate-3-phosphate occurred during both the drying to the paste over a period of ~24 h and during the heating of the paste to 50 °C for ~24 h (Supplementary Figure 286, 287 and 288).

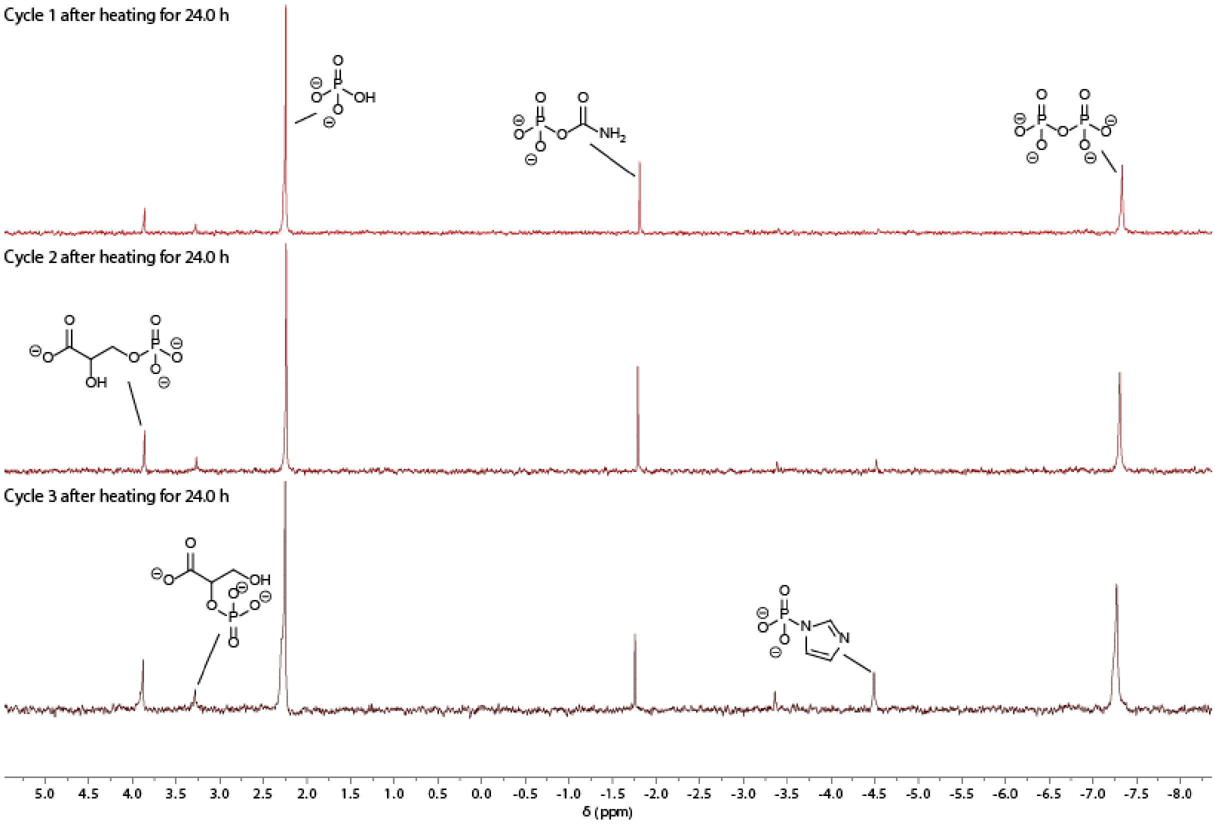

Supplementary Figure 286: Representative ^{31}P NMR spectra for the three cycles with 100 mM sodium glycerate, 100 mM potassium cyanate and 20 mM sodium phosphate in 100 mM imidazole buffer. For each cycle the ^{31}P NMR spectrum after the reaction had been dried to a paste and the ^{31}P NMR spectrum after heating the reaction to 50 °C for ~24 h are shown. All NMR spectra were measured at pH 7.3 in 9 : 1 H_2O : D_2O.

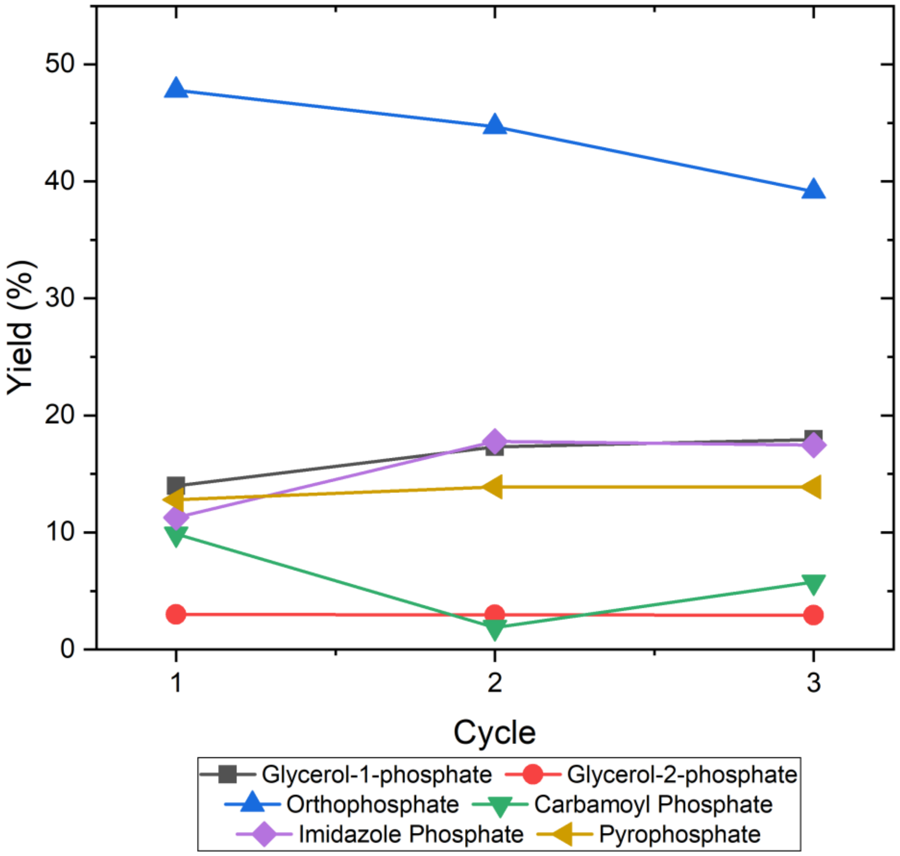

Supplementary Figure 287: Changes in yield for all phosphorous species over the course of three cycles for the reaction with 100 mM sodium glycerate, 100 mM potassium cyanate and 20 mM sodium phosphate in 100 mM imidazole buffer.

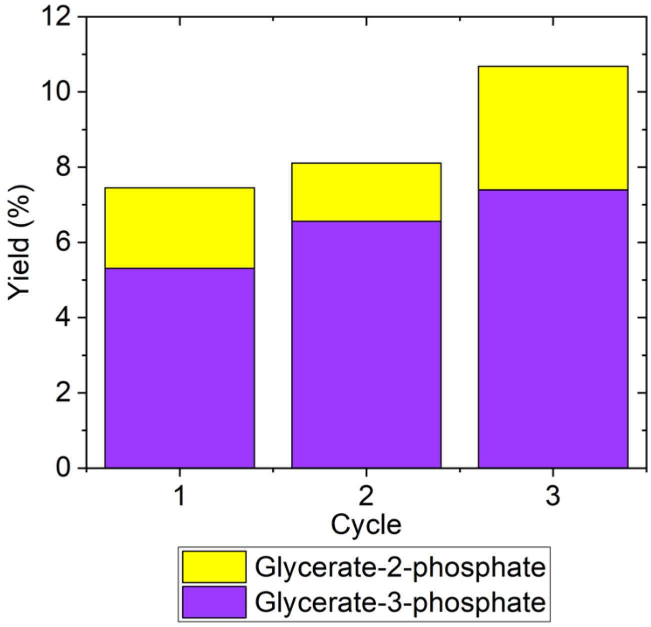

Supplementary Figure 288: Yield of glycerate-2-phosphate and glycerate-3-phosphate after each cycle for the reaction with 100 mM sodium glycerate, 100 mM potassium cyanate and 20 mM sodium phosphate in 100 mM imidazole buffer.

S7.11.5 Phosphorylation of glycerate with 50 mM orthophosphate and 100 mM reagent concentrations

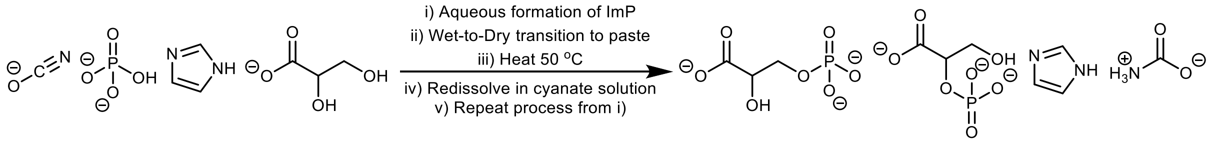

This reaction was performed according to the procedure in Section S7.11.1 but with 100 mM of sodium glycerate (42.4 mg, 0.4 mmol) and 50 mM sodium phosphate dibasic dihydrate salt (222.5 mg, 1.25 mmol).

A total of three cycles were performed. Formation of imidazole phosphate was observed in solution and remained present after drying the solution to a paste. Formation of glycerate-2-phosphate and glycerate-3-phosphate occurred during both the drying to the paste over a period of ~24 h and during the heating of the paste to 50 °C for ~24 h (Supplementary Figure 289, 290 and 291).

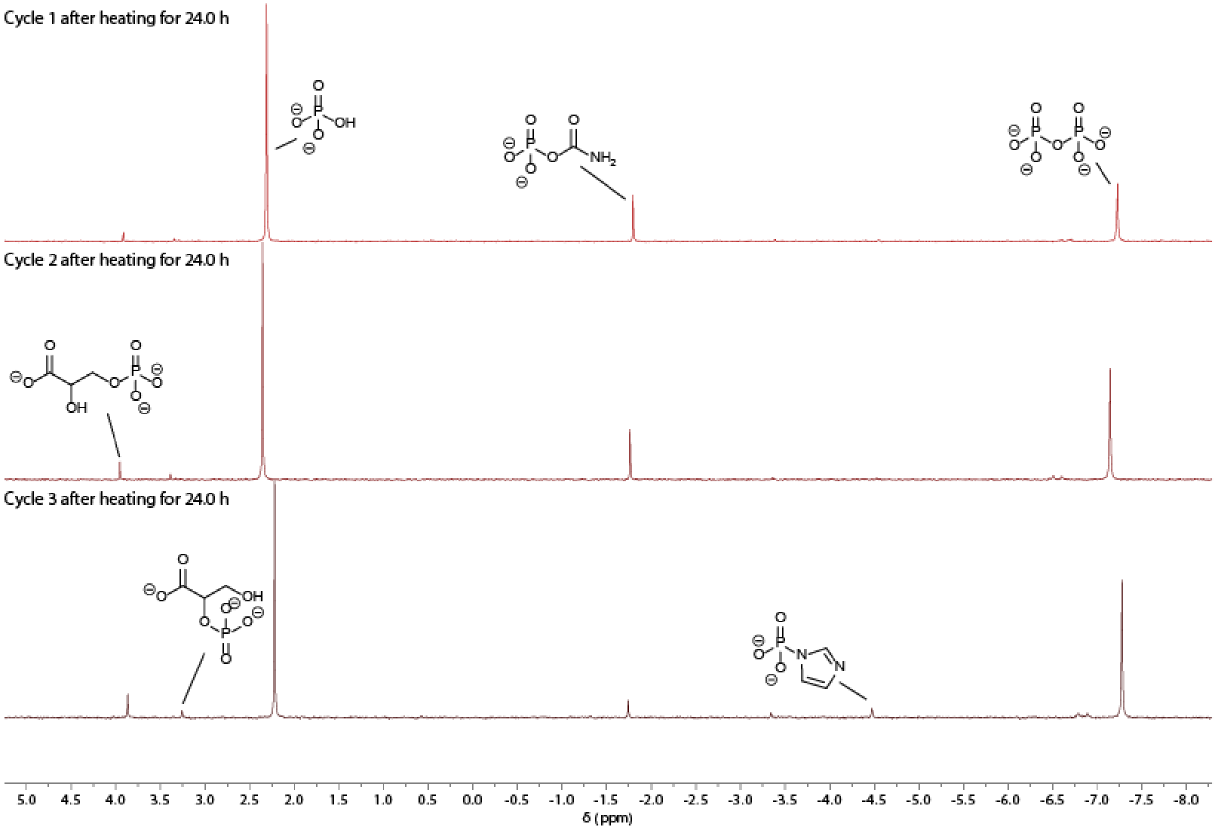

Supplementary Figure 289: Representative ^{31}P NMR spectra for the three cycles with 100 mM sodium glycerate, 100 mM potassium cyanate and 50 mM sodium phosphate in 100 mM imidazole buffer. For each cycle the ^{31}P NMR spectrum after the reaction had been dried to a paste and the ^{31}P NMR spectrum after heating the reaction to 50 °C for ~24 h are shown. All NMR spectra were measured at pH 7.3 in 9 : 1 H_2O : D_2O.

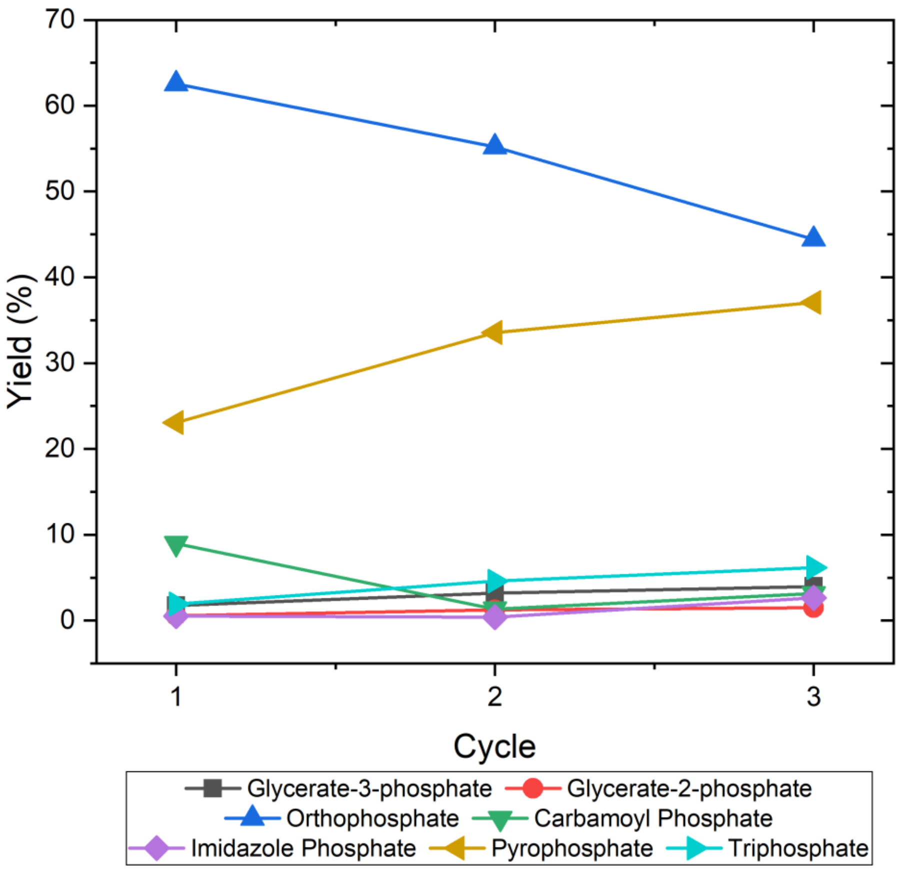

Supplementary Figure 290: Changes in yield for all phosphorous species over the course of three cycles for the reaction with 100 mM sodium glycerate, 100 mM potassium cyanate and 50 mM sodium phosphate in 100 mM imidazole buffer.

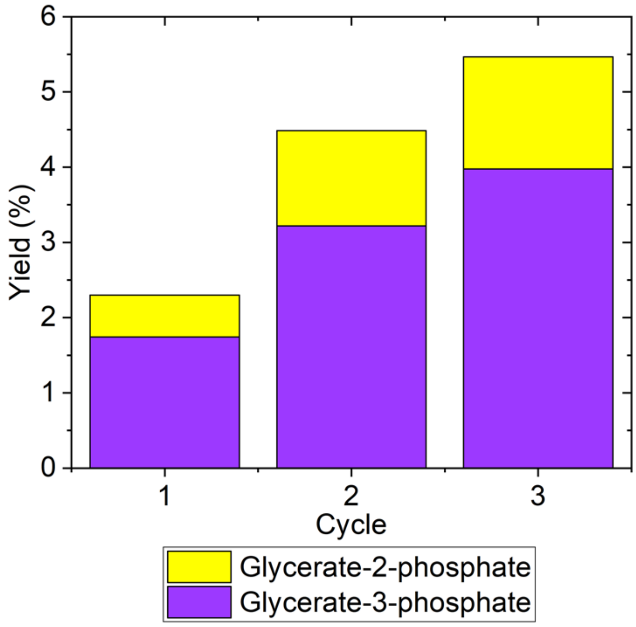

Supplementary Figure 291: Yield of glycerate-2-phosphate and glycerate-3-phosphate after each cycle for the reaction with 100 mM sodium glycerate, 100 mM potassium cyanate and 50 mM sodium phosphate in 100 mM imidazole buffer.

S7.11.6 *Phosphorylation of adenosine-5'-monophosphate with 20 mM orthophosphate and 100 mM reagent concentrations*

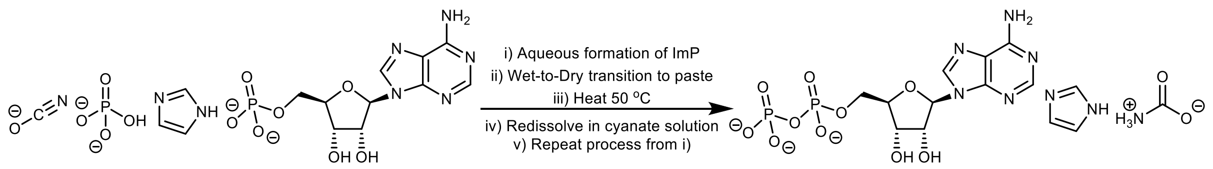

This reaction was performed according to the procedure in Section S7.11.1 but with 100 mM of adenosine-5'-monophosphate disodium salt (188.5 mg, 0.4 mmol).

A total of three cycles were performed. Formation of imidazole phosphate was observed in solution and remained present after drying the solution to a paste. Formation of adenosine-5'-diphosphate occurred during both the drying to the paste over a period of ~24 h and during the heating of the paste to 50 °C for ~24 h (Supplementary Figure 292, 293 and 294).

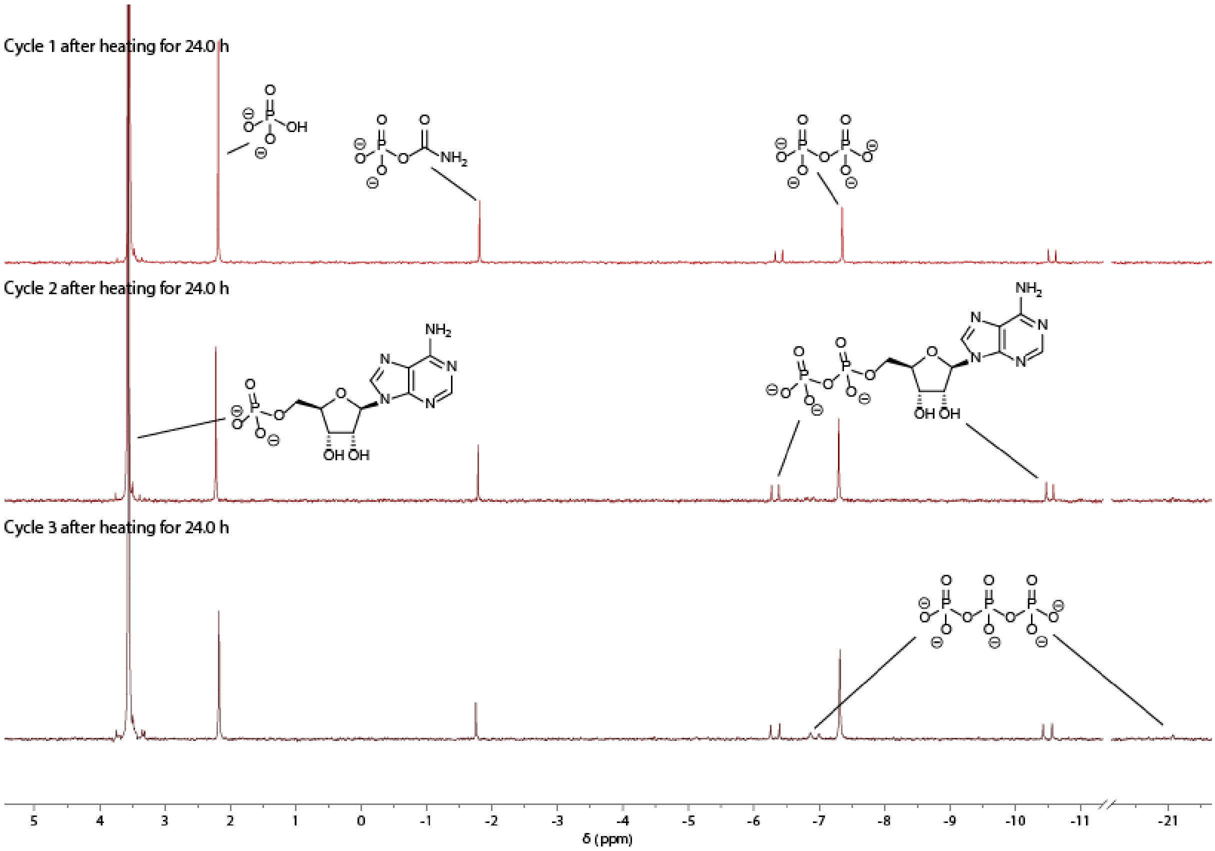

Supplementary Figure 292: Representative ^{31}P NMR spectra for the three cycles with 100 mM adenosine-5'-monophosphate, 100 mM potassium cyanate and 20 mM sodium phosphate in 100 mM imidazole buffer. For each cycle the ^{31}P NMR spectrum after the reaction had been dried to a paste and the ^{31}P NMR spectrum after heating the reaction to 50 °C for ~24 h are shown. All NMR spectra were measured at pH 7.3 in 9 : 1 H_2O : D_2O.

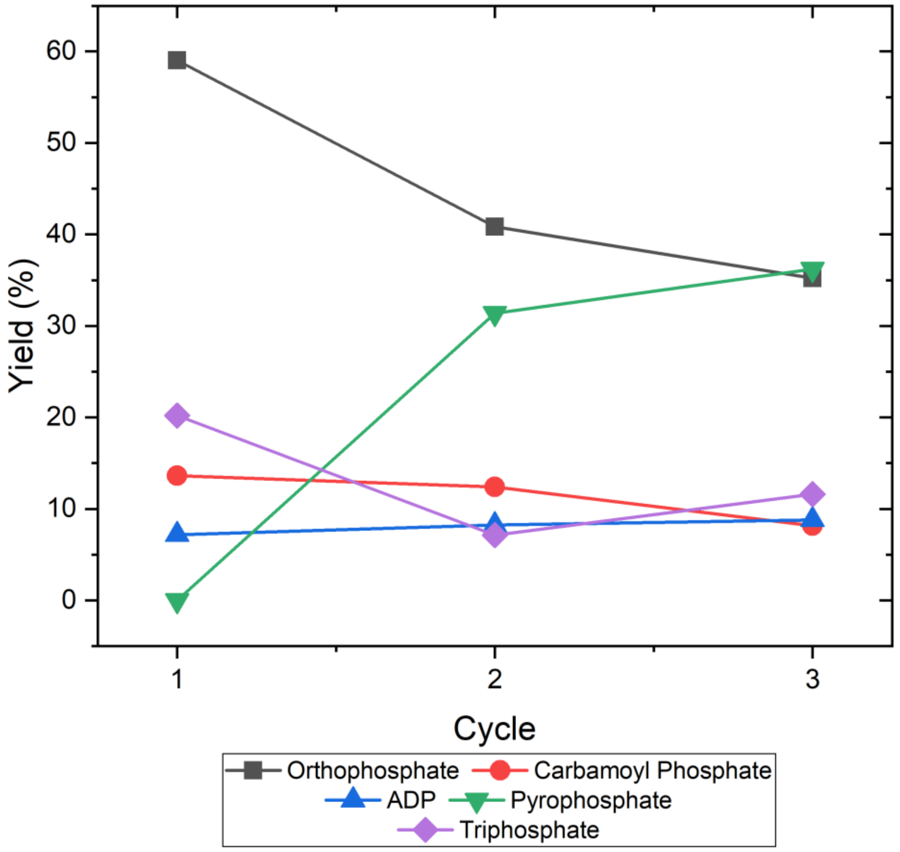

Supplementary Figure 293: Changes in yield for all phosphorous species over the course of three cycles for the reaction with 100 mM adenosine-5'-monophosphate, 100 mM potassium cyanate and 20 mM sodium phosphate in 100 mM imidazole buffer.

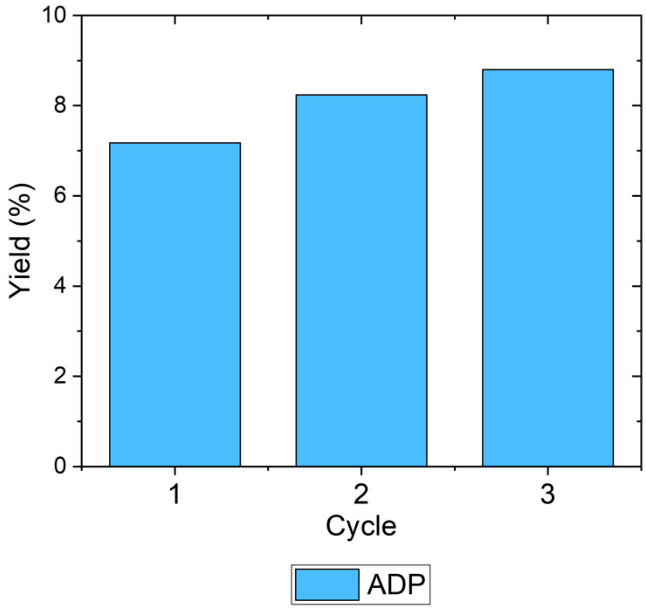

Supplementary Figure 294: Yield of adenosine-5'-diphosphate after each cycle for the reaction with 100 mM adenosine-5'-monophosphate, 100 mM potassium cyanate and 20 mM sodium phosphate in 100 mM imidazole buffer.

S7.11.7 Phosphorylation of adenosine-5'-monophosphate with 50 mM orthophosphate and 100 mM reagent concentrations

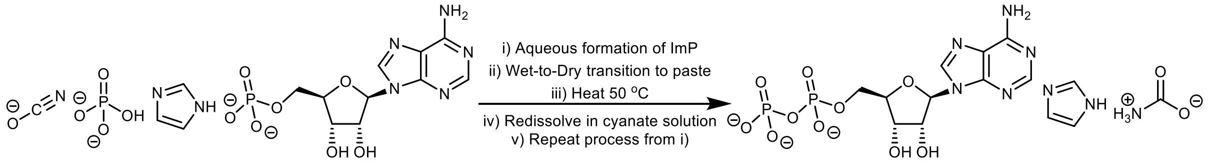

This reaction was performed according to the procedure in Section S7.11.1 but with 100 mM of adenosine-5'-monophosphate disodium salt (188.5 mg, 0.4 mmol) and 50 mM sodium phosphate dibasic dihydrate salt (222.5 mg, 1.25 mmol).

A total of three cycles were performed. Formation of imidazole phosphate was observed in solution and remained present after drying the solution to a paste. Formation of adenosine-5'-diphosphate occurred during both the drying to the paste over a period of ~24 h and during the heating of the paste to 50 °C for ~24 h (Supplementary Figure 295, 296 and 297).

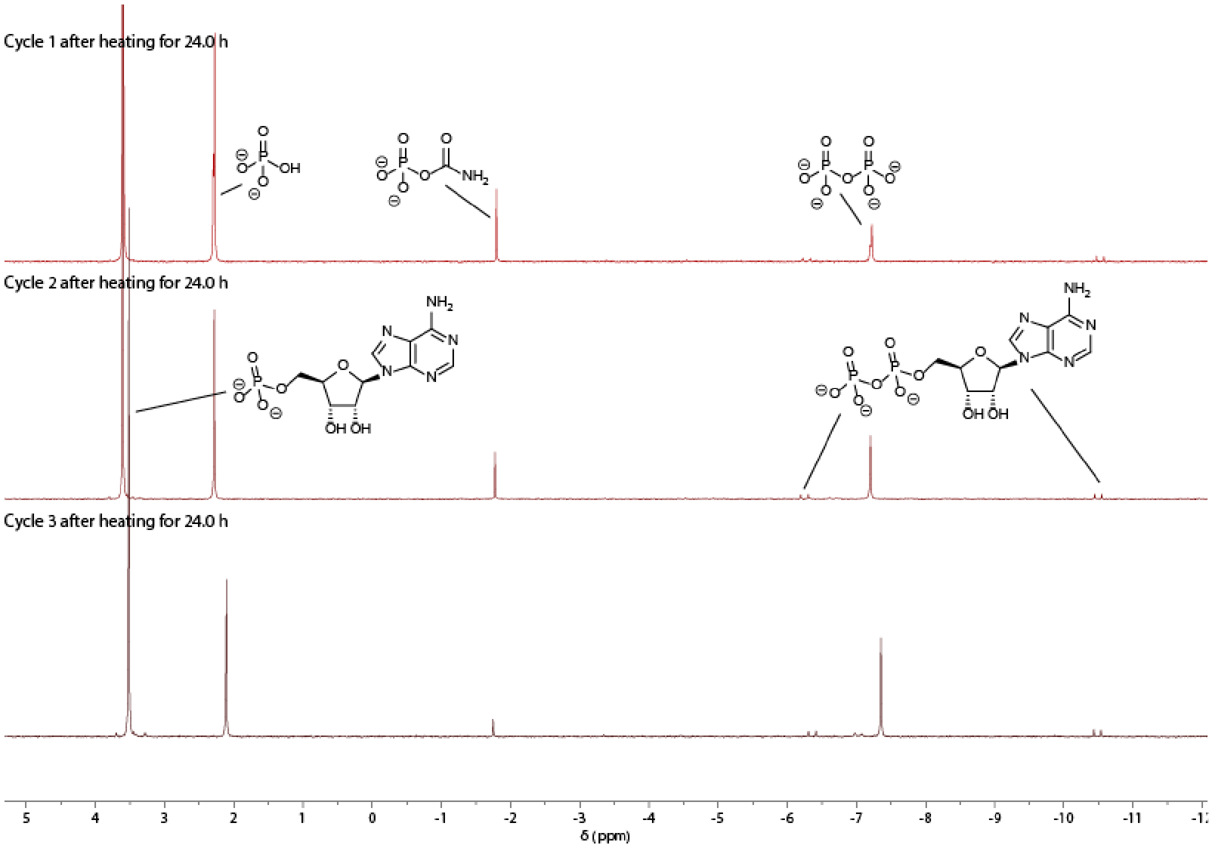

Supplementary Figure 295: Representative ^{31}P NMR spectra for the three cycles with 100 mM adenosine-5'-monophosphate, 100 mM potassium cyanate and 50 mM sodium phosphate in 100 mM imidazole buffer. For each cycle the ^{31}P NMR spectrum after the reaction had been dried to a paste and the ^{31}P NMR spectrum after heating the reaction to 50 °C for ~24 h are shown. All NMR spectra were measured at pH 7.3 in 9 : 1 H_2O : D_2O.

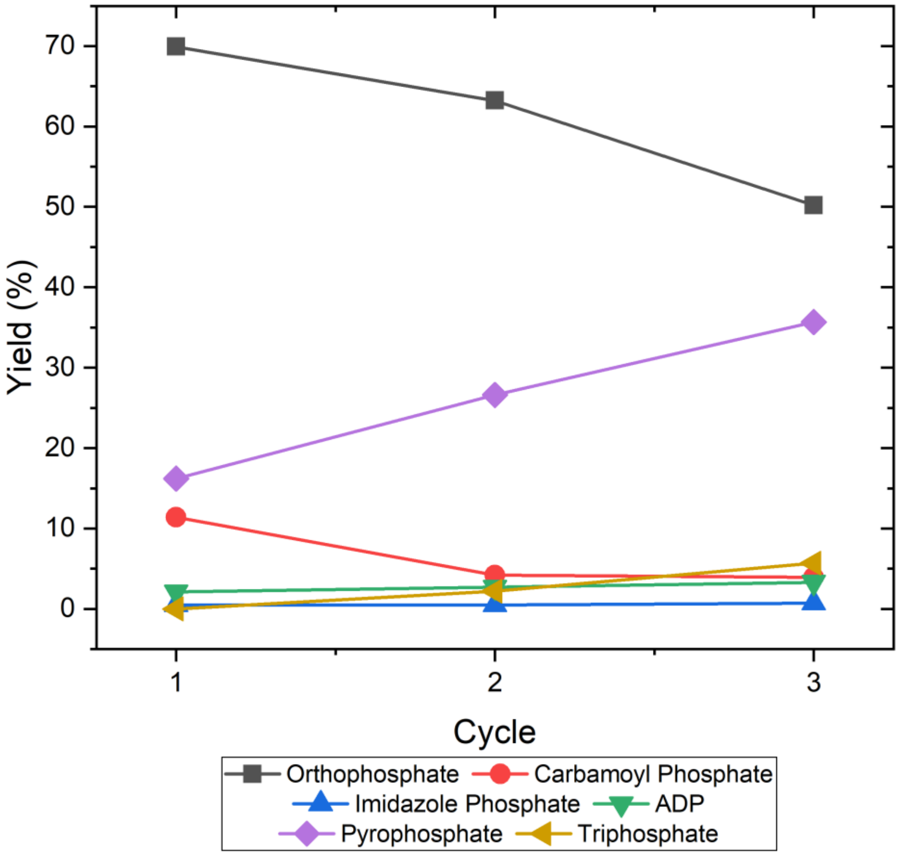

Supplementary Figure 296: Changes in yield for all phosphorous species over the course of three cycles for the reaction with 100 mM adenosine-5'-monophosphate, 100 mM potassium cyanate and 50 mM sodium phosphate in 100 mM imidazole buffer.

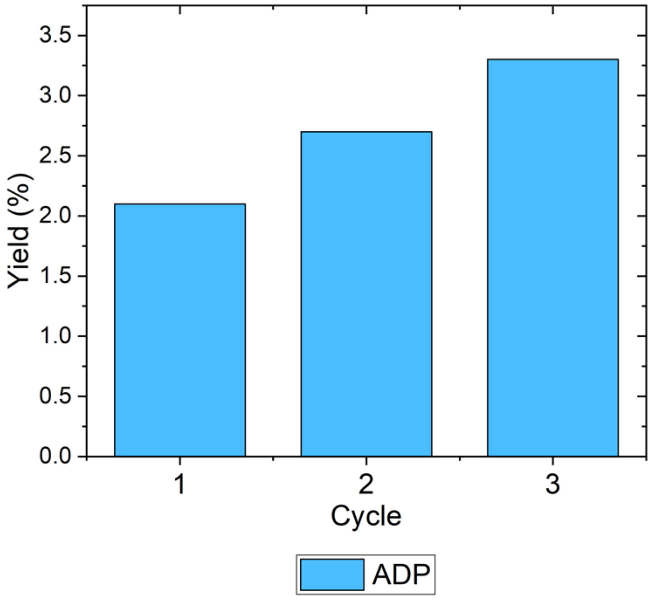

Supplementary Figure 297: Yield of adenosine-5'-diphosphate after each cycle for the reaction with 100 mM adenosine-5'-monophosphate, 100 mM potassium cyanate and 50 mM sodium phosphate in 100 mM imidazole buffer.

S7.11.8 Phosphorylation of serine with 20 mM orthophosphate and 100 mM reagent concentrations

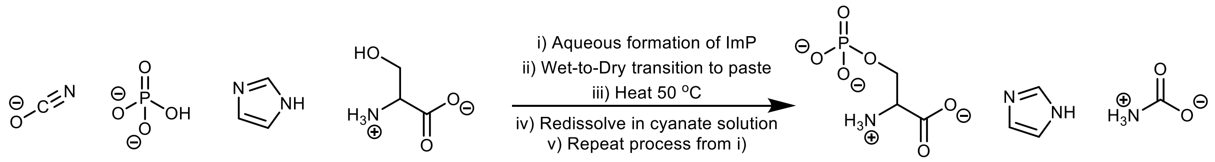

This reaction was performed according to the procedure in Section S7.11.1 but with 100 mM of serine (36.8 mg, 0.4 mmol).

A total of three cycles were performed. Formation of imidazole phosphate was observed in solution and remained present after drying the solution to a paste. Formation of O-phosphorylated serine occurred during both the drying to the paste over a period of ~24 h and during the heating of the paste to 50 °C for ~24 h (Supplementary Figure 298, 299 and 300).

In the ^{1}H NMR spectra, multiple peaks consistent with the formation of N-carbamoyl serine and Ser-Ser dipeptide formation were observed.[3]

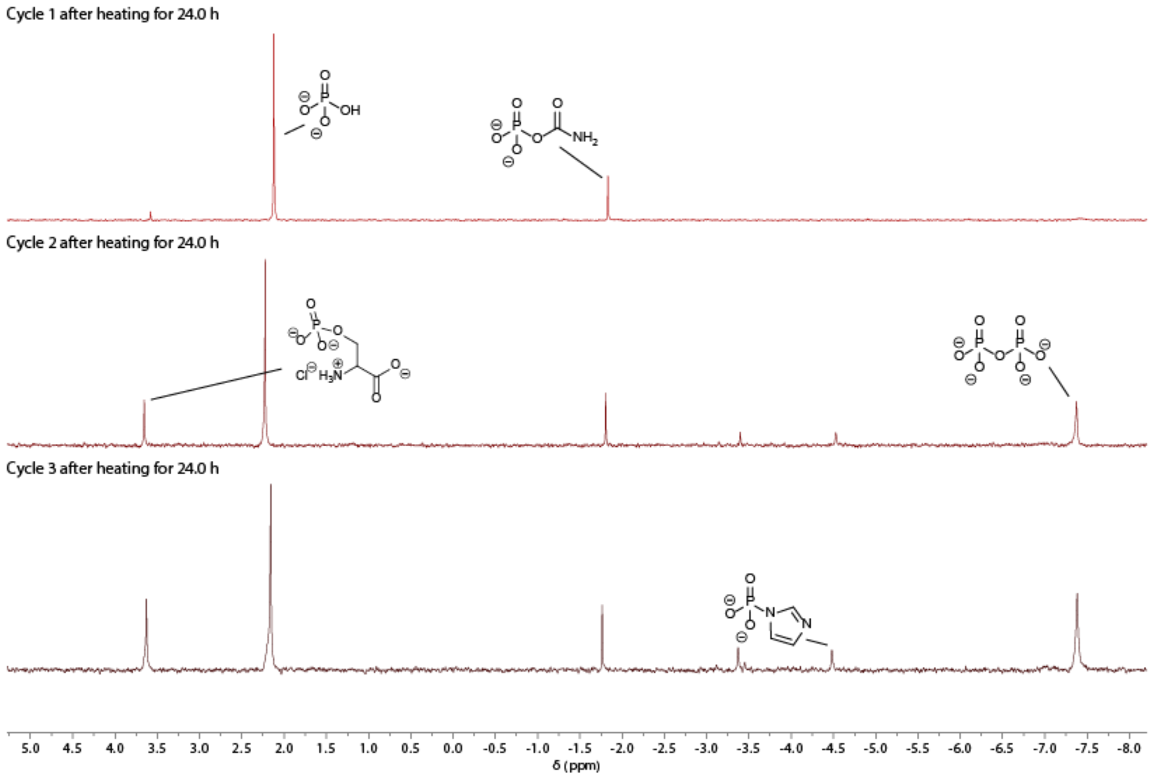

Supplementary Figure 298: Representative ^{31}P NMR spectra for the three cycles with 100 mM serine, 100 mM potassium cyanate and 20 mM sodium phosphate in 100 mM imidazole buffer. For each cycle the ^{31}P NMR spectrum after the reaction had been dried to a paste and the ^{31}P NMR spectrum after heating the reaction to 50 °C for ~24 h are shown. All NMR spectra were measured at pH 7.3 in 9 : 1 H_2O : D_2O.

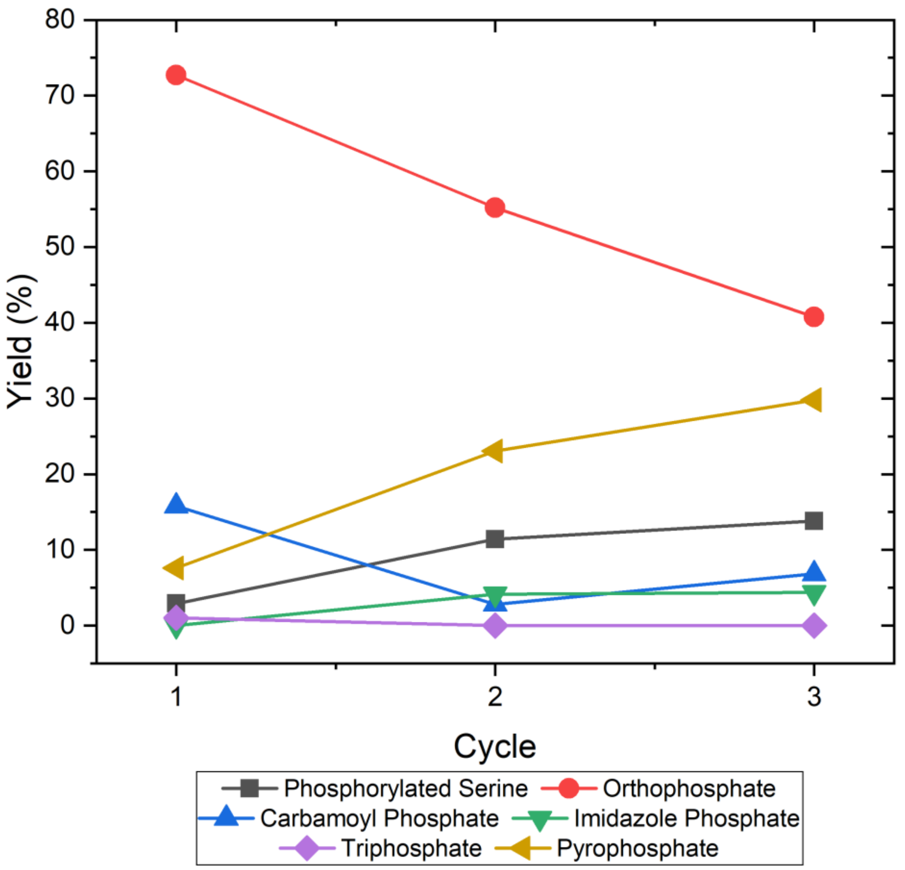

Supplementary Figure 299: Changes in yield for all phosphorous species over the course of three cycles for the reaction with 100 mM serine, 100 mM potassium cyanate and 20 mM sodium phosphate in 100 mM imidazole buffer.

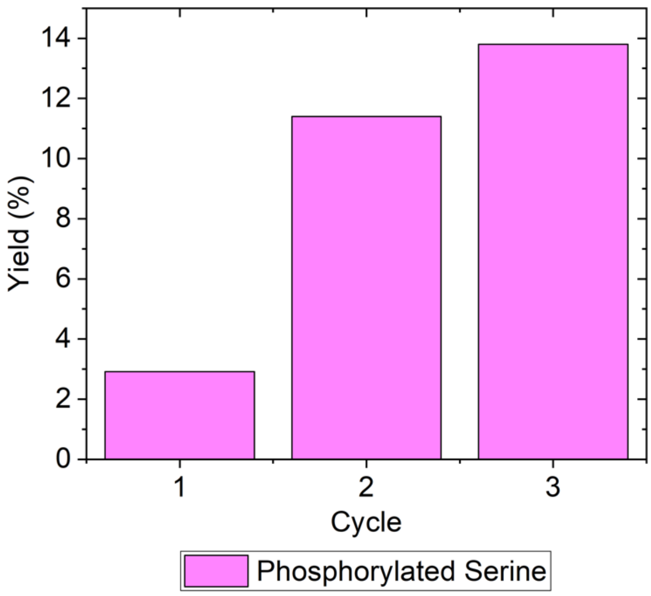

Supplementary Figure 300: Yield of O-phosphorylated serine after each cycle for the reaction with 100 mM serine, 100 mM potassium cyanate and 20 mM sodium phosphate in 100 mM imidazole buffer.

S7.11.9 Phosphorylation of serine with 50 mM orthophosphate and 100 mM reagent concentrations

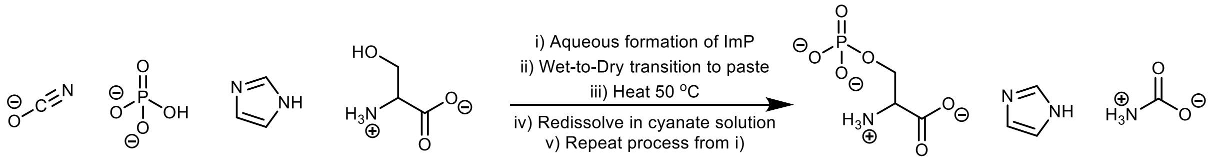

This reaction was performed according to the procedure in Section S7.11.1 but with 100 mM of serine (36.8 mg, 0.4 mmol) and 50 mM sodium phosphate dibasic dihydrate salt (222.5 mg, 1.25 mmol).

A total of three cycles were performed. Formation of imidazole phosphate was observed in solution and remained present after drying the solution to a paste. Formation of O-phosphorylated serine occurred during both the drying to the paste over a period of ~24 h and during the heating of the paste to 50 °C for ~24 h (Supplementary Figure 301, 302 and 303).

In the ^{1}H NMR spectra, multiple peaks consistent with the formation of N-carbamoyl serine and Ser-Ser dipeptide formation were observed.[3]

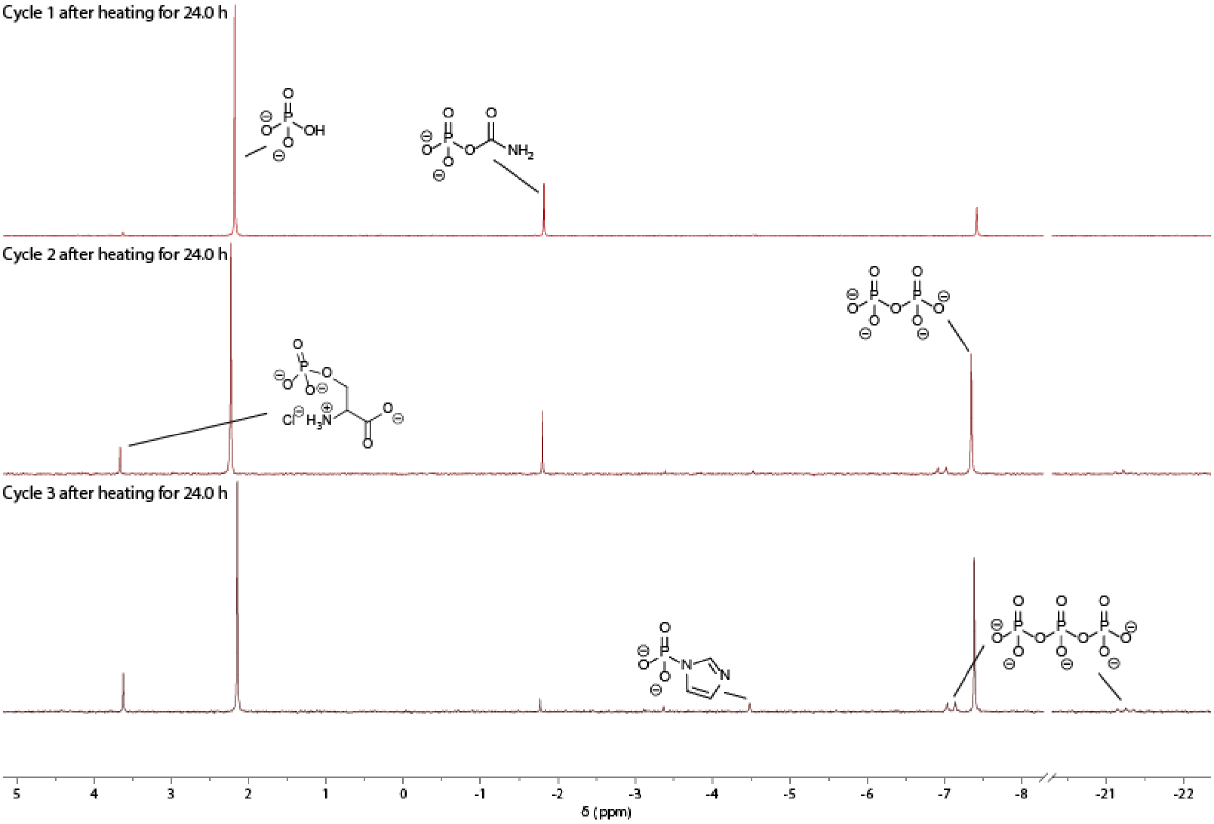

Supplementary Figure 301: Representative ^{31}P NMR spectra for the three cycles with 100 mM serine, 100 mM potassium cyanate and 50 mM sodium phosphate in 100 mM imidazole buffer. For each cycle the ^{31}P NMR spectrum after the reaction had been dried to a paste and the ^{31}P NMR spectrum after heating the reaction to 50 °C for ~24 h are shown. All NMR spectra were measured at pH 7.3 in 9 : 1 H_2O : D_2O.

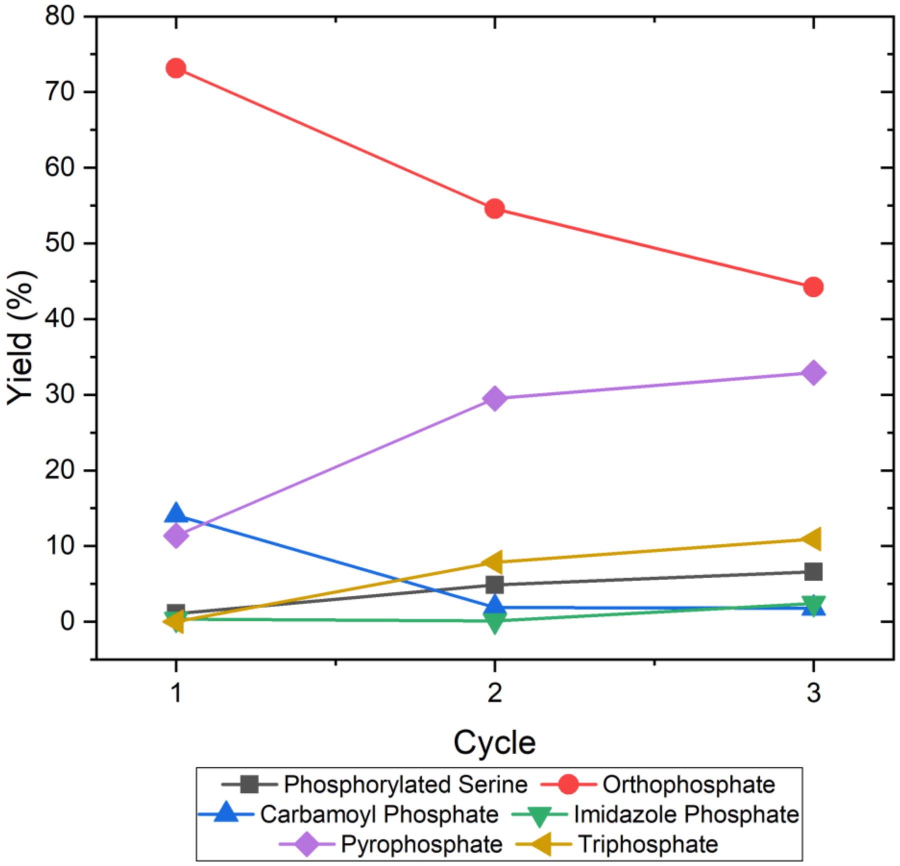

Supplementary Figure 302: Changes in yield for all phosphorous species over the course of three cycles for the reaction with 100 mM serine, 100 mM potassium cyanate and 50 mM sodium phosphate in 100 mM imidazole buffer.

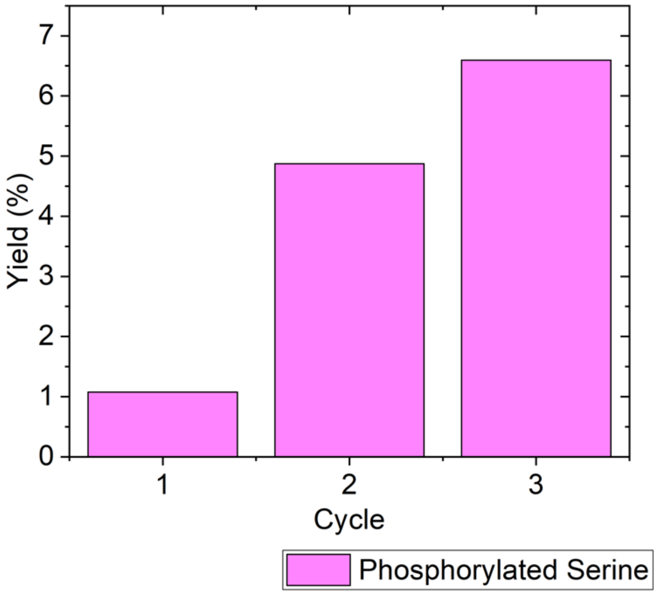

Supplementary Figure 303: Yield of O-phosphorylated serine after each cycle for the reaction with 100 mM serine, 100 mM potassium cyanate and 50 mM sodium phosphate in 100 mM imidazole buffer.

S7.11.10 Phosphorylation of cytidine with 20 mM orthophosphate and 100 mM reagent concentrations

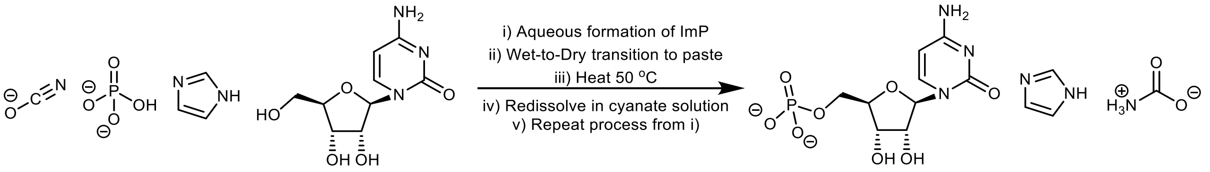

This reaction was performed according to the procedure in Section S7.11.1 but with 100 mM of cytidine (97.3 mg, 0.4 mmol).

A total of three cycles were performed. Formation of imidazole phosphate was observed in solution and remained present after drying the solution to a paste. Formation of cytidine-5'-monophosphate, cytidine-2'-monophosphate and cytidine-3'-monophosphate occurred during both the drying to the paste over a period of ~24 h and during the heating of the paste to 50 °C for ~24 h (Supplementary Figure 304, 305 and 306).

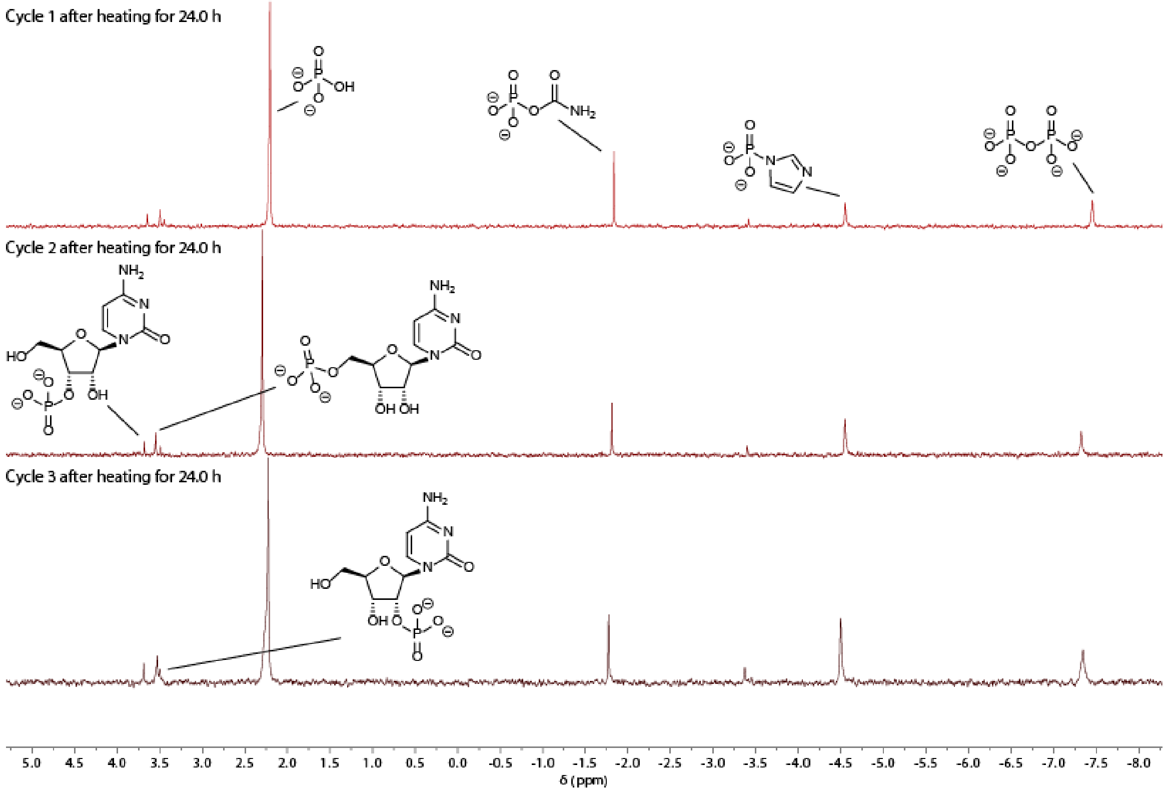

Supplementary Figure 304: Representative ^{31}P NMR spectra for the three cycles with 100 mM of cytidine, 100 mM potassium cyanate and 20 mM sodium phosphate in 100 mM imidazole buffer. For each cycle the ^{31}P NMR spectrum after the reaction had been dried to a paste and the ^{31}P NMR spectrum after heating the reaction to 50 °C for ~24 h are shown. All NMR spectra were measured at pH 7.3 in 9 : 1 H_2O : D_2O.

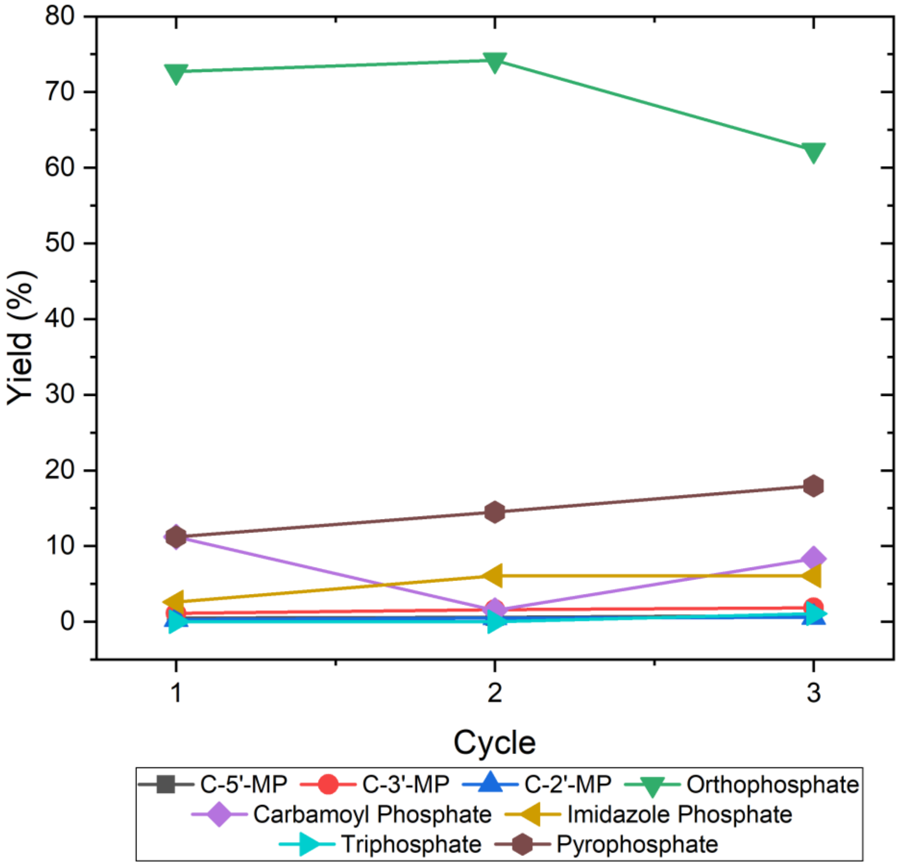

Supplementary Figure 305: Changes in yield for all phosphorous species over the course of three cycles for the reaction with 100 mM of cytidine, 100 mM potassium cyanate and 20 mM sodium phosphate in 100 mM imidazole buffer.

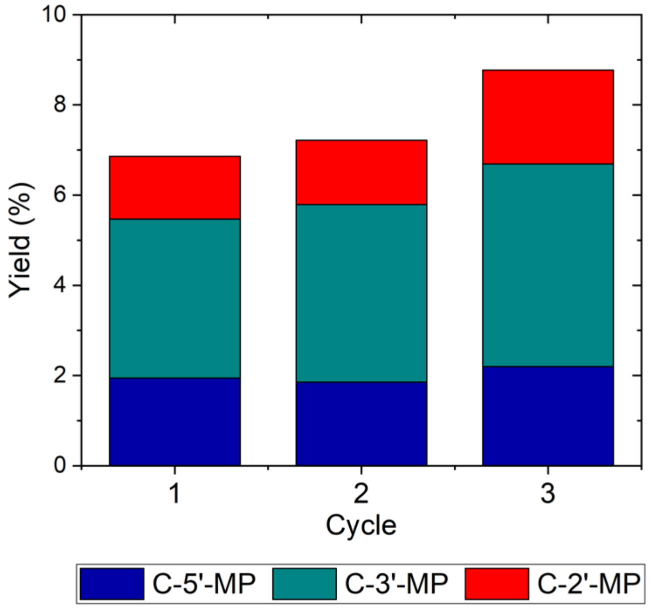

Supplementary Figure 306: Yield of cytidine-5'-monophosphate, cytidine-2'-monophosphate and cytidine-3'-monophosphate after each cycle for the reaction with 100 mM of cytidine, 100 mM potassium cyanate and 20 mM sodium phosphate in 100 mM imidazole buffer.

S7.11.11 Phosphorylation of cytidine with 50 mM orthophosphate and 100 mM reagent concentrations

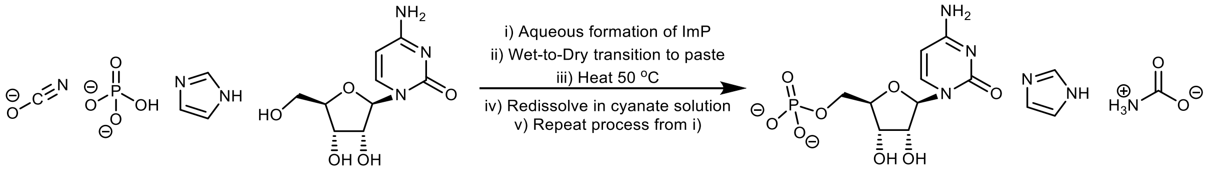

This reaction was performed according to the procedure in Section S7.11.1 but with 100 mM of cytidine (97.3 mg, 0.4 mmol) and 50 mM sodium phosphate dibasic dihydrate salt (222.5 mg, 1.25 mmol).

A total of three cycles were performed. Formation of imidazole phosphate was observed in solution and remained present after drying the solution to a paste. Formation of cytidine-5'-monophosphate, cytidine-2'-monophosphate and cytidine-3'-monophosphate occurred during both the drying to the paste over a period of ~24 h and during the heating of the paste to 50 °C for ~24 h (Supplementary Figure 307, 308 and 309).

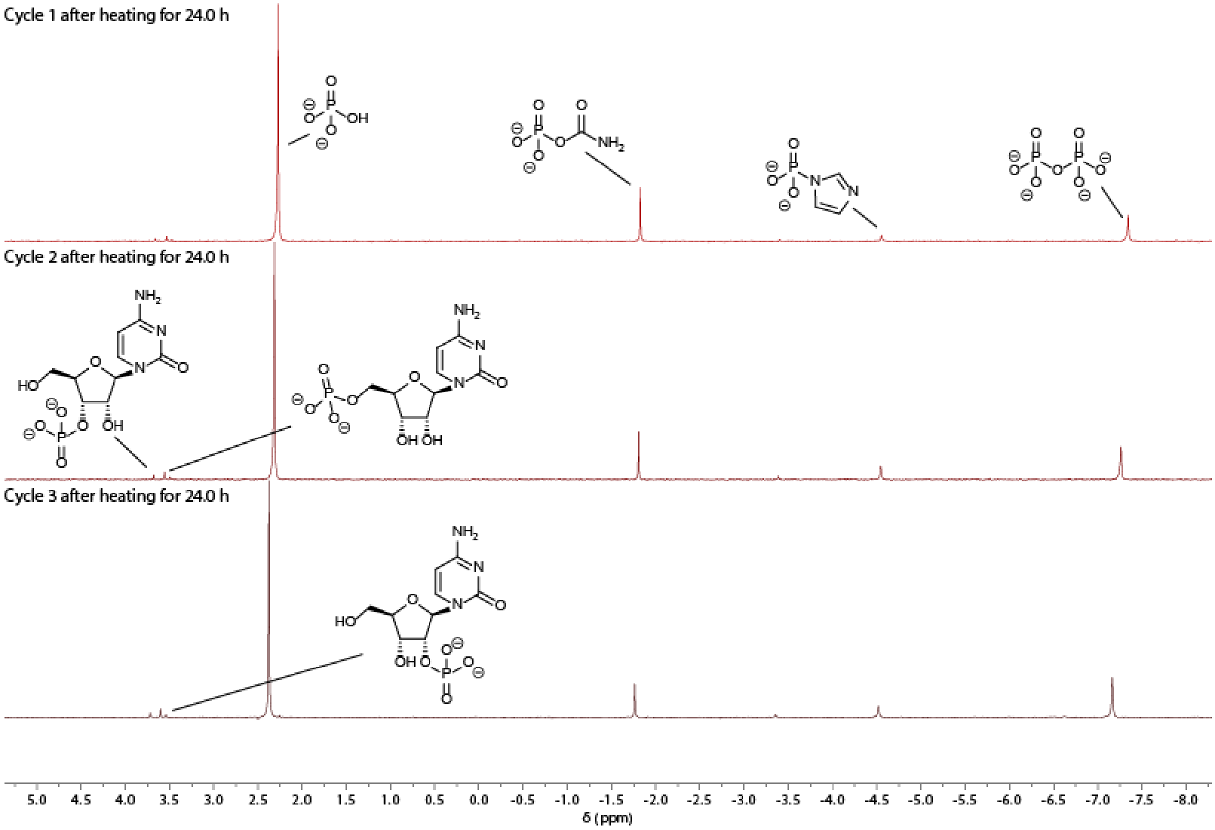

Supplementary Figure 307: Representative ^{31}P NMR spectra for the three cycles with 100 mM of cytidine, 100 mM potassium cyanate and 50 mM sodium phosphate in 100 mM imidazole buffer. For each cycle the ^{31}P NMR spectrum after the reaction had been dried to a paste and the ^{31}P NMR spectrum after heating the reaction to 50 °C for ~24 h are shown. All NMR spectra were measured at pH 7.3 in 9 : 1 H_2O : D_2O.

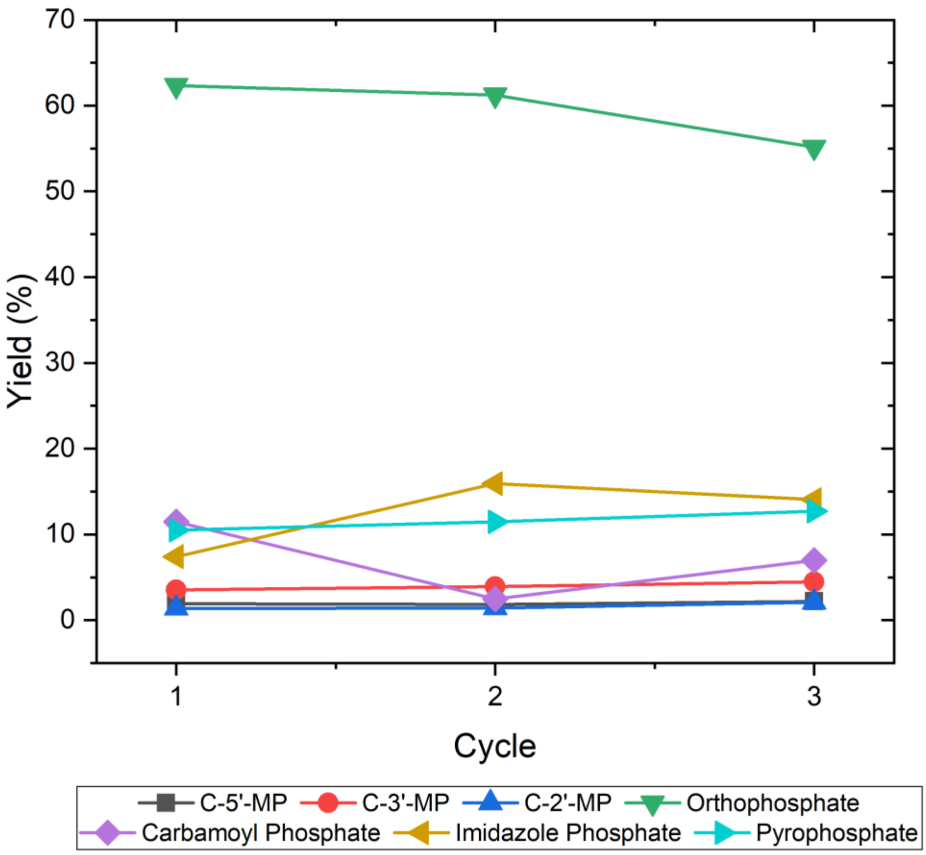

Supplementary Figure 308: Changes in yield for all phosphorous species over the course of three cycles for the reaction with 100 mM of cytidine, 100 mM potassium cyanate and 50 mM sodium phosphate in 100 mM imidazole buffer.

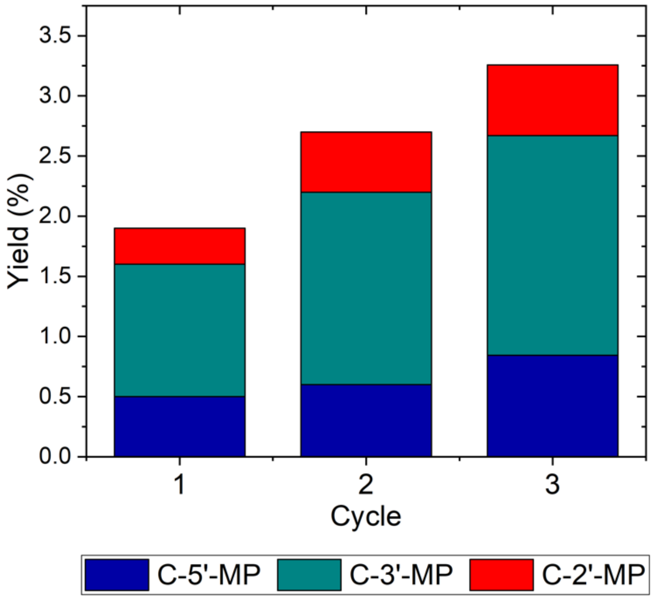

Supplementary Figure 309: Yield of cytidine-5'-monophosphate, cytidine-2'-monophosphate and cytidine-3'-monophosphate after each cycle for the reaction with 100 mM of cytidine, 100 mM potassium cyanate and 50 mM sodium phosphate in 100 mM imidazole buffer.

S7.12 Phosphorylation of glycerol with orthophosphate recycling at reagent concentrations of 10 – 100 mM

S7.12.1 Supplementary Experimental method for orthophosphate recycling with a wet/dry cycle at reagent concentrations of 10 – 100 mM

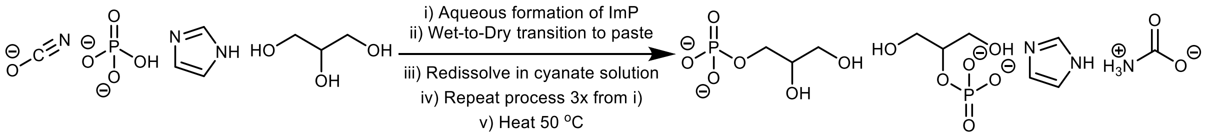

A 100 mM potassium cyanate stock solution was prepared with 203 mg (2.5 mmol) of potassium cyanate and 25 mL of 9 : 1 H_2O : D_2O. 5.0 mL of this solution was used to dissolve 17.0 mg (0.25 mmol) of imidazole, 44.5 mg (0.25 mmol) of sodium phosphate dibasic dihydrate salt and 46.0 mg (0.5 mmol) of glycerol to give a 100 mM potassium cyanate, 50 mM imidazole, 50 mM sodium phosphate and 100 mM glycerol solution. This solution was left for ~24 h and then poured into a petri dish (10 cm diameter) as a single puddle and left with the lid off to dry at 22 °C for 24 h. The resulting paste was then redissolved in 5.0 mL of 100 mM potassium cyanate in 9 : 1 H_2O : D_2O. Again this solution was left for ~24 h before being left to dry again. The reaction was followed by ^{31}P-NMR and ^{1}H-NMR spectroscopy. This cycle was repeated a further two times and then the pastes were scraped into an Eppendorf, 0.8 μL/mg of MilliQ water was added to rewet the paste and then the paste was heated on a thermoshaker at 50 °C and 600 rpm for a total of 31 h. After 7 h the pastes were dissolved in 9: 1 H_2O : D_2O and checked by ^{31}P NMR spectroscopy, imidazole phosphate was still present and therefore the solutions were left to dry again and then heated on the thermoshaker at 50 °C and 600 rpm for a further 24 h.

Reactions with other concentrations of reagents were conducted using an identical procedure.

S7.12.2 Phosphorylation of glycerol with orthophosphate recycling at [KOCN] = 100 mM, [Na_2HPO_4] = 50 mM, [imidazole] = 50 mM and [glycerol] = 100 mM

This reaction was performed according to the procedure in Section S7.12.1.

Formation of glycerol-1-phosphate and glycerol-2-phosphate occurred during the drying to the paste and during the heating of the paste to 50 °C at the end (Supplementary Figure 310, 311 and 312).

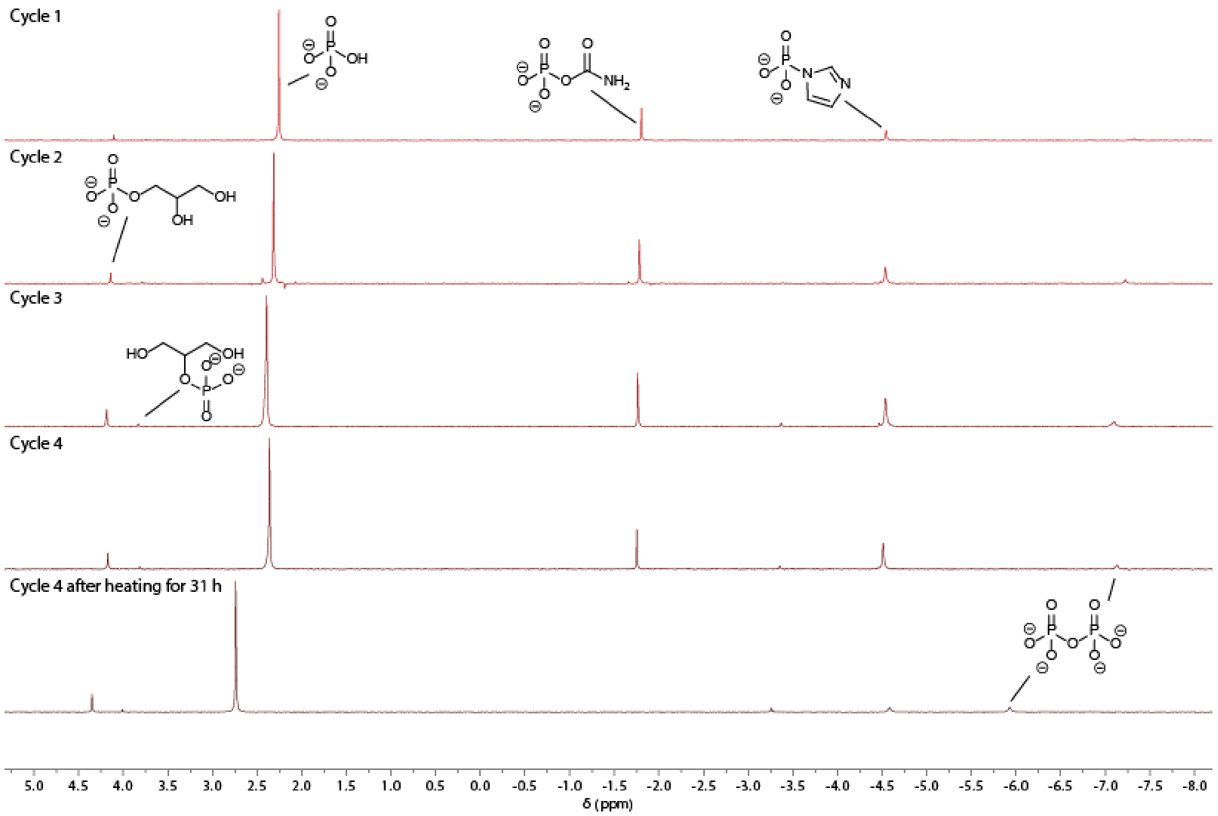

Supplementary Figure 310: Representative ^{31}P NMR spectra for the three cycles with 100 mM glycerol, 50 mM potassium cyanate and 50 mM sodium phosphate in 100 mM imidazole buffer. The ^{31}P NMR spectrum at the end of each cycle and the ^{31}P NMR spectrum after heating the paste to 50 °C for ~31 h are shown. All NMR spectra were measured in 9 : 1 H_2O : D_2O.

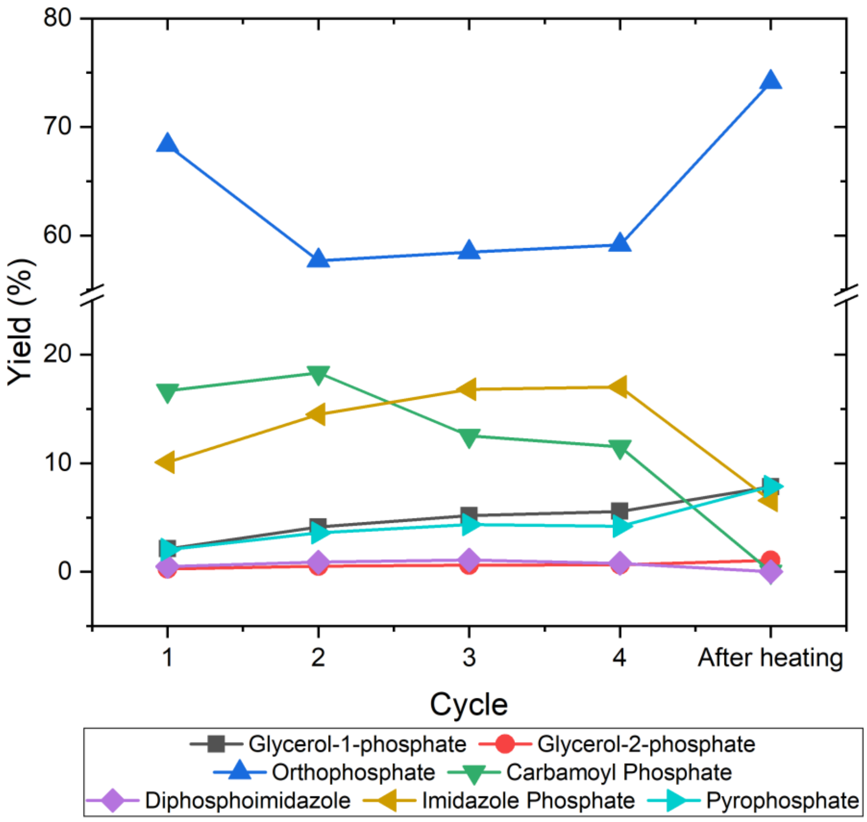

Supplementary Figure 311: Changes in yield for all phosphorous species over the course of three cycles for the reaction with 100 mM glycerol, 50 mM potassium cyanate and 50 mM sodium phosphate in 100 mM imidazole buffer.

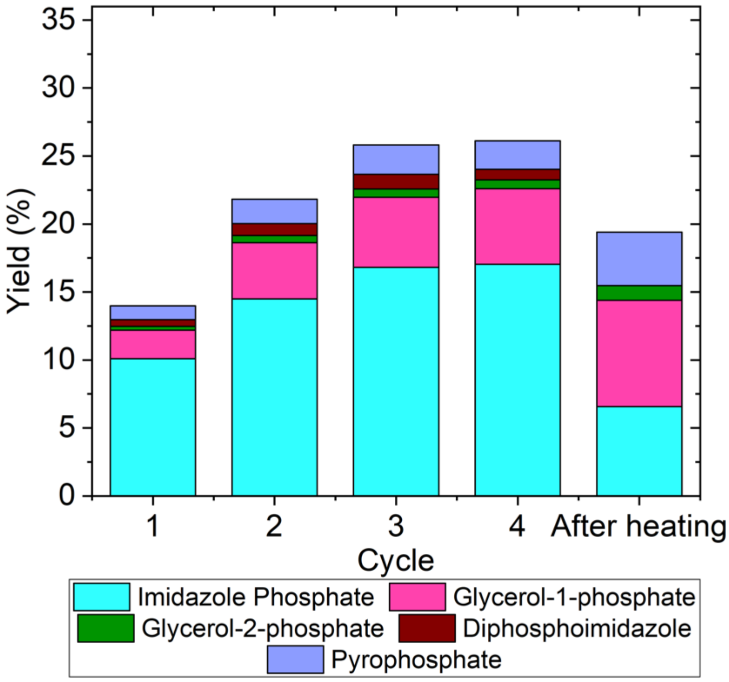

Supplementary Figure 312: Yield of glycerol-1-phosphate and glycerol-2-phosphate after each cycle for the reaction with 100 mM glycerol, 50 mM potassium cyanate and 50 mM sodium phosphate in 100 mM imidazole buffer.

S7.12.3 Phosphorylation of glycerol with orthophosphate recycling at [KOCN] = 50 mM, [Na_2HPO_4] = 50 mM, [imidazole] = 50 mM and [glycerol] = 50 mM

This reaction was performed according to the procedure in Section S7.12.1 but using a 50 mM potassium cyanate stock solution that was prepared with 81.1 mg (1.0 mmol) of potassium cyanate and 20 mL of 9 : 1 H_2O : D_2O. 5.0 mL of this solution was used to dissolve 17.0 mg (0.25 mmol) of imidazole, 44.5 mg (0.25 mmol) of sodium phosphate dibasic dihydrate salt and 23.0 (0.25 mmol) of glycerol to give a 50 mM potassium cyanate, 50 mM imidazole, 50 mM sodium phosphate and 50 mM glycerol solution.

Formation of glycerol-1-phosphate and glycerol-2-phosphate occurred during the drying to the paste and during the heating of the paste to 50 °C at the end (Supplementary Figure 313, 314 and 315).

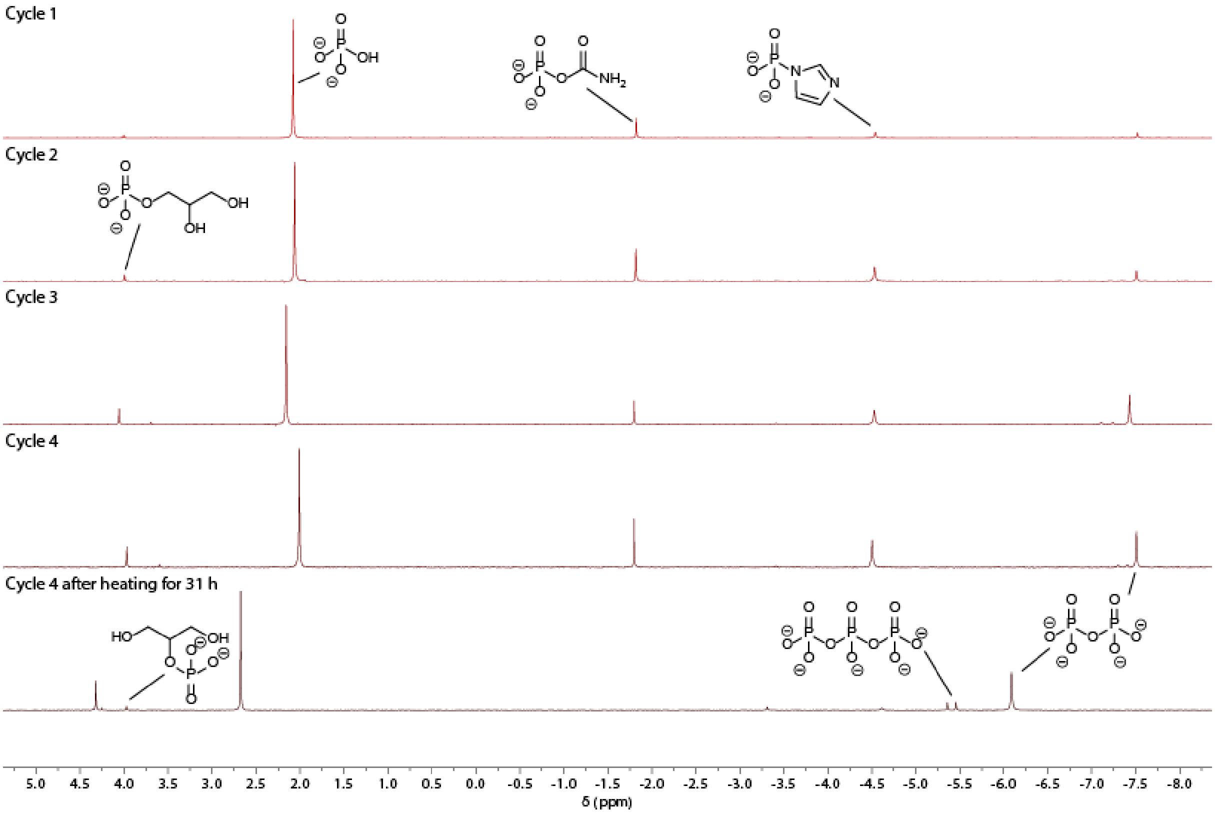

Supplementary Figure 313: Representative ^{31}P NMR spectra for the three cycles with 50 mM glycerol, 50 mM potassium cyanate and 50 mM sodium phosphate in 50 mM imidazole buffer. The ^{31}P NMR spectrum at the end of each cycle and the ^{31}P NMR spectrum after heating the paste to 50 °C for ~31 h are shown. All NMR spectra were measured in 9 : 1 H_2O : D_2O.

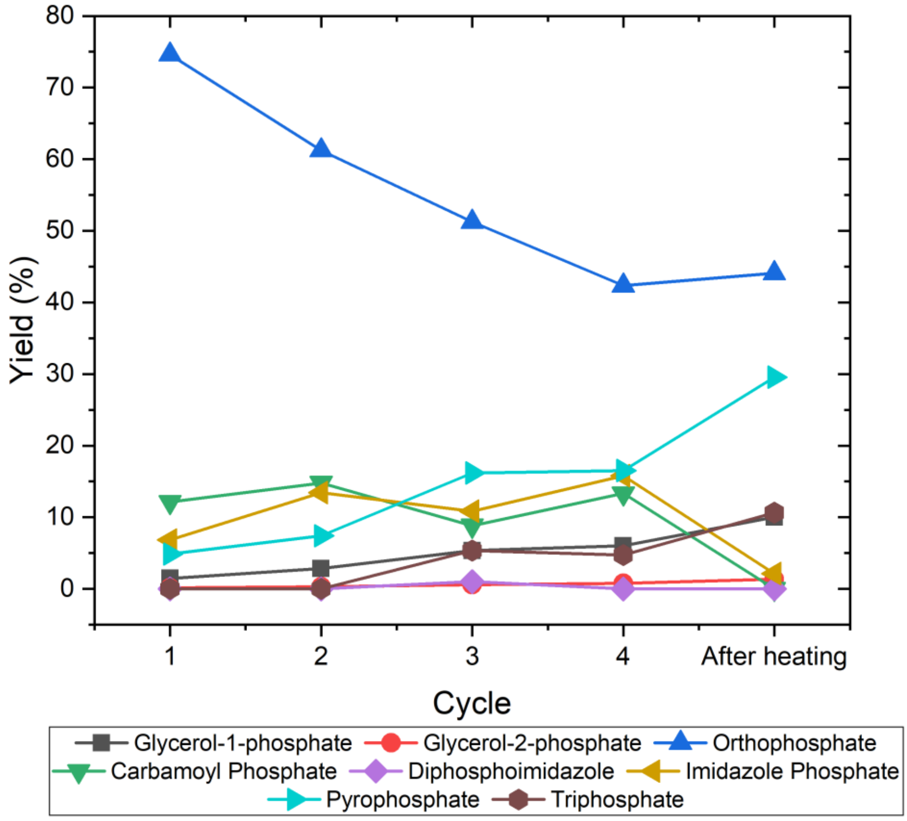

Supplementary Figure 314: Changes in yield for all phosphorous species over the course of three cycles for the reaction with 50 mM glycerol, 50 mM potassium cyanate and 50 mM sodium phosphate in 50 mM imidazole buffer.

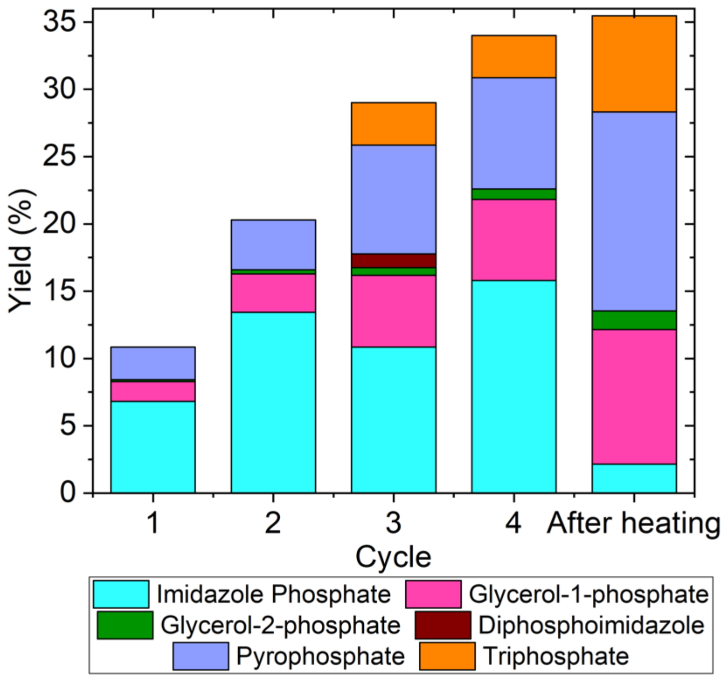

Supplementary Figure 315: Yield of glycerol-1-phosphate and glycerol-2-phosphate after each cycle for the reaction with 100 mM glycerol, 50 mM potassium cyanate and 50 mM sodium phosphate in 100 mM imidazole buffer.

S7.12.4 Phosphorylation of glycerol with orthophosphate recycling at [KOCN] = 20 mM, [Na_2HPO_4] = 20 mM, [imidazole] = 20 mM and [glycerol] = 20 mM

This reaction was performed according to the procedure in Section S7.12.1 but using a 50 mM potassium cyanate stock solution that was prepared with 24.3 mg (0.3 mmol) of potassium cyanate and 15 mL of 9 : 1 H_2O : D_2O. 5.0 mL of this solution was used to dissolve 6.8 mg (0.1 mmol) of imidazole, 17.8 mg (0.1 mmol) of sodium phosphate dibasic dihydrate salt and 23.0 (0.25 mmol) of glycerol to give a 20 mM potassium cyanate, 20 mM imidazole, 20 mM sodium phosphate and 20 mM glycerol solution.

Formation of glycerol-1-phosphate and glycerol-2-phosphate occurred during the drying to the paste and during the heating of the paste to 50 °C at the end (Supplementary Figure 316, 317 and 318).

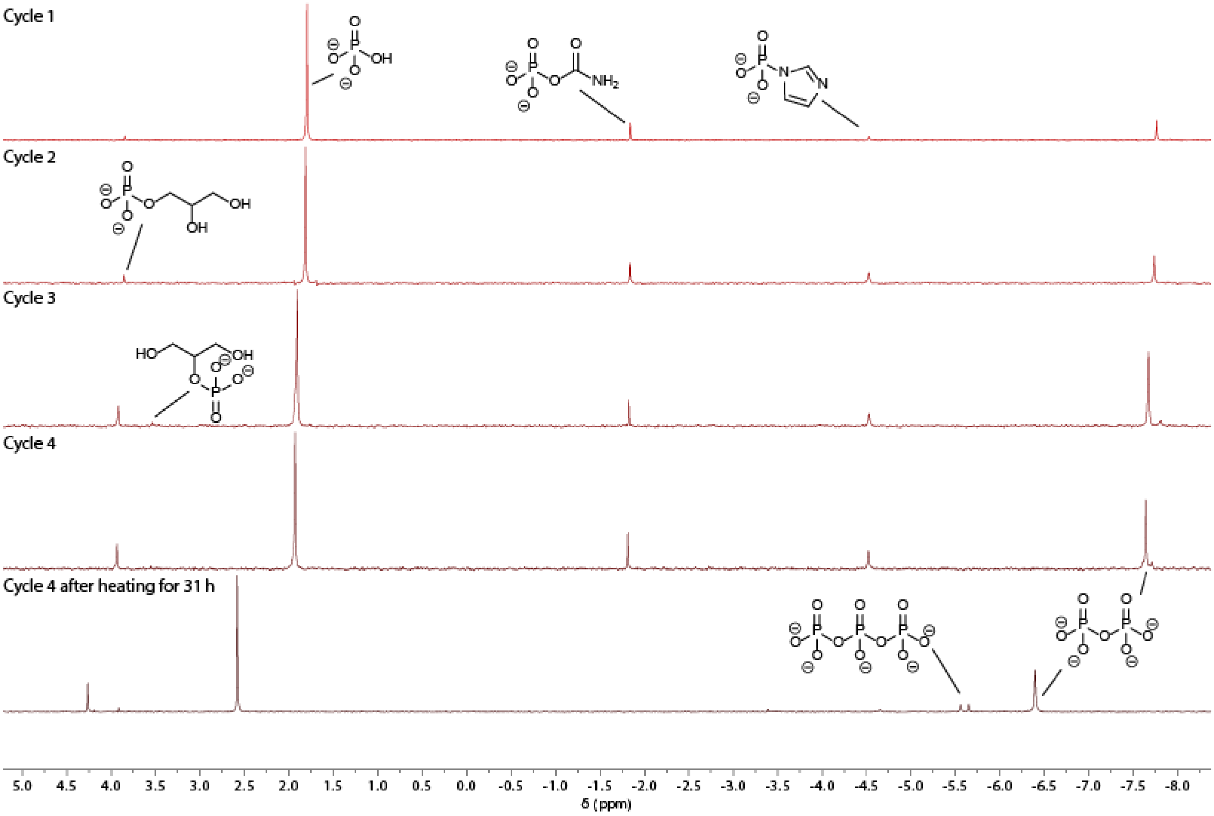

Supplementary Figure 316: Representative ^{31}P NMR spectra for the three cycles with 20 mM glycerol, 20 mM potassium cyanate and 20 mM sodium phosphate in 20 mM imidazole buffer. The ^{31}P NMR spectrum at the end of each cycle and the ^{31}P NMR spectrum after heating the paste to 50 °C for ~31 h are shown. All NMR spectra were measured in 9 : 1 H_2O : D_2O.

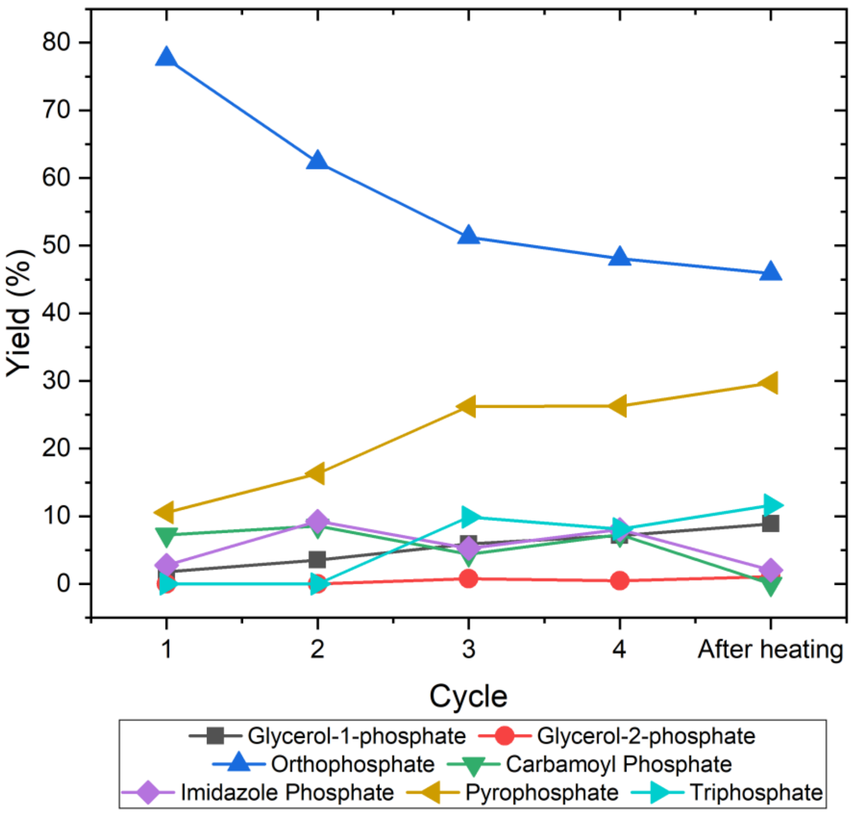

Supplementary Figure 317: Changes in yield for all phosphorous species over the course of three cycles for the reaction with 20 mM glycerol, 20 mM potassium cyanate and 20 mM sodium phosphate in 20 mM imidazole buffer.

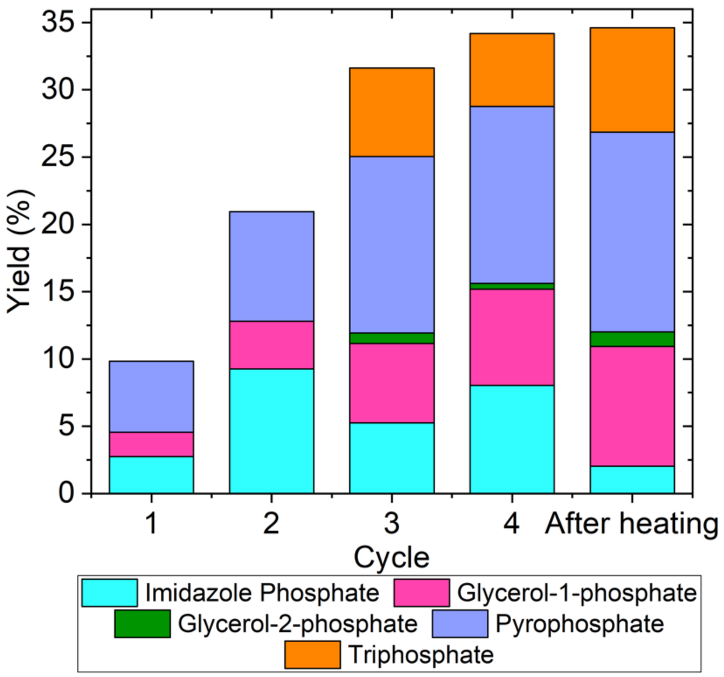

Supplementary Figure 318: Yield of glycerol-1-phosphate and glycerol-2-phosphate after each cycle for the reaction with 20 mM glycerol, 20 mM potassium cyanate and 20 mM sodium phosphate in 20 mM imidazole buffer.

S7.12.4 *Phosphorylation of glycerol with orthophosphate recycling at [KOCN] = 20 mM, [Na_2HPO_4] = 10 mM, [imidazole] = 10 mM and [glycerol] = 20 mM*

This reaction was performed according to the procedure in Section S7.12.1 but using a 50 mM potassium cyanate stock solution which was prepared with 24.3 mg (0.3 mmol) of potassium cyanate and 15 mL of 9 : 1 H_2O : D_2O. 5.0 mL of this solution was used to dissolve 3.4 mg (0.05 mmol) of imidazole, 8.9 mg (0.05 mmol) of sodium phosphate dibasic dihydrate salt and 9.2 (0.1 mmol) of glycerol to give a 20 mM potassium cyanate, 10 mM imidazole, 10 mM sodium phosphate and 20 mM glycerol solution.

Formation of glycerol-1-phosphate and glycerol-2-phosphate occurred during the drying to the paste and during the heating of the paste to 50 °C at the end (Supplementary Figure 319, 320 and 321).

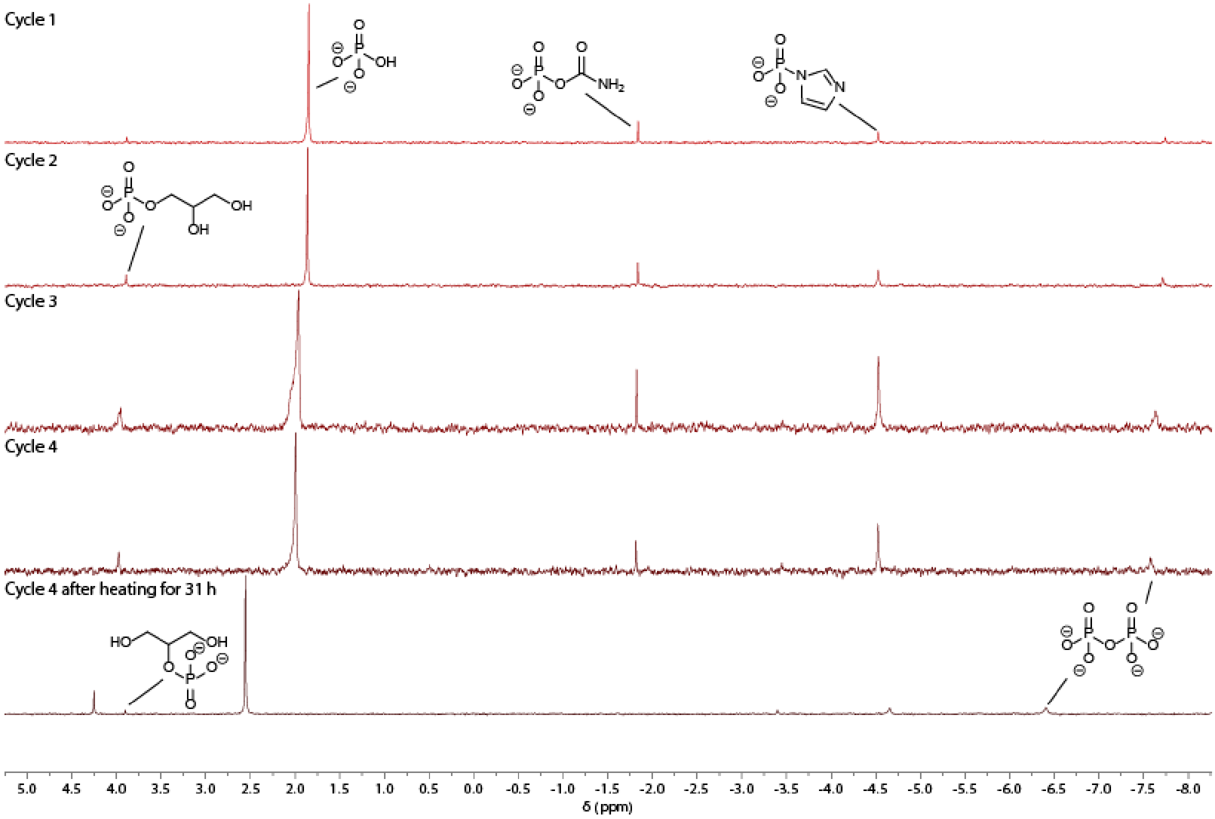

Supplementary Figure 319: Representative ^{31}P NMR spectra for the three cycles with 20 mM glycerol, 20 mM potassium cyanate and 10 mM sodium phosphate in 10 mM imidazole buffer. The ^{31}P NMR spectrum at the end of each cycle and the ^{31}P NMR spectrum after heating the paste to 50 °C for ~31 h are shown. All NMR spectra were measured in 9 : 1 H_2O : D_2O.

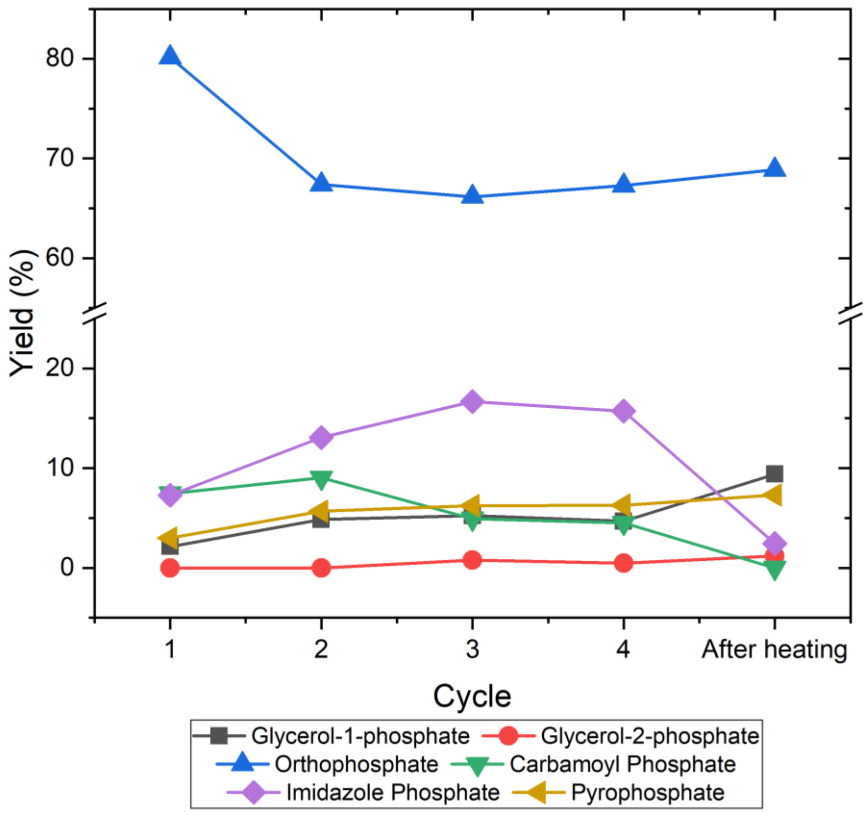

Supplementary Figure 320: Changes in yield for all phosphorous species over the course of three cycles for the reaction with 20 mM glycerol, 20 mM potassium cyanate and 10 mM sodium phosphate in 10 mM imidazole buffer.

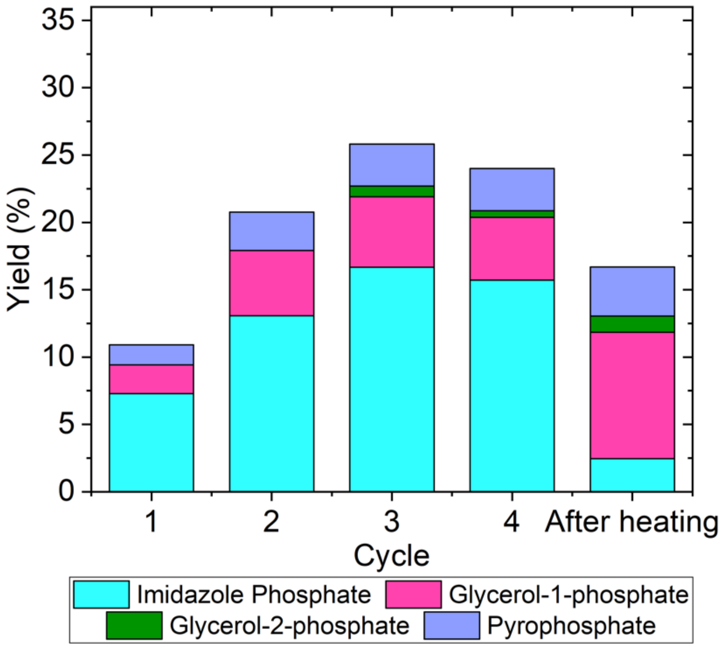

Supplementary Figure 321: Yield of glycerol-1-phosphate and glycerol-2-phosphate after each cycle for the reaction with 20 mM glycerol, 20 mM potassium cyanate and 10 mM sodium phosphate in 10 mM imidazole buffer.

S8 Synthesis of compounds for spiking

S8.1 Synthesis of phosphoserine methyl ester

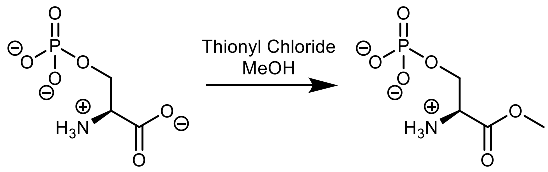

An adapted procedure from G. Fölsch, *Acta Chemica Scandinavica* **1966**, *20*, 459 – 473 was used to prepare phosphoserine methyl ester.

104 mg (0.56 mmol) of O-phosphoserine was added to a 50 mL round-bottom flask and partially dissolved in 1.46 mL of methanol – a small amount of the white solid remained. The round-bottom flask was placed under an inert atmosphere with argon. Thionyl chloride (400 μL, 5.4 mmol) was added dropwise to the solution which caused the remaining white solid to disappear. The solution turned a light-yellow colour during this step. The solution was left to stir overnight under an inert atmosphere. In the morning the solvent was removed *in-vacuo* to leave a yellow oil. The oil was dissolved in methanol and diethyl ether was added dropwise to precipitate out the target compound as a sticky white precipitate. The precipitate was filtered off and the solid washed with 3 x 650 μL diethyl ether. The solid was dried *in-vacuo* for 2 h. Mass of this solid is 128 mg (quantitative yield) ^{31}P NMR (202.5 MHz, 9 : 1 H_2O : D_2O): *phosphoserine methyl ester* δ (ppm) = – 1.24 (s, 1P). ^{1}H NMR (500.1 MHz, 9 : 1 H_2O : D_2O): *phosphoserine methyl ester* δ (ppm) = 4.33 (s, 1H), 4.19 (m, 2H), 3.79 (s, 1H).

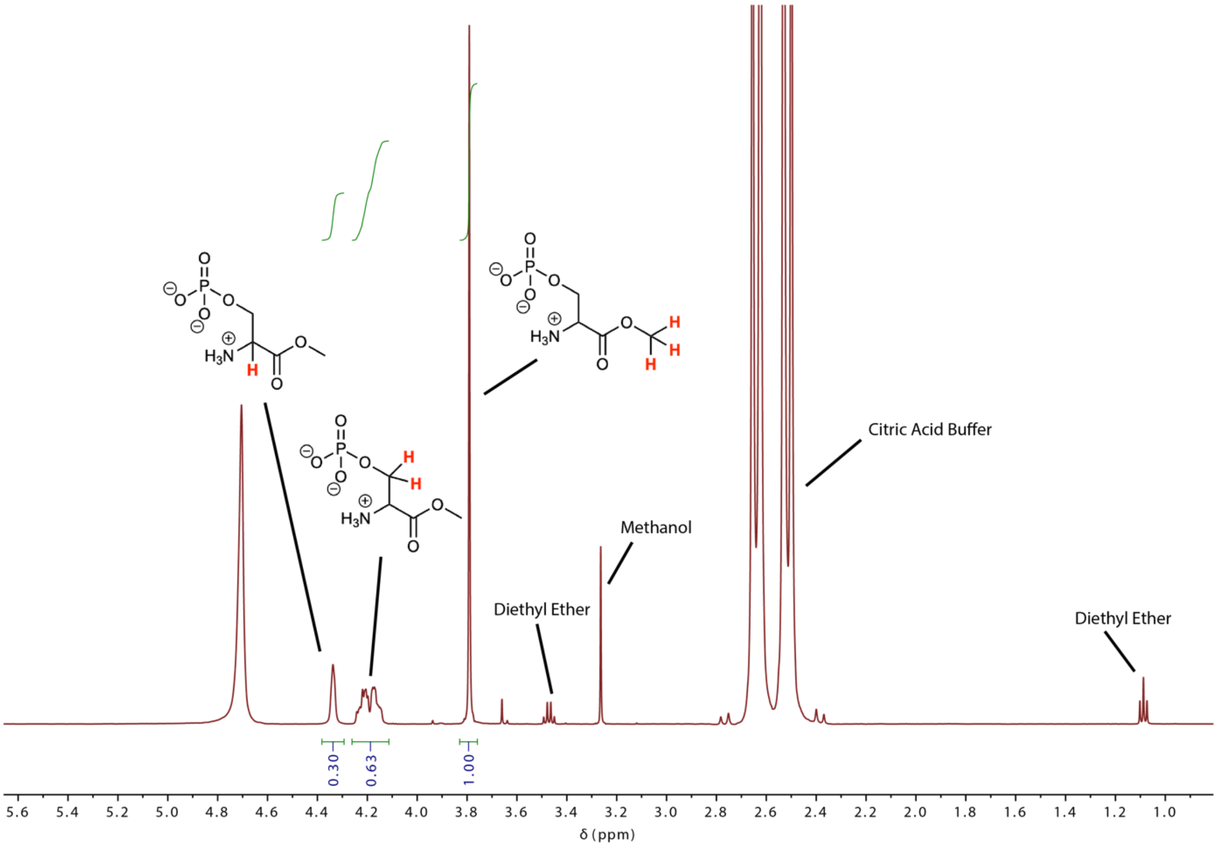

Supplementary Figure 322: ^{1}H NMR spectrum of phosphoserine methyl ester in 0.5 M citric acid buffer at pH 6.5 9 : 1 H_2O : D_2O.

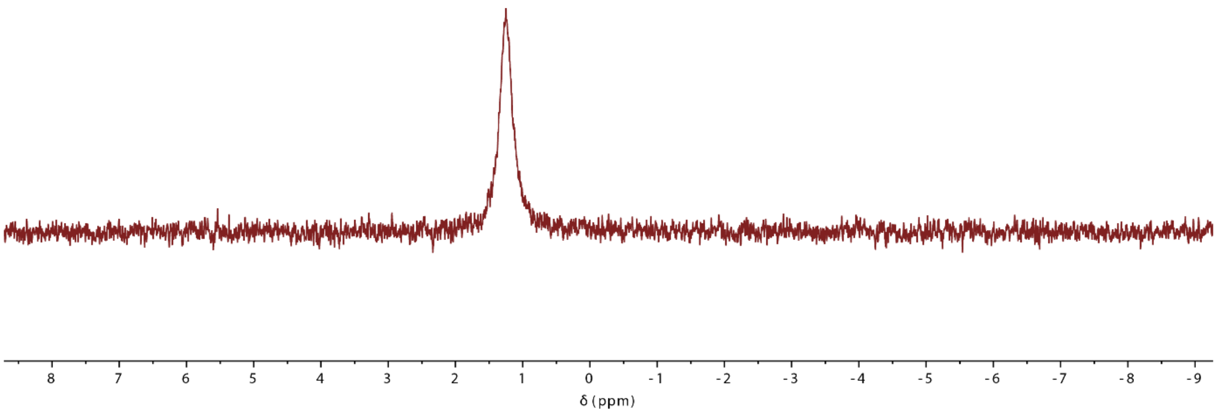

Supplementary Figure 323: ^{31}P NMR spectrum of phosphoserine methyl ester in 0.5 M citric acid buffer at pH 6.5 9 : 1 H_2O : D_2O.

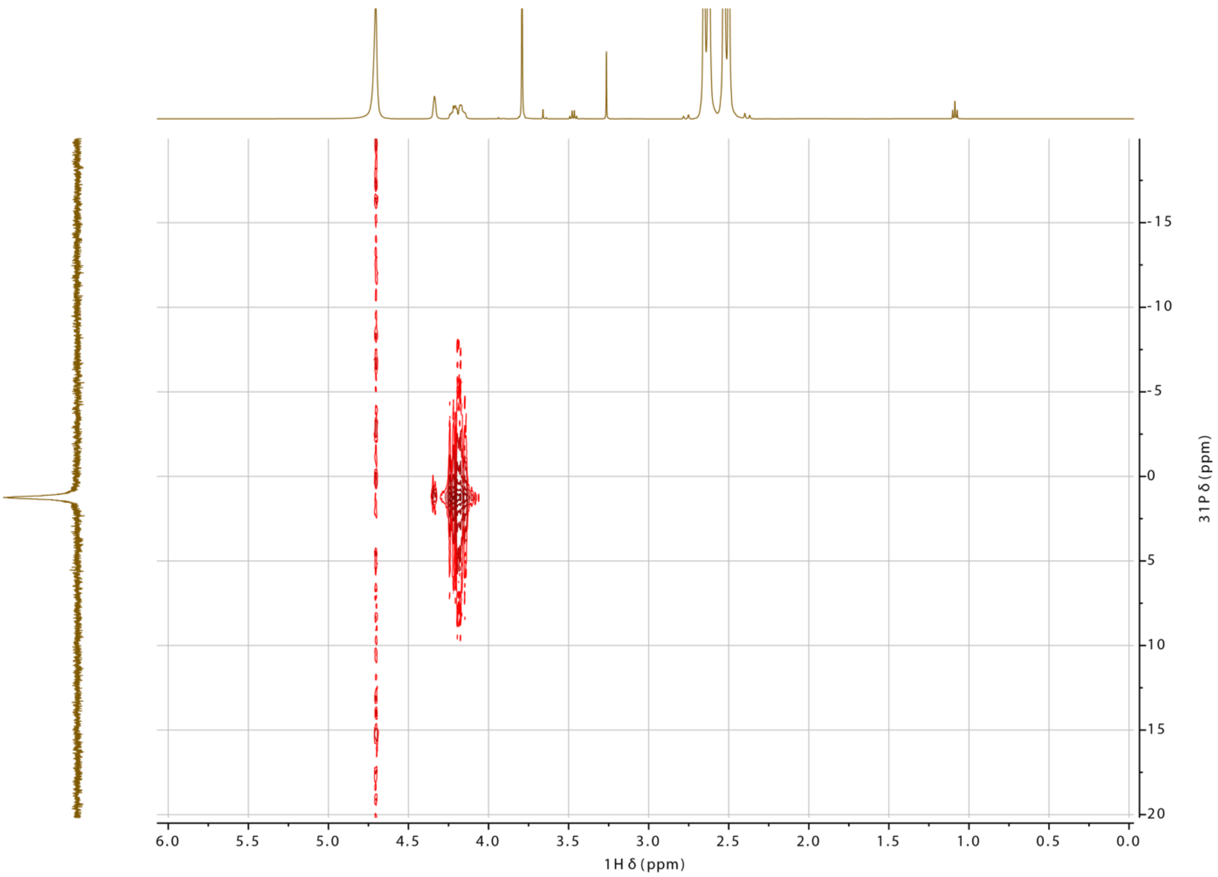

Supplementary Figure 324: ^{1}H ^{31}P HMBC NMR spectrum of phosphoserine methyl ester in 0.5 M citric acid buffer at pH 6.5 9 : 1 H_2O : D_2O.

S9 Supplementary References

1. Rosenberg, T. A simple preparation method for diphosphoimidazole. *Arch. Biochem. Biophys.* **105,** 315-318 (1964).
2. Cooperman, B. S. & Lloyd, G. J. Nucleophilic attack by zinc(II)-pyridine-2-carbaldoxime anion on phosphorylimidazole. Model for enzymic phosphate transfer. *J. Am. Chem. Soc.* **93,** 4883-4889 (1971).
3. Danger, G., Boiteau, L., Cottet, H. & Pascal, R. The Peptide Formation Mediated by cyanate Revisited. N-Carboxyanhydrides as Accessible Intermediates in the Decomposition of N-carbamoylamino acids. *J. Am. Chem. Soc.* **128,** 7412-7413 (2006).
